# Chirality Transfer in Gold(I)-Catalysed Direct Allylic Etherifications of Unactivated Alcohols: Experimental and Computational Study

**DOI:** 10.1002/chem.201501607

**Published:** 2015-08-06

**Authors:** Graeme Barker, David G Johnson, Paul C Young, Stuart A Macgregor, Ai-Lan Lee

**Affiliations:** Institute of Chemical Sciences, Heriot-Watt University Edinburgh EH14 4AS (UK)

**Keywords:** alcohols, allylations, asymmetric reactions, chirality transfer, gold

## Abstract

Gold(I)-catalysed direct allylic etherifications have been successfully carried out with chirality transfer to yield enantioenriched, γ-substituted secondary allylic ethers. Our investigations include a full substrate-scope screen to ascertain substituent effects on the regioselectivity, stereoselectivity and efficiency of chirality transfer, as well as control experiments to elucidate the mechanistic subtleties of the chirality-transfer process. Crucially, addition of molecular sieves was found to be necessary to ensure efficient and general chirality transfer. Computational studies suggest that the efficiency of chirality transfer is linked to the aggregation of the alcohol nucleophile around the reactive π-bound Au–allylic ether complex. With a single alcohol nucleophile, a high degree of chirality transfer is predicted. However, if three alcohols are present, alternative proton transfer chain mechanisms that erode the efficiency of chirality transfer become competitive.

## Introduction

α-Chiral ethers are present in many natural products, biologically active molecules and synthetic intermediates.^1^ Therefore, much effort has been directed towards efficient routes to enantiomerically enriched chiral ethers through allylic etherification reactions.^2^ Within this context, there is currently ongoing interest in utilising *unactivated* allylic alcohol electrophiles in transition-metal-catalysed allylations of various nucleophiles,^3^ as the use of unactivated allylic alcohol electrophiles reduces the number of synthetic steps required (by virtue of not requiring prior derivatisation) and minimises byproduct formation. In terms of asymmetric intermolecular etherifications, a recent notable advance by Carreira et al. uses Ir catalysis to effect allylic etherifications on secondary allylic alcohols through formal S_N_2 selectivity.^4^

**Scheme 1:**
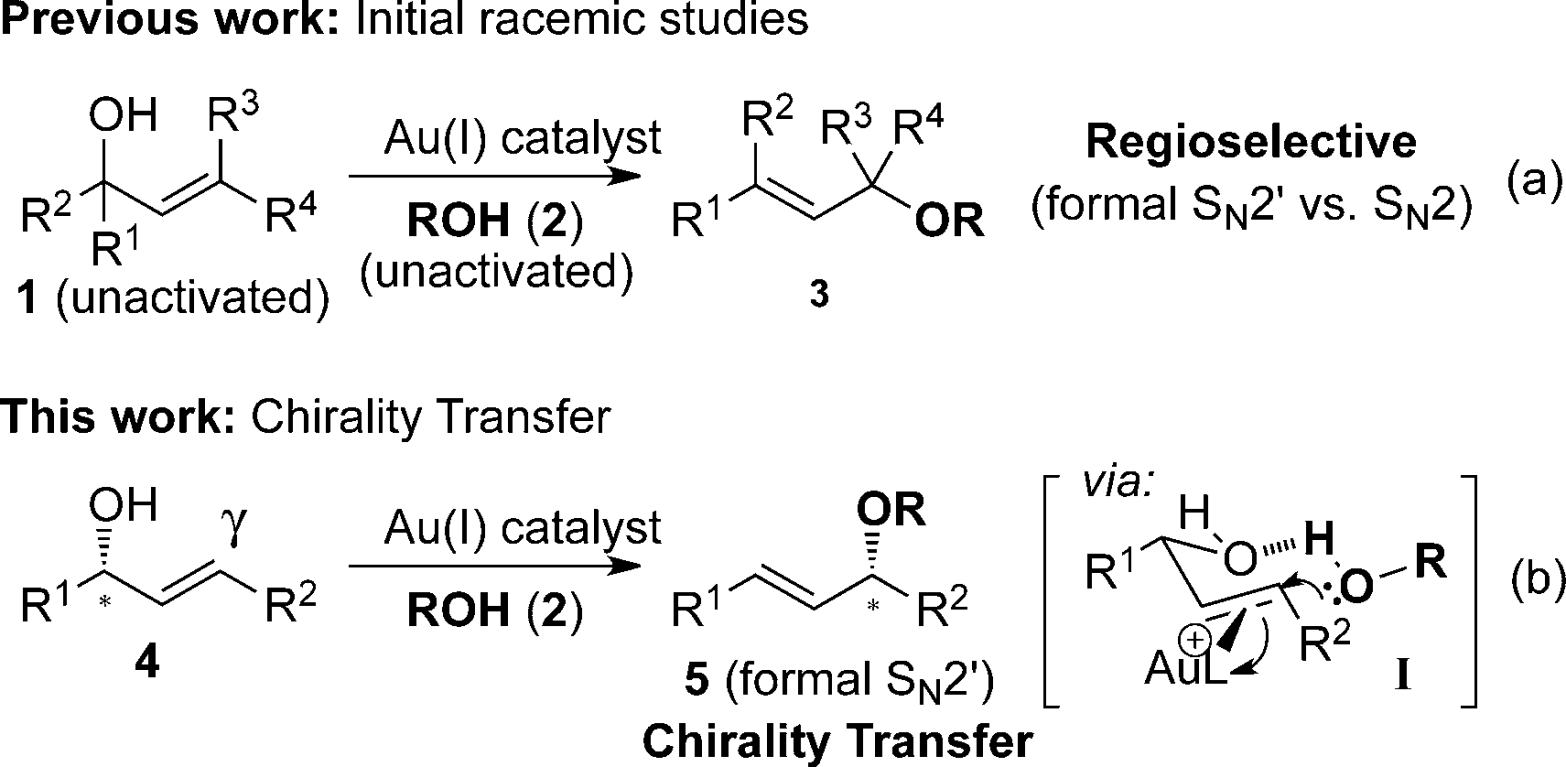
Previous work (racemic studies) and current chirality-transfer target.

One of the key research efforts within our group has been to develop gold-catalysed^5^ regioselective methods towards allylic ethers^6^ and allylic thioethers.^7^ Within this context, we recently developed a mild and air-stable gold(I)-catalysed direct allylic etherification of allylic alcohols.^8^ This dehydrative formal S_N_2′ procedure^9^ requires neither the allylic alcohol electrophile nor the alcohol nucleophile to be activated (either to install a leaving group in the former or form an alkoxide in the latter), leading to mild reaction conditions that are tolerant of various functional groups as well as air and moisture (Scheme 1 a).3a, 10 We were keen to extend this methodology to asymmetric methods by investigating various chiral, non-racemic γ-substituted substrates, which should be amenable to chirality transfer. In theory, an enantioenriched chiral allylic alcohol with γ-substitution (e.g., **4**, Scheme 1), which is easily accessible in good enantioselectivities by Sharpless kinetic resolution^11^ or enzyme resolution,^12^ should be able to transfer its chirality^13^ to the allylic ether product **5**, especially if a 6-membered ring hydrogen-bonded intermediate **I** is involved (Scheme 1 b). Access to chiral, non-racemic α,γ-disubstituted allylic ethers such as **5** from unactivated alcohols also nicely complements recent Ir-catalysed allylation methods by Carreira et al.,4a which are confined to formation of unsubstituted secondary allylic ethers (R^1^=H). It should be noted that shortly after our initial communication,8a Mukherjee and Widenhoefer disclosed an independent report on the same reaction.^14^ Using a different set of catalysts and conditions, they carried out a substrate-scope study on the racemic reaction. In addition, they also elegantly show one example of a chirality-transfer reaction (see below). However, as the substrate scope of their chirality-transfer reaction was not reported and there was room for improvement with regards to the regioselectivity (5:1 of formal S_N_2′/S_N_2 **5**/**6**), we decided that it was still important to continue with our independent studies. These are reported here, and include optimisation to give greatly improved regioselectivities, full substrate-scope studies and experimental and computational mechanistic investigations.

## Results and Discussion

Our investigation began with the optimisation of reaction conditions to improve the regioselectivity for allylic etherifications, using secondary allylic alcohol **4 a** as a model substrate (Table 1). Our previously reported conditions provided a poor 2:1 ratio of formal S_N_2′/S_N_2 (**5 aa**/**6 aa**, entry 1), which needed to be improved drastically before chirality transfers could be investigated. During our optimisation, we discovered that addition of molecular sieves (MS) to the reaction mixture greatly improved the selectivity, exclusively yielding the formal S_N_2′ product **5 aa** (entries 2–8). 3 Å MS provided slightly higher yields compared to 4 Å MS (entry 2 vs. entry 5), however, the yields were modest when only 5 mol % of gold catalyst was employed (42 % and 56 %, respectively). Portion-wise addition of gold catalyst (2×5 mol %) greatly improved the yields (entries 3 and 6), but addition of another portion of molecular sieves makes little difference (entry 3 vs. entry 4). Finally, as a compromise between shorter reaction times and acceptable yield, we settled for the protocol shown in entry 8 as our optimised conditions for investigating the chirality-transfer reaction. Note that under these newly optimised conditions, the formal S_N_2′ product **5** is formed exclusively for all subsequent substrate-scope screens (Tables 2 and2, 3).

**Table 1:** Optimisation of catalytic conditions for secondary allylic alcohols.

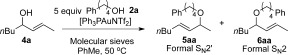					
Entry	Mol % Cat.	*t* [h]	MS	Results^[a]^
1	10	48	None	62 %, 2:1 **5**/**6**, 10:1 *E*/*Z*
2	5	67	4 Å MS	42 %, >20:1 **5**/**6**, 9:1 *E*/*Z*
3	2×5	24+39	4 Å MS	84 %, >20:1 **5**/**6**, 9:1 *E*/*Z*
4	2×5	24+39	2×4 Å MS	81 %, >20:1 **5**/**6**, 8:1 *E*/*Z*
5	5	66	3 Å MS	56 %, >20:1 **5**/**6**, 9:1 *E*/*Z*
6	2×5	20+20	3 Å MS	90 %, >20:1 **5**/**6**, 7:1 *E*/*Z*
7	2×2.5	20+20	3 Å MS	67 %, >20:1 **5**/**6**, 10:1 *E*/*Z*
8	2×5	8+16	3 Å MS	84 %, >20:1 **5**/**6**, 7:1 *E*/*Z*

[a] Isolated yields. **5**/**6** and *E*/*Z* ratios determined by ^1^H NMR analysis.

With these optimised conditions in hand, we turned our attention to effecting chirality transfer in etherifications of a range of enantioenriched allylic alcohols (Table 2). For this assay, alcohol **2 b** was chosen as the nucleophile for ease of chiral stationary phase (CSP)-HPLC enantiomer separation in the product. Gratifyingly, our first attempt with enantioenriched *n-*butyl allylic alcohol (*R*)-**4 a** gave the desired product (*E*)-**5 ab** in 88:12 e.r. from >99:1 e.r. of the starting material. The minor (*Z*)-isomer (*Z*)-**5 ab** was obtained in 84:16 e.r. (entry 1). Alternatively, starting with (*Z*)-allylic alcohol **4 b**, the opposite enantiomer of the product could be obtained with good transfer of chirality (entry 2). It should be noted, however, that the *Z*-allylic alcohol starting materials (e.g., **4 b**, entry 2) are more difficult to access in high e.r., resulting in poorer e.r. of product **5 bb**, despite displaying a good degree of chirality transfer (81:19→76:24 e.r.). Reversing the substituents at the α- and γ-positions of the allylic alcohol similarly gave high yield of product **5 cb** with a high degree of chirality transfer (entry 3). Replacing the *n*-butyl substituent with the sterically more demanding cyclohexyl also works well with the Cy at the *α*-position, but only moderately at the γ-position (entries 4–5). To verify that a more sterically hindered substituent at the γ-position causes a drop in chirality transfer, allylic alcohol **4 f**, with an *i*Pr at the γ-position was investigated. Indeed, moderate chirality transfer of >99:1→77:23 e.r. is observed (entry 6).

**Table 2:** Allylic alcohol scope.

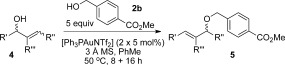					
Entry	Allylic alcohol	Product	Result^[a]^
1	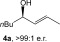	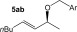	78 %, 7:1 *E*/*Z*
			88:12 e.r. (*E*)^[b]^
			16:84 e.r. (*Z*)^[b]^
2	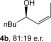	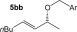	71 %, 11:1 *E*/*Z*
			76:24 e.r. (*E*)^[b]^
			16:84 e.r. (*Z*)^[b]^
3	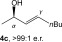	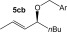	78 %, 5:1 *E*/*Z*
			98:2 e.r. (*E*)^[b]^
			16:84 e.r. (*Z*)^[b]^
4	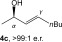	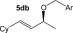	77 %, 10:1 *E*/*Z*
			89:11 e.r. (*E*)^[c]^
			46:54 e.r. (*Z*)^[c]^
5	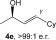	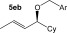	80 %, 9:1 *E*/*Z*
			77:23 e.r. (*E*)^[c]^
			47:53 e.r. (*Z*)^[c]^
6	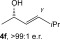	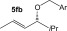	81 %, 5:1 *E*/*Z*
			77:23 e.r. (*E*)^[c]^
			13:87 e.r. (*Z*)^[c]^
7	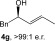	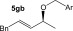	74 %,>20:1 *E*/*Z*
			99:1 e.r. (*E*)^[c]^
8	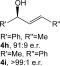	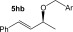	from **4 h**: 67 %, 14:1 *E*/*Z* racemic
			from **4 i**: 73 %, 12:1 *E*/*Z* racemic
9	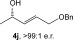	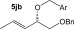	59 %, 6:1 *E*/*Z*
			98:2 e.r. (*E*)^[c]^
			38:62 e.r. (*Z*)^[c]^
10	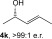	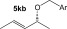	79 %, 9:1 *E*/*Z*
			71:29 e.r. (*E*)^[c,d]^
11	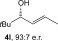	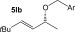	71 %, >20:1 *E*/*Z*
			67:33 e.r. (*E*)^[c]^
12	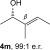	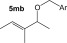	72 %, >20:1 *E*/*Z*
			51:49 e.r. (*E*)^[c]^
13	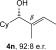	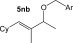	62 %, >20:1 *E*/*Z*
			53:47 e.r. (*E*)^[c]^

[a] Isolated yields. >20:1 **5**/**6** (formal S_N_2′/formal S_N_2) by ^1^H NMR analysis where applicable. *E*/*Z* ratios determined by ^1^H NMR analysis. [b] Determined by CSP-HPLC of a derivative. [c] Determined by CSP-HPLC. [d] E.r. of *Z* isomer not determined.

Benzyl-substituted allylic alcohol **4 g** provided the best result in this assay, with excellent chirality transfer (>99:1→99:1 e.r.) and >20:1 *E*/*Z* observed (entry 7). Replacing the benzyl in **4 g** (entry 7) with a Ph substituent (**4 h**, entry 8) causes a drastic change (racemic product **5 hb**). Unlike with alkyl substituents (entries 1 vs. 3 and entries 4 vs. 5), swapping the Ph substituent around now forms the same product (**4 i**→**5 hb**, entry 8), so aryl substituents appear to be detrimental to both chirality transfer and formal S_N_2′ selectivity. Next, we decided to compare our procedure with substrate **4 j**, which was the substrate chosen by Widenhoefer et al. in their studies (entry 9).^14^ The chirality transfer is once again excellent (99:1→98:2 e.r.). It should be noted that using our newly optimised conditions, etherification proceeds with significantly higher formal S_N_2′ selectivity (>20:1 vs. 5:1 formal S_N_2′/S_N_2).

Certain substituents on the allylic alcohol substrate were found to cause the chirality transfer to proceed moderately to poorly (entries 10–13). For example, dimethyl allylic alcohol **4 k** gave a high degree of racemisation (71:29 e.r. of *E*-**5 kb** from **4 k** of >99:1 e.r.); likewise, increasing the steric bulk of the substituent at the alcohol centre to *tert-*butyl alcohol **4 l** also led to some racemisation during reaction (entry 11). Substrates with β-substituents performed the worst: **4 m** and **4 n** both give excellent >20:1 *E*/*Z* ratios, but almost complete racemisation under these conditions (entries 12–13) and are therefore not suitable substrates for chirality transfer.

We next turned our attention to investigating the tolerance of a range of different nucleophile alcohols by using model allylic alcohol substrate (*R*)-**4 a** (Table 3).^15^ Although *para*-bromobenzyl alcohol **2 d** (entry 3) gave a comparable result to the original nucleophile alcohol **2 b**, benzyl alcohol **2 c** and *para*-methoxybenzyl alcohol **2 e** yielded products **5 ac** and **5 ae**, respectively, with a greater degree of chirality transfer (97:3 e.r., entry 2 and >95:5 e.r., entry 4).^16^ Furfuryl alcohol **2 f** was also tolerated, though with a reduction of enantioenrichment in product **5 af** (entry 5). We then turned our attention to alkyl alcohols. Extending the alkyl chain of benzyl alcohol by two methylene units preserved yield, formal S_N_2′/S_N_2 and *E*/*Z* alkene selectivity as well as chirality-transfer efficiency (entry 2 vs. entry 6). Next, we set out to test functional group tolerance. Pleasingly, trifluoromethyl substitution of the nucleophile was tolerated (entry 7) as were haloalkanes (entry 8) and unprotected terminal alkenes (entry 9). When utilising diol **2 k**, reaction occurred exclusively through the primary alcohol to give **5 ak** in high yield and selectivity (entry 10). No product from subsequent reaction through the tertiary alcohol was observed. Acid-labile groups such as acetals **2 l** and **2 m** were also found to be compatible with the reaction (entries 11 and 12). Finally, we demonstrated that the reaction also proceeds very well (>99:1 e.r.) using a more hindered secondary nucleophile alcohol such as cyclohexanol **2 n** (entry 13).

**Table 3:** Nucleophile alcohol scope.

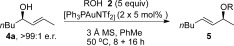			
Entry	Nucleophile2	Product	Result^[a]^
1	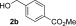	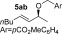	78 %, 7:1 *E*/*Z*
			88:12 e.r. (*E*)^[b]^
			21:79 e.r. (*Z*)^[b]^
2	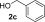	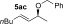	82 %,10:1 *E*/*Z*
			97:3 e.r. (*E*)^[c]^
			<1:99 e.r. (*Z*)^[c]^
3	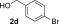	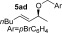	73 %, 6:1 *E*/*Z*
			87:13 e.r. (*E*)^[b]^
			25:75 e.r. (*Z*)^[b]^
4	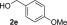	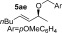	84 %,10:1 *E*/*Z*
			>95:5 e.r. (*E*)^[d]^
			<5:95 e.r. (*Z*)^[d]^
5	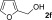	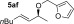	76 %, 4:1 *E*/*Z*
			86:14 e.r. (*E*)^[c]^
			9:91 e.r. (*Z*)^[c]^
6	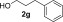	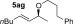	76 %,10:1 *E*/*Z*
			98:2 e.r. (*E*)^[c]^
			4:96 e.r. (*Z*)^[c]^
7	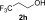	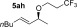	74 %, 2:1 *E*/*Z*
			93:7 e.r. (*E*)^[e]^
			29:71 e.r. (*Z*)^[e]^
8	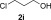	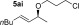	67 %, 6:1 *E*/*Z*
			94:6 e.r. (*E*)^[e]^
			35:65 e.r. (*Z*)^[e]^
9^[f]^	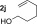	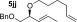	71 %, 6:1 *E*/*Z*
			98:2 e.r. (*E*)^[c]^
			14:86 e.r. (*Z*)^[c]^
10	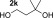	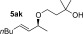	81 %, 5:1 *E*/*Z*
			86:14 (*E*)^[c]^
			9:91 e.r. (*Z*)^[c]^
11	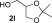	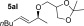	83 %, 1:1.1 d.r.
			>20:1 *E*/*Z*
			98:2 e.r. (d 1)^[e]^
			97:3 e.r. (d 2)^[e]^
12	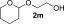	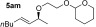	77 %,>20:1 d.r.
			10:1 *E*/*Z*
			93:7 e.r. (*E*)^[c]^
			10:90 e.r. (*Z*)^[c]^
13^[g]^	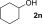	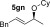	76 %,14:1 *E*/*Z*
			>99:1 e.r. (*E*)^[c]^
			7:93 e.r. (*Z*)^[c]^

[a] Isolated yields, >20:1 **5**/**6** (formal S_N_2′/formal S_N_2) by ^1^H NMR analysis*. E*/*Z* ratios and d.r. values determined by ^1^H NMR analysis. [b] Determined by CSP-HPLC of a derivative. [c] Determined by CSP-HPLC. [d] Determined by chiral shift ^1^H NMR spectroscopy. [e] Determined by CSP-GC. [f] Using allylic alcohol (*S*)-**4 j**, >99:1 e.r. [g] Using allylic alcohol (*R*)-**4 g**, >99:1 e.r.

**Scheme 2:**
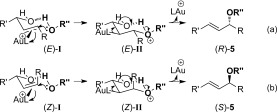
Proposed mechanism for successful chirality transfer and stereospecificity.

The mechanism that we originally proposed for the allylic etherification reaction8a can also account for the chirality transfer and stereospecificity of the reaction (Scheme 1). As gold(I) is an excellent π-Lewis acid,5e it is likely to activate the alkene functionality in the allylic alcohol towards attack by an external alcohol nucleophile (**I**, Scheme 2).10f Demetallation and elimination of water (enabled by intramolecular hydrogen-bonding, **II**) will then regenerate the catalyst and produce the desired allylic ether product **5**. A tightly bound chair-like 6-membered ring transition state^17^ is required for efficient chirality transfer, and also accounts for the stereospecificity of the *E* and *Z* isomers. As shown in Scheme 1 a, the *E* isomer has its substituent R in the equatorial position, whereas the *Z* isomer has R axial (Scheme 2 b), thus leading to the different stereochemical outcomes. Having the substituent R′ equatorial also accounts for the *E*-selectivity of the reaction.

It is clear from the proposed mechanism in Scheme 1 that the hydrogen-bonded 6-membered transition state **I** is crucial for the chirality transfer, and also the *E*-selectivity. Any erosion of *ee* could therefore be attributed to the disruption of this hydrogen-bonding pattern that would allow the reaction to occur without this 6-membered transition state **I**. One such mechanism is explored in the computational section below (see Scheme 9). However, a second possibility for erosion of *ee* is the racemisation of the product **5** through isomerisation between the formal S_N_2′ (**5**) and formal S_N_2 (**6**) products, catalysed by gold(I).6b, c, 17 Indeed, during our related studies using thiols for thioetherification reactions, chirality transfer does *not* occur in the thioetherification reactions.7a, 18 Experimental and computational studies showed that the racemisation is due to isomerisation between the formal S_N_2′ and S_N_2 thioether products. Clearly, using alcohol instead of thiol as a nucleophile allows for successful chirality transfer, except in certain substrates, such as β-substituted **4 m** and **4 n** (entries 12–13, Table 2). Therefore, we carried out several control experiments to ascertain the role of isomerisation of the products **5** and **6** in the erosion of *ee*.

**Scheme 3:**
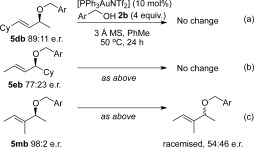
Resubjection of products 5 db, 5 eb and 5 mb to the reaction conditions.

Firstly, product **5 db** (entry 4, Table 2) was resubjected to the reaction conditions and no change was observed after 24 h (Scheme 3 a). Next, **5 eb** was investigated, as this product was formed with only moderate chirality transfer (77:23 e.r., entry 5, Table 2). Once again, no change was observed upon resubjection to the reaction conditions (Scheme 3 b). Finally, product **5 mb**, which is formed as a racemic mixture by our method (entry 12, Table 2) was investigated. This species, obtained in 98:2 e.r. by an alternative route,^19^ was found to racemise upon resubjection to the reaction conditions (Scheme 3 c).^20^ From these results, it appears that slight erosion of *ee* is *not* caused by isomerisation/racemisation of the product (see later and Scheme 9 for plausible racemisation mechanism). However, instances of complete racemisation, such as the formation of **5 mb** and **5 nb** from β-substituted **4 m** and **4 n**, respectively, could be due to isomerisation and racemisation of the products under the reaction conditions.

**Scheme 4:**
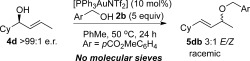
Allylic etherification of 4 d without molecular sieves results in racemic product.

Next, we wanted to ascertain the role of molecular sieves in the reaction. It is clear from the results in Table 1 that addition of molecular sieves is the key factor to improving the formal S_N_2′/S_N_2 (**5**/:**6**) regioselectivity. Our next control reaction (Scheme 4) shows that molecular sieves are also crucial for chirality transfer and *E*/*Z* selectivities. Removing molecular sieves from the reaction results in a completely racemic product **5 db** and a poor 3:1 *E*/*Z* ratio (vs. 89:11 e.r. and 9:1 *E*/*Z* with molecular sieves added).^21^

**Scheme 5:**
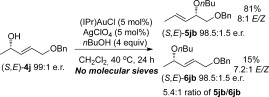
Results by Widenhoefer et al.^14^

**Scheme 6:**
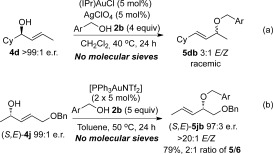
Control reactions to ascertain effects of conditions vs. substrate. (a) Standard substrate by using the conditions of Widenhoefer et al. (b) Substrate 4 j by using conditions that usually result in racemisation.

Previously, Widenhoefer et al. had shown that chirality transfer is possible on substrate **4 j**, without the need for molecular sieves (Scheme 5).^14^ Having just ascertained that molecular sieves are crucial to avoid racemisation, we therefore thought it important to investigate whether the conditions in Scheme 5 allow for the omission of molecular sieves in chirality-transfer reactions, or whether successful chirality transfer without molecular sieves is in fact specific to substrate **4 j**. Our results in Scheme 6 show that the latter is true. Employing the conditions of Widenhoefer et al. on substrate **4 d** (which undergoes chirality transfer with molecular sieves under our conditions, Table 2, but racemises in the absence of 3 Å MS, Scheme 4), results in racemic product **5 db**. However, employing substrate **4 j** under conditions that usually result in racemisation (i.e., no 3 Å MS), results in efficient chirality transfer. Therefore, it appears that the substituent on substrate **4 j** plays a significant role in allowing the chirality-transfer process to proceed efficiently even without molecular sieves. For a more general substrate scope, however, the addition of molecular sieves to the reaction is crucial for successful chirality transfer.

**Scheme 7:**
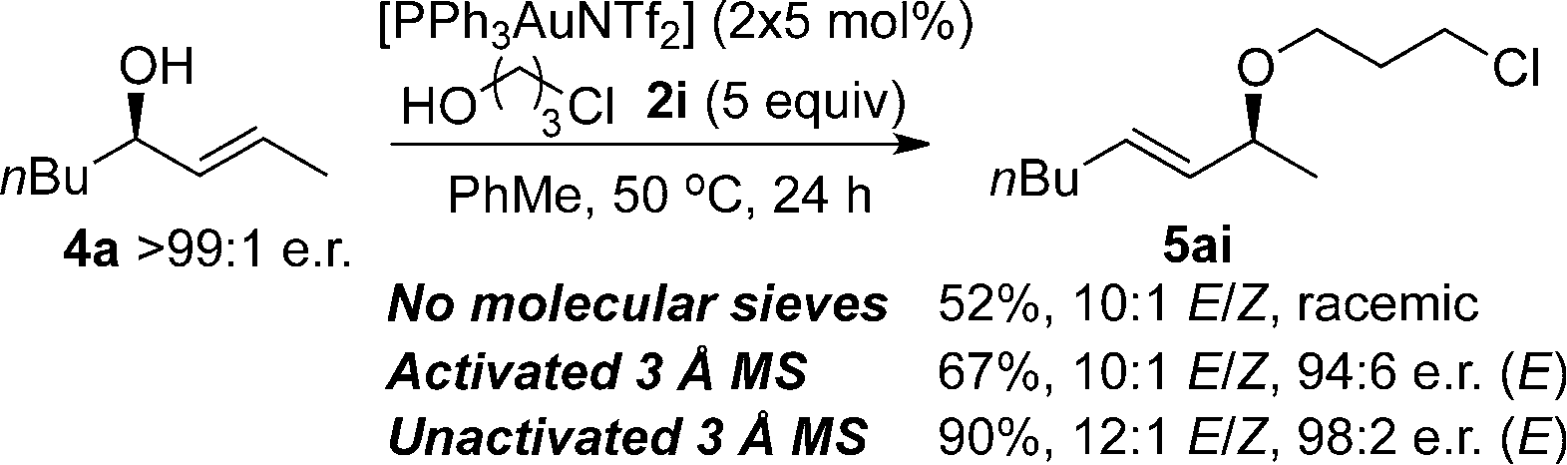
Comparing results of reactions with no molecular sieves, activated sieves and unactivated sieves.

The effect of molecular sieves in the reaction is stark as well as puzzling. There are several possibilities regarding the mode of action of molecular sieves in the reaction that may lead to the observed chirality-transfer outcome. Possible reasons for this could be: i) removal of excess water from the reaction; ii) the slightly basic nature of molecular sieves, which may deactivate the gold catalyst;^22^ and iii) the polar surface of molecular sieves may result in the reaction occurring closer to the surface, thereby changing the aggregation levels or transition state. However, a control reaction to test point (i) shows that chirality transfer is observed regardless of whether the molecular sieves are activated or not, thus ruling out this possibility (Scheme 7). In fact, the reaction occurs with even better yields and e.r. with *unactivated* vs. activated sieves (67 %, 94:6 e.r. vs. 90 %, 98:2 e.r., Scheme 7).

Density functional theory (DFT) calculations were therefore employed to explore the mechanism of these direct allylic etherification reactions. In particular, we sought to understand why our initial expectation of chirality transfer (cf. Scheme 1 and 2) was not borne out, except in the presence of molecular sieves. In the calculations we have studied the symmetrically substituted dimethyl allylic alcohol **4 k** (as the *R,E*-isomer) reacting with ethanol (**2 o**) to give **5 ko**. This choice removes the potential complication of any subsequent S_N_2′ reaction at **5 ko** as this would return the same **5 ko** product. Experimental studies indicate that the catalysis is not significantly affected by the nature of the alcohol and so ethanol was chosen for simplicity. The calculations (run with SDD pseudopotentials and basis sets on Au and P, with d-orbital polarization on the latter, and 6-31g** basis sets on other centres) report free energies derived from a BP86-D3(toluene) protocol, that is, gas-phase free energies based on BP86 optimisations, corrected for dispersion and toluene solvation (using Grimme’s D3 parameter set and the PCM approach respectively, see Supporting Information for full details).

**Scheme 8:**
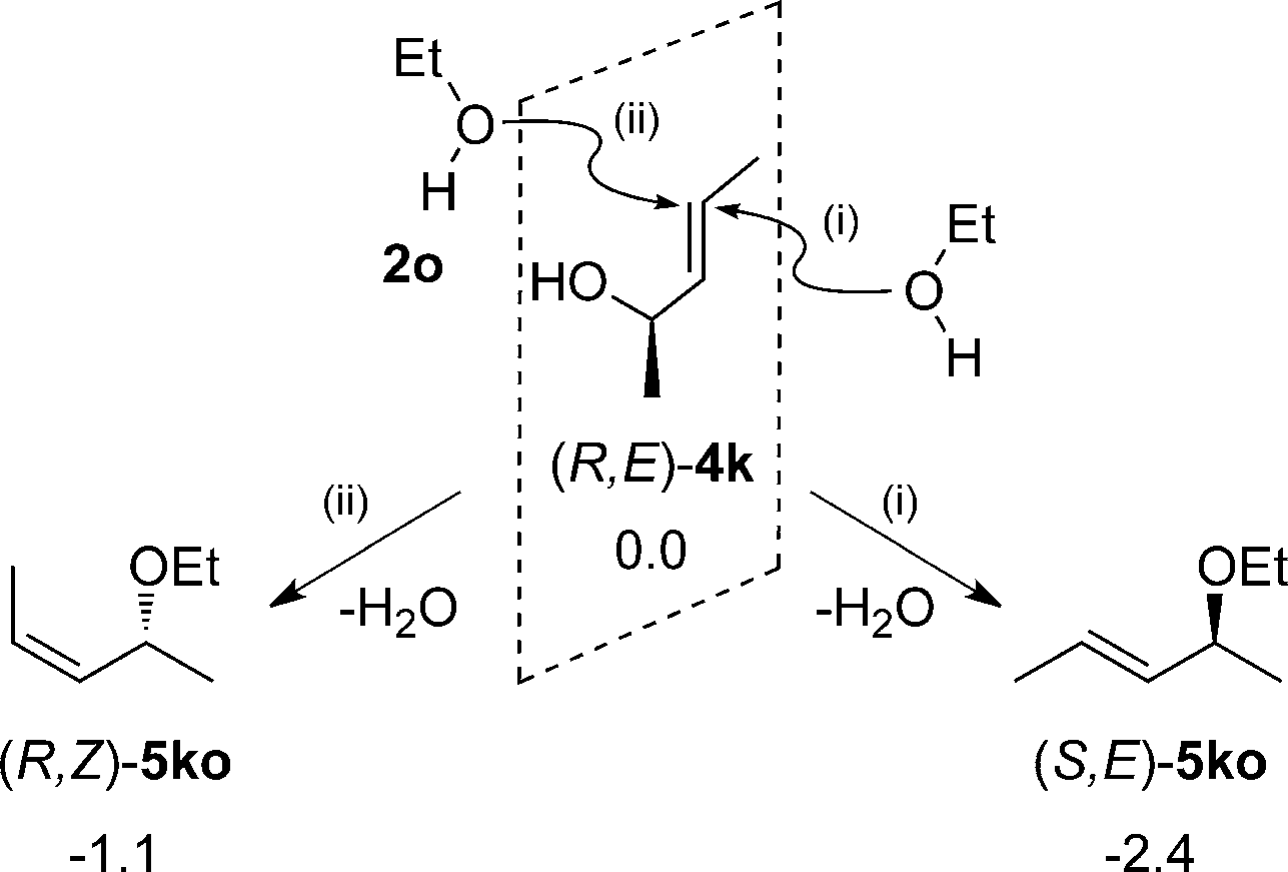
Possible outcomes of the Au-mediated reaction of (*R*,*E*)-4 k with ethanol (2 o) to give either (*S*,*E*)- or (*R*,*Z*)-5 ko. Computed product free energies are indicated in kcal mol^−1^, relative to the reactant set to 0.0 kcal mol^−1^.

The Au-catalysed direct allylic etherification reaction is thought to proceed^23^ via coordination of the {Au(PPh_3_)}^+^ fragment at the C=C π-bond of the allylic alcohol. As shown by Mukherjee and Widenhoefer,^14^ if the alcohol nucleophile attacks at the opposite face to Au then only two outcomes are possible with an enantiopure substrate: with (*R,E*)-**4 k** either (*S,E*)-**5 ko** or (*R,Z*)-**5 ko** will be formed (Scheme 8). The formation of both products (alongside water) is computed to be thermodynamically downhill, with the *E*-isomer favoured over the *Z*-form by 1.3 kcal mol^−1^. This equates to a *E*/*Z* ratio of approximately 9:1 at 298 K, fairly typical of the *E*/*Z* selectivities seen with dialkyl-substituted allylic alcohols (Tables 1 and 2). This result also suggests the reaction may be proceeding under thermodynamic control.

**Figure 1:**
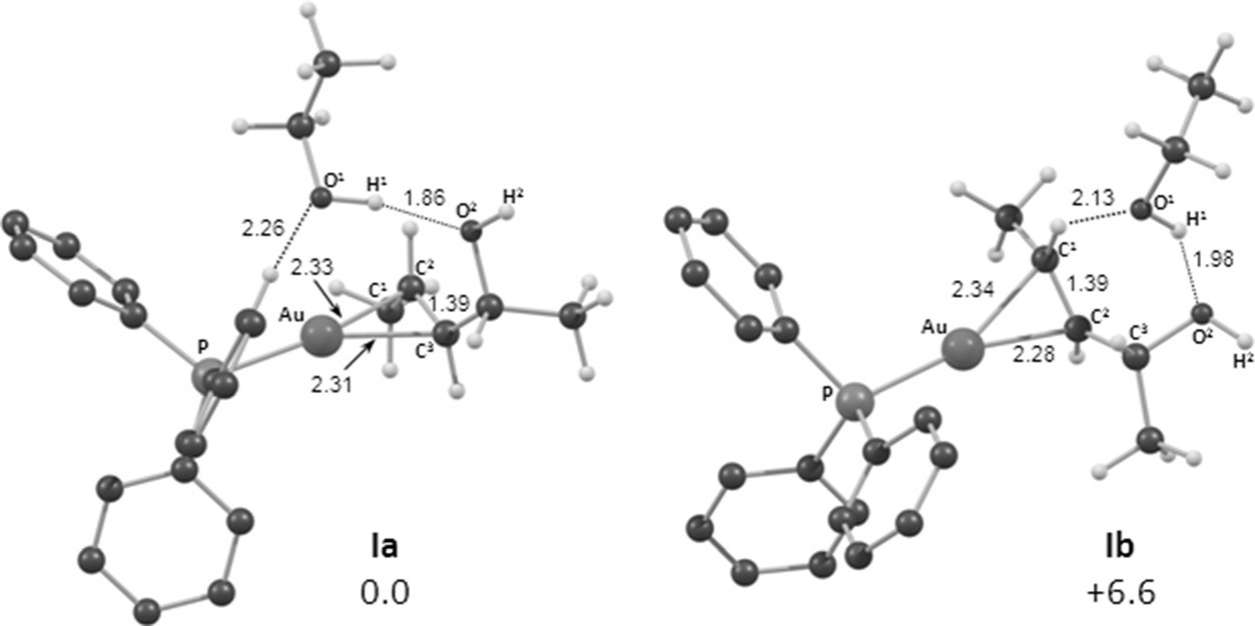
Computed structures of two forms of [(Ph_3_P)Au{(*R*,*E*)-4 k}]^+^⋅EtOH, I, with computed free energies (kcal mol^−1^, relative to I a set to zero) and selected distances in Å. Phosphine H atoms are omitted for clarity, with the exception of that interacting with the EtOH molecule in I a.

For the computed mechanism, we consider the direct etherification to start from the π-bound adduct [(Ph_3_P)Au{(*R,E*)-**4 k**}]^+^**⋅**EtOH, **I**, in which the EtOH is hydrogen-bonded to the OH group of the allylic alcohol.^24^ Several arrangements of this adduct were located in the course of this study and the most stable of these, **I a**, has the EtOH lying over the Au centre (i.e., *syn* to Au), with interactions to both the O of the allyl group (1.86 Å) and also to one C−H bond of the PPh_3_ ligand (2.26 Å, see Figure 1, which also provides the associated labelling scheme). The most stable adduct, where the EtOH is located on the other side of the C=C π-bond (i.e., *anti* to Au, **I b**), is 6.6 kcal mol^−1^ higher in energy, with the EtOH showing close contacts with one allylic proton, as well as the OH group. All energies in this section will be quoted relative to **I a** set to 0.0 kcal mol^−1^.

**Figure 2:**
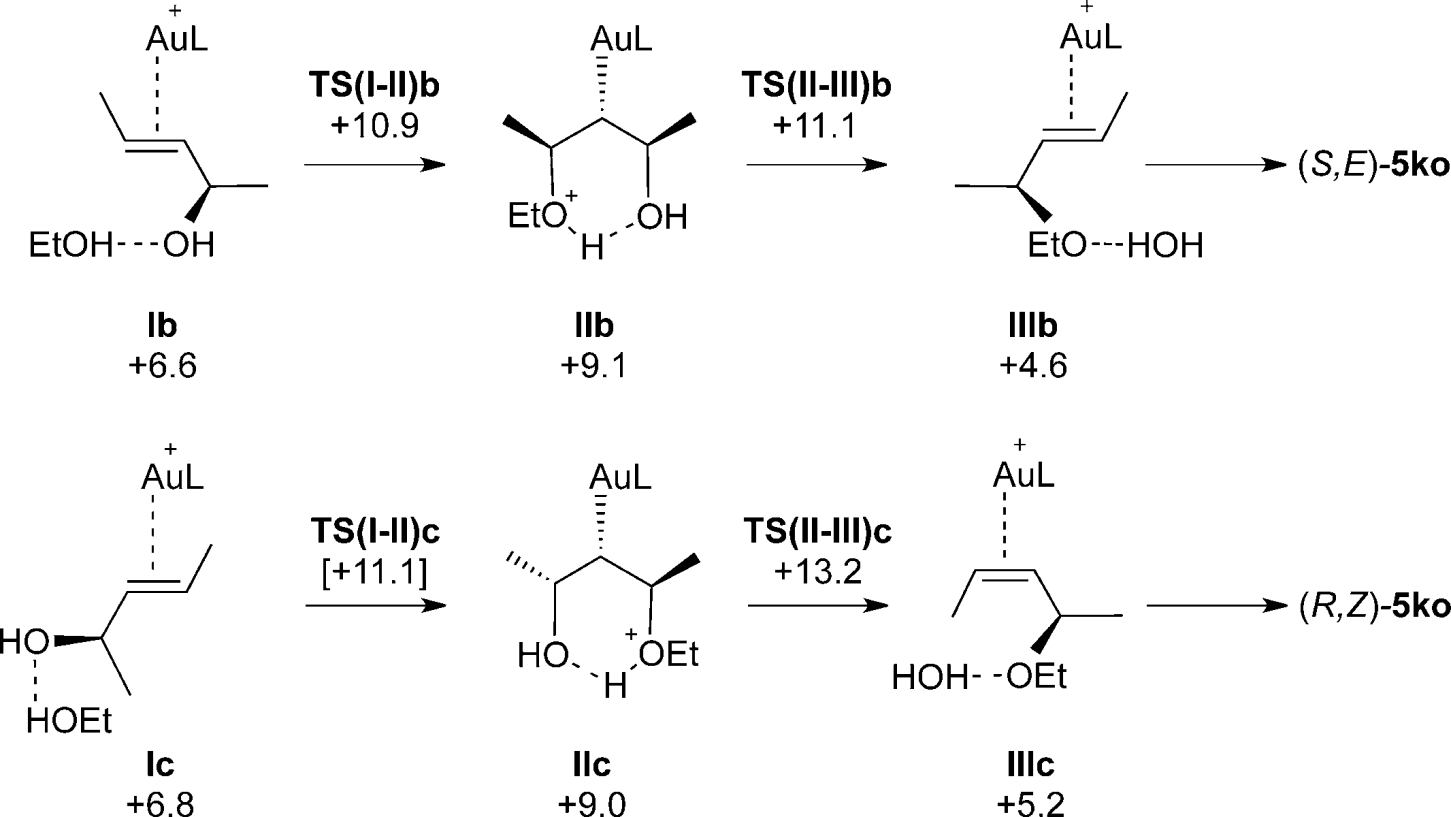
Key intermediates and energetics for the Au-catalysed reaction of (*R*,*E*)-4 k with ethanol (2 o) through *anti* attack to give either (*S*,*E*)-5 ko or (*R*,*Z*)-5 ko (L=PPh_3_). Computed free energies are indicated in kcal mol^−1^ and quoted relative to I a set to 0.0 kcal mol^−1^.

**Figure 3:**
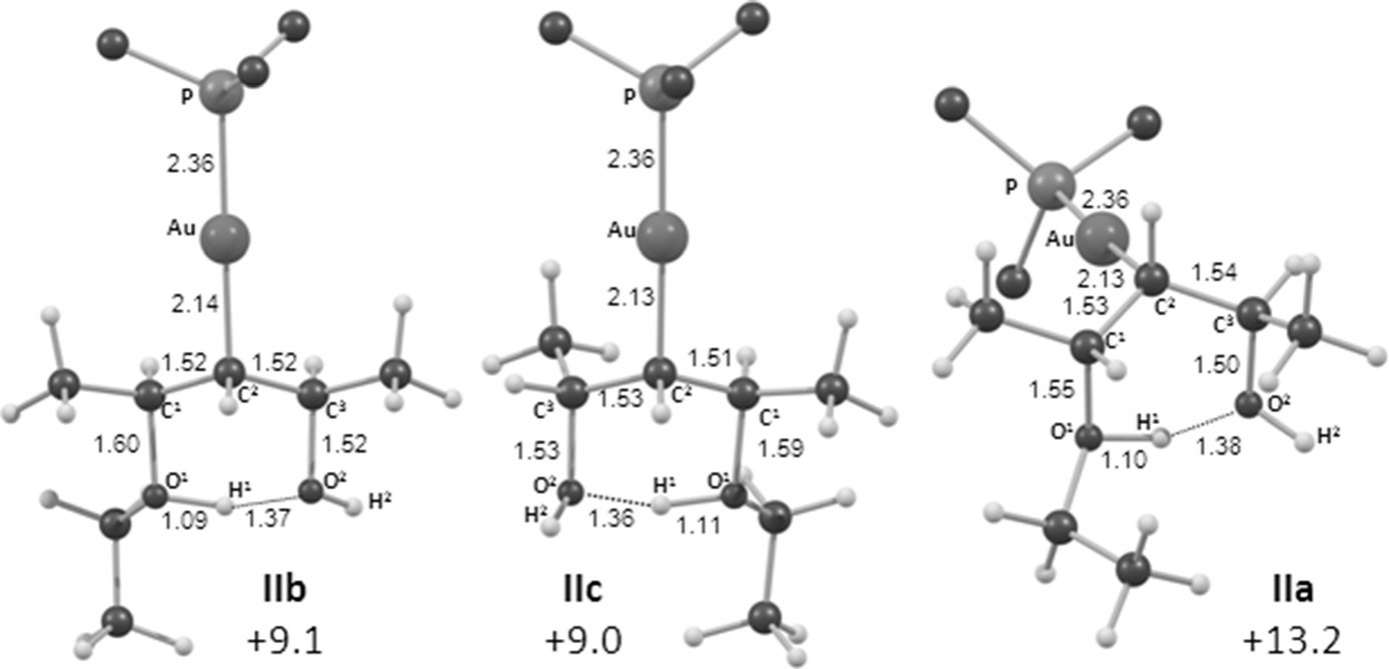
Different forms of intermediate II: II b and II c are alternative species formed by *anti* attack of ethanol, whereas II a is formed by *syn* attack. Computed free energies (kcal mol^−1^) are quoted relative to I a set to zero and selected distances are in Å. Phosphine phenyl substitutents are truncated at the *ipso* carbon for clarity.

The key steps and associated energetics for direct etherification through *anti*-attack of EtOH are outlined in Figure 2. Starting from **I b**, C−O bond formation proceeds through a transition state at +10.9 kcal mol^−1^ to give intermediate **II b** at +9.1 kcal mol^−1^. The computed structure of this species is shown in Figure 3 and displays the anticipated hydrogen-bonded chair-like structure with the Au and both Me substituents all occupying equatorial positions. Similar structures have been reported at a {Au(NHC)}^+^ fragment.17a From here H^+^ transfer induces loss of water and concomitant formation of the Au−C^3^ bond to give intermediate **III b** in which the allylic ether product is bound through the C^2^=C^3^ bond and water is hydrogen-bonded to the ether oxygen. Dissociation will give **5 ko** as the *S,E*-form. Of the two transition states, the higher is **TS(II–III)b** at +11.1 kcal mol^−1^. An analogous series of events accounts for the formation of (*R,Z*)-**5 ko**. Starting from **I c** (*G*=+6.8 kcal mol^−1^), the chair-like intermediate **II c** is formed via **TS(I–II)c** at +11.1 kcal mol^−1^. **II c** is similar to **II b** but now has one methyl substituent in an axial position. Loss of H_2_O via **TS(II–III)c** at +13.2 kcal mol^−1^ leads to **III c** from which the allylic ether product is lost as the (*R,Z*)-form. Overall, these two allylic etherification processes proceed with modest barriers (<14 kcal mol^−1^). In addition, as these reactions are only marginally downhill thermodynamically (Scheme 8), they are likely to be reversible under the reaction conditions. Hence, a thermodynamic distribution of products is seen that favours the (*S,E*)*-***5 ko** product.

**Figure 4:**
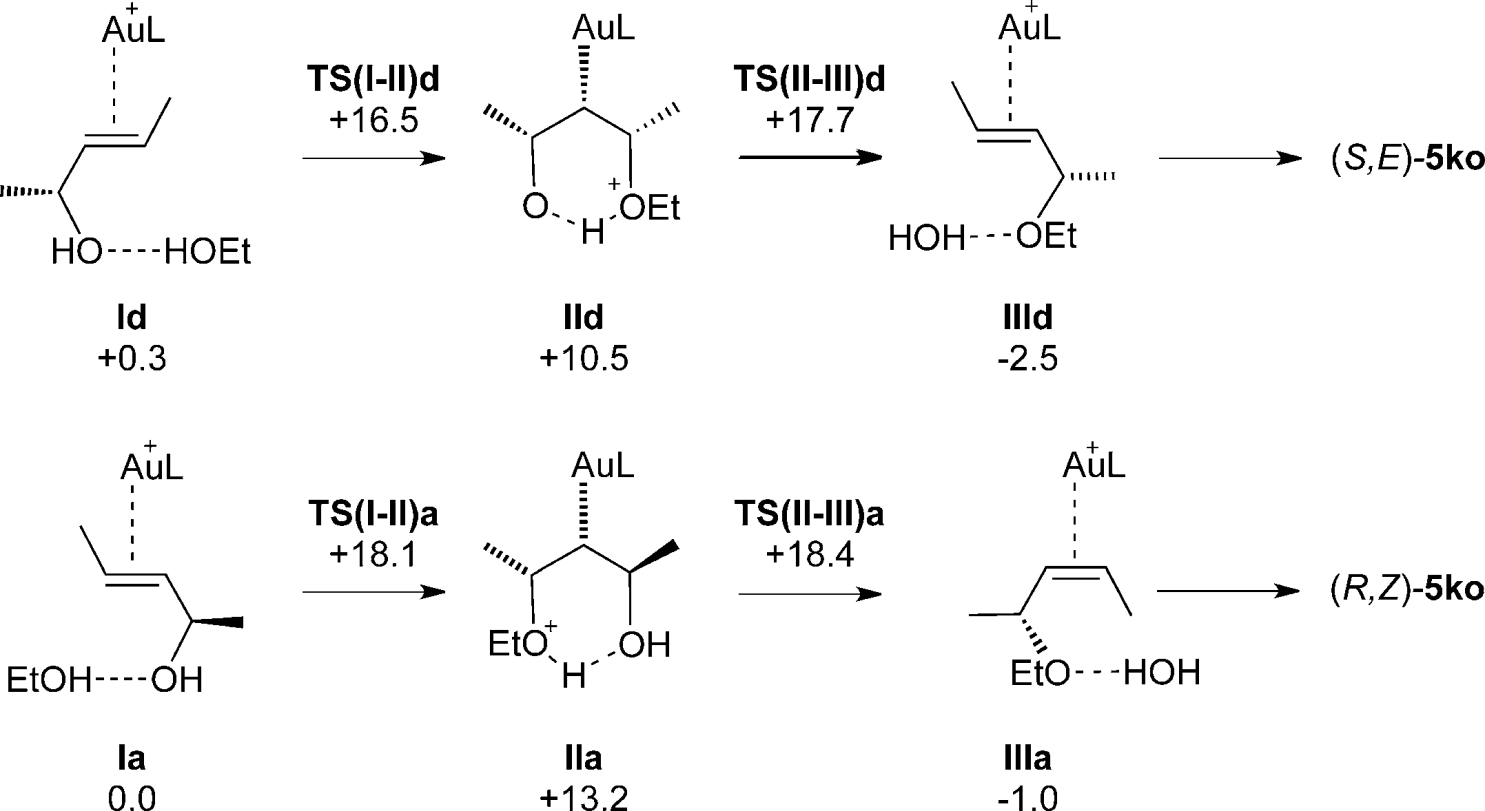
Key intermediates and energetics for the Au-catalysed reaction of (*R*,*E*)-4 k with ethanol (2o) by *syn* attack to give either (*S*,*E*)-5 ko or (*R*,*Z*)-5 ko (L=PPh_3_). Computed free energies are indicated in kcal mol^−1^ and quoted relative to I a set to 0.0 kcal mol^−1^.

The observation of the enhanced stability of precursor **I a** in which EtOH is positioned *syn* to Au suggests the possibility of alternative *syn* attack mechanisms and two such processes have been characterised (see Figure 4). Hydrogen-bonded chair-like intermediates **II a** and **II d** are located, but now with the Au in an axial position (see Figure 3 for the structure of **II a**). Loss of water from **II a** and **II d** then leads to the formation of (*R,Z*)-**5 ko** and (*S,E*)-**5 ko**, respectively, that is, the same products as seen in the *anti* attack processes. *syn* attack, however, entails barriers of 17.7 and 18.4 kcal mol^−1^, and so these processes will not be competitive with the *anti* attack mechanisms described above.

**Scheme 9:**
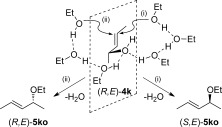
Possible mechanisms accounting for loss of chirality transfer in the Au-mediated reactions of (*R*,*E*)-4 k with three ethanol molecules to form both (*S*,*E*)-5 ko (pathway (i)) and (*R*,*E*)-5 ko (pathway (ii)).

The mechanisms outlined so far are consistent with the transfer of chirality shown in Widenhoefer’s example (Scheme 5) and anticipated prior to our work. However, our experimental studies have shown that in most cases such chirality transfer only occurs in the presence of molecular sieves and that in fact under sieve-free conditions loss of chirality dominates. To account for this a mechanism, involving nucleophilic attack at one face of the allylic alcohol and loss of water from the opposite face is required. One way to achieve this is to invoke a proton chain transfer mechanism^25^ involving several EtOH molecules. This is illustrated in Scheme 9 for the case of three EtOH molecules. Pathway (i) is equivalent to the *anti* attack in Scheme 8, where a three EtOH molecule chain now promotes loss of water *anti* to Au with formation of (*S,E*)-**5 ko**. In contrast, pathway (ii) is able to accommodate a *syn* attack by EtOH while still delivering a proton onto the allylic hydroxyl group that is in an *anti* position. This leads to the formation of (*R,E*)-**5 ko**. If the barriers to pathways (i) and (ii) are comparable, the result will be the loss of chirality transfer that is seen experimentally.

**Figure 5:**
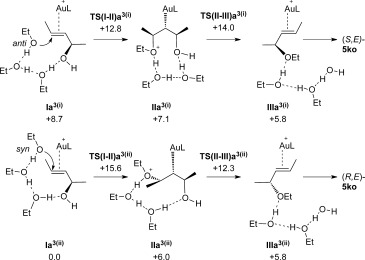
Key intermediates and energetics for the Au-catalysed reaction of (*R*,*E*)-4 k in the presence of three ethanol molecules to give (i) (*S*,*E*)-5 ko and (ii) (*R*,*E*)-5 ko (L=PPh_3_). Computed free energies are indicated in kcal mol^−1^ and quoted relative to I a^3(i)^ set to 0.0 kcal mol^−1^.

A model incorporating three ethanol molecules was set up to test these ideas (see Figure 5), three being the minimum number of EtOH molecules required to access pathway (ii), which requires both faces of the allylic alcohol substrate to be engaged. As in the single EtOH system, the most stable form of the hydrogen-bonded precursor has one EtOH positioned over the Au centre. This species, **I a^3(ii)^**, leads ultimately to the (*R,E*)-**5 ko** product along pathway (ii), as described below. The alternative arrangement relevant for pathway (i) is seen in **I a^3(i)^** and lies 8.7 kcal mol^−1^ higher in energy. Both the initial attack of EtOH at this species (via **TS(I–II)a^3(i)^** at +12.8 kcal mol^−1^) and the subsequent loss of water (via **TS(II–III)a^3(i)^** at +14.0 kcal mol^−1^) occur *anti* to the Au and hence yield the (*S,E*)-**5 ko** product. The overall barrier for pathway (i) is 14.0 kcal mol^−1^.

**Figure 6:**
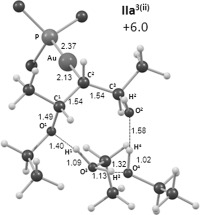
Computed structure of intermediate II a^3(ii)^ located along pathway (ii) on route to the formation of (*R*,*E*)-5 ko. The computed free energy is in kcal mol^−1^ and is relative to I a^3(i)^ set to zero. Selected distances are in Å and phosphine phenyl substituents are truncated at the *ipso* carbon for clarity.

In pathway (ii), the EtOH molecule lying over the Au centre in **I a^3(ii)^** is linked through two hydrogen-bonded EtOH molecules to the allylic hydroxyl group, which maintains a position *anti* to the Au. *syn* attack of EtOH proceeds to give **II a^3(ii)^** (*G*=+6.0 kcal mol^−1^) via **TS(I–II)a^3(ii)^** at +15.6 kcal mol^−1^. Figure 6 shows the computed structure of **II a^3(ii)^** and highlights the *anti* arrangement of the EtOH nucleophile and the putative H_2_O leaving group. In this case, the water dissociation is the easier step and so (*R,E*)-**5 ko** is formed with an overall barrier of 15.6 kcal mol^−1^. Similar barrier heights are therefore computed for the formation of both (*S,E*)-**5 ko** (Δ*G*^≠^_calc_=14.0 kcal mol^−1^) and (*R,E*)-**5 ko** (Δ*G*^≠^_calc_=15.6 kcal mol^−1^) and this, coupled with reversibility of these transformations, means that both enantiomers will be formed over the timescale of the reaction, leading to the unexpected loss of chirality transfer.^26^

**Scheme 10:**
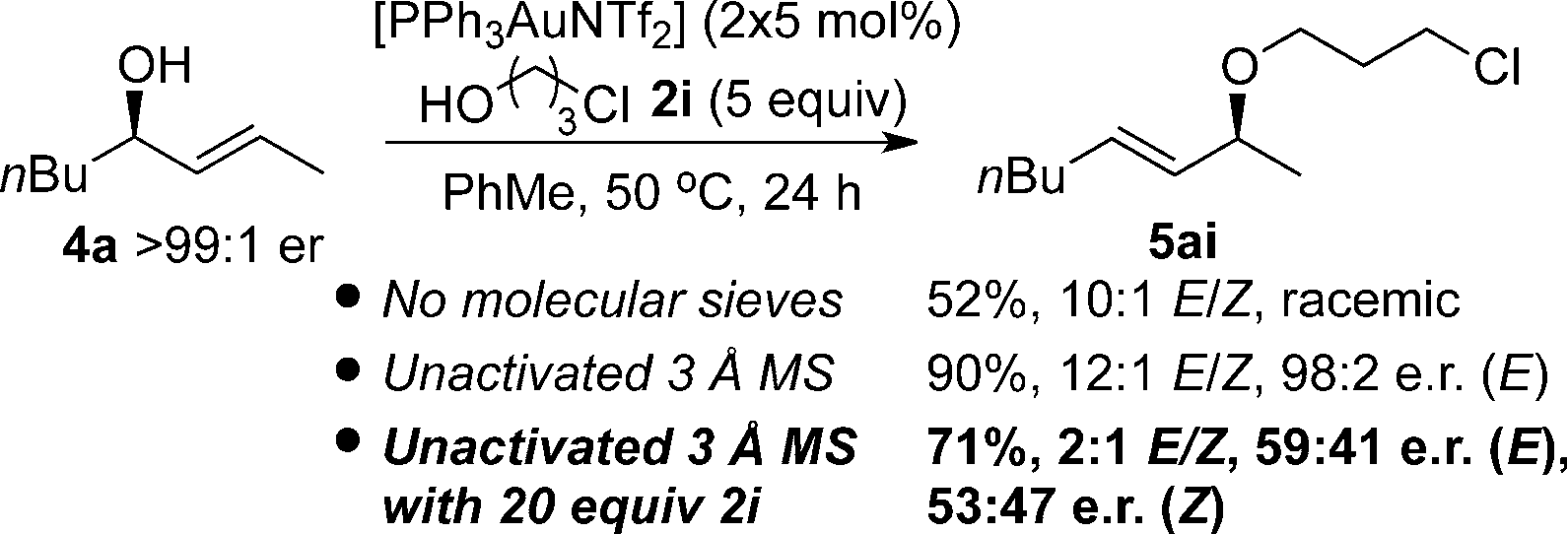
Comparing results of reactions with no molecular sieves, unactivated sieves and large excess of alcohol nucleophile.

**Scheme 11:**
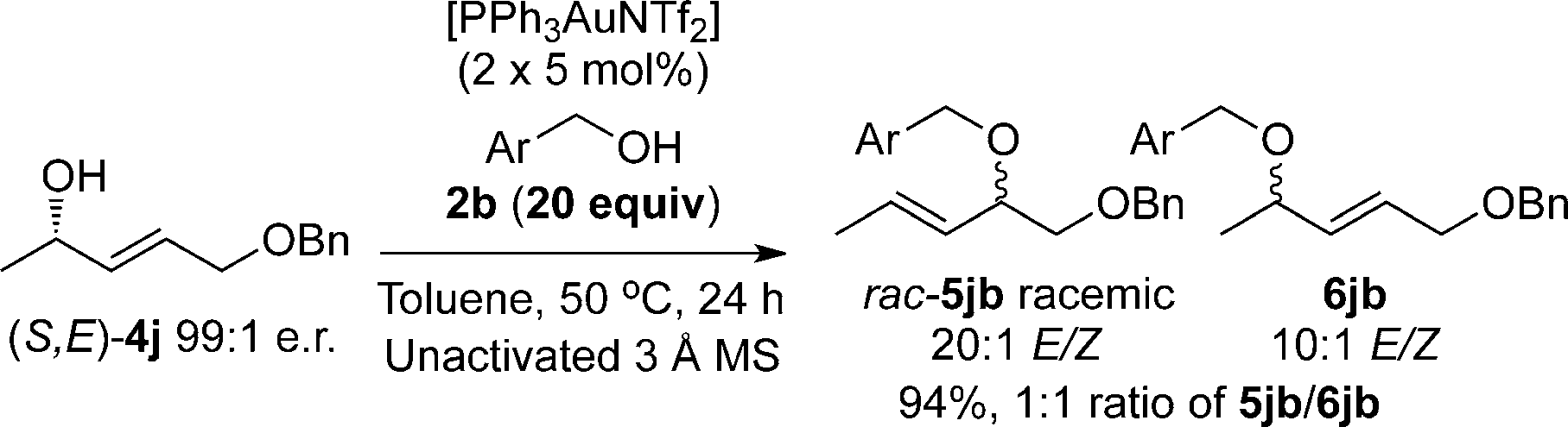
Large excess of alcohol nucleophile results in racemisation with 4 j, overriding the effect of the ether moiety (CH_2_OBn) and molecular sieves.

A plausible explanation for the requirement of molecular sieves for efficient chirality transfer is their role in disrupting the type of proton chain transfer mechanism (i.e., aggregation levels of alcohol) shown in Scheme 9, which are a potential cause of racemisation. To test our hypothesis, the reaction was carried out with a large excess of alcohol nucleophile^27^ (Scheme 10), which should outcompete the role of the molecular sieves. Indeed, very poor enantiomeric ratios are observed in the presence of 20 equivalents of alcohol **2 i** (Scheme 10), thereby lending support to our theory. Following this train of thought, we postulated that the reason substrate **4 j** does not require molecular sieves for chirality transfer (Schemes 5 and 6) is because the ether moiety (-CH_2_OBn) within the substrate may be playing a similar role to molecular sieves: disrupting the proton chain transfer mechanism, presumably by hydrogen-bonding. Indeed, when the reaction with **4 j** is repeated with a large excess (20 equiv) of alcohol nucleophile, this overrides any effect of the ether moiety as well as molecular sieves and produces racemic product **5 jb** in a poor 1:1 **5 jb**/**6 jb** regioselectivity (Scheme 11).

**Scheme 12:**
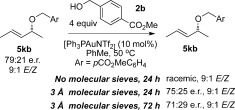
Resubjecting 5 kb to reaction conditions: racemisation without molecular sieves and much slower erosion of e.r. with molecular sieves.

An additional explanation for erosion of chirality transfer is possible for allylic ether products such as **5 kb**. In this case, a second formal S_N_2′ reaction on **5 kb** will lead to the same product, but with chirality transfer to the opposite enantiomer. Calculations indicate that the barrier for the second formal S_N_2′ is readily accessible (via a transition state at 9.8 kcal mol^−1^ for **5 ko**). As, by definition, the energies of these two enantiomers of **5 kb** are the same, these reversible processes will produce a racemic mixture. This is exemplified by control experiments shown in Scheme 12, where the product **5 kb** from Table 2, entry 10 is resubjected to the reaction conditions without sieves to produce a racemic mixture. In contrast, in the presence of sieves, this process is considerably slowed (Scheme 12). Although the reasons for the remarkable impact of the molecular sieves on these transformations are currently unclear, nevertheless, their effect in providing kinetic control for these experiments is remarkable and, moreover, synthetically useful.

## Conclusion

We have successfully developed conditions for highly regioselective, gold(I)-catalysed direct allylic etherification of alcohols with chirality transfer to access enantioenriched γ-substituted secondary allylic ethers. A thorough substrate screen shows that very high levels of chirality transfer can be achieved (up to >99:1 e.r.). The reaction is very functional-group tolerant and proceeds in the presence of unprotected groups such as alkyl halides, tertiary alcohols, alkenes and acid-sensitive acetals. Both primary and the more hindered secondary alcohol nucleophiles are tolerated well. Furthermore, we demonstrate that the addition of molecular sieves is crucial not only for excellent formal S_N_2′ selectivity, but also to ensure efficient chirality transfer. The molecular sieves need not be activated to achieve this effect, which implies that it is *not* aiding the selectivity by removal of water. DFT calculations suggest that chirality transfer should proceed under conditions that promote the reaction of a single alcohol as nucleophile. However, at higher alcohol concentrations proton chain transfer mechanisms become accessible, which permit alternative pathways that will erode the chirality transfer. A plausible role of the molecular sieves is to disrupt the aggregation of alcohol molecules in order to prevent loss of chirality through this pathway. Whatever the underlying reasons, the impact of molecular sieves in controlling the outcome of these allylic etherification reactions is remarkable and synthetically useful.

## Experimental Section

### General procedure

A solution of [PPh_3_AuNTf_2_] (2:1 toluene adduct, 5 mol %), allylic alcohol **4** (0.101 mmol), alcohol **2** (0.506 mmol) and 3 Å molecular sieves (8 mg) in toluene (260 μL) was stirred at 50 °C under air for 8 h. Then, [PPh_3_AuNTf_2_] (2:1 toluene adduct, 5 mol %) was added and the resulting solution was stirred at 50 °C for a further 16 h. The resulting solution was filtered through a short plug of silica, washing with 9:1 hexane/Et_2_O. The filtrate was evaporated under reduced pressure to give the crude product, which was purified by flash column chromatography. Full experimental procedures, characterisation for all new compounds and copies of ^1^H and ^13^C NMR spectra are provided in the Supporting Information.

## Supporting Information

As a service to our authors and readers, this journal provides supporting information supplied by the authors. Such materials are peer reviewed and may be re-organized for online delivery, but are not copy-edited or typeset. Technical support issues arising from supporting information (other than missing files) should be addressed to the authors.

miscellaneous_information
