# Supplementary material for: Chirality Transfer in Gold(I)-Catalysed Direct Allylic Etherifications of Unactivated Alcohols: Experimental and Computational Study
Source: Chemistry. 2015 Aug 6;21(39):13748–57. doi: 10.1002/chem.201501607 (PMC4586480; doi:10.1002/chem.201501607)
Supplement: Supplementary file 1 — miscellaneous_information [file chem0021-13748-sd1.pdf]

CHEMISTRY
A European Journal

Supporting Information

Chirality Transfer in Gold(I)-Catalysed Direct Allylic Etherifications of Unactivated Alcohols: Experimental and Computational Study

Graeme Barker, David G. Johnson, Paul C. Young, Stuart A. Macgregor,* and Ai-Lan Lee*[a]

chem_201501607_sm_miscellaneous_information.pdf

Supporting Information

Table of contents

Experimental Supporting Information: Pages 3-189

1. General Experimental Section pg 3

2. Experimental Procedures pg 4

3. ^{1}H NMR and ^{13}C NMR Spectra and HPLC/GC Traces of Synthesised Compounds pg 42

4. References for Experimental Section pg 189

Computational Supporting Information: Pages 190 -303

Computational Details: 191

Organic Products 192

Ligand Exchange Energies 199

Pathways for Direct Etherification: 207

(i) Single EtOH nucleophile, Pathway (i), anti attack 207

(i) Single EtOH nucleophile, Pathway (ii), *syn* attack 233

(iii) Three EtOH nucleophiles 259

1) General Experimental Section

^1H NMR spectra was recorded on Bruker AV 300 and AV 400 spectrometers at 300 and 400 MHz respectively and referenced to residual solvent. ^{13}C NMR spectra were recorded using the same spectrometers at 75 and 100 MHz respectively. Chemical shift data are quoted in parts per million (ppm) and are referenced to tetramethylsilane (TMS) or to residual solvent peaks ($CDCl_3$ at δ_H 7.26). *J* values are given in Hz and s, d, dd, dt, t, q and m abbreviations correspond to singlet, doublet, doublet of doublet, doublet of triplet, triplet, quartet and multiplet. Mass spectra were obtained at the EPSRC National Mass Spectrometry Service Centre in Swansea and APCI represents atmospheric pressure chemical ionisation. Infrared spectra were obtained on Perkin-Elmer Spectrum 100 FT-IR Universal ATR Sampling Accessory, deposited neat to a diamond/ZnSe plate. Flash column chromatography was carried out using Matrix silica gel 60 from Fisher Chemicals and TLC was performed using Merck silica gel 60 F254 pre-coated sheets and visualised by UV (254 nm) or stained by the use of aqueous acidic $KMnO_4$ or aqueous acidic ammonium molybdate as appropriate. Petrol ether refers to petroleum ether (40-60 °C). Chemicals were purchased from Sigma-Aldrich, Acros, Fisher and Apollo chemical companies and used without further purification. Tetrahydrofuran was dried by distillation from sodium – benzophenone under nitrogen or using an MBRAUN SPS-800 solvent purification system. High performance liquid chromatography (HPLC) was carried out on Agilent Technologies 1120 Compact LC. Gas chromatography was carried out on a Shimadzu GC2014 with FID.

The gold(I)-catalysed reactions were carried out in screw cap 1 dram vials unless otherwise indicated. No special precautions to exclude air or moisture were taken unless otherwise indicated.

2) Experimental Procedures

2.1) General Experimental Procedures

General Procedure A – Sharpless Resolution of Allylic Alcohols[1]

$Ti(O^iPr)_4$ (1.2 eq.) was added dropwise to a stirred solution of racemic allylic alcohol (1.0 eq.) and L-(+)-DIPT (1.2 eq.) in CH_2Cl_2 (0.1 M solution of allylic alcohol) at –20 °C under Ar. The resulting solution was stirred at –20 °C for 30 min. Then, a 6 M solution of TBHP (0.6 eq.) in dioxane was added dropwise. The resulting solution was stirred at –20 °C for 16 h. Then, a solution of $FeSO_4.7H_2O$ (10 eq.) and citric acid (5.5 eq) in H_2O (1 M solution of $FeSO_4.7H_2O$) was added in one portion. The resulting solution was allowed to warm to rt and vigorously stirred until two layers were evident. The layers were separated, extracting the aqueous with CH_2Cl_2. The combined organic layers were dried ($MgSO_4$) and evaporated under reduced pressure to give the crude product.

General Procedure B – Enzymatic Resolution of Allylic Alcohols[1]

Amano AK Lipase (from *P. Fluorescens*, 75 mg/1.00 mmol of allylic alcohol) was added in one portion to a stirred solution of vinyl acetate (2.7 eq.), allylic alcohol (1.0 eq.) and 4 Å molecular sieves (75 mg/1.00 mmol of allylic alcohol) in hexane (0.07 M solution of allylic alcohol). The resulting suspension was stirred at rt under air, monitoring the reaction by either chiral GC (until >99:1 er was obtained) or by ^1H NMR (until 50% conversion to allylic ester was obtained). Then, the resulting suspension was filtered over Celite®, washing with Et_2O. The filtrate was evaporated under reduced pressure to give the crude product.

General Procedure C – Optimised Conditions for Chirality transfer

In all cases, identical procedures were carried out on *racemic* allylic alcohols to obtain racemic samples of allylic ethers for determination of separation conditions by CSP-HPLC or CSP-GC.

A solution of PPh_3AuNTf_2 (2:1) toluene adduct (to give 5 mol% Au), allylic alcohol (0.101 mmol), alcohol (0.506 mmol) and 3 Å molecular sieves (8 mg) in PhMe (260 µL) was stirred at 50 °C under air for 8 h. Then, PPh_3AuNTf_2 (2:1) toluene adduct (to give 5 mol% Au) was added and the resulting solution was stirred at 50 °C for 16 h. The resulting solution was filtered over a short plug of silica, washing with 9:1 hexane:Et_2O. The filtrate was evaporated under reduced pressure to give the crude product.

2.2) Synthesis of Allylic Alcohols

(*E*)-Oct-2-en-4-ol, 4a

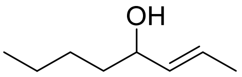

4a

^{n}BuLi (19.97 mL of a 1.6 M solution, 31.95 mmol) was added dropwise to a stirred solution of crotonaldehyde (2.0 g, 2.36 mL, 28.53 mmol) in THF (40 mL) at –78 °C under Ar. The resulting solution was stirred at –78 °C for 2 h. The resulting solution was then allowed to warm to rt, and 1 M $HCl_{(aq)}$ (30 mL) was added. The layers were separated, extracting the aqueous with Et_2O (2 × 40 mL). The combined organic layers were washed with saturated $NaHCO_3$ (60 mL) and saturated brine (60 mL), dried ($MgSO_4$) and evaporated under reduced pressure to give the crude product. Purification by flash column chromatography on silica with 9:1→5:1 hexane:Et_2O gave the title product **4a** (2.82 g, 77%) as a colourless oil, R_F 0.3 (5:1 hexane:Et_2O); IR (film) 3371 (OH), 2957, 2931, 2860, 1453, 1378, 1256, 1180, 1138, 1024, 1004, 964, 938, 898, 795, 730, 609 cm^{-1}; ^{1}H NMR (300 MHz, $CDCl_3$) δ H 5.64 (1H, dqd, *J* = 15.2, 6.3, 0.7 Hz, MeCH=), 5.46 (1H, ddq, *J* = 15.3, 7.1, 1.5 Hz, OCHC*H*=), 4.07-3.91 (1H, , OCH), 1.68 (3H, dd, *J* = 6.3, 1.5 Hz, =CH*Me*), 1.57-1.39 (3H, m, CH_2), 1.34-1.26 (3H, m, CH_2), 0.89 (3H, br t , *J* = 6.9 Hz, CH_2*Me*); ^{13}C NMR (75 MHz, $CDCl_3$) δ C 134.4 (=CH), 126.8 (=CH), 73.3 (OCH), 37.1 (CH_2), 27.8 (CH_2), 22.7 (CH_2), 17.8 (Me), 14.1 (Me); HRMS $C_8H_{15}O$ ([M–H]$^+$) requires 127.1117, found 127.1114; CSP-GC (Chiraldex-GTA, 65 °C, 34.8 cm s^{-1}) (*S*)-**4a** 25.79 min, (*R*)-**4a** 26.78 min.

(*R*,*E*)-Oct-2-en-4-ol, (*R*)-4a

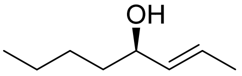

(*R*)-**4a**

Following general procedure **A**, *rac*-**4a** (2.0 g, 15.6 mmol), $Ti(O^{i}Pr)_4$ (5.32 g, 5.54 mL, 18.72 mmol), L-(+)-DIPT (4.61 g, 3.83 mL, 18.72 mmol) and TBPH (1.56 mL of a 6 M solution in dioxane, 9.36 mmol) in CH_2Cl_2 (156 mL) then $FeSO_4.7H_2O$ (47.0 g) and citric acid (17.2 g) in H_2O (156 mL) gave the crude product. Purification by flash column chromatography on silica with 9:1→5:1 hexane:Et_2O as eluent gave the title product (*R*)-**4a** (415 mg, 21%, >99:1 er by CSP-GC) as a colourless oil, $[\alpha]_D$ = +8.5 (*c* = 0.94 in $CHCl_3$).

Oct-2-yn-4-ol

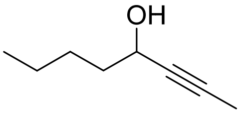

1-Propynylmagnesium bromide (30.18 mL of a 0.5 M solution in THF, 15.09 mmol) was added dropwise to a stirred solution of valeraldehyde (1.0 g, 1.23 mL, 11.61 mmol) in THF (10 mL) at 0 °C under Ar. The resulting solution was stirred, allowing to warm from 0 °C to rt over 16 h. Then, a saturated solution of $NH_4.Cl_{(aq)}$ (10 mL) was added. The layers were separated, extracting the aqueous with Et_2O (3 × 15 mL). The combined organic layers were dried ($MgSO_4$) and evaporated under reduced pressure to give the crude product. Purification by flash column chromatography on silica with 10:1→5:1 pentane:Et_2O as eluent gave oct-2-yn-4-ol (1.22 g, 84%) as a colourless oil, R_F 0.1 (10:1 pentane:Et_2O); IR (film) 3347 (OH), 2957, 2936, 2861, 1380, 1338, 1150, 1103, 1004, 986, 737, 587 cm^{-1}; ^{1}H NMR (300 MHz, $CDCl_3$) δ H 4.31 (1H, br s, OCH), 1.83 (3H, d, J = 2.1 Hz, ≡CMe), 1.71-1.59 (2H, m, OCHCH_2), 1.49-1.25 (4H, m, MeCH_2 + MeCH_2CH_2), 0.90 (3H, t, J = 7.0 Hz, CH_2*Me*); ^{13}C NMR (75 MHz, $CDCl_3$) δ C 80.9 (≡C), 80.7 (≡C), 62.8 (OCH), 38.0 (CH_2), 27.5 (CH_2), 22.5 (CH_2), 14.1 (≡C*Me*), 3.6 (CH_2*Me*); HRMS $C_8H_{13}O$ ($[M–H]^+$) requires 125.0961, found 125.0959.

(*Z*)-Oct-2-en-4-ol, 4b

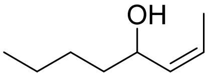

4b

Lindlar catalyst (50 mg) was added in one portion to a stirred solution of oct-2-yn-4-ol (1.0 g, 7.92 mmol) and quinoline (16 drops) in MeOH (150 mL). The resulting solution was stirred at rt under H_2 for 45 min. The resulting solution was filtered over Celite®, washing with MeOH. The filtrate was evaporated under reduced pressure to give the crude product. Purification by flash column chromatography on silica with 10:1 pentane:Et_2O as eluent gave (*Z*)-oct-2-en-4-ol **4b** (710 mg, 70%) as a colourless oil, R_F 0.2 (10:1 pentane:Et_2O); IR (film) 3334 (OH), 2956, 2929, 2860, 1456, 1076, 1017, 966, 727, 609 cm^{-1}; ^{1}H NMR (300 MHz, $CDCl_3$) δ H 5.55 (1H, dqd, J = 10.9, 6.9, 0.9 Hz, =C*H*Me), 5.37 (ddq, J = 10.9, 8.8, 1.7 Hz, OCHC*H*=), 4.43 (1H, m, OCH), 1.68 (1H, br s, OH), 1.65 (3H, dd, J = 6.9, 1.7 Hz, =CH*Me*), 1.62-1.52 (1H, m, =CHCH_AH_B), 1.48-1.17 (5H, m, =CHC$H_A$$H_B$ + MeCH_2CH_2 + MeCH_2), 0.88 (3H, t, J = 6.9 Hz, CH_2*Me*); ^{13}C NMR (75 MHz, $CDCl_3$) δ 133.8 (=CH), 126.1 (=CH), 67.4 (OCH), 37.2 (=CHCH_2), 27.6 (CH_2), 22.8 (CH_2), 14.1 (Me), 13.4 (Me); HRMS (APCI) $C_8H_{18}N$ ($[M–H_2O+NH_4]^+$) requires 128.1434, found 128.1430; CSP-GC (β-Dex, 90 °C, 35 cm s^{-1}) (*R*)-**4b** 16.82 min, (*S*)-**4b** 17.52 min.

(*R*,*Z*)-Oct-2-en-4-ol (*R*)-4b

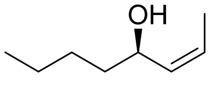

(*R*)-**4b**

Following general procedure **A**, *rac*-**4b** (710 mg, 5.54 mmol), $Ti(O^iPr)_4$ (1.87 g, 1.95 mL, 6.66 mmol), L-(+)-DIPT (1.64 g, 1.36 mL, 6.66 mmol) and TBHP (5.53 μL of a 6 M solution in dioxane, 3.32 mmol) in CH_2Cl_2 (50 mL) then $FeSO_4.7H_2O$ (15.54 g) and citric acid (5.62

g) in H_2O (50 mL) gave the crude product. Purification by flash column chromatography on silica with 9:1 pentane:Et_2O as eluent gave (*R*)-**4b** (240 mg, 34%, 81:19 er by CSP-GC) as a colourless oil, $[\alpha]_D = +14.8$ (c = 1.49 in $CHCl_3$).

Oct-3-yn-2-ol

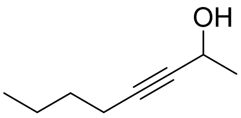

^nBuLi (14.8 mL of a 1.6 M solution in hexanes, 23.74 mmol) was added dropwise to a stirred solution of 1-hexyne (1.5 g, 2.1 mL, 18.26 mmol) in THF (20 mL) at –78 °C under Ar. The resulting solution was stirred at –78 °C for 10 min, and acetaldehyde (1.04 g, 1.3 mL, 23.74 mmol) was added dropwise. The resulting solution was stirred at –78 °C for 10 min and then allowed to warm to rt. Then, saturated $NH_4.Cl_{(aq)}$ (15 mL) was added. The layers were separated, extracting the aqueous with Et_2O (3 × 20 mL). The combined organic layers were dried ($MgSO_4$) and evaporated under reduced pressure to give the crude product. Purification by flash column chromatography on silica with 10:1→5:1 hexane:Et_2O as eluent gave oct-3-yn-2-ol (2.11 g, 92%) as a colourless oil, R_F 0.2 (10:1 hexane:Et_2O); IR (film) 3338 (OH), 2958, 2933, 2873, 2212, 1667, 1456, 1362, 1328, 1228, 1155, 1073, 1007, 968, 893, 859, 728, 658, 584 cm^{-1}; ^1H NMR (300 MHz, $CDCl_3$) δ H 5.06-4.04 (1H, m, OCH), 2.19 (2H, app. td, J = 6.9, 1.9 Hz, ≡CCH_2), 1.87 (1H, d, J = 5.2 Hz, OH), 1.54-1.31 (4H, m, MeCH_2 + $MeCH_2CH_2$), 1.41 (3H, d, J = 6.5 Hz, OCH*Me*), 0.90 (3H, t, J = 7.1 Hz, CH_2*Me*); ^{13}C NMR (75 MHz, $CDCl_3$) δ C 84.7 (≡C), 82.2 (≡C), 58.6 (OCH), 30.8 (CH_2), 24.8 (Me), 22.0 (CH_2), 18.4 (CH_2), 13.6 (Me); HRMS (APCI) $C_8H_{13}O$ ($[M–H]^+$) requires 125.0961, found 125.0958.

(*E*)-Oct-3-en-2-ol, 4c

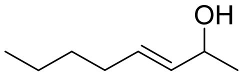

4c

A solution of oct-3-yn-2-ol (1.0 g, 7.92 mmol) in THF (10 mL) was added dropwise to a stirred suspension of $LiAlH_4$ (601 mg, 15.84 mmol) in THF (10 mL) at rt under Ar. The resulting solution was stirred under reflux under Ar for 4 h. Then, the solution was allowed to cool to rt and 1 M $NaOH_{(aq)}$ was added dropwise until a white precipitate formed. The solids were filtered off over Celite®, washing with Et_2O. The filtrate was evaporated under reduced pressure to give the crude product. Purification by flash column chromatography on silica with 9:1 hexane:Et_2O as eluent gave the title product **4c** (894 mg, 88%) as a colourless oil, R_F 0.1 (9:1 hexane:Et_2O); IR (film) 3371 (OH), 2959, 2928, 2873, 1714, 1669, 1625, 1457, 1365, 1256, 1179, 1149, 1123, 1060, 969, 947, 921, 864, 737, 609, 578 cm^{-1}; ^1H NMR (300 MHz, $CDCl_3$) δ H 5.61 (1H, d br t, J = 15.4, 5.9 Hz, CH_2C*H*=), 5.48 (1H, dd br t, J = 15.4, 6.2, 1.2 Hz, OCHC*H*=), 4.23 (1H, dq, J = 6.1, 6.1 Hz, OCH), 2.00 (2H, app. q, J = 6.8 Hz, =CHCH_2), 1.75 (1H, br s, OH), 1.37-1.27 (4H, m, MeCH_2 + $MeCH_2CH_2$), 1.23 (3H, d, J =

6.1 Hz, OCH*Me*), 0.87 (3H, t, *J* = 7.3 Hz, *Me*CH_2); ^{13}C NMR (75 MHz, $CDCl_3$) δ C 134.2 (=CH), 131.2 (=CH), 69.0 (OCH), 31.9 (CH_2), 31.4 (CH_2), 23.5 (OCH*Me*), 22.3 (CH_2), 14.0 (CH_2*Me*); HRMS (APCI) C8H15O ($[M–H]^+$) requires 127.1117, found 127.1114; CSP-GC (β-Dex, 80 °C, 35 cm s^{-1}) (*R*)-**4c** 25.41 min, (*S*)-**4c** 26.22 min.

(*R*,*E*)-Oct-3-en-2-ol (*R*)-4c

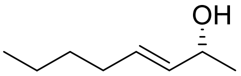

(*R*)-**4c**

Following general procedure **A**, *rac*-**4c** (300 mg, 2.34 mmol), $Ti(O^iPr)_4$ (720 mg, 823 μL, 2.81 mmol), L-(+)-DIPT (692 mg, 575 μL, 2.81 mmol) and TBPH (233 μL of a 6.0 M solution in dioxane, 1.40 mmol) in CH_2Cl_2 (23 mL) then $FeSO_4$ (6.66 g) and citric acid (2.41 g) in H_2O (22 mL) gave the crude product. Purification by flash column chromatography on silica with 9:1 hexane:Et_2O as eluent gave (*R*)-**4c** (97 mg, 32%, >99:1 er by CSP-GC) as a colourless oil, $[\alpha]_D$ = +10.0 (*c* = 0.2 in $CHCl_3$).

(*E*)-1-Cyclohexylbut-2-en-1-ol 4d

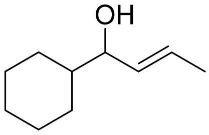

4d

Li (685 mg of a 25% weight dispersion in mineral oil, 24.68 mmol) was added to THF (20 mL) at rt under Ar. The resulting suspension was stirred vigerously at rt for 30 min. Then the suspension was cooled to 0 °C and a solution of bromocyclohexane (1.0 g, 735 μL, 6.17 mmol) and crotonaldehyde (540 mg, 639 μL, 7.71 mmol) in THF (20 mL) was added. The resulting solution was stirred at 0 °C for 30 min then allowed to warm to rt. Then, a saturated solution of $NaHCO_{3(aq)}$ (30 mL) was added. The layers were separated, extracting the aqueous with Et_2O (3 × 40 mL). The combined organic layers were dried ($MgSO_4$) and evaporated under reduced pressure to give the crude product. Purification by flash column chromatography on silica with 9:1→5:1 hexane:Et_2O as eluent gave the title product **4d** (371 mg, 39%) as a colourless oil, R_F 0.2 (9:1 hexane:Et_2O); IR (film) 3354 (OH), 2921, 2851, 1449, 1377, 1307, 1261, 1066, 1000, 949, 928, 891, 842, 643, 586, 558 cm^{-1}; ^{1}H NMR (300 MHz, $CDCl_3$) δ H 5.61 (1H, dqd, *J* = 15.3, 6.5, 0.7 Hz, Me*H*C=), 5.45 (1H, ddq, *J* = 15.3, 7.3, 1.4 Hz, MeCH=C*H*), 3.73 (1H, app. T, *J* = 7.3 Hz, OHC*H*), 1.89-1.54 (m, 9H), 1.43-0.83 (m, 6H); ^{13}C NMR (75 MHz, $CDCl_3$) δ C 132.9 (HC=), 127.6 (HC=), 77.8 (HOCH), 43.8 (*C*HCHOH), 28.9 (CH_2), 28.8 (CH_2), 26.7 (CH_2), 26.3 (CH_2), 26.2 (CH_2), 17.8 (*Me*HC=); HRMS (APCI) C10H20N ($[M–H_2O+NH_4]^+$) requires 154.1590, found 154.1586; CSP-GC (Chiraldex-GTA, 100 °C, 35 cm s^{-1}) (*R*)-**4d** 28.22 min, (*S*)-**4d** 29.26 min.

(*R*, *E*)-1-Cyclohexylbut-2-en-1-ol (*R*)-4d

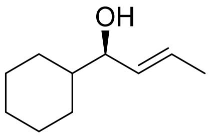

(*R*)-**4d**

Following general procedure **A**, *rac*-**4d** (200 mg, 1.13 mmol), $Ti(O^iPr)_4$ (439 mg, 457 µL, 1.56 mmol), L-(+)-DIPT (384 mg, 319 µL, 1.56 mmol) and TBHP (130 µL of a 6.0 M solution in dioxane, 0.78 mmol) in CH_2Cl_2 (13 mL) then $FeSO_4.7H_2O$ (3.7 g) and citric acid (1.34 g) in H_2O (12 mL) gave the crude product. Purification by flash column chromatography on silica with 9:1→5:1 hexane:Et_2O as eluent gave (*R*)-**4d** (67 mg, 33%, >99:1 er by CSP-GC) as a colourless oil, $[\alpha]_D = -1.0$ (c = 2.0 in $CHCl_3$).

4-Cyclohexylbut-3-yn-2-ol

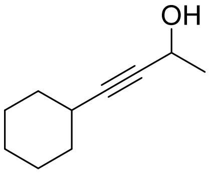

^nBuLi (15.01 mL of a 1.6M solution in hexanes, 24.02 mmol) was added dropwise to a stirred solution of cyclohexylacetylene (2.0 g, 2.41 mL, 18.48 mmol) in THF (20 mL) at –78 °C under Ar. The resulting solution was stirred at –78 °C for 10 min and acetaldehyde (1.06 g, 1.36 mL, 24.02 mmol) was added. The resulting solution was stirred at –78 °C for 10 min and then allowed to warm to rt. Saturated $NH_4Cl_{(aq)}$ (20 mL) was then added and the layers were separated, extracting the aqueous with Et_2O (3 × 20 mL). The combined organic layers were dried ($MgSO_4$) and evaporated under reduced pressure to give the crude product. Purification by flash column chromatography on silica with 9:1→4:1 pentane:Et_2O as eluent gave 4-cyclohexylbut-3-yn-2-ol (2.41 g, 86%) as a colourless oil, R_F 0.2 (9:1 pentane:Et_2O); IR (film) 3327 (OH), 2927, 2853, 1448, 1368, 1327, 1296, 1157, 1132, 1111, 1076, 1017, 975, 896, 878, 844, 634, 585, 573 cm^{-1}; ^1H NMR (300 MHz, $CDCl_3$) δ H 4.50 (1H, dqd, J = 6.5, 5.0, 1.6 Hz, OCH), 2.40-2.27 (1H, m, ≡CCH), 2.15 (1H, br d, J = 6.5 Hz, OH), 1.82-1.58 (4H, m, Cy), 1.50-1.23 (6H, m, Cy), 1.40 (3H, d, 5.0 Hz, Me); ^{13}C NMR (75 MHz, $CDCl_3$) δ C 88.7 (C≡), 82.3 (C≡), 58.6 (OCH), 32.7 (CH_2), 29.0 (CH), 25.9 (CH_2), 25.0 (Me), 24.9 (CH_2). Spectroscopic data consistent with that previously reported.[3]

(*E*)-4-Cyclohexylbut-3-en-2-ol, 4e

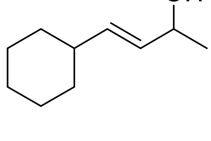

4e

A solution of 4-cyclohexylbut-3-yn-2-ol (1.8 g, 11.82 mmol) in THF (10 mL) was added dropwise to a stirred suspension of $LiAlH_4$ (897 mg, 23.64 mmol) in THF (10 mL) at rt under Ar. The resulting solution was stirred under reflux under Ar for 4 h. Then, the solution was allowed to cool to rt and 1 M $NaOH_{(aq)}$ was added dropwise until a white precipitate formed. The solids were filtered off over Celite®, washing with Et_2O. The filtrate was evaporated under reduced pressure to give the crude product. Purifcation by flash column chromatography on silica with 9:1→5:1 pentane:Et_2O as eluent gave the title product **4e** (1.43 g, 78%) as a colourless oil, R_F 0.1 (9:1 pentane:Et_2O); IR (film) 3331 (OH), 2969, 2921, 2850, 1448, 1367, 1130, 1056, 966, 941, 892, 864, 842, 569 cm^{-1}; ^1H NMR (300 MHz, $CDCl_3$) δ H 5.54 (1H, dd, J = 15.7, 6.3 Hz, =CH), 5.42 (1H, ddd, J = 15.7, 6.2, 0.7 Hz, =CH), 4.21 (1H, dq, J = 6.3, 6.3 Hz, OCH), 1.97-1.81 (1H, m, =CHC*H*), 1.83 (1H, br s, OH), 1.75-1.58 (4H, m, Cy), 1.33-0.94 (6H, m, Cy), 1.21 (3H, d, J = 6.3 Hz, Me); ^{13}C NMR (75 MHz, $CDCl_3$) δ 136.8 (=CH), 131.7 (=CH), 69.1 (OCH), 40.2 (CH), 32.9 (CH_2), 26.2 (CH_2), 26.1 (CH_2), 23.5 (Me); CSP-HPLC (Chiralcel OD-H, 99:1 hexane:IPA, 1.0 mL min^{-1}) (*R*)-**4e** 9.35 min, (*S*)-**4e** 10.15 min. Spectroscopic data consistent with that previously reported.[4]

(*R*, *E*)-4-Cyclohexylbut-3-en-2-ol, (*R*)-4e

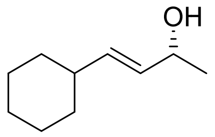

(*R*)-**4e**

Following general procedure **A**, *rac*-**4e** (600 mg, 3.89 mmol), $Ti(O^iPr)_4$ (1.33 g, 1.38 mL, 4.67 mmol), L-(+)-DIPT (1.15 g, 950 μL, 4.67 mmol) and TBHP (388 μL of a 6.0 M solution in dioxane, 2.33 mmol) in CH_2Cl_2 (40 mL) then $FeSO_4$ (11.71 g) and citric acid (4.29 g) in H_2O (40 mL) gave the crude product. Purification by flash column chromatography on silica with 9:1→5:1 pentane:Et_2O as eluent gave (*R*)-**4e** (280 mg, 47%, >99:1 er by CSP-HPLC) as a colourless oil, $[\alpha]_D$ = 10.8 (c = 2.03 in $CHCl_3$).

(*E*)-5-Methylhex-3-en-2-ol, 4f

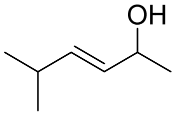

4f

MeLi (16.6 mL of a 1.6 M solution in Et_2O, 26.49 mmol) was added dropwise to a stirred solution of 4-methyl-2-pentenal (2.0 g, 1.37 mL, 20.38 mmol) in THF (15 mL) at 0 °C under Ar. The resulting solution was stirred at 0 °C for 30 min. Then, the resulting solution was allowed to warm to rt, and saturated $NH_4Cl_{(aq)}$ (10 mL) was added. The layers were separated, extracting the aqueous with Et_2O (3 × 15 mL). The combined organic layers were dried ($MgSO_4$) and evaporated under reduced pressure to give the crude product. Purification by flash column chromatography on silica with 9:1→5:1 hexane:Et_2O as eluent gave the title

product **4f** (1.66 g, 71%) as a colourless oil, R_F0.1 (9:1 hexane:Et_2O); IR (film) 3335, 2960, 2929, 2869, 1464, 1382, 1365, 1293, 1154, 1119, 1089, 1055, 969, 932, 870, 833, 624 cm^{-1}; ^{1}H NMR (300 MHz, $CDCl_3$) δ H 5.57 (1H, ddd, *J* = 15.5, 6.4, 0.9 Hz, HC=), 5.42 (1H, ddd, *J* = 15.5, 6.4, 1.1 Hz, HC=), 4.22 (1H, dq, *J* = 6.4, 6.4 Hz, C*H*OH), 2.24 (1H, d of septets, *J* = 6.4, 6.4 Hz, C*H*Me_2), 1.81 (1H, br s, OH), 1.22 (3H, d, *J* = 6.4 Hz, Me), 0.96 (6H, dd, *J* = 6.4, 1.1 Hz, =C*H*^{i}Pr); ^{13}C NMR (75 MHz, $CDCl_3$) δ C 138.0 (HC=), 131.3 (HC=), 69.0 (CHOH), 30.6 (*C*HMe_2), 23.6 (*Me*CHOH), 22.4 (2 × CH*Me*$_2$); HRMS (APCI) $C_7H_{13}O$ ($[M–H]^+$) requires 113.0961, found 113.0956; CSP-GC (Chiraldex-GTA, 60 °C, 35 cm s^{-1}) (*S*)-**4f** 10.83 min, (*R*)-**4f** 11.29 min.

(*R*, *E*)-5-Methylhex-3-en-2-ol, (*R*)-4f

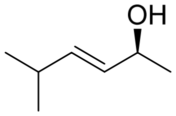

(*R*)-**4f**

Following general procedure **B**, *rac*-**4f** (1.0 g, 8.76 mmol), AmanoAK (300 mg), vinyl acetate (2.04 g, 2.18 mL, 23.65 mmol) and 4 Å molecular sieves (500 mg) in hexane (33 mL) gave the crude product. Purification by flash column chromatography on silica with 9:1→5:1 pentane:Et_2O as eluent gave (*R*)-**4f** (345 mg, 34%, >99:1 er by CSP-GC) as a colourless oil, $[\alpha]_D$ = –8.6 (*c* = 1.85 in $CHCl_3$).

(*E*)-1-Phenylpent-3-en-2-ol, 4g

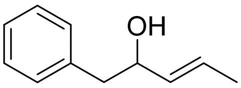

4g

BnMgBr (7.99 mL of a 2.0 M solution in THF, 15.98 mmol) was added dropwise to a stirred solution of crotonaldehyde (1.0 g, 1.18 mL, 14.27 mmol) in THF (20 mL) at 0 °C under Ar. The resulting solution was stirred at 0 °C for 30 min and saturated $NH_4.Cl_{(aq)}$ (20 mL) was added. The layers were separated, extracting the aqueous with Et_2O (3 × 30 mL). The combined organic layers were dried ($MgSO_4$) and evaporated under reduced pressure to give the crude product. Purification by flash column chromatography on silica with 9:1→4:1 pentane:Et_2O as eluent gave the title product **4g** (783 mg, 34%) as a colourless oil, R_F 0.1 (9:1 pentane:Et_2O); IR (film) 3373 (OH), 3027, 2916, 1494, 1453, 1377, 1265, 1116, 1085, 1029, 963, 909, 857, 826, 744, 698, 604 cm^{-1}; ^{1}H NMR (300 MHz, $CDCl_3$) δ H 7.44-7.21 (5H, m, Ar), 5.71 (1H, dqd, *J* = 15.3, 6.3, 0.9 Hz, MeC*H*=), 5.58 (1H, ddq, *J* = 15.3, 6.4, 1.3 Hz, OCHC*H*=), 4.31 (1H, app. br q, *J* = 6.4 Hz, OCH), 2.88 (1H, dd, *J* = 13.5, 5.3 Hz, ArC*H*$_A$H$_B$), 2.80 (1H, dd, *J* = 13.5, 7.7 Hz, ArCH$_A$*H*$_B$), 1.88 (1H, br s, OH), 1.73 (3H, d, *J* = 6.3 Hz, Me); ^{13}C NMR (75 MHz, $CDCl_3$) δ C 138.2 (*ipso*-Ar), 133.3 (Ar), 129.6 (Ar), 128.4 (Ar), 127.0 (=CH), 126.4 (=CH), 73.6 (OCH), 44.2 (CH_2), 17.7 (Me); HRMS (APCI) $C_{11}H_{13}O$ ($[M–H]^+$) requires 161.0961, found 161.0957; CSP-GC (β-Dex, 115 °C, 35 cm s^{-1}) (*R*)-**4g** 56.67 min, (*S*)-**4g** 57.86 min.

(*R*, *E*)-2-Phenylpent-3-en-2-ol, (*R*)-4g

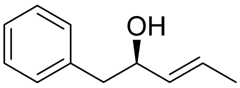

(*R*)-**4g**

Following general procedure **A**, *rac*-**4g** (783 mg, 6.83 mmol), Ti(O^{i}Pr)$_4$ (1.65 g, 1.72 mL, 5.80 mmol), L-(+)-DIPT (1.43 g, 1.19 mL, 5.80 mmol) and TBHP (483 µL of a 6.0 M solution in dioxane, 2.90 mmol) in CH_2Cl_2 (50 mL) then $FeSO_4$ (14.5 g) and citric acid (5.31 g) in H_2O (50 mL) gave the crude product. Purification by flash column chromatography on silica with 9:1→4:1 pentane:Et_2O as eluent gave pentenol (*R*)-**4g** (210 mg, 27%, >99:1 er by CSP-GC) as a colourless oil, $[\alpha]_D = 1.5$ ($c = 1.35$ in $CHCl_3$).

(*E*)-1-Phenylbut-2-en-1-ol, 4h

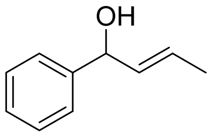

4h

PhMgBr (39.9 mL of a 1.0 M solution in THF, 39.9 mmol) was added dropwise to a stirred solution of crotonaldehyde (2.0 g, 2.36 mL, 28.53 mmol) in THF (20 mL) at 0 °C under Ar. The resulting solution was stirred at 0 °C for 30 min. The resulting solution was warmed to rt and saturated $NH_4.Cl_{(aq)}$ (75 mL) was added. The layers were separated, extracting the aqueous with Et_2O (3 × 50 mL). The combined organic layers were washed with brine (2 × 50 mL), dried (Na_2SO_4) and evaporated under reduced pressure to give the crude product. Purificatin by flash column chromatography on silica with 9:1→5:1 petrol:Et_2O gave butenol **4h** (1.6 g, 38%) as a colourless oil, R_F 0.2 (5:1 petrol:Et_2O); IR (film) 3339 (OH), 3030, 2966, 2855, 1671, 1491, 1449, 1377, 1193, 1068, 1003, 962, 911, 845, 746, 692, 630 cm^{-1}; ^{1}H NMR (300 MHz, $CDCl_3$) δ H 7.41-7.23 (5H, m, Ar), 5.70-5.59 (2H, m, 2 × CH=), 5.16 (1H, d, J = 6.0 Hz, C*H*OH), 1.95 (1H, br s, OH), 1.71 (3H, d, J = 6.0 Hz, =CHC*H*$_3$); ^{13}C NMR (75 MHz, $CDCl_3$) δ(75 MHz, $CDCl_3$) δ C 143.5 (*ipso*-Ar), 133.8 (CH), 128.7 (CH), 127.7 (CH), 127.7 (CH), 126.3 (CH), 75.3 (OCH), 17.9 (Me); CSP-HPLC (Chiralcel OD-H, 95:5 hexane:IPA, 1.0 mL min^{-1}) (*S*)-**4h** 7.63 min, (*R*)-**4h** 9.26 min.

(*R*, *E*)-1-phenylbut-2-en-1-ol (*R*)-4h

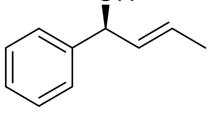

(*R*)-4h

L-(+)-DIPT (200 mg, 165 µL, 0.81 mmol) was added dropwise to a stirred solution of *rac*-**4h** (400 mg, 2.70 mmol) and 4 Å molecular sieves (205 mg) in CH_2Cl_2 (11 mL) at –20 °C under Ar. The resulting solution was stirred at –20 °C for 30 min. Then, Ti(O^{i}Pr)$_4$ (230 mg, 240 µL,

0.81 mmol) was added dropwise. The resulting solution was stirred at –20 °C for 45 min. Then, TBHP (300 µL of a 6 M solution in dioxane, 1.8 mmol) was added dropwise. The resulting solution was stirred at –20 °C for 16 h. Then, a solution of $FeSO_4.7H_2O$ (1.67 g) and tartaric acid (0.57 g) in H_2O (11 mL) was added. The resulting solution was allowed to warm to rt and stirred vigorously until two layers were evident. The layers were separated, extracting the queous with CH_2Cl_2 (3 × 25 mL). The combined organic layers were dried (Na_2SO_4) and evaporated under reduced pressure to give the crude product. Purification by flash column chromatography on silica with 9:1→5:1 petrol:Et_2O gave (*R*)-**4h** (157 mg, 39%, 91:9 er by CSP-HPLC) as a colourless oil, $[\alpha]_D$ = +9.3 (*c* = 1.0 in $CHCl_3$).

(*E*)-4-Phenylbut-3-en-2-ol 4i

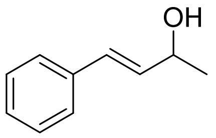

4i

$NaBH_4$ (22.4 g, 590 mmol) in MeOH (85 mL) was added to 4-phenylbut-3-en-2-one (8.65 g 59.17 mmol) in MeOH (85 mL) at 0 °C under Ar. The resulting solution was warmed to rt and stirred for 16 h. Then, saturated $NH_4.Cl_{(aq)}$ (45 mL) was added. MeOH was removed under reduced pressure, and the remaining aqueous solution extracted with Et_2O (3 × 140 mL). the combined organic layers were dried ($MgSO_4$) and evaporated under reduced pressure to give the crude product. Purfication by flash column chromatography on silica with 3:1 → 1:1 petrol:Et_2O as eluent gave butenol **4i** (7.29 g, 83%) as a colourless oil, R_F 0.3 (3:1 petrol:Et_2O); IR (film) 3422 (OH), 2977, 2966, 1492, 1447, 1412, 1359, 1305, 1142, 1059, 1029, 974, 964, 935, 912, 876, 824, 751, 690, 603, 561 cm^{-1}; ^{1}H NMR (300 MHz, $CDCl_3$) δ H 7.36-7.14 (5H, m, Ar), 6.51 (1H, br d, *J* = 16.1 Hz, ArC*H*=), 6.21 (1H, dd, *J* = 16.1, 6.4 Hz, OCHC*H*=), 4.44 (1H, dqd, *J* = 6.4, 6.4, 1.2 Hz, OCH), 1.73 (1H, br s, OH), 1.32 (3H, d, *J* = 6.4 Hz, Me); ^{13}C NMR (75 MHz, $CDCl_3$) δ C 136.8 (*ipso*-Ar), 133.7 (CH), 129.5 (CH), 128.7 (CH), 127.8 (CH), 126.6 (CH), 69.1 (OCH), 23.6 (Me); CSP-HPLC (Chiralcel OD-H, 95:5 hexane:IPA, 1.0 mL min^{-1}) (*R*)-**4i** 11.16 min, (*S*)-**4i** 16.65 min.

(*S*, *E*)-4-Phenylbut-3-en-2-ol (*S*)-4i

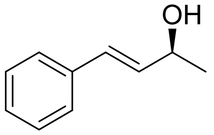

(*S*)-4i

Using general procedure **B**, butenol **4i** (1.0 g, 6.75 mmol), vinyl acetate (1.57 g, 1.68 mL, 18.22 mmol), AmanoAK (500 mg) and 4 Å molecular sieves (500 mg) in hexane (100 mL) gave the crude product. Purification by flash colum chromatography on silica with 3:1 → 1:1 petrol:Et_2O as eluent gave butenol (*S*)-**4i** (494 g, 49%, >99:1 er by CSP-HPLC) as a colourless oil, $[\alpha]_D$ = –23.8 (*c* = 10 in $CHCl_3$).

(*S*)-(But-3-yn-2-yloxy)(*tert*-butyl)dimethylsilane[6]

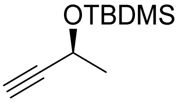

(*S*)-1-Methylbut-3-yne (1.0 g, 14.27 mmol) was added to a stirred solution of TBDMSCl (2.19 g, 14.56 mmol) and imidazole (991 mg, 14.56 mmol) in Et_2O (16 mL) at rt under Ar. The resulting solution was stirrer at rt for 16 h. Then, saturated $NH_4.Cl_{(aq)}$ (11 mL) was added. The layers were separated, extracting the aqueous with Et_2O (3 × 15 mL). The combined organic layers were dried ($MgSO_4$) and evaporated under reduced pressure to give the crude product. Purification by flash column chromatography on silica with 99:1 pentane:Et_2O as eluent gave (*S*)-(but-3-yn-2-yloxy)(*tert*-butyl)dimethylsilane (1.73 g, 66%) as a colourless oil, R_F 0.4 (99:1 pentane:Et_2O); IR (film) 2956, 2930, 2858, 1473, 1251, 1121, 1102, 1058, 974, 835, 804, 776, 654, 628 cm^{-1}; ^1H NMR (300 MHz, $CDCl_3$) δ H 4.51 (1H, qd, *J* = 6.5, 2.1 Hz, OCH), 2.36 (1H, d, *J* = 2.1 Hz, ≡CH), 1.42 (3H, d, *J* = 6.5 Hz, OCH*Me*), 0.90 (9H, s, ^tBu), 0.13 (3H, s, SiMe), 0.10 (3H, s, SiMe); ^{13}C NMR (75 MHz, $CDCl_3$) δ C 86.6 (≡C), 71.3 (≡CH), 58.9 (OCH), 25.9 (C*Me*$_3$), 25.5 (OCH*Me*), 18.4 (*C*Me$_3$), –4.5 (SiMe), –4.9 (SiMe); HRMS (APCI) $C_{10}H_{21}OSi$ ($[M+H]^+$) requires 185.1356, found 185.1352.

4-(*tert*-Butyldimethylsilyloxy)pent-2-yn-1-ol[6]

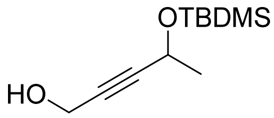

^nBuLi (4.57 mL of a 1.6 M solution in hexanes, 7.32 mmol) was added to a stirred solution of 2-*tert*-butyldimethylsiloxybut-3-yne (1.0 g, 5.42 mmol) in THF (6 mL) at –78 °C under Ar. The resulting solution was stirred at –78 °C for 30 min and a suspension of paraformaldehyde (244 mg, 8.13 mmol) in THF (4 mL) was added. The resulting solution was stirred, allowing to warm from –78 °C to rt over 90 min. Then, saturated $NH_4Cl_{(aq)}$ (10 mL) was added. The layers were separated, extracting the aqueous with Et_2O (3 × 15 mL). The combined organic layers were dried ($MgSO_4$) and evaporated under reduced pressure to give the crude product. Purification by flash column chromatography on silica with 9:1→4:1 pentane:Et_2O as eluent gave 4-(*tert*-butyldimethylsilyloxy)pent-2-yn-1-ol (965 mg, 83%) as a colourless oil, R_F 0.1 (9:1 pentane:Et_2O); IR (film) 3339, 2955, 2930, 2857, 1472, 1463, 1389. 1361, 1340, 1316, 1253, 1153, 1100, 1087, 1031, 1004, 946, 829, 812, 776, 666 cm^{-1}; ^1H NMR (300 MHz, $CDCl_3$) δ H 4.53 (1H, qt, *J* = 6.5, 1.7 Hz, OCH), 4.25 (2H, dd, *J* = 5.8, 1.7 Hz, OCH_2), 2.21 (1H, t, *J* = 5.8 Hz, OH), 1.39 (3H, d, *J* = 6.5 Hz, OCH*Me*), 0.88 (9H, s, C*Me*$_3$), 0.10 (3H, s, SiMe), 0.09 (3H, s, SiMe); ^{13}C NMR (75 MHz, $CDCl_3$) δ C 88.2 (≡C), 81.5 (≡C), 59.1 (OCH), 51.1 (OCH_2), 25.9 (C*Me*$_3$), 25.4 (OCH*Me*), 18.3 (*C*Me$_3$), –4.5 (SiMe), –4.8 (SiMe); HRMS (APCI) $C_{11}H_{22}O_2NSi$ ($[M+NH_4]^+$) requires 232.1727, found 232.1727.

(*S*)-4-(*tert*-Butyldimethylsilyloxy)pent-2-yn-1-ol[6]

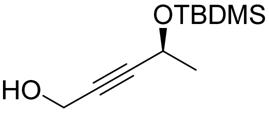

^{n}BuLi (7.91 mL of a 1.6 M solution in hexanes, 12.66 mmol) was added dropwise to a stirred solution of (*S*)-(but-3-yn-2-yloxy)(*tert*-butyl)dimethylsilane (1.73 g, 9.38 mmol) in THF (12 mL) at –78 °C under Ar. The resulting solution was stirred at –78 °C for 30 min and a suspension of paraformaldehyde (380 mg, 812.66 mmol) in THF (8 mL) was added. The resulting solution was stirred, allowing to warm from –78 °C to rt over 90 min. Then, saturated $NH_4Cl_{(aq)}$ (10 mL) was added. The layers were separated, extracting the aqueous with Et_2O (3 × 30 mL). The combined organic layers were dried ($MgSO_4$) and evaporated under reduced pressure to give the crude product. Purification by flash column chromatography on silica with 9:1→4:1 pentane:Et_2O as eluent gave (*S*)-4-(*tert*-butyldimethylsilyloxy)pent-2-yn-1-ol (1.49 g, 74%) as a colourless oil.

(*E*)-5-(Benzyloxy)pent-3-en-2-ol, 4j[6]

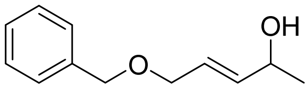

4j

A solution of 4-(*tert*-butyldimethylsilyloxy)pent-2-yn-1-ol (965 mg, 4.50 mmol) in THF (13 mL) was added dropwise to a stirred solution of $LiAlH_4$ (256 mg, 6.75 mmol) in THF (13 mL) at 0 °C under Ar. The resulting solution was allowed to warm to rt and stirred for 1 h. Then, 1 M $NaOH_{(aq)}$ was added dropwise until a white precipitate formed. The solids were filtered off over Celite®, washing with Et_2O. Then, the filtrate was evaporated under reduced pressure. The residue was taken up in THF (8 mL) and added dropwise to a stirred solution of NaH (360 mg of a 60% suspension in mineral oil, 9.0 mmol) in THF (11 mL) at rt under Ar. The resulting solution was stirred at rt for 1 h. Then, $^{n}Bu_4NI$ (81 mg, 0.22 mmol) followed by BnCl (1.71 g, 1.55 mL, 13.50 mmol) were added. The resulting solution was stirred at rt for 16 h and H_2O was added. The layers were separated, extracting the aqueous with Et_2O (3 × 10 mL). The combined organic layers were dried ($MgSO_4$) and evaporated under reduced pressure. The residue was passed over a plug of silica, eluting with 4:1 hexane:EtOAc. The filtrate was evaporated under reduced pressure. The residue was stirred in THF (20 mL) and TBAF (6.75 mL of a 1.0 M solution in THF, 6.75 mmol) was added. The resulting solution was stirred at rt for 2 h. H_2O (20 mL) was added, and the layers were separated, extracting the aqueous with EtOAc (3 × 15 mL). The combined organic layers were dried ($MgSO_4$) and evaporated under reduced pressure to give the crude product. Purification by flash column chromatography on silica with 4:1→2:1 pentane:Et_2O as eluent gave **4j** (327 mg, 38%) as a colouress oil, R_F 0.4 (2:1 pentane:Et_2O); IR (film) 3405 (OH), 2972, 2855, 1496, 1453, 1371, 1240, 1116, 1046, 1027, 969, 863, 845, 735, 697, 607 cm^{-1}; ^{1}H NMR (300 MHz, $CDCl_3$) δ H 7.36-7.15 (5H, m, Ar), 5.75-5.67 (2H, m, 2 × =CH), 4.44 (2H, s, ArCH_2), 4.29-4.18 (1H, m, OCH), 3.96-3.90 (=CHCH_2), 1.88 (1H, br s, OH), 1.19 (3H, d, J = 6.4 Hz, =CH*Me*); ^{13}C

NMR (75 MHz, $CDCl_3$) δ C 138.2 (*ipso*-Ar), 137.3 (CH), 128.5 (CH), 127.8 (CH), 127.7 (CH), 126.1 (CH), 72.3 (CH_2), 70.2 (CH_2), 68.2 (OCH), 23.3 (Me). Spectroscopic data consistent with that previously reported.[6]

(*S*,*E*)-5-(Benzyloxy)pent-3-en-2-ol, (*S*)-4j[6]

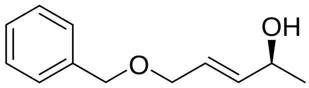

(*S*)-**4j**

A solution of (*S*)-4-(*tert*-butyldimethylsilyloxy)pent-2-yn-1-ol (1.49 g, 6.95 mmol) in THF (20 mL) was added dropwise to a stirred solution of $LiAlH_4$ (395 mg, 10.42 mmol) in THF (20 mL) at 0 °C under Ar. The resulting solution was allowed to warm to rt and stirred for 1 h. Then, 1 M $NaOH_{(aq)}$ was added dropwise until a white precipitate formed. The solids were filtered off over Celite®, washing with Et_2O. Then, the filtrate was evaporated under reduced pressure. The residue was taken up in THF (12 mL) and added dropwise to a stirred solution of NaH (556 mg of a 60% suspension in mineral oil, 13.9 mmol) in THF (17 mL) at rt under Ar. The resulting solution was stirred at rt for 1 h. Then, $^nBu_4NI$ (126 mg, 0.34 mmol) followed by BnCl (2.64 g, 2.40 mL, 20.83 mmol) were added. The resulting solution was stirred at rt for 16 h and H_2O was added. The layers were separated, extracting the aqueous with Et_2O (3 × 16 mL). The combined organic layers were dried ($MgSO_4$) and evaporated under reduced pressure. The residue was passed over a plug of silica, eluting with 4:1 hexane:EtOAc. The filtrate was evaporated under reduced pressure. The residue was stirred in THF (30 mL) and TBAF (10.42 mL of a 1.0 M solution in THF, 10.42 mmol) was added. The resulting solution was stirred at rt for 2 h. H_2O (30 mL) was added, and the layers were separated, extracting the aqueous with EtOAc (3 × 20 mL). The combined organic layers were dried ($MgSO_4$) and evaporated under reduced pressure to give the crude product. Purification by flash column chromatography on silica with 4:1→2:1 pentane:Et_2O as eluent gave (S)-**4j** (223 mg, 17%) as a colouress oil, $[\alpha]_D = -13.4$ (c = 2.98 in $CHCl_3$).

(*R*, *E*)-Pent-3-en-2-ol, (*R*)-4k

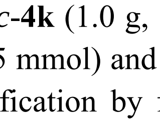

(*R*)-**4k**

Following general procedure **B**, *rac*-**4k** (1.0 g, 1.19 mL, 11.61 mmol), AmanoAK (300 mg), vinyl acetate (2.7 g, 2.89 mL, 31.35 mmol) and 4 Å molecular sieves (500 mg) in hexane (43 mL) gave the crude product. Purification by flash column chromatography on silica with 7:1→3:1 pentane:Et_2O as eluent gave (*R*)-**4k** (231 mg, 23%, >99:1 er by CSP-GC) as a colourless oil, $[\alpha]_D = -10.0$ (c = 1.0 in $CHCl_3$).

(*E*)-2,2-Dimethylhex-4-en-3-ol, 4l

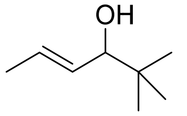

4l

^{t}BuLi (19.5 mL of a 1.9 M colution in hexanes, 37.09 mmol) was added dropwise to a stirred solution of crotonaldehyde in THF (15 mL) at 0 °C under Ar. The resulting solution was stirred at 0 °C for 30 min. Then, the solution was allowed to warm to rt and saturated $NH_4.Cl_{(aq)}$ (20 mL) was added. The layers were separated, extracting the aqueous with Et_2O (3 × 20 mL). The combined organic layers were dried ($MgSO_4$) and evaporated under reduced pressure to give the crude product. Purification by flash column chromatography on silica with 9:1→5:1 hexane:Et_2O as eluent gave the title product **4l** (821 mg, 22%) as a colourless oil, R_F 0.3 (9:1 hexane:Et_2O); IR (film) 3427 (OH), 2953, 2917, 2869, 1478, 1462, 1447, 1393, 1378, 1363, 1295, 1239, 1216, 1184, 1119, 1085, 1032, 1016, 995, 967, 925, 896, 751, 612, 593, 583, 572, 562 cm^{-1}; ^{1}H NMR (300 MHz, $CDCl_3$) δH 5.65 (1H, dqd, *J* = 15.3, 5.9, 0.5 Hz, =C*H*Me), 5.53 (1H, ddq, *J* = 15.3, 7.5, 1.3 Hz, OHCHC*H*=), 3.67 (1H, d, *J* = 7.5 Hz, OHC*H*), 1.70 (3H, dd, *J* = 5.9, 1.3 Hz, =CHMe), 1.50 (br s, 1H, OH), 0.89 (s, 9H, C*Me*$_3$); ^{13}C NMR (75 MHz, $CDCl_3$) δC 131.1 (HC=), 128.4 (HC=), 81.2 (CHOH), 34.8 (*C*Me$_3$), 25.8 (C*Me*$_3$), 17.9 (=CH*Me*); HRMS (APCI) $C_8H_{15}O$ ([M–H]$^+$) requires 127.1117, found 127.1115; CSP-GC (Chiraldex-GTA, 50 °C, 20 cm s^{-1}) (*S*)-**4l** 64.65 min, (*R*)-**4l** 66.62 min.

(*R*, *E*)-2,2-Dimethylhex-4-en-3-ol, (*R*)-4l[2]

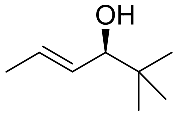

(*R*)-**4l**

A 0.6 M solution of (*Z*)-propenyllithium was prepared by addition of a solution of *Z*-bromopropene (1.05 g, 747 µL, 8.7 mmol) in Et_2O (1 mL) to a stirred suspension of Li (1.21 g of a 25% weright suspension in mineral oil, 43.5 mmol) in THF (13 mL) at –35 °C under Ar. The resulting solution was stirred at –35 °C for 2 h. Then, the solution was allowed to settle, and precipitate-free liquor was carefully decanted off. A solution of $ZnBr_2$ (1.96 g, 8.7 mmol) in Et_2O (14.5 mL) was added to a stirred aliquot of (*Z*)-propenyllithium solution (9.3 mL) at –35 °C under Ar. The resulting solution was allowed to warm to 0 °C and stirred at 0 °C for 1 h. Then, ^{n}BuLi (3.62 mL of a 1.6 M solution in hexanes, 5.8 mmol) was added, followed by a solution of (–)-*N*-methylephidrine (1.04 g, 5.8 mmol) in PhMe (35 mL). The resulting solution was stirred at 0 °C for 1 h. Then, pivaldehyde (500 mg, 630 µL, 5.8 mmol) was added. The resulting solution was stirred at 0 °C for 1 h. Then, saturated $NH_4.Cl_{(aq)}$ (30 mL) was added. The layers were separated, extracting the aqueous with Et_2O (3 × 30 mL). The combined organic layers were washed with saturated $NH_4.Cl_{(aq)}$ (40 mL), dried (Na_2SO_4) and evaporated under reduced pressure to give the crude product. Purification by flash

column chromatography on silica with 9:1→5:1 pentane:Et_2O as eluent gave (*R*)-**4l** (283 mg, 38%, 7:93 er by CSP-GC) as a colourless oil, $[\alpha]_D = -2.7$ (c = 1.3 in $CHCl_3$).

(*E*)-3-Methylpent-3-en-2-ol, 4m

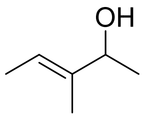

4m

MeLi (19.0 mL of a 1.6 M solution in Et_2O, 30.91 mmol) was added dropwise to a stirred solution of tiglic aldehyde (1.0 g, 1.3 mL, 23.78 mmol) in THF (15 mL) at 0 °C under Ar. The resulting solution was stirred at 0 °C for 30 min the allowed to warm to rt. Then, saturated $NH_4.Cl_{(aq)}$ (10 mL) was added, and the layers were separated, extracting the aqueous with Et_2O (3 × 15 mL). The combined organic layers were dried ($MgSO_4$) and evaporated under reduced pressure to give the crude product. Purification by flash column chromatography on silica with 9:1→5:1 pentane:Et_2O as eluent gave the title product **4m** (1.44 g, 60%) as a colourless oil, R_F 0.1 (9:1 pentane:Et_2O); IR (film) 3344 (OH), 2973, 2920, 1445, 1380, 1366, 1111, 1078, 984, 942, 881, 826, 611 cm^{-1}; ^1H NMR (300 MHz, $CDCl_3$) δ H 5.51-5.41 (1H, m, HC=), 4.18 (1H, q, J = 6.5 Hz, OCH), 1.66-1.55 (6H, m, =CH*Me* + =C*Me*), 1.22 (3H, d, J = 6.5 Hz, OCH*Me*); ^{13}C NMR (75 MHz, $CDCl_3$) δ C 139.3 (=C), 119.3 (=CH), 73.5 (OCH), 21.6 (Me), 13.1 (Me), 11.1 (Me); CSP-GC (Chiraldex-GTA, 60 °C, 35 cm s^{-1}) (*R*)-**4m** 10.50 min, (*S*)-**4m** 11.29 min.

(*R*, *E*)-3-Methylpent-3-en-2-ol (*R*)-4m

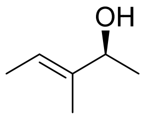

(*R*)-**4m**

Following general procedure **B**, *rac*-**4m** (1.0 g, 9.98 mmol), AmanoAK (300 mg), vinyl acetate (2.32 g, 2.49 mL, 27.00 mmol) and 4 Å molecular sieves (500 mg) in hexane (35 mL) gave the crude product. Purification by flash column chromatography on silica with 9:1→5:1 pentane:Et_2O as eluent gave (*R*)-**4m** (258 mg, 26%, 99:1 er by CSP-GC) as a colourless oil, $[\alpha]_D = -10.8$ (c = 1.11 in $CHCl_3$).

(*E*)-1-Cyclohexyl-2-methylbut-2-en-1-ol, 4n

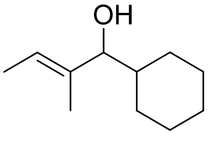

4n

Li (1.32 g of a 25% weight dispersion in mineral oil, 47.56 mmol) was added to THF (12 mL) at rt under Ar. The resulting suspension was vigerously stirred at rt for 30 min. The resulting suspension was cooled to 0 °C and a solution of bromocyclohexane (1.93 g, 1.42 mL, 11.89 mmol) and tiglic aldehyde (1.0 g, 11.89 mmol) in THF (8 mL) was added. The resulting solution was stirred at 0 °C for 30 min. The resulting solution was allowed to warm to rt and saturated $NaHCO_3$ (30 mL) was added. The layers were separated, extracting the aqueous with Et_2O (3 × 40 mL). The combined organic layers were dried ($MgSO_4$) and evaporated under reduced pressure to give the crude product. Purification by flash column chromatography on silica with 9:1→5:1 pentane:Et_2O as eluent gave **4n** (1.19 g, 60%) as a colourless oil, R_F 0.3 (9:1 pentane:Et_2O); IR (film) 3363 (OH), 2919, 2850, 1448, 1379, 1306, 1290, 1260, 1080, 1069, 1003, 971, 891, 846, 828, 796, 687, 612, 584 cm^{-1}; ^1H NMR (300 MHz, $CDCl_3$) δ H 5.40 (1H, q, J = 6.6 Hz, HC=), 3.60 (1H, dd, J = 8.3, 2.0 Hz, OCH), 2.05-1.92 (1H, m, OCHC*H*), 1.80-1.54 (2H, m, Cy), 1.60 (3H, d, J = 6.6 Hz, *Me*CH=), 1.57 (3H, s, =CMe), 1.46-1.34 (3H, m, Cy), 1.27-1.07 (3H, m, Cy), 1.01-0.74 (2H, m, Cy); ^{13}C NMR (100.6 MHz, $CDCl_3$) δ C 137.3 (C=), 121.9 (HC=), 83.2 (OCH), 41.0 (OCH*C*H), 29.8 (CH_2), 29.4 (CH_2), 26.7 (CH_2), 26.4 (CH_2), 26.2 (CH_2), 13.0 (Me), 11.1 (Me); HRMS (APCI) $C_{11}H_{19}O$ ($[M–H]^+$) requires 167.1430, found 167.1426; CSP-GC (β-Dex, 140 °C, 35 cm s^{-1}) (*S*)-**4n** 12.53 min, (*R*)-**4n** 12.95 min.

(*R*, *E*)-1-Cyclohexyl-2-methylbut-2-en-1-ol, (*R*)-4n

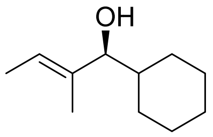

(*R*)-**4n**

Following general procedure **A**, *rac*-**4n** (219 mg, 1.3 mmol), $Ti(O^iPr)_4$ (439 mg, 457 μL, 1.56 mmol), L-DIPT (3.84 mg, 319 μL, 1.56 mmol) and TBHP (130 μL of a 6.0 M solution in dioxane, 0.78 mmol) gave the crude product. Purification by flash column chromatography on silica with 9:1→5:1 pentane:Et_2O as eluent gave (*R*)-**4n** (34 mg, 15%, 92:8 er by CSP-GC) as a colourless oil, $[\alpha]_D$ = –6.3 (c = 1.7 in $CHCl_3$).

2.3) Allylic Alcohol Scope

(*S*,*E*)-Methyl 4-((oct-3-en-2-yloxy)methyl)benzoate 5ab from (*R*,*E*)-4a

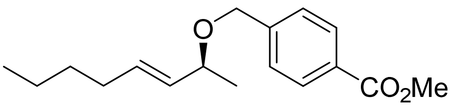

(*R*)-**5ab**

Following general procedure **C**, PPh_3AuNTf_2 (2:1) toluene adduct (2 × 4 mg, 2 × 0.00254 mmol), (*R*,*E*)-Oct-2-en-4-ol (*R*)-**4a** (13 mg, 0.101 mmol, >99:1 er), methyl 4-hydroxymethylbenzoate (84 mg, 0.506 mmol) and 3 Å molecular sieves (8 mg) in PhMe (260 μL) gave the crude product. Purification by flash column chromatography on silica with 49:1→9:1 pentane:Et_2O as eluent gave **5b** (19 mg, 78%, 7:1 *E*:*Z* by ^{1}H NMR, 88:12 er (of *E* alkene) and 16:84 er (of *Z* alkene) by CSP-HPLC of a derivative **S1**, see below) as a colourless oil, R_F 0.2 (9:1 pentane:Et_2O); IR ($CHCl_3$) 2956, 1719 (C=O), 1437, 1408, 1378, 1104, 1018, 875, 822, 731, 667, 617, 605, 589, 576, 563 cm^{-1}; ^{1}H NMR (300 MHz, $CDCl_3$) (7:1 mixture of alkene isomers) δ 8.04-7.96 (2.32H, m, *E* + *Z* Ar), 7.43-7.35 (2.32H, m, *E* + *Z* Ar), 5.61 (1H, dt, *J* = 15.4, 6.8 Hz, *E* CH_2CH=), 5.60-5.51 (0.16H, m, *Z* CH_2CH=), 5.37 (1H, ddt, *J* = 15.4, 7.9, 1.4 Hz, *E* OCHC*H*=), 5.40-5.29 (0.16H, m, *Z* OCHC*H*=), 4.61 (0.16H, d, *J* = 13.0 Hz, *Z* OCH_AH_B), 4.59 (1H, d, *J* = 12.7 Hz, *E* OCH_AH_B), 4.41 (1H, d, *J* = 12.7 Hz, *E* OCH_AH_B), 4.40 (0.16H, d, *J* = 13.0 Hz, *Z* OCH_AH_B), 4.30 (0.16H, dqd, *J* = 9.1, 6.2, 1.1 Hz, *Z* OCH), 3.91 (s, 3.48H, *E* + *Z* OMe), 3.87 (1H, dq, *J* = 7.9, 5.7 Hz, *E* OCH), 2.11-1.96 (2.32H, m, *E* + *Z* =CHCH_2), 1.74 (0.48H, dd, *J* = 6.2, 1.6 Hz, *Z* OCH*Me*), 1.47-1.23 (4.64H, m, *E* + *Z* MeCH_2 + $MeCH_2CH_2$), 1.28 (3H, d, *J* = 5.7 Hz, *E* OCH*Me*), 0.96-0.83 (3.48H, m, *E* + *Z* CH_2*Me*); ^{13}C NMR (75 MHz, $CDCl_3$) (*Z* alkene peaks not observed) δ C 167.2 (C=O), 144.6 (*ipso*-Ar), 133.8 (=CH), 131.7 (=CH), 129.8 (Ar), 129.2 (*ipso*-Ar), 127.3 (Ar), 76.5 (OCH), 69.2 (OCH_2), 52.2 (OMe), 32.0 (CH_2), 31.5 (CH_2), 22.4 (CH_2), 21.9 (OCH*Me*), 14.1 (CH_2*Me*); HRMS (NSI) $C_{17}H_{25}O_3$ ([M + H]$^+$) requires 277.1798, found 277.1798.

(*R*,*E*)-Methyl 4-((oct-3-en-2-yloxy)methyl)benzoate 5bb from (*R*, *Z*)-4b

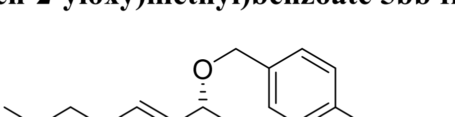

(*S*)-**5bb**

Following general procedure **C**, PPh_3AuNTf_2 (2:1) toluene adduct (2 × 4 mg, 2 × 0.00254 mmol), (*R*,*Z*)-oct-2-en-4-ol (*R*)-**4b** (13 mg, 0.101 mmol, 81:19 er), methyl 4-hydroxymethylbenzoate (84 mg, 0.506 mmol) and 3 Å molecular sieves (8 mg) in PhMe (260 μL) gave the crude product. Purification by flash column chromatography on silica with 49:1→9:1 pentane:Et_2O as eluent gave **5bb** (20 mg, 71% and 11:1 *E*:*Z* by ^{1}H NMR, 24:76 er (of *E* alkene) and 84:16 er (of *Z* alkene) by CSP-HPLC of a derivative **S2**, see below) as a colourless oil.

(*S*,*E*)-(4-((Oct-3-en-2-yloxy)methyl)phenyl)methanol (S1)

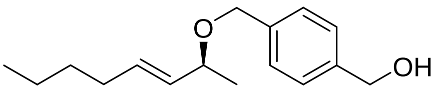

$LiAlH_4$ (13 mg, 0.344 mmol) was added in one portion to a stirred solution of (*S*,*E*)-methyl 4-((oct-3-en-2-yloxy)methyl)benzoate **5ab** (19 mg, 0.069 mmol, 7:1 *E*:*Z*) in THF (1.5 mL) at rt under Ar. The resulting solution was stirred under reflux under Ar for 16 h. Then, the resulting solution was allowed to cool to rt and 1 M $NaOH_{(aq)}$ was added dropwise until a white precipitate formed. The solids were filtered off over Celite®, washing with Et_2O. The filtrate was evaporated under reduced pressure to give the crude product. Purification by flash column chromatography on silica with 4:1→2:1 pentane:Et_2O as eluent gave (*S*,*E*)-(4-((oct-3-en-2-yloxy)methyl)phenyl)methanol (12 mg, 71%, 7:1 *E*:*Z*, 88:12 er (of *E* alkene) and 16:84 er (of *Z* alkene) by CSP-HPLC) as a colourless oil, R_F 0.1 (4:1 pentane:Et_2O); IR ($CHCl_3$) 3372 (OH), 2957, 2927, 2859, 1455, 1421, 1369, 1214, 1090, 1064, 1017, 971, 914, 806, 754, 666, 612, 586, 565 cm^{-1}; ^{1}H NMR (300 MHz, $CDCl_3$) δ H 7.33 (4.4H, s, Ar), 5.61 (1H, dt, *J* = 15.2, 6.7 Hz, *E* CH_2CH=), 5.59-5.50 (0.1H, m, *Z* CH_2CH=), 5.37 (1H, ddt, *J* = 15.2, 7.9, 1.4 Hz, *E* OCHC*H*=), 5.38-5.29 (0.1H, m, *Z* OCHCH_2), 4.68 (2.2H, br s, *E* + *Z* HOCH_2), 4.56 (0.1H, d, *J* = 12.0 Hz, *Z* CHOCH_AH_B), 4.55 (1H, d, *J* = 12.0 Hz, *E*CHOCH_AH_B), 4.36 (1H, d, *J* = 12.0 Hz, *E* CHOCH_AH_B), 4.35 (0.1H, d, *J* = 12.0 Hz, *Z* CHOCH_ACH_B), 3.86 (1H, dq, *J* = 7.9, 6.4 Hz, *E* OCH), 3.70-3.60 (0.1H, m, *Z* OCH), 2.12-2.00 (m, 2.2H, *E* + *Z* =CHCH_2), 1.74 (0.3H, dd, *J* = 6.4, 1.6 Hz, *Z* OCH*Me*), 1.61 (1.1H, br s, *E* + *Z* OH), 1.40-1.26 (4.4H, m, *E* + *Z* MeCH_2 + MeCH_2CH_2), 1.27 (3H, d, *J* = 6.4 Hz, *E*OCH*Me*), 0.89-0.85 (3.3H, m, *E* + *Z* CH_2*Me*); ^{13}C NMR (75 MHz, $CDCl_3$) (*Z* alkene not observed) δ 140.1 (*ipso*-Ar), 138.7 (*ipso*-Ar), 133.6 (CH), 131.9 (CH), 128.1 (CH), 127.2 (CH), 76.0 (OCH), 69.4 (OCH_2), 65.4 (OCH_2), 32.0 (CH_2), 31.6 (CH_2), 22.4 (CH_2), 21.9 (Me), 14.1 (Me); HRMS (APCI) $C_{16}H_{28}NO_2$ ($[M+NH_4]^+$) requires 266.2115, found 266.2110; CSP-HPLC (Chiralcel OD-H, 99:1 hexane:IPA, 0.5 mL min^{-1}) (*R*, *Z*)-**S1** 27.23 min, (*S*, *Z*)-**S1** 28.99 min, (*S*, *E*)-**S1** 30.28 min, (*R*, *E*)-**S1** 33.27 min.

(*R*,*E*)-(4-((Oct-3-en-2-yloxy)methyl)phenyl)methanol (S2)

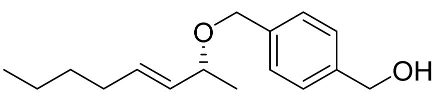

$LiAlH_4$ (14 mg, 0.34 mmol) was added in one portion to a stirred solution of (*R*,*E*)-methyl 4-((oct-3-en-2-yloxy)methyl)benzoate (*R*)-**5bb** (20 mg, 0.071 mmol, 11:1 *E*:*Z*) in THF (1.5 mL) at rt under Ar. The resulting solution was stirred under reflux under Ar for 16 h. Then, the resulting solution was allowed to cool to rt and 1 M $NaOH_{(aq)}$ was added dropwise until a white precipitate formed. The solids were filtered off over Celite®, washing with Et_2O. The filtrate was evaporated under reduced pressure to give the crude product. Purification by flash column chromatography on silica with 4:1→2:1 pentane:Et_2O as eluent gave (*R*,*E*)-(4-((oct-

3-en-2-yloxy)methyl)phenyl)methanol **S2** (12 mg, 67%, 11:1 *E*:*Z*, 24:76 er (of *E* alkene) and 84:16 er (of *Z* alkene) by CSP-HPLC) as a colourless oil.

(*S*, *E*)-Methyl 4-((oct-2-en-4-yloxy)methyl)benzoate (*S*)-5cb from (*R*, *E*)-4c

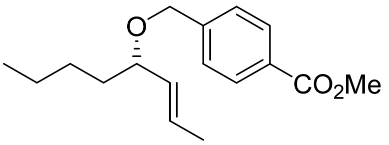

(*S*)-**5cb**

Following general procedure **C**, PPh_3AuNTf_2 (2:1) toluene adduct (2 × 4 mg, 2 × 0.00254 mmol), (*R*,*E*)-Oct-3-en-2-ol (*R*)-**4c** (13 mg, 0.101 mmol, >99:1 er), methyl 4-hydroxymethylbenzoate (84 mg, 0.506 mmol) and 3 Å molecular sieves (8 mg) in PhMe (260 μL) gave the crude product. Purification by flash column chromatography on silica with 49:1→9:1 pentane:Et_2O as eluent gave (*S*)-**5cb** (22 mg, 78% and 5:1 *E*:*Z* by ^{1}H NMR, 98:2 er (of *E* alkene) and 16:84 er (of *Z* alkene) by CSP-HPLC of a derivative) as a colourless oil, R_F 0.2 (9:1 pentane:Et_2O); IR ($CHCl_3$) 2926, 1721 (C=O), 1436, 1378, 1274, 1215, 1104, 1019, 731, 667 cm^{-1}; ^{1}H NMR (300 MHz, $CDCl_3$) (5:1 mixture of alkene isomers) δ H 8.04-7.95 (2.4H, m, *E* + *Z* Ar), 7.43-7.35 (2.4H, m, *E* + *Z* Ar), 5.71 (0.2H, dqd, *J* = 11.0, 6.9, 0.9 Hz, *Z* MeC*H*=), 5.62 (1H, dqd, *J* = 15.1, 6.4, 0.5 Hz, *E* MeC*H*=), 5.42-5.27 (0.2H, m, *Z* OCHC*H*=), 5.35 (1H, ddq, *J* = 15.1, 8.2, 1.6 Hz, *E* OCHC*H*=), 4.61 (1H, d, *J* = 12.9 Hz, *E* OCH_AH_B), 4.59 (0.2H, d, *J* = 13.0 Hz, *Z* OCH_AH_B), 4.39 (0.2H, d, *J* = 13.0 Hz, *Z* OCH_AH_B), 4.38 (1H, d, *J* = 12.9 Hz, *E* OCH_AH_B), 4.14 (0.2H, dtd, *J* = 9.4, 6.3, 0.8 Hz, *Z* OCH), 3.91 (3.6H, br s, *E* + *Z* OMe), 3.65 (1H, dt, *J* = 8.2, 6.5 Hz, *E* OCH), 1.74 (3H, dd, *J* = 6.4, 1.6 Hz, *E* =CH*Me*), 1.70-1.61 (1H, m, *E* $=CHCH_AH_B$), 1.62 (0.6H, dd, *J* = 6.9, 1.7 Hz, *Z* =CH*Me*), 1.55-1.22 (6.2H, m, *Z* $=CHCH_AH_B$ + *E* + *Z* $=CHCH_AH_B$ + $MeCH_2$ + $MeCH_2CH_2$), 0.94-0.84 (3.6H, m, *E* + *Z* CH_2Me); ^{13}C NMR (75 MHz, $CDCl_3$) (mixture of alkene isomers) δ C 167.2 (*E* + *Z* C=O), 144.7 (*E* *ipso*-Ar), 144.6 (*Z* *ipso*-Ar), 132.1 (*E* =CH), 131.8 (*Z* =CH), 131.7 (*E* + *Z* *ipso*-Ar), 129.8 (Z Ar), 129.7 (*E* Ar), 129.2 (Z =CH), 129.1 (*E* =CH), 127.4 (Z Ar), 127.3 (*E* Ar), 80.8 (*E* OCH), 74.2 (*Z* OCH), 69.3 (*Z* OCH_2), 69.2 (*E* OCH_2), 52.2 (*E* + *Z* OMe), 35.6 (*E* $=CHCH_2$), 35.4 (*Z* $=CHCH_2$), 27.8 (*E* CH_2), 27.7 (*Z* CH_2), 22.9 (*E* CH_2), 22.8 (*Z* CH_2), 22.4 (*Z* =CH*Me*), 17.9 (*E* =CH*Me*), 14.2 (*E* CH_2Me), 14.1 (*Z* CH_2Me); HRMS (NSI) $C_{17}H_{25}O_3$ ($[M + H]^+$) requires 277.1798, found 277.1797.

(*S*,*E*)-(4-((Oct-2-en-4-yloxy)methyl)phenyl)methanol (S3)

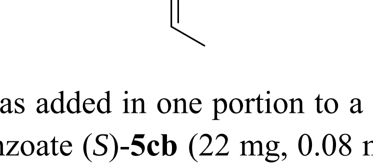

$LiAlH_4$ (15 mg, 0.398 mmol) was added in one portion to a stirred solution of (*S*, *E*)-methyl 4-((oct-2-en-4-yloxy)methyl)benzoate (*S*)-**5cb** (22 mg, 0.08 mmol, 5:1 *E*:*Z*) in THF (1.5 mL) at rt under Ar. The resulting solution was stirred under reflux under Ar for 16 h. Then, the

resulting solution was allowed to cool to rt and 1 M $NaOH_{(aq)}$ was added dropwise until a white precipitate formed. The solids were filtered off over Celite®, washing with Et_2O. The filtrate was evaporated under reduced pressure to give the crude product. Purification by flash column chromatography on silica with 4:1→2:1 pentane:Et_2O as eluent gave (*S*,*E*)-(4-((oct-2-en-4-yloxy)methyl)phenyl)methanol (15 mg, 75%, 5:1 *E*:*Z*, 98:2 er (of *E* alkene) and 16:84 er (of *Z* alkene) by CSP-HPLC) as a colourless oil, R_F 0.1 (4:1 pentane:Et_2O); IR ($CHCl_3$) 3381 (OH), 2930, 2859, 1454, 1421, 1378, 1215, 1054, 1017, 969, 806, 756, 559 cm^{-1}; ^1H NMR (300 MHz, $CDCl_3$) (5:1 mixture of alkene isomers) δ 7.33 (4.8H, br s, *E* + *Z* Ar), 5.71 (0.2H, dqd, *J* = 11.1, 7.0, 0.8 Hz, *Z* MeC*H*=), 5.61 (1H, dq, *J* = 15.3, 6.3 Hz, *E* MeC*H*=), 5.42-5.33 (0.2H, m, *Z* OCHC*H*=), 5.35 (1H, ddq, *J* = 15.3, 8.2, 1.6 Hz, *E* OCHC*H*=), 4.72-4.64 (2.4H, m, *E* + *Z* OHCH_2), 4.56 (1H, d, *J* = 12.0 Hz, *E* OCH_AH_B), 4.55 (0.2H, d, *J* = 12.1 Hz, *Z* OCH_AH_B), 4.33 (0.2H, d, *J* = 12.1 Hz, *Z* OCH_AH_B), 4.32 (1H, d, *J* = 12.0 Hz, *E* OCH_AH_B), 3.91-3.82 (0.2H, m, *Z* OCH), 3.65 (1H, ddd, *J* = 8.2, 8.2, 8.0 Hz, *E* OCH), 1.74 (3H, dd, *J* = 6.3, 1.6 Hz, *E* =CH*Me*), 1.65-1.58 (1.6H, m, *E* OCHCH_AH_B + *Z* =CH*Me*), 1.39-1.23 (6.2H, m, *Z* OCHCH_AH_B + *E* + *Z* CH_2), 0.93-0.82 (3.6H, m, *E* + *Z* CH_2*Me*); ^{13}C NMR (75 MHz, $CDCl_3$) (*Z* alkene peaks not observed) δ C 140.0 (*ipso*-Ar), 138.8 (*ipso*-Ar), 132.4 (=CH), 128.8 (=CH), 128.1 (Ar), 127.1 (Ar), 80.3 (OCH), 69.5 (OCH_2), 65.4 (OCH_2), 35.6 (CH_2), 27.8 (CH_2), 22.8 (CH_2), 17.9 (=CH*Me*), 14.2 (CH_2*Me*); HRMS (NSI) $C_{16}H_{28}O_2N$ ($[M+NH_4]^+$) requires 266.2115, found 266.2112; CSP-HPLC (Chiralcel OD-H, 99.3:0.7 hexane:IPA, 1 mL min^{-1}) (*S*, *E*)-**S3** 27.26 min, (*R*, *E*)-**S3** 30.59 min, (*S*, *Z*)-**S3** 33.90 min, (*R*, *Z*)-**S3** 36.75 min.

(*S*, *E*)-Methyl 4-((4-cyclohexylbut-3-en-2-yloxy)methyl)benzoate (*S*)-5db

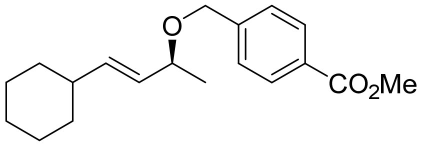

(*S*)-**5db**

Following general procedure **C**, PPh_3AuNTf_2 (2:1) toluene adduct (2 × 4 mg, 2 × 0.00254 mmol), (*R*, *E*)-1-cyclohexylbut-2-en-1-ol (*R*)-**4d** (16mg, 0.101 mmol, >99:1 er), methyl 4-hydroxymethylbenzoate (84 mg, 0.506 mmol) and 3 Å molecular sieves (8 mg) in PhMe (260 μL) gave the crude product. Purification by flash column chromatography on silica with 49:1→9:1 hexane:Et_2O as eluent gave (*S*)-**5db** (24 mg, 77% and 10:1 *E*:*Z* by ^1H NMR, 89:11 er (of *E* alkene) and 46:54 er (of *Z* alkene) by CSP-HPLC) as a colourless oil, R_F 0.2 (9:1 hexane:Et_2O); IR ($CHCl_3$) 2923, 2851, 1722 (C=O), 1435, 1275, 1192, 1174, 1105, 1074, 1019, 970, 847, 754, 704 cm^{-1}; ^1H (300 MHz, $CDCl_3$) (10:1 mixture of *E*:*Z* alkene isomers) δ 8.06-7.95 (2.2H, m, Ar), 7.44-7.34 (2.2H, m, Ar), 5.62-5.50 (0.1H, m, *Z* CyC*H*=), 5.56 (1H, dd, *J* = 15.6, 6.6 Hz, *E* CyC*H*=), 5.38-5.25 (0.1H, m, *Z* OCHC*H*=), 5.31 (1H, ddd, *J* = 15.6, 7.9, 1.3 Hz, *E* OCHC*H*=), 4.61 (0.1H, d, *J* = 13.0 Hz, *Z* OCH_AH_B), 4.58 (1H, d, *J* = 13.0 Hz, *E* OCH_AH_B), 4.40 (1H, d, *J* = 13.0 Hz, *E* $OCH_AC H_B$), 4.34 (0.1H, d, *J* = 13.0 Hz, *Z* $OCH_AC H_B$), 3.91 (3.3H, s, *E* + *Z* OMe), 3.85 (1H, dq, *J* = 7.9, 6.3 Hz, *E* OCH), 3.40-3.33 (0.1H, m, *Z* OCH), 2.05-1.89 (1.1H, m, *E* + *Z* =CHC*H*), 1.81-1.59 (5.5H, m, *E* + *Z* Cy + *Z* OCH*Me*), 1.38-0.98 (6.6H, m, *E* + *Z* Cy), 1.28 (3.3H, d, *J* = 6.3 Hz, *E* OCH*Me*); ^{13}C NMR

(75 MHz, $CDCl_3$) (*Z* alkene not observed) δ 167.2 (C=O), 144.7 (*ipso*-Ar), 139.7 (CH), 129.8 (CH), 129.2 (*ipso*-Ar), 129.0 (CH), 127.4 (CH), 76.7 (OCH), 69.1 (OCH_2), 52.2 (OMe), 40.4 (=CH*C*H), 33.0 (CH_2), 31.7 (CH_2), 26.1 (CH_2), 22.8 (CH_2), 22.0 (CH_2), 14.3 (OCH*Me*); HRMS (NSI) $C_{19}H_{27}O_3$ ($[M+H]^+$) requires 303.1955, found 303.1956; CSP-HPLC (Chiralpak IB, 99.9:0.1 hexane:IPA, 1 mL min^{-1}) (*S*, *Z*)-**5db** 7.62 min, (*R*, *Z*)-**5db** 8.01 min, (*S*, *E*)-**5db** (8.24 min, (*R*, *E*)-**5db** 9.91 min.

(*E*)-Methyl 4-((4-cyclohexylbut-3-en-2-yloxy)methyl)benzoate *Rac*-5db (Scheme 4)

A solution of PPh_3AuNTf_2 (2:1) toluene adduct (8 g, 0.00508 mol), (*R*, *E*)-1-cyclohexylbut-2-en-1-ol (*R*)-**4d** (16mg, 0.101 mmol, >99:1 er) and methyl 4-hydroxymethylbenzoate (84 mg, 0.506 mmol) in PhMe (260 μL) was stirred at 50 °C under air for 24 h. The resulting solution was filtered over a short plug of silica, washing with 9:1 hexane:Et_2O. The filtrate was evaporated under reduced pressure to give the crude product. Purification by flash coumn chromatography on silica with 49:1→9:1 pentane:Et_2O as eluent gave (*E*)-methyl 4-((4-cyclohexylbut-3-en-2-yloxy)methyl)benzoate *rac*-**5db** (16 mg, 51%, >20:1 S_N2':S_N2, 3:1 *E*:*Z*, racemic) as a colourless oil.

(*E*)-Methyl 4-((4-cyclohexylbut-3-en-2-yloxy)methyl)benzoate *Rac*-5db (Scheme 7, Eq. 1)

To a solution of (*R*, *E*)-1-cyclohexylbut-2-en-1-ol (*R*)-**4d** (16mg, 0.101 mmol, >99:1 er) and methyl 4-hydroxymethylbenzoate (67 mg, 0.404 mmol) in CH_2Cl_2 (168 μL) was added (IPr)AuCl (3 mg, 0.00505 mmol) followed by $AgClO_4$ (1 mg, 0.00505 mml). The resulting solution was stirred at 40 °C under air for 24 h. The resulting solution was filtered over a short silica plug, washing with 9:1 hexane:Et_2O. The filtrate was evaporated under reduced pressure to give the crude product. Purificatin by flash column chromatography on silica with 49:1→9:1 pentane:Et_2O as eluent gave (*E*)-methyl 4-((4-cyclohexylbut-3-en-2-yloxy)methyl)benzoate *rac*-**5db** (25 mg, 81%, >20:1 S_N2':S_N2, 3:1 *E*:*Z*, racemic) as a colourless oil.

Resubjection of (*S*, *E*)-Methyl 4-((4-cyclohexylbut-3-en-2-yloxy)methyl)benzoate (*S*)-5db (Scheme 3, Eq 1)

PPh_3AuNTf_2 (2:1) toluene adduct (6 mg, 0.008 mmol) was added to (*S*, *E*)-Methyl 4-((4-cyclohexylbut-3-en-2-yloxy)methyl)benzoate (*S*)-**5db** (24 mg, 0.079 mmol, 89:11 er (of *E* alkene), 3 Å molecular sieves (6 mg) and methyl 4-hydroxymethylbenzoate (53 mg, 0.32 mmol) in PhMe (203 μL). The resulting solution was stirred at 50 °C for 24 h. The resulting solution was filtered over a plug of silica, eluting with 9:1 pentane:Et_2O. The filtrate was evaporated under reduced pressure to give the crude product. Purification by flash column chromatography on silica with 49:1→9:1 hexane:Et_2O as eluent gave benzoate (*S*)-**5db** (16 mg, 67% and 10:1 *E*:*Z* by ^{1}H NMR, ~90:10 er (of *E* alkene) by CSP-HPLC) as a colourless oil.

(*S*, *E*)-Methyl 4-((1-cyclohexylbut-2-enyloxy)methyl)benzoate (*S*)-5eb

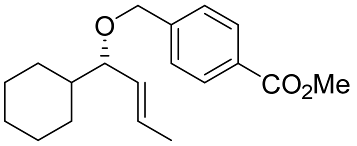

(*S*)-**5eb**

Following general procedure **C**, PPh_3AuNTf_2 (2 × 4 mg, 2 × 0.00254 mmol), (*R*, *E*)-4-cyclohexylbut-3-en-2-ol (*R*)-**4e** (16 mg, 0.101 mmol, >99:1 er), methyl 4-hydroxymethylbenzoate (84 mg, 0.506 mmol) and 3 Å molecular sieves (8 mg) in PhMe (260 μL) gave the crude product. Purification by flash column chromatography on silica with 49:1→9:1 pentane:Et_2O as eluent gave (*S*)-**5eb** (24 mg, 80%, 9:1 *E*:*Z* by ^{1}H NMR, 77:23 er (of *E* alkene) and 53:47 er (of *Z* alkene) by CSP-HPLC) as a colourless oil, R_F 0.4 (9:1 pentane:Et_2O); IR (film) 2922, 2851, 1722 (C=O), 1434, 1274, 1191, 1174, 1104, 1019, 969, 890, 838, 754, 732, 704 cm^{-1}; ^{1}H NMR (300 MHz, $CDCl_3$) (6:1 *E*:*Z*) δ H 8.04-7.94 (2.24H, m, *E* + *Z* Ar), 7.43-7.35 (2.24H, m, *E* + *Z* Ar), 5.60-5.50 (0.12H, m, *Z* MeC*H*=), 5.57 (1H, dqd, *J* = 15.3, 6.4, 0.4 Hz, *E* MeC*H*=), 5.35-5.23 (0.12H, m, *Z* OCHC*H*=), 5.34 (1H, ddq, *J* = 15.3, 8.6, 1.5 Hz, *E* OCHC*H*=), 4.61 (1H, d, *J* = 12.9 Hz, *E* OC$H_A$$H_B$), 4.58 (0.12H, d, *J* = 12.7 Hz, *Z* OC$H_A$$H_B$), 4.40 (0.12H, d, *J* = 12.7 Hz, *Z* OC$H_A$$H_B$), 4.34 (1H, d, *J* = 12.9 Hz, *E* OC$H_A$$H_B$), 4.22 (0.12H, dd, *J* = 5.7, 3.5 Hz, *Z* OCH), 3.91 (3.36H, br s, *E* + *Z* OMe), 3.36 (1H, dd, *J* = 8.6, 7.3 Hz, *E* OCH), 2.03-1.90 (1.12H, m, *E* + *Z* OCHC*H*), 1.81 (0.36H, dd, *J* = 6.3, 1.4 Hz, *Z* =CH*Me*),1.76-1.58 (4.8H, m, *E* + *Z* Cy), 1.74 (3H, dd, *J* = 6.4, 1.5 Hz, *E* =CH*Me*), 1.32-1.12 (4.8H, m, *E* + *Z* Cy), 1.03-0.83 (2.24H, m, *E* + *Z* Cy); ^{13}C NMR (75 MHz, $CDCl_3$) (*Z* isomer not observed) δ C 167.2 (C=O), 144.9 (*ipso*-Ar), 130.6 (CH), 129.9 (CH), 129.8 (CH), 129.7 (CH), 129.1 (*ipso*-Ar), 127.4 (CH), 85.4 (OCH), 69.3 (OCH_2), 52.2 (OMe), 42.7 (OCH*C*H), 29.5 (CH_2), 29.2 (CH_2), 26.8 (CH_2), 26.3 (CH_2), 26.2 (CH_2), 17.9 (Me); HRMS (APCI) $C_{19}H_{27}O_3$ ($[M+H]^+$) requires 303.1955, found 303.1956; CSP-HPLC (Chiralpak IA, 99.5:0.5 hexane:IPA, 0.5 mL min^{-1}) (*S*, *E*)-**5eb** 12.07 min, (*R*, *E*)-**5eb** 13.11 min, *Z* alkene peaks at 17.17min and 18.52 min.

Resubjection of (*S*, *E*)-Methyl 4-((1-cyclohexylbut-2-enyloxy)methyl)benzoate (*S*)-5eb (Scheme 3, Eq. 2)

PPh_3AuNTf_2 (2:1) toluene adduct (6 mg, 0.008 mmol) was added to (*S*, *E*)-Methyl 4-((1-cyclohexylbut-2-enyloxy)methyl)benzoate (*S*)-**5eb** (24 mg, 0.079 mmol, 77:23 er (of *E* alkene) and 53:47 er (of *Z* alkene)), 3 Å molecular sieves (6 mg) and methyl 4-hydroxymethylbenzoate (53 mg, 0.32 mmol) in PhMe (203 μL). The resulting solution was stirred at 50 °C for 24 h. The resulting solution was filtered over a plug of silica, eluting with 9:1 pentane:Et_2O. The filtrate was evaporated under reduced pressure to give the crude product. Purification by flash column chromatography on silica with 49:1→9:1 hexane:Et_2O as eluent gave benzoate (*S*)-**5eb** (15 mg, 62%, >20:1 and 9:1 *E*:*Z* by ^{1}H NMR, 78:22 er (of *E* alkene) and 54:46 er (of *Z* alkene) by CSP-HPLC) as a colourless oil.

(*R*, *E*)-Methyl 4-((2-methylhex-4-en-3-yloxy)methyl)benzoate (*R*)-5fb

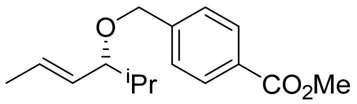

(*R*)-**5fb**

Following general procedure **C**, PPh_3AuNTf_2 (2:1) toluene adduct (2 × 4 mg, 2 × 0.00254 mmol), (*R*, *E*)-5-methylhex-3-en-2-ol (*R*)-**4f** (11 mg, 0.101 mmol, >99:1 er), methyl 4-hydroxymethylbenzoate (84 mg, 0.506 mmol) and 3 Å molecular sieves (8 mg) in PhMe (260 μL) gave the crude product. Purification by flash column chromatography on silica with 49:1→9:1 pentane:Et_2O as eluent gave benzoate (*R*)-**5fb** (21 mg, 81% and 5:1 *E*:*Z* by ^{1}H NMR, 23:77 er (of *E* alkene) and 87:13 er (of *Z* alkene) by CSP-HPLC) as a colourless oil, R_F 0.4 (9:1 pentane:Et_2O); IR ($CHCl_3$) 2957, 1723 (C=O), 1436, 1384, 1276, 1193, 1175, 1105, 1019, 970, 757, 732 cm^{-1}; ^{1}H NMR (300 MHz, $CDCl_3$) (5:1 mixture of *E*:*Z* alkenes) δ 8.04-7.96 (2.4H, m, Ar), 7.44-7.35 (2.4H, m, Ar), 5.78 (0.2H, qd, *J* = 7.0, 0.9 Hz, *Z* MeC*H*=), 5.60 (dqd, *J* = 15.3, 6.4, 0.5 Hz, *E* MeC*H*=), 5.35 (1H, ddq, *J* = 15.3, 8.6, 1.5 Hz, *E* OCHC*H*=), 5.22-5.14 (0.2H, m, *Z* OCHC*H*=), 4.67-4.55 (0.2H, m, *Z* OCH_AH_B), 4.61 (1H, d, *J* = 12.9 Hz, *E* OCH_AH_B), 4.42-4.34 (0.2H, m, *Z* OCH_AH_B), 4.36 (2H, d, *J* = 12.9 Hz, *E* OCH_AH_B), 3.91 (3.6H, s, *E* + *Z* OMe), 3.71 (0.2H, dd, *J* = 9.5, 2.4 Hz, *Z* OCH), 3.35 (1H, dd, *J* = 8.6, 6.7 Hz, *E* OCH), 1.88-1.70 (1.2H, m, *E* + *Z* $CHMe_2$), 1.75 (3H, dd, *J* = 6.4, 1.5 Hz, *E* *Me*CH=), 1.61 (0.6H, dd, *J* = 7.0, 0.2 Hz, *Z* *Me*CH=), 0.99-0.93 (3.6H, m, *E* + *Z* $CHMe_AMe_B$), 0.90-0.83 (3.6H, m, *E* + *Z* $CHMe_AMe_B$); ^{13}C NMR (75 MHz, $CDCl_3$) (*Z* alkene unobserved) δ 167.2 (C=O), 144.9 (*ipso*-Ar), 130.2 (CH), 130.1 (CH), 129.7 (*ipso*-Ar), 129.1 (CH), 127.3 (CH), 86.1 (OCH), 69.3 (OCH_2), 52.2 (OMe), 33.0 ($CHMe_2$), 19.0 (Me), 18.6 (Me), 17.9 (Me); HRMS (NSI) $C_{16}H_{23}O_3$ ($[M+H]^+$) requires 263.1642, found 263.1642; CSP-HPLC (Chiralpak IB, 99.9:0.1 hexane:IPA, 0.5 mL min^{-1}) (*R*, *Z*)-**5fb** 14.75 min, (*S*, *E*)-**5fb** 15.68 min, (*R*, *E*)-**5fb** 16.58 min, (*S*, *Z*)-**5fb** 18.34 min.

(*S*, *E*)-Methyl 4-((5-phenylpent-3-en-2-yloxy)methyl)benzoate (*S*)-5gb

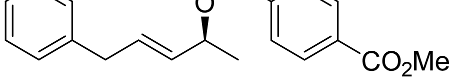

(*S*)-**5gb**

Following general procedure **C**, PPh_3AuNTf_2 (2:1) toluene adduct (2 × 4 mg, 2 × 0.00254 mmol), (*R*, *E*)-2-phenylpent-3-en-2-ol (*R*)-**4g** (16 mg, 0.101 mmol, >99:1 er), methyl 4-hydroxymethylbenzoate (84 mg, 0.506 mmol) and 3 Å molecular sieves (8 mg) in PhMe (260 μL) gave the crude product. Purification by flash column chromatography on silica with 49:1→9:1 pentane:Et_2O as eluent gave benzoate (*S*)-**5gb** (23 mg, 74%, >20:1 *E*:*Z* by ^{1}H NMR, 99:1 er by CSP-HPLC) as a colourless oil, R_F 0.2 (9:1 pentane:Et_2O); $[\alpha]_D$ = –27.9 (*c* = 0.215 in $CHCl_3$); IR (film) 2974, 1719 (C=O), 1613, 1494, 1453, 1434, 1415, 1370, 1274, 1191, 1174, 1145, 1104, 1082, 1019, 970, 912, 847, 800, 753, 698, 635, 593 cm^{-1}; ^{1}H NMR (300 MHz, $CDCl_3$) δ H 8.03-7.96 (2H, m, Ar), 7.41-7.15 (7H, m, Ar), 5.79 (1H, dt, *J* = 15.4, 6.9 Hz, =C*H*CH_2), 5.49 (1H, ddt, *J* = 15.4, 7.9, 1.4 Hz, OCHC*H*=), 4.61 (1H, d, *J* = 12.8 Hz, OCH_AH_B), 4.43 (1H, d, *J* = 12.8 Hz, OCH_AH_B), 3.99-3.87 (1H, m, OCH), 3.91 (3H, s, OMe),

3.40 (2H, d, J = 6.9 Hz, =CHCH_2), 1.31 (3H, d, J = 6.3 Hz, OCH*Me*); ^{13}C NMR (75 MHz, $CDCl_3$) δ C 167.1 (C=O), 144.5 (*ipso*-Ar), 140.2 (*ipso*-Ar), 133.3 (=CH), 132.0 (=CH), 129.8 (Ar), 129.2 (*ipso*-Ar), 128.6 (Ar), 128.6 (Ar), 127.3 (Ar), 126.3 (Ar), 76.3 (OCH), 69.4 (OCH_2), 52.2 (OMe), 38.8 (=CHCH_2), 21.8 (Me); HRMS (APCI) $C_{20}H_{26}NO_3$ ($[M+NH_4]^+$) requires 328.1907, found 382.1907; CSP-HPLC (Chiralpak IA, 99:1 hexane:IPA, 0.5 mL min^{-1}) (*R*, *E*)-**5gb** 12.72 min, (*S*, *E*)-**5gb** 13.78 min.

Methyl (E)-4-(((4-phenylbut-3-en-2-yl)oxy)methyl)benzoate 5hb from **(*R*, *E*)-1-phenylbut-2-en-1-ol (*R*)-4h**

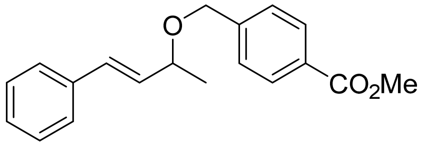

5hb

Following general procedure **C**, PPh_3AuNTf_2 (2:1) toluene adduct (2 × 4 mg, 2 × 0.00254 mmol), (*R*, *E*)-1-phenylbut-2-en-1-ol (*R*)-**4h** (15 mg, 0.101 mmol, 91:9 er), methyl 4-hydroxymethylbenzoate (84 g, 0.506 mmol) and 3 Å molecular sieves (8 mg) in PhMe (260 µL) gave the crude product. Purification by flash column chromatography on silica with 49:1→9:1 pentane:Et_2O gave benzoate **5hb** (20 mg, 67%, 12:1 *E*:*Z* by ^{1}H NMR, 49:51 er by CSP-HPLC) as a colourless oil, R_F 0.3 (9:1 pentane:Et_2O); IR ($CHCl_3$) 3025, 1717 (C=O), 1614, 1494, 1436, 1278, 1216, 1193, 1175, 1145, 1107, 1078, 1019, 968, 929, 840, 745, 692, 667 cm^{-1}; ^{1}H NMR (400 MHz, $CDCl_3$) (12:1 mixture of *E*:*Z* alkenes) δ H 8.03-7.96 (2.16 H, m, *E* + *Z* Ar), 7.45-7.17 (7.56 H, m, *E* + *Z* Ar), 6.57-6.49 (0.08H, m, *Z* ArC*H*=), 6.52 (1H, d, J = 16.0 Hz, *E* ArC*H*=), 6.19-6.10 (0.08H, m, *Z* CH=), 6.14 (1H, dd, J = 16.0, 8.1 Hz, *E* CH=), 4.14-4.04 (1.08H, m, *E* + *Z* OCH), 3.90 (3.24H, br s, *E* + *Z* OMe), 1.71 (0.24H, br d, J = 5.7 Hz, *Z* CH*Me*), 1.39 (3H, d, J = 6.4 Hz, *E* CH*Me*); ^{13}C NMR(75 MHz, $CDCl_3$) (*Z* alkene peaks not observed) δ 167.2 (C=O), 144.4 (*ipso*-Ar), 136.7 (*ipso*-Ar), 131.9 (CH), 131.5 (CH), 129.9 (CH), 129.4 (*ipso*-Ar), 128.8 (CH), 128.0 (CH), 127.4 (CH), 126.7 (CH), 76.6 (OCH), 69.7 (OCH_2), 52.3 (OMe), 21.9 (Me); HRMS (NSI) $C_{19}H_{24}NO_3$ ($[M + NH_4]^+$) requires 314.1751, found 314.1750; CSP-HPLC (Chiralcel OD-H, 99.5:0.5 hexane:IPA, 1.0 mL min^{-1}) enantiomers at 20.73 and 26.62 min.

Methyl (E)-4-(((4-phenylbut-3-en-2-yl)oxy)methyl)benzoate 5hb from **(*S*, *E*)-4-Phenylbut-3-en-2-ol (*S*)-4i**

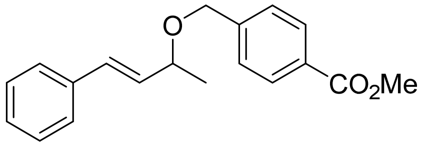

5hb

Following general procedure **C**, PPh_3AuNTf_2 (2:1) toluene adduct (2 × 4 mg, 2 × 0.00254 mmol), (*S*, *E*)-4-Phenylbut-3-en-2-ol (*S*)-**4i** (15 mg, 0.101 mmol, >99:1 er), methyl 4-

hydroxymethylbenzoate (84 g, 0.506 mmol) and 3 Å molecular sieves (8 mg) in PhMe (260 μL) gave the crude product. Purification by flash column chromatography on silica with 49:1→9:1 pentane:Et_2O gave benzoate **5hb** (22 mg, 73%, >20:1 *E*:*Z* by ^{1}H NMR, 50:50 er by CSP-HPLC) as a colourless oil.

(*S*, *E*)-Methyl 4-((1-(benzyloxy)pent-3-en-2-yloxy)methyl)benzoate (*S*)-5jb

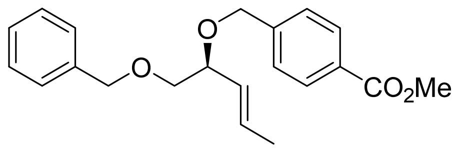

(*S*)-**5jb**

Following general procedure **C**, PPh_3AuNTf_2 (2:1) toluene adduct (2 × 4 mg, 2 × 0.00254 mmol), (*S*,*E*)-5-(benzyloxy)pent-3-en-2-ol (*S*)-**4j** (19 mg, 0.101 mmol, >99:1 er), methyl 4-hydroxymethylbenzoate (84 mg, 0.506 mmol) and 3 Å molecular sieves (8 mg) in PhMe (260 μL) gave the crude product. Purification by flash column chromatography on silica with 49:1→9:1 pentane:Et_2O as eluent gave benzoate (*S*)-**5jb** (20 mg, 59%, 6:1 *E*:*Z* by ^{1}H NMR, 98:2 er (of *E* alkene) and 62:38 er (of *Z* alkene) by CSP-HPLC) as a colourless oil, R_F 0.1 (9:1 pentane:Et_2O); IR ($CHCl_3$) 2857, 1719 (C=O), 1614, 1436, 1280, 1217, 1176, 1108, 1020, 969, 751, 698, 667 cm^{-1}; ^{1}H NMR (300 MHz, $CDCl_3$) (6:1 *E*:*Z* alkenes) δ H 8.03-7.95 (2.44H, m, *E* + *Z* Ar), 7.46-7.38 (2.44H, m, *E* + *Z* Ar), 7.37-7.23 (6.1H, m, *E* + *Z* Ar), 5.79-5.67 (0.22H, m, *Z* MeC*H*=), 5.74 (1H, dqd, *J* = 15.5, 6.5, 0.7 Hz, *E* MeC*H*=), 5.43-5.33 (0.22H, m, *Z* OCHC*H*=), 5.42 (1H, ddq, *J* = 15.5, 7.9, 1.6 Hz, *E* OCHC*H*=), 4.72-4.47 (4.88H, m, 2 × *E* + *Z* OCH_2Ar), 4.47-4.40 (0.22H, m, *Z* OCH), 4.03-3.95 (1H, m, *E* OCH), 3.91 (3.66H, br s, *E* + *Z* OMe), 3.63 (0.22H, dd, *J* = 10.5, 7.0 Hz, *Z* OCH_AH_BCH), 3.60 (1H, dd, *J* = 10.5, 6.7 Hz, *E* OCH_AH_BCH), 3.52 (1H, dd, *J* = 10.5, 4.3 Hz, *E* OCH_AH_BCH), 3.49 (0.22H, dd, *J* = 10.5, 5.7 Hz, *Z* OCH_AH_BCH), 1.74 (3H, dd, *J* = 6.5, 1.6 Hz, *E* =CH*Me*), 1.63 (0.22H, dd, *J* = 7.0, 1.7 Hz, *Z* =CH*Me*); ^{13}C NMR (75 MHz, $CDCl_3$) (*Z* alkene isomer not observed) δ C 167.2 (C=O), 144.4 (*ipso*-Ar), 138.5 (*ipso*-Ar), 130.7 (CH), 129.7 (CH), 129.2 (*ipso*-Ar), 128.5 (CH), 127.8 (CH), 127.3 (CH), 79.7 (OCH), 73.5 (OCH_2), 73.4 (OCH_2), 69.7 (CH_2), 52.2 (OMe), 18.0 (Me); HRMS (NSI) $C_{21}H_{24}O_4Na$ ($[M+Na]^+$) requires 363.1567, found 363.1567; CSP-HPLC (Chiralcel OD-H, 99.5:0.5 hexane:IPA, 0.5 mL min^{-1}) (*S*, *Z*)-**5jb** 31.78 min, (*R*, *Z*)-**5jb** 40.10 min, (*R*, *E*)-**5jb** 44.14 min, (*S*, *E*)-**5jb** 49.53 min.

(*S*, *E*)-Methyl 4-((1-(benzyloxy)pent-3-en-2-yloxy)methyl)benzoate (*S*)-5jb (Scheme 6, Eq. 2)

A solution of PPh_3AuNTf_2 (2:1) toluene adduct (4 mg, 0.00254 mol), (*S*,*E*)-5-(benzyloxy)pent-3-en-2-ol (*S*)-**4j** (19 mg, 0.101 mmol, >99:1 er), and methyl 4-hydroxymethylbenzoate (84 mg, 0.506 mmol) in PhMe (260 μL) was stirred at 50 °C under air for 8 h. A second portion of PPh_3AuNTf_2 (2:1) toluene adduct (4 mg, 0.00254 mol) was added, and the resulting solution was stirred at 50 °C under air for 8 h. The resulting solution was filtered over a short plug of silica, washing with 9:1 hexane:Et_2O. The filtrate was

evaporated under reduced pressure to give the crude product. Purification by flash coumn chromatography on silica with 49:1→9:1 pentane:Et_2O as eluent gave benzoate (*S*)-**5jb** (27 mg, 79%, 2:1 formal S_N2' (**5jb**):formal S_N2 (**6jb**) and >20:1 *E*:*Z* by ^{1}H NMR, 97:3 er (of S_N2' product **5jb**) by CSP-HPLC) as a colourless oil.

(*E*)-Methyl 4-((1-(benzyloxy)pent-3-en-2-yloxy)methyl)benzoate *rac*-5jb (Scheme 11)

A solution of PPh_3AuNTf_2 (2:1) toluene adduct (4 mg, 0.00254 mol), (*S*,*E*)-5-(benzyloxy)pent-3-en-2-ol (*S*)-**4j** (19 mg, 0.101 mmol, >99:1 er), and methyl 4-hydroxymethylbenzoate (336 mg, 2.02 mmol) in PhMe (260 μL) was stirred at 50 °C under air for 8 h. A second portion of PPh_3AuNTf_2 (2:1) toluene adduct (4 mg, 0.00254 mol) was added, and the resulting solution was stirred at 50 °C under air for 16 h. The resulting solution was filtered over a short plug of silica, washing with 9:1 hexane:Et_2O. The filtrate was evaporated under reduced pressure to give the crude product. Purification by flash coumn chromatography on silica with 49:1→9:1 pentane:Et_2O as eluent gave benzoate (*S*)-**5jb** (27 mg, 79%, 1:1 formal S_N2' (**5jb**):formal S_N2 (**6jb**). Formal S_N2' product **5jb** was present in 20:1 *E*:*Z* and formal S_N2 was present in 10:1 *E*:*Z* ratios by ^{1}H NMR, 50:50 er (of S_N2' product (*E*)-**5jb**) and 50:50 er (of S_N2' product (*Z*)-**5jb**) by CSP-HPLC) as a colourless oil.

(*S*, *E*)-Methyl 4-((pent-3-en-2-yloxy)methyl)benzoate (*S*)-4k

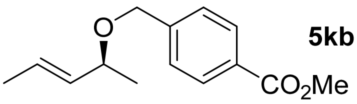

5kb

Following general procedure **C**, PPh_3AuNTf_2 (2:1) toluene adduct (2 × 4 mg, 2 × 0.00254 mmol), (*R*, *E*)-pent-3-en-2-ol (*R*)-**4k** (9mg, 0.101 mmol, >99:1 er), methyl 4-hydroxymethylbenzoate (84 mg, 0.506 mmol) and 3 Å molecular sieves (8 mg) in PhMe (260 μL) gave the crude product. Purification by flash column chromatography on silica with 49:1→9:1 hexane:Et_2O as eluent gave (*S*)-**5kb** (19 mg, 79%, 9:1 *E*:*Z* by ^{1}H NMR, 29:71 er (of *E* alkene) by CSP-HPLC) as a colourless oil, R_F 0.2 (9:1 hexane:Et_2O); IR (film) 2969, 1723 (C=O), 1614, 1435, 1274, 1192, 1174, 1104, 1085, 1019, 967, 908, 846, 798, 754, 702 cm^{-1}; ^{1}H NMR (300 MHz, $CDCl_3$) (10:1 mixture of *E*:*Z* alkenes) δ H 8.04-7.96 (2.2H, m, Ar), 7.44-7.36 (2.2H, m, Ar), 5.72-5.59 (0.1H, m, *Z* MeC*H*=), 5.64 (1H, dqd, *J* = 15.3, 6.5, 0.5 Hz, *E* MeC*H*=), 5.40 (1H, ddq, *J* = 15.3, 7.9, 1.6 Hz, *E* OCHC*H*=), 5.43-5.32 (0.1H, m, *Z* OCHC*H*=), 4.60 (0.1H, d, *J* = 12.7 Hz, *Z* OCH_ACH_B), 4.59 (1H, d, *J* = 12.7 Hz, *E* OC$H_A$$H_B$), 4.47-4.37 (1.1H, m, *E* + *Z* OC$H_A$$H_B$), 4.33 (0.1H, dqd, *J* = 8.9, 6.3, 1.0 Hz, *Z* OCH), 3.91 (3.3H, br s, *E* + *Z* OMe), 3.89 (1H, br dq, *J* = 7.9, 6.7 Hz, *E* OCH), 1.72 (3H, dd, *J* = 6.5, 1.6 Hz, *E* *Me*CH=), 1.62 (0.3H, dd, *J* = 6.9, 1.7 Hz, *Z* *Me*CH=), 1.28 (3H, d, *J* = 6.7 Hz, *E* OCH*Me*), 1.27 (0.3H, d, *J* = 6.3 Hz, *Z* OCH*Me*); ^{13}C NMR (75 MHz, $CDCl_3$) (mixture of *E* and *Z* alkenes) δ 167.2 (*E* + *Z* C=O), 144.6 (*ipso*-Ar), 144.5 (*ipso*-Ar), 133.1 (CH), 132.7 (CH), 129.8 (CH), 129.8 (CH), 129.3 (*ipso*-Ar), 129.2 (*ipso*-Ar), 128.2 (CH), 127.4 (CH), 127.3 (CH), 127.0 (CH), 76.4 (OCH), 70.3 (OCH), 69.3 (OCH_2), 69.2 (OCH_2), 52.2 (*E* + *Z*

OMe), 22.8 (Me), 21.7 (Me), 21.5 (Me), 17.8 (Me); HRMS (NSI) $C_{14}H_{19}O_3$ ($[M+H]^+$) requires 235.1329, found 235.1329; CSP-HPLC (Chiralcel OD-H, 99.8:0.2 hexane:IPA, 1.0 mL min^{-1}) (*R*, *E*)-**5kb** 9.43 min, (*S*, *E*)-**5kb** 10.44 min, *Z* alkene peaks inseperable.

(*R*, *E*)-Methyl 4-((5,5-dimethylhex-3-en-2-yloxy)methyl)benzoate (*R*)-5lb

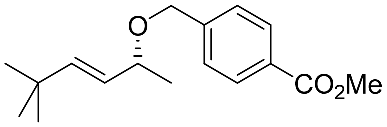

(*R*)-**5lb**

Following general procedure **C**, PPh_3AuNTf_2 (2:1) toluene adduct (2 × 4 mg, 2 × 0.00254 mmol), (*R*, *E*)-2,2-dimethylhex-4-en-3-ol (*R*)-**4l** (13 mg, 0.101 mmol, 92:8 er), methyl 4-hydroxymethylbenzoate (84 mg, 0.506 mmol) and 3 Å molecular sieves (8 mg) in PhMe (260 μL) gave the crude product. Purification by flash column chromatography on silica with 49:1→9:1 hexane:Et_2O as eluent gave benzoate (*R*)-**5lb** (20mg, 71%, >20:1 *E*:*Z* by ^{1}H NMR, 67:33 er by CSP-HPLC) as a colourless oil, R_F 0.3 (9:1 hexane:Et_2O); $[\alpha]_D$ = 11.1 (*c* = 0.18 in $CHCl_3$); IR ($CHCl_3$) 2959, 2864, 1724 (C=O), 1435, 1365, 1277, 1193, 1145, 1108, 1082, 1020, 975, 755 cm^{-1}; ^{1}H NMR (300 MHz, $CDCl_3$) δ 8.00 (2H, d, *J* = 8.4 Hz, Ar), 7.39 (2H, d, *J* = 8.4 Hz, Ar), 5.62 (1H, d, *J* = 15.7 Hz, ^{t}BuC*H*=), 5.27 (1H, dd, *J* = 15.7, 7.9 Hz, OCHC*H*=), 4.57 (1H, d, *J* = 12.8 Hz, OCH_ACH_B), 4.41 (1H, d, *J* = 12.8 Hz, OCH_ACH_B), 3.91 (3H, s, OMe), 3.86 (1H, dqd, *J* = 7.9, 6.4, 0.6 Hz, OCH), 1.29 (3H, d, *J* = 6.4 Hz, OCH*Me*), 1.02 (9H, s, ^{t}Bu); ^{13}C NMR (100.6 MHz, $CDCl_3$) δ 167.2 (C=O), 144.8 (CH), 144.7 (*ipso*-Ar), 129.8 (CH), 129.2 (*ipso*-Ar), 127.4 (CH), 126.3 (CH), 77.4 (OMe), 69.1 (OCH_2), 52.2 (OCH), 33.0 (CMe_3), 29.7 (CMe_3), 22.0 (OCH*Me*); HRMS (NSI) $C_{17}H_{25}O_3$ ($[M+H]^+$) requires 277.1798, found 277.1801; CSP-HPLC (Chiralpak IB, 99.9:0.1 hexane:IPA, 1.0 mL min^{-1}) (*S*, *E*)-**5lb** 7.80 min, (*R*, *E*)-**5lb** 8.29 min.

(*E*)-Methyl 4-((3-methylpent-3-en-2-yloxy)methyl)benzoate 5mb

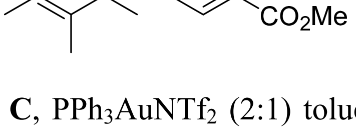

5mb

Following general procedure **C**, PPh_3AuNTf_2 (2:1) toluene adduct (2 × 4 mg, 2 × 0.00254 mmol), (*R*,*E*)-3-methylpent-3-en-2-ol (*R*)-**4m** (10 mg, 0.101 mmol, 99:1 er), methyl 4-hydroxymethylbenzoate (84 mg, 0.506 mmol) and 3 Å molecular sieves (8 mg) in PhMe (260 μL) gave the crude product. Purification by flash column chromatography on silica with 49:1→9:1 hexane:Et_2O as eluent gave **5mb** (18 mg, 72% and >20:1 *E*:*Z* by ^{1}H NMR, 49:51 by CSP-HPLC) as a colourless oil, R_F 0.3 (9:1 hexane:Et_2O); 2978, 1721 (C=O), 1613, 1435, 1415, 1370, 1275, 1217, 1192, 1175, 1104, 1087, 1019, 983, 895, 836, 752, 704, 667, 595, 568, 563, 553 cm^{-1}; ^{1}H NMR (300 MHz, $CDCl_3$) δ 8.00 (2H, dapp. t, *J* = 8.3, 1.8 Hz, Ar), 7.39 (2H, d, *J* = 8.3 Hz, Ar), 5.44 (1H, qq, *J* = 6.6, 0.6 Hz, =CH), 4.47 (1H, d, *J* = 12.7 Hz,

OCH_AH$_B$), 4.29 (1H, d, J = 12.7 Hz, OCH$_A$$H_B$), 3.91 (3H, s, OMe), 3.85 (1H, q, J = 6.7 Hz, OCH), 1.64 (3H, dq, J = 6.6, 1.0 Hz, =CH*Me*), 1.60 (3H, dq, J = 1.0, 0.6 Hz, =C*Me*), 1.27 (3H, d, J = 6.7 Hz, OCH*Me*); ^{13}C NMR (100.6 MHz, $CDCl_3$) δ 167.2 (C=O), 144.8 (*ipso*-Ar), 136.6 (*ipso*-Ar), 129.8 (CH), 129.3 (CH), 127.3 (CH), 122.2 (CH), 81.2 (OCH), 69.2 (OCH_2), 52.1 (OMe), 20.2 (Me), 13.2 (Me), 10.4 (Me); HRMS (NSI) $C_{15}H_{21}O_3$ ($[M+H]^+$) requires 249.1485, found 249.1485; CSP-HPLC (Chiralpak IB, 99.9:0.1 hexane:IPA, 1.0 mL min^{-1}) peaks at 9.20 and 10.34 min.

Methyl (*S*,*E*)-4-(((3-methylpent-3-en-2-yl)oxy)methyl)benzoate (*S*)-5mb

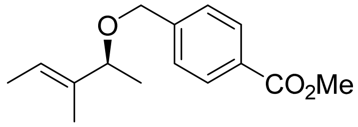

(*S*)-**5mb**

To a stirred solution of (*R*,*E*)-3-methylpent-3-en-2-ol (*R*)-**4m** (100 mg, 1.0 mmol, >991 er by CSP-GC) in THF (3 mL) was added NaH (60 mg of a 60% suspension in mineral oil, 1.5 mmol). The resulting solution was stirred at rt under Ar for 30 min. Then, a solution of methyl 4-(bromomethyl)benzoate (344 mg, 1.5 mml) in THF (2 mL) was added. The resulting solution was stirred at rt under Ar for 16 h. Then, saturated $NH_4.Cl_{(aq)}$ (6 mL) was added. The two layers were seperated, extracting the aqueous with Et_2O (3 × 10 mL). The combined organic layers were dried ($MgSO_4$) and evaporated under reduced pressure to give the crude product. Purification by flash column chromatography on silica with 49:1→9:1 pentane:Et_2O as eluent gave methyl (*S*,*E*)-4-(((3-methylpent-3-en-2-yl)oxy)methyl)benzoate (*S*)-**5mb** (12 mg, 5%, 98:2 er by CSP-HPLC) as a colourless oil.

"Resubjection" of (*E*)-Methyl 4-((3-methylpent-3-en-2-yloxy)methyl)benzoate 5mb (Scheme 3, Eq. 3)

PPh_3AuNTf_2 (2:1) toluene adduct (4 mg, 0.002 mmol) was added to (*E*)-Methyl 4-((3-methylpent-3-en-2-yloxy)methyl)benzoate (*S*)-**5mb** (12 mg, 0.048 mmol, 98:2 er (of *E* alkene), 3 Å molecular sieves (4 mg) and methyl 4-hydroxymethylbenzoate (32 mg, 0.19 mmol) in PhMe (124 μL). The resulting solution was stirred at 50 °C for 24 h. The resulting solution was filtered over a plug of silica, eluting with 9:1 pentane:Et_2O. The filtrate was evaporated under reduced pressure to give the crude product. Purification by flash column chromatography on silica with 49:1→9:1 hexane:Et_2O as eluent gave benzoate **5mb** (6 mg, 50%, 54:46 er (of *E* alkene) by CSP-HPLC) as a colourless oil.

(*R*, *E*)-Methyl 4-((4-cyclohexyl-3-methylbut-3-en-2-yloxy)methyl)benzoate (*R*)-5nb

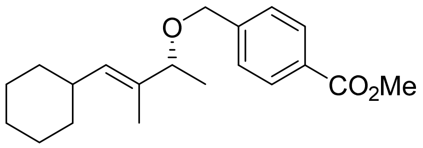

(*R*)-**5nb**

Following general procedure **C**, PPh_3AuNTf_2 (2:1) toluene adduct (2 × 4 mg, 2 × 0.00254 mmol), (*R*, *E*)-1-cyclohexyl-2-methylbut-2-en-1-ol (*R*)-**4n** (9 mg, 0.101 mmol, 92:8 er), methyl 4-hydroxymethylbenzoate (84 mg, 0.506 mmol) and 3 Å molecular sieves (8 mg) in PhMe (260 μL) gave the crude product. Purification by flash column chromatography on silica with 49:1→9:1 hexane:Et_2O as eluent gave (*R*)-**5nb** (20 mg, 62%, >20:1 *E*:*Z* by ^{1}H NMR, 53:47 er by CSP-HPLC) as a colourless oil, R_F0.3 (9:1 hexane:Et_2O); IR ($CHCl_3$) 2923, 2850, 1723 (C=O), 1435, 1310, 1275, 1192, 1174, 1104, 1080, 1019, 976, 896, 852, 753, 704 cm^{-1}; ^{1}H NMR (300 MHz, $CDCl_3$) δ 8.00 (2H, dd, *J* = 8.3, 1.7 Hz, Ar), 7.38 (2H, br d, *J* = 8.3 Hz, Ar), 5.18 (1H, dd, *J* = 8.9, 0.7 Hz, =CH), 4.47 (1H, d, *J* = 12.7 Hz, OCH_AH_B), 4.29 (OCH_AH_B), 3.91 (3H, s, OMe), 3.79 (1H, qd, *J* = 6.4, 0.2 Hz, OCH), 2.31-2.15 (1H, m, =CHC*H*), 1.79-1.55 (5H, m, Cy), 1.60 (3H, d, 0.7 Hz, =CMe), 1.40-0.94 (m, 5H, Cy), 1.26 (3H, d, *J* = 6.4 Hz, OCH*Me*); ^{13}C NMR (75 MHz, $CDCl_3$) δ 167.2 (C=O), 144.7 (*ipso*-Ar), 134.8 (CH), 133.3 (*ipso*-Ar), 129.8 (CH), 129.2 (CH), 127.4 (CH), 81.1 (OCH), 68.9 (OCH_2), 52.2 (OMe), 36.8 (*C*HCH=), 33.5 (CH_2), 33.1 (CH_2), 26.2 (CH_2), 26.1 (CH_2), 26.1 (CH_2), 20.3 (=C*Me*), 10.6 (OCH*Me*); HRMS (NSI) $C_{20}H_{29}O_4$ ($[M+H_2O–H]^+$) requires 333.2060, found 333.2062; CSP-HPLC (Chiralpak IA, 99.9:0.1 hexane:IPA, 1.0 mL min^{-1}) (*R*, *E*)-**5nb** 14.99 min, (*S*, *E*)-**5nb** 22.53 min.

2.4) Nucleophile Alcohol Scope

(*S*, *E*)-((Oct-3-en-2-yloxy)methyl)benzene (*S*)-5ac

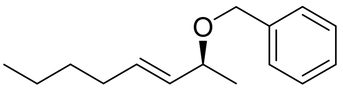

(*S*)-**5ac**

Following general procedure **C**, PPh_3AuNTf_2 (2:1) toluene adduct (2 × 4 mg, 2 × 0.00254 mmol), (*R*,*E*)-oct-2-en-4-ol (*R*)-**4a** (13 mg, 0.101 mmol, >99:1 er), benzyl alcohol (55 mg, 52 μL, 0.506 mmol) and 3 Å molecular sieves (8 mg) in PhMe (260 μL) gave the crude product. Purification by flash column chromatography on silica with 49:1→9:1 pentane:Et_2O as eluent gave ether (*S*)-**5ac** (18 mg, 82% and 10:1 *E*:*Z* by ^{1}H NMR, 97:3 er (of *E* alkene) and >99:1 er (of *Z* alkene) by CSP-HPLC) as a colourless oil, R_F 0.2 (49:1 pentane:Et_2O); IR (film) 2957, 2927, 2858, 1495, 1454, 1369, 1305, 1202, 1093, 1069, 1028, 970, 914, 841, 731, 615 cm^{-1}; ^{1}H NMR (300 MHz, $CDCl_3$) (10:1 mixture of *E*:*Z* alkenes) δ H 7.36-7.31 (4.4H, m, *E* + *Z* *o*- and *m*-Ar), 7.30-7.22 (1.1H, m, *E* + *Z* *p*-Ar), 5.61 (1H, d app. t, *J* = 15.4, 6.7 Hz, *E* =C*H*CH_2), 5.56 (0.1H, d app. t d, *J* = 11.1, 7.5, 0.8 Hz, *Z* =C*H*CH_2), 5.39 (1H, dd app. t, *J* = 15.4, 7.8, 1.3 Hz, *E* OCHC*H*=), 5.40-5.31 (0.1H, m, *Z* OCHC*H*=), 4.57 (0.1H, d, *J* = 11.8 Hz, *Z* OCH_AH_B), 4.56 (1H, d, *J* = 12.0 Hz, *E* CH_AH_B), 4.36 (1H, d, *J* = 12.0 Hz, *E* OCH_AH_B), 4.36 (0.1H, d, *J* = 11.8 Hz, *Z* OCH_AH_B), 3.88 (1H, dq, *J* = 7.6, 6.7 Hz, *E* OCH), 3.71-3.61 (0.1H, m, *Z* OCH), 2.13-2.00 (2.2H, m, *E* + *Z* =CHCH_2), 1.74 (0.3H, dd, *J* = 6.4, 1.6 Hz, *Z*

OCH*Me*), 1.40-1.25 (4.4H, m, *E* + *Z* MeC*H*$_2$ and MeCH$_2$C*H*$_2$), 1.27 (3H, d, *J* = 6.7 Hz, *E* OCH*Me*), 0.95-0.87 (3.3H, m, *E* + *Z* CH$_2$*Me*); ^{13}C NMR (75 MHz, CDCl$_3$) (10:1 mixture of *E*:*Z* alkenes) δ C 139.2 (*ipso*-Ar), 139.2 (*ipso*-Ar), 133.5 (=CH), 132.7 (=CH), 132.1 (=CH), 132.0 (=CH), 128.4 (Ar), 127.8 (Ar), 127.4 (Ar), 76.0 (OCH), 70.3 (OCH), 69.9 (OCH$_2$), 69.7 (OCH$_2$), 35.6 (CH$_2$), 32.0 (CH$_2$), 31.5 (CH$_2$), 27.5 (CH$_2$), 22.5 (CH$_2$), 22.4 (CH$_2$), 21.9 (Me), 21.8 (Me), 14.2 (Me), 14.1 (Me); HRMS (APCI) $C_{15}H_{26}NO$ ([M+NH$_4$]$^+$) requires 236.2009, found 236.2002; CSP-HPLC (Chiralcel OD-H, 99.9:0.1 hexane:IPA, 1.0 mL min^{-1}) (*S*, *E*)-**5ac** 5.42 min, (*R*, *E*)-**5ac** 6.04 min, (*S*, *Z*)-**5ac** 6.81 min, (*R*, *Z*)-**5ac** not observed.

(*S*, *E*)-1-Bromo-4-((oct-3-en-2-yloxy)methyl)benzene (*S*)-5ad

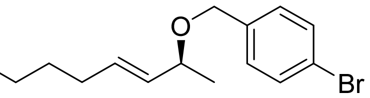

(*S*)-**5ad**

Following general procedure **C**, PPh$_3$AuNTf$_2$ (2:1) toluene adduct (2 × 4 mg, 2 × 0.00254 mmol), (*R*,*E*)-oct-2-en-4-ol (*R*)-**4a** (13 mg, 0.101 mmol, >99:1 er), 4-bromobenzyl alcohol (95 mg, 0.506 mmol) and 3 Å molecular sieves (8 mg) in PhMe (260 μL) gave the crude product. Purification by flash column chromatography on silica with 49:1→9:1 pentane:Et$_2$O as eluent gave ether (*S*)-**5ad** (22 mg, 73% and 6:1 *E*:*Z* by ^{1}H NMR, 87:13 er (of *E* alkene) and 25:75 er (of *Z* alkene) by CSP-HPLC of a derivative **5ac**, see below) as a colourless oil, R_F 0.6 (9:1 pentane:Et$_2$O); IR (film) 2957, 2926, 2857, 1487, 1465, 1369, 1069, 1012, 970, 914, 801 cm^{-1}; ^{1}H NMR (300 MHz, CDCl$_3$) (6:1 mixture of alkene isomers) δ H 7.48-7.41 (0.32H, m, *Z* Ar), 7.45 (2H, d, *J* = 8.3 Hz, *E* Ar), 7.23-7.16 (0.32H, m, *Z* Ar), 7.20 (2H, d, *J* = 8.3 Hz, *E* Ar), 5.60 (1H, dt, *J* = 15.4, 6.8 Hz, *E* CH$_2$C*H*=), 5.63-5.52 (0.16H, m, *Z* CH$_2$C*H*=), 5.36 (1H, ddt, *J* = 15.4, 7.9, 1.3 Hz, *E* OCHC*H*=), 5.38-5.28 (0.16H, m, *Z* OCHC*H*=), 4.51 (0.16H, d, *J* = 12.1 Hz, *Z* OC*H*$_A$H$_B$), 4.49 (1H, d, *J* = 11.7 Hz, *E* OC*H*$_A$H$_B$), 4.31 (1H, d, *J* = 11.7 Hz, *E* OCH$_A$*H*$_B$), 4.27 (0.16H, d, *J* = 12.1 Hz, *Z* OCH$_A$*H*$_B$), 3.85 (1H, dq, *J* = 7.9, 6.5 Hz, *E* OCH), 3.68-3.58 (0.16H, m, *Z* OCH), 2.12-1.96 (2.32H, m, *E* + *Z* =CHC*H*$_2$), 1.74 (0.48H, dd, *J* = 6.2, 1.3 Hz, *Z* OCH*Me*), 1.39-1.26 (4.64H, m, *E* + *Z* MeC*H*$_2$ + MeCH$_2$C*H*$_2$), 1.26 (3H, d, *J* = 6.5 Hz, *E* OCH*Me*), 0.95-0.86 (3.48H, m, *E* + *Z* CH$_2$*Me*); ^{13}C NMR (75.5 MHz, CDCl$_3$) (mixture of alkene isomers) δ C 138.3 (*ipso*-Ar), 138.2 (*ipso*-Ar), 133.7 (CH=), 132.9 (CH=), 132.2 (CH=), 131.8 (CH=), 131.5 (Ar), 131.5 (Ar), 129.4 (2 × Ar), 121.2 (2 × *ipso*-Ar), 80.5 (OCH), 76.2 (OCH), 70.4 (OCH$_2$), 69.0 (OCH$_2$), 35.6 (CH$_2$), 32.0 (CH$_2$), 31.5 (CH$_2$), 27.8 (CH$_2$), 22.5 (CH$_2$), 22.4 (CH$_2$), 21.9 (Me), 21.8 (Me), 14.2 (Me), 14.1 (Me); HRMS (APCI) $C_{15}H_{25}NOBr$ ([M+NH$_4$]$^+$) requires 314.1114, found 314.1107.

(*S*,*E*)-(3-(Oct-3-en-2-yloxy)methyl)benzene (*S*)-5ac from (*S*,*E*)-1-Bromo-4-((oct-3-en-2-yloxy)methyl)benzene (*S*)-5ad

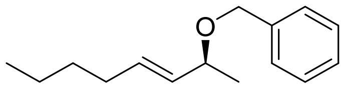

(*S*)-**5ac**

^{n}BuLi (55 μL of a 1.6 M solution in hexanes, 0.087 mmol) was added dropwise to a stirred solution of (*S*, *E*)-1-Bromo-4-((oct-3-en-2-yloxy)methyl)benzene (*S*)-**5ad** (22 mg, 0.067 mmol) in THF (1 mL) at –78 °C under Ar. The resulting solution was stirred at –78 °C for 10 min and saturated $NH_4Cl_{(aq)}$ (4 mL) was added. The layers were separated, extracting the aqueous with Et_2O (3 × 10 mL). The combined organic layers were dried ($MgSO_4$) and evaporated under reduced pressure. Purification by flash column chromatography gave ether (*S*)-**5ad** (12 mg, 80%, 6:1 *E*:*Z* by ^{1}H NMR, 87:13 er (of *E* alkene) and 25:75 er (of *Z* alkene) by CSP-HPLC).

(*S*, *E*)-1-Methoxy-4-((oct-3-en-2-yloxy)methyl)benzene (*S*)-5ae

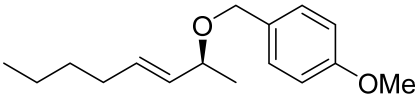

(*S*)-**5ae**

Following general procedure **C**, PPh_3AuNTf_2 (2:1) toluene adduct (2 × 4 mg, 2 × 0.00254 mmol), (*R*,*E*)-oct-2-en-4-ol (*R*)-**4a** (13 mg, 0.101 mmol, >99:1 er), 4-methoxybenzyl alcohol (70 mg, 0.506 mmol) and 3 Å molecular sieves (8 mg) in PhMe (260 μL) gave the crude product. Purification by flash column chromatography on silica with 49:1→9:1 pentane:Et_2O as eluent gave ether (*S*)-**5ae** (20 mg, 80% and 10:1 *E*:*Z* by ^{1}H NMR, >95:5 er (of *E* alkene) and >95:5 er (of *Z* alkene) by chiral shift ^{1}H NMR) as a colourless oil, R_F 0.4 (9:1 pentane:Et_2O); IR (film) 2956, 2927, 2857, 1613, 1512, 1464, 1368, 1301, 1245, 1171, 1069, 1036, 970, 820 cm^{-1}; ^{1}H NMR (300 MHz, $CDCl_3$) (10:1 *E*:*Z* alkene isomers) δ H 7.29-7.22 (1.1H, m, *E* + *Z* Ar), 6.91-6.82 (1.1H, m, *E* + *Z* Ar), 5.61 (1H, dt, *J* = 15.4, 6.7 Hz, *E* CH_2CH=), 5.59-5.50 (0.1H, m, *Z* CH_2CH=), 5.38 (1H, ddt, *J* = 15.4, 7.9, 1.4 Hz, *E* OCHC*H*=), 5.36-5.30 (0.1H, m, *Z* OCHC*H*=), 4.50 (0.1H, d, *J* = 11.6 Hz, *Z* OCH_AH_B), 4.49 (1H, d, *J* = 11.5 Hz, *E* OCH_AH_B), 4.29 (1H, d, *J* = 11.5 Hz, *E* OCH_AH_B), 4.26 (0.1H, d, *J* = 11.6 Hz, *Z* OCH_AH_B), 3.86 (1H, dq, *J* = 7.9, 6.5 Hz, *E* OCH), 3.86-3.76 (0.1H, m, *Z* OCH), 3.80 (3.3H, br s, *E* + *Z* OMe), 2.17-1.96 (2.2H, m, *E* + *Z* =$CHCH_2$), 1.75 (0.3H, dd, *J* = 6.4, 1.5 Hz, *Z* OCH*Me*), 1.43-1.28 (4.4H, m, *E* + *Z* $MeCH_2$ + $MeCH_2CH_2$), 1.25 (3H, d, *J* = 6.5 Hz, *E* OCH*Me*), 0.92 (3H, t, *J* = 7.1 Hz, *E* CH_2Me), 0.90 (0.3H, t, *J* = 6.3 Hz, *Z* CH_2Me); ^{13}C NMR (75 MHz, $CDCl_3$) (mixture of *E* and *Z* alkenes) δ C 159.1 (2 × *ipso*-Ar), 133.3 (CH), 132.5 (CH), 132.2 (CH), 132.1 (CH), 131.4 (*ipso*-Ar), 131.2 (*ipso*-Ar), 129.4 (CH), 128.5 (CH), 113.9 (CH), 113.8 (CH), 80.0 (OCH), 75.7 (OCH), 70.0 (OCH_2), 69.4 (OCH_2), 55.4 (2 × OMe), 35.6 (CH_2), 32.0 (CH_2), 31.5 (CH_2), 27.5 (CH_2), 22.8 (CH_2), 22.3 (CH_2), 21.9 (Me), 17.9 (Me), 14.2 (Me), 14.1 (Me); HRMS (APCI) $C_{16}H_{28}NO_2$ ($[M+NH_4]^+$) requires 266.2115, found 266.2112.

A sample of (*S*)-**5ae** from above (4.3 mg, 0.017 mmol) and (*R*)-(–)-1-(9-anthryl)-2,2,2-trifluoroethanol (19 mg, 0.068 mmol, 4 eq.) in $CDCl_3$ was analysed by ^{1}H NMR

spectroscopy. Inspection of the signal at 5.61 ppm (CH_2CH= of the *E* isomer) revealed no signal corresponding to (*R*, *E*)-**5ae** (compared to a corresponding sample of *racemic*-**5ae**. We thus conclude that the *E* alkene isomer of **5ae** is present in >95:5 er. Inspection of the signal at 1.75 ppm (OCH*Me* of the *Z* isomer) revealed no signal corresponding to (*R*, *Z*)-**5ae** (compared to a corresponding sample of *racemic*-**5ae**. We thus conclude that the *Z* isomer of **5ae** is present in >95:5 er.

(*S*, *E*)-2-((Oct-3-en-2-yloxy)methyl)furan (*S*)-5af

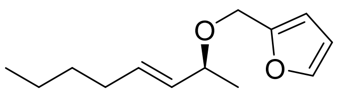

(*S*)-**5af**

Following general procedure **C**, PPh_3AuNTf_2 (2:1) toluene adduct (2 × 4 mg, 2 × 0.00254 mmol), (*R*,*E*)-oct-2-en-4-ol (*R*)-**4a** (13 mg, 0.101 mmol, >99:1 er), furfuryl alcohol (50 mg, 44 µL, 0.506 mmol) and 3 Å molecular sieves (8 mg) in PhMe (260 µL) gave the crude product. Purification by flash column chromatography on silica with 49:1→9:1 pentane:Et_2O as eluent gave ether (*S*)-**5af** (16 mg, 76%, 4:1 *E*:*Z* by ^{1}H NMR, 21:79 er (of *E* alkene) and 59:41 er (of *Z* alkene) by CSP-HPLC) as a colourless oil, R_F 0.6 (9:1 pentane:Et_2O), IR (film) 2957, 2927, 2857, 1150, 1065, 1014, 971, 919, 885, 807, 732, 599 cm^{-1}; ^{1}H NMR (300 MHz, $CDCl_3$) (4:1 mixture of alkene isomers) δ 7.42-7.37 (1.24H, m, *E* + *Z* Ar), 6.35-6.30 (1.24H, m, *E* + *Z* Ar), 6.28-6.24 (1.24H, m, *E* + *Z* Ar), 5.69-5.57 (0.24H, m, *Z* CH_2CH=), 5.64 (1H, dt, *J* = 15.4, 6.7 Hz, *E* CH_2CH=), 5.35 (1H, ddt, *J* = 15.4, 7.9, 1.4 Hz, *E* OCHC*H*=), 5.36-5.27 (0.24H, m, *Z* OCHC*H*=), 4.48 (0.24H, d, *J* = 12.6 Hz, *Z* OCH_AH_B), 4.47 (1H, d, *J* = 12.6 Hz, *E* OCH_AH_B), 4.31 (1H, d, *J* = 12.6 Hz, *E* OCH_AH_B), 4.28 (0.24H, d, *J* = 12.6 Hz, *Z* OCH_AH_B), 3.88 (1H, dq, *J* = 7.9, 6.4 Hz, *E* OCH), 3.65 (0.24H, q, *J* = 8.1 Hz, *Z* OCH), 2.13-2.00 (2.48H, m, *E* + *Z* =$CHCH_2$), 1.74 (0.72H, dd, *J* = 8.1, 1.5 Hz, *Z* OCH*Me*), 1.46-1.19 (7.96H, m, *E* OCH*Me* + *E* + *Z* $MeCH_2$ + $MeCH_2CH_2$), 0.95-0.85 (3.72H, m, *E* + *Z* CH_2*Me*); ^{13}C NMR (75 MHz, $CDCl_3$) (mixture of alkene isomers) δ 152.6 (2 × *ipso*-Ar), 142.7 (2 × Ar), 134.0 (CH=), 133.0 (CH=), 132.0 (CH=), 131.6 (CH=), 110.3 (2 × Ar), 108.9 (2 × Ar), 80.2 (OCH), 76.1 (OCH), 61.9 (OCH_2), 61.8 (OCH_2), 35.4 (CH_2), 32.0 (CH_2), 31.5 (CH_2), 29.9 (CH_2), 22.8 (CH_2), 22.4 (CH_2), 21.9 (Me), 17.9 (Me), 14.2 (Me), 14.1 (Me); HRMS (APCI) $C_{13}H_{21}O_2$ ([M+H]$^+$) requires 209.1536, found 209.1534; CSP-HPLC (Chiralcel OD-H, 99.9:0.1 hexane:IPA, 0.5 mL min^{-1}) (*S*, *Z*)-**5af** 8.93 min), (*R*, *Z*)-**5af** 9.27 min, (*R*, *E*)-**5af** 9.64 min, (*S*, *E*)-**5af** 9.91 min.

(*S*, *E*)-(3-(Oct-3-en-2-yloxy)propyl)benzene (*S*)-5ag

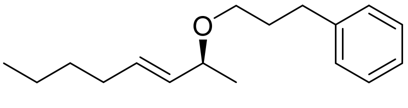

(*S*)-**5ag**

Following general procedure **C**, PPh_3AuNTf_2 (2:1) toluene adduct (2 × 4 mg, 2 × 0.00254 mmol), (*R*,*E*)-oct-2-en-4-ol (*R*)-**4a** (13 mg, 0.101 mmol, >99:1 er), 3-phenyl-1-propanol (69

mg, 69 μL, 0.506 mmol) and 3 Å molecular sieves (8 mg) in PhMe (260 μL) gave the crude product. Purification by flash column chromatography on silica with 49:1→9:1 pentane:Et_2O as eluent gave ether (*S*)-**5ag** (19 mg, 76% and 10:1 *E*:*Z* by ^{1}H NMR, 2:98 er (of *E* alkene) and 96:4 er (of *Z* alkene) by CSP-HPLC) as a colourless oil, R_F 0.2 (49:1 pentane:Et_2O); IR (film) 2955, 2927, 2856, 1496, 1454, 1368, 1317, 1153, 1098, 968, 909, 744 cm^{-1}; ^{1}H NMR (300 MHz, $CDCl_3$) (10:1 mixture of *E*:*Z* alkenes) δ H 7.34-7.14 (5.5H, m, Ar), 5.56 (1H, dt, *J* = 15.4, 6.6 Hz, *E* CH_2CH=), 5.57-5.43 (0.1H, m, *Z* CH_2CH=), 5.33 (1H, ddt, *J* = 15.4, 7.5, 1.3 Hz, *E* OCHC*H*=), 5.36-5.24 (0.1H, m, *Z* OCHC*H*=), 4.19 (0.1H, dqd, *J* = 8.8, 6.4, 0.9 Hz, *Z* OCH), 3.76 (1H, dq, *J* = 7.5, 7.5 Hz, *E* OCH), 3.55-3.43 (0.1H, m, *Z* $ArCH_AH_B$), 3.46 (1H, dt, *J* = 9.5, 6.6 Hz, *E* $ArCH_AH_B$), 3.36-3.19 (0.1H, m, *Z* $ArCH_AH_B$), 3.28 (1H, dt, *J* = 9.5, 6.5 Hz, *E* $ArCH_AH_B$), 2.74-2.62 (0.2H, m, *Z* OCH_2), 2.72-2.63 (2H, m, *E* OCH_2), 2.14-1.97 (2.2H, m, *E* + *Z* CH_2), 1.94-1.81 (2.2H, m, *E* + *Z* CH_2), 1.70 (0.3H, dd, *J* = 6.4, 1.6 Hz, *Z* OCH*Me*), 1.48-1.18 (4.4H, m, *E* + *Z* CH_2), 1.23 (3H, d, *J* = 7.5 Hz, *E* OCH*Me*), 0.95-0.84 (3.3H, m, *E* + *Z* CH_2Me); ^{13}C NMR (75 MHz, $CDCl_3$) (mixture of *E* and *Z* alkenes) δ C 142.3 (*ipso*-Ar), 132.7 (=CH), 132.5 (=CH), 132.3 (=CH), 132.1 (=CH), 128.6 (Ar), 128.4 (Ar), 125.8 (Ar), 76.6 (OCH), 71.0 (OCH), 67.3 (OCH_2), 67.2 (OCH_2), 32.6 (CH_2), 32.6 (CH_2), 32.0 (CH_2), 32.0 (CH_2), 31.7 (CH_2), 31.6 (CH_2), 31.5 (CH_2), 27.5 (CH_2), 22.5 (CH_2), 22.3 (CH_2), 21.9 (Me), 21.8 (Me), 14.2 (Me), 14.1 (Me); HRMS (APCI) $C_{17}H_{27}O$ ($[M+H]^+$) requires 247.2056, found 247.2050; CSP-HPLC (Chiralcel OD-H, 99.9:0.1 hexane:IPA, 0.5 mL min^{-1}) (*S*, *Z*)-**5ag** 10.55 min, (*R*, *Z*)-**5ag** 11.09 min, (*R*, *E*)-**5ag** 16.34 min, (*S*, *E*)-**5ag** 17.08 min.

(*S*, *E*)-2-(3,3,3-Trifluoropropoxy)oct-3-ene (*S*)-5ah

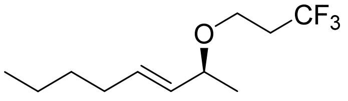

(*S*)-**5ah**

Following general procedure **C**, PPh_3AuNTf_2 (2:1) toluene adduct (2 × 4 mg, 2 × 0.00254 mmol), (*R*,*E*)-oct-2-en-4-ol (*R*)-**4a** (13 mg, 0.101 mmol, >99:1 er), 3,3,3-trifluoropropanol (58 mg, 45 μL, 0.506 mmol) and 3 Å molecular sieves (8 mg) in PhMe (260 μL) gave the crude product. Purification by flash column chromatography on silica with 99:1→19:1 pentane:Et_2O as eluent gave ether (*S*)-**5ah** (17 mg, 74%, 2:1 *E*:*Z* by ^{1}H NMR, 93:7 er (of *E* alkene) and 71:29 er (of *Z* alkene) by CSP-GC) as a colourless oil, R_F 0.4 (19:1 pentane:Et_2O); IR ($CHCl_3$) 2958, 2927, 2859, 1254, 1159, 1065, 968 cm^{-1}; ^{1}H NMR (300 MHz, $CDCl_3$) (2:1 mixture of *E*:*Z* alkenes) δ H 5.69-5.49 (1.7H, m, *E* + *Z* CH_2CH=), 5.47-5.32 (0.7H, m, *Z* OCHC*H*=), 5.33 (1H, ddt, *J* = 15.3, 7.9, 1.4 Hz, *E* OCHC*H*=), 3.94 (0.7H, dq, *J* = 6.3, 6.3 Hz, *Z* OCH), 3.81 (1H, dq, *J* = 7.9, 6.3 Hz, *E* OCH), 3.69 (1H, dt, *J* = 9.9, 6.9 Hz, *E* OCH_AH_B), 3.50 (0.7H, dt, *J* = 9.9, 6.8 Hz, *Z* OCH_AH_B), 2.49-2.31 (1.7H, m, *E* + *Z* OCH_AH_B), 2.14-1.97 (3.4H, m, *E* + *Z* =$CHCH_2$), 1.71 (2.1H, dd, *J* = 6.3, 1.5 Hz, *Z* OCH*Me*), 1.49-1.15 (13.2H, m, *E* OCH*Me* + *E* + *Z* CH_2), 0.93 (2.1H, t, *J* = 7.3 Hz, *Z* CH_2Me), 0.92 (3H, t, *J* = 7.0 Hz, *E* CH_2Me); ^{13}C NMR (75 MHz, $CDCl_3$) (mixture of alkene isomers) δ C 133.7 (=CH), 133.3 (=CH), 133.1 (=CH), 131.5 (=CH), 73.2 (OCH), 72.7 (OCH), 60.7 (OCH_2), 60.7 (OCH_2), 35.3 (CH_2), 35.2 (CH_2), 32.1 (CH_2), 32.0 (CH_2), 31.5 (CH_2), 31.4

(CH_2), 22.6 (q, J = 38.9 Hz, CF_3), 22.4 (CH_2), 22.3 (CH_2), 21.7 (Me), 20.9 (Me), 14.1 (Me), 14.0 (Me); CSP-GC (β-Dex, 65 °C, 35 cm s^{-1}) (*S*,*Z*)-**5ah** 26.54 min, (*R*,*Z*)-**5ah** 28.46 min, (*S*,*E*)-**5ah** 33.98 min, (*R*,*E*)-**5ah** 36.53 min.

(*S*,*E*)-2-(3-Chloropropoxy)oct-3-ene (*S*)-5ai

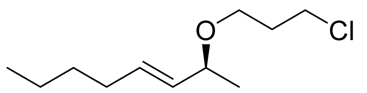

(*S*)-**5ai**

Following general procedure **C**, PPh_3AuNTf_2 (2:1) toluene adduct (2 × 4 mg, 2 × 0.00254 mmol), (*R*,*E*)-oct-2-en-4-ol (*R*)-**4a** (13 mg, 0.101 mmol, >99:1 er), 3-chloropropanol (48 mg, 42 µL, 0.506 mmol) and 3 Å molecular sieves (8 mg) in PhMe (260 µL) gave the crude product. Purification by flash column chromatography on silica with 99:1→49:1 hexane:Et_2O as eluent gave ether (*S*)-**5ai** (14 mg, 67% and 10:1 *E*:*Z* by ^{1}H NMR, 94:6 er (of *E* alkene) and 65:35 er (of *Z* alkene) by CSP-GC) as a colourless oil, R_F 0.2 (49:1 hexane:Et_2O) IR (film) 2958, 2927, 2859, 1099, 1070, 967, 727, 657 cm^{-1}; ^{1}H NMR (300 MHz, $CDCl_3$) (10:1 mixture of *E*:*Z* alkenes) δ H 5.59 (1H, dtd, J = 15.4, 6.8, 0.6 Hz, *E* CH_2CH=), 5.58-5.46 (0.1H, m, *Z* CH_2CH=), 5.31 (1H, ddt, J = 15.4, 7.7, 1.5 Hz, *E* OCHC*H*=), 5.30-5.22 (0.1H, m, *Z* OCHC*H*=), 4.21 (0.1H, dqd, J = 8.9, 6.3, 0.9 Hz, *Z* OCH), 3.77 (1H, dq, J = 7.7, 6.7 Hz, *E* OCH), 3.64 (1H, ddd, J = 6.5, 6.5, 0.8 Hz, *E* $ClCH_AH_B$), 3.62-3.59 (0.1H, m, *Z* $ClCH_AH_B$), 3.60-3.53 (0.1H, m, *Z* OCH_AH_B), 3.57 (1H, dt, J = 9.6, 6.0 Hz, *E* OCH_AH_B), 3.40 (1H, dt, J = 9.6, 5.8 Hz, *E* OCH_AH_B), 3.40-3.32 (0.1H, m, *Z* OCH_AH_B), 2.08-1.95 (2.2 H, m, *E* + *Z* =$CHCH_2$), 2.01-1.93 (0.1H, m, *Z* $ClCH_AH_B$), 1.99 (1H, ddd, J = 12.5, 6.5, 6.0 Hz, *E* $ClCH_AH_B$), 1.71 (0.3H, dd, J = 6.3, 1.6 Hz, *Z* OCH*Me*), 1.45-1.14 (m, 6.6H, *E* + *Z* CH_2), 1.21 (3H, d, J = 6.7 Hz, *E* OCH*Me*), 0.93-0.86 (3.3H, m, *E* + *Z* CH_2Me); ^{13}C NMR (75 MHz, $CDCl_3$) (mixture of *E* and *Z* alkenes) δ C 133.1 (=CH), 132.4 (=CH), 132.1 (=CH), 132.0 (=CH), 77.0 (OCH), 76.9 (OCH), 64.5 (OCH_2), 64.4 (OCH_2), 42.6 (CH_2), 42.4 (CH_2), 33.2 (CH_2), 33.1 (CH_2), 32.0 (CH_2), 32.0 (CH_2), 31.5 (2 × CH_2), 22.5 (CH_2), 22.4 (CH_2), 21.8 (CH_2Me), 21.7 (CH_2Me), 14.2 (OCH*Me*), 14.1 (OCH*Me*); HRMS (APCI) $C_{11}H_{20}OCl$ ([M – H]$^+$) requires 203.1197, found 203.1195; CSP-GC (β-Dex, 85 °C, 35 cm s^{-1}) (*S*, *Z*)-**5ai** 74.28 min, (*R*, *Z*)-**5ai** 75.80 min, (*S*, *E*)-**5ai** 97.15 min, (*R*, *E*)-**5ai** 98.71 min.

(*S*,*E*)-2-(3-Chloropropoxy)oct-3-ene (*S*)-5ai (Scheme 10, Eq. 2, using *unactivated* molecular sieves)

A solution of PPh_3AuNTf_2 (2:1) toluene adduct (4 mg, 0.00254 mmol), (*R*,*E*)-oct-2-en-4-ol (*R*)-**4a** (13 mg, 0.101 mmol, >99:1 er), 3-chloropropanol (48 mg, 42 µL, 0.506 mmol) and *unactivated* 3 Å molecular sieves (8 mg) in PhMe (260 µL) was stirred at 50 °C under air for 8 h. Then, PPh_3AuNTf_2 (2:1) toluene adduct (4 mg, 0.00254 mmol) was added and the resulting solution was stirred at 50 °C for 16 h. The resulting solution was filtered over a short plug of silica, washing with 9:1 hexane:Et_2O. The filtrate was evaporated under reduced pressure to give the crude product. Purification by flash column chromatography on silica

with 99:1→49:1 hexane:Et_2O as eluent gave ether (*S*)-**5ai** (19 mg, 90% and 12:1 *E*:*Z* by ^{1}H NMR, 98:2 er (of *E* alkene) and 78:22 er (of *Z* alkene) by CSP-GC) as a colourless oil.

(*E*)-2-(3-Chloropropoxy)oct-3-ene *Rac*-5ai (Scheme 10, Eq.1, no molecular sieves)

A solution of PPh_3AuNTf_2 (2:1) toluene adduct (4 mg, 0.00254 mmol), (*R*,*E*)-oct-2-en-4-ol (*R*)-**4a** (13 mg, 0.101 mmol, >99:1 er) and 3-chloropropanol (48 mg, 42 μL, 0.506 mmol) in PhMe (260 μL) was stirred at 50 °C under air for 8 h. Then, PPh_3AuNTf_2 (2:1) toluene adduct (4 mg, 0.00254 mmol) was added and the resulting solution was stirred at 50 °C for 16 h. The resulting solution was filtered over a short plug of silica, washing with 9:1 hexane:Et_2O. The filtrate was evaporated under reduced pressure to give the crude product. Purification by flash column chromatography on silica with 99:1→49:1 hexane:Et_2O as eluent gave ether (*S*)-**5ai** (11 mg, 52% and 12:1 *E*:*Z* by ^{1}H NMR, 53:47 er (of *E* alkene) and 51:49 er (of *Z* alkene) by CSP-GC) as a colourless oil.

(*E*)-2-(3-Chloropropoxy)oct-3-ene *Rac*-5ai (Scheme 10, Eq.3, 20 eq. allylic alcohol)

A solution of $Ph_3PAuNTf_2$ (2:1) toluene adduct (4 mg, 0.00254 mmol, (*R*, *E*)-oct-2-en-4-ol (*R*)-**4a** (13 mg, 0.101 mmoL, >99:1 er and 3-chloropropanol (191 mg, 169 μL, 2.02 mmol) in PhMe (260 μL) was stirred at 50 °C under air for 8 h. Then, PPh_3AuNTf_2 (2:1) toluene adduct (4 mg, 0.00254 mmol) was added and the resulting solution was stirred at 50 °C for 16 h. The resulting solution was filtered over a short plug of silica, washing with 9:1 hexane:Et_2O. The filtrate was evaporated under reduced pressure to give the crude product. Purification by flash column chromatography on silica with 99:1→49:1 hexane:Et_2O as eluent gave ether (*S*)-**5ai** (15 mg, 71% and 2:1 *E*:*Z* by ^{1}H NMR, 59:41 er (of *E* alkene) and 53:47 er (of *Z* alkene) by CSP-GC) as a colourless oil.

(*S*, *E*)-((4-(hex-5-enyloxy)pent-2-enyloxy)methyl)benzene (*S*)-5jj

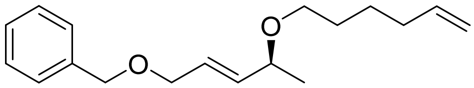

(*S*)-**5jj**

Following general procedure **C**, PPh_3AuNTf_2 (2:1) toluene adduct (2 × 4 mg, 2 × 0.00254 mmol), (*S*,*E*)-5-(benzyloxy)pent-3-en-2-ol (*S*)-**4j** (16 mg, 0.101 mmol, >99:1 er), 5-hexen-1-ol (51 mg, 61 μL, 0.506 mmol) and 3 Å molecular sieves (8 mg) in PhMe (260 μL) gave the crude product. Purification by flash column chromatography on silica with 49:1→9:1 pentane:Et_2O as eluent gave ether (*S*)-**5jj** (20 mg, 71% and 6:1 *E*:*Z* by ^{1}H NMR, 98:2 er (of *E* alkene) and 86:14 er (of *Z* alkene) by CSP-HPLC) as a colourless oil, R_F 0.4 (9:1 pentane:Et_2O); IR ($CHCl_3$) 2925, 1454, 1098, 910 cm^{-1}; ^{1}H NMR (300 MHz, $CDCl_3$) (6:1 *E*:*Z* mixture of alkene isomers) δ H (7.35-7.32 (2.28H, m, Ar), 7.29-7.26 (1.17H, m, Ar), 5.80 (1H, ddapp. t, *J* = 15.4, 6.9, 6.9 Hz, *E* OCHC*H*=), 5.86-5.74 (0.17H, m, *Z* OCHC*H*=), 5.76-5.65 (0.17H, m, *Z* MeC*H*=), 5.70 (1H, dqd, *J* = 15.4, 6.5, 1.0 Hz, *E* MeC*H*=), 5.38-5.29 (0.17H, m, *Z* CH_2C*H*=), 5.36 (1H, dddd, *J* = 15.5, 7.8, 3.3, 1.7 Hz, *E* CH_2C*H*=), 5.03-4.91

(m, 0.34H, *Z* =CH_2), 5.02-4.91 (m, 2H, *E* =CH_2), 4.62 (0.17H, d, *J* = 12.5 Hz, *Z* ArCH_AH_B), 4.61 (1H, d, *J* = 12.1 Hz, *E* ArCH_AH_B), 4.56 (0.17H, d, *J* = 12.5 Hz, *Z* ArCH_AH_B), 4.55 (1H, d, *J* = 12.1 Hz, *E* ArCH_AH_B), 3.89-3.82 (1.17H, m, *E* + *Z* OCH), 3.54-3.31 (4.68H, m, 2 × *E* + *Z* OCH_2), 2.10-2.02 (2.34H, m, *E* + *Z* =CHCH_2), 1.72 (3H, ddd, *J* = 6.9, 1.5, 0.6 Hz, *E* =CH*Me*), 1.68 (0.51H, dd, *J* = 6.9, 1.8 Hz, *Z* =CH*Me*), 1.63-1.55 (2.34H, m, *E* + *Z* CH_2), 1.49-1.42 (2.34H, m, *E* + *Z* CH_2); ^{13}C NMR (300 MHz, $CDCl_3$) (*Z* alkene not observed) δ C 139.0 (CH), 138.7 (*ipso*-Ar), 129.5 (CH), 129.4 (CH), 128.4 (CH), 127.8 (CH), 127.6 (CH), 114.5 (=CH_2), 79.9 (OCH), 73.5 (2 × OCH_2), 68.7 (OCH_2), 33.7 (CH_2), 29.5 (CH_2), 25.7 (CH_2), 18.0 (Me); HRMS (APCI) $C_{18}H_{30}NO_2$ ([M+NH_4]$^+$) requires 292.2271, found 292.2277; CSP-HPLC (Chiralcel OD-H, 99.9:0.1 hexane:IPA, 0.5 mL min^{-1}) (*S*, *Z*)-**5jj** 20.87 min, (*R*, *Z*)-**5jj** 26.10 min, (*R*, *E*,)-**5jj** 22.83 min, (*S*, *E*)-**5jj** 28.37 min.

(*S*, *E*)-2-Methyl-4-(oct-3-en-2-yloxy)butan-2-ol (*S*)-5ak

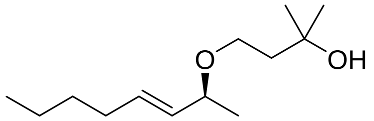

(*S*)-**5ak**

Following general procedure **C**, PPh_3AuNTf_2 (2:1) toluene adduct (2 × 4 mg, 2 × 0.00254 mmol), (*R*,*E*)-oct-2-en-4-ol (*R*)-**4a** (13 mg, 0.101 mmol, >99:1 er), 3-methyl-1,3-butane diol (58 mg, 60 μL, 0.506 mmol) and 3 Å molecular sieves (8 mg) in PhMe (260 μL) gave the crude product. Purification by flash column chromatography on silica with 4:1→2:1 pentane:Et_2O as eluent gave ether (*S*)-**5ak** (17 mg, 81% and 5:1 *E*:*Z* by ^{1}H NMR, 86:14 er (of *E* alkene) and 9:91 er (of *Z* alkene) by CSP-HPLC) as a colourless oil, R_F 0.1 (4:1 pentane:Et_2O); IR (film) 3445 (OH), 2967, 2927, 2860, 1466, 1400, 1370, 1273, 1217, 1152, 1090, 1062, 1038, 970, 937, 916, 880, 815, 728, 577, 566 cm^{-1}; ^{1}H NMR (300 MHz, $CDCl_3$) (5:1 mixture of alkene isomers) δ H 5.60 (1H, dt, *J* = 15.4, 6.8 Hz, *E* =C*H*CH_2), 5.55 (0.2H, dtd, *J* = 11.1, 7.6, 0.8 Hz, *Z* =C*H*CH_2), 5.31 (1H, ddt, *J* = 15.4, 7.9, 1.3 Hz, *E* OCHC*H*=), 5.31-5.21 (0.2H, m, *Z* OCHC*H*=), 4.21 (0.2H, dqd, *J* = 9.0, 6.3, 0.8 Hz, *Z* OCH), 3.76 (1H, dq, *J* = 7.9, 6.5 Hz, *E* OCH), 3.76-3.66 (1.2H, m, *E* + *Z* OCH_AH_B), 3.60 (1,2H, br s, *E* + *Z* OH), 3.55-3.42 (1.2H, m, *E* + *Z* OCH_AH_B), 2.13-1.96 (2.4H, m, *E* + *Z* =CHCH_2), 1.79-1.62 (3.0H, m, *E* + *Z* OCH_2CH_2 + *Z* OCH*Me*), 1.37-1.19 (12.6H, m, *E* + *Z* MeCH_2 + $MeCH_2CH_2$ + CMe_2 + *E* OCH*Me*), 0.94-0.83 (3.6H, m, *E* + *Z* CH_2Me); ^{13}C NMR (75 MHz, $CDCl_3$) (mixture of alkene isomers) δ C 133.7 (CH=), 132.9 (CH=), 131.6 (CH=), 131.5 (CH=), 77.5 (OCH), 71.6 (OCH), 70.7 (2 × OC), 65.5 (OCH_2), 65.4 (OCH_2), 41.5 (CH_2), 41.5 (CH_2), 32.0 (CH_2), 31.9 (CH_2), 31.5 (CH_2), 29.6 (Me), 29.5 (Me), 29.3 (2 × Me), 27.5 (CH_2), 22.5 (CH_2), 22.3 (CH_2), 21.9 (Me), 21.8 (Me), 14.1 (Me), 14.0 (Me); HRMS (APCI) $C_{13}H_{27}O_3$ ([M+H]$^+$) requires 215.2006, found 215.2000; CSP-HPLC (Chiralcel OD-H, 99.9:0.1 hexane:IPA, 0.5 mL min^{-1}) (*S*, *E*)-**5ak** 15.92 min, (*R*, *E*)-**5ak** 18.04 min, (*R*, *Z*)-**5ak** 41.73 min, (*S*, *Z*)-**5ak** 43.63 min.

2,2-Dimethyl-4-(((*S*, *E*)-oct-3-en-2-yloxy)methyl)-1,3-dioxolane (*S*)-5al

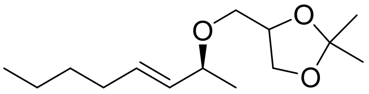

(*S*)-**5al**

Following general procedure **C**, PPh_3AuNTf_2 (2:1) toluene adduct (2 × 4 mg, 2 × 0.00254 mmol), (*R*,*E*)-oct-2-en-4-ol (*R*)-**4a** (13 mg, 0.101 mmol, >99:1 er), solketal (67 mg, 63 µL, 0.506 mmol) and 3 Å molecular sieves (8 mg) in PhMe (260 µL) gave the crude product. Purification by flash column chromatography on silica with 49:1→9:1 pentane:Et_2O as eluent gave ether (*S*)-**5al** (20 mg, 83%, >20:1 *E*:*Z* and 1:1.1 dr by ^{1}H NMR, 98:2 er (of first diastereomer off column) and 97:3 er (of second diastereomer off column) by CSP-GC) as a colourless oil, R_F 0.2 (9:1 pentane:Et_2O); IR (film) 2958, 2928, 2861, 1456, 1379, 1369, 1255, 1213, 1153, 1055, 971, 915, 844, 792, 738 cm^{-1}; ^{1}H NMR (300 MHz, $CDCl_3$) (1:1.1 mixture of diastereomers) δ H 5.65-5.52 (2.1H, m, d 1 and d 2 OCHC*H*=), 5.38-5.24 (2.1H, m, d 1 and d 2 CH_2CH=), 4.30-4.18 (2.1H, m, d 1 and d 2 OCH), 4.09-4.01 (2.1H, m, d 1 and d 2 OCH), 3.87-3.64 (4.2H, m, d 1 and d 2 2 × OCH), 3.55 (1.1H, dd, *J* = 9.8, 5.5 Hz, d 1 *or* d 2 OCH), 3.42-3.38 (2.1H, m, d 1 and d 2 OCH), 3.26 (1H, dd, *J* = 9.8, 5.9 Hz, d 1 *or* d 2 OCH), 2.08-1.98 (4.2H, m, d 1 and d 2 =CHCH_2), 1.47-1.26 (21H, m, d 1 and d 2 MeCH_2 + $MeCH_2CH_2$ + CMe_2), 1.25-1.19 (6.3H, m, d 1 and d 2 OCH*Me*), 0.94-0.85 (6.3H, m, d 1 and d 2 CH_2*Me*); ^{13}C NMR (75 MHz, $CDCl_3$) (mixture of diastereomers d 1 and d 2) δ 133.5 (=CH), 133.4 (=CH), 131.8 (=CH), 131.8 (=CH), 109.5 (O*C*O), 109.4 (O*C*O), 77.6 (OCH), 77.4 (OCH), 75.1 (OCH), 75.0 (OCH), 69.2 (OCH_2), 69.1 (OCH_2), 67.4 (OCH_2), 67.3 (OCH_2), 32.0 (2 × CH_2), 31.5 (2 × CH_2), 27.0 (Me), 26.9 (Me), 25.6 (2 × Me), 22.5 (CH_2), 22.3 (CH_2), 21.8 (Me), 21.7 (Me), 14.1 (2 × Me); HRMS (APCI) $C_{14}H_{30}NO_3$ ($[M+NH_4]^+$) requires 260.2220, found 260.2217; CSP-GC (β-Dex, 140 °C, 35 cm s^{-1}) (*S*-diastereomer 1) 16.30 min, (*R*-diastereomer 1) 16.73 min, (*R*-diastereomer 2) 16.99 min, (*S*-diastereomer 2) 17.40 min.

2-(2-((*S*, *E*)-Oct-3-en-2-yloxy)ethoxy)tetrahydro-2H-pyran (*S*)-5am

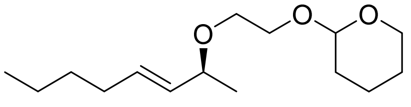

(*S*)-**5am**

Following general procedure **C**, PPh_3AuNTf_2 (2:1) toluene adduct (2 × 4 mg, 2 × 0.00254 mmol), (*R*,*E*)-oct-2-en-4-ol (*R*)-**4a** (13 mg, 0.101 mmol, >99:1 er), 2-tetrahydro-2H-pyran-2-yloxy)ethanol (82 mg, 76 µL, 0.506 mmol) and 3 Å molecular sieves (8 mg) in PhMe (260 µL) gave the crude product. Purification by flash column chromatography on silica with 49:1→9:1 pentane:Et_2O as eluent gave ether (*S*)-**5am** (20 mg, 77%, 10:1 *E*:*Z* and >20:1 dr by ^{1}H NMR, 7:93 er (of *E* alkene) and 10:90 er (of *Z* alkene) by CSP-HPLC) as a colourless oil, R_F 0.2 (9:1 pentane:Et_2O; IR ($CHCl_3$) 2927, 2871, 1124, 1074, 1035, 1020, 991, 971 cm^{-1}; ^{1}H NMR (300 MHz, $CDCl_3$) δ H 5.59 (1H, d app. t, *J* = 15.5, 6.7 Hz, *E* CH_2CH=), 5.53-5.45 (0.1H, m, *Z* CH_2CH=), 5.33 (1H, ddd app. t, *J* = 15.5, 7.6, 3.0, 1.3 Hz, *E* OCHC*H*=), 5.28-5.23 (0.1H, m, *Z* OCHC*H*=), 4.67-4.60 (1.1H, m, *E* + *Z* OCHO), 4.32-4.25 (0.1H, m, *Z* OCH), 3.95-3.77 (3.2H, m, *E* OCH + *E* + *Z* OCH_2), 3.68-3.42 (4.4H, m, *E* + *Z* OCH_2), 2.08-1.98 (2.2H, m, =CHCH_2), 1.74-1.47 (5.5H, m, *E* + *Z* CH_2), 1.40-1.20 (8.8H, m, *E* + *Z*

OCH*Me* + *E* + *Z* CH_2), 0.94-0.84 (3.3H, m, *E* + *Z* CH_2*Me*); ^{13}C NMR (75 MHz, $CDCl_3$) (mixture of *E* + *Z* alkenes) δ C 133.1 (=CH), 133.0 (=CH), 132.1 (2 × =CH), 99.1 (OCHO), 99.0 (OCHO), 77.0 (OCH), 77.0 (OCH), 67.1 (2 × OCH_2), 67.0 (OCH_2), 66.8 (OCH_2), 62.4 (OCH_2), 62.3 (OCH_2), 31.5 (2 × CH_2), 30.8 (CH_2), 30.7 (CH_2), 25.6 (2 × CH_2), 22.5 (CH_2), 22.4 (CH_2), 21.9 (Me), 21.8 (Me), 19.7 (CH_2), 19.6 (CH_2), 14.2 (Me), 14.1 (Me); HRMS (APCI) $C_{15}H_{29}O_3$ ($[M+H]^+$) requires 257.2111, found 257.2107; CSP-HPLC (Chiralpak IA, 99.8:0.2 hexane:IPA, 1.0 mL min^{-1}) (*R*, *E*)-**5am** 6.98 min, (*S*, *E*)-**5am** 7.31 min, (*R*, *Z*)-**5am** 9.19 min, (*S*, *E*)-**5am** 10.44 min.

(*S*, *E*)-(4-(Cyclohexyloxy)pent-2-enyl)benzene (*S*)-5gn

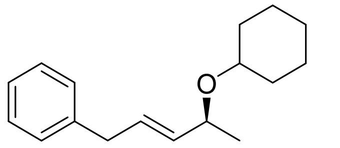

(*S*)-**5gn**

Following general procedure **C**, PPh_3AuNTf_2 (2:1) toluene adduct (2 × 4 mg, 2 × 0.00254 mmol), (*R*, *E*)-2-phenylpent-3-en-2-ol (*R*)-**4g** (16 mg, 0.101 mmol, >99:1 er), cyclohexanol (51 mg, 54 μL, 0.506 mmol) and 3 Å molecular sieves (8 mg) in PhMe (260 μL) gave the crude product. Purification by flash column chromatography on silica with 99:1→19:1 pentane:Et_2O as eluent gave ether (*S*)-**5gn** (19 mg, 76% and 14:1 *E*:*Z* by ^{1}H NMR, >99:1 er (of *E* alkene) and 7:93 er (of *Z* alkene) by CSP-HPLC) as a colourless oil, R_F 0.4 (19:1 pentane:Et_2O); IR (2971, 2928, 2854, 1495, 1451, 1366, 1137, 1076, 1025, 969, 887, 744, 697, 600 cm^{-1}; ^{1}H NMR (300 MHz, $CDCl_3$) (14:1 *E*:*Z*) δ H 7.37-7.28 (2.16H, m, *E* + *Z* Ar), 7.28-7.18 (3.24H, m, *E* + *Z* Ar), 5.75 (1H, dtd, *J* = 15.4, 6.9, 0.8 Hz, *E* $ArCH_2C$*H*=), 5.74-5.65 (0.08H, m, *Z* $ArCH_2C$*H*=), 5.51 (ddt, *J* = 15.4, 7.4, 1.4 Hz, *E* OCHC*H*=), 5.55-5.46 (0.08H, m, *Z* OCHC*H*=), 4.54 (0.08H, dqd, *J* = 8.7, 6.3, 0.9 Hz, *Z* OC*H*CH=), 4.03 (1H, dq, *J* = 7.4, 6.5 Hz, *E* OC*H*CH=), 3.51-3.27 (1.08H, m, *E* + *Z* OCH), 3.44-3.38 (2.16H, m, *E* + *Z* ArC*H*$_2$), 2.01-1.64 (4.56H, m, *E* + *Z* Cy + *Z* OCH*Me*), 1.61-1.49 (1.08H, m, *E* + *Z* Cy), 1.41-1.12 (5.4H, m, *E* + *Z* Cy), 1.26 (3H, d, *J* = 6.5 Hz, *E* OCH*Me*); ^{13}C NMR (75 MHz, $CDCl_3$) δ C 140.6 (*ipso*-Ar), 134.9 (CH), 130.0 (CH), 128.6 (CH), 128.5 (CH), 126.2 (CH), 74.6 (OCH), 73.0 (OCH), 38.8 (CH_2), 33.7 (CH_2), 32.3 (CH_2), 26.0 (CH_2), 24.7 (CH_2), 24.5 (CH_2), 22.3 (Me); HRMS (APCI) $C_{17}H_{28}NO$ ($[M+NH_4]^+$) requires 262.2165, found 262.2164; CSP-HPLC (Chiralpak IA, 100% hexane, 1.0 mL min^{-1}) (*R*, *Z*)-**5gn** 6.03 min, (*S*, *E*)-**5gn** 7.16 min, (*S*, *Z*)-**5gn** 9.81 min, (*R*, *E*)-**5gn** 12.39 min.

3) ^{1}H NMR and ^{13}C NMR Spectra and HPLC/GC Traces of Synthesised Compounds

(*R*,*E*)-Oct-2-en-4-ol (*R*)-4a

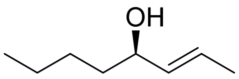

(*R*)-**4a**

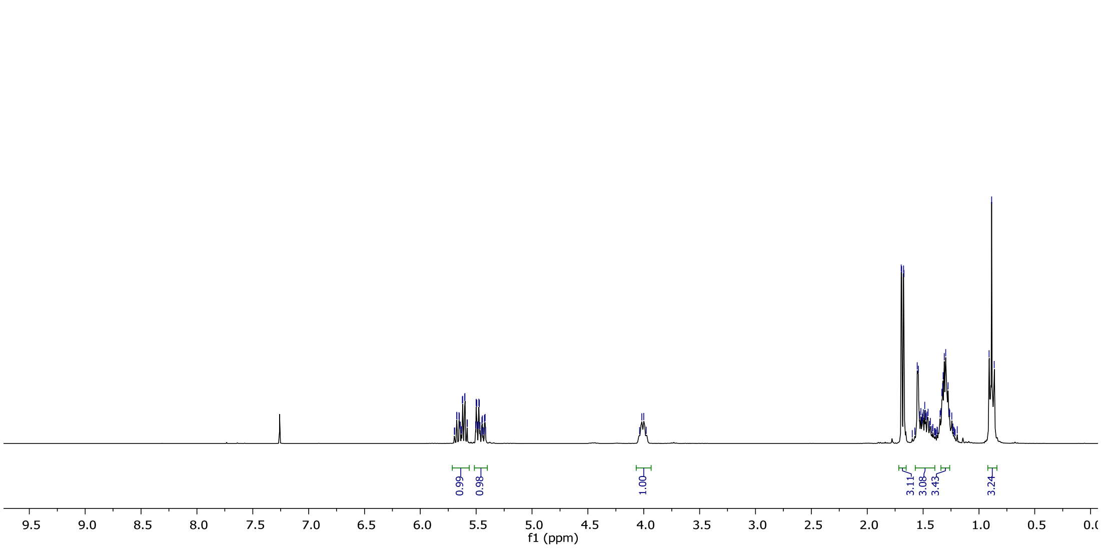

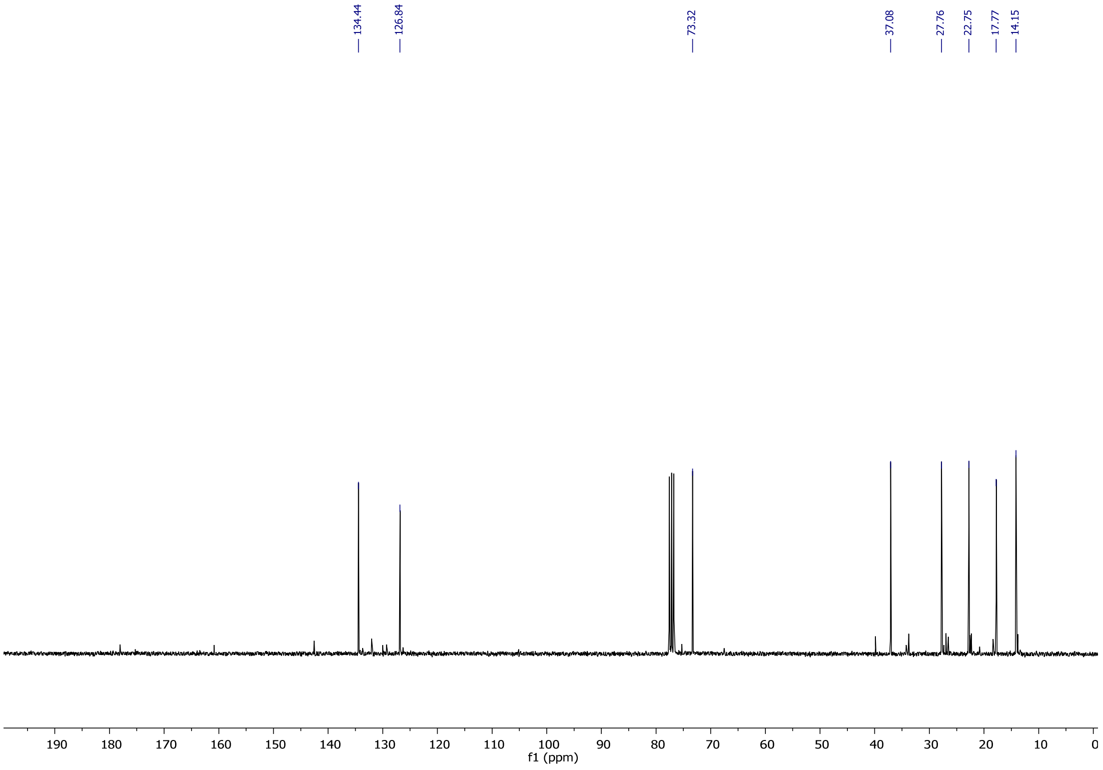

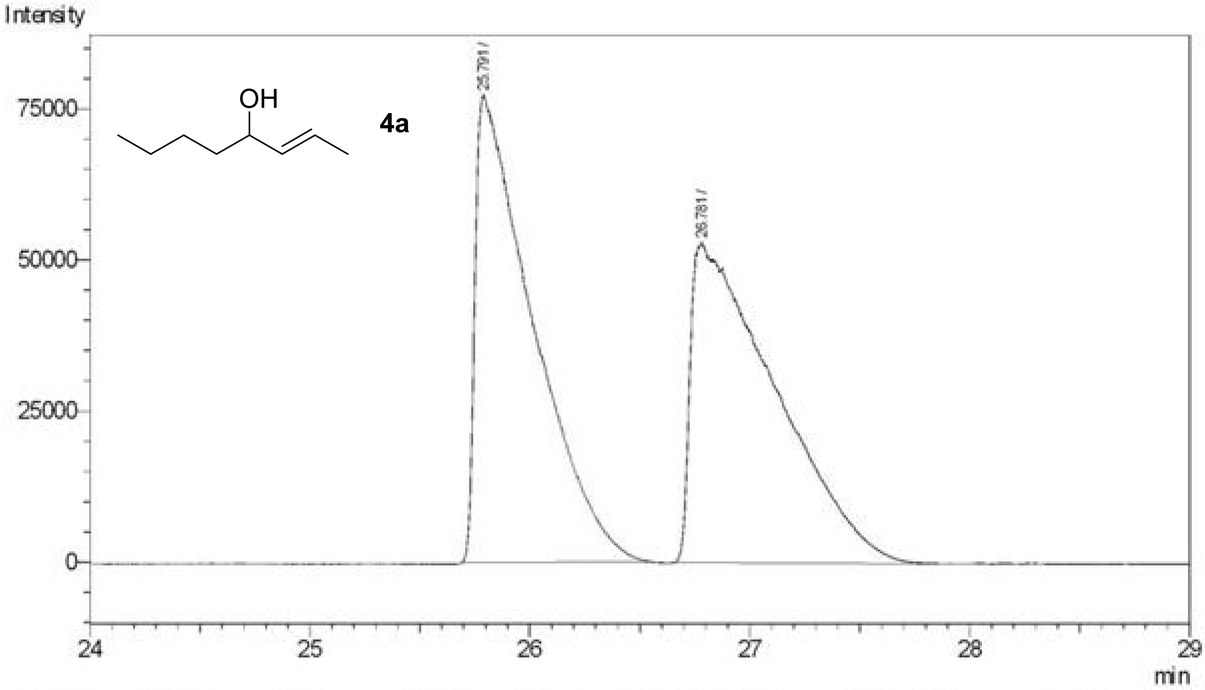

Peak#	Ret.Time	Area	Height	Conc.	Unit	Mark	ID#	Cmpd Name	Area%
1	25.791	1408411	77287	49.951					49.9511
2	26.781	1411169	52911	50.049					50.0489
Total		2819580	130198						100.0000

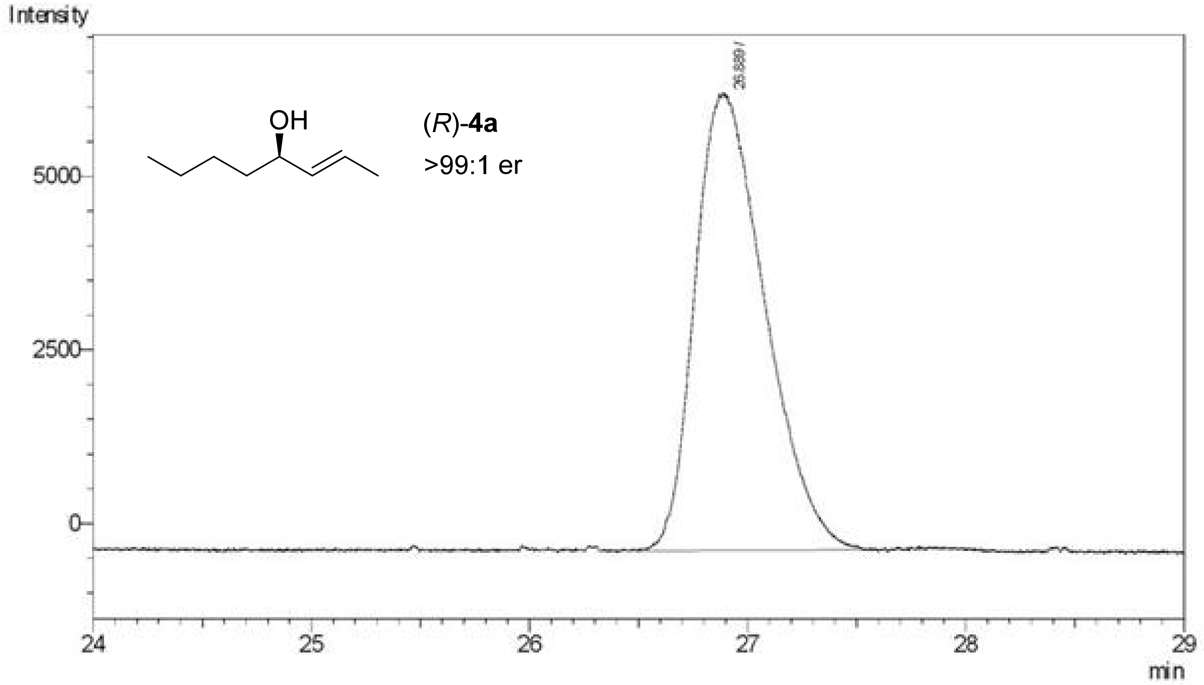

Peak#	Ret.Time	Area	Height	Conc.	Unit	Mark	ID#	Cmpd Name	Area%
1	26.889	147256	6581	100.000					100.0000
Total		147256	6581						100.0000

Oct-2-yn-4-ol

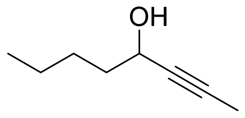

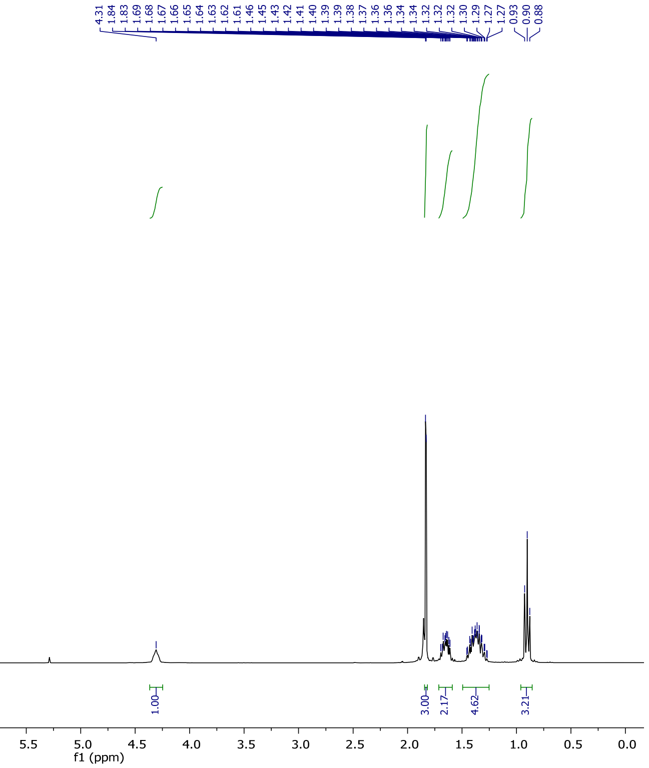

4.31
1.84
1.83
1.69
1.68
1.67
1.66
1.65
1.64
1.63
1.62
1.61
1.46
1.45
1.43
1.42
1.41
1.40
1.39
1.39
1.38
1.37
1.36
1.36
1.34
1.34
1.32
1.32
1.32
1.30
1.29
1.27
1.27
0.93
0.90
0.88
1.00
3.00
2.17
4.62
3.21
9.5
9.0
8.5
8.0
7.5
7.0
6.5
6.0
5.5
5.0
4.5
4.0
3.5
3.0
2.5
2.0
1.5
1.0
0.5
0.0
f1 (ppm)

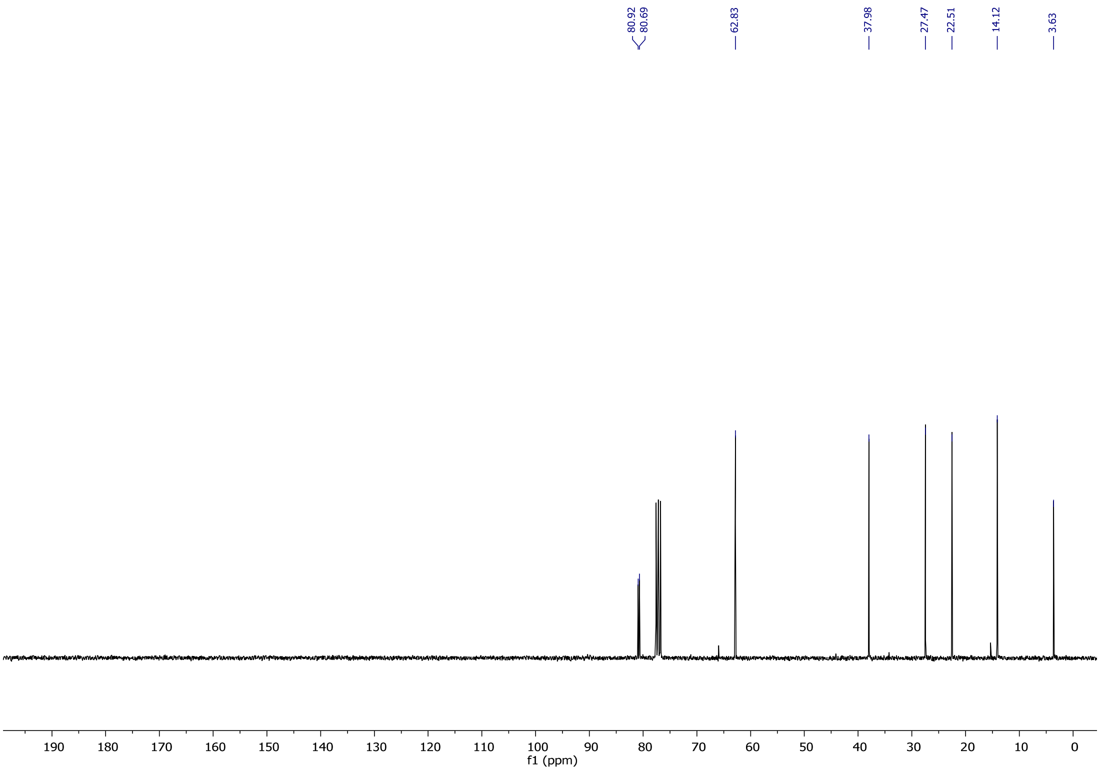

80.92
80.69
62.83
37.98
27.47
22.51
14.12
3.63
190
180
170
160
150
140
130
120
110
100
90
80
70
60
50
40
30
20
10
0
f1 (ppm)

(*R*,*Z*)-Oct-2-en-4-ol (*R*)-4b

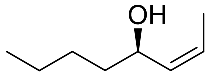

(*R*)-**4b**

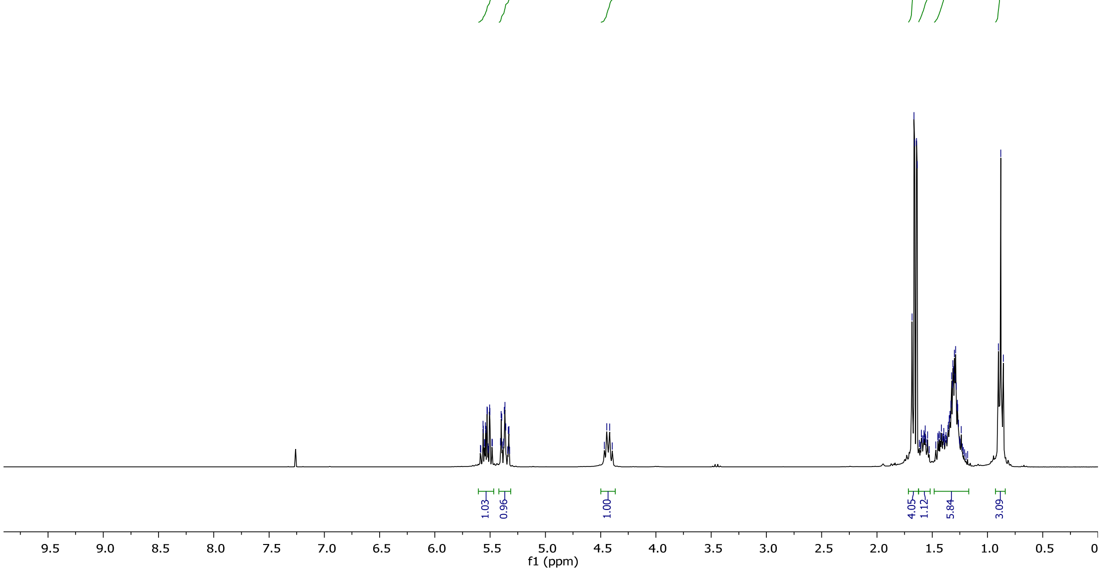

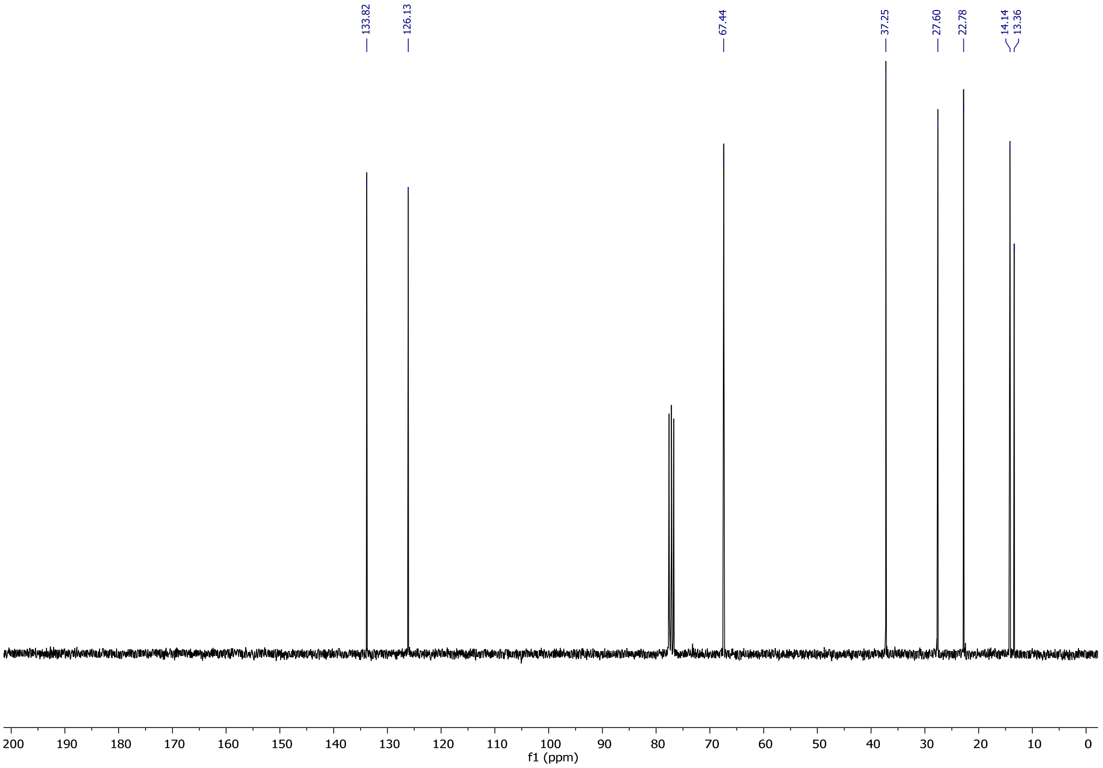

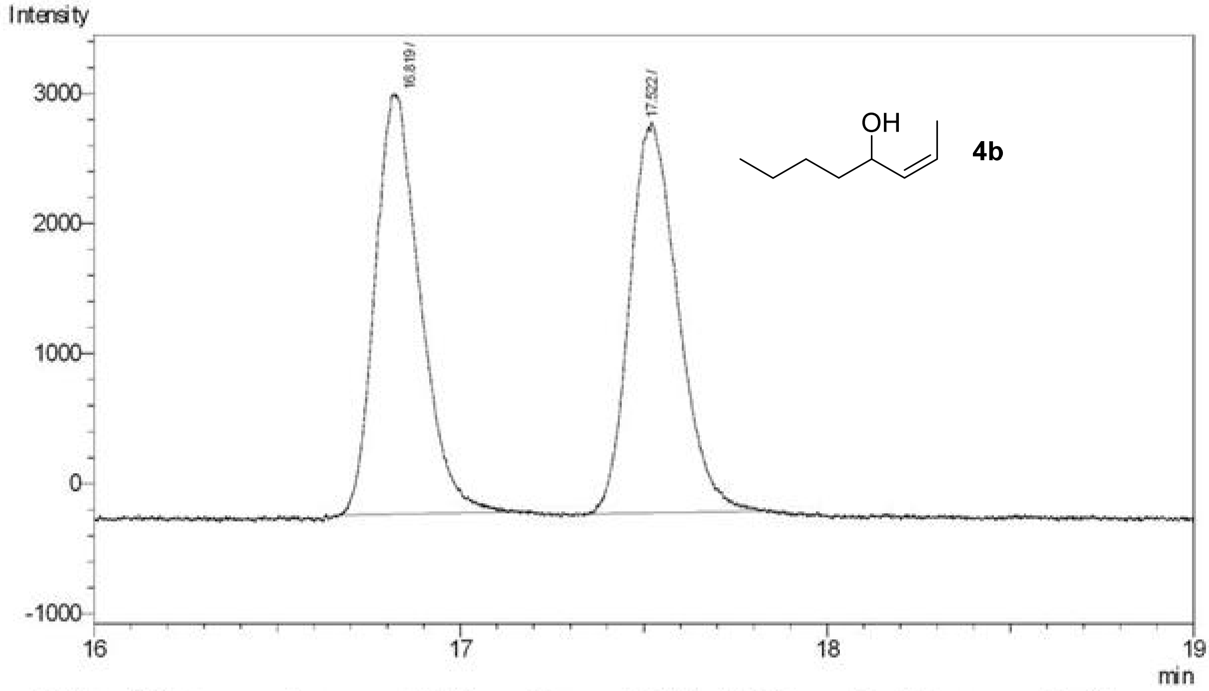

Peak#	Ret.Time	Area	Height	Conc.	Unit	Mark	ID#	Cmpd Name	Area%
1	16.819	27612	3223	50.038					50.0383
2	17.522	27570	2996	49.962					49.9617
Total		55182	6219						100.0000

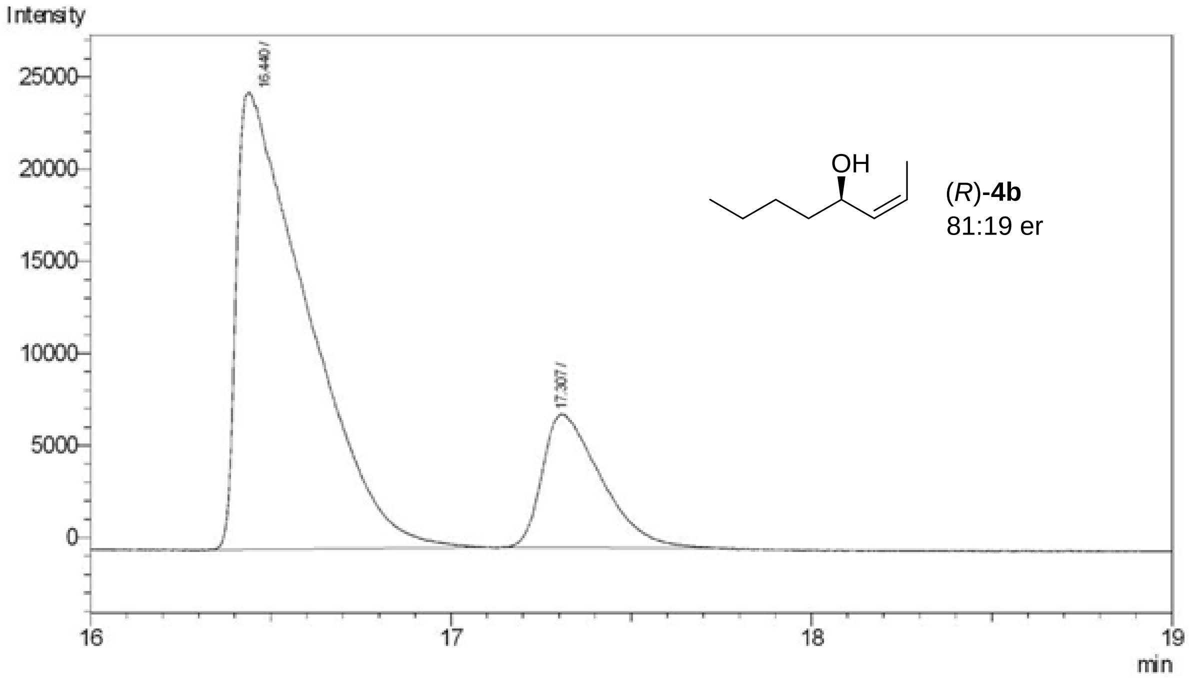

Peak#	Ret.Time	Area	Height	Conc.	Unit	Mark	ID#	Cmpd Name	Area%
1	16.440	337479	24788	80.552					80.5521
2	17.307	81479	7216	19.448					19.4479
Total		418958	32004						100.0000

Oct-3-yn-2-ol

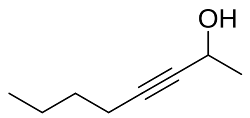

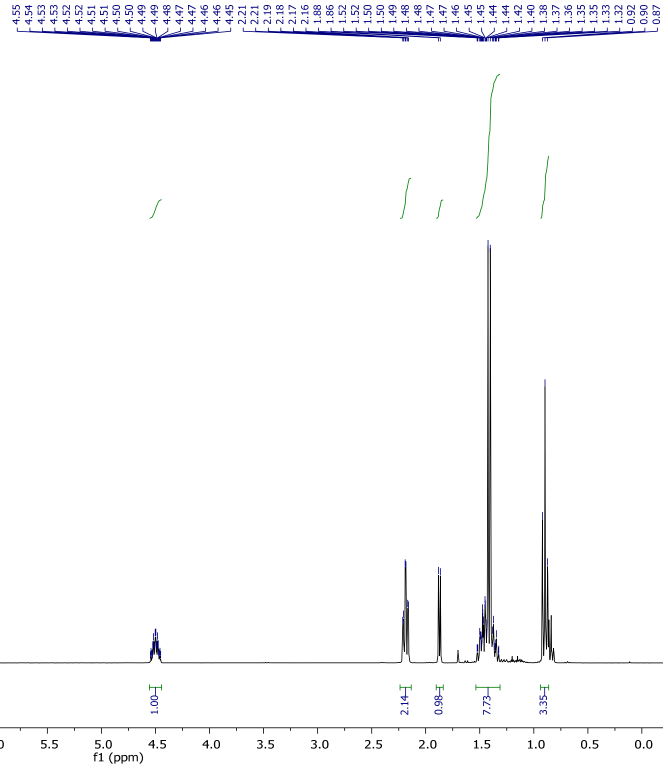

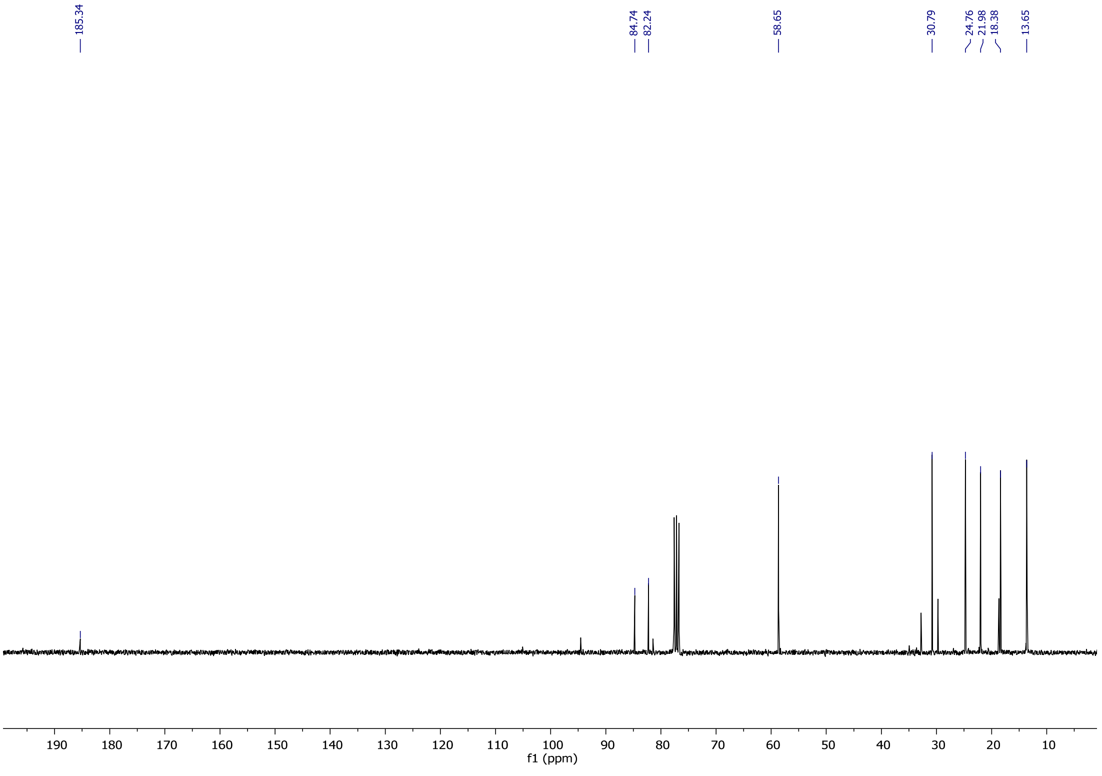

(*R*,*E*)-Oct-3-en-2-ol (*R*)-4c

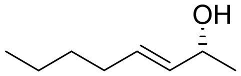

(*R*)-**4c**

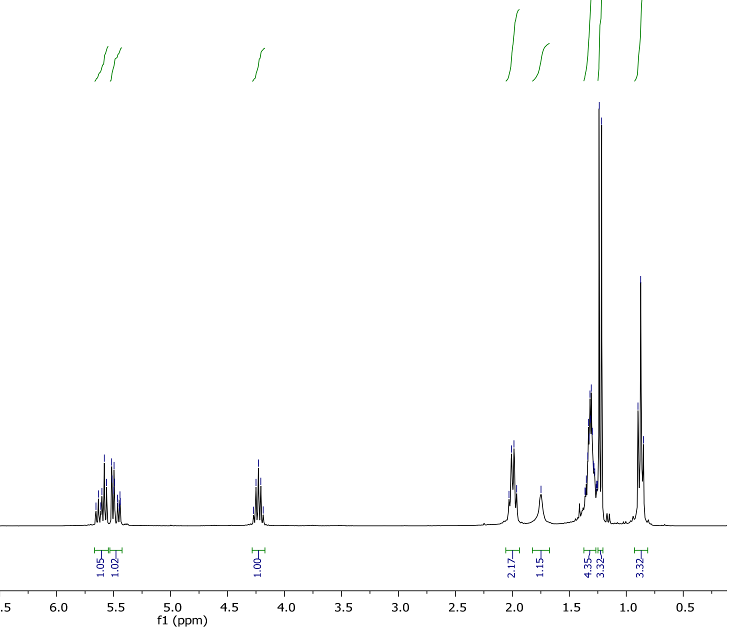

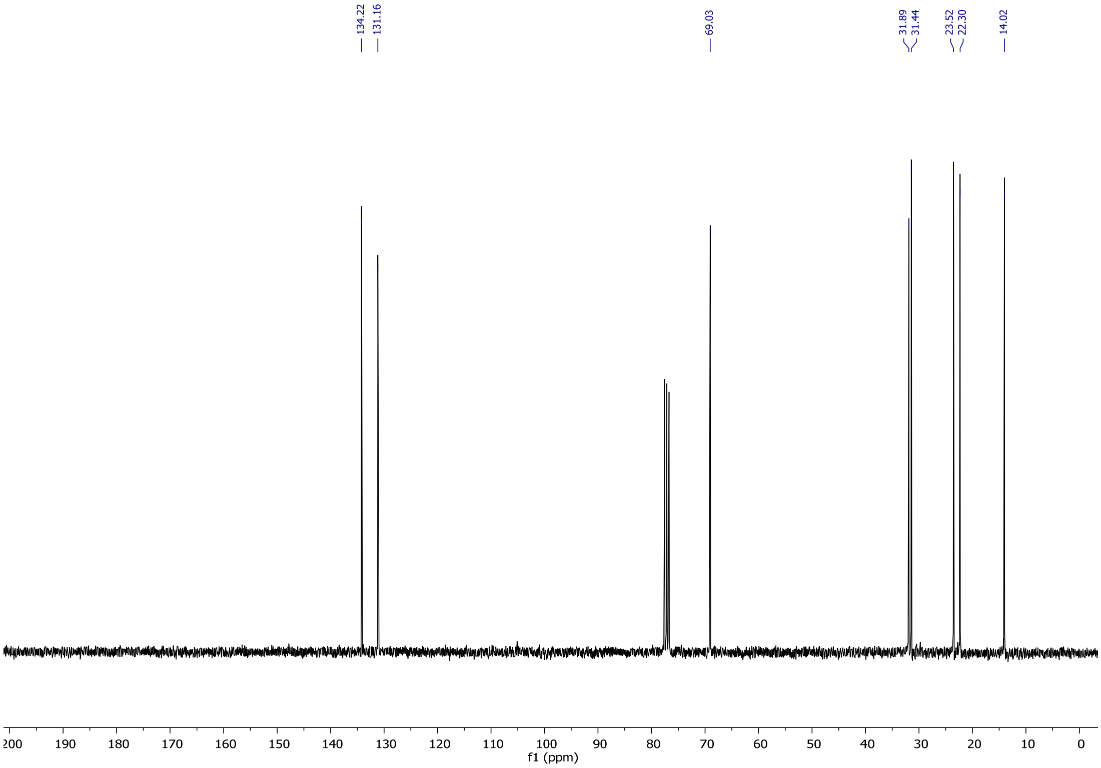

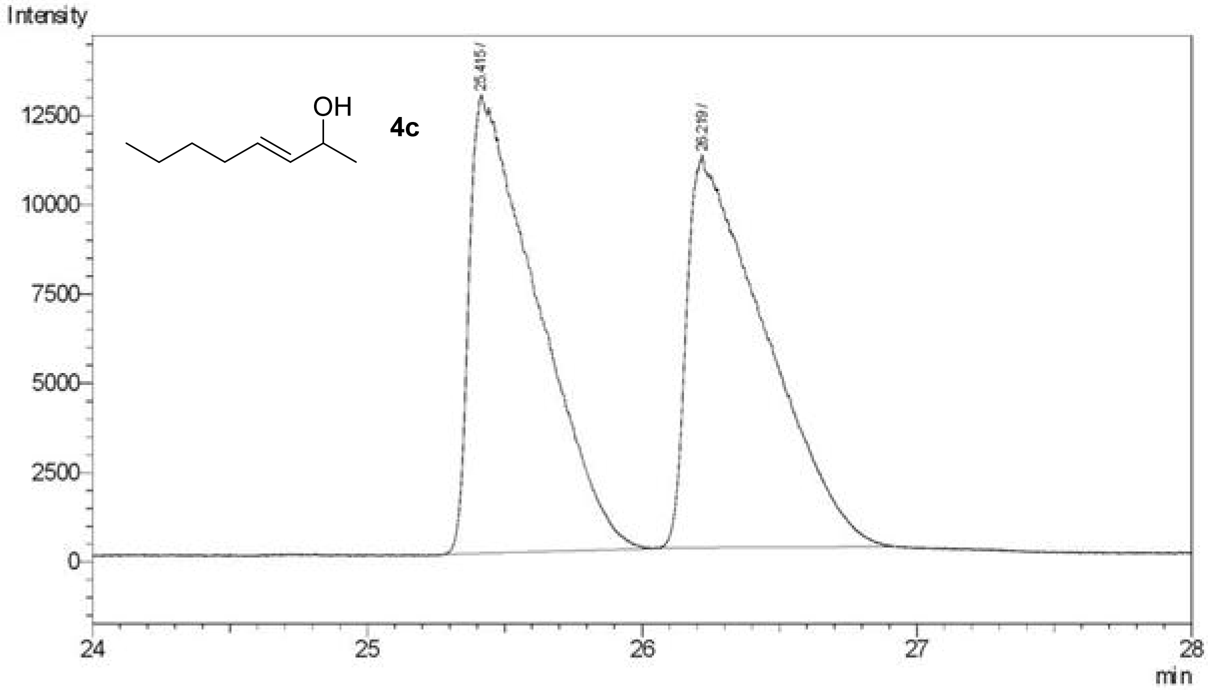

Peak#	Ret.Time	Area	Height	Conc.	Unit	Mark	ID#	Cmpd Name	Area%
1	25.415	220775	12793	50.296					50.2960
2	26.219	218176	10938	49.704					49.7040
Total		438951	23731						100.0000

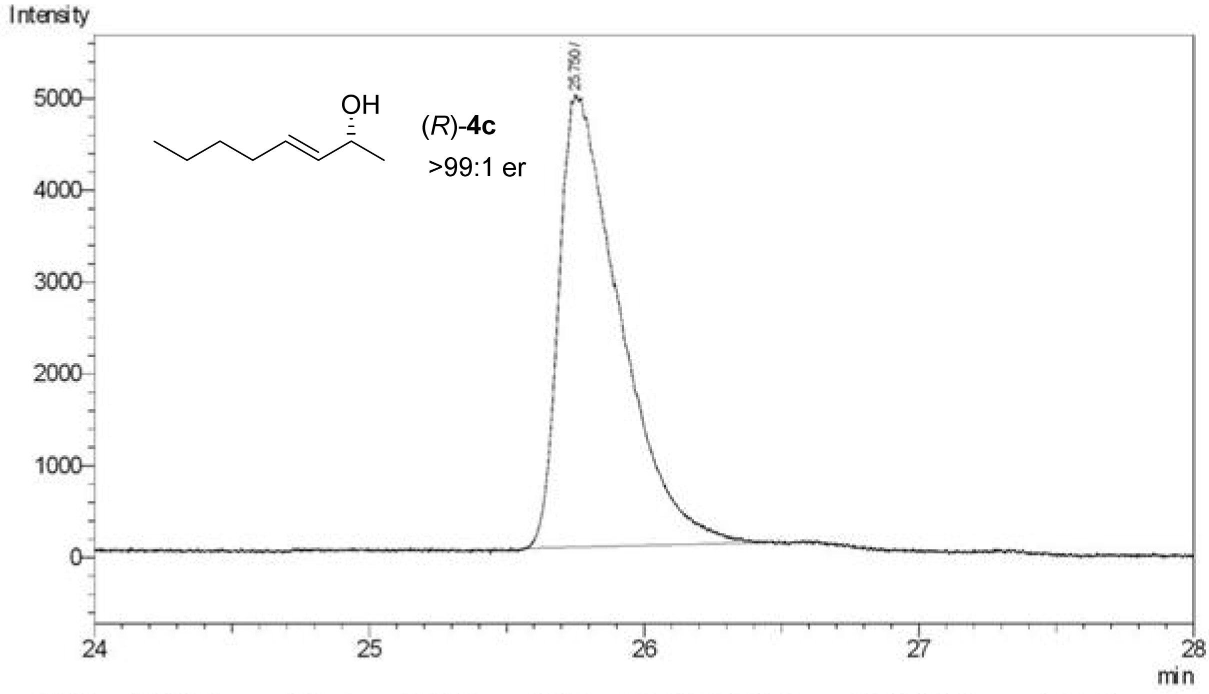

Peak#	Ret.Time	Area	Height	Conc.	Unit	Mark	ID#	Cmpd Name	Area%
1	25.750	76227	4911	100.000					100.0000
Total		76227	4911						100.0000

(*R*, *E*)-1-Cyclohexylbut-2-en-1-ol (*R*)-4d

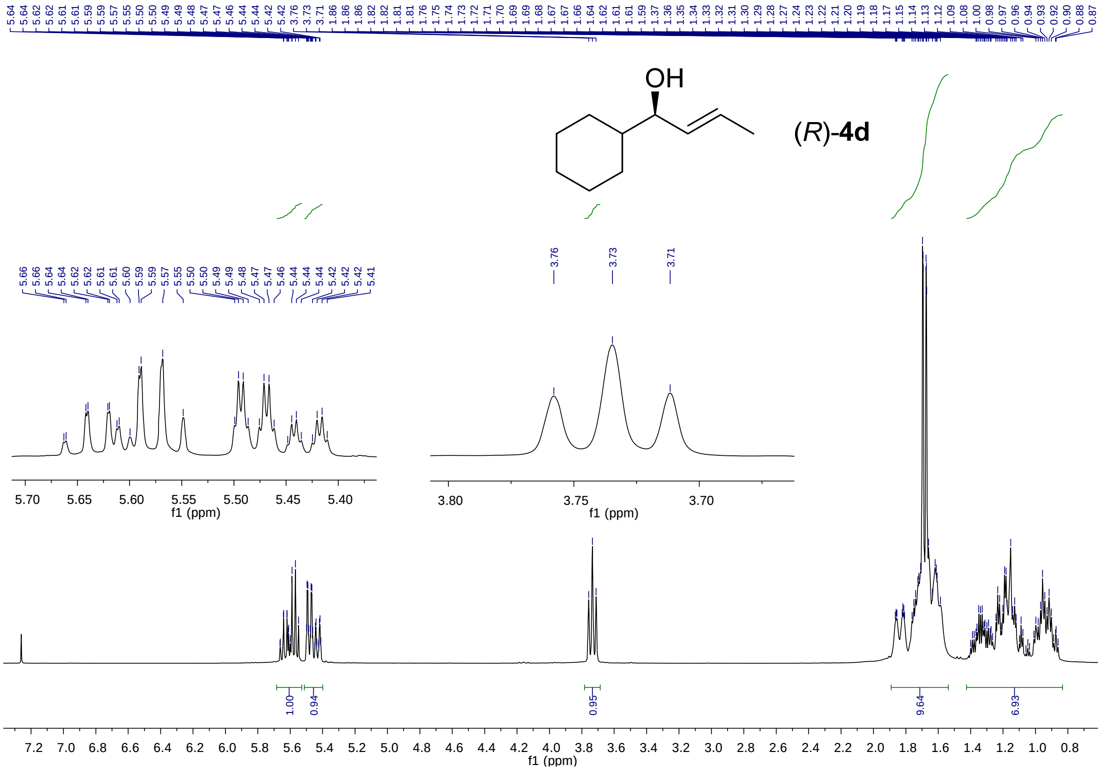

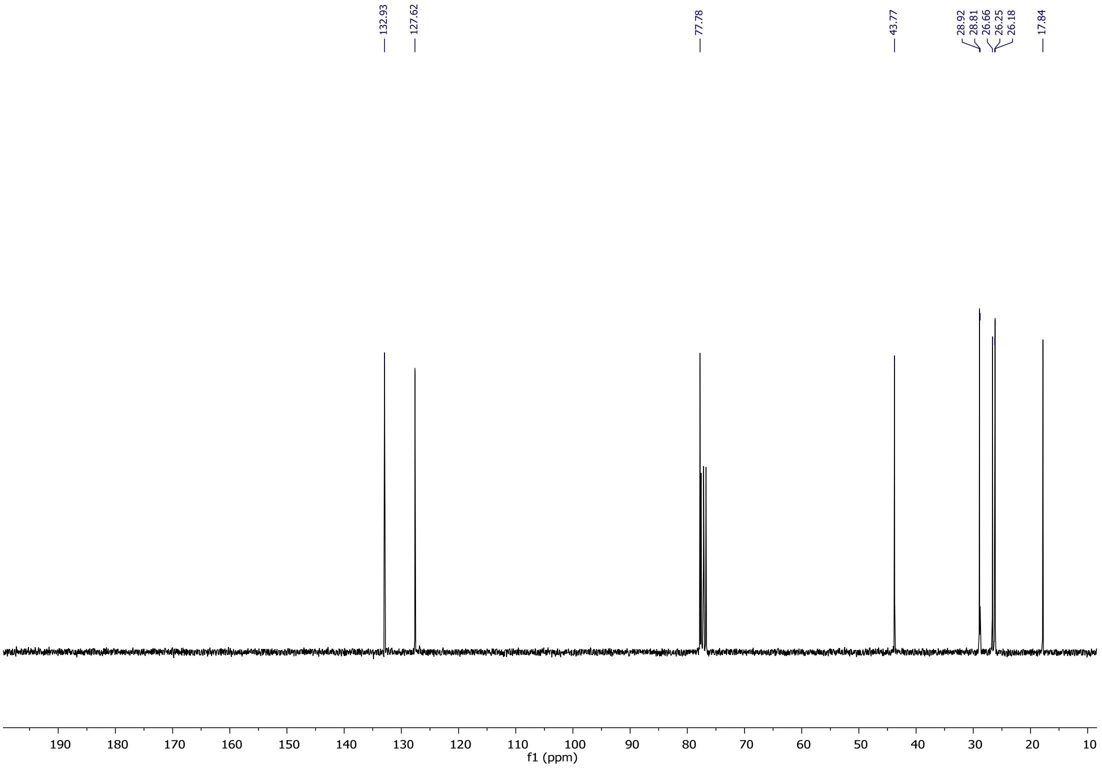

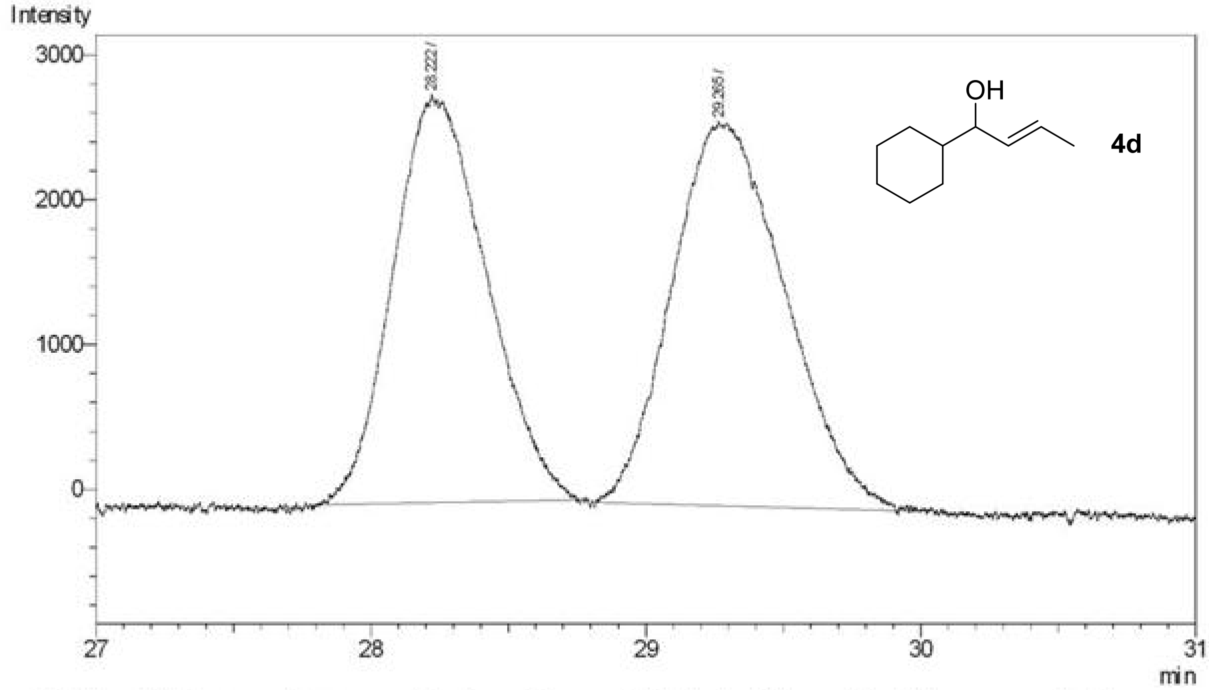

Peak#	Ret.Time	Area	Height	Conc.	Unit	Mark	ID#	Cmpd Name	Area%
1	28.222	66576	2801	46.778					46.7778
2	29.265	75748	2647	53.222					53.2222
Total		142324	5448						100.0000

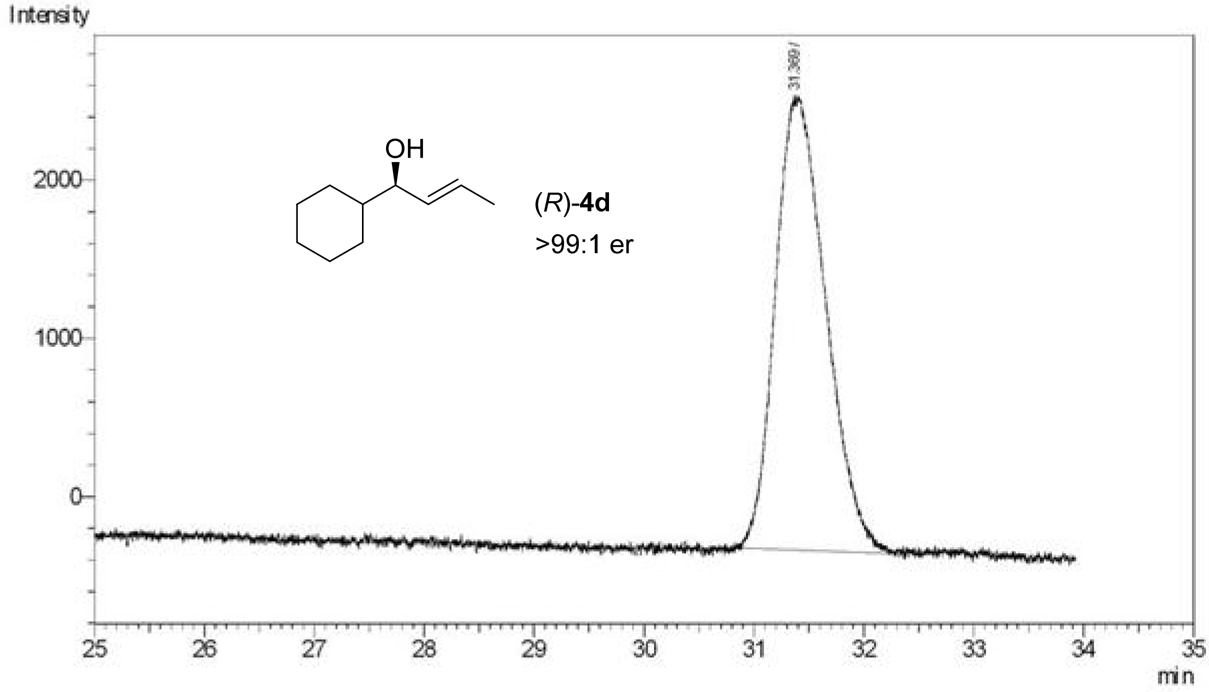

Peak#	Ret.Time	Area	Height	Conc.	Unit	Mark	ID#	Cmpd Name	Area%
1	31.369	91372	2871	100.000					100.0000
Total		91372	2871						100.0000

4-Cyclohexylbut-3-yn-2-ol

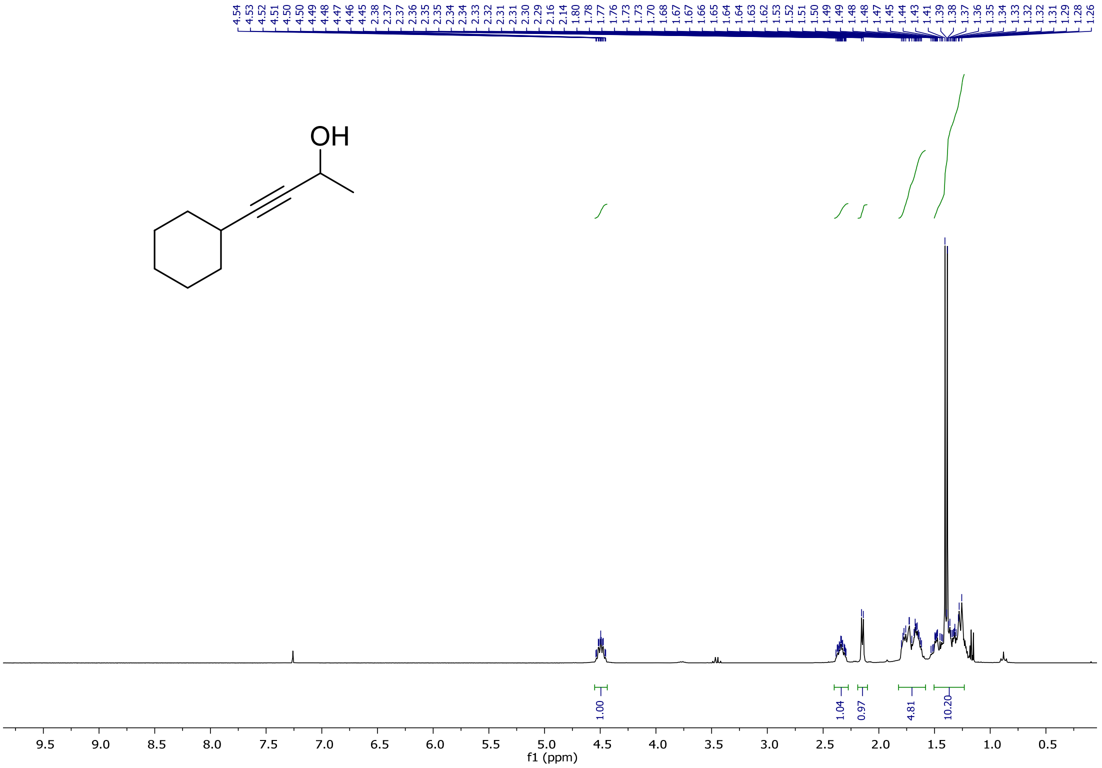

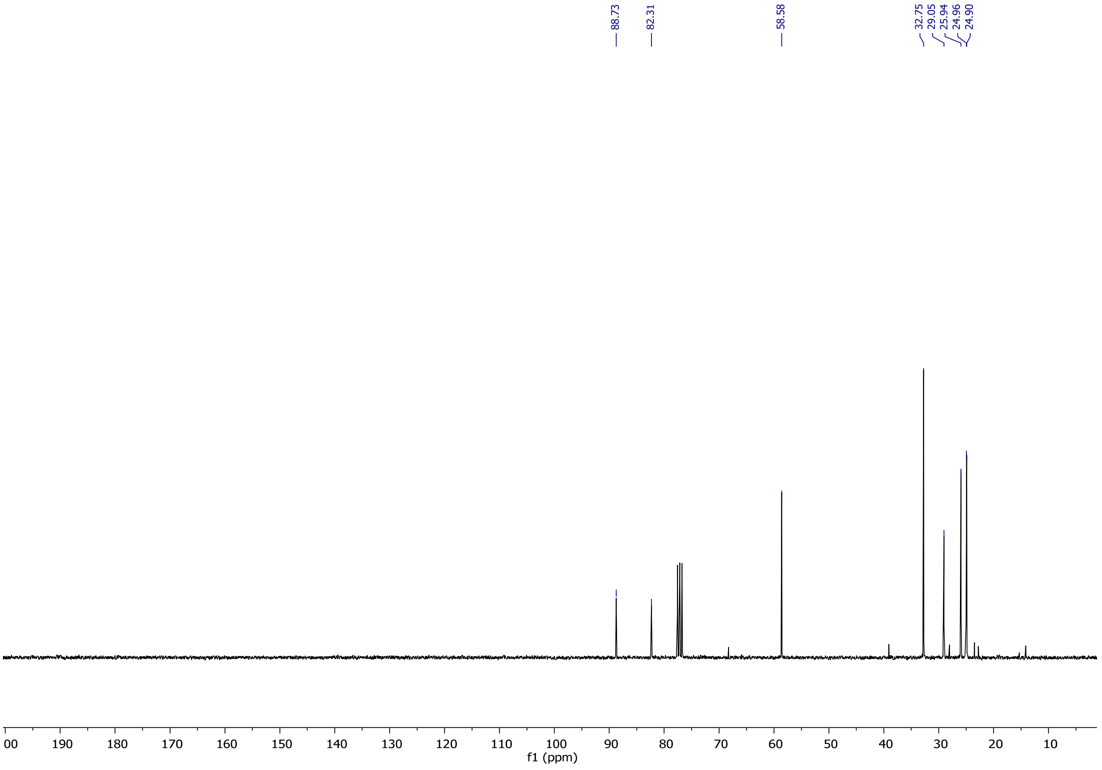

(*R*, *E*)-4-Cyclohexylbut-3-en-2-ol (*R*)-4e

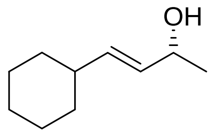

(*R*)-**4e**

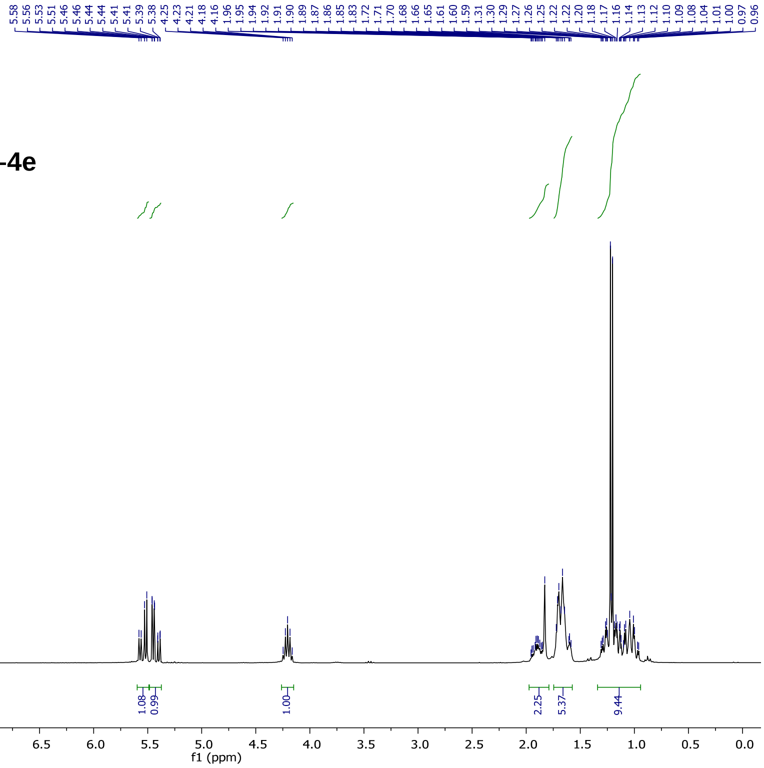

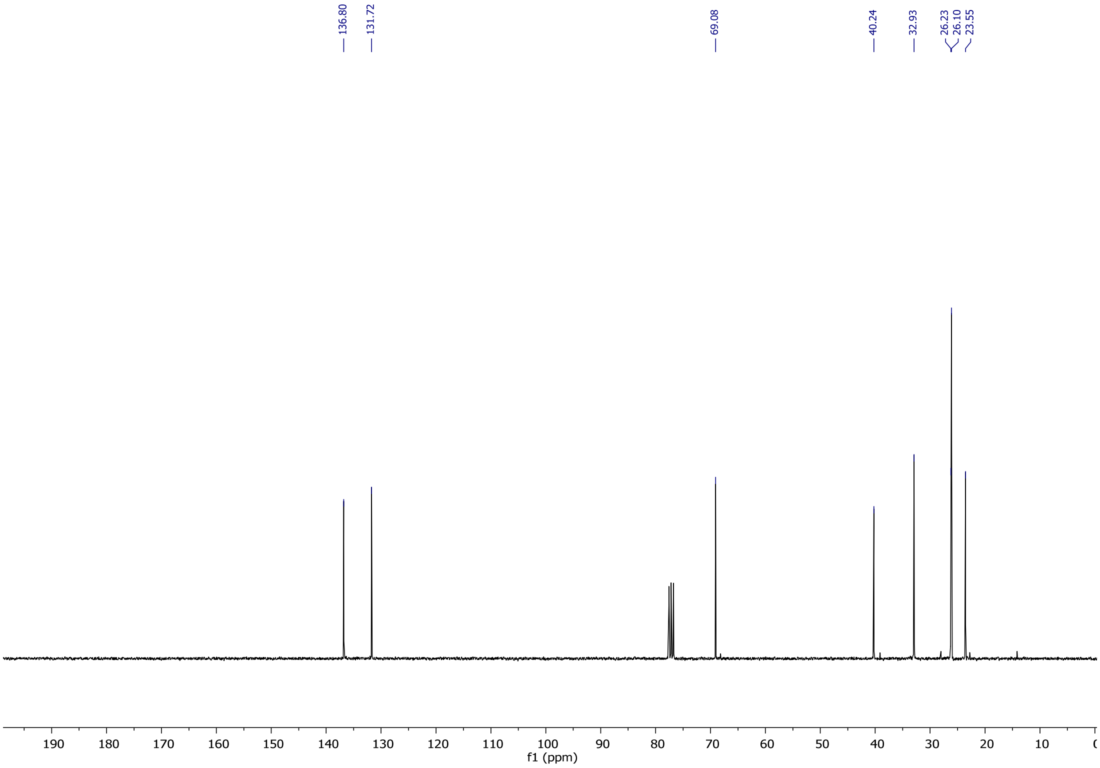

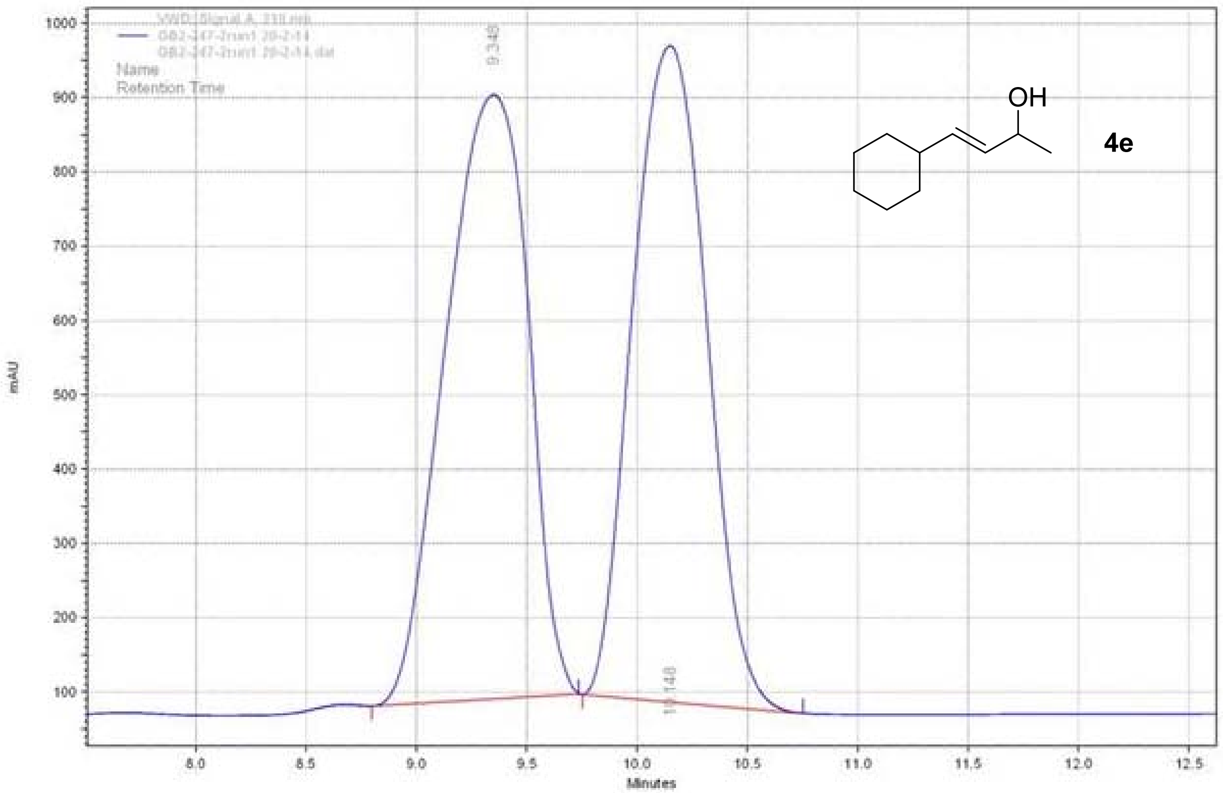

C:\EZChrom Elite\Enterprise\Projects\Default\Data\GB2-247-2run1 20-2-14.dat, VWD: Signal A, 210 nm

VWD: Signal A, 210 nm Results

Retention Time	Area	Area %	Height	Height %
9.348	354883948	50.61	13636734	47.93
10.148	346337457	49.39	14815560	52.07

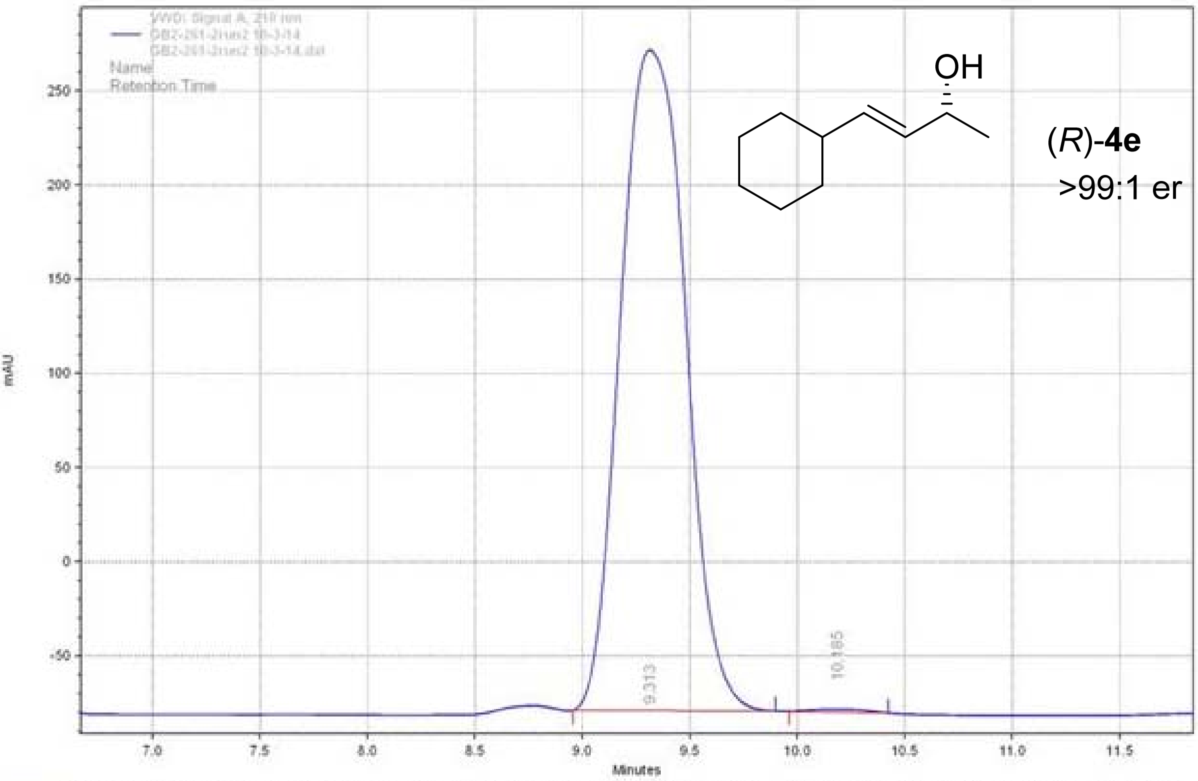

C:\EZChrom Elite\Enterprise\Projects\Default\Data\GB2-261-2run2 10-3-14.dat, VWD: Signal A, 210 nm

VWD: Signal A, 210 nm Results

Retention Time	Area	Area %	Height	Height %
9.313	124415322	99.66	5883136	99.60
10.185	429154	0.34	23830	0.40

(*R*, *E*)-5-Methylhex-3-en-2-ol (*R*)-4f

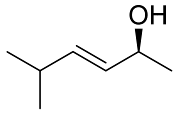

(*R*)-**4f**

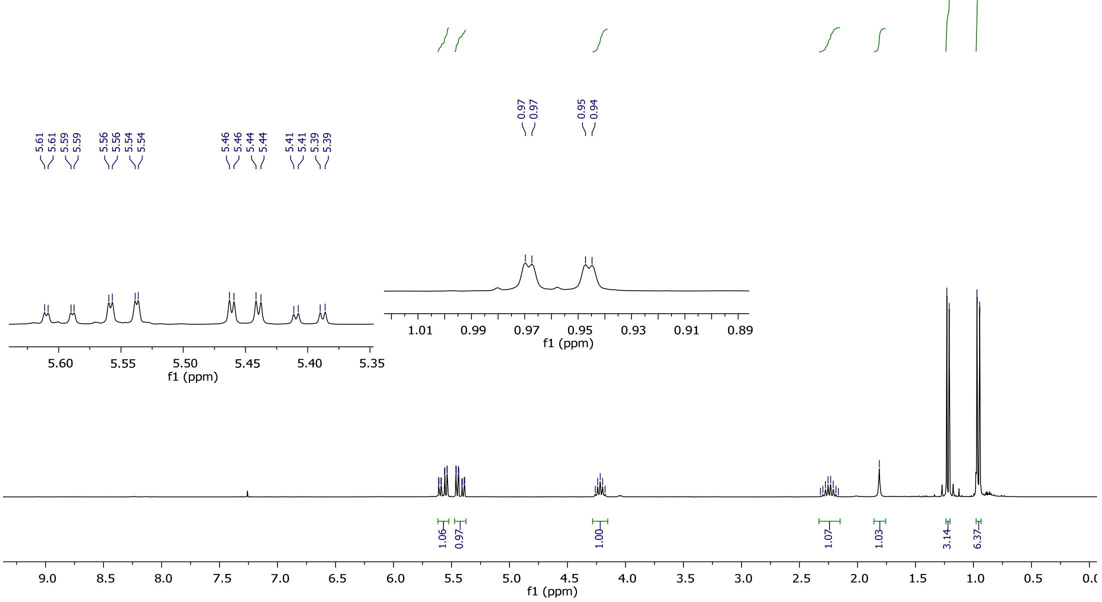

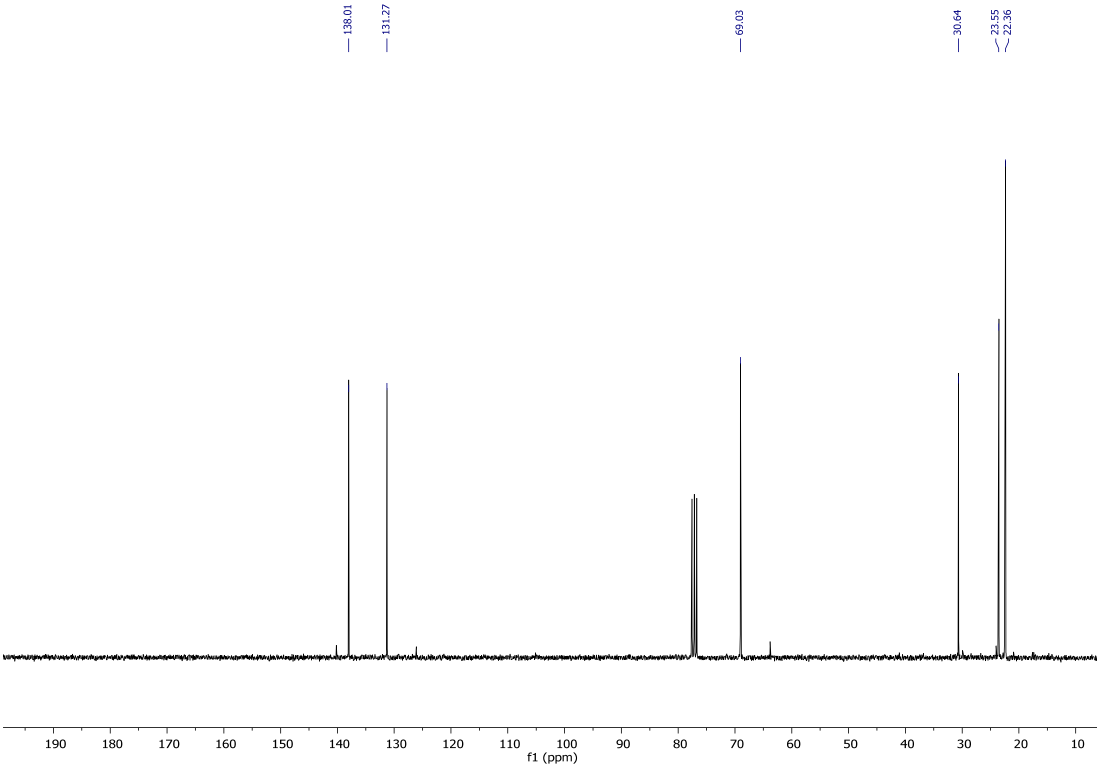

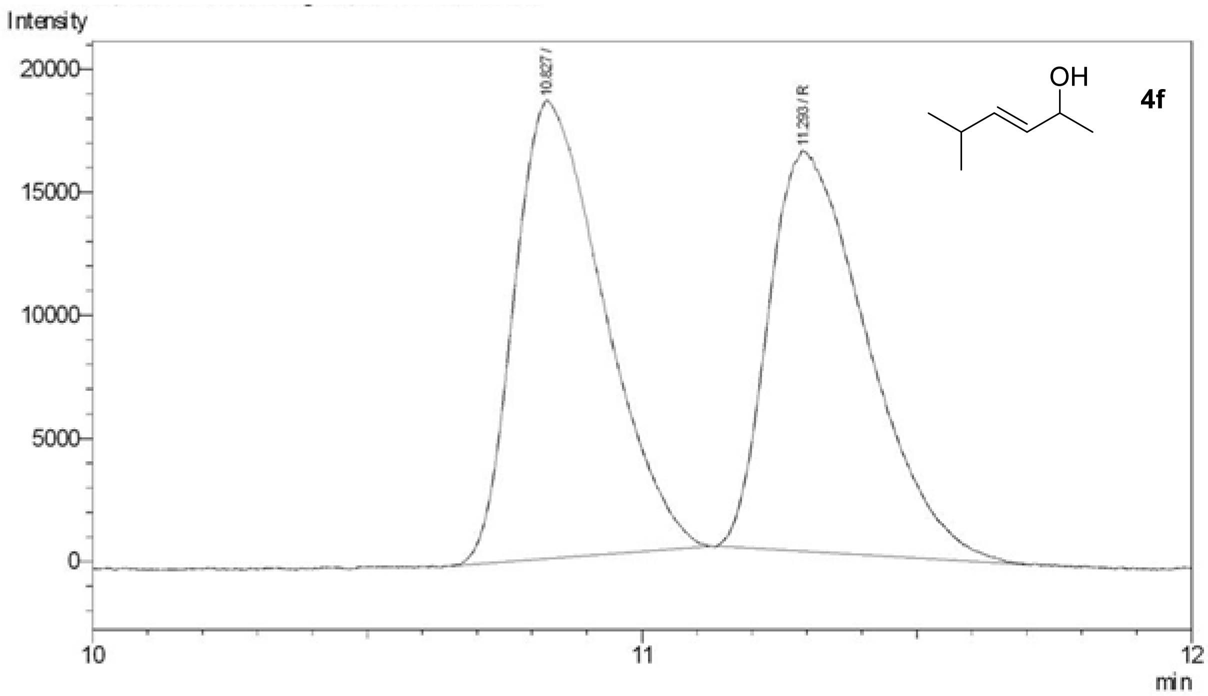

Peak#	Ret.Time	Area	Height	Conc.	Unit	Mark	ID#	Cmpd Name	Area%
1	10.827	210437	18611	0.000					50.8565
2	11.293	203348	16257	0.000	ppm		1	R	49.1435
Total		413785	34868						100.0000

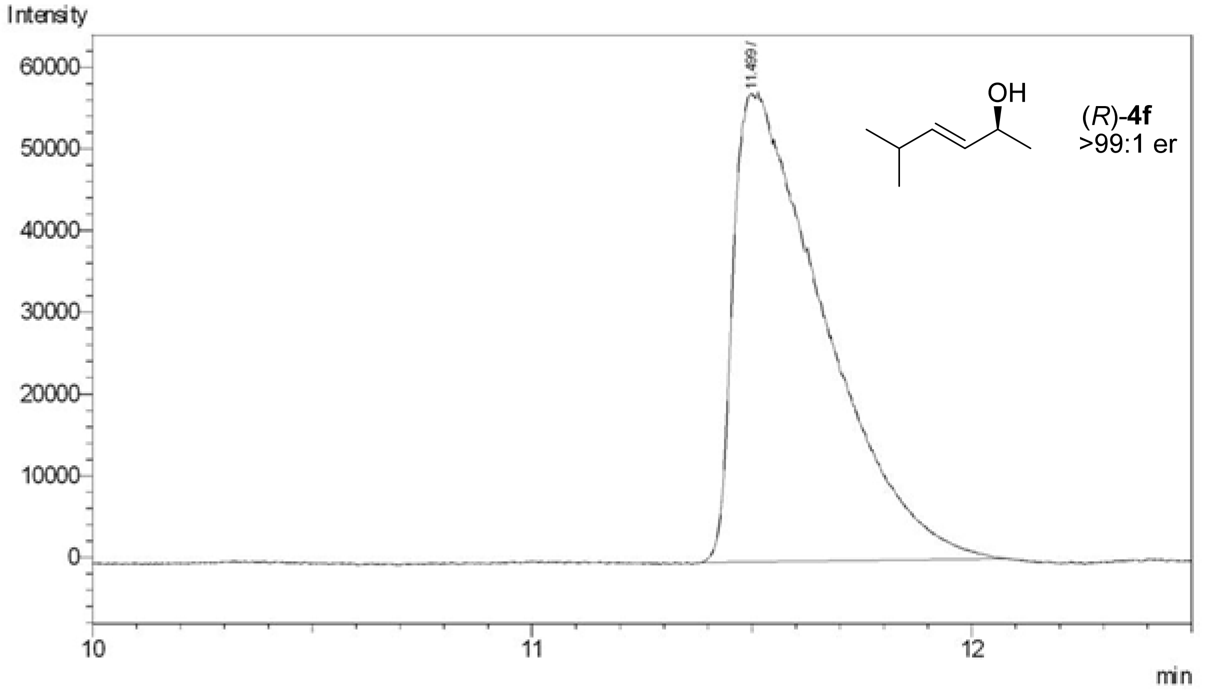

Peak#	Ret.Time	Area	Height	Conc.	Unit	Mark	ID#	Cmpd Name	Area%
1	11.499	824536	57262	100.000					100.0000
Total		824536	57262						100.0000

(*R*, *E*)-2-Phenylpent-3-en-2-ol (*R*)-4g

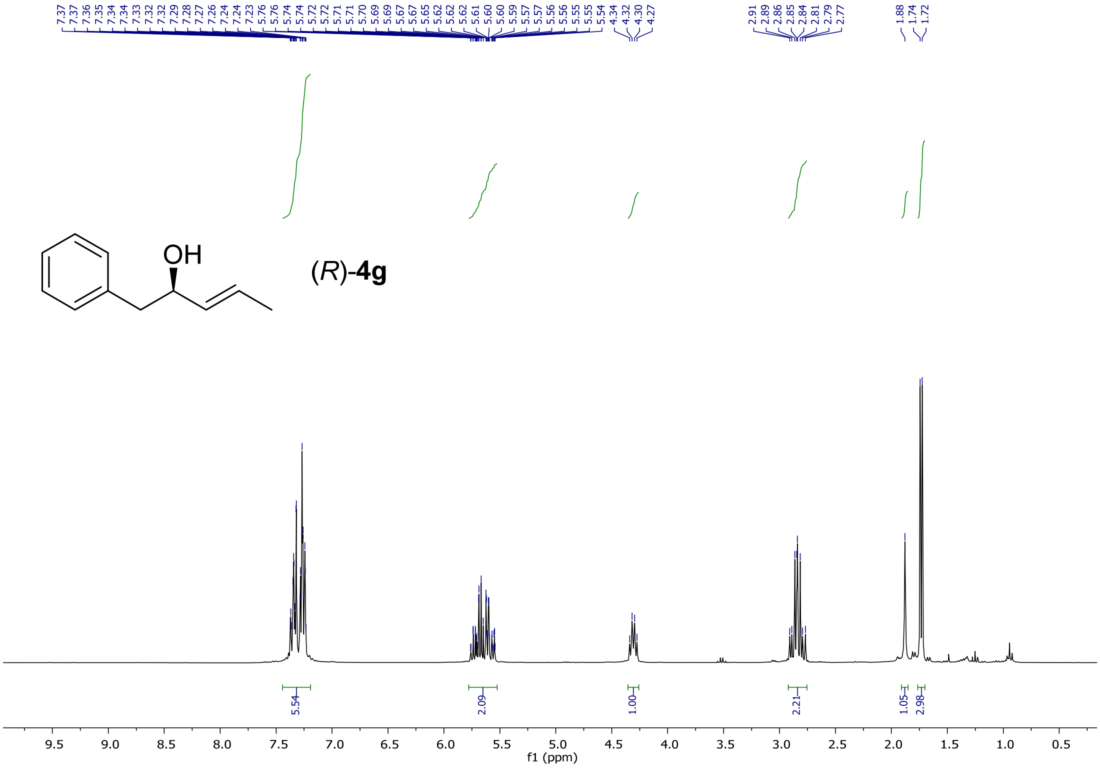

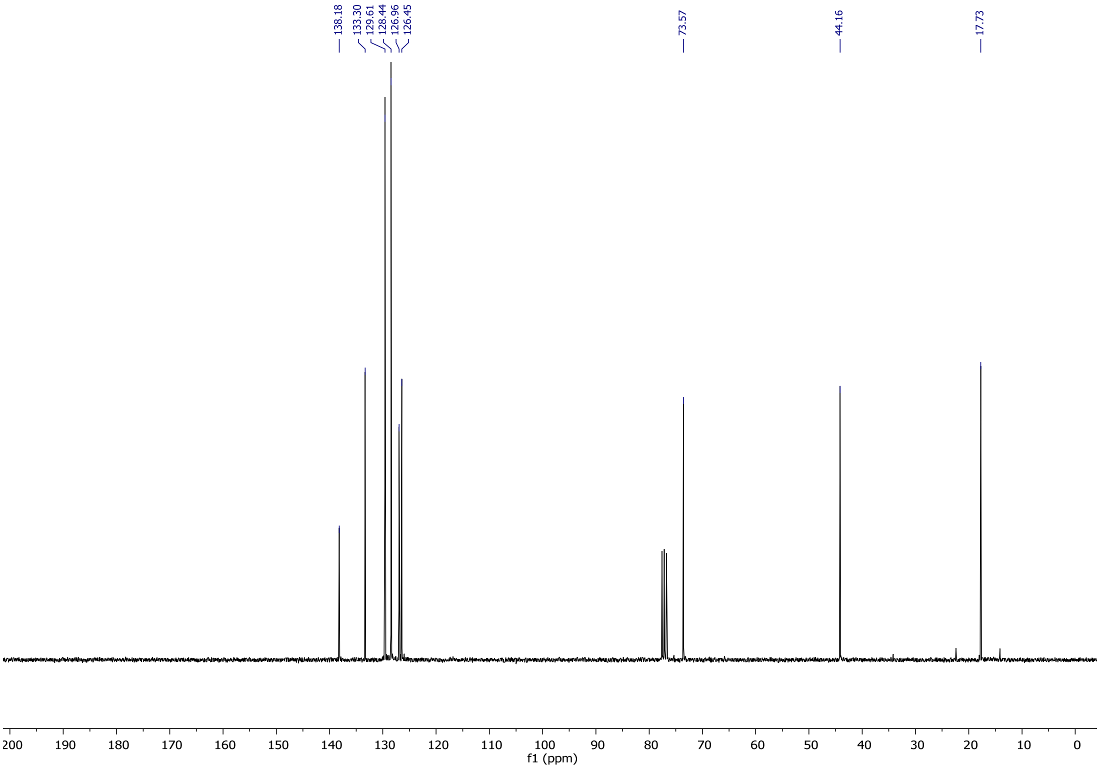

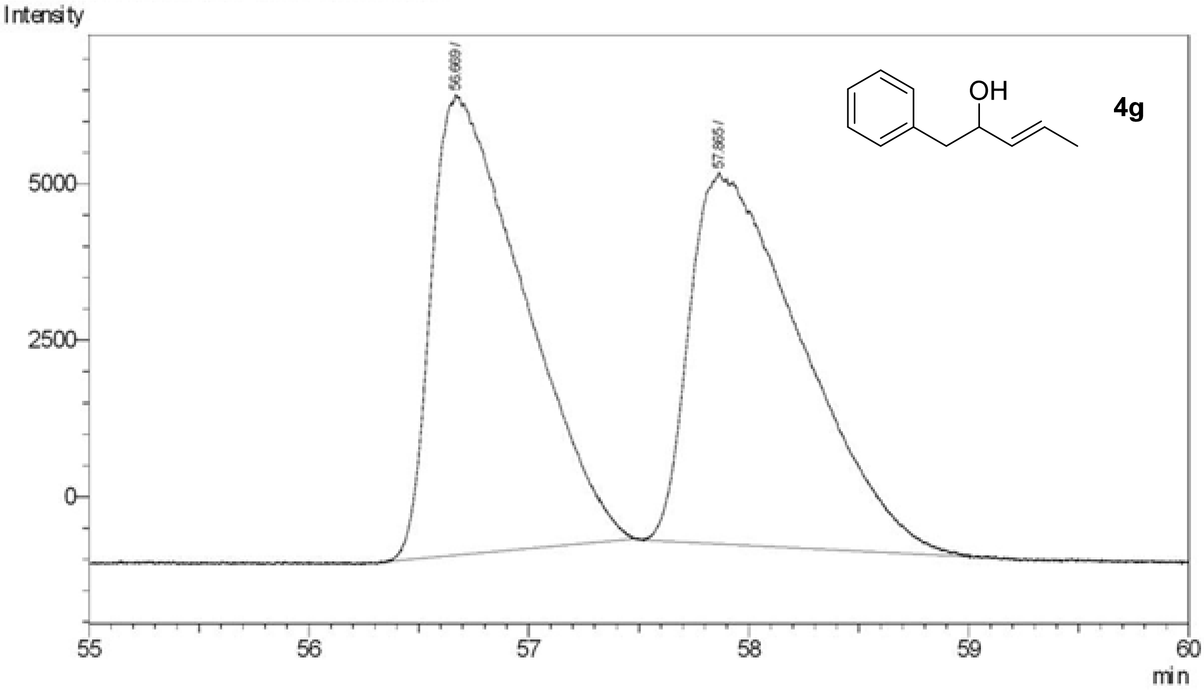

Peak#	Ret.Time	Area	Height	Conc.	Unit	Mark	ID#	Cmpd Name	Area%
1	56.669	215007	7343	50.334					50.3345
2	57.865	212150	5930	49.666					49.6655
Total		427157	13273						100.0000

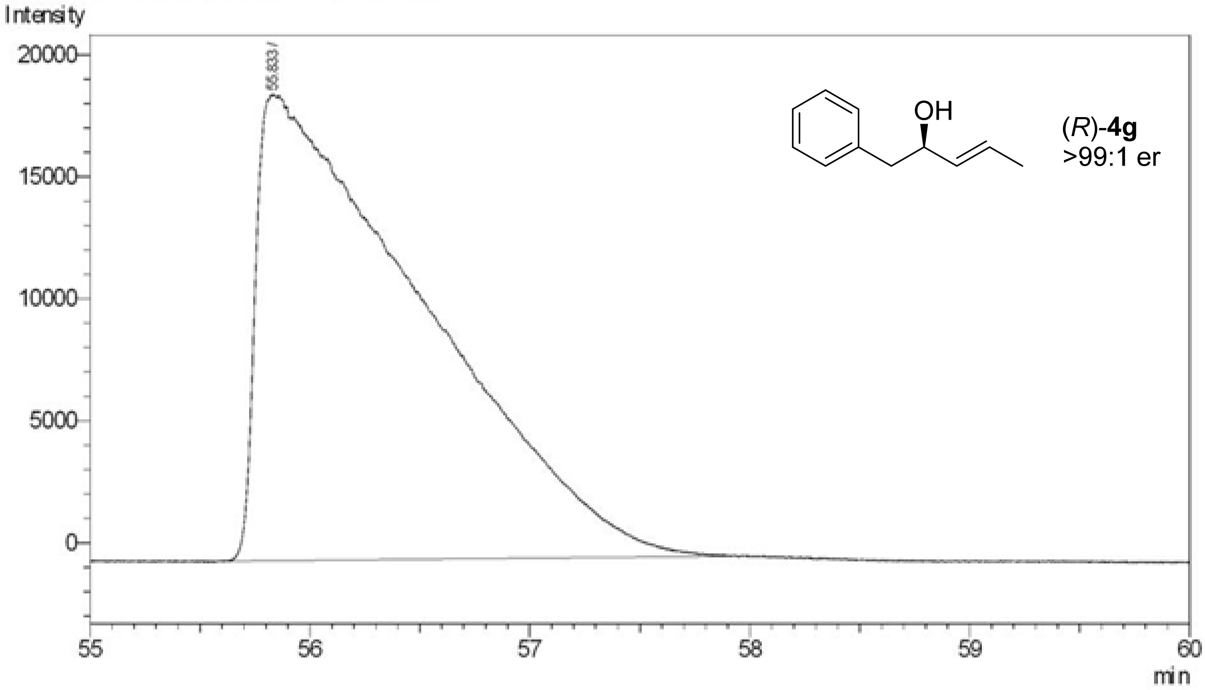

Peak#	Ret.Time	Area	Height	Conc.	Unit	Mark	ID#	Cmpd Name	Area%
1	55.833	1010271	19096	100.000					100.0000
Total		1010271	19096						100.0000

(*R*, *E*)-1-phenylbut-2-en-1-ol (*R*)-4h

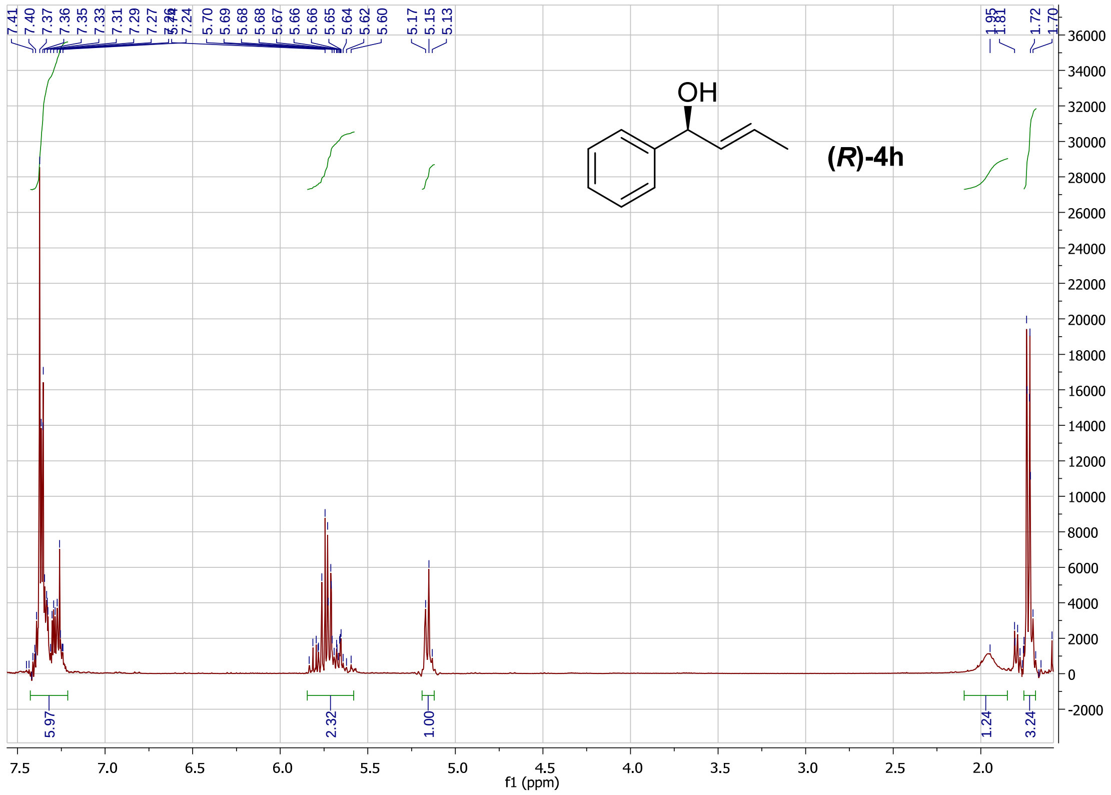

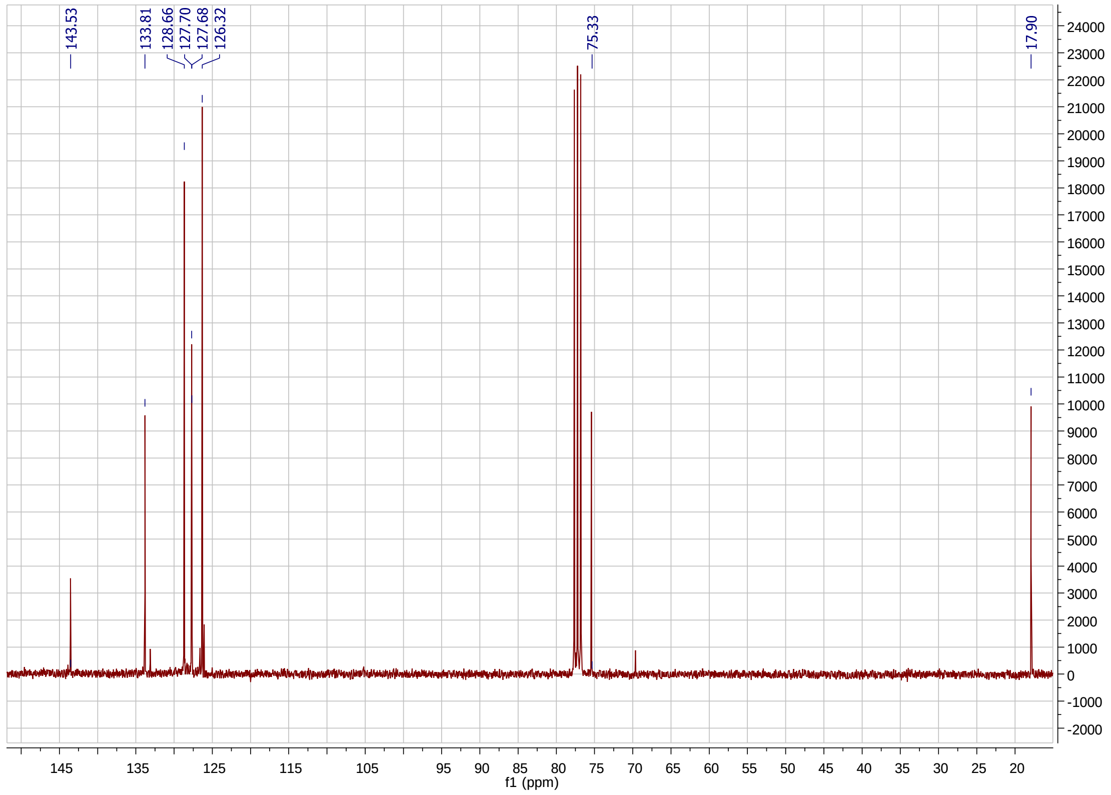

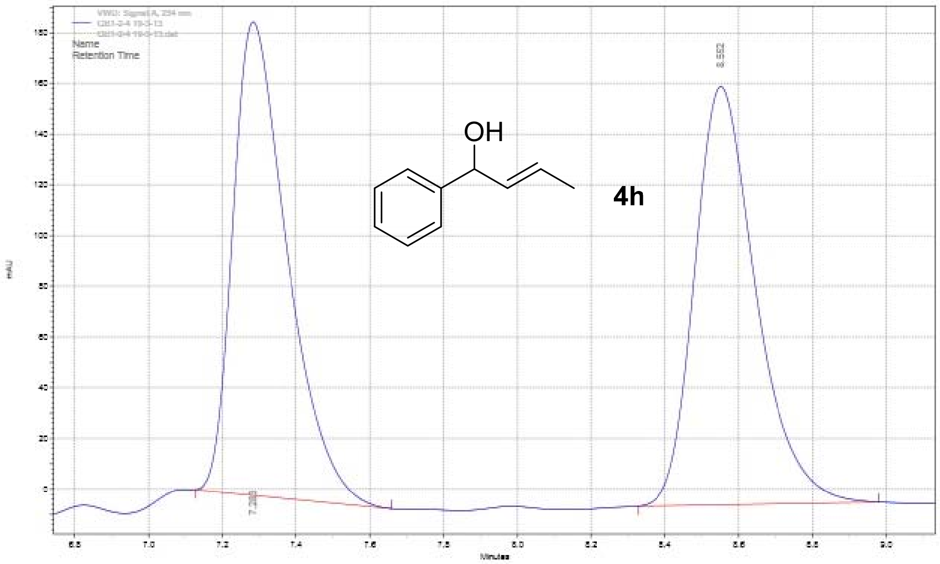

C:\EZChrom Elite\Enterprise\Projects\Default\Data\GB1-2-4 19-3-13.dat, VWD: Signal A, 254 nm

VWD: Signal A,
254 nm Results

Retention Time	Area	Area %	Height	Height %
7.283	32154684	50.20	3133012	53.10
8.552	31893933	49.80	2766815	46.90

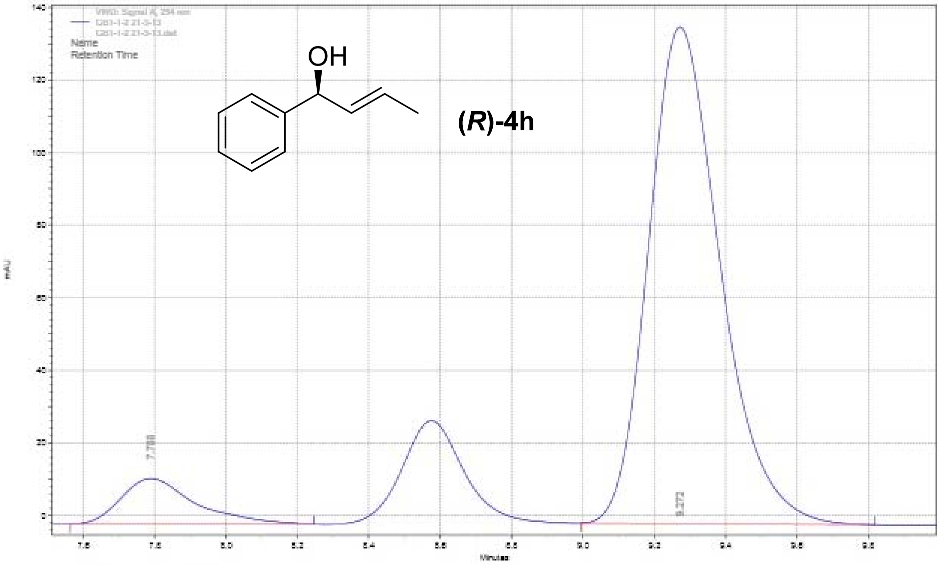

VWD: Signal A,
254 nm Results

Retention Time	Area	Area %	Height	Height %
7.788	3089909	8.77	212011	8.45
9.272	32154713	91.23	2298004	91.55

(*S*, *E*)-4-Phenylbut-3-en-2-ol (*S*)-4i

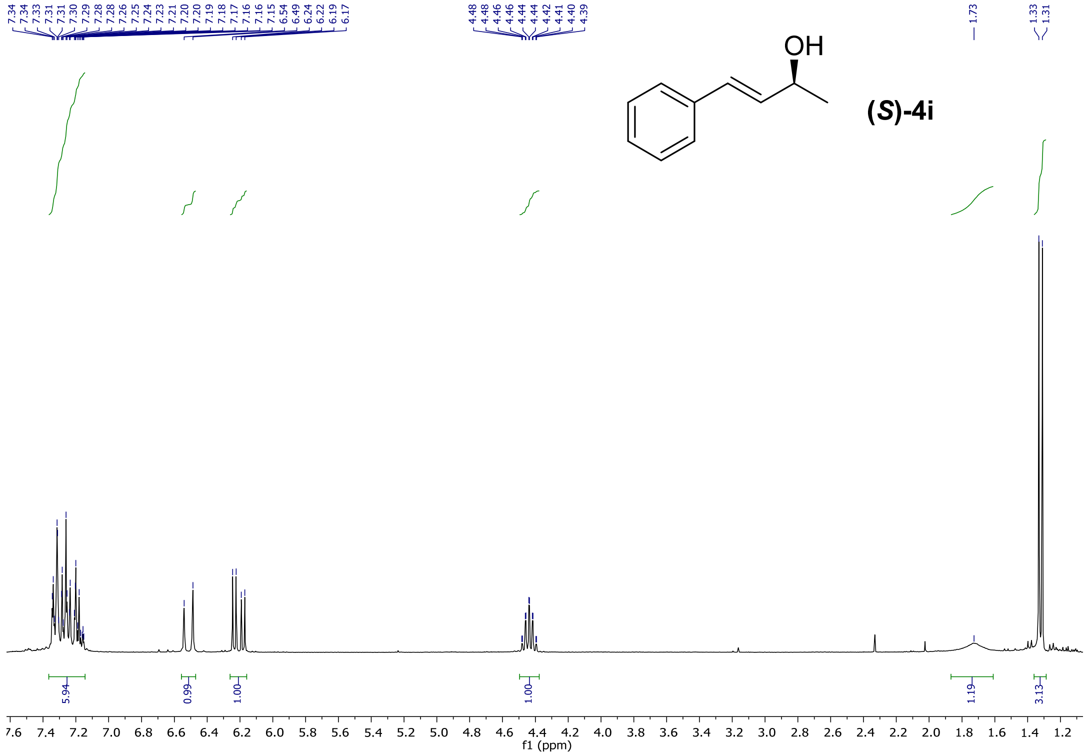

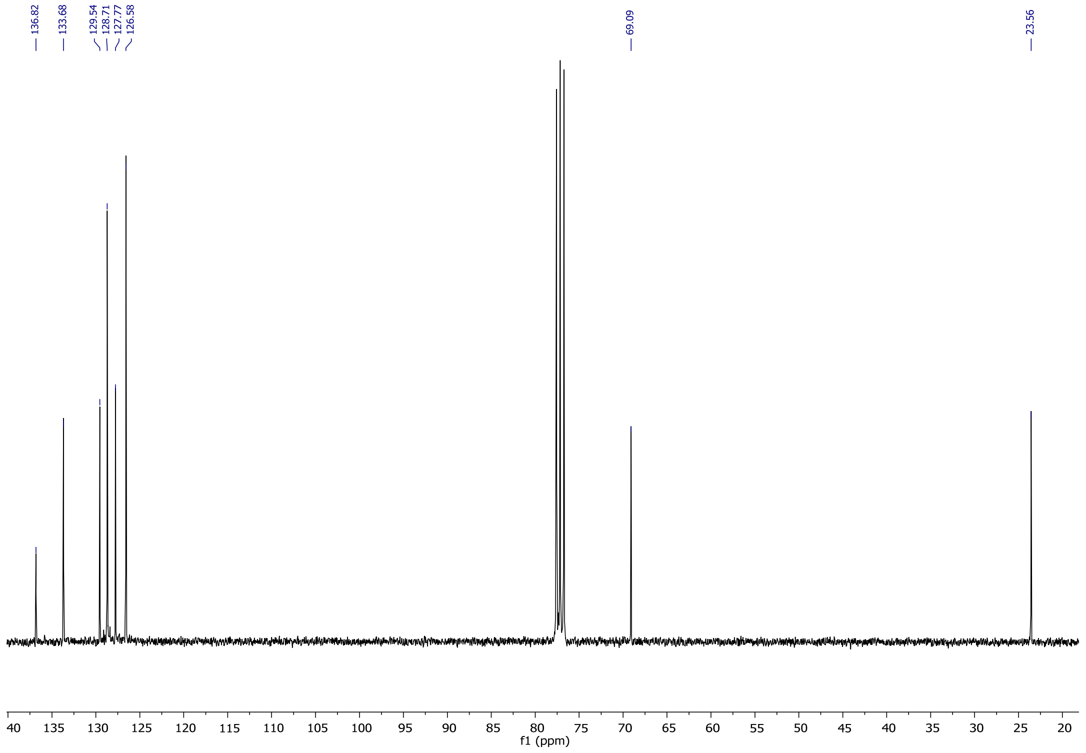

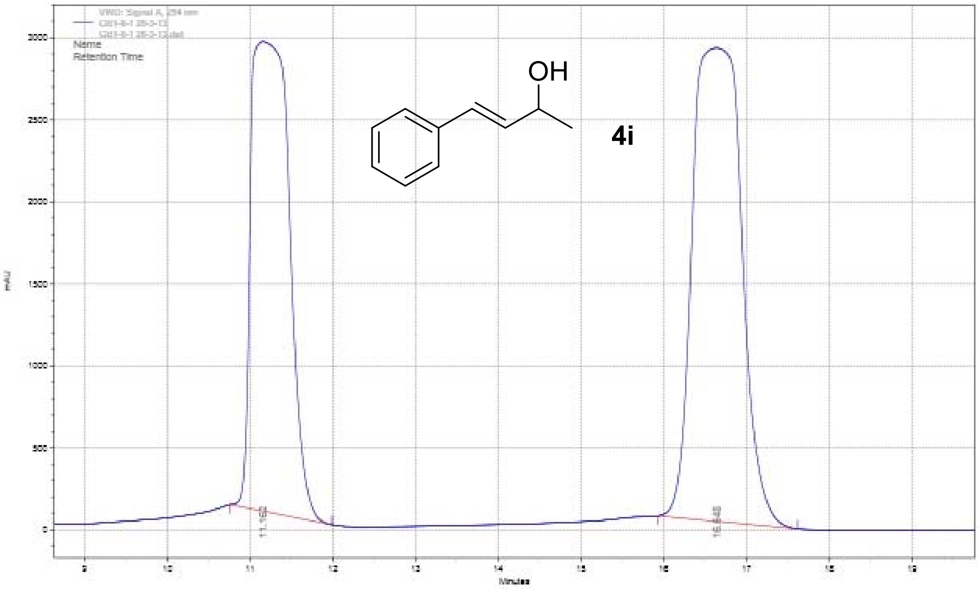

C:\EZChrom Elite\Enterprise\Projects\Default\Data\GB1-8-1 26-3-13.dat, VWD: Signal A, 254 nm

VWD: Signal A,
254 nm Results

Retention Time	Area	Area %	Height	Height %
11.162	1551827327	43.65	48015153	49.80
16.648	2003516851	56.35	48405340	50.20

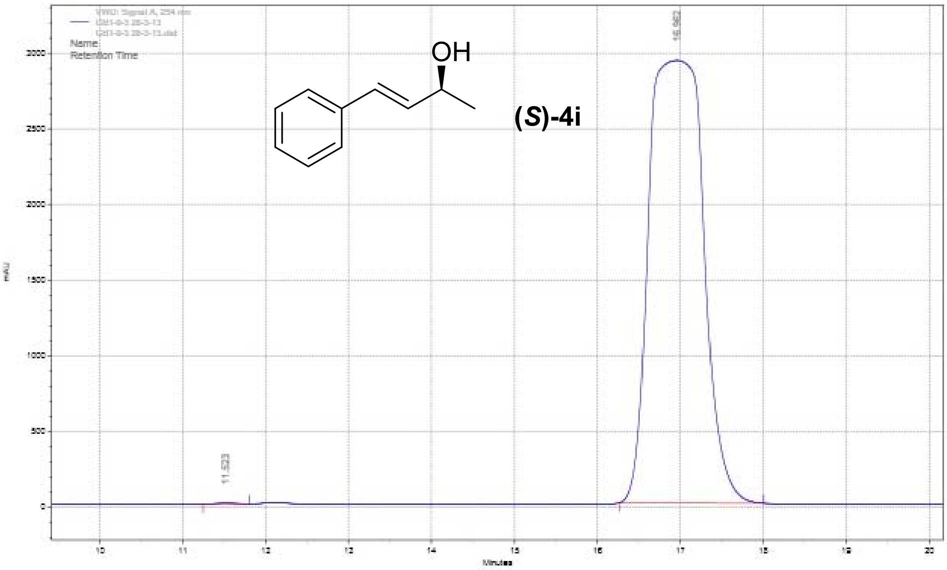

C:\EZChrom Elite\Enterprise\Projects\Default\Data\GB1-9-3 28-3-13.dat, VWD: Signal A, 254 nm

VWD: Signal A,
254 nm Results

Retention Time	Area	Area %	Height	Height %
11.523	1206238	0.05	80486	0.16
16.962	2246535158	99.95	49079774	99.84

(*S*)-(But-3-yn-2-yloxy)(*tert*-butyl)dimethylsilane

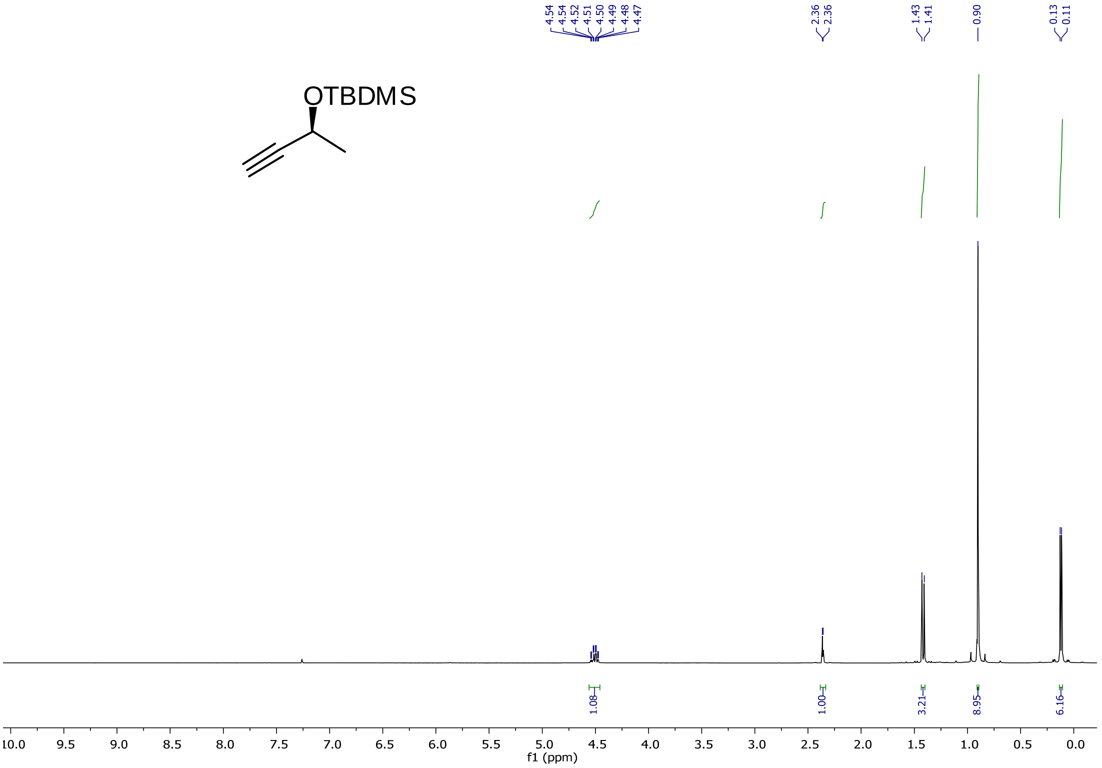

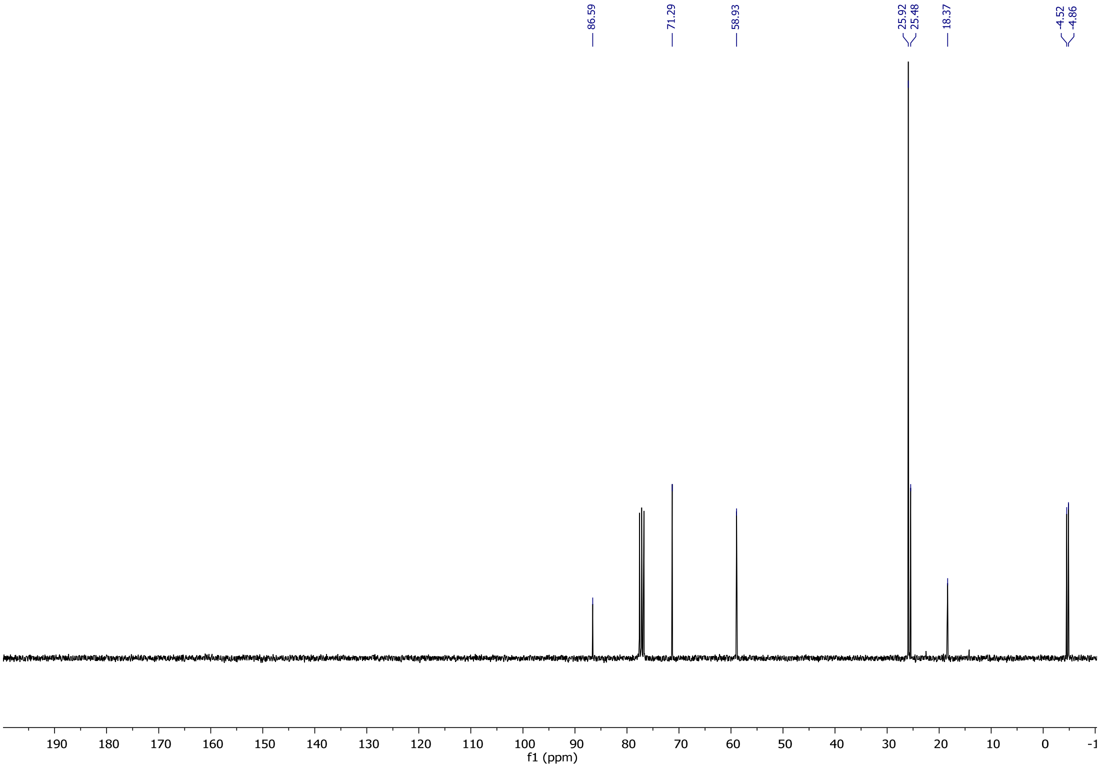

(*S*)-4-(*tert*-Butyldimethylsilyloxy)pent-2-yn-1-ol

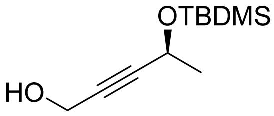

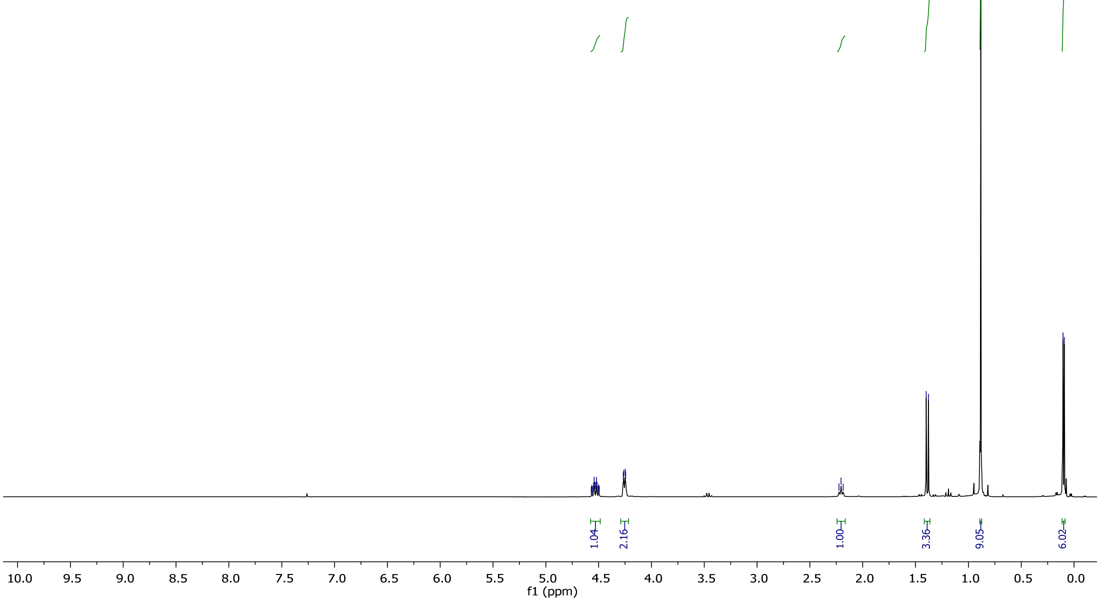

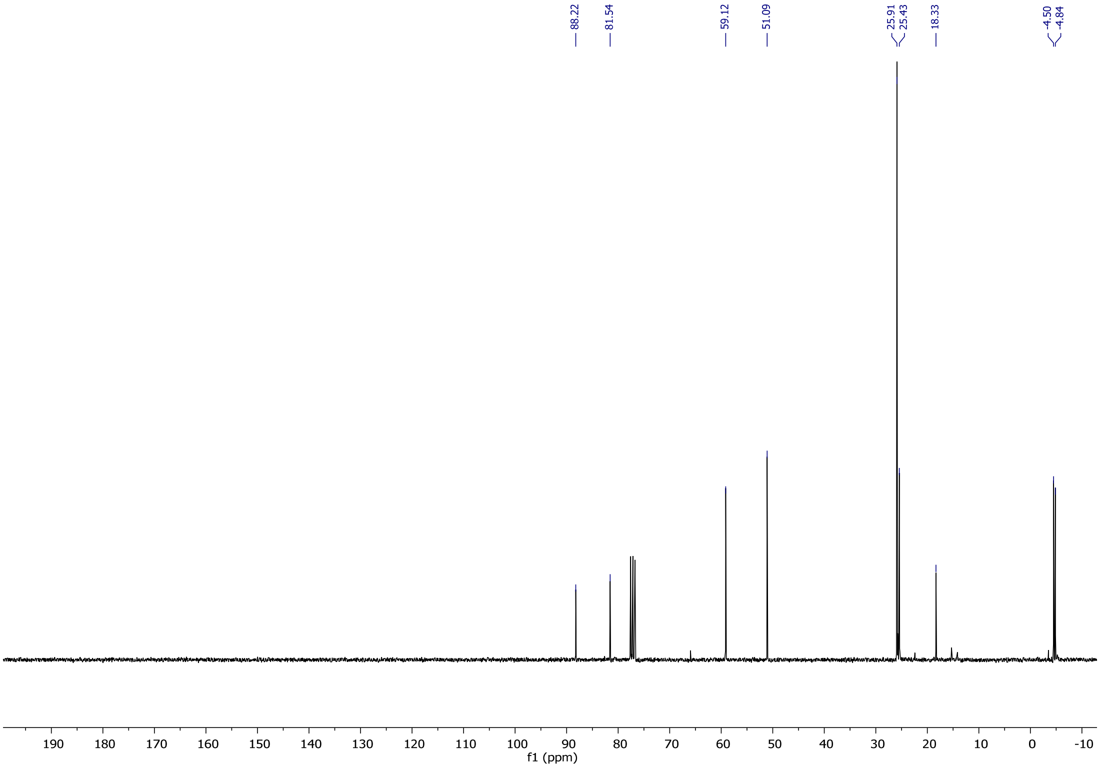

(*S*,*E*)-5-(Benzyloxy)pent-3-en-2-ol (*S*)-4j

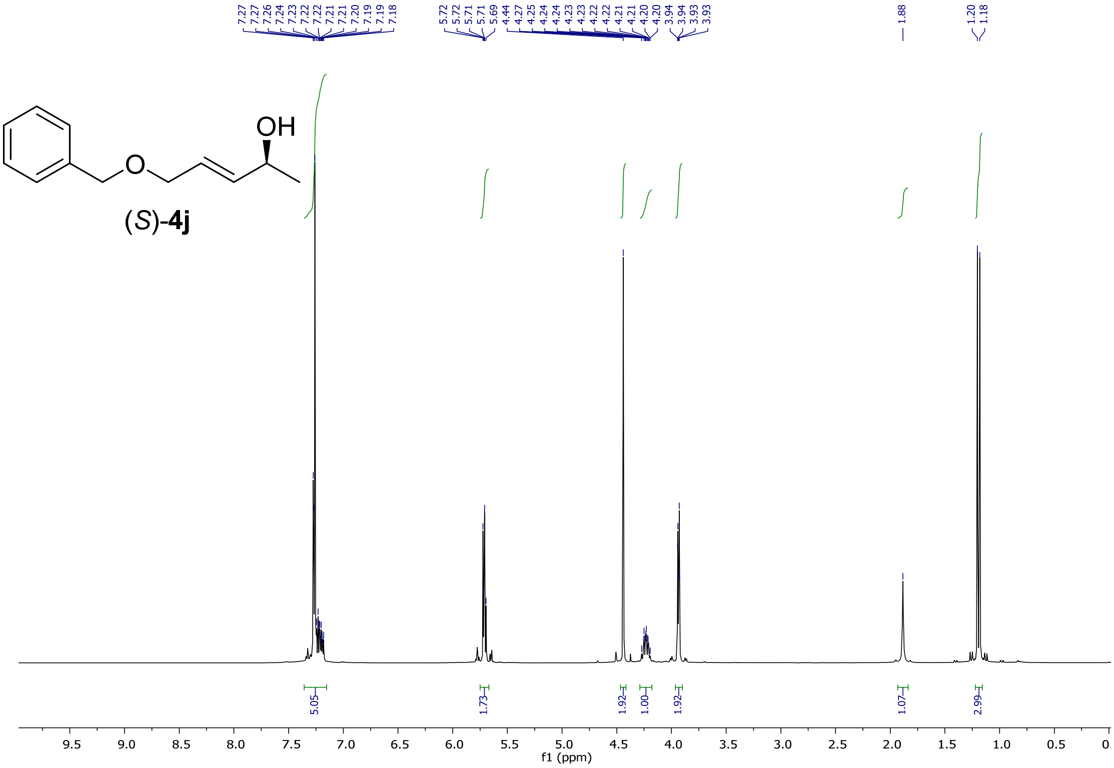

(*S*)-**4j**

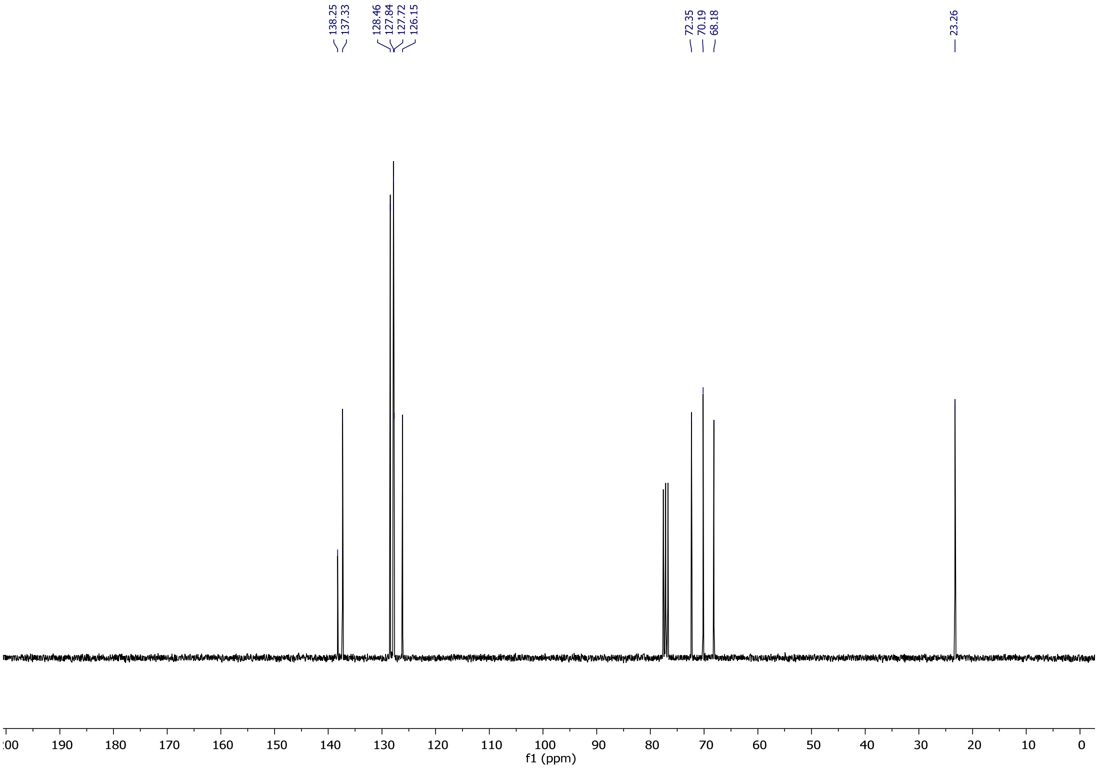

(*R*, *E*)-Pent-3-en-2-ol (*R*)-4k

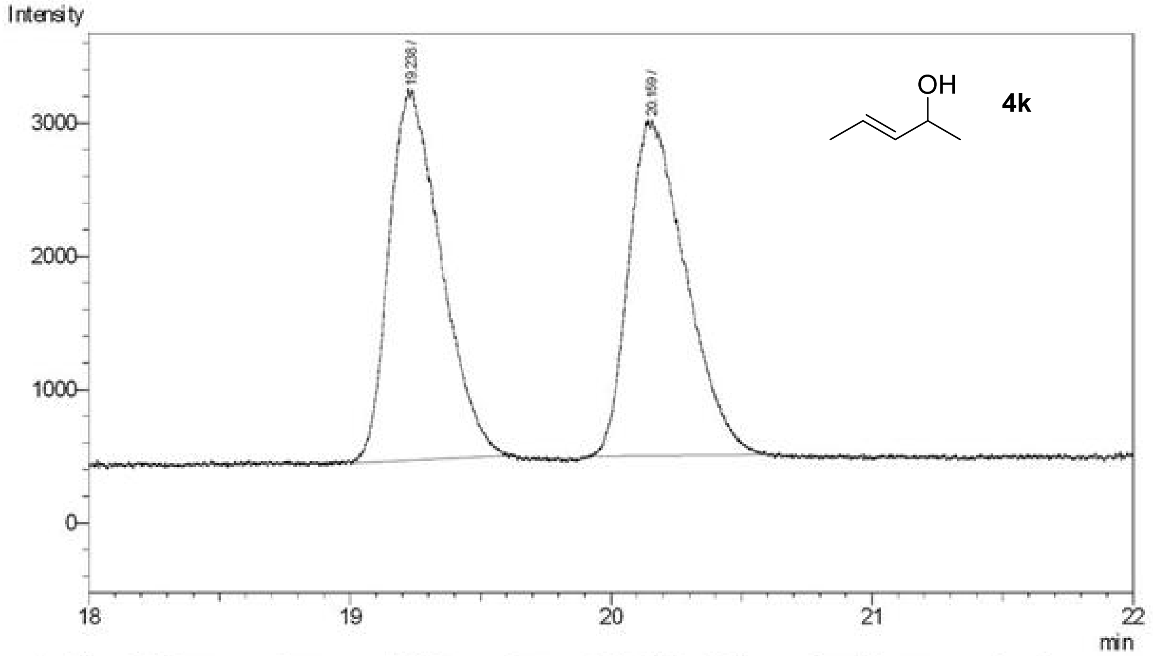

Peak#	Ret.Time	Area	Height	Conc.	Unit	Mark	ID#	Cmpd Name	Area%
1	19.238	38518	2769	50.083					50.0831
2	20.159	38391	2514	49.917					49.9169
Total		76909	5283						100.0000

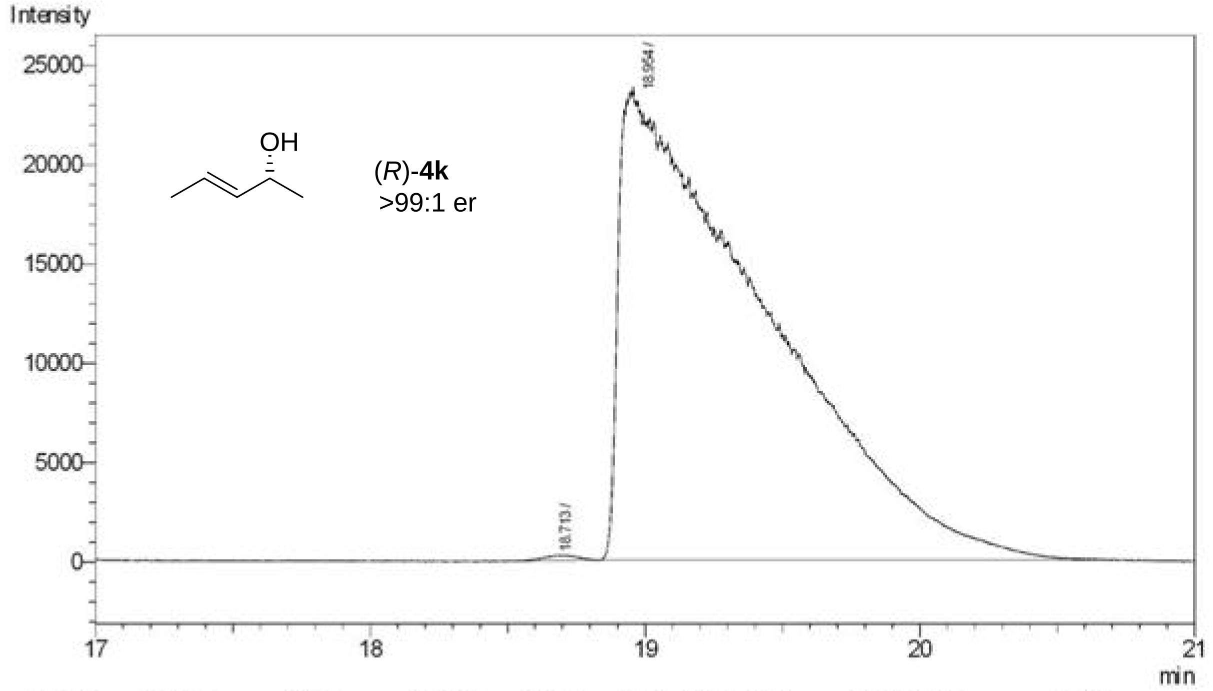

Peak#	Ret.Time	Area	Height	Conc.	Unit	Mark	ID#	Cmpd Name	Area%
1	18.713	2081	246	0.238					0.2375
2	18.954	874210	23558	99.762					99.7625
Total		876291	23804						100.0000

(*R*, *E*)-2,2-Dimethylhex-4-en-3-ol (*R*)-4l

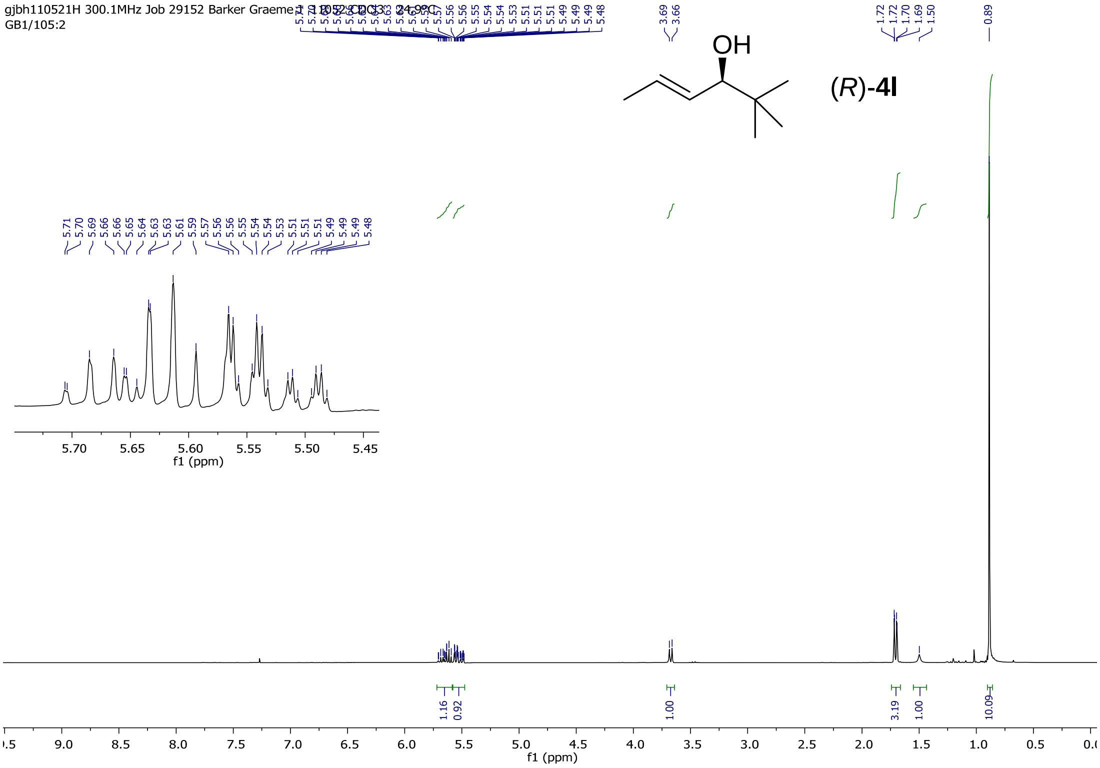

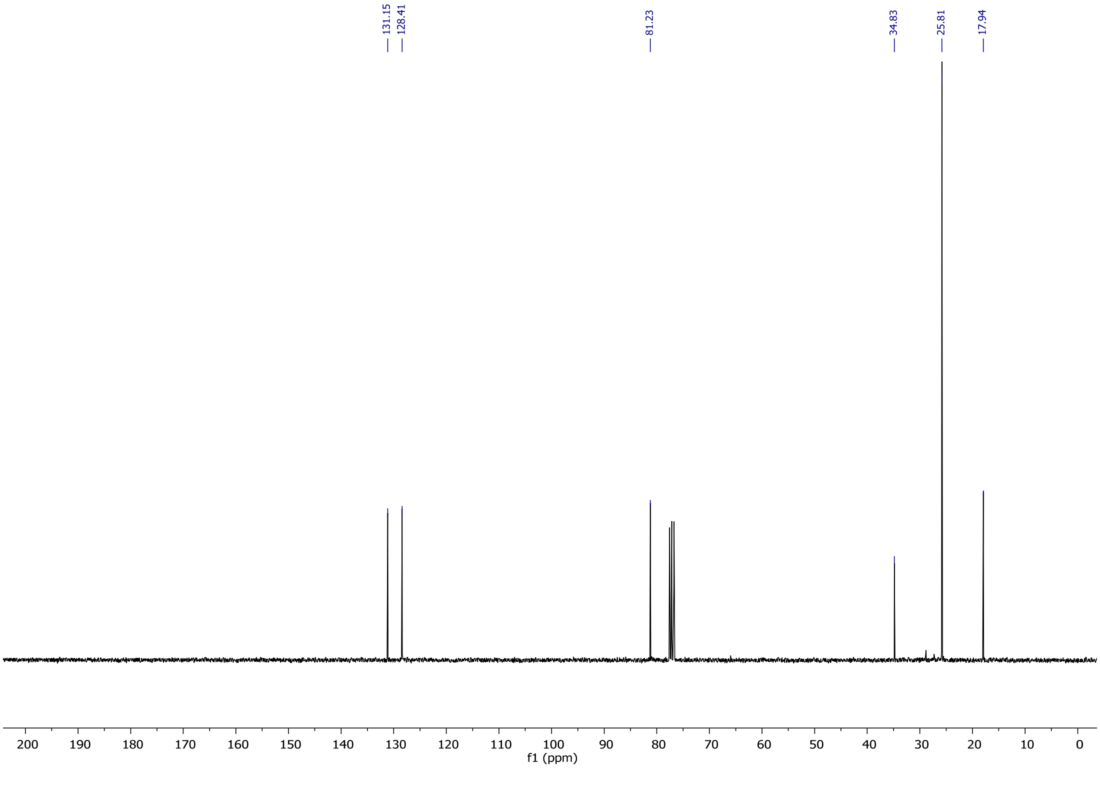

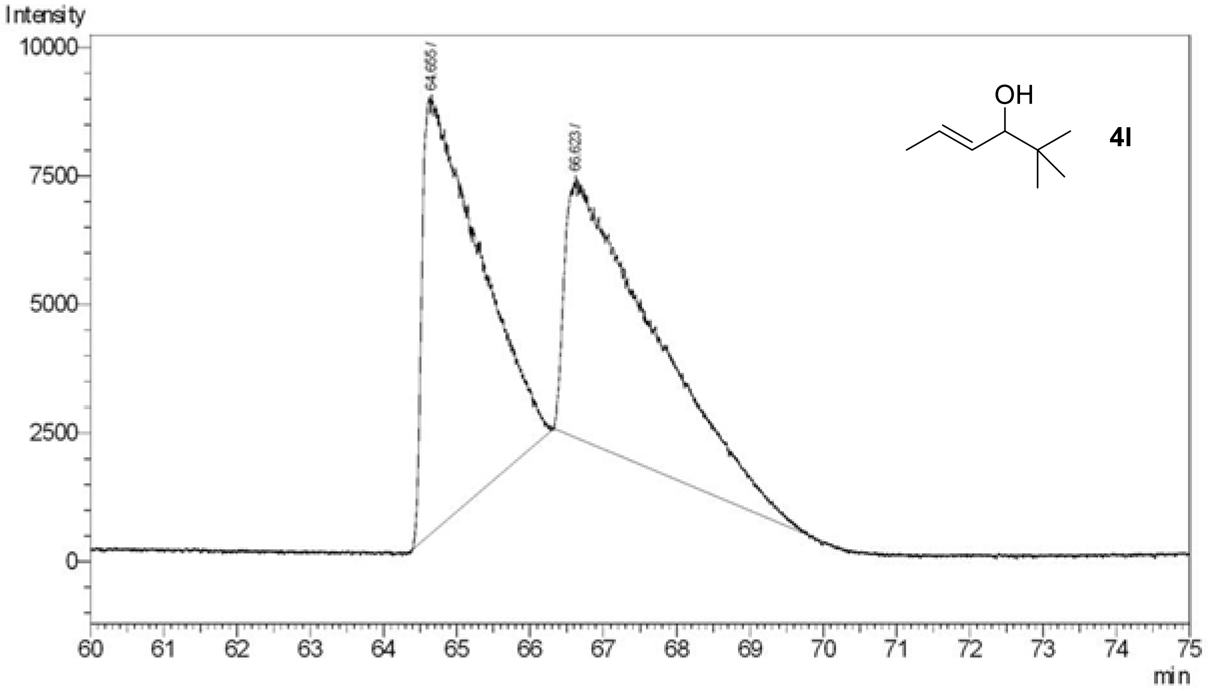

Peak#	Ret.Time	Area	Height	Conc.	Unit	Mark	ID#	Cmpd Name	Area%
1	64.655	453863	8513	51.149					51.1491
2	66.623	433471	5080	48.851					48.8509
Total		887334	13593						100.0000

-

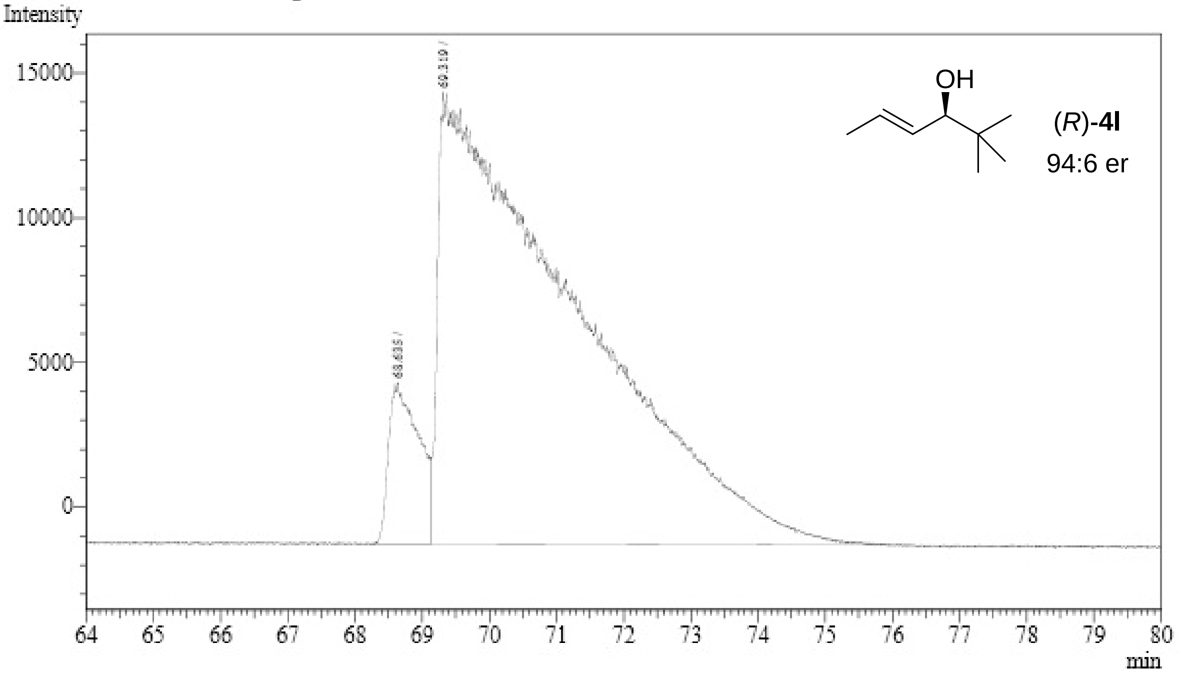

Peak#	Ret.Time	Area	Height	Conc.	Unit	Mark	ID#	Cmpd Name	Area%
1	68.635	174793	5583	7.334					7.3339
2	69.319	2208578	15603	92.666		V			92.6661
Total		2383371	21186						100.0000

(*R*, *E*)-3-Methylpent-3-en-2-ol (*R*)-4m

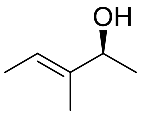

(*R*)-**4m**

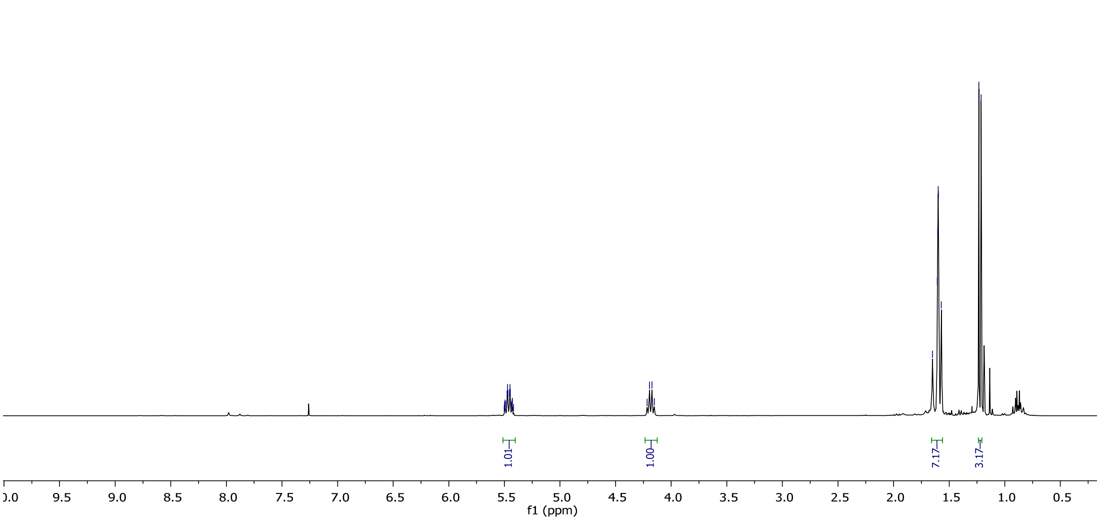

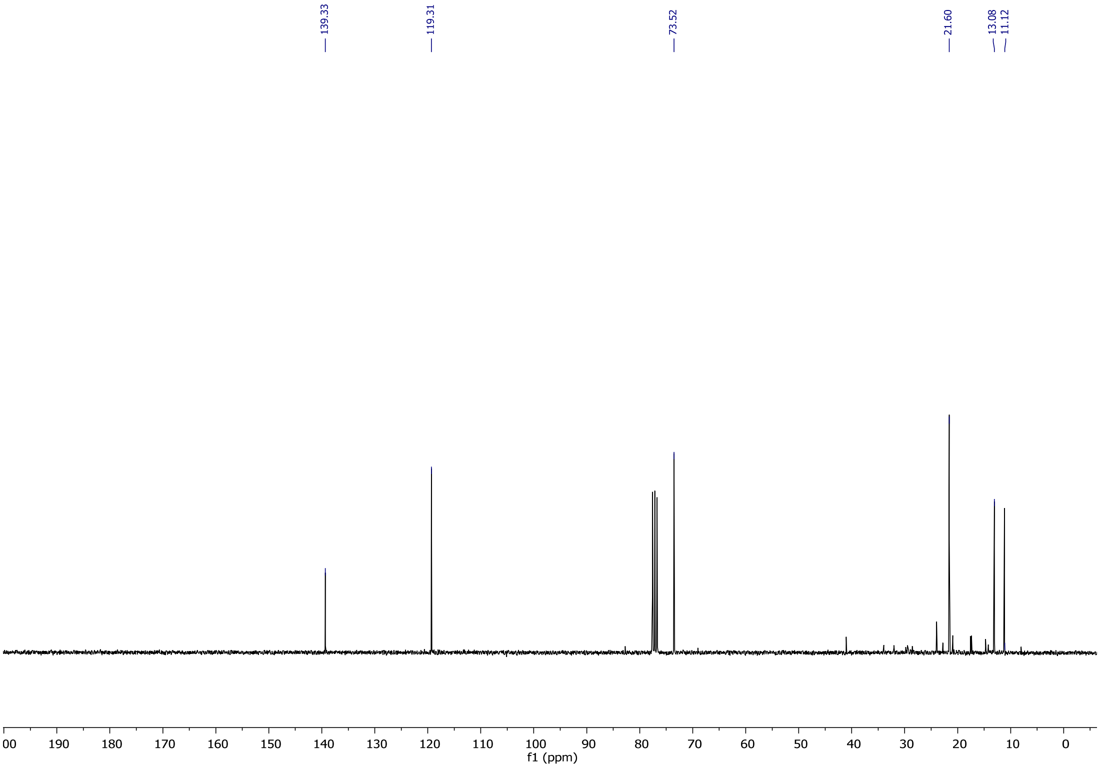

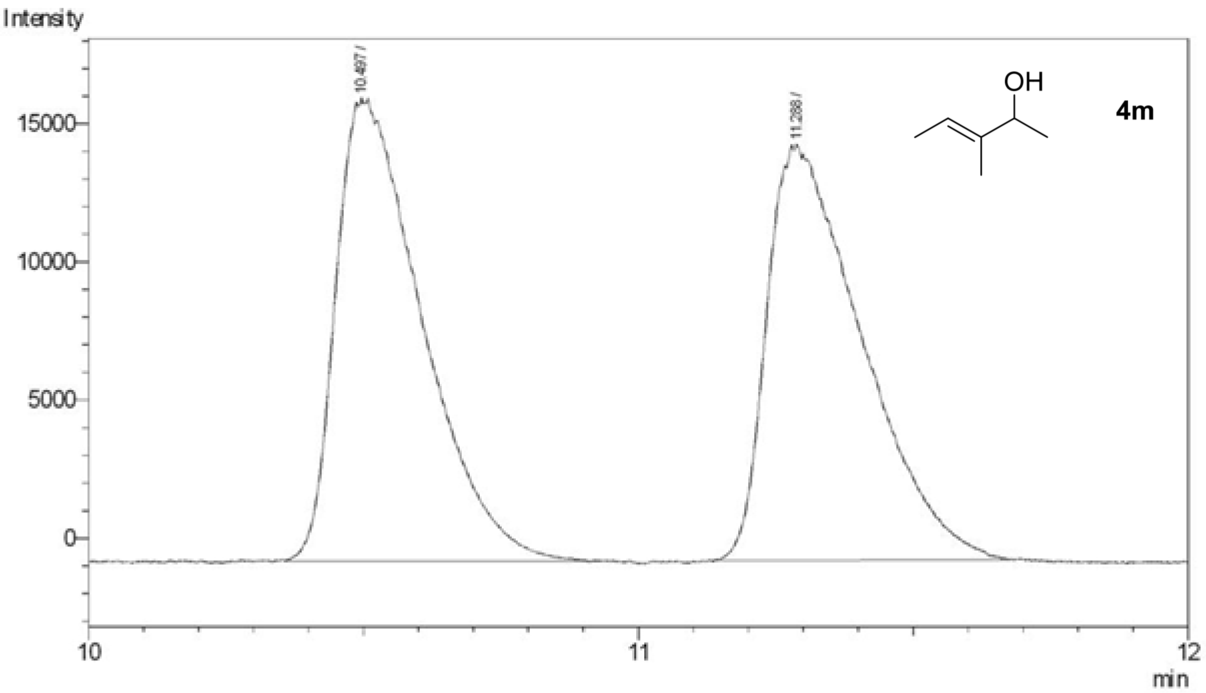

Peak#	Ret.Time	Area	Height	Conc.	Unit	Mark	ID#	Cmpd Name	Area%
1	10.497	182082	16685	50.225					50.2249
2	11.288	180452	15019	49.775					49.7751
Total		362534	31704						100.0000

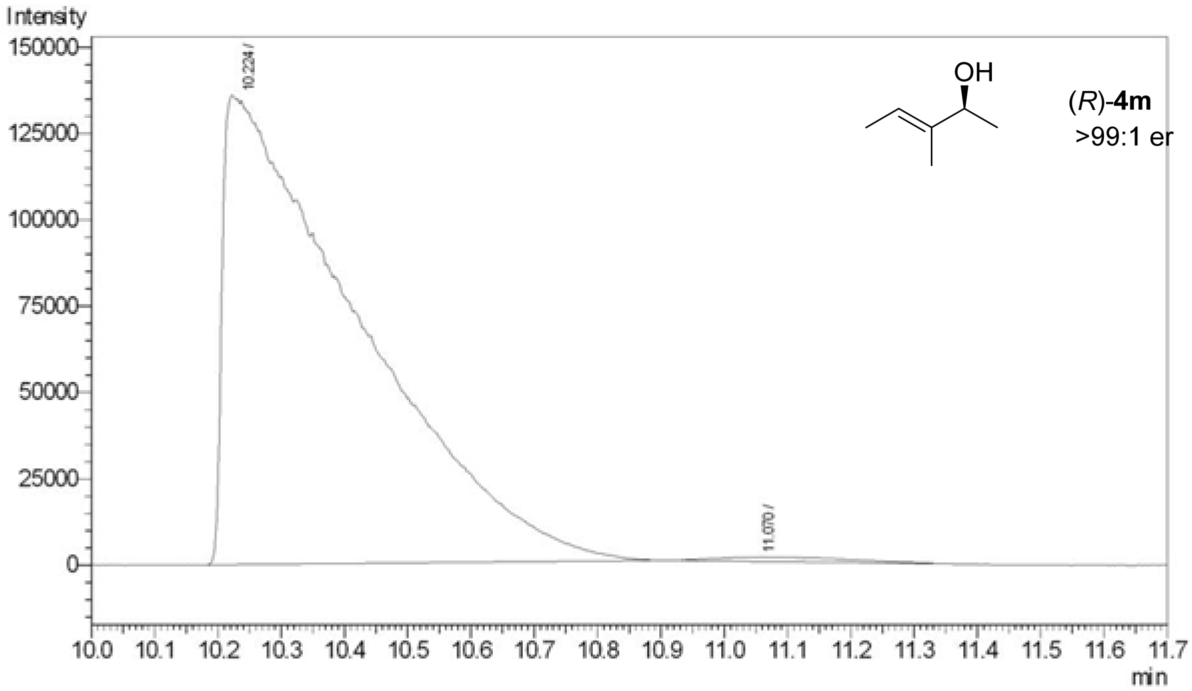

Peak#	Ret.Time	Area	Height	Conc.	Unit	Mark	ID#	Cmpd Name	Area%
1	10.224	2022331	135824	99.079					99.0790
2	11.070	18799	1337	0.921					0.9210
Total		2041130	137161						100.0000

(*R*, *E*)-1-Cyclohexyl-2-methylbut-2-en-1-ol (*R*)-4n

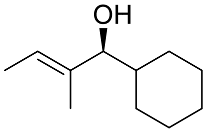

(*R*)-**4n**

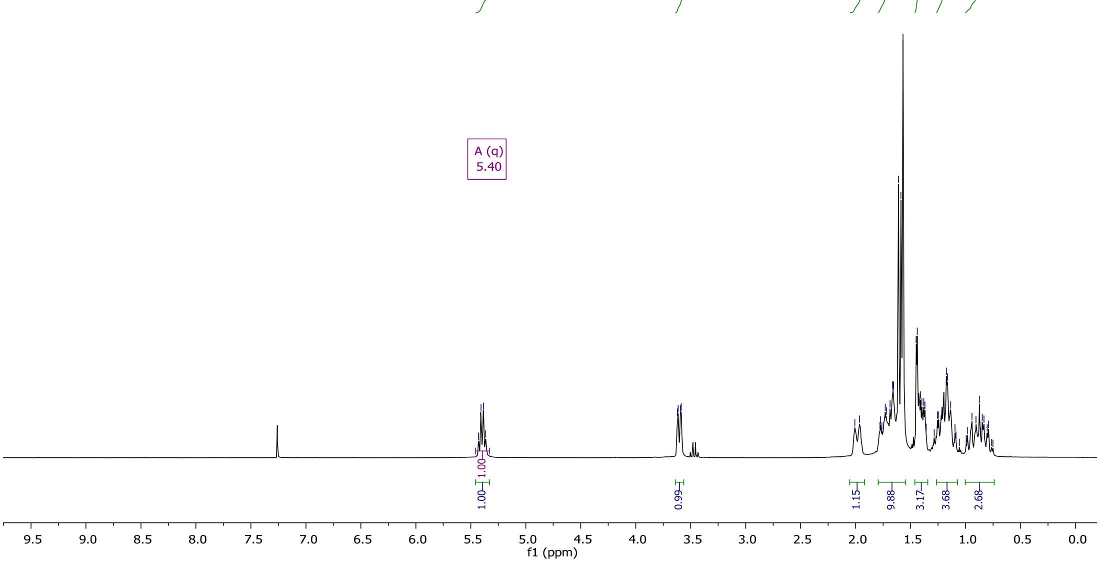

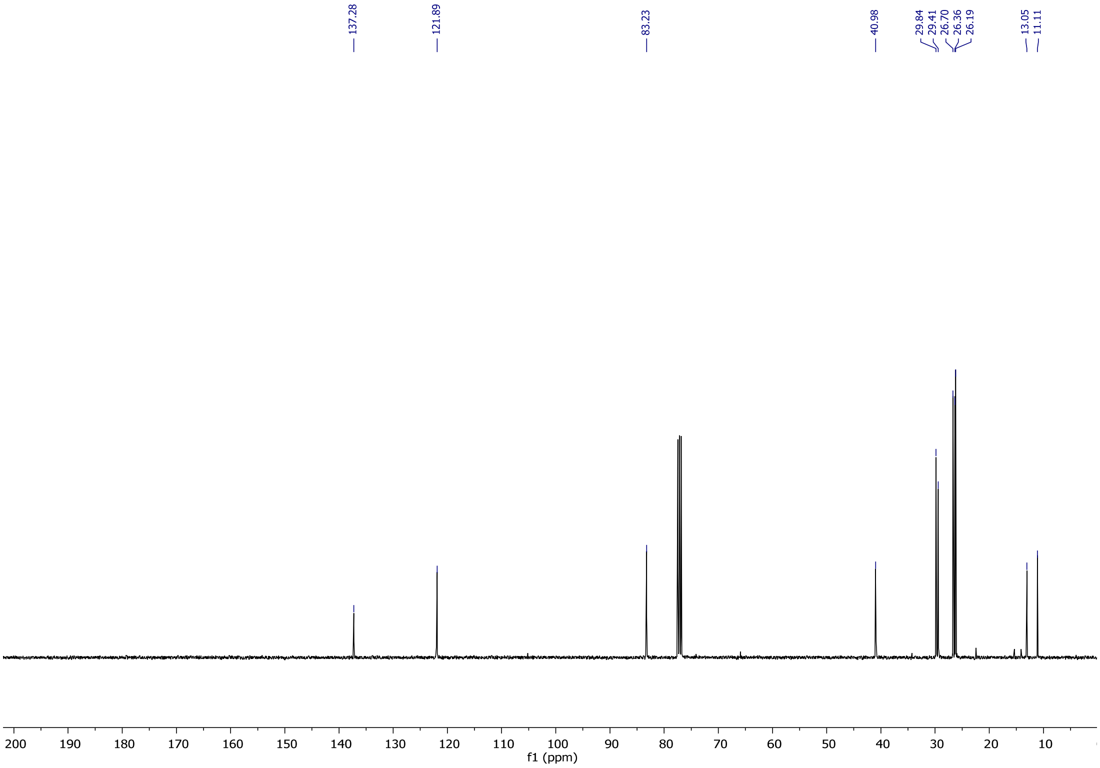

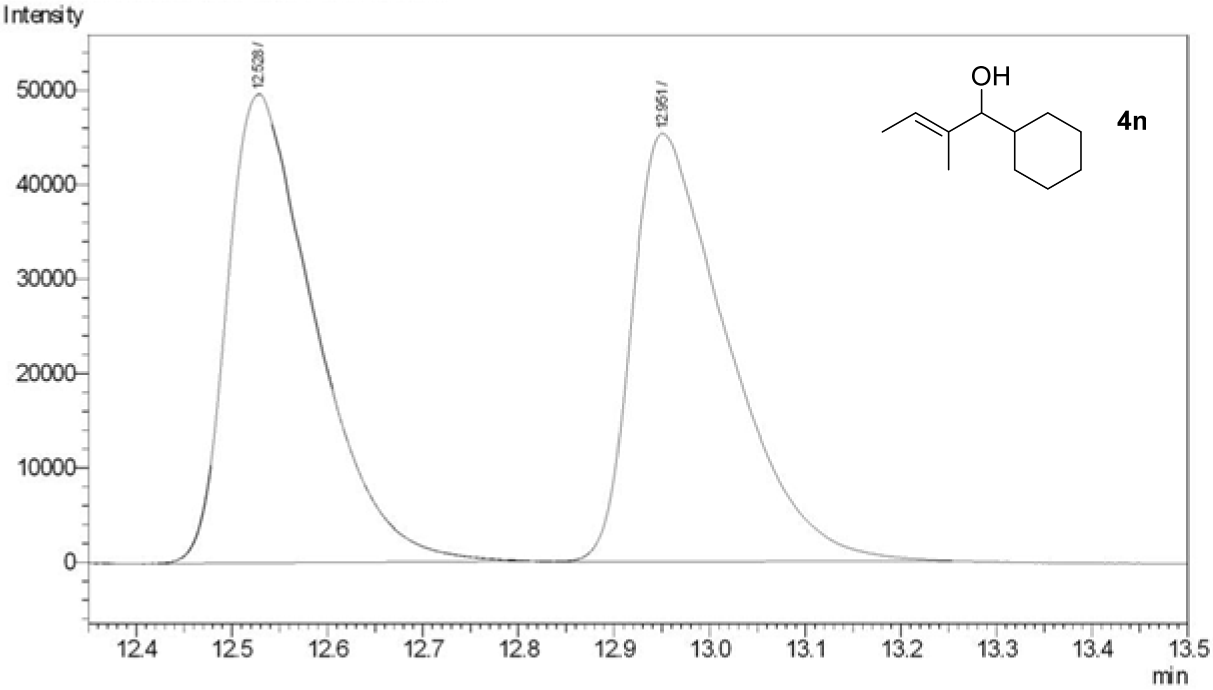

Peak#	Ret.Time	Area	Height	Conc.	Unit	Mark	ID#	Cmpd Name	Area%
1	12.528	318067	49625	50.120					50.1204
2	12.951	316539	45225	49.880					49.8796
Total		634606	94850						100.0000

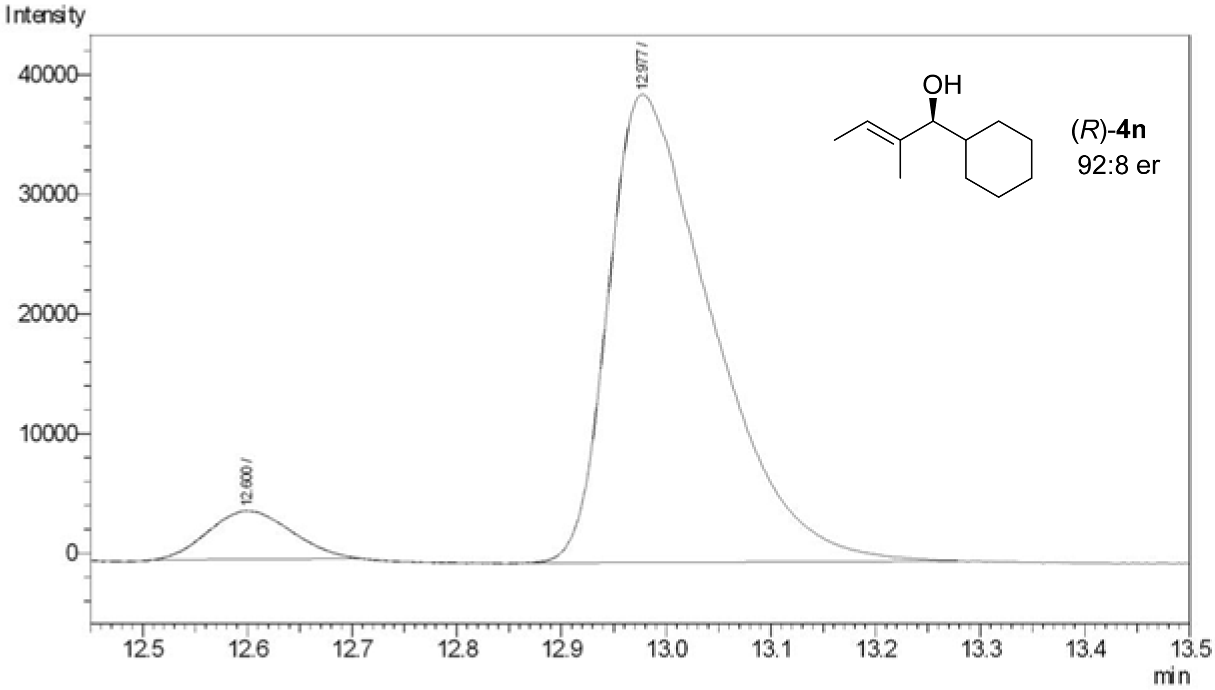

Peak#	Ret.Time	Area	Height	Conc.	Unit	Mark	ID#	Cmpd Name	Area%
1	12.600	22487	4038	7.676					7.6760
2	12.977	270463	39092	92.324					92.3240
Total		292950	43130						100.0000

Allylic Alcohol Scope Spectra

(*E*)-Methyl 4-((oct-3-en-2-yloxy)methyl)benzoate 5ab

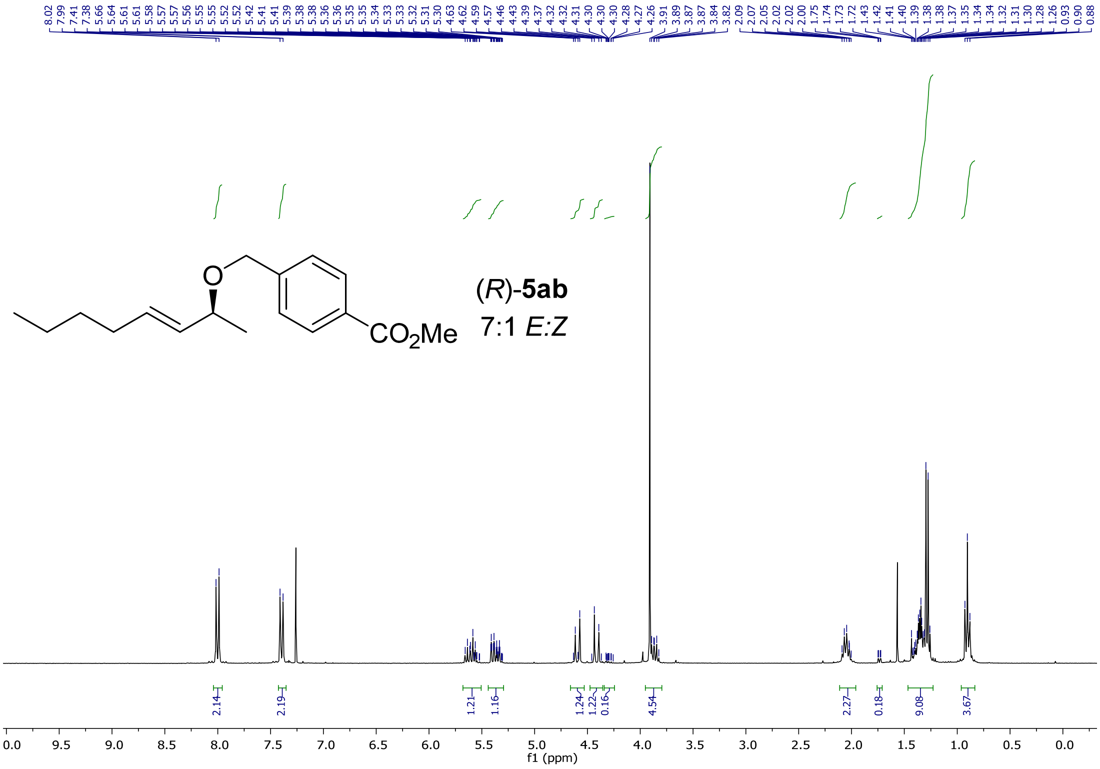

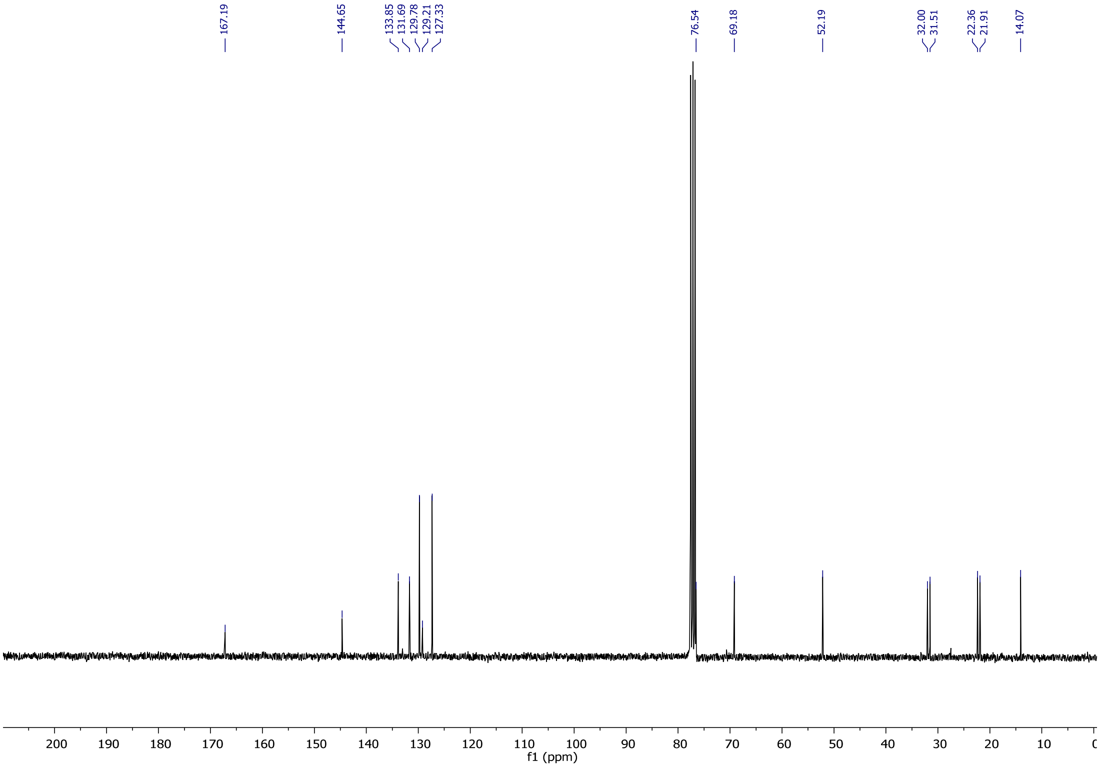

(*E*)-(4-((Oct-3-en-2-yloxy)methyl)phenyl)methanol (S1)

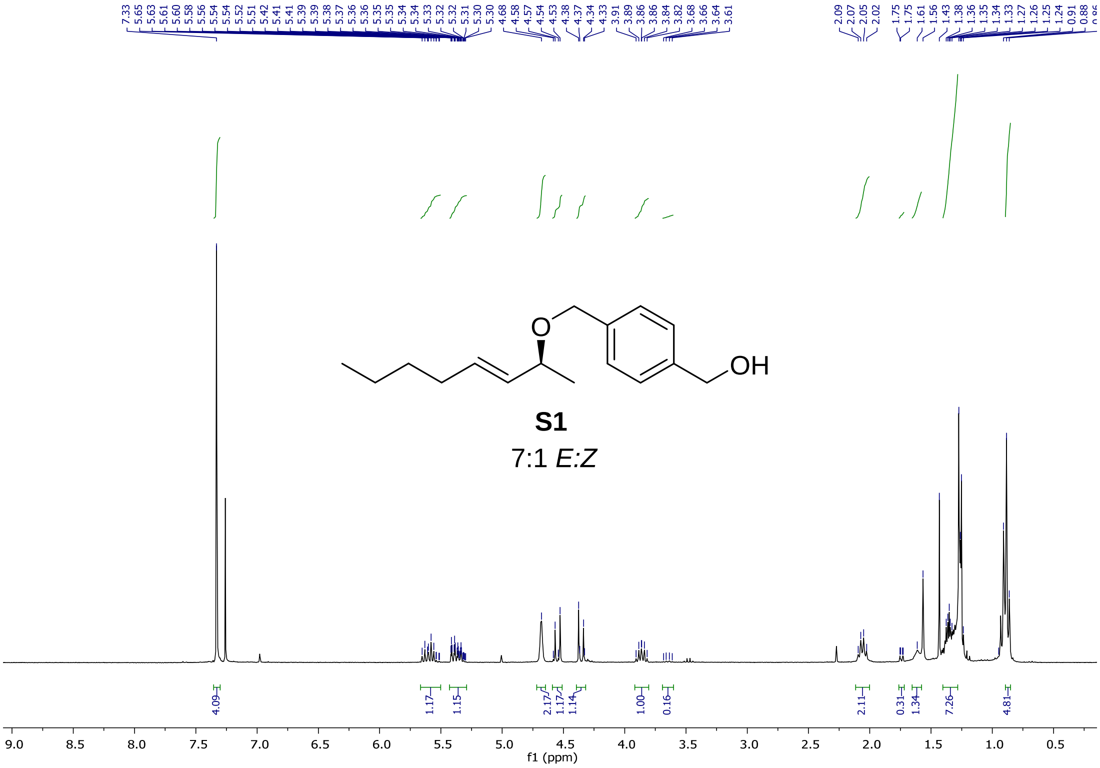

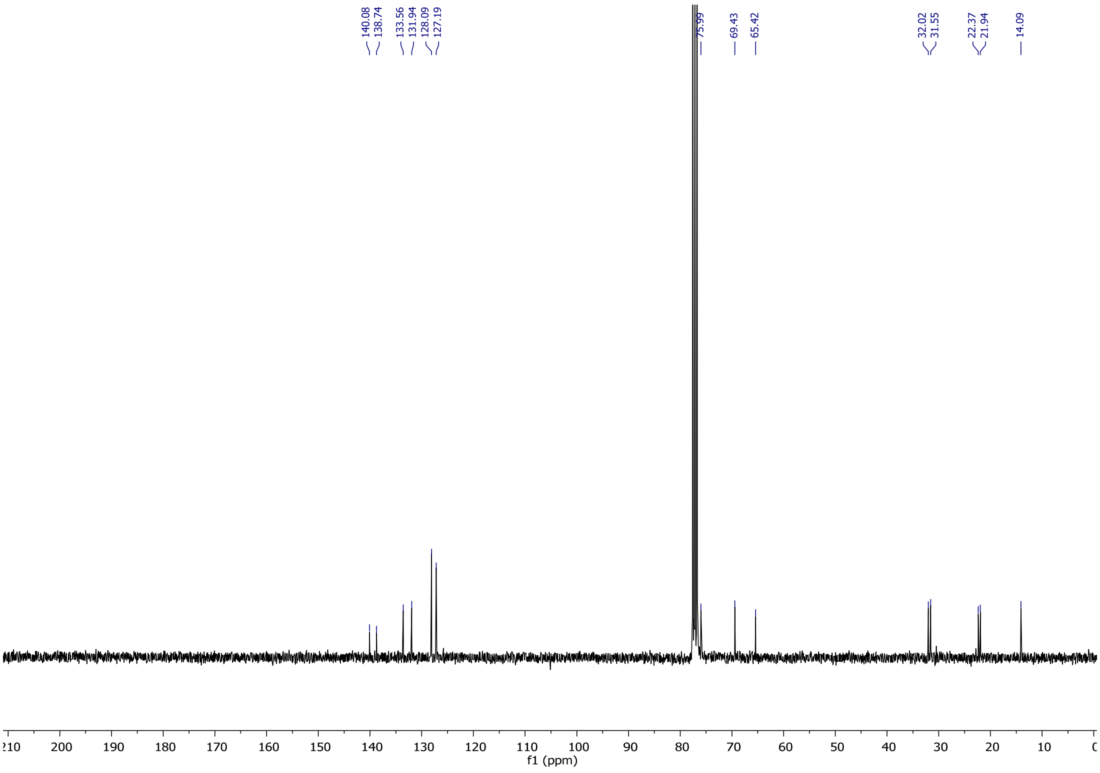

***Rac*-(*E*)-(4-((Oct-3-en-2-yloxy)methyl)phenyl)methanol S1**

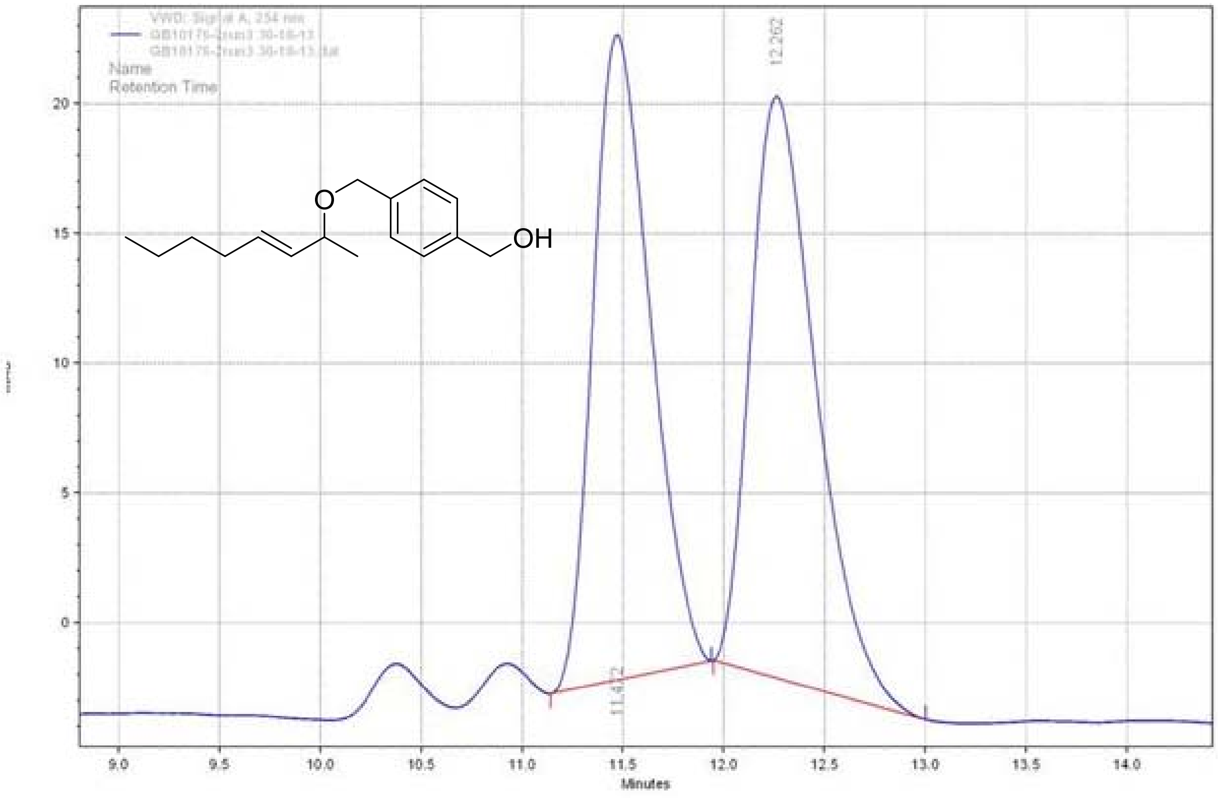

— C:\EZChrom Elite\Enterprise\Projects\Default\Data\GB10176-2run3 30-10-13.dat, VWD: Signal A, 254 nm

VWD: Signal A, 254 nm Results

Retention Time	Area	Area %	Height	Height %
11.472	8262970	49.30	416863	52.60
12.262	8497042	50.70	375721	47.40

***Rac*-(*Z*)-(4-((Oct-3-en-2-yloxy)methyl)phenyl)methanol**

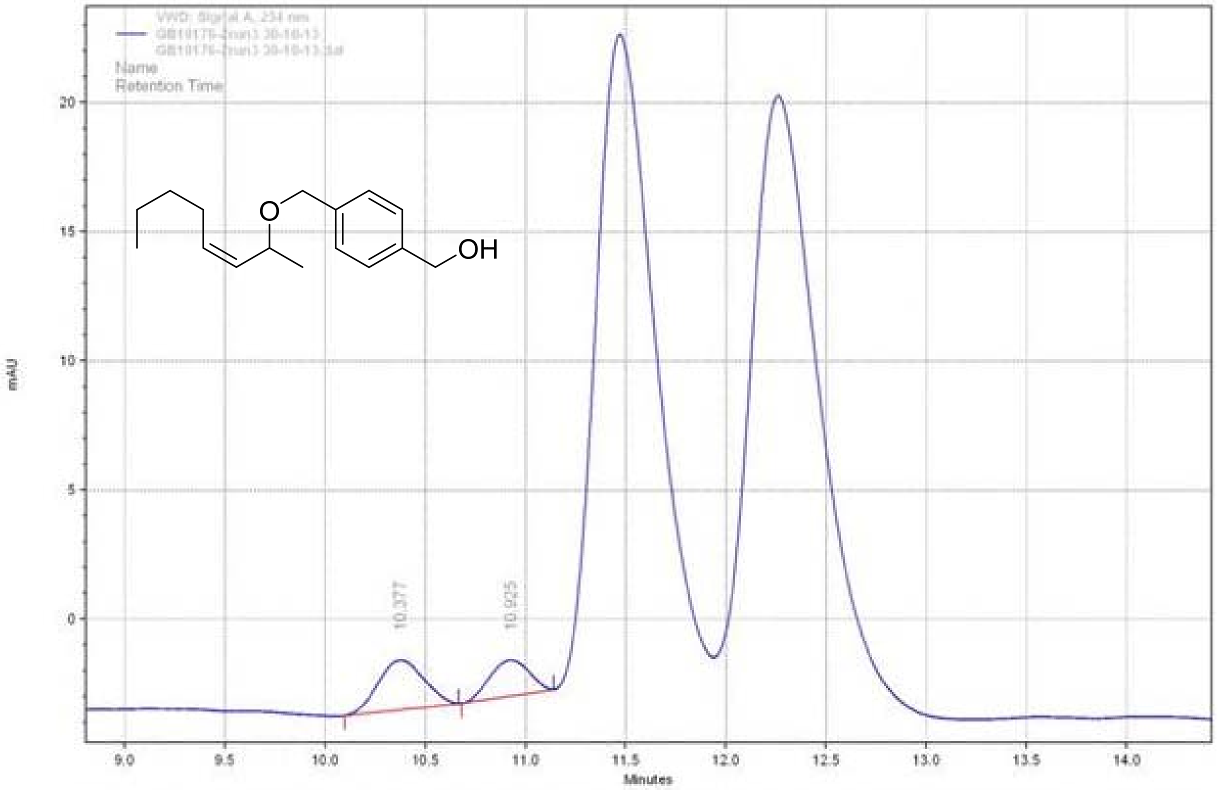

C:\EZChrom Elite\Enterprise\Projects\Default\Data\GB10176-2run3 30-10-13.dat, VWD: Signal A, 254 nm

VWD: Signal A,
254 nm Results

Retention Time	Area	Area %	Height	Height %
10.377	507058	61.38	32110	57.88
10.925	319014	38.62	23366	42.12

(*S*,*E*)-(4-((Oct-3-en-2-yloxy)methyl)phenyl)methanol

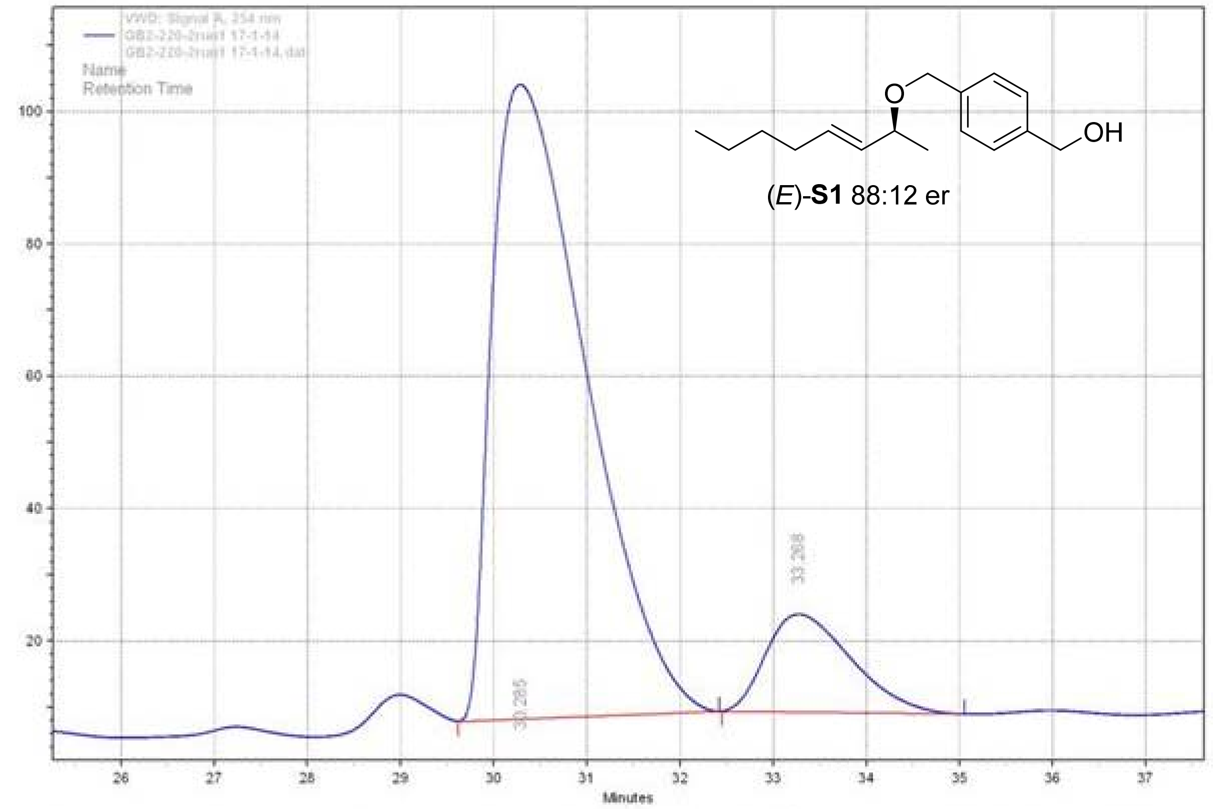

— C:\EZChrom Elite\Enterprise\Projects\Default\Data\GB2-220-2run1 17-1-14.dat, VWD: Signal A, 254 nm

VWD: Signal A, 254 nm Results

Retention Time	Area	Area %	Height	Height %
30.285	112657427	87.69	1608003	86.64
33.268	15814298	12.31	247895	13.36

This is the derivative to determine the er of the E isomer of **5ab**.

(*S*,*Z*)-(4-((Oct-3-en-2-yloxy)methyl)phenyl)methanol

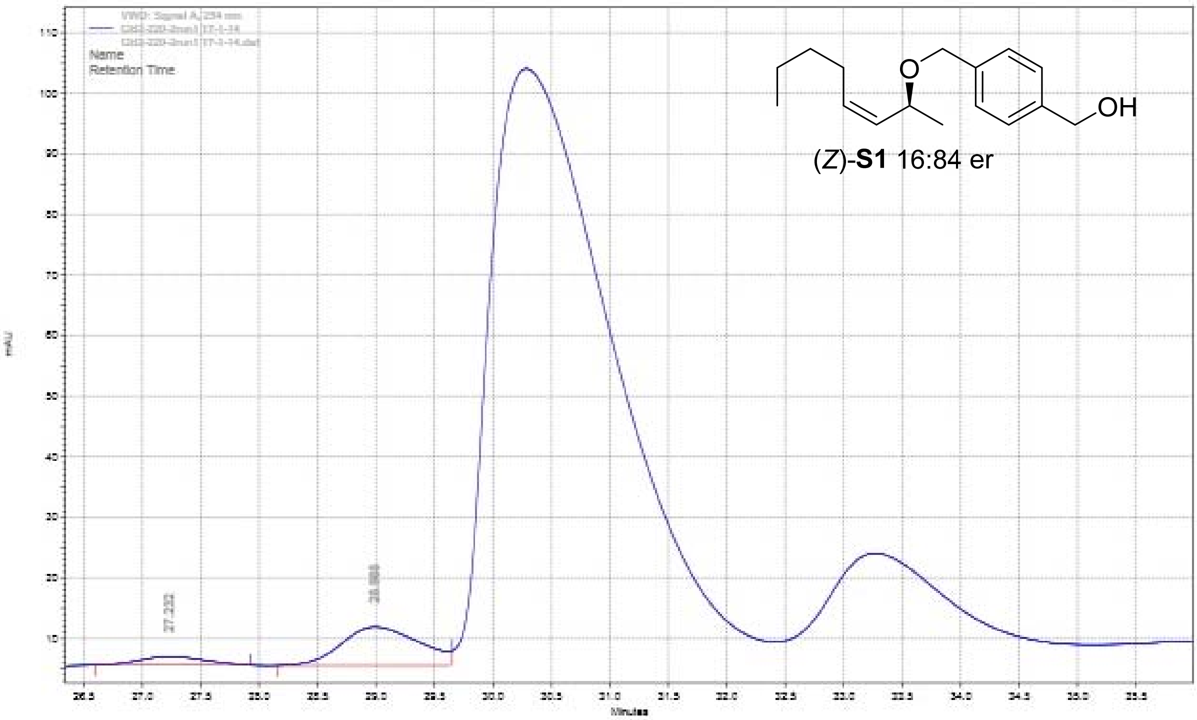

C:\EZChrom Elite\Enterprise\Projects\Default\Data\GB2-220-2run1 17-1-14.dat, VWD: Signal A, 254 nm

VWD: Signal A,
254 nm Results

Retention Time	Area	Area %	Height	Height %
27.232	920820	15.62	24185	18.48
28.988	4974398	84.38	106665	81.52

This is the derivative to determine the er of the Z isomer of **5ab**.

(*R*,*E*)-(4-((Oct-3-en-2-yloxy)methyl)phenyl)methanol S1

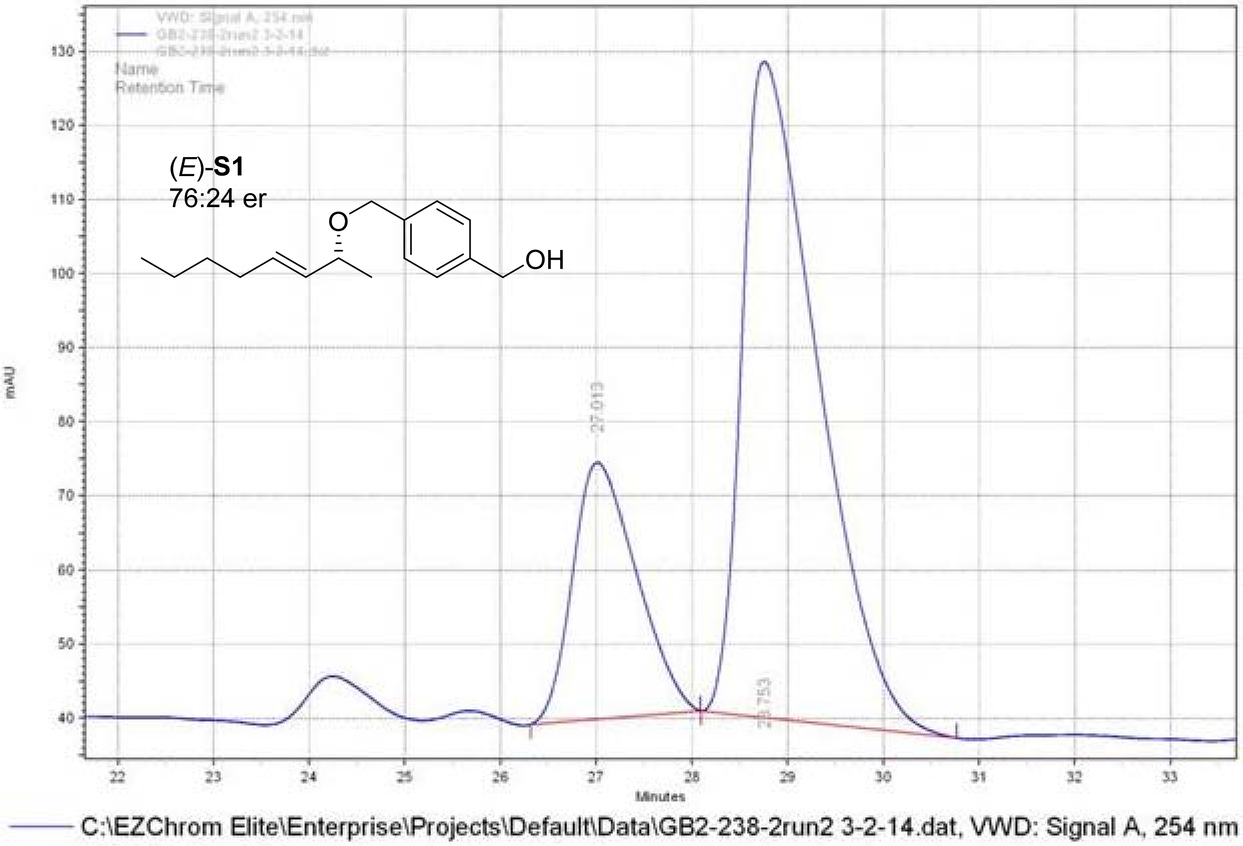

VWD: Signal A, 254 nm Results

Retention Time	Area	Area %	Height	Height %
27.013	26728278	24.49	579878	28.08
28.753	82415894	75.51	1485204	71.92

This is the derivative to determine the er of the E isomer of **5bb**.

(*R*,*Z*)-(4-((Oct-3-en-2-yloxy)methyl)phenyl)methanol S1

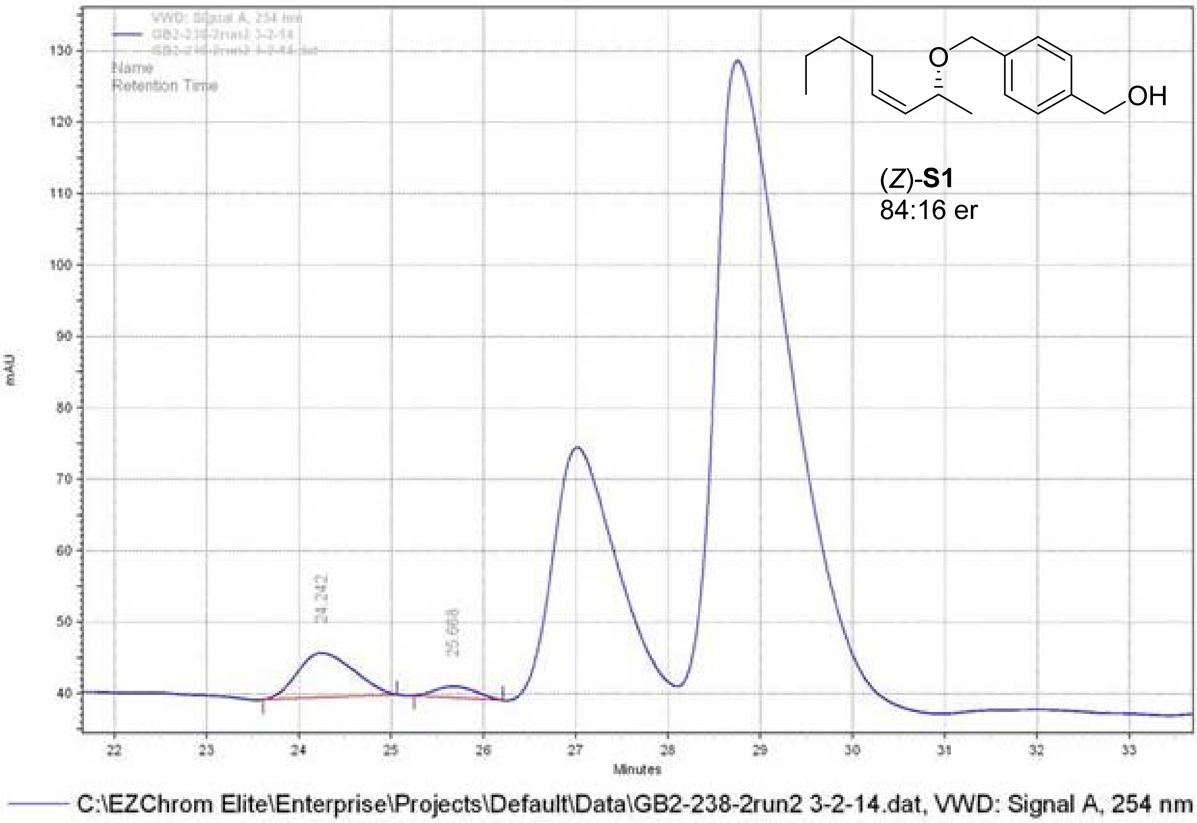

VWD: Signal A,
254 nm Results

Retention Time	Area	Area %	Height	Height %
24.242	4411760	84.29	104515	79.78
25.668	822028	15.71	26490	20.22

This is the derivative to determine the er of the Z isomer of **5bb**.

(*S*, *E*)-Methyl 4-((oct-2-en-4-yloxy)methyl)benzoate (*S*)-5cb

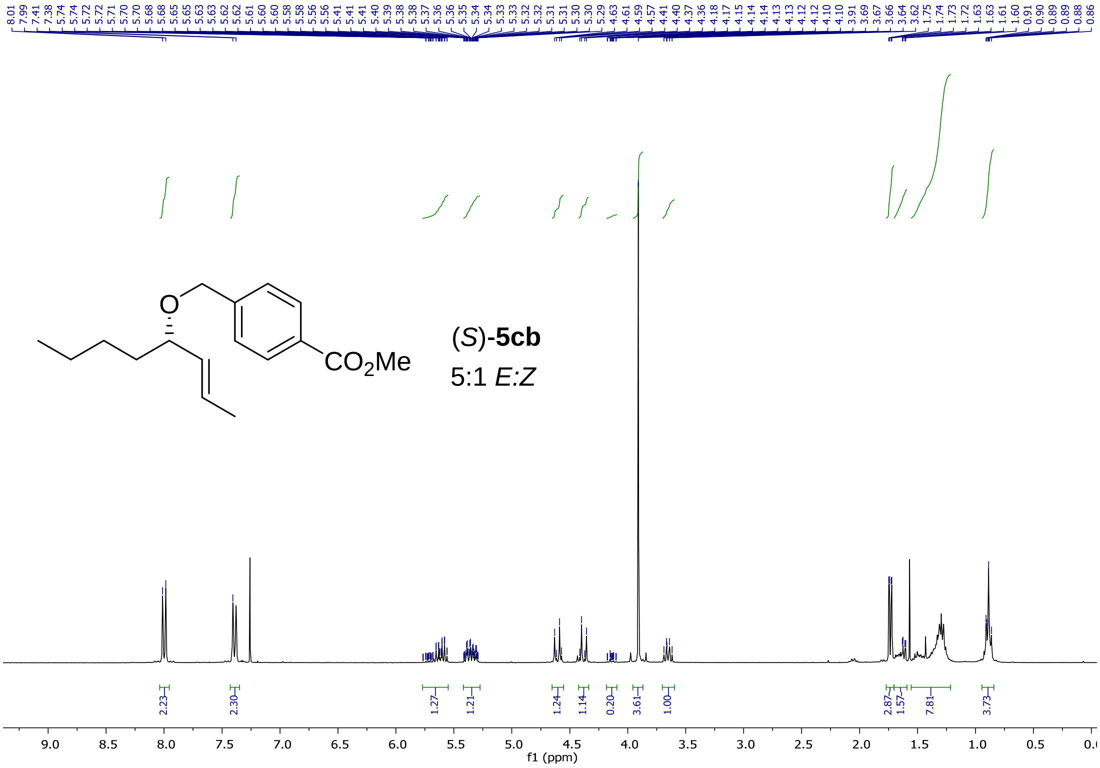

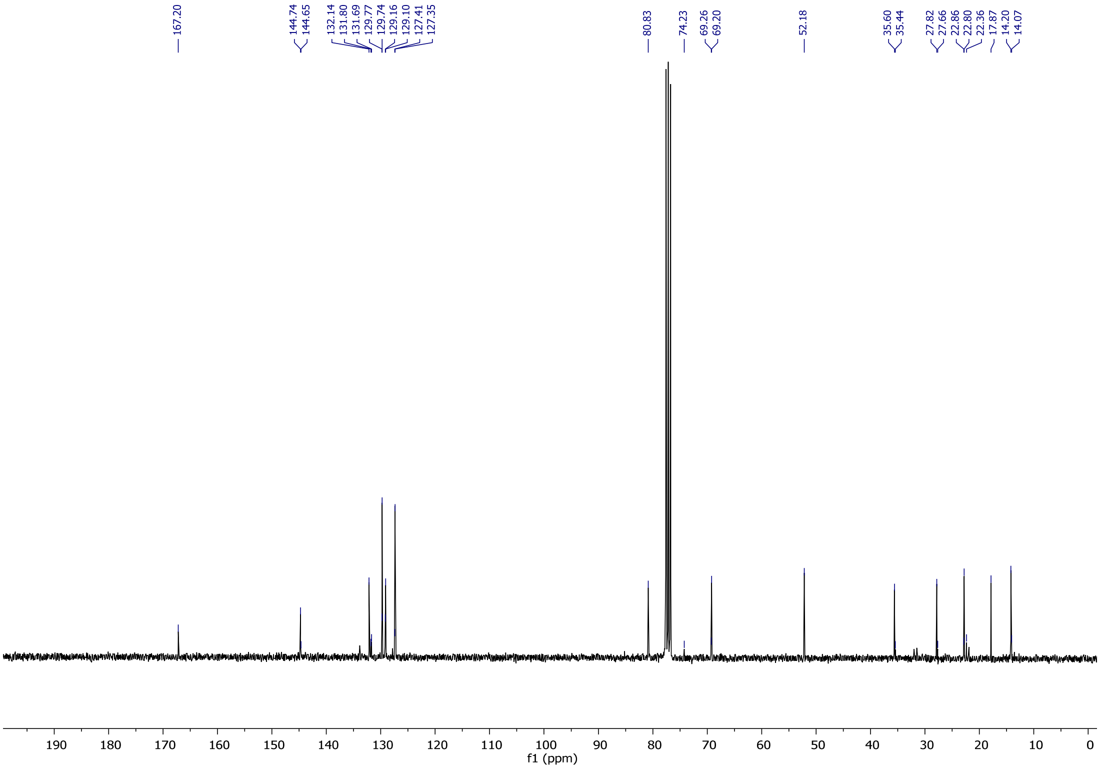

(*S*,*E*)-(4-((Oct-2-en-4-yloxy)methyl)phenyl)methanol S2

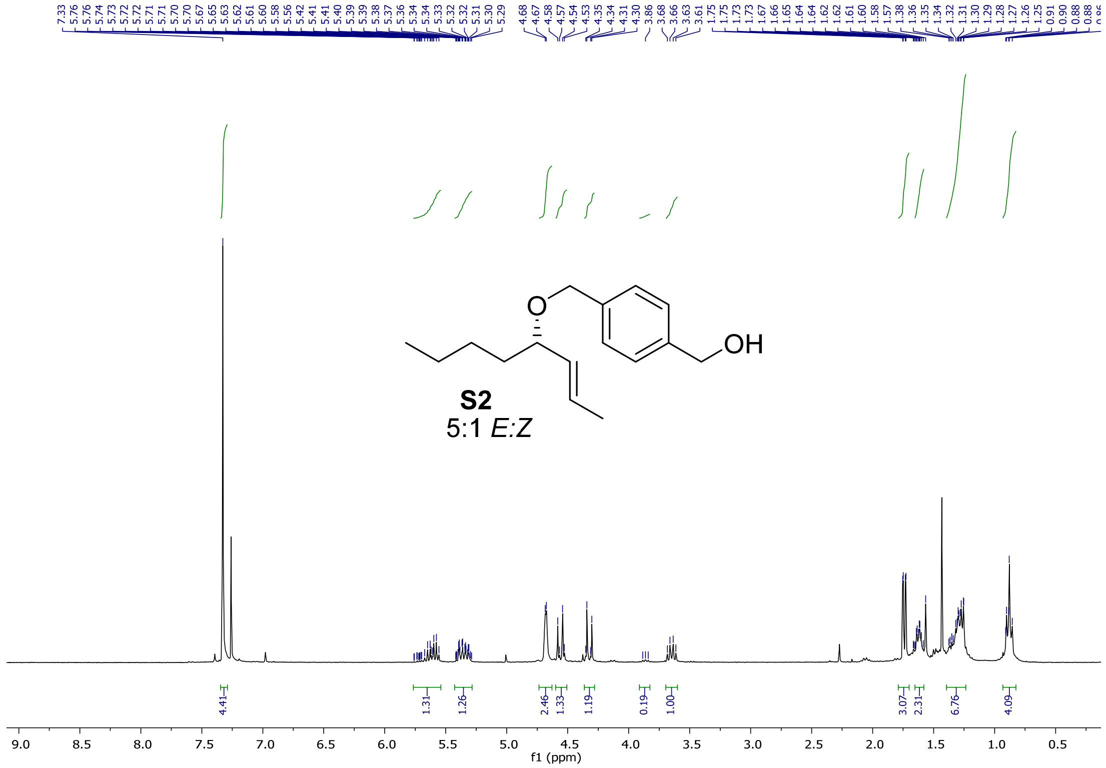

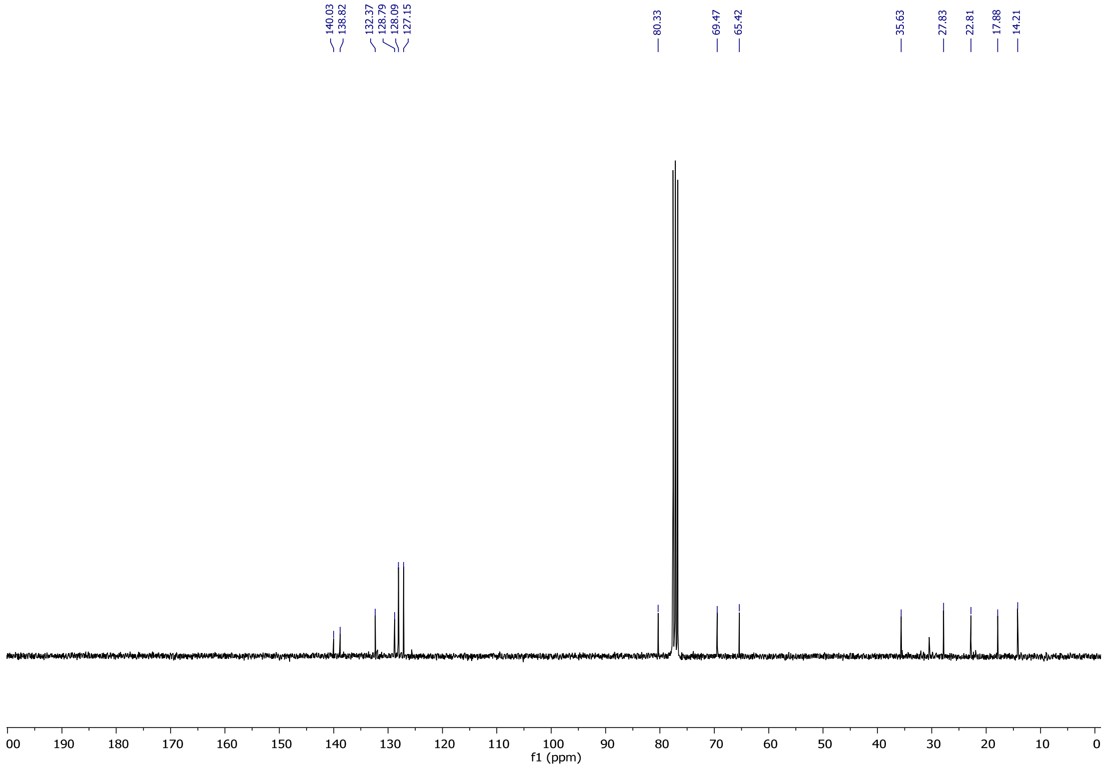

***Rac*-(*E*)-(4-((Oct-2-en-4-yloxy)methyl)phenyl)methanol S2 (*E*-Isomer)**

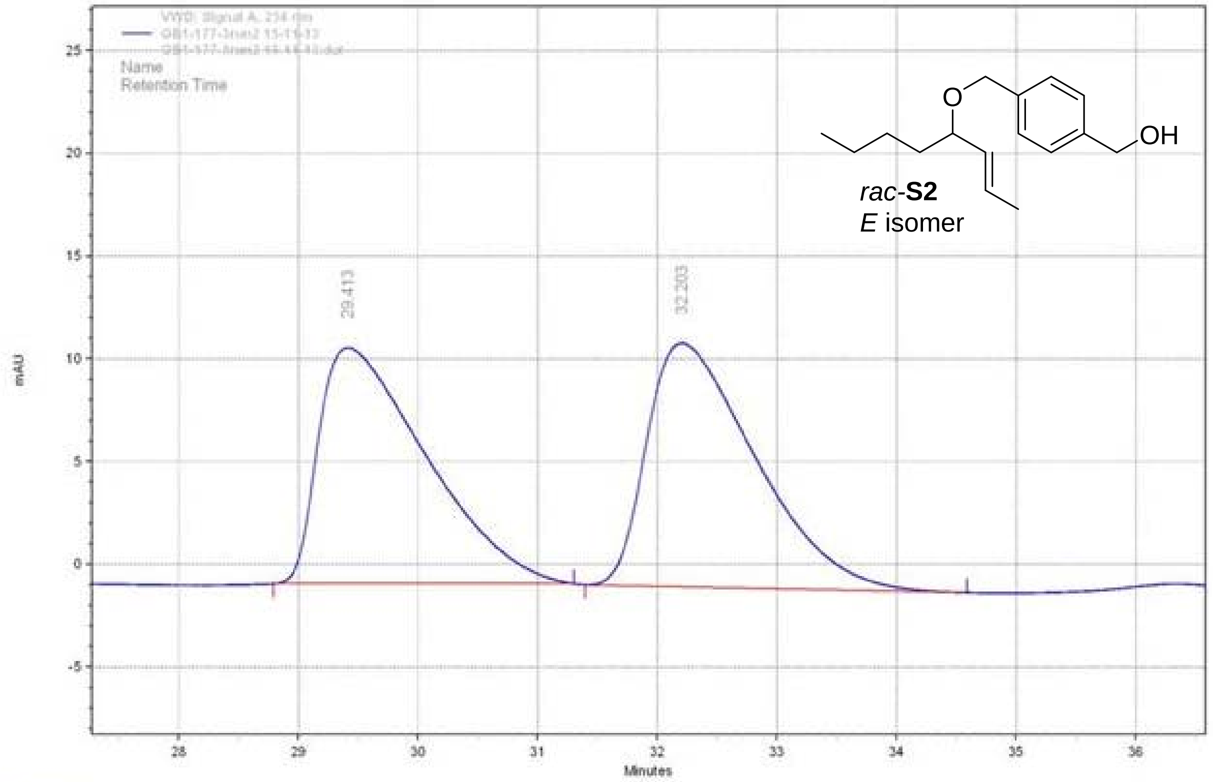

C:\EZChrom Elite\Enterprise\Projects\Default\Data\GB1-177-3run2 15-11-13.dat, VWD: Signal A, 254 nm

VWD: Signal A,
254 nm Results

Retention Time	Area	Area %	Height	Height %
29.413	11969246	48.81	192697	49.20
32.203	12554571	51.19	198933	50.80

***Rac*-(*Z*)-(4-((Oct-2-en-4-yloxy)methyl)phenyl)methanol (*Z*-Isomer)**

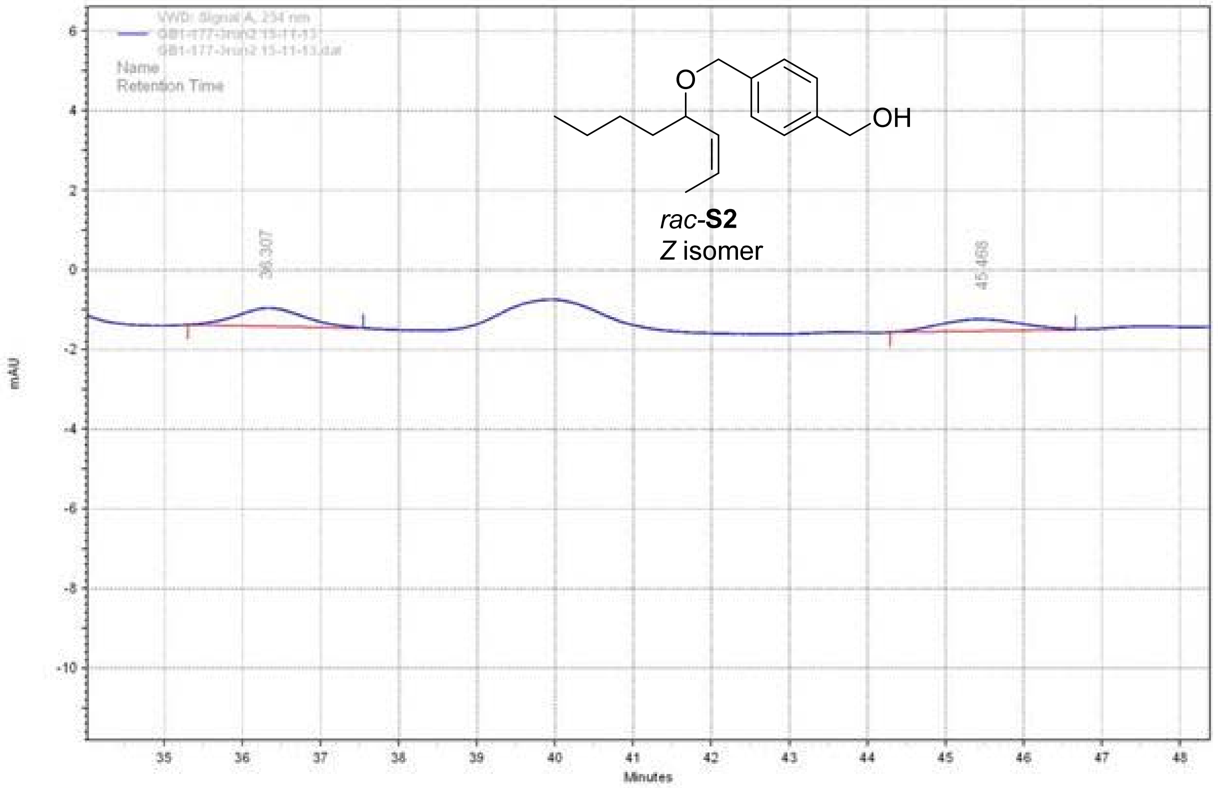

C:\EZChrom Elite\Enterprise\Projects\Default\Data\GB1-177-3run2 15-11-13.dat, VWD: Signal A, 254 nm

VWD: Signal A, 254 nm Results

Retention Time	Area	Area %	Height	Height %
36.307	462353	56.29	7625	60.44
45.468	359015	43.71	4991	39.56

(*S*,*E*)-(4-((Oct-2-en-4-yloxy)methyl)phenyl)methanol

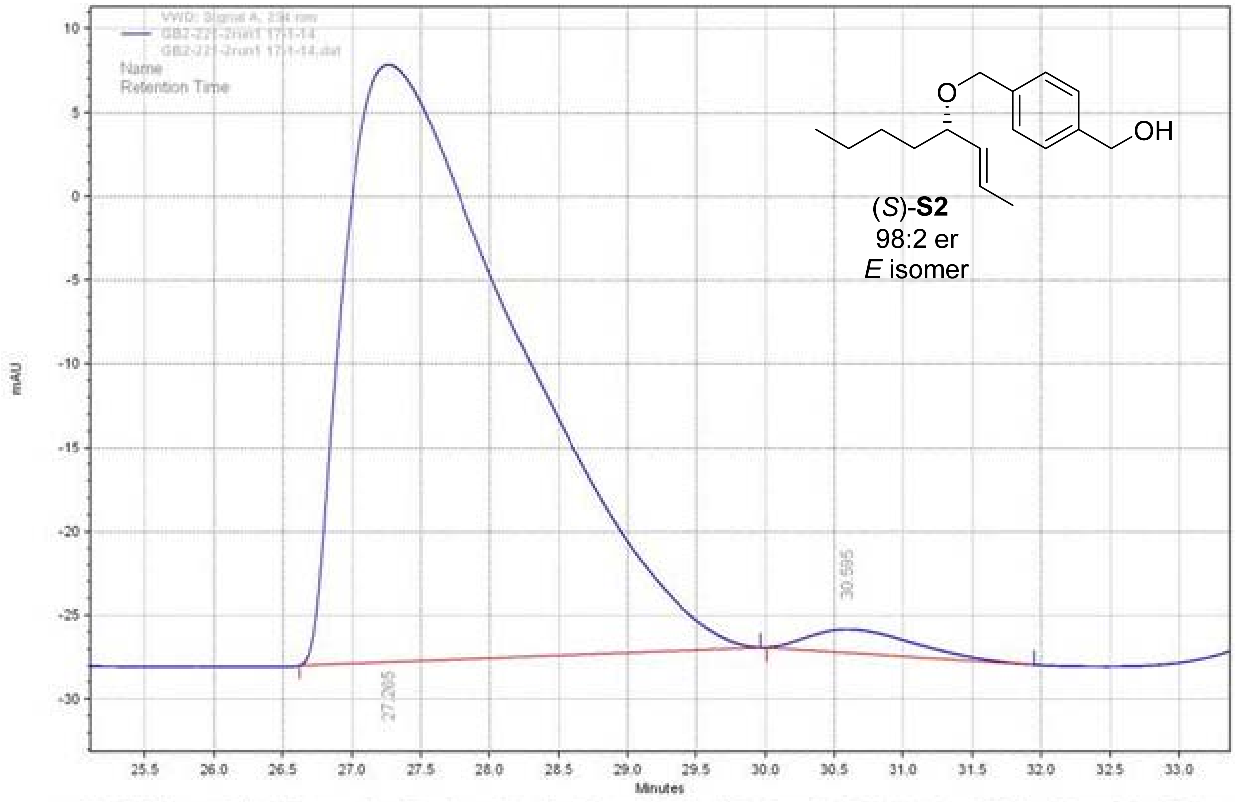

C:\EZChrom Elite\Enterprise\Projects\Default\Data\GB2-221-2run1 17-1-14.dat, VWD: Signal A, 254 nm

VWD: Signal A, 254 nm Results

Retention Time	Area	Area %	Height	Height %
27.265	52122689	97.68	597426	96.28
30.595	1235736	2.32	23101	3.72

This is the derivative to determine the er of the *E* isomer of **5cb**.

(*S*,*Z*)-(4-((Oct-2-en-4-yloxy)methyl)phenyl)methanol

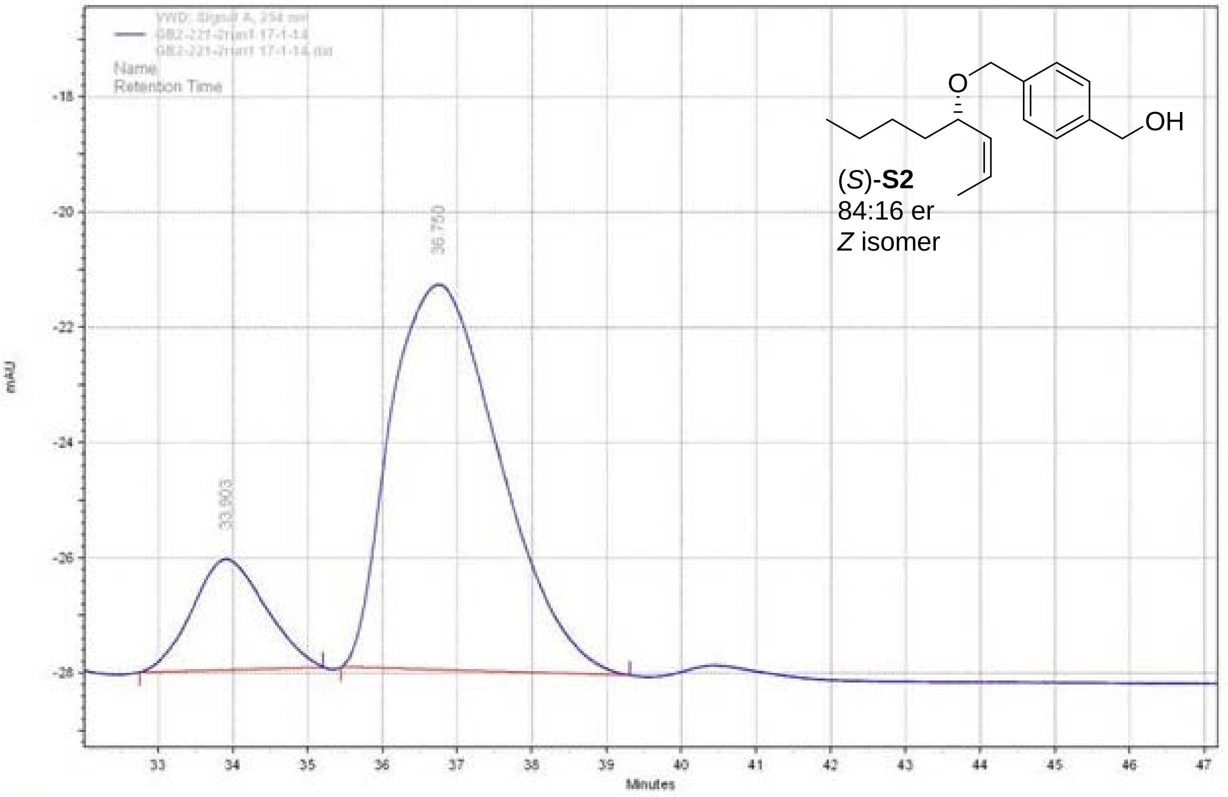

C:\EZChrom Elite\Enterprise\Projects\Default\Data\GB2-221-2run1 17-1-14.dat, VWD: Signal A, 254 nm

VWD: Signal A, 254 nm Results

Retention Time	Area	Area %	Height	Height %
33.903	2177258	16.05	32167	22.30
36.750	11385465	83.95	112064	77.70

This is the derivative to determine the er of the *Z* isomer of **5cb**.

(*S*, *E*)-Methyl 4-((4-cyclohexylbut-3-en-2-yloxy)methyl)benzoate (*S*)-5db

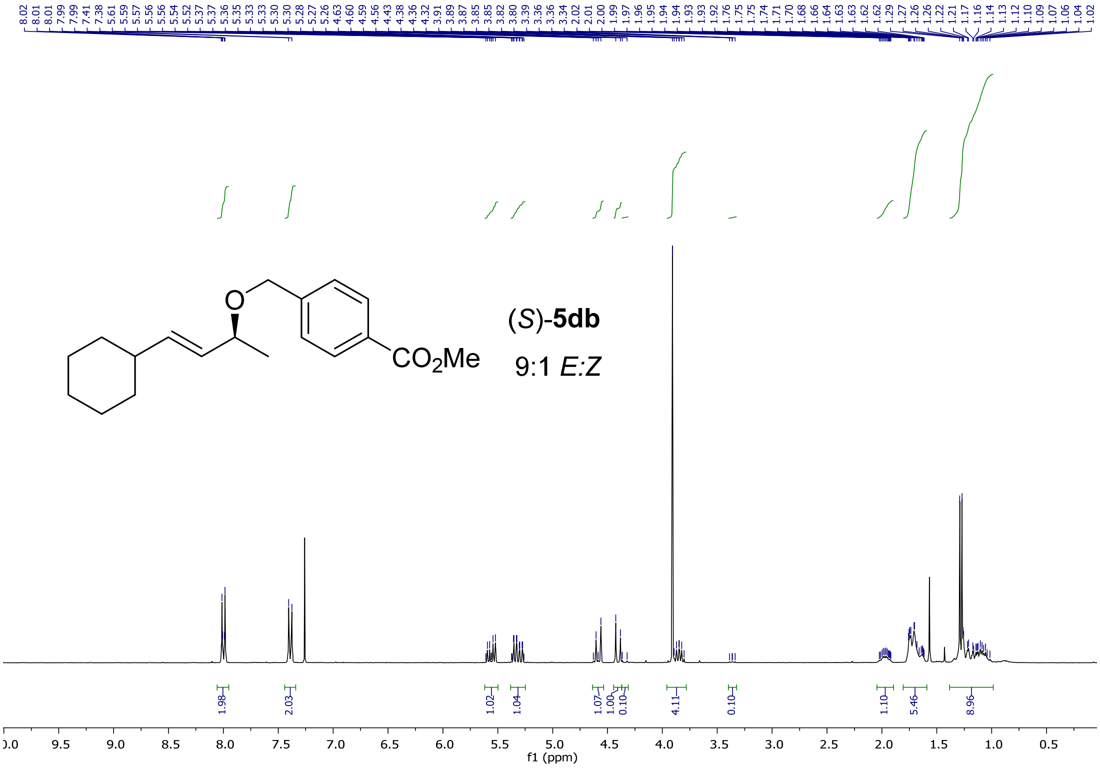

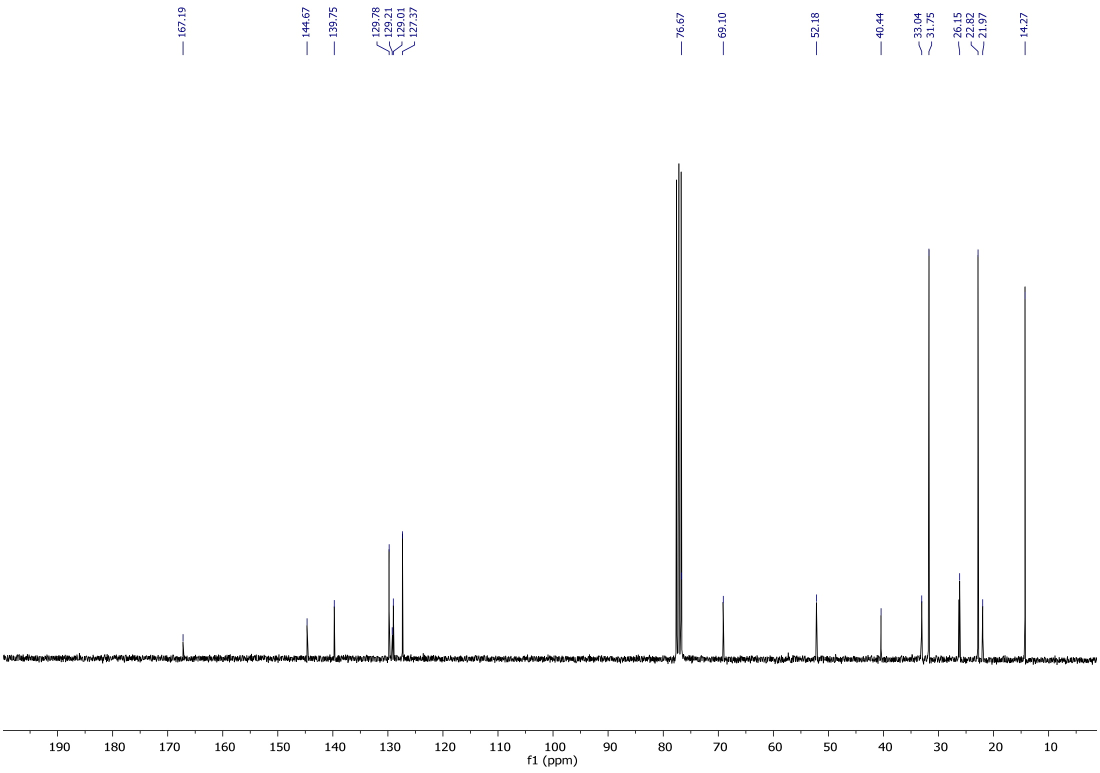

***Rac*-(*E*)-Methyl 4-((4-cyclohexylbut-3-en-2-yloxy)methyl)benzoate *rac*-5db**

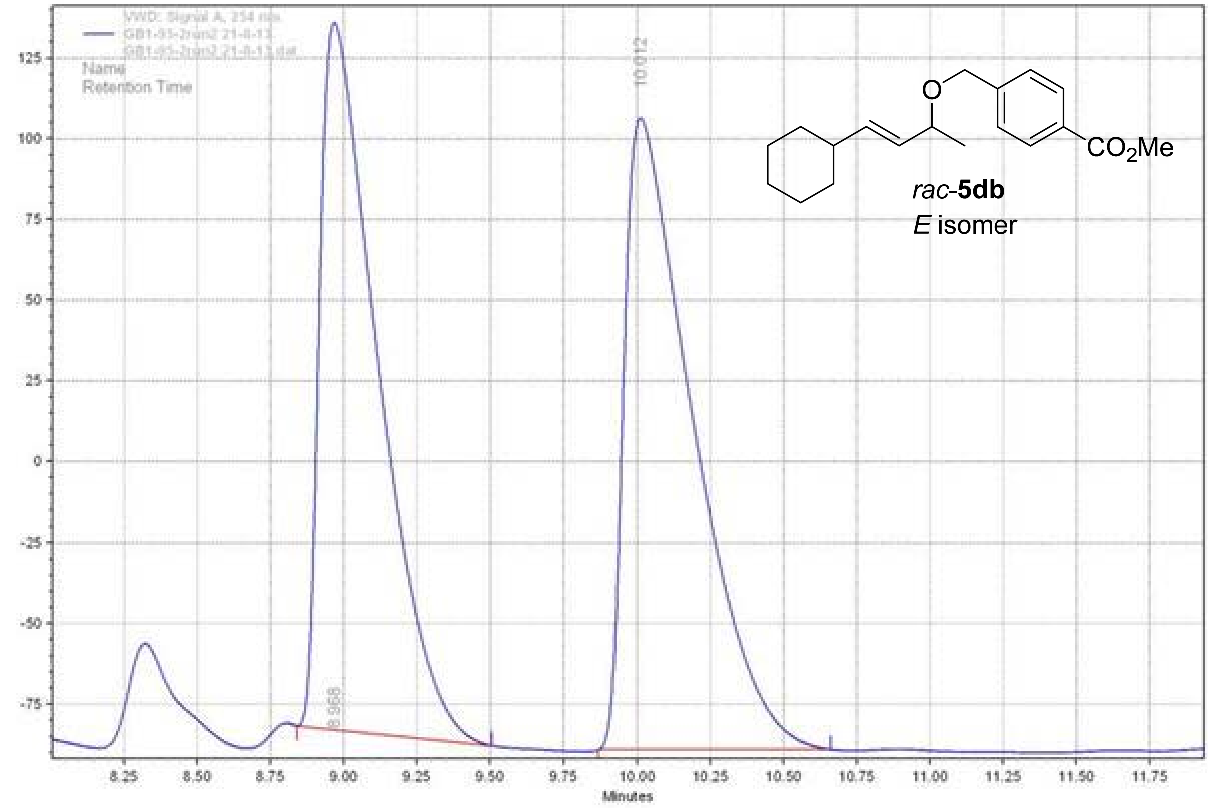

C:\EZChrom Elite\Enterprise\Projects\Default\Data\GB1-95-2run2 21-8-13.dat, VWD: Signal A, 254 nm

VWD: Signal A, 254 nm Results

Retention Time	Area	Area %	Height	Height %
8.968	50872808	48.78	3670718	52.82
10.012	53422575	51.22	3278547	47.18

***Rac*-(*Z*)-Methyl 4-((4-cyclohexylbut-3-en-2-yloxy)methyl)benzoate *rac*-5db (minor Z isomer)**

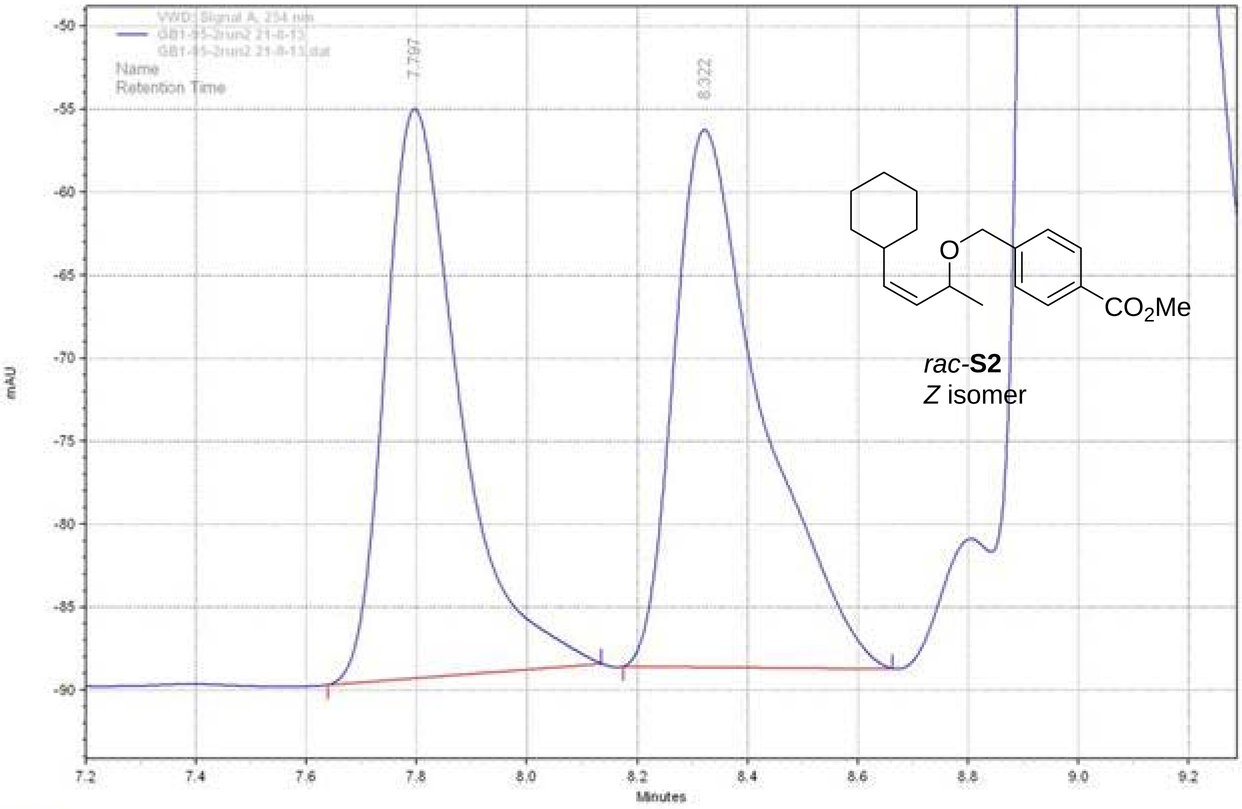

C:\EZChrom Elite\Enterprise\Projects\Default\Data\GB1-95-2run2 21-8-13.dat, VWD: Signal A, 254 nm

VWD: Signal A, 254 nm Results

Retention Time	Area	Area %	Height	Height %
7.797	5422228	47.34	575294	51.45
8.322	6031347	52.66	542841	48.55

(*S*, *E*)-Methyl 4-((4-cyclohexylbut-3-en-2-yloxy)methyl)benzoate (*S*)-5db

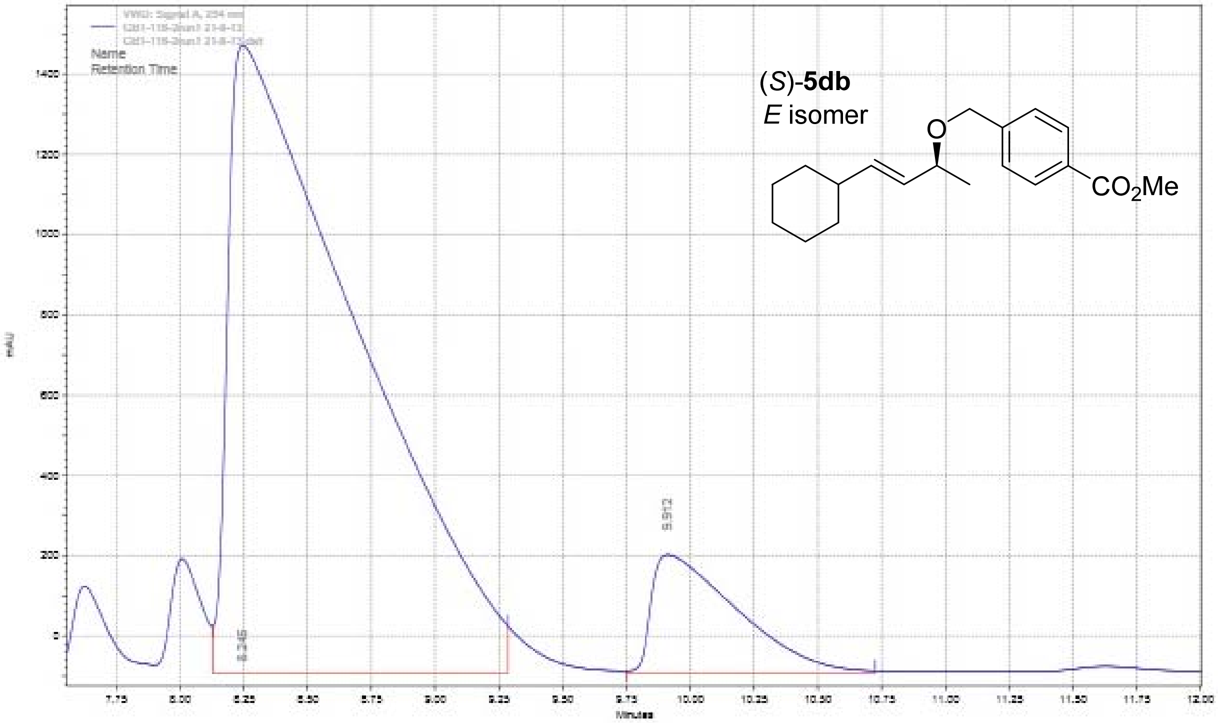

C:\EZChrom Elite\Enterprise\Projects\Default\Data\GB1-116-2run1 21-8-13.dat, VWD: Signal A, 254 nm

VWD: Signal A,
254 nm Results

Retention Time	Area	Area %	Height	Height %
8.245	927337739	89.00	26243609	84.17
9.912	114655826	11.00	4936292	15.83

(*S*, *Z*)-Methyl 4-((4-cyclohexylbut-3-en-2-yloxy)methyl)benzoate (*S*)-5db

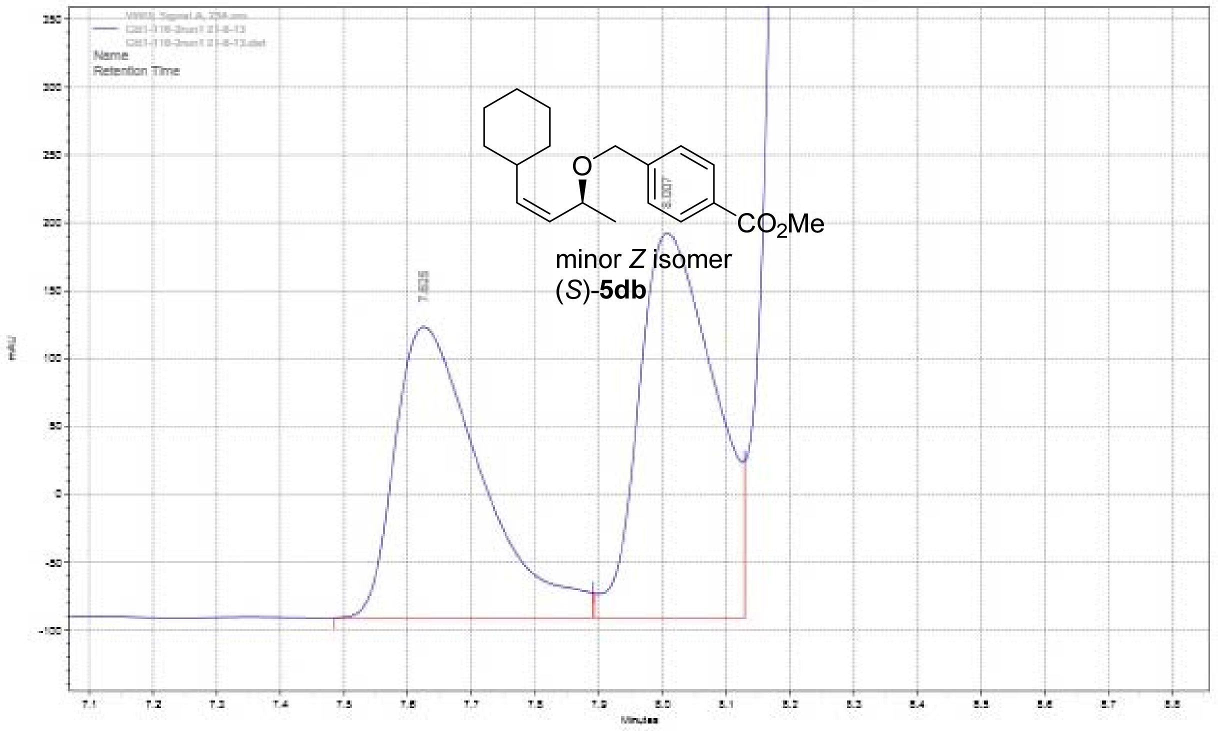

C:\EZChrom Elite\Enterprise\Projects\Default\Data\GB1-116-2run1 21-8-13.dat, VWD: Signal A, 254 nm

VWD: Signal A, 254 nm Results

Retention Time	Area	Area %	Height	Height %
7.625	33765701	46.42	3592344	43.05
8.007	38977525	53.58	4752131	56.95

(*S*, *E*)-Methyl 4-((4-cyclohexylbut-3-en-2-yloxy)methyl)benzoate (*S*)-5db after resubjection to catalytic conditions (Scheme 3, Eq. 1)

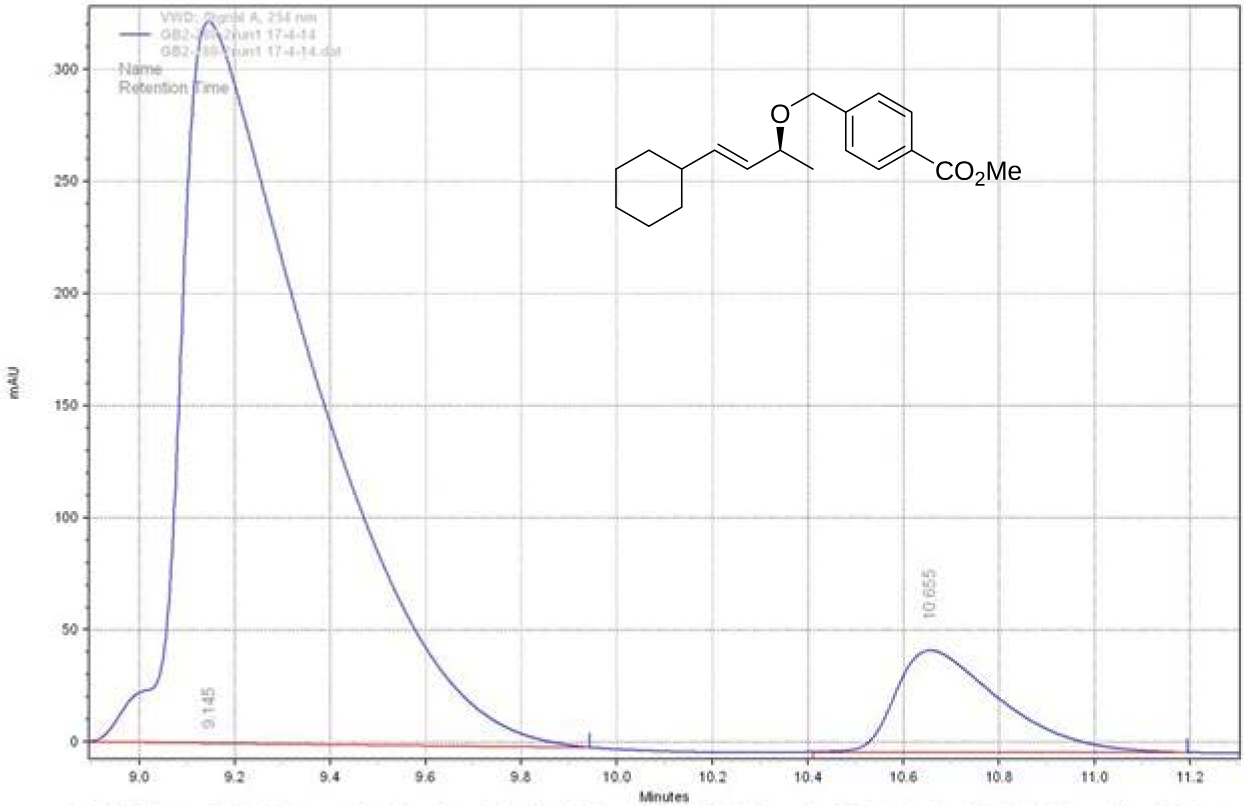

C:\EZChrom Elite\Enterprise\Projects\Default\Data\GB2-288-2run1 17-4-14.dat, VWD: Signal A, 254 nm

VWD: Signal A, 254 nm Results

Retention Time	Area	Area %	Height	Height %
9.145	104981506	90.09	5398055	87.64
10.655	11554521	9.91	761375	12.36

(*S*, *Z*)-Methyl 4-((4-cyclohexylbut-3-en-2-yloxy)methyl)benzoate (*S*)-5db after resubjection to catalytic conditions (Scheme 3, Eq. 1)

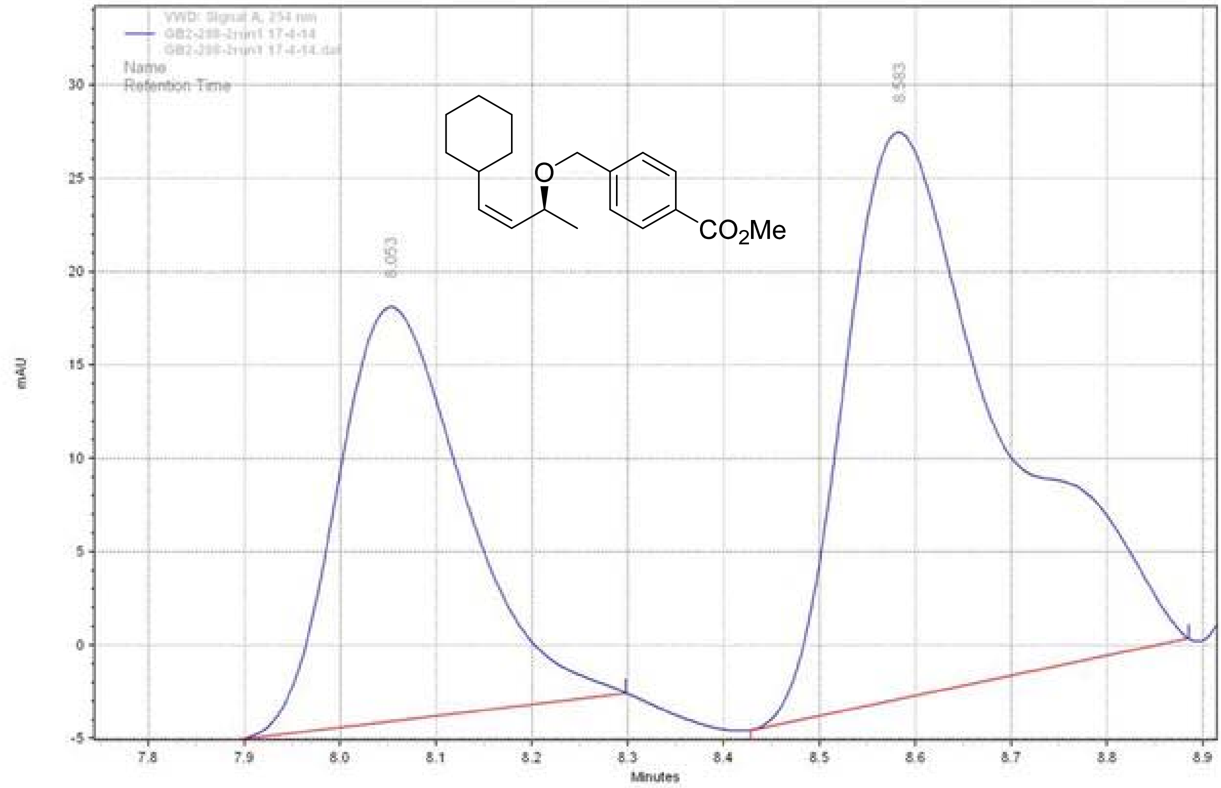

C:\EZChrom Elite\Enterprise\Projects\Default\Data\GB2-288-2run1 17-4-14.dat, VWD: Signal A, 254 nm

VWD: Signal A, 254 nm Results

Retention Time	Area	Area %	Height	Height %
8.053	3448618	37.42	372247	42.24
8.583	5766682	62.58	509029	57.76

(*S*, *E*)-Methyl 4-((4-cyclohexylbut-3-en-2-yloxy)methyl)benzoate (*S*)-5db (Scheme 4)

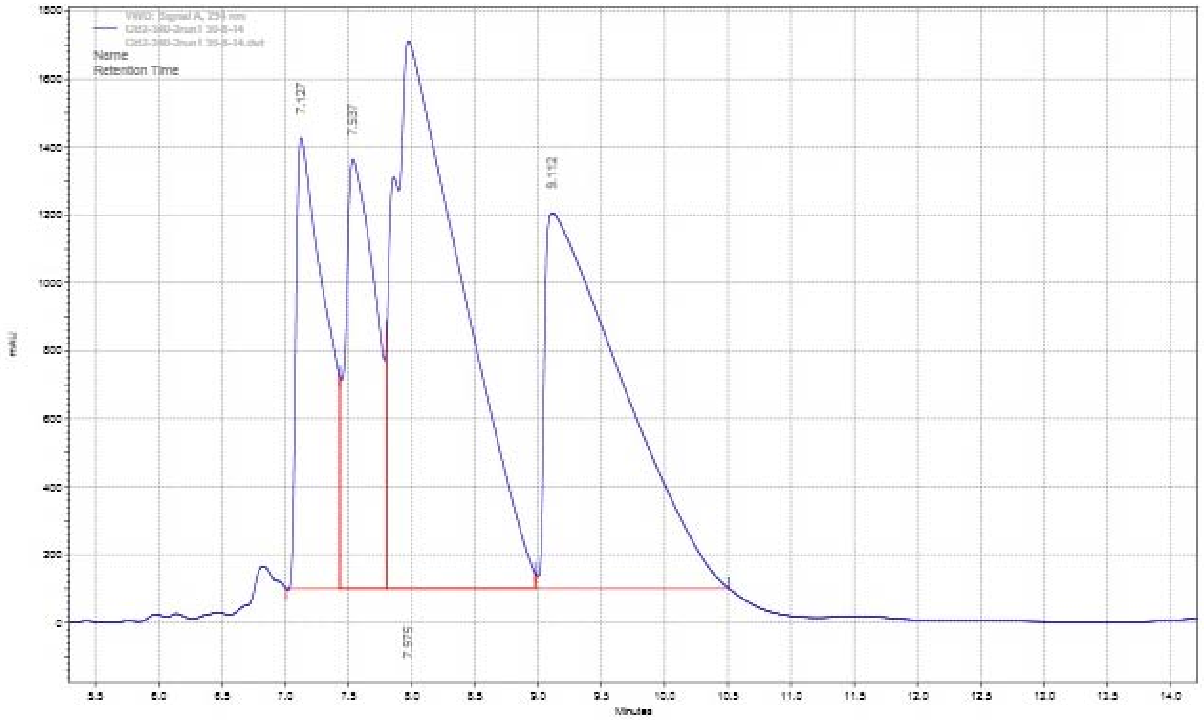

C:\EZChrom Elite\Enterprise\Projects\Default\Data\GB2-340-2run1 30-6-14.dat, VWD: Signal A, 254 nm

VWD: Signal A,
254 nm Results

Retention Time	Area	Area %	Height	Height %
7.127	353034093	14.09	22248321	24.99
7.537	358749121	14.32	21189045	23.80
7.975	1010085820	40.31	27038582	30.37
9.112	784181074	31.29	18557080	20.84

(*S*, *E*)-Methyl 4-((4-cyclohexylbut-3-en-2-yloxy)methyl)benzoate (*S*)-5db (Scheme 7, Eq. 1)

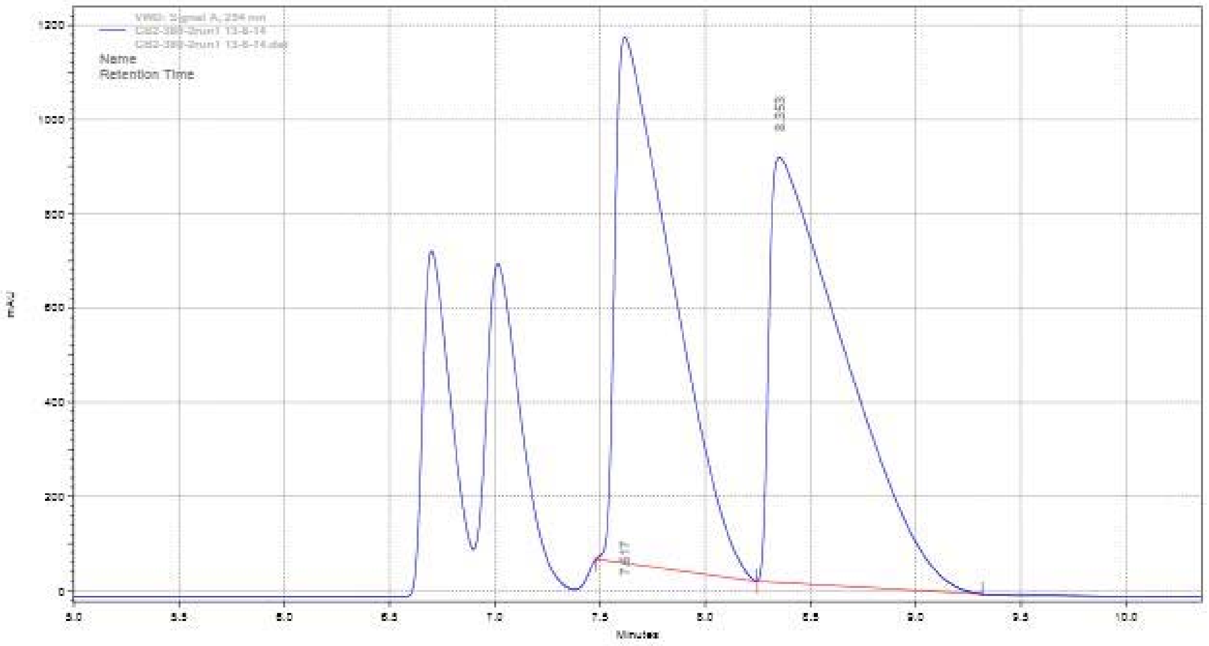

C:\EZChrom Elite\Enterprise\Projects\Default\Data\GB2-380-2run1 13-8-14.dat, VWD: Signal A, 254 nm

VWD: Signal A,
254 nm Results

Retention Time	Area	Area %	Height	Height %
7.617	354462075	48.33	18714946	55.29
8.353	378999458	51.67	15133383	44.71

(*S*, *Z*)-Methyl 4-((4-cyclohexylbut-3-en-2-yloxy)methyl)benzoate (*S*)-5db (Scheme 7, Eq. 1)

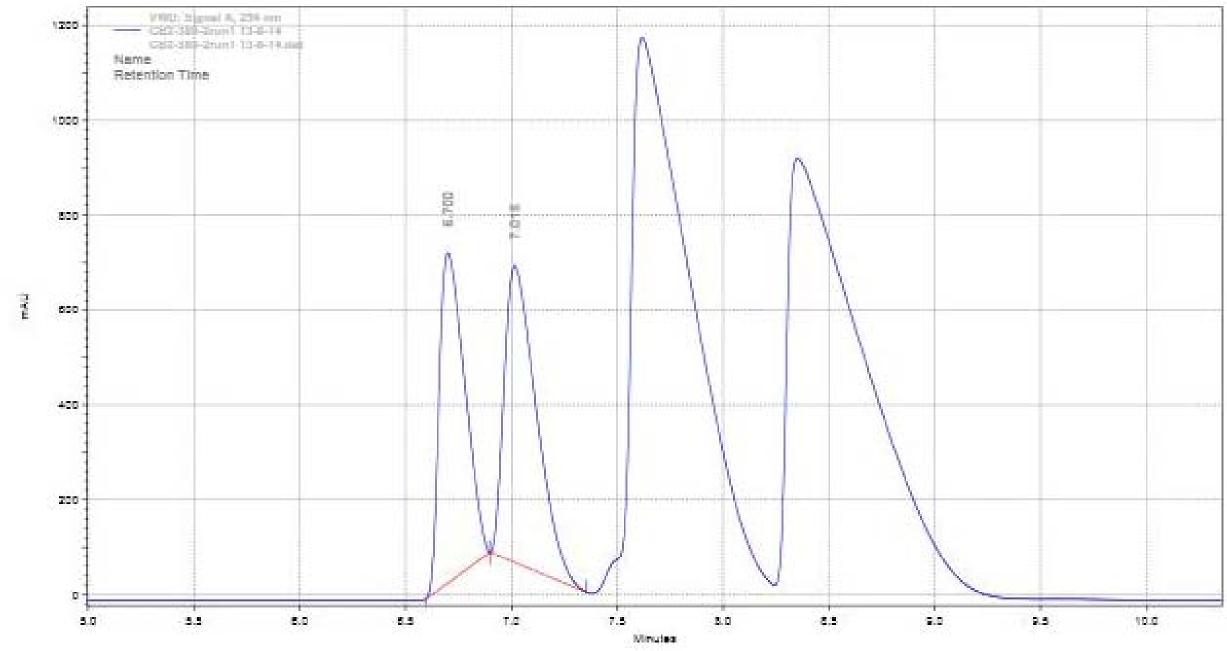

C:\EZChrom Elite\Enterprise\Projects\Default\Data\GB2-380-2run1 13-8-14.dat, VWD: Signal A, 254 nm

VWD: Signal A,
254 nm Results

Retention Time	Area	Area %	Height	Height %
6.700	97665895	46.85	11668391	52.64
7.015	110820023	53.15	10496363	47.36

(*S*, *E*)-Methyl 4-((1-cyclohexylbut-2-enyloxy)methyl)benzoate (*S*)-5eb

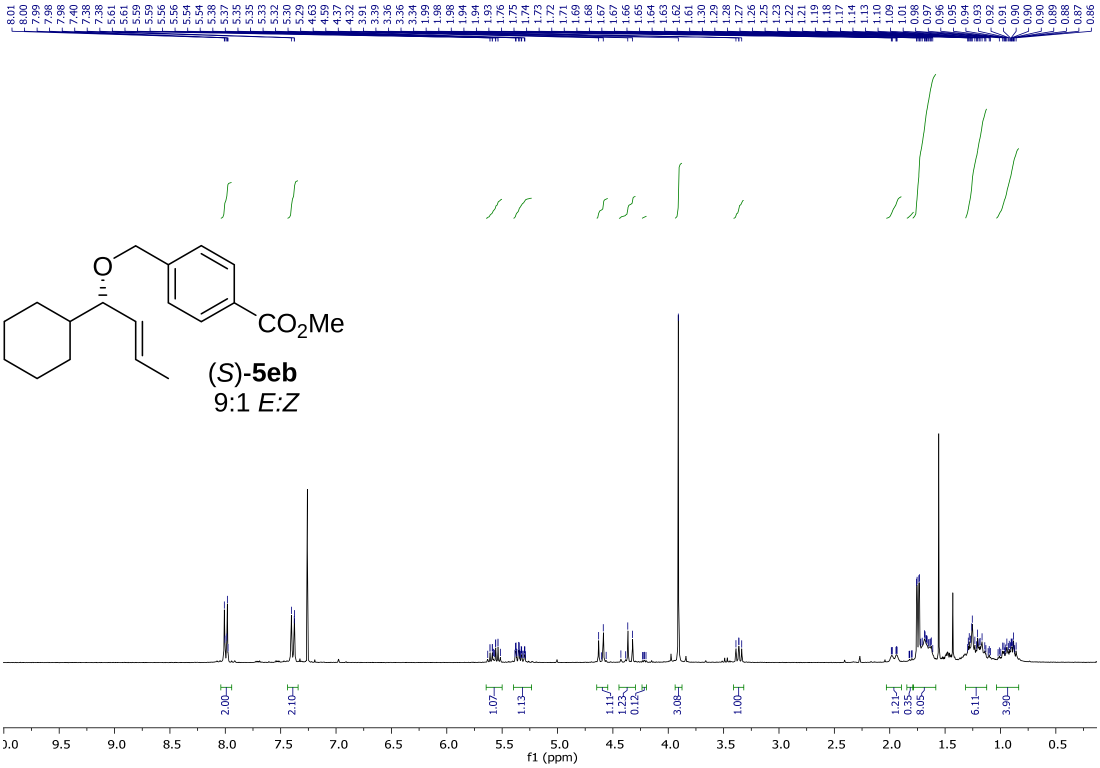

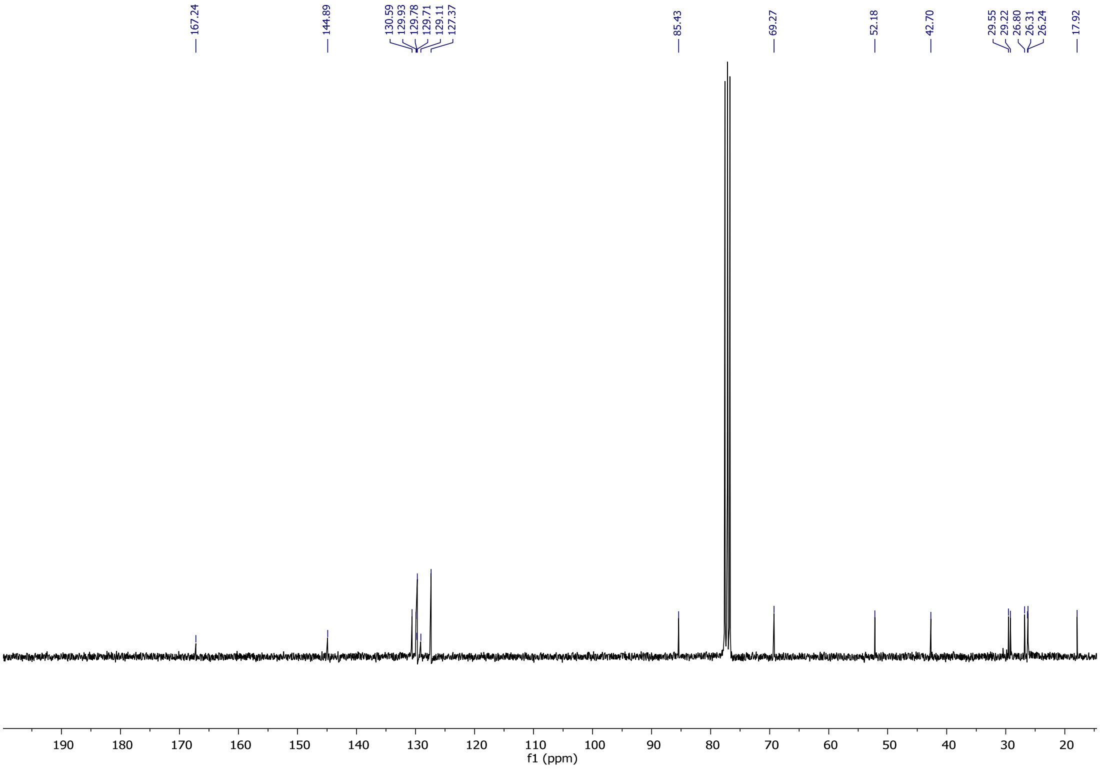

***Rac*-(*E*)-Methyl 4-((1-cyclohexylbut-2-enyloxy)methyl)benzoate *rac*-5eb**

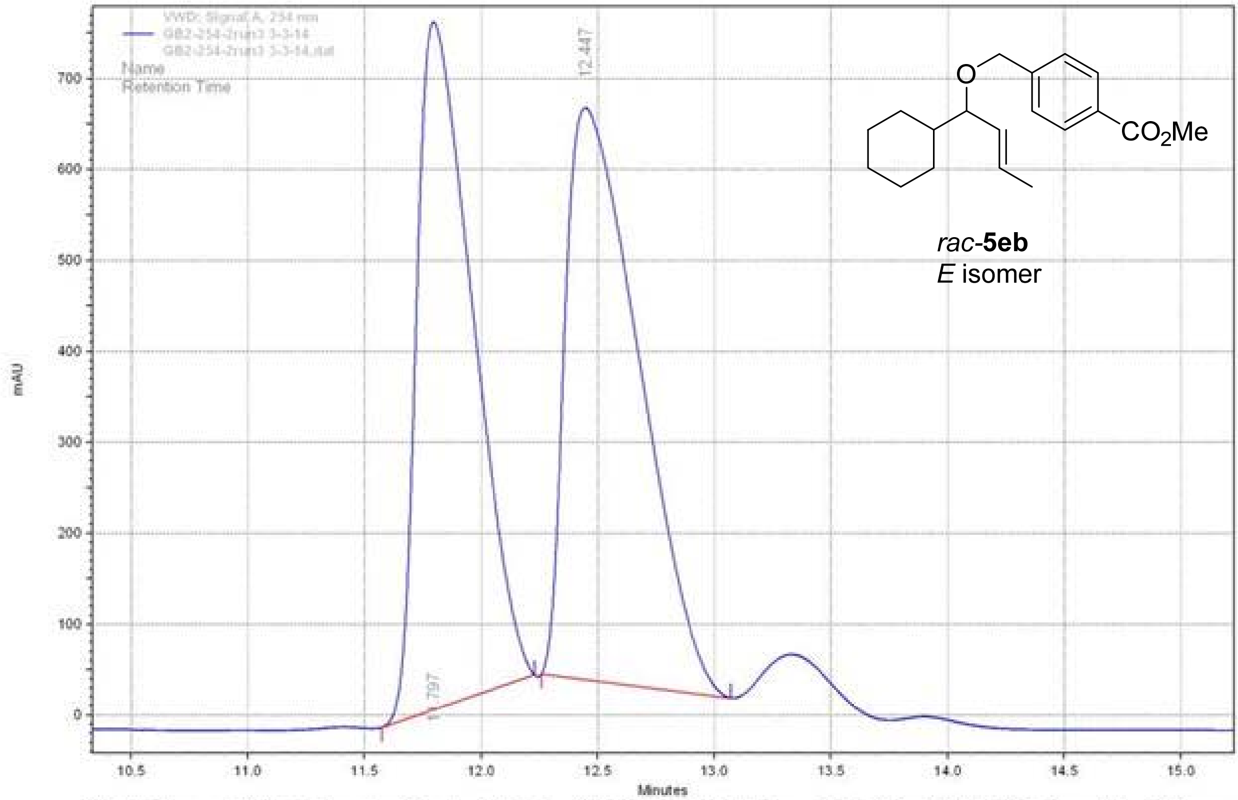

C:\EZChrom Elite\Enterprise\Projects\Default\Data\GB2-254-2run3 3-3-14.dat, VWD: Signal A, 254 nm

VWD: Signal A,
254 nm Results

Retention Time	Area	Area %	Height	Height %
11.797	211688387	47.74	12673227	54.59
12.447	231773710	52.26	10541836	45.41

***Rac*-(*Z*)-Methyl 4-((1-cyclohexylbut-2-enyloxy)methyl)benzoate *rac*-5eb**

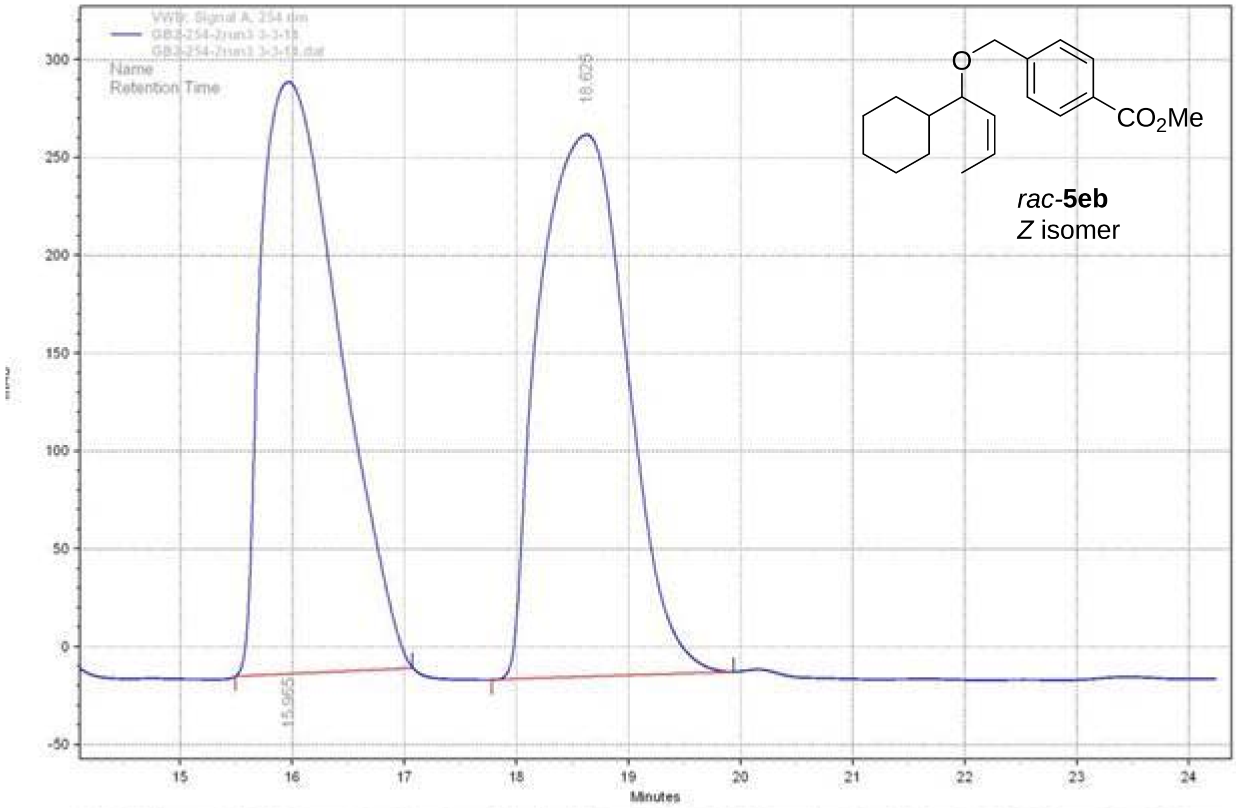

C:\EZChrom Elite\Enterprise\Projects\Default\Data\GB2-254-2run3 3-3-14.dat, VWD: Signal A, 254 nm

VWD: Signal A,
254 nm Results

Retention Time	Area	Area %	Height	Height %
15.965	246043934	49.29	5075534	52.20
18.625	253137579	50.71	4647337	47.80

(*S*, *E*)-Methyl 4-((1-cyclohexylbut-2-enyloxy)methyl)benzoate (*S*)-5eb

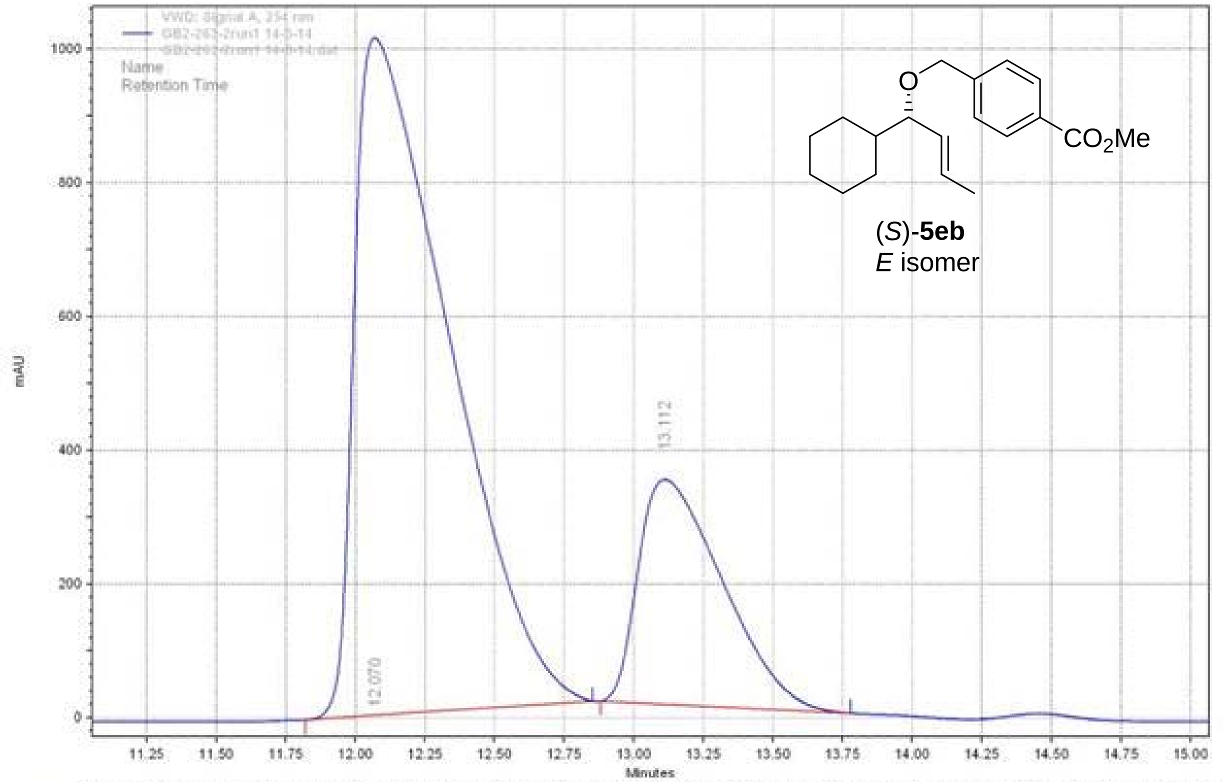

C:\EZChrom Elite\Enterprise\Projects\Default\Data\GB2-263-2run1 14-3-14.dat, VWD: Signal A, 254 nm

VWD: Signal A, 254 nm Results

Retention Time	Area	Area %	Height	Height %
12.070	401544555	77.15	16997230	75.09
13.112	118949263	22.85	5637391	24.91

(*S*, *Z*)-Methyl 4-((1-cyclohexylbut-2-enyloxy)methyl)benzoate (*S*)-5eb

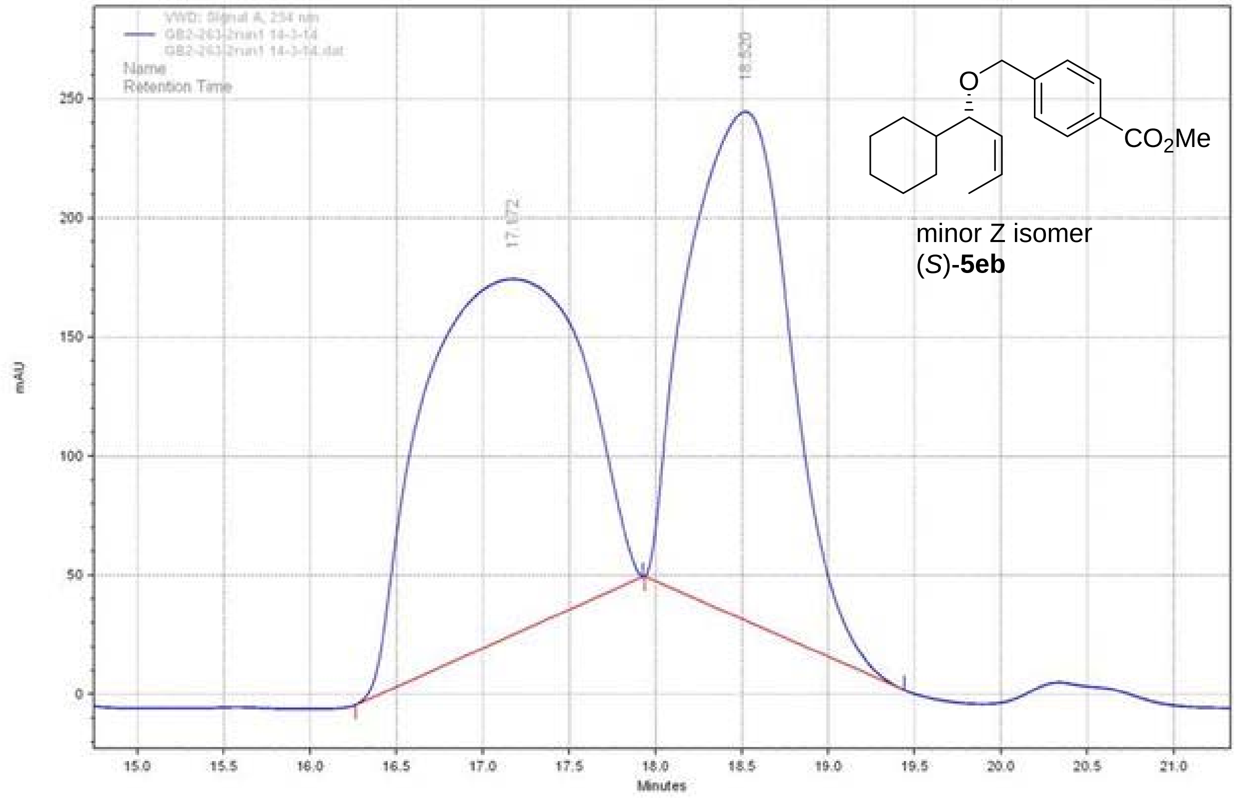

C:\EZChrom Elite\Enterprise\Projects\Default\Data\GB2-263-2run1 14-3-14.dat, VWD: Signal A, 254 nm

VWD: Signal A, 254 nm Results

Retention Time	Area	Area %	Height	Height %
17.172	164792911	52.76	2506088	41.13
18.520	147570555	47.24	3586529	58.87

(*R*, *E*)-Methyl 4-((2-methylhex-4-en-3-yloxy)methyl)benzoate (*R*)-5fb

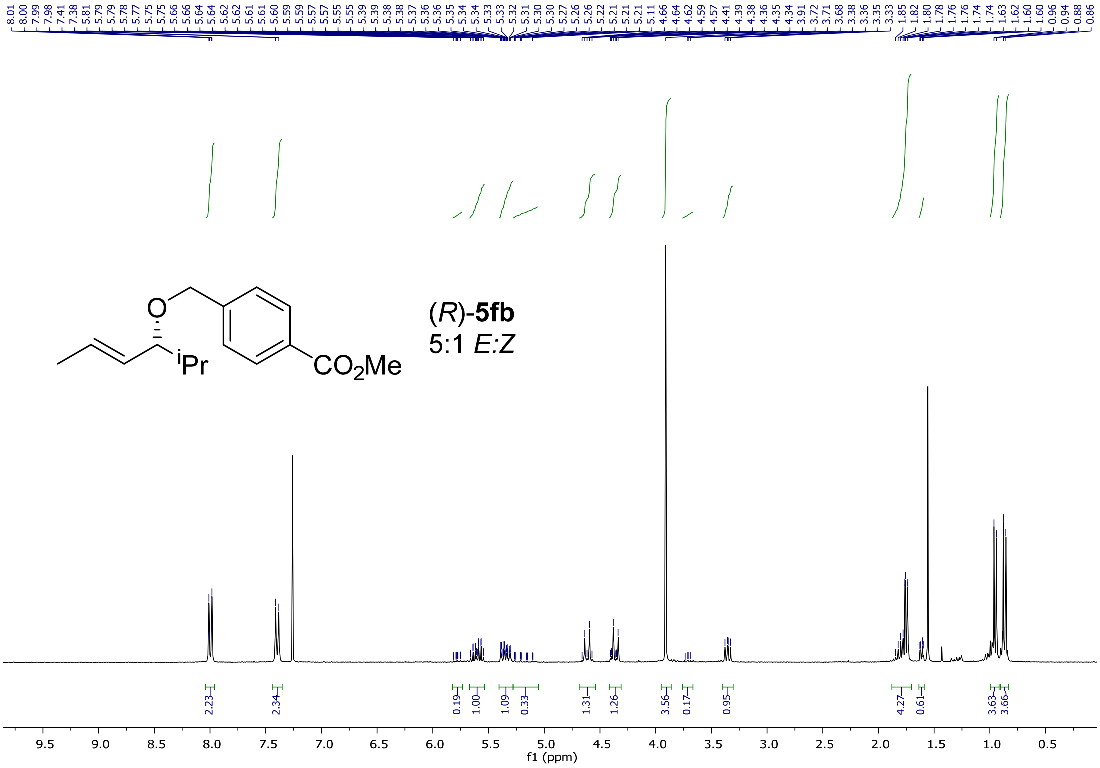

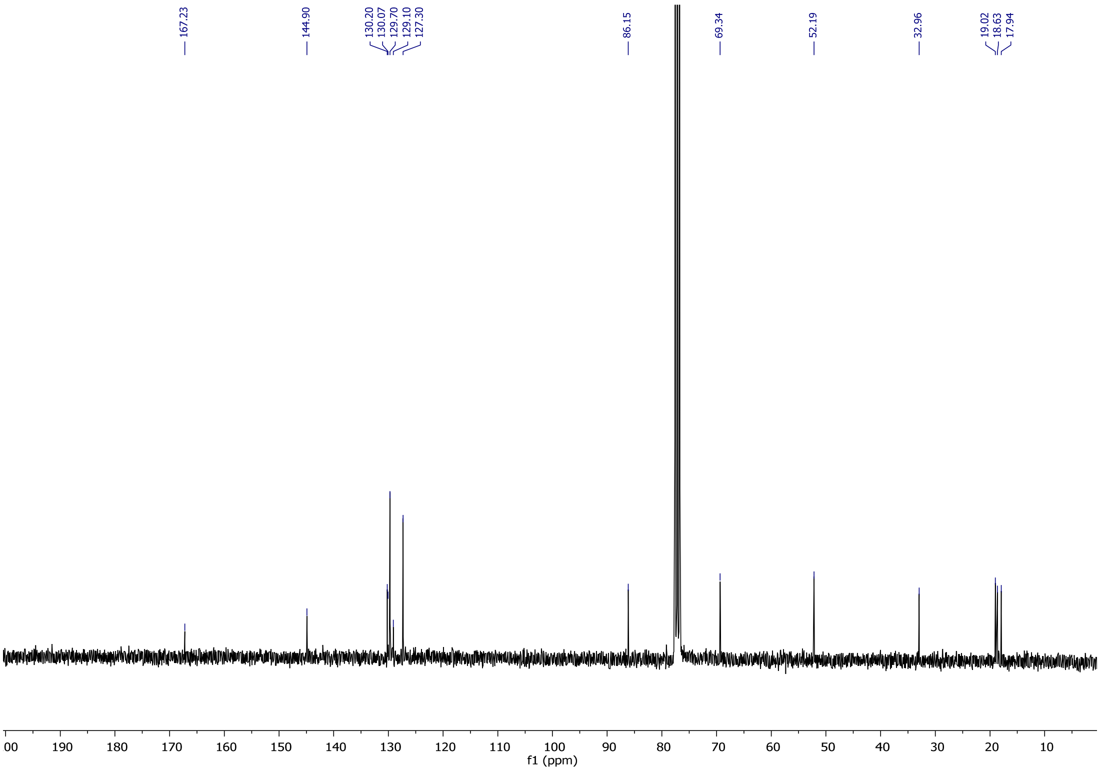

***Rac*-(*E*)-Methyl 4-((2-methylhex-4-en-3-yloxy)methyl)benzoate *rac*-5fb**

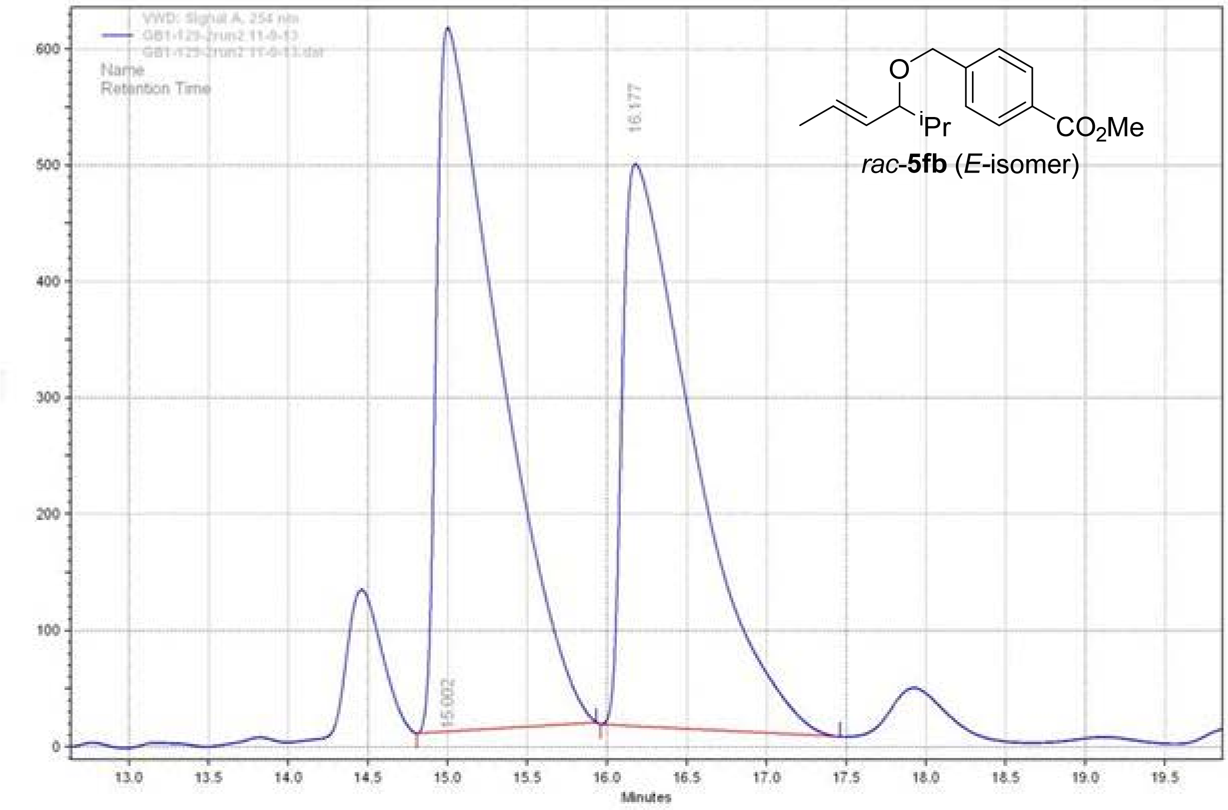

C:\EZChrom Elite\Enterprise\Projects\Default\Data\GB1-129-2run2 11-9-13.dat, VWD: Signal A, 254 nm

VWD: Signal A, 254 nm Results

Retention Time	Area	Area %	Height	Height %
15.002	278859011	52.67	10153832	55.61
16.177	250547612	47.33	8105740	44.39

***Rac*-(*Z*)-Methyl 4-((2-methylhex-4-en-3-yloxy)methyl)benzoate *rac*-5fb**

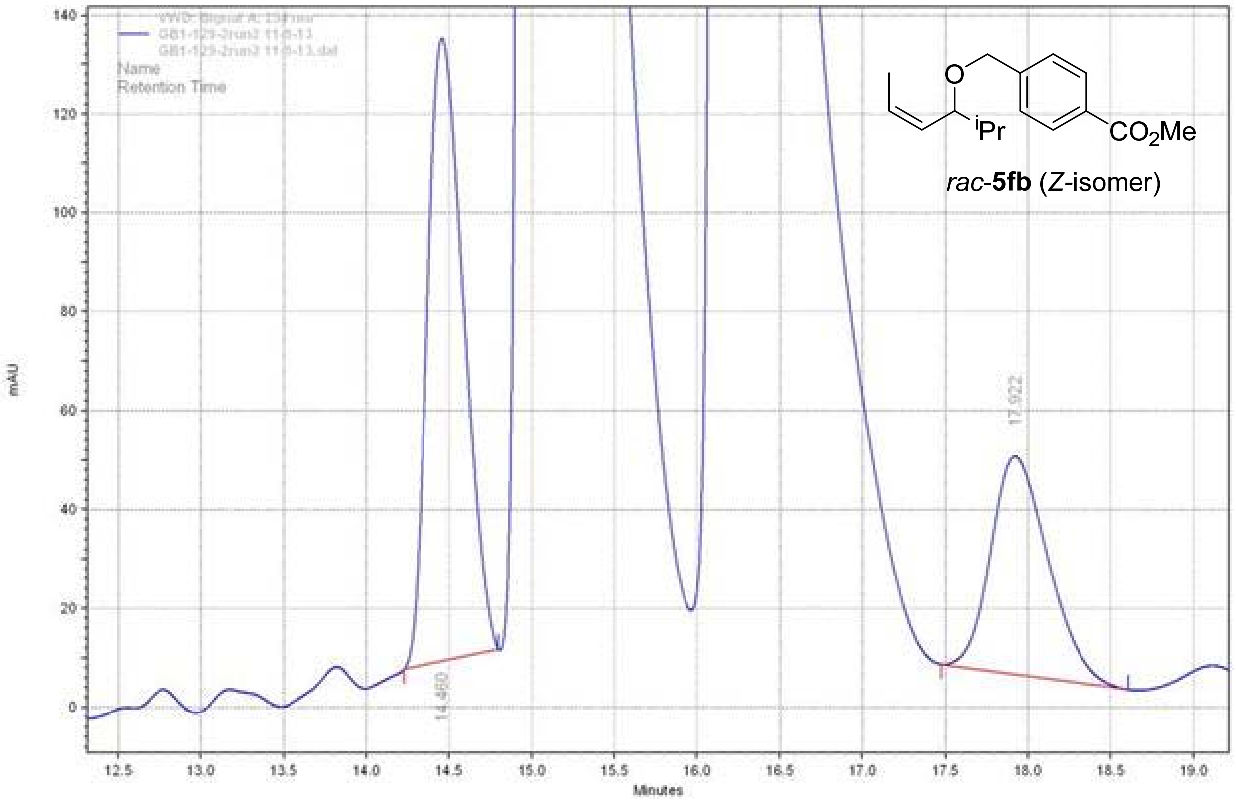

C:\EZChrom Elite\Enterprise\Projects\Default\Data\GB1-129-2run2 11-9-13.dat, VWD: Signal A, 254 nm

VWD: Signal A,
254 nm Results

Retention Time	Area	Area %	Height	Height %
14.460	31996008	64.23	2111308	74.07
17.922	17815020	35.77	738987	25.93

(*R*, *E*)-Methyl 4-((2-methylhex-4-en-3-yloxy)methyl)benzoate (*R*)-5fb

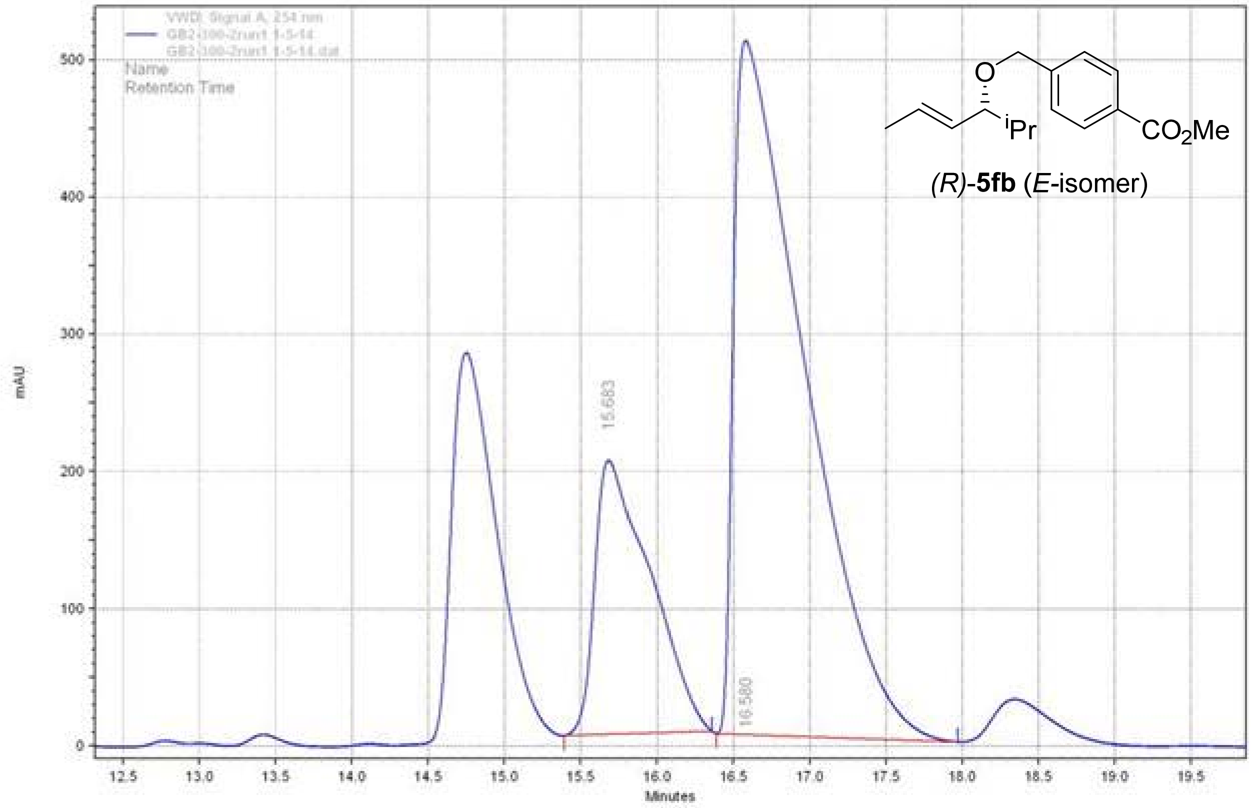

C:\EZChrom Elite\Enterprise\Projects\Default\Data\GB2-300-2run1 1-5-14.dat, VWD: Signal A, 254 nm

VWD: Signal A, 254 nm Results

Retention Time	Area	Area %	Height	Height %
15.683	84338116	23.38	3345520	28.28
16.580	276365377	76.62	8482652	71.72

(*R*, *Z*)-Methyl 4-((2-methylhex-4-en-3-yloxy)methyl)benzoate (*Z*)-5fb

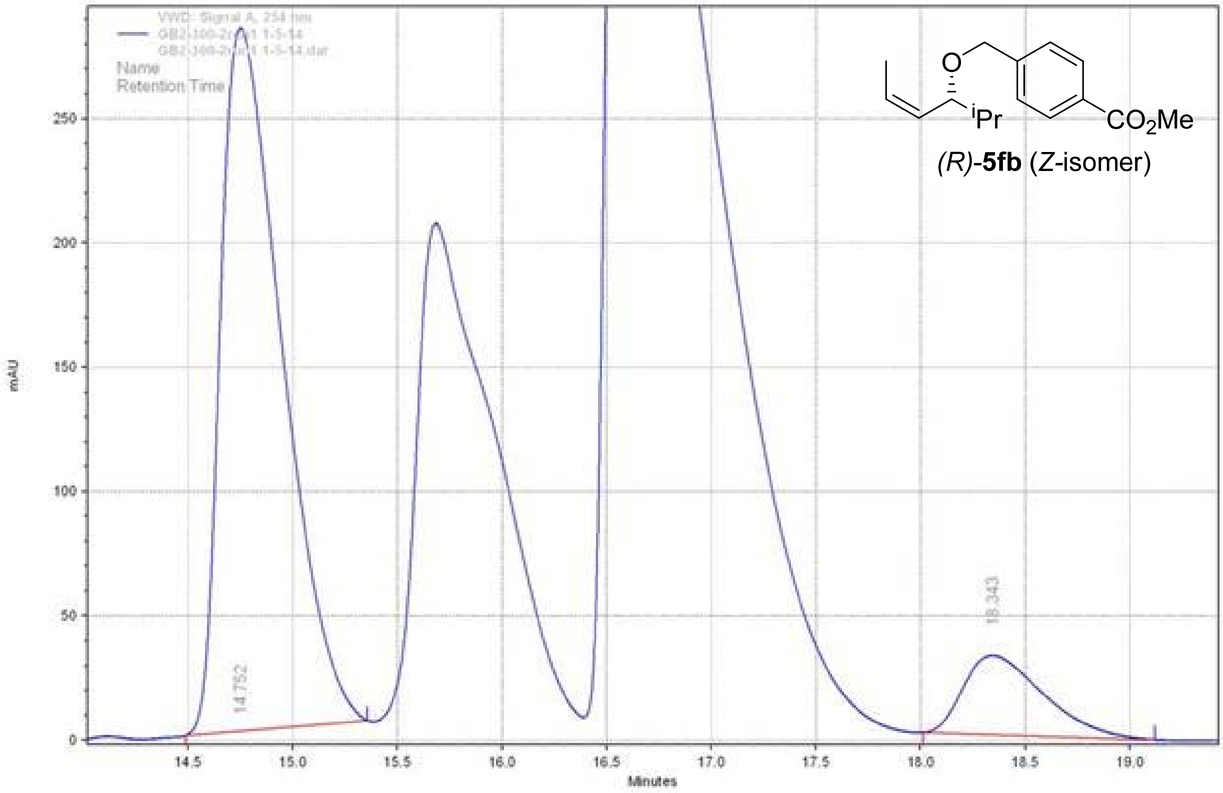

C:\EZChrom Elite\Enterprise\Projects\Default\Data\GB2-300-2run1 1-5-14.dat, VWD: Signal A, 254 nm

VWD: Signal A, 254 nm Results

Retention Time	Area	Area %	Height	Height %
14.752	97055923	87.33	4743638	89.89
18.343	14084661	12.67	533767	10.11

(*S*, *E*)-Methyl 4-((5-phenylpent-3-en-2-yloxy)methyl)benzoate (*S*)-5gb

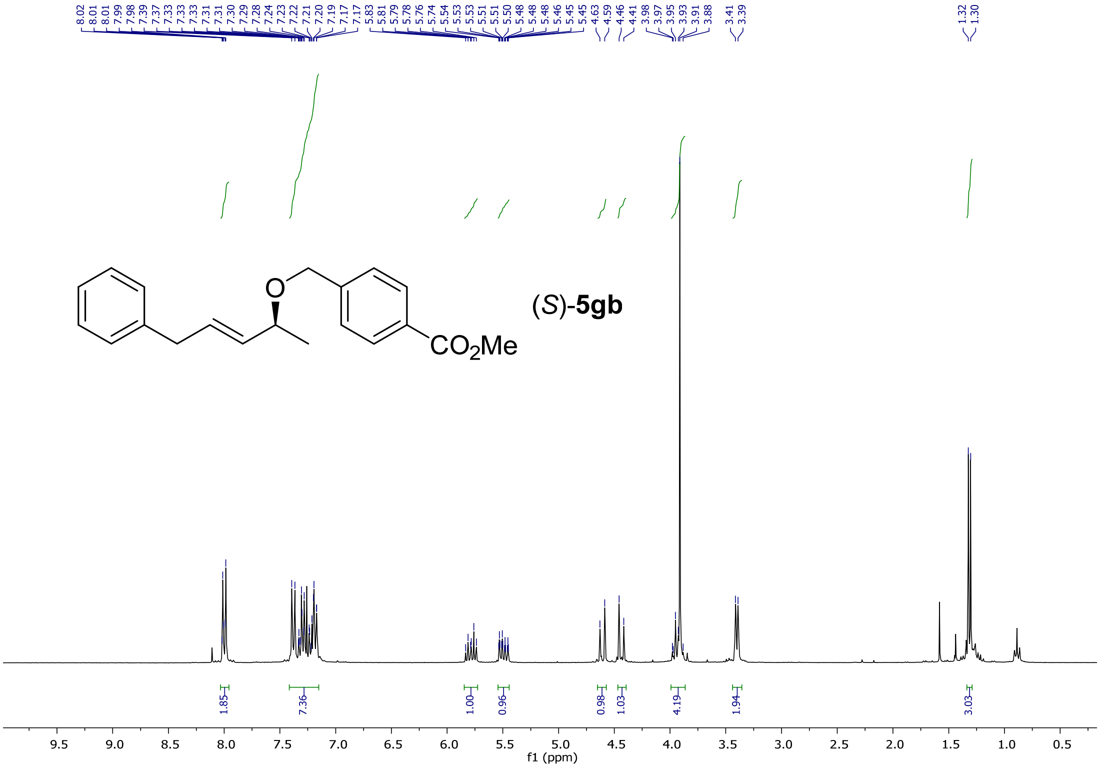

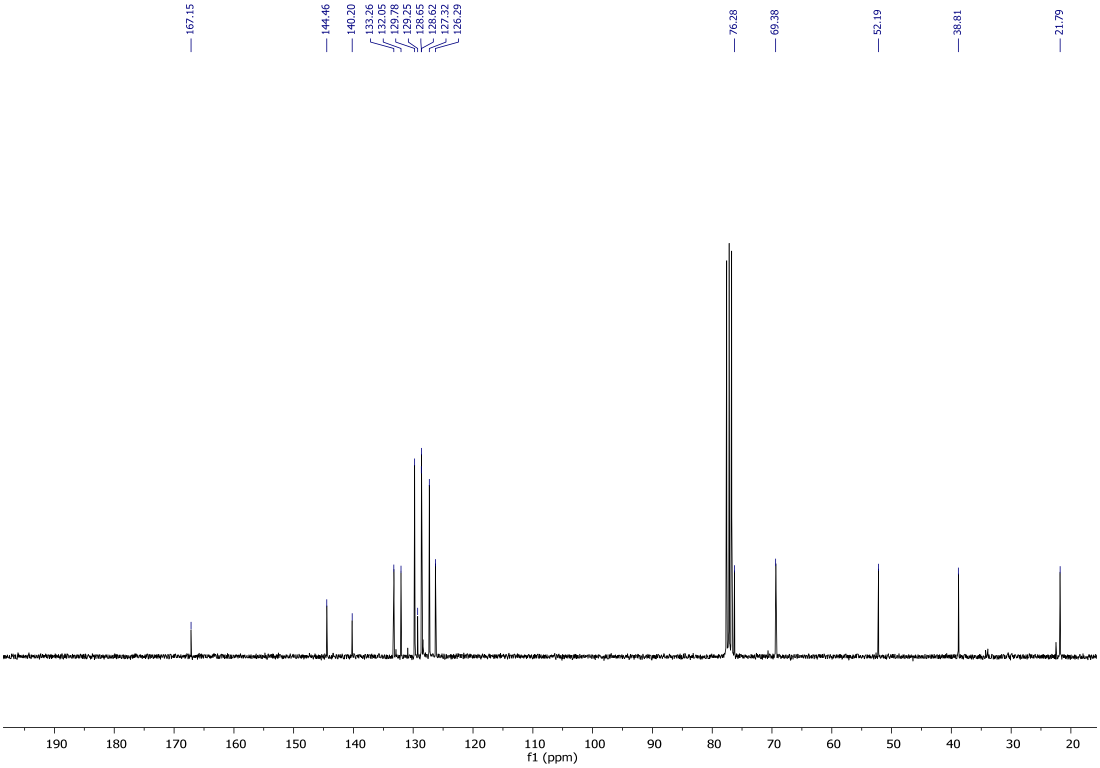

Rac-(E)-Methyl 4-((5-phenylpent-3-en-2-yloxy)methyl)benzoate rac-5gb

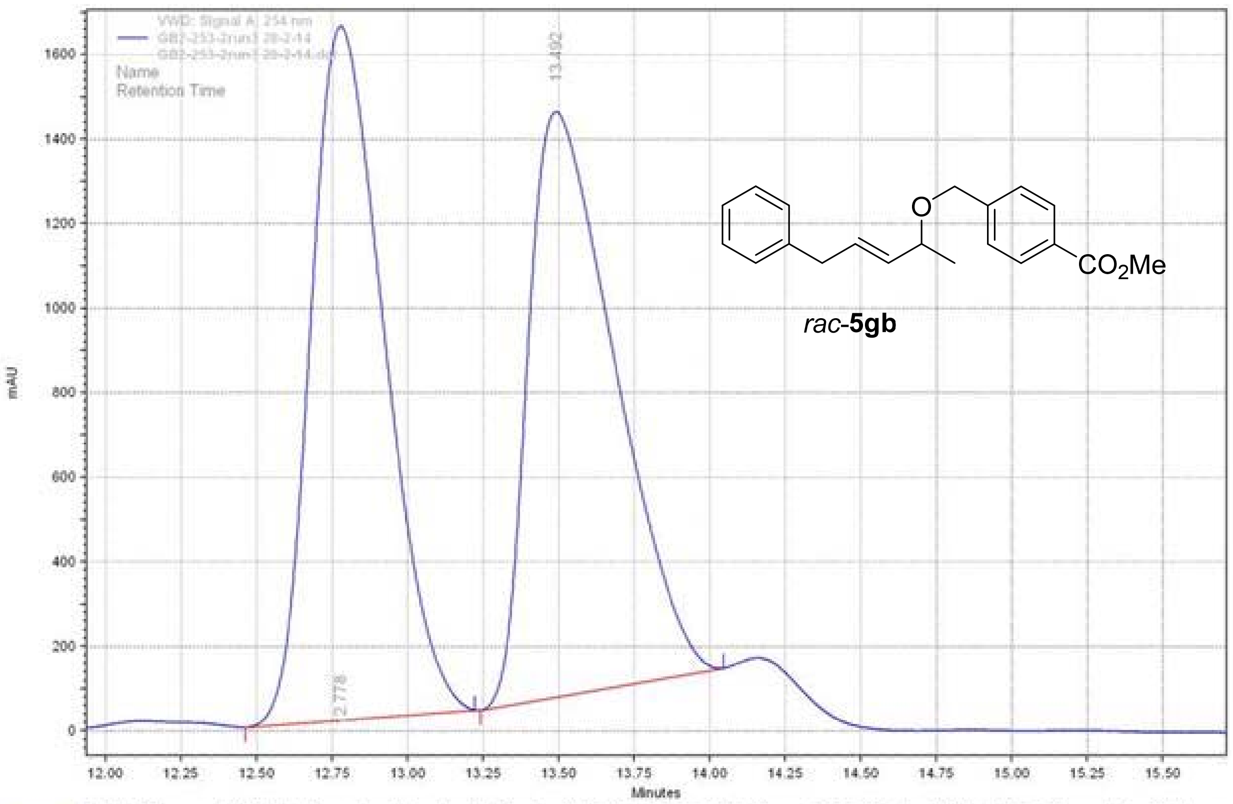

C:\EZChrom Elite\Enterprise\Projects\Default\Data\GB2-253-2run3 28-2-14.dat, VWD: Signal A, 254 nm

VWD: Signal A, 254 nm Results

Retention Time	Area	Area %	Height	Height %
12.778	466568498	50.19	27540326	54.25
13.492	463077932	49.81	23226977	45.75

(*S*, *E*)-Methyl 4-((5-phenylpent-3-en-2-yloxy)methyl)benzoate (*S*)-5gb

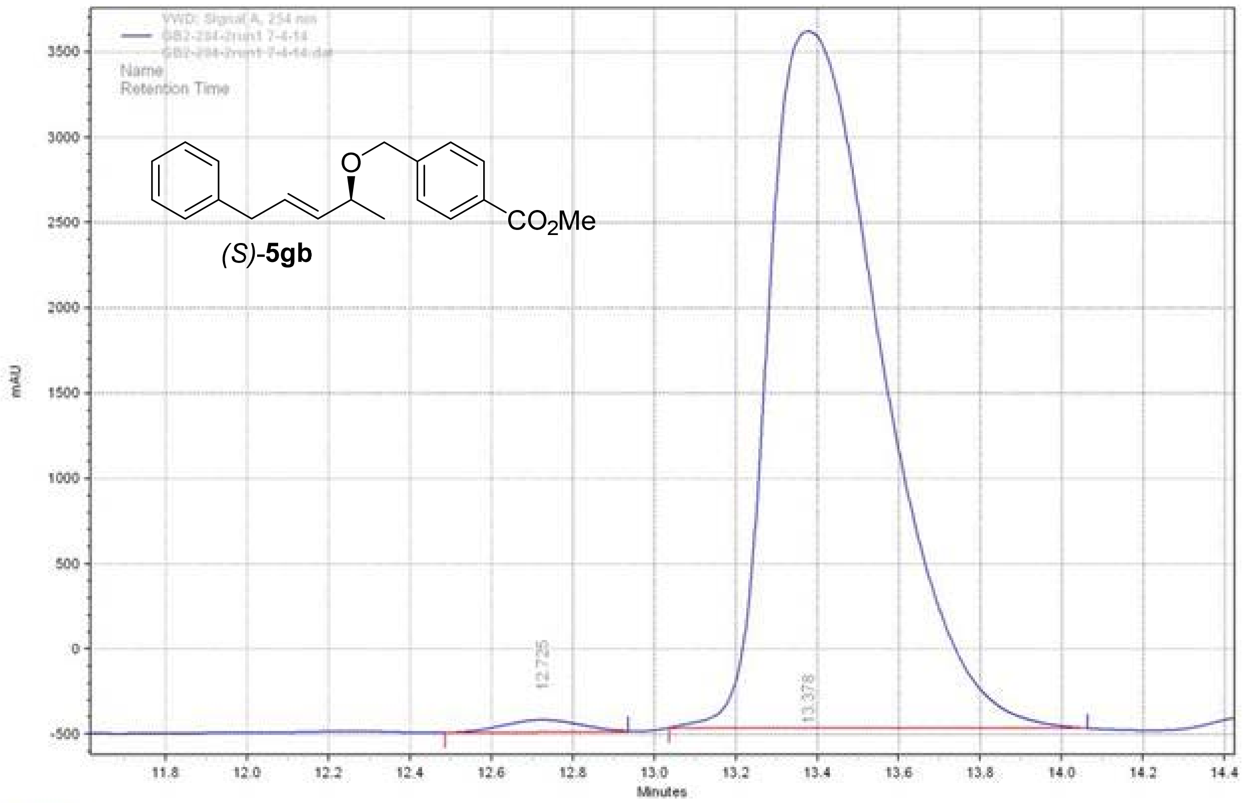

C:\EZChrom Elite\Enterprise\Projects\Default\Data\GB2-284-2run1 7-4-14.dat, VWD: Signal A, 254 nm

VWD: Signal A, 254 nm Results

Retention Time	Area	Area %	Height	Height %
12.725	15207131	1.14	1174186	1.68
13.378	1324447029	98.86	68521490	98.32

Methyl (E)-4-(((4-phenylbut-3-en-2-yl)oxy)methyl)benzoate 5hb

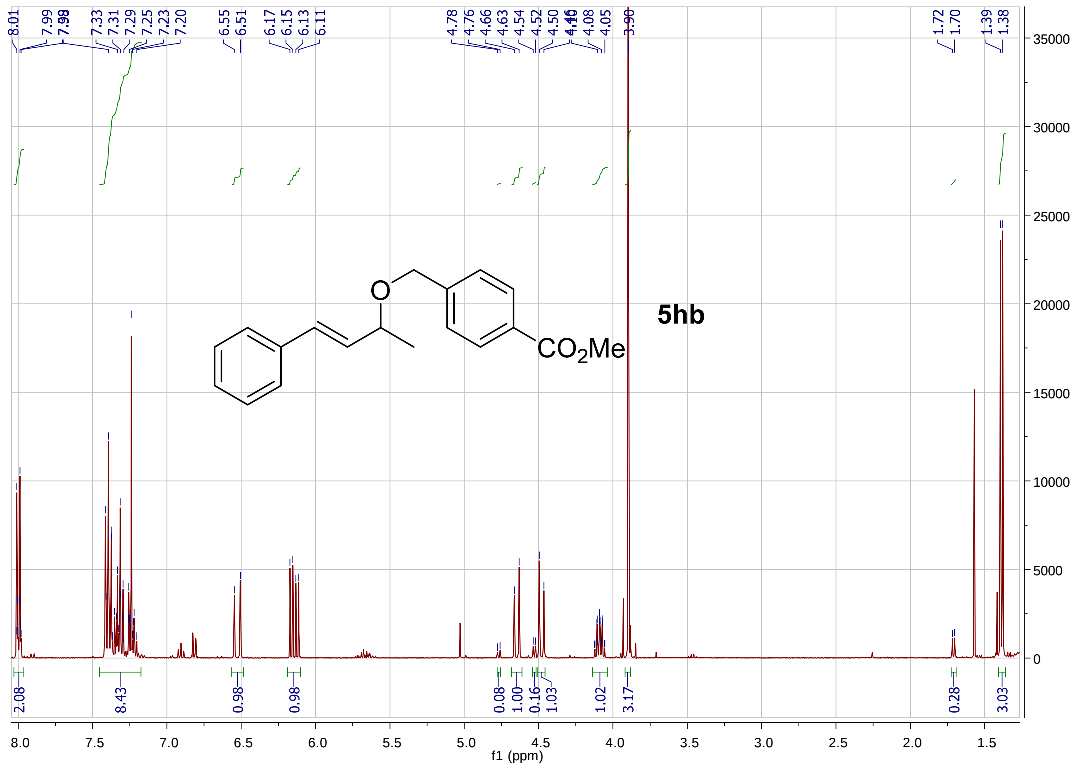

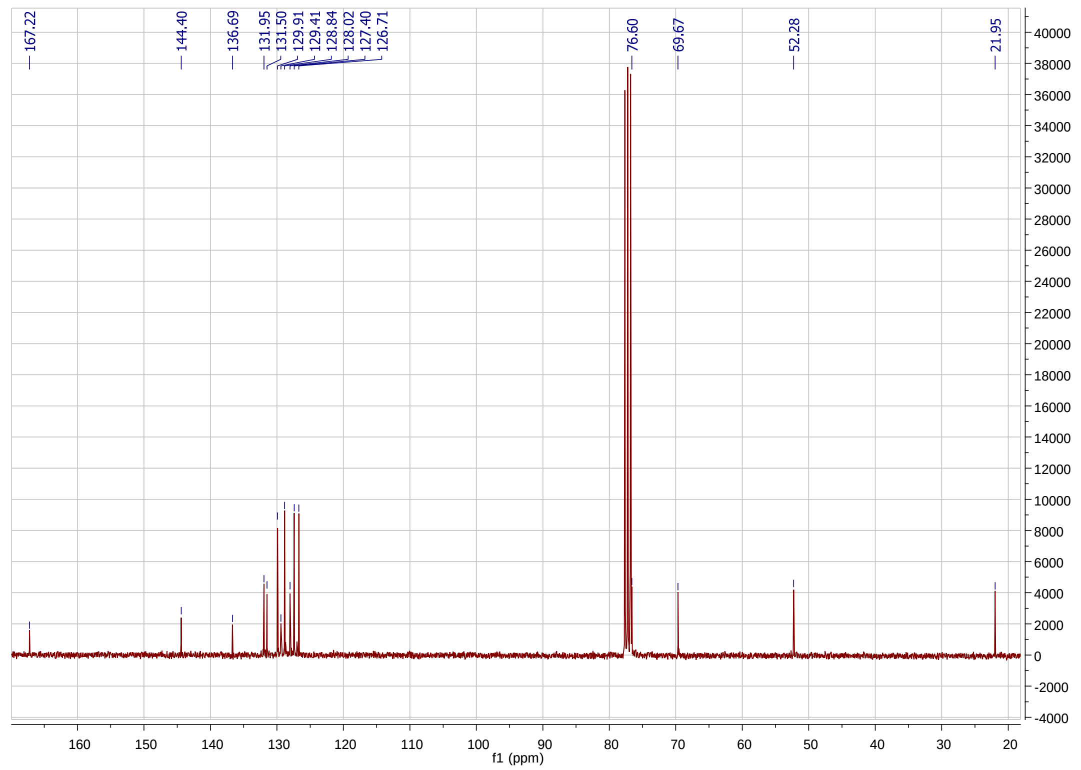

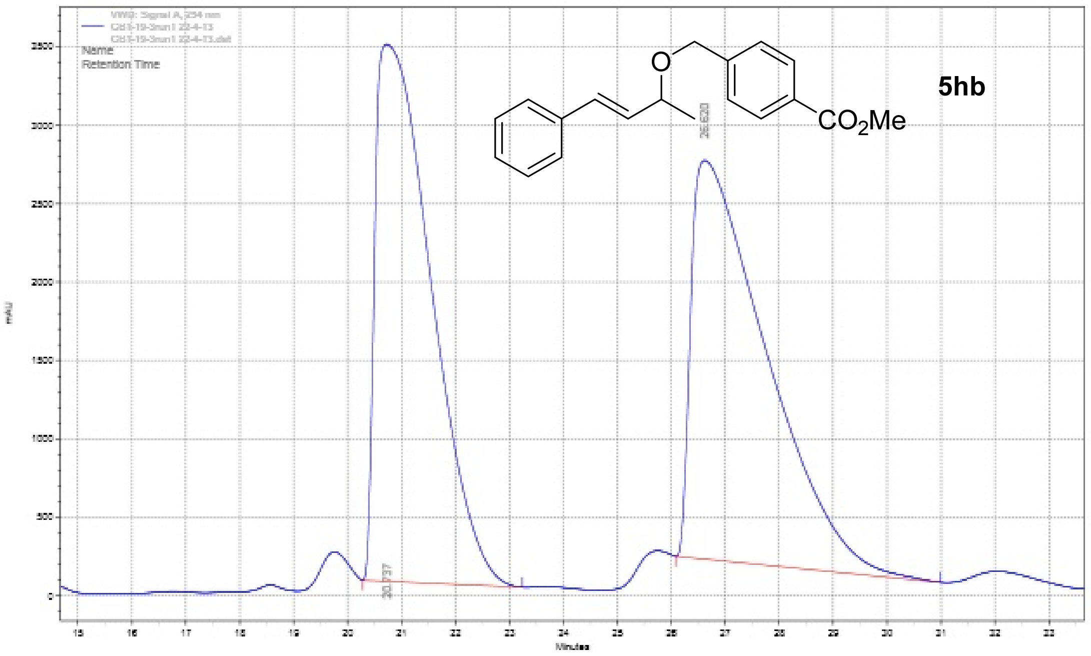

C:\EZChrom Elite\Enterprise\Projects\Default\Data\GB1-19-3run1 22-4-13.dat, VWD: Signal A, 254 nm

VWD: Signal A, 254 nm Results

Retention Time	Area	Area %	Height	Height %
20.737	4143865156	49.95	57455762	57.40
26.620	4151892496	50.05	42645454	42.60

(*S*, *E*)-Methyl 4-((1-(benzyloxy)pent-3-en-2-yloxy)methyl)benzoate (*S*)-5jb

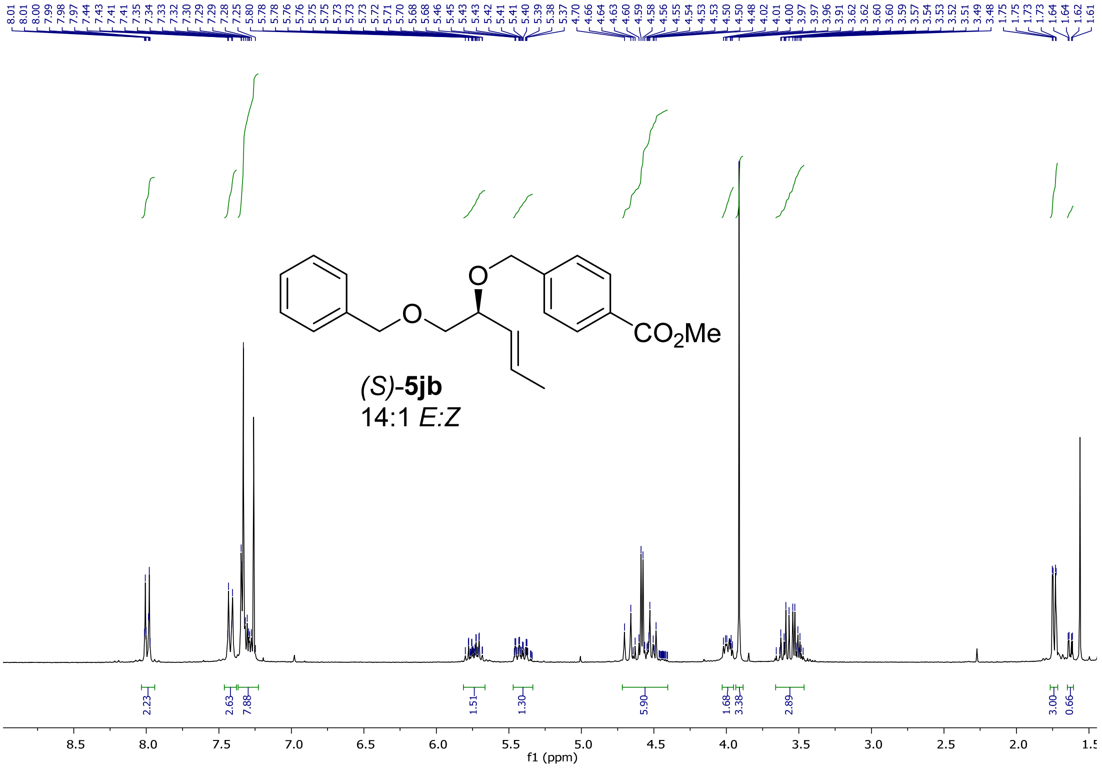

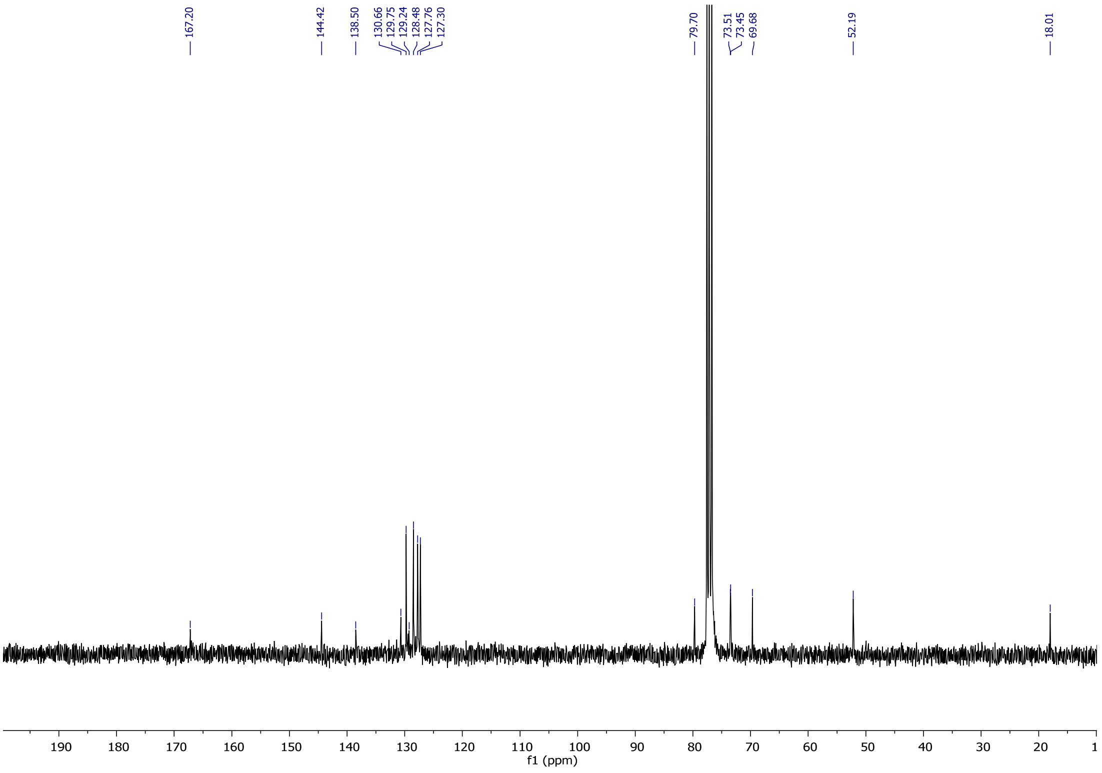

***Rac*-(*E*)-Methyl 4-((1-(benzyloxy)pent-3-en-2-yloxy)methyl)benzoate *rac*-5jb**

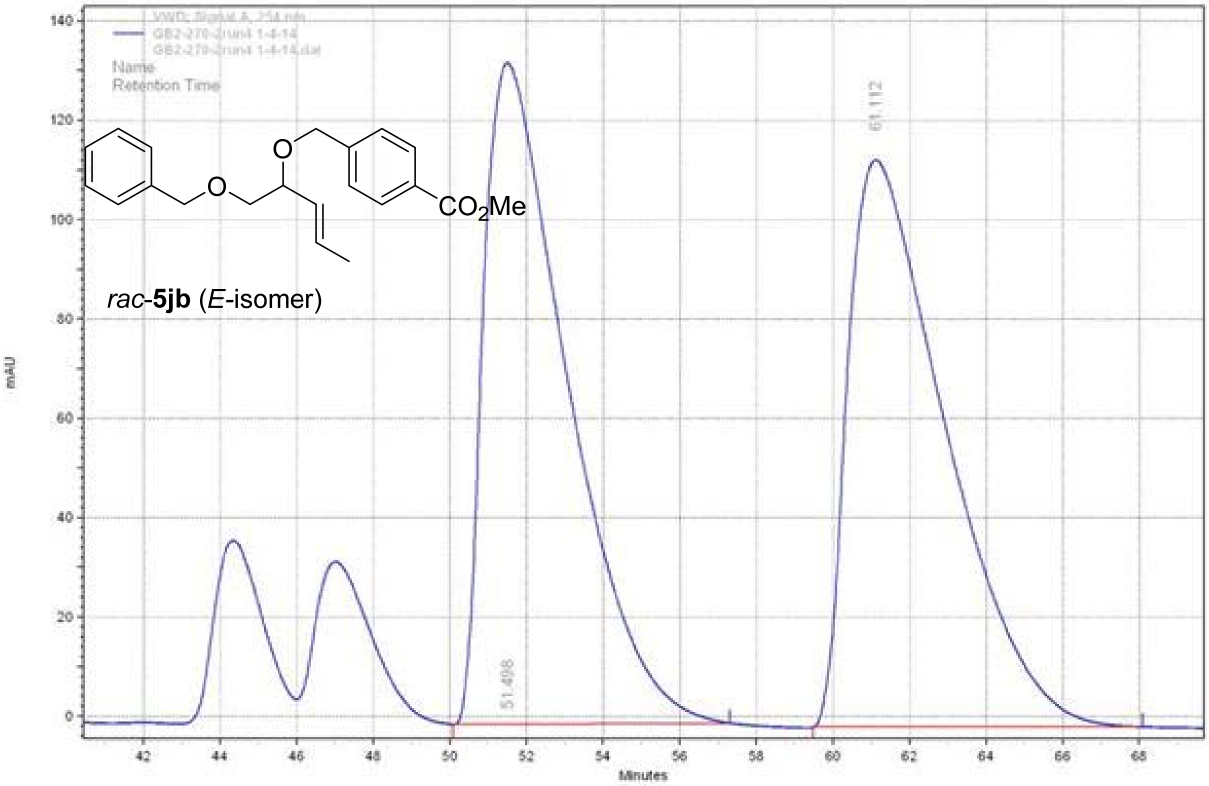

C:\EZChrom Elite\Enterprise\Projects\Default\Data\GB2-270-2run4 1-4-14.dat, VWD: Signal A, 254 nm

VWD: Signal A, 254 nm Results

Retention Time	Area	Area %	Height	Height %
51.498	339946003	50.04	2234107	53.84
61.112	339404254	49.96	1915548	46.16

Rac-(_Z_)-Methyl 4-((1-(benzyloxy)pent-3-en-2-yloxy)methyl)benzoate _rac_-5jb

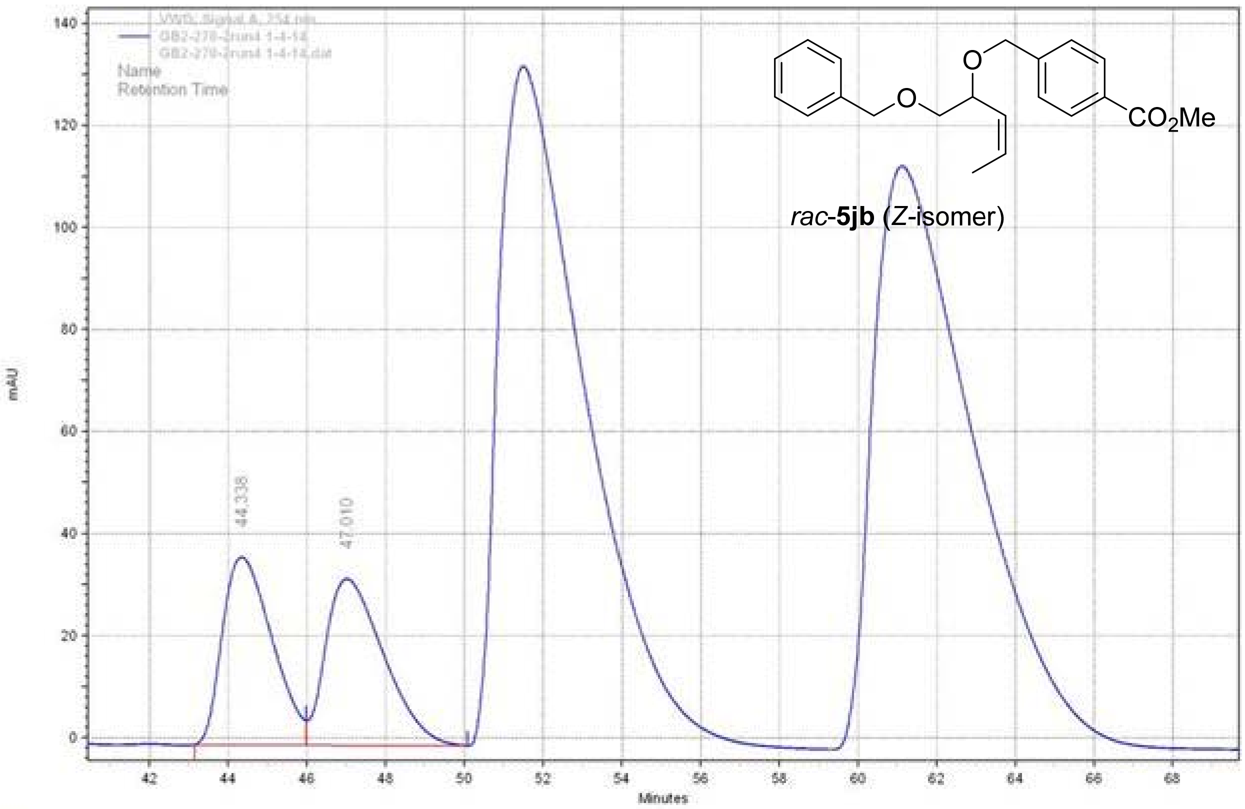

C:\EZChrom Elite\Enterprise\Projects\Default\Data\GB2-270-2run4 1-4-14.dat, VWD: Signal A, 254 nm

VWD: Signal A, 254 nm Results

Retention Time	Area	Area %	Height	Height %
44.338	54066576	49.06	616769	52.99
47.010	56130221	50.94	547258	47.01

(*S*, *E*)-Methyl 4-((1-(benzyloxy)pent-3-en-2-yloxy)methyl)benzoate (*S*)-5jb

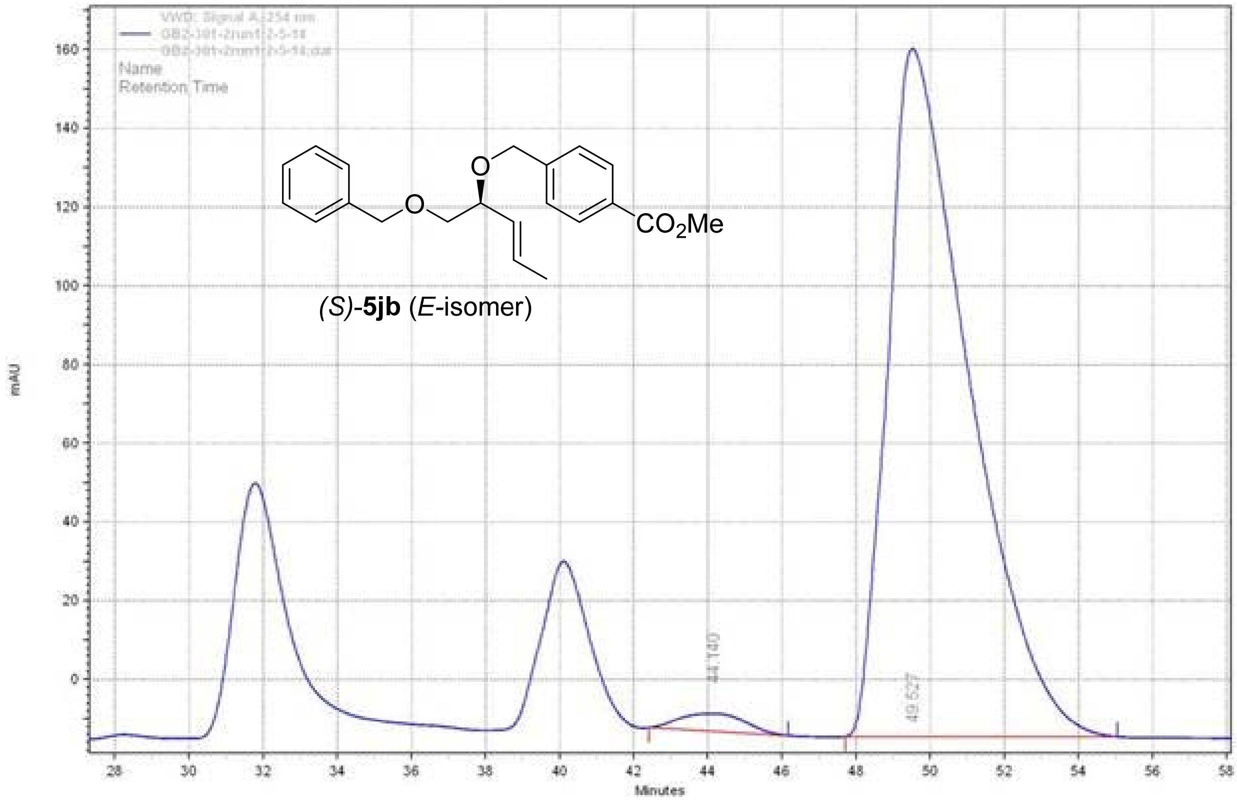

C:\EZChrom Elite\Enterprise\Projects\Default\Data\GB2-301-2run1 2-5-14.dat, VWD: Signal A, 254 nm

VWD: Signal A,
254 nm Results

Retention Time	Area	Area %	Height	Height %
44.140	9379266	2.06	76559	2.54
49.527	446999124	97.94	2931798	97.46

(*S*, *Z*)-Methyl 4-((1-(benzyloxy)pent-3-en-2-yloxy)methyl)benzoate (*S*)-5jb

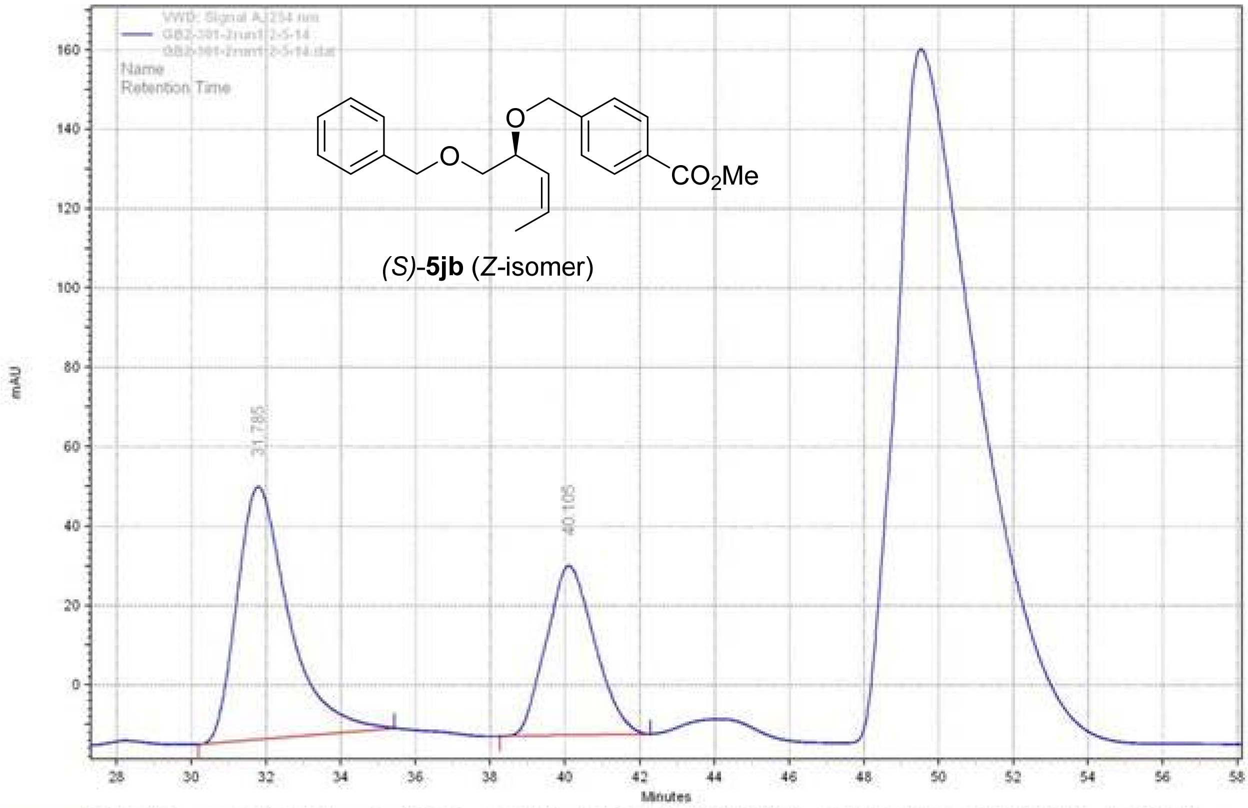

C:\EZChrom Elite\Enterprise\Projects\Default\Data\GB2-301-2run1 2-5-14.dat, VWD: Signal A, 254 nm

VWD: Signal A, 254 nm Results

Retention Time	Area	Area %	Height	Height %
31.785	102973112	61.58	1067636	59.83
40.105	64231997	38.42	716857	40.17

(*S*, *E*)-Methyl 4-((1-(benzyloxy)pent-3-en-2-yloxy)methyl)benzoate (*S*)-5jb (Scheme 7, Eq. 2)

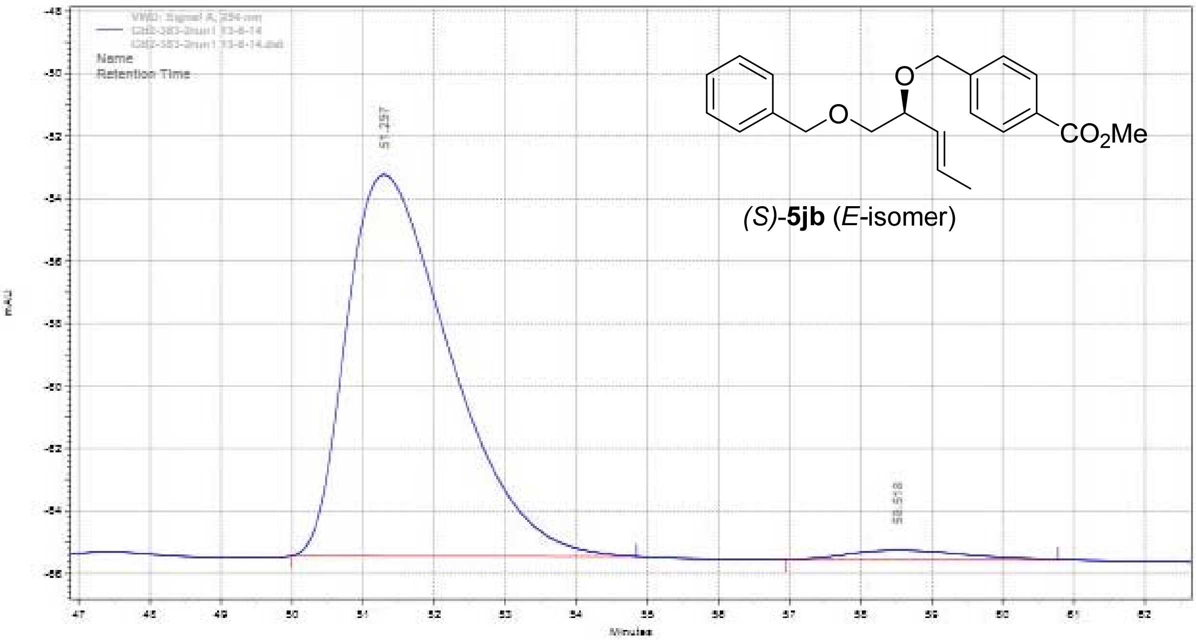

C:\EZChrom Elite\Enterprise\Projects\Default\Data\GB2-383-2run1 13-8-14.dat, VWD: Signal A, 254 nm

VWD: Signal A,
254 nm Results

Retention Time	Area	Area %	Height	Height %
51.297	20794754	97.39	204756	97.61
58.518	556444	2.61	5020	2.39

(*S*, *E*)-Methyl 4-((pent-3-en-2-yloxy)methyl)benzoate (*S*)-5kb

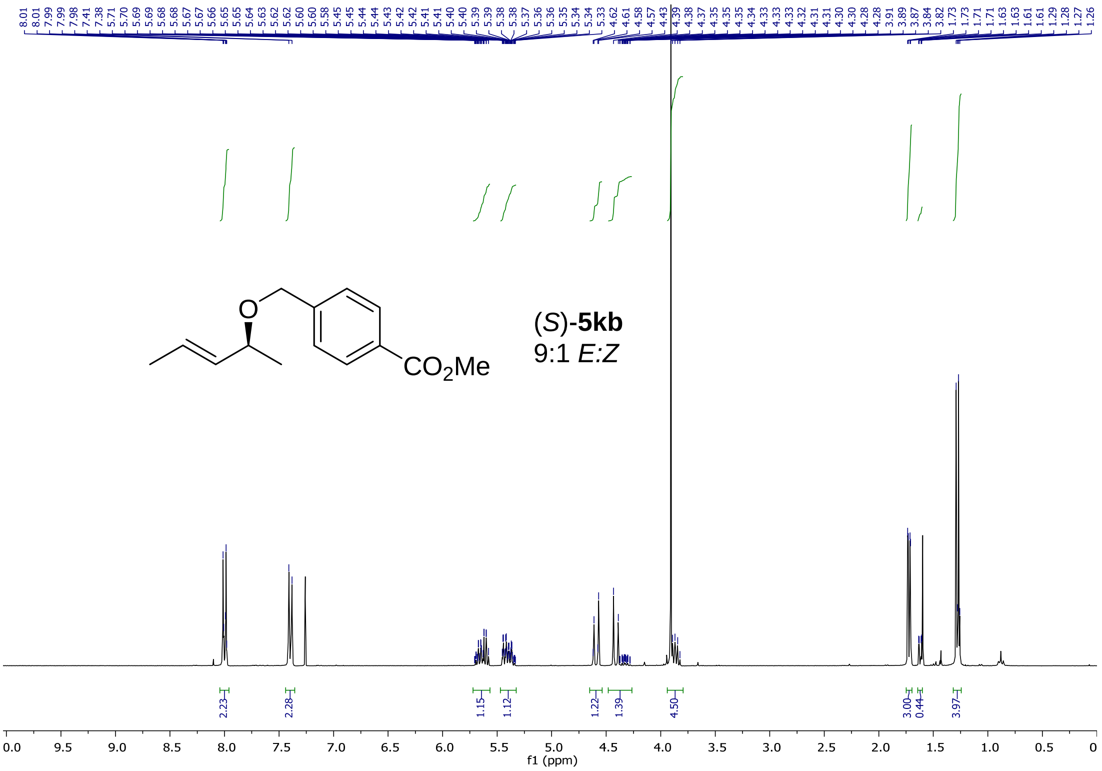

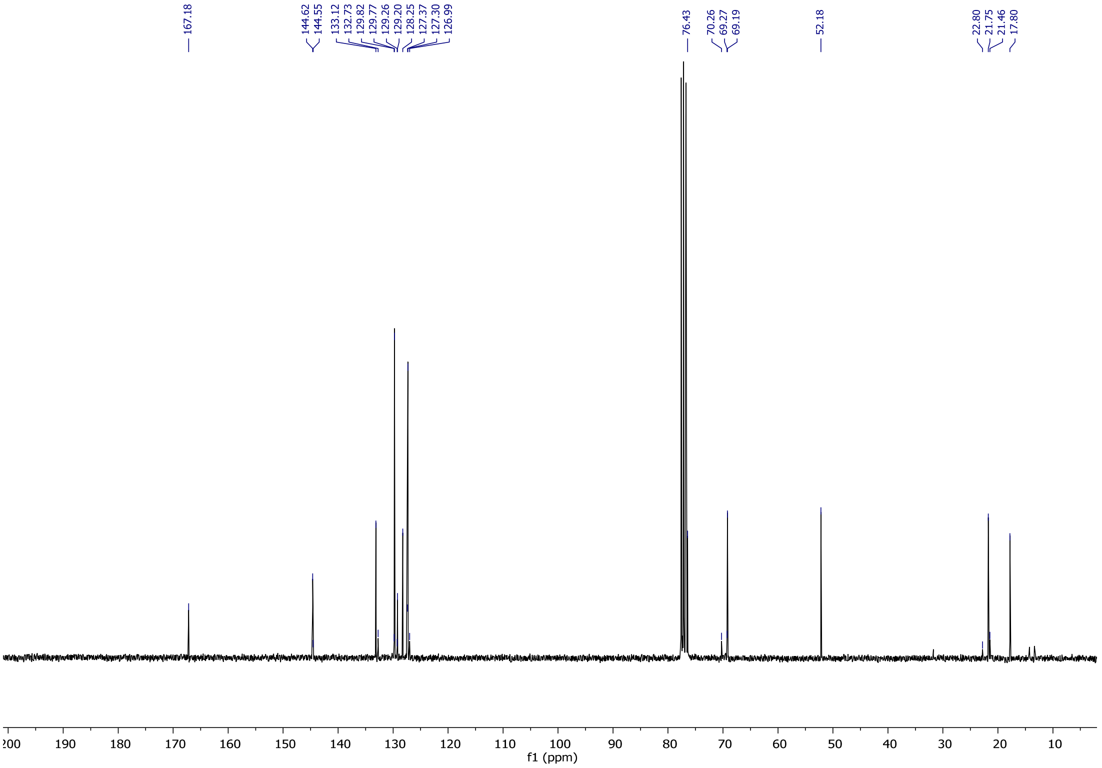

***Rac*-(*E*)-Methyl 4-((pent-3-en-2-yloxy)methyl)benzoate *rac*-5kb**

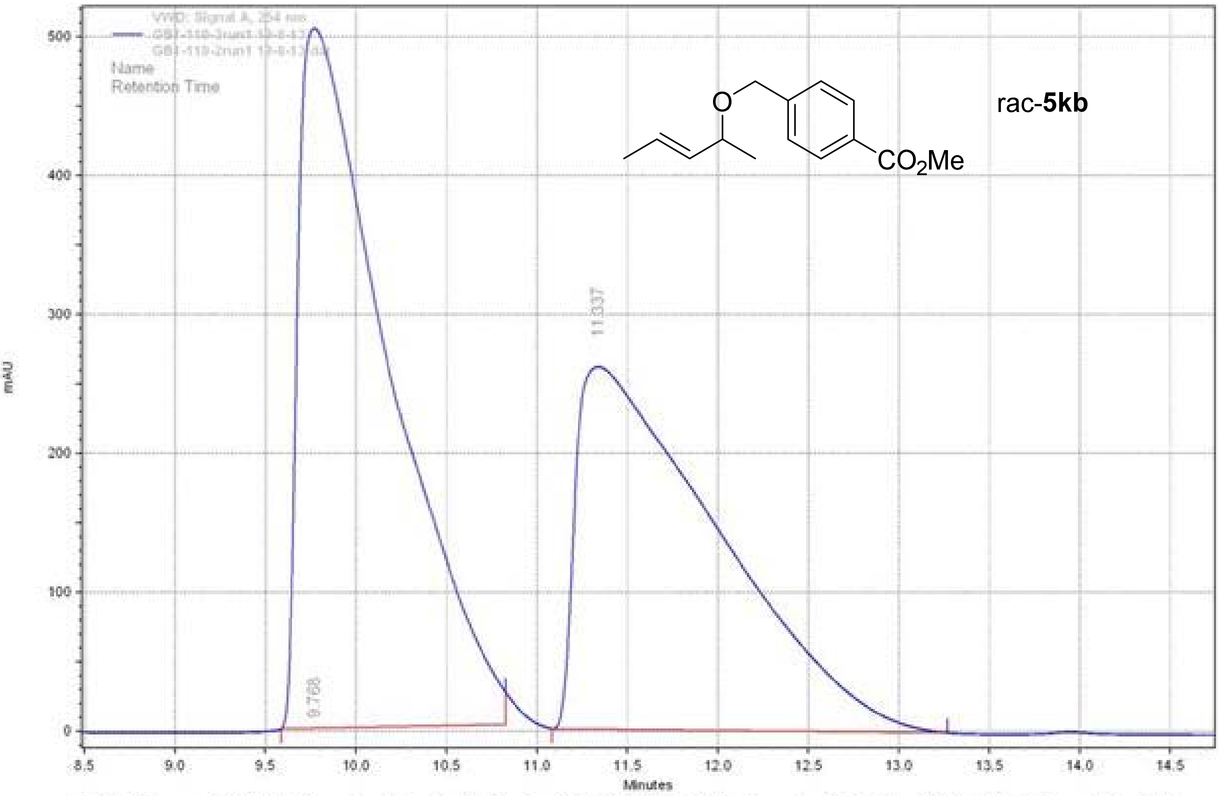

C:\EZChrom Elite\Enterprise\Projects\Default\Data\GB1-110-2run1 19-8-13.dat, VWD: Signal A, 254 nm

VWD: Signal A,
254 nm Results

Retention Time	Area	Area %	Height	Height %
9.768	294575301	55.23	8453524	65.88
11.337	238785388	44.77	4377342	34.12

(S, E)-Methyl 4-((pent-3-en-2-yloxy)methyl)benzoate (*S*)-5kb

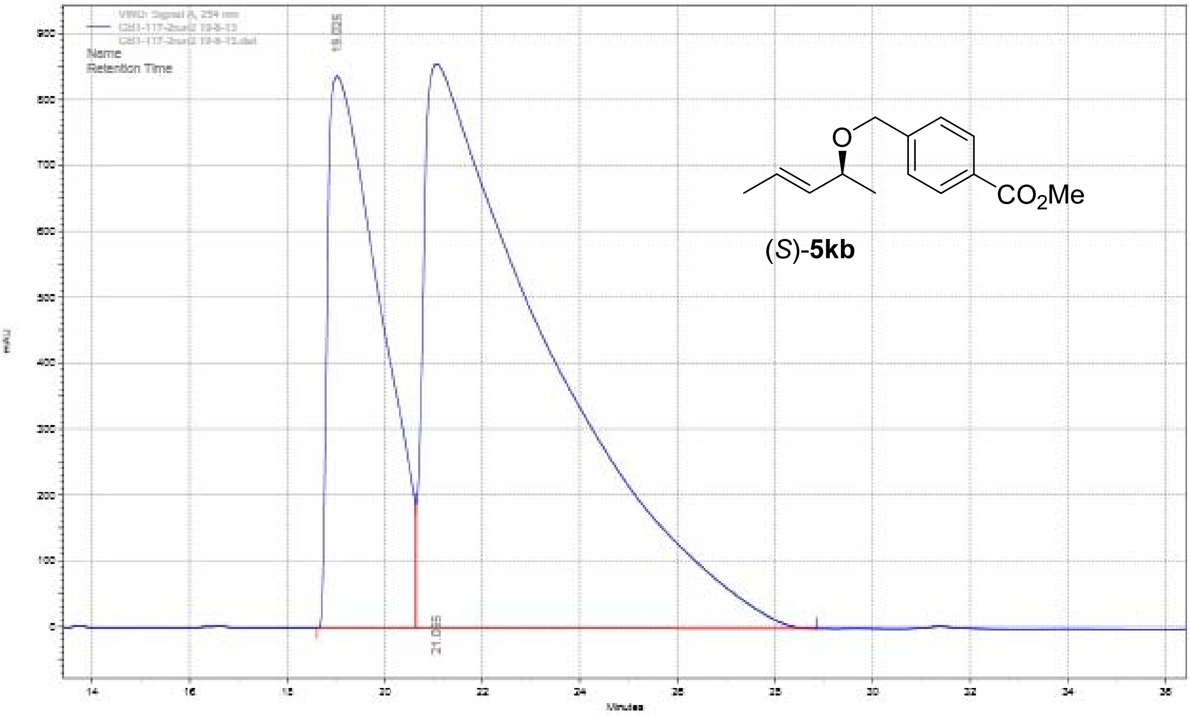

C:\EZChrom Elite\Enterprise\Projects\Default\Data\GB1-117-2run2 19-8-13.dat, VWD: Signal A, 254 nm

VWD: Signal A,
254 nm Results

Retention Time	Area	Area %	Height	Height %
19.025	1044987468	29.07	14037032	49.42
21.065	2550120010	70.93	14366540	50.58

(*R*, *E*)-Methyl 4-((5,5-dimethylhex-3-en-2-yloxy)methyl)benzoate (*R*)-5lb

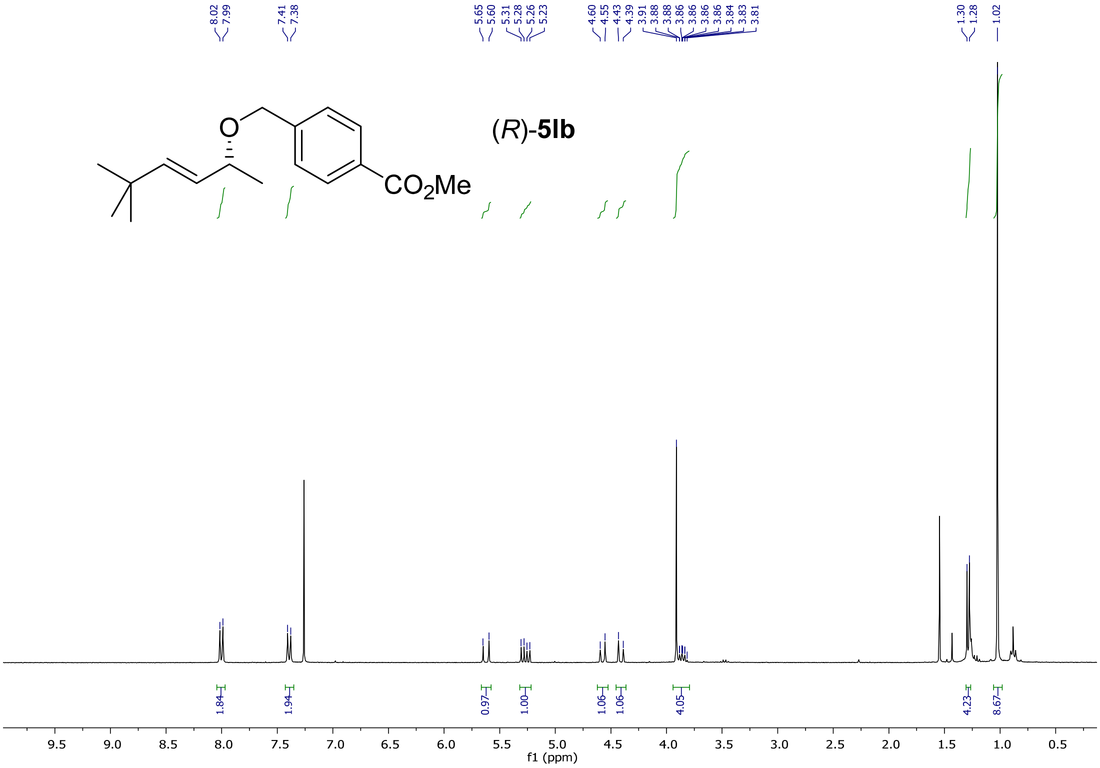

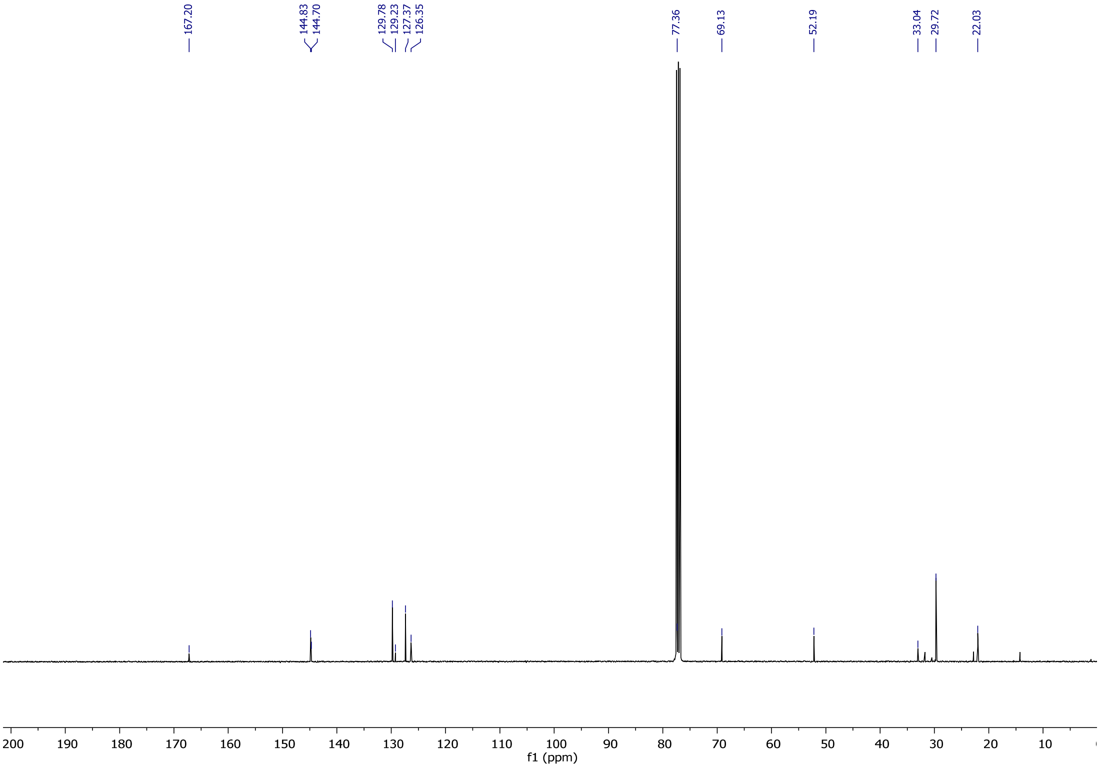

***Rac*-(*E*)-Methyl 4-((5,5-dimethylhex-3-en-2-yloxy)methyl)benzoate *rac*-5lb**

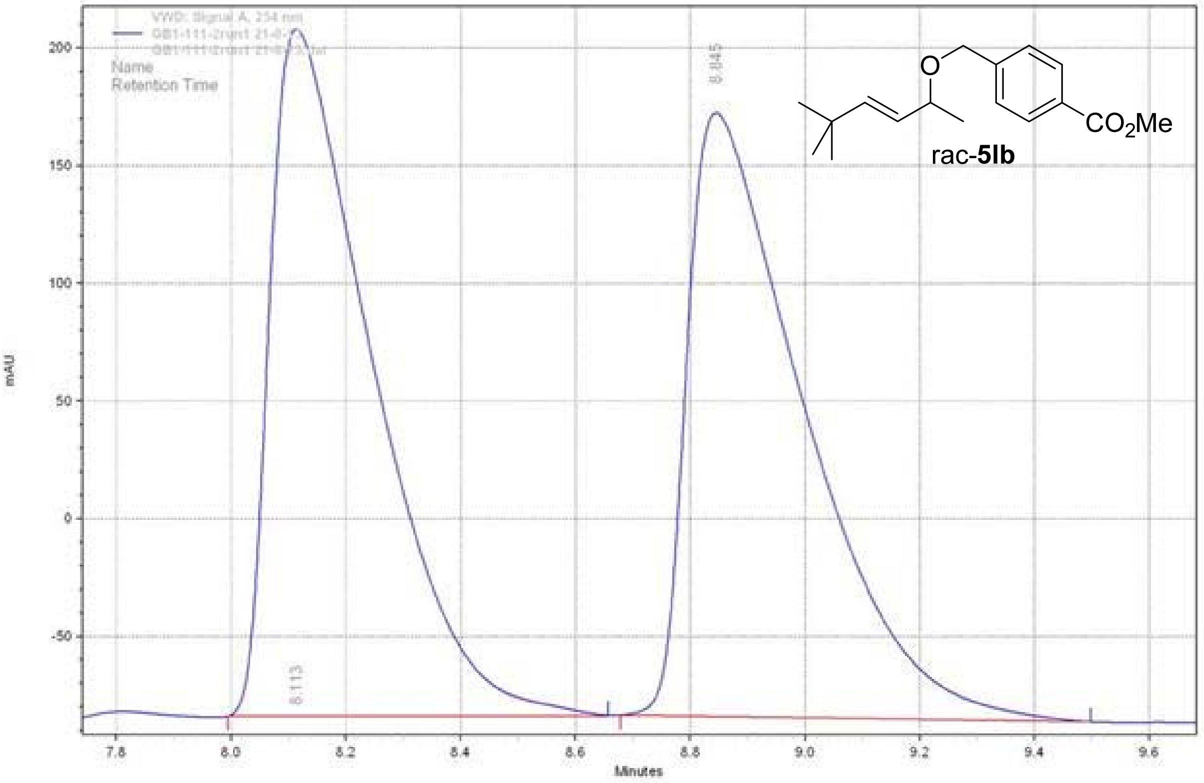

C:\EZChrom Elite\Enterprise\Projects\Default\Data\GB1-111-2run1 21-8-13.dat, VWD: Signal A, 254 nm

VWD: Signal A, 254 nm Results

Retention Time	Area	Area %	Height	Height %
8.113	60638007	49.76	4895883	53.23
8.845	61234540	50.24	4302111	46.77

(*R*, *E*)-Methyl 4-((5,5-dimethylhex-3-en-2-yloxy)methyl)benzoate (*R*)-5lb

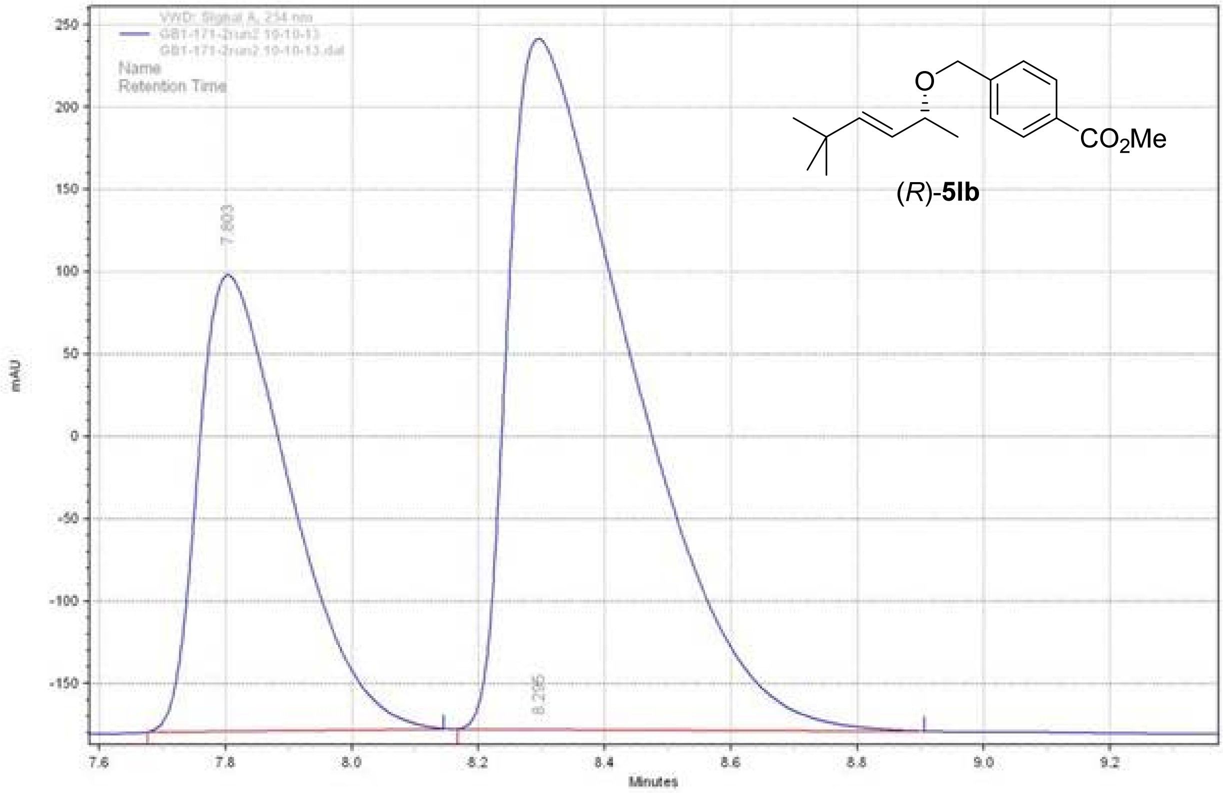

C:\EZChrom Elite\Enterprise\Projects\Default\Data\GB1-171-2run2 10-10-13.dat, VWD: Signal A, 254 nm

VWD: Signal A,
254 nm Results

Retention Time	Area	Area %	Height	Height %
7.803	46833071	32.76	4651426	39.79
8.295	96146714	67.24	7038848	60.21

(*E*)-Methyl 4-((3-methylpent-3-en-2-yloxy)methyl)benzoate 5mb

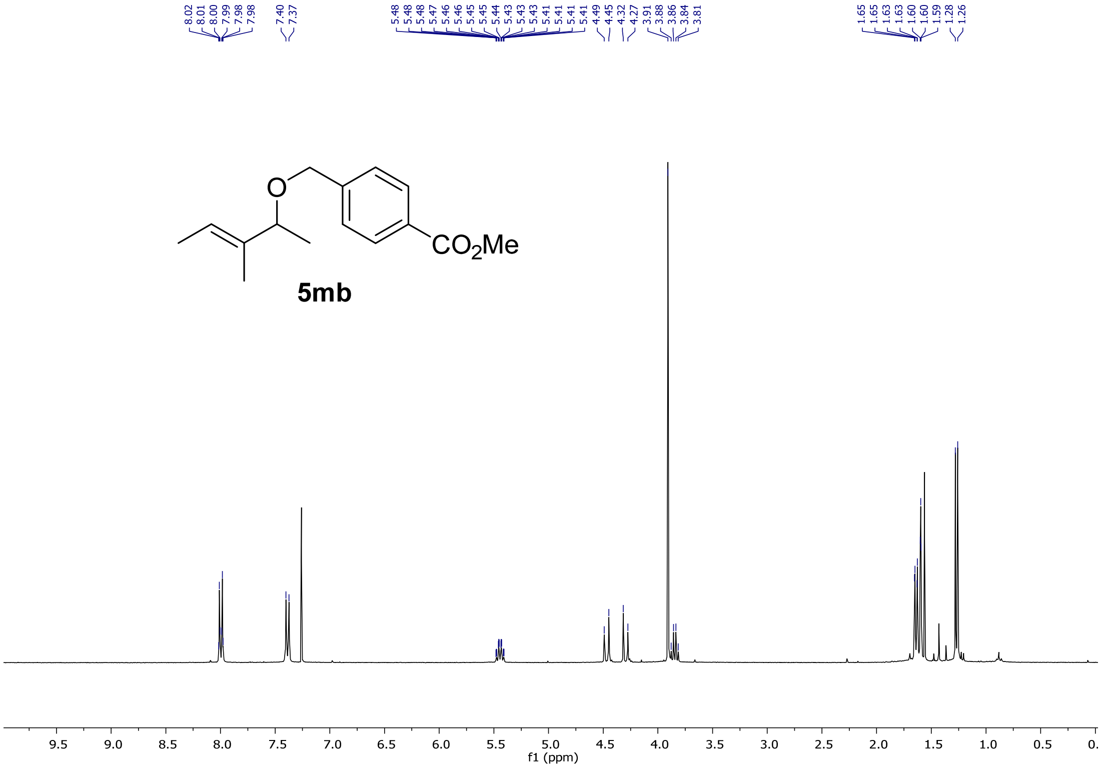

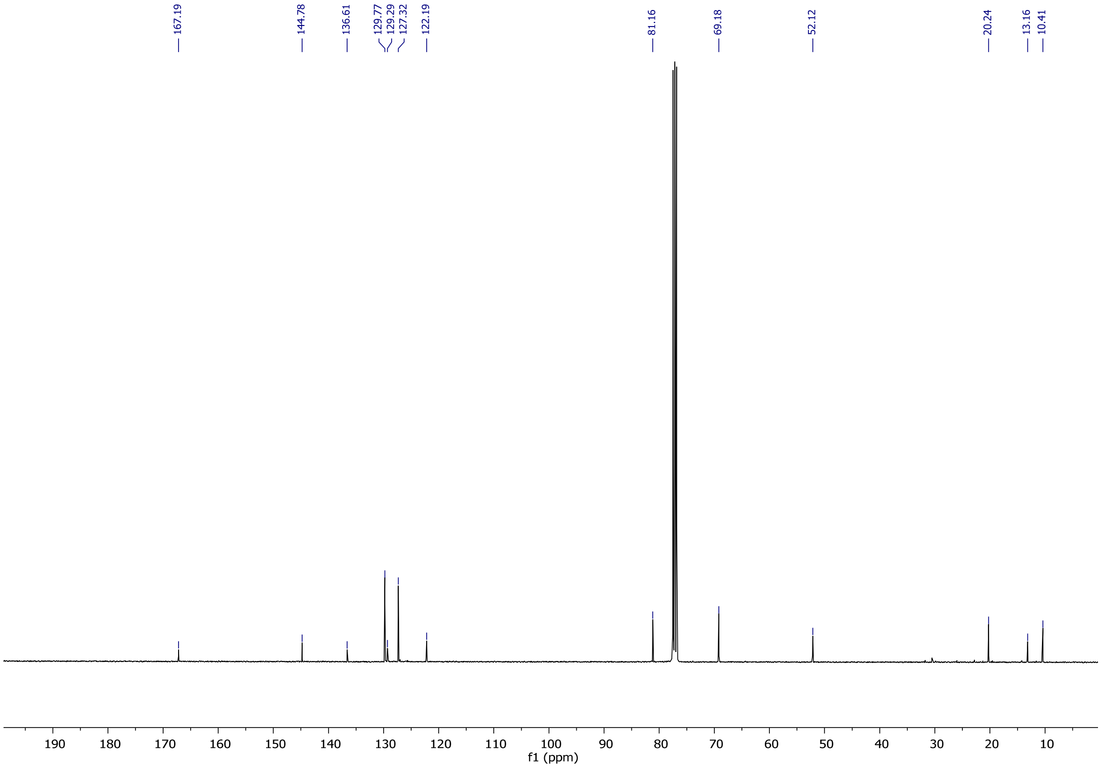

(*E*)-Methyl 4-((3-methylpent-3-en-2-yloxy)methyl)benzoate *rac*-5mb

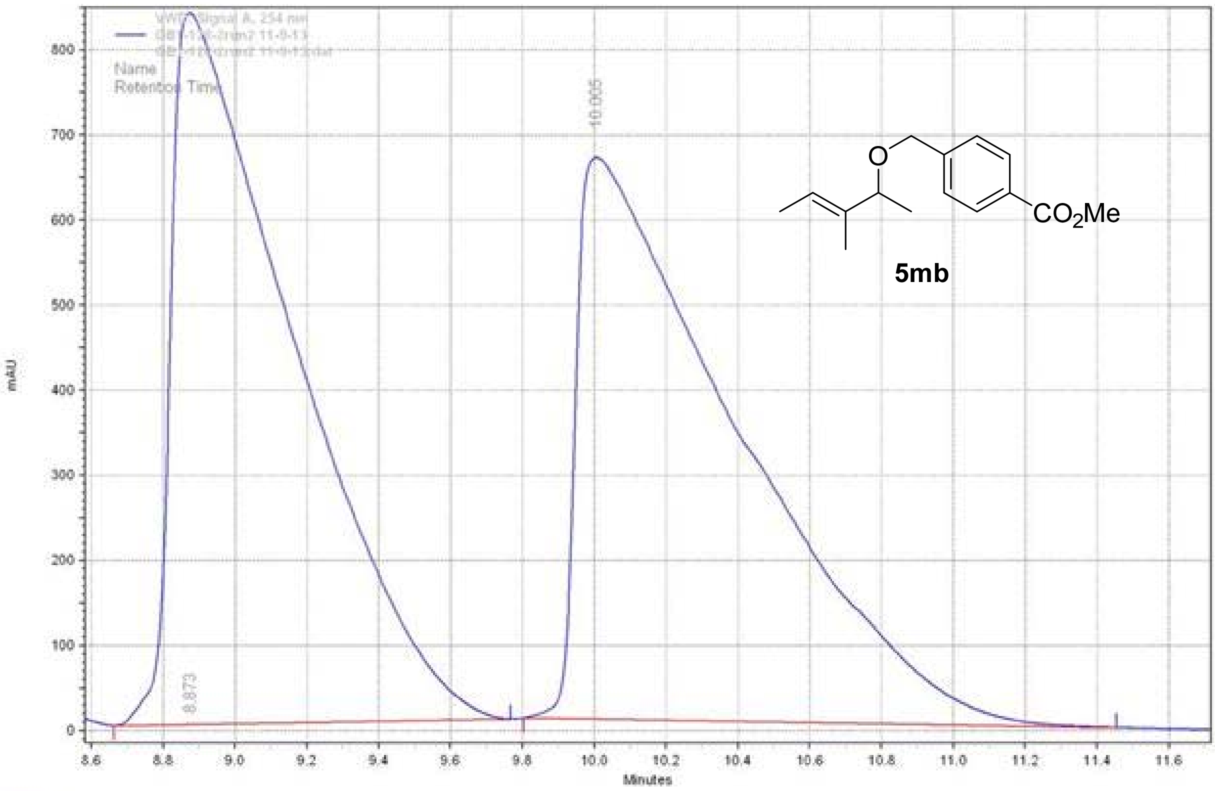

C:\EZChrom Elite\Enterprise\Projects\Default\Data\GB1-128-2run2 11-9-13.dat, VWD: Signal A, 254 nm

VWD: Signal A,
254 nm Results

Retention Time	Area	Area %	Height	Height %
8.873	335979748	49.32	14025244	55.87
10.005	345272680	50.68	11076633	44.13

(*R*, *E*)-Methyl 4-((4-cyclohexyl-3-methylbut-3-en-2-yloxy)methyl)benzoate (*R*)-5nb

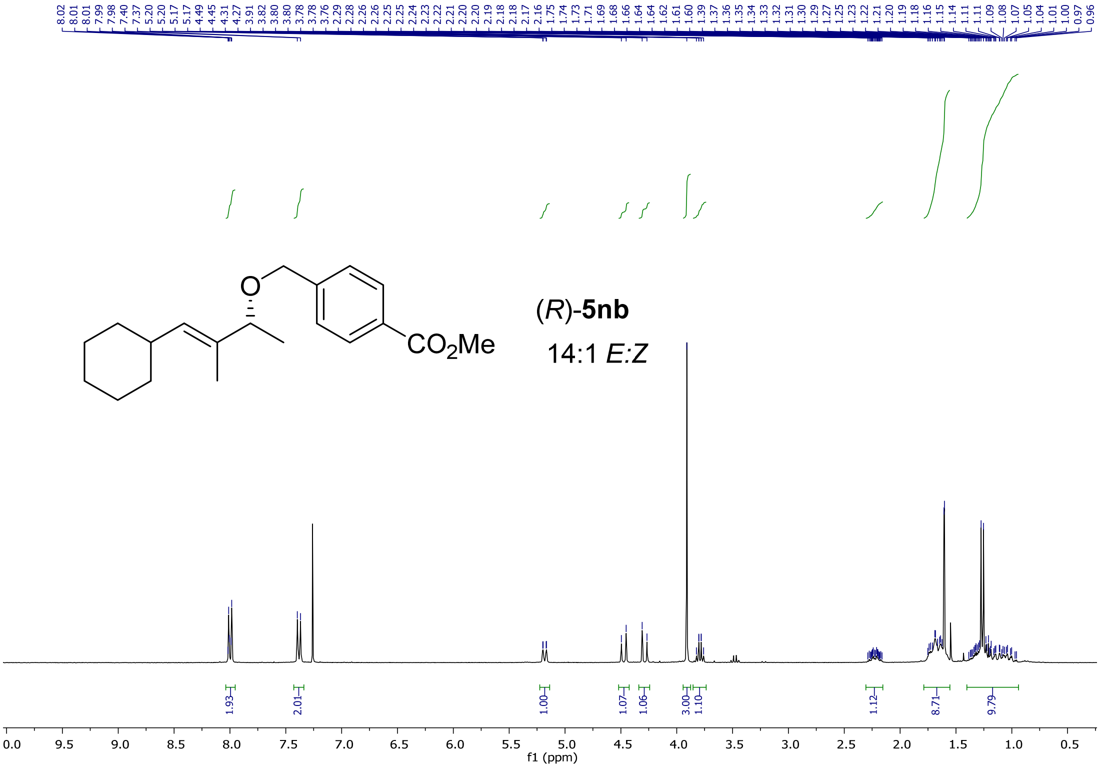

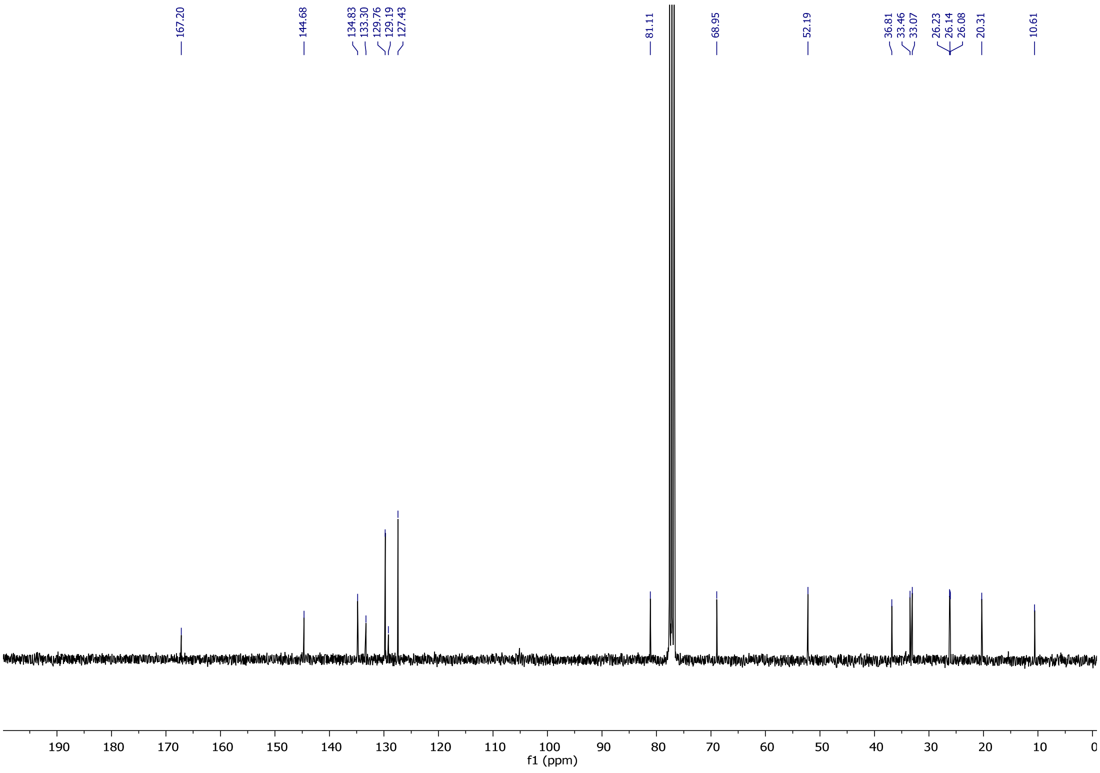

***Rac*-(*E*)-Methyl 4-((4-cyclohexyl-3-methylbut-3-en-2-yloxy)methyl)benzoate *rac*-5nb**

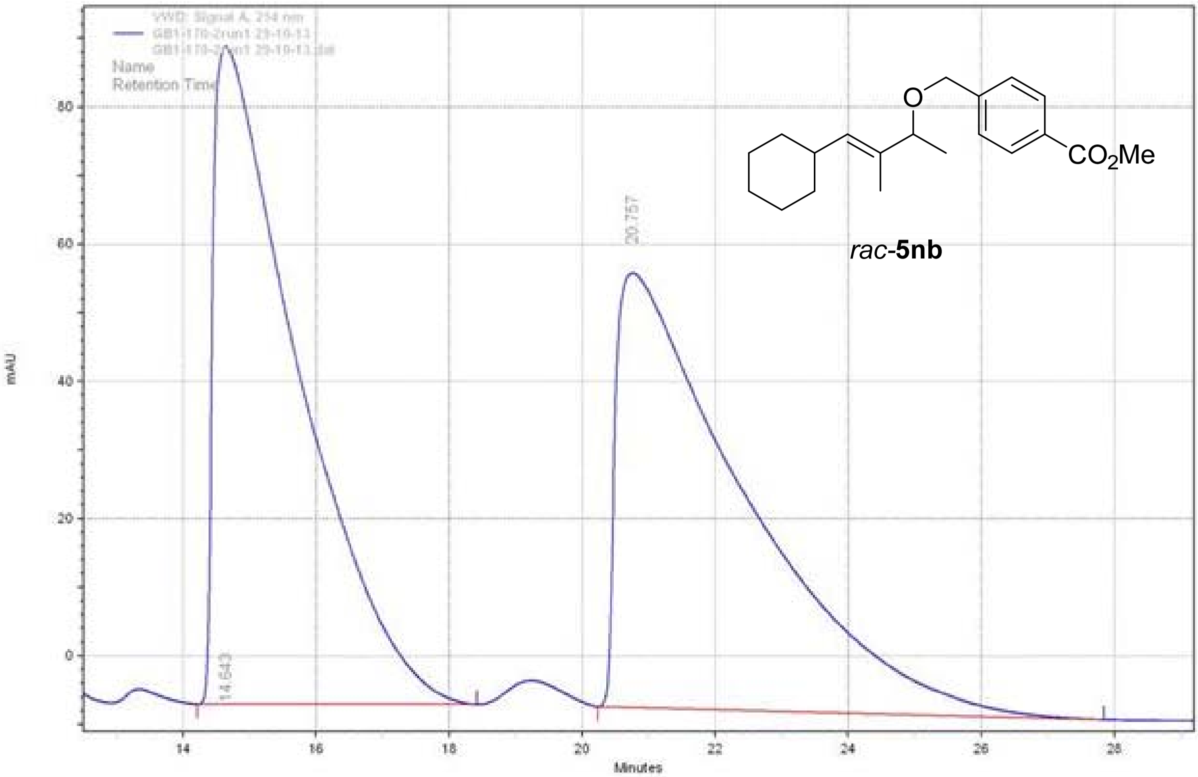

rac-**5nb**

C:\EZChrom Elite\Enterprise\Projects\Default\Data\GB1-170-2run1 29-10-13.dat, VWD: Signal A, 254 nm

VWD: Signal A,
254 nm Results

Retention Time	Area	Area %	Height	Height %
14.643	142535094	49.88	1607630	60.21
20.757	143224938	50.12	1062588	39.79

(*R*, *E*)-Methyl 4-((4-cyclohexyl-3-methylbut-3-en-2-yloxy)methyl)benzoate "*(R)*-5nb"

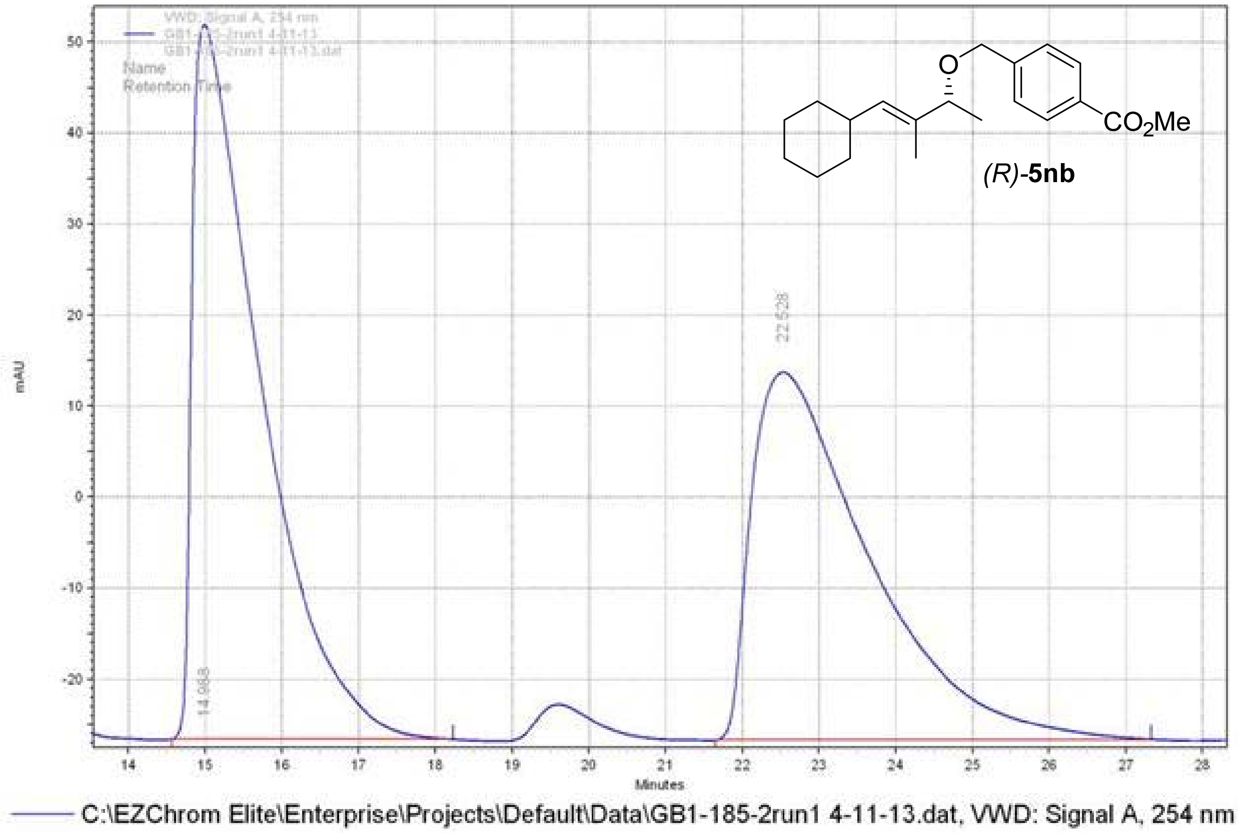

VWD: Signal A, 254 nm Results

Retention Time	Area	Area %	Height	Height %
14.988	81383927	52.99	1316308	66.01
22.528	72211812	47.01	677884	33.99

Nucleophile Alcohol Scope Spectra

(*S*, *E*)-((Oct-3-en-2-yloxy)methyl)benzene (*S*)-5ac

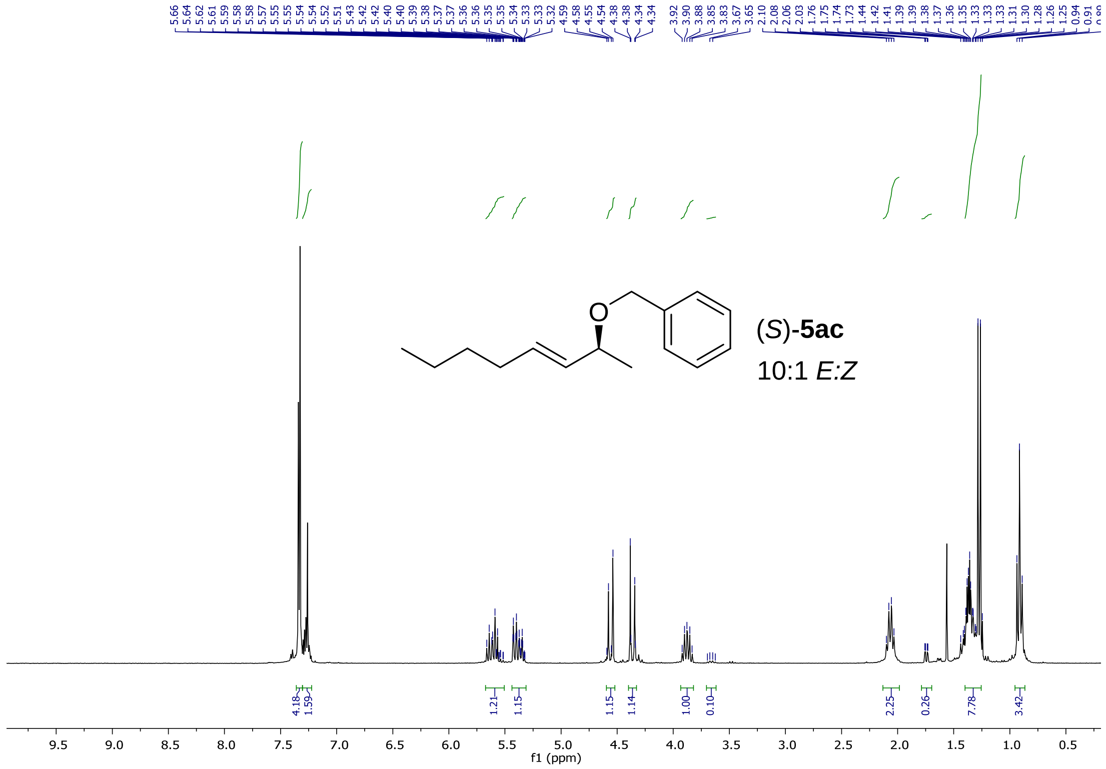

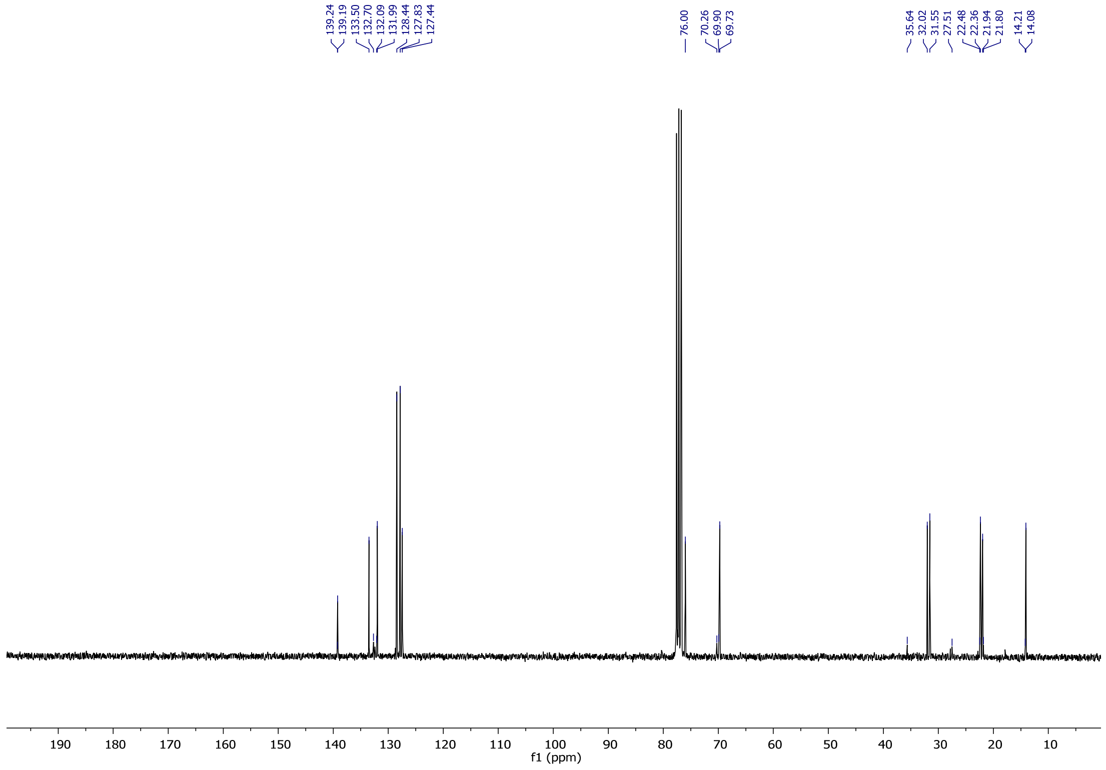

***Rac*-(*E*)-((Oct-3-en-2-yloxy)methyl)benzene *rac*-5ac**

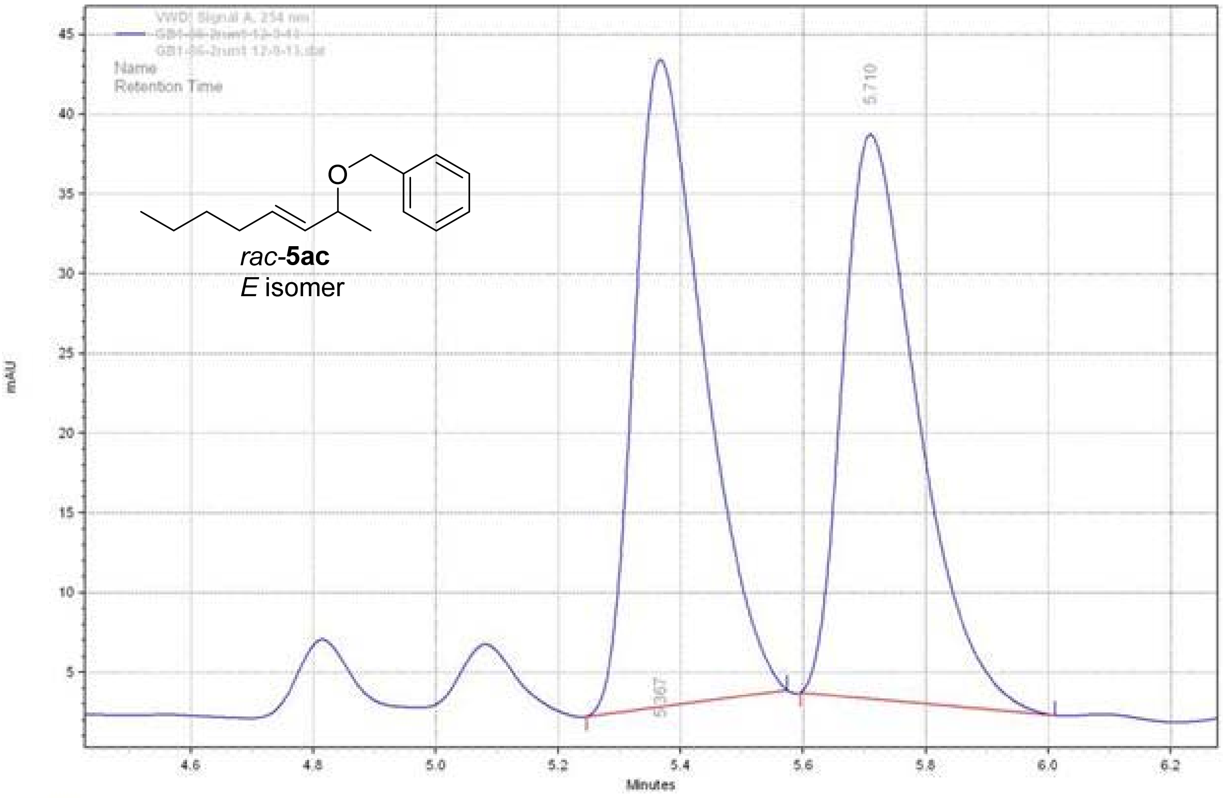

C:\EZChrom Elite\Enterprise\Projects\Default\Data\GB1-86-2run1 12-9-13.dat, VWD: Signal A, 254 nm

VWD: Signal A,
254 nm Results

Retention Time	Area	Area %	Height	Height %
5.367	5390030	51.54	680549	53.40
5.710	5068020	48.46	593780	46.60

***Rac*-(*Z*)-((Oct-3-en-2-yloxy)methyl)benzene *rac*-5ac**

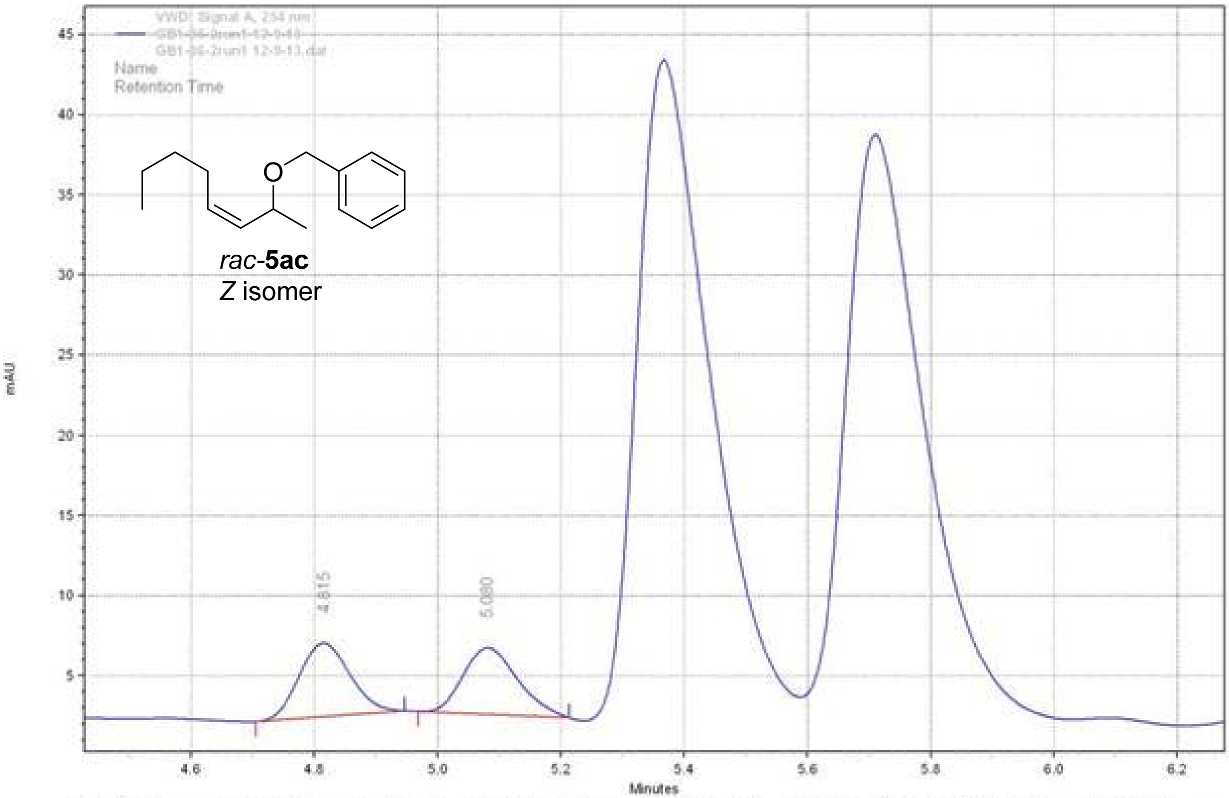

C:\EZChrom Elite\Enterprise\Projects\Default\Data\GB1-86-2run1 12-9-13.dat, VWD: Signal A, 254 nm

VWD: Signal A,
254 nm Results

Retention Time	Area	Area %	Height	Height %
4.815	441519	50.68	77403	52.58
5.080	429707	49.32	69817	47.42

(*S*, *E*)-((Oct-3-en-2-yloxy)methyl)benzene (*S*)-5ac

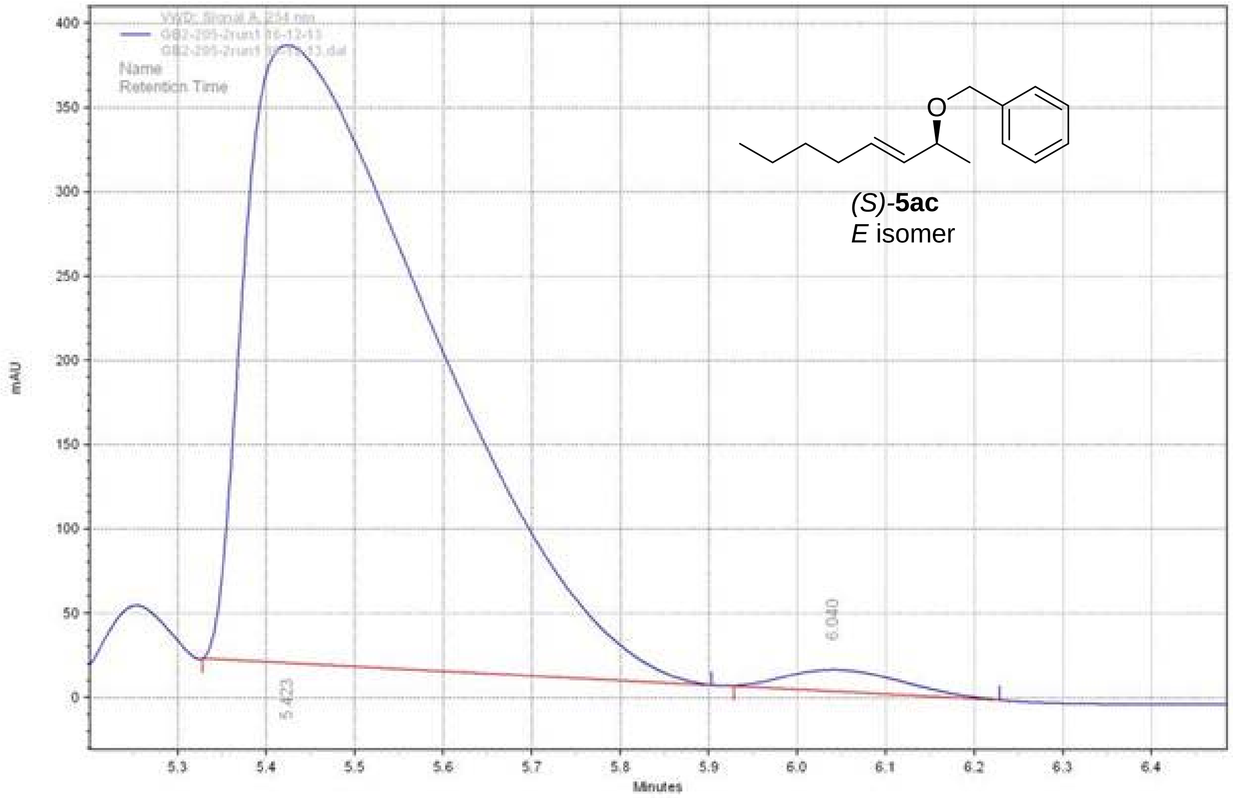

C:\EZChrom Elite\Enterprise\Projects\Default\Data\GB2-205-2run1 16-12-13.dat, VWD: Signal A, 254 nm

VWD: Signal A,
254 nm Results

Retention Time	Area	Area %	Height	Height %
5.423	90946394	97.90	6144874	96.66
6.040	1953850	2.10	212367	3.34

(*S*, *Z*)-((Oct-3-en-2-yloxy)methyl)benzene (*S*)-5ac

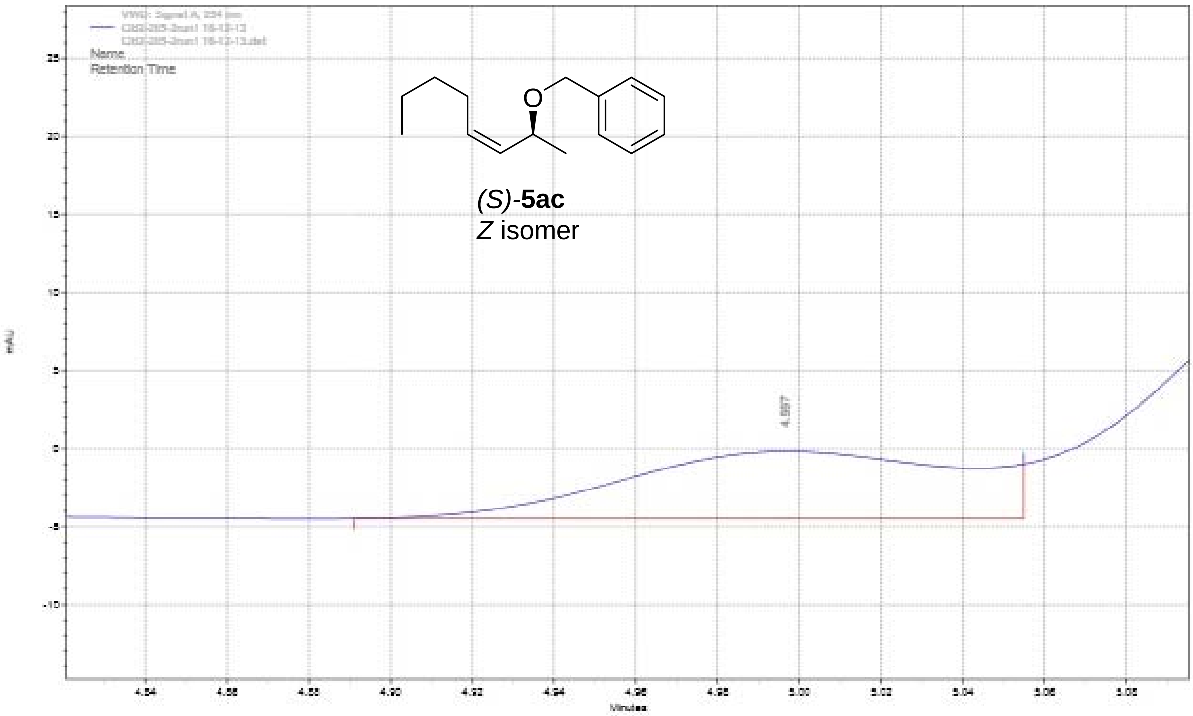

C:\EZChrom Elite\Enterprise\Projects\Default\Data\GB2-205-2run1 16-12-13.dat, VWD: Signal A, 254 nm

VWD: Signal A, 254 nm Results

Retention Time	Area	Area %	Height	Height %
4.997	413688	100.00	72018	100.00

(*S*,*E*)-1-Bromo-4-((oct-3-en-2-yloxy)methyl)benzene (*S*)-5ad

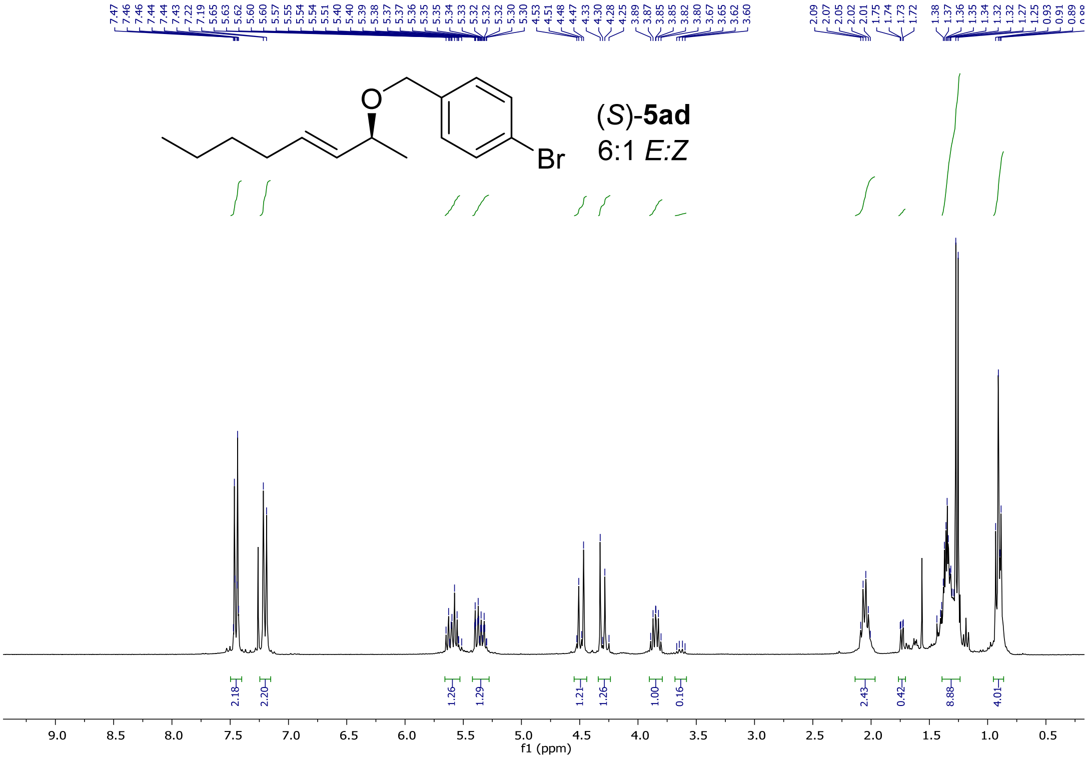

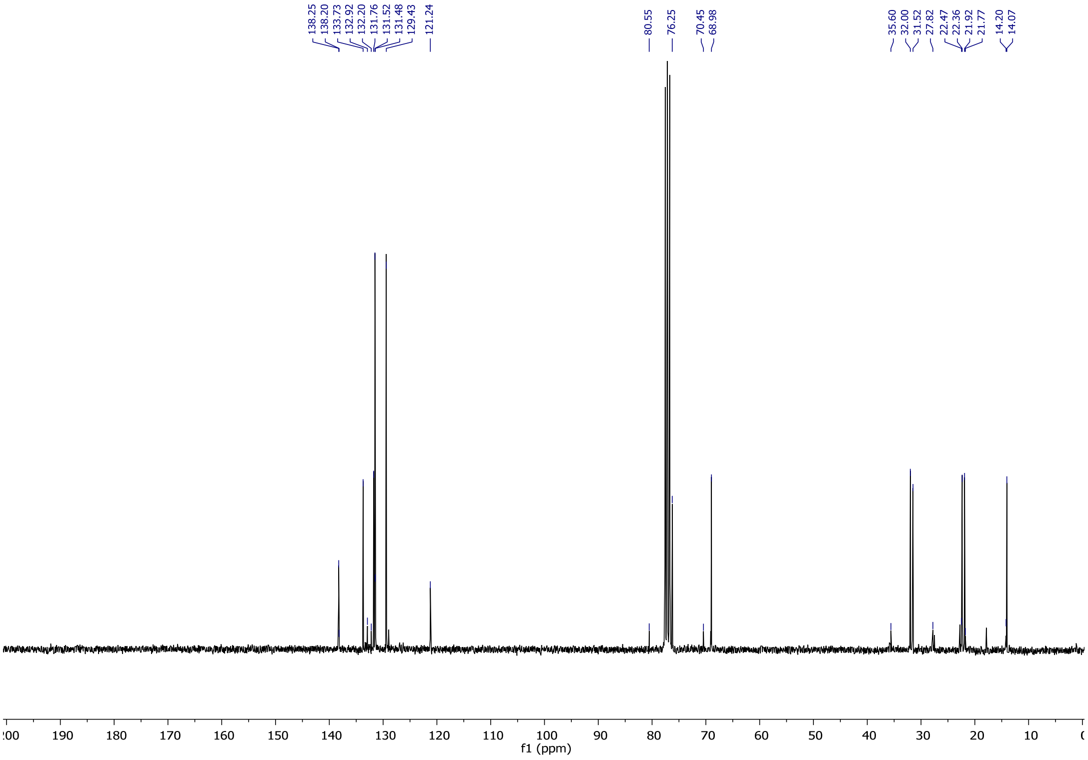

(*S*,*E*)-((Oct-3-en-2-yloxy)methyl)benzene (*S*)-5ac from (*S*,*E*)-1-Bromo-4-((oct-3-en-2-yloxy)methyl)benzene (*S*)-5ad (to determine er of (*S*)-**5ad**)

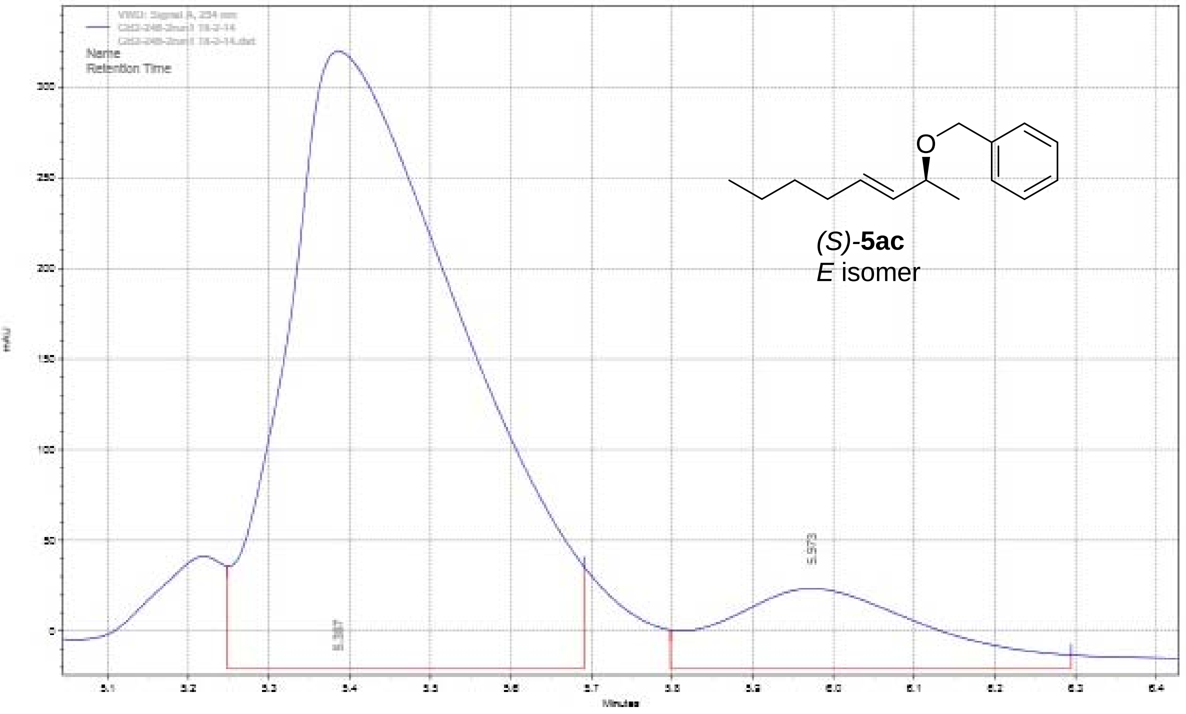

C:\EZChrom Elite\Enterprise\Projects\Default\Data\GB2-246-2run1 18-2-14.dat, VWD: Signal A, 254 nm

VWD: Signal A, 254 nm Results

Retention Time	Area	Area %	Height	Height %
5.387	86359973	86.91	5712353	88.56
5.973	13009552	13.09	737849	11.44

(*S*,*Z*)-((Oct-3-en-2-yloxy)methyl)benzene (*S*)-5ac from (*S*,*Z*)-1-Bromo-4-((oct-3-en-2-yloxy)methyl)benzene (*S*)-5ad (to determine er of (*S*)-**5ad**)

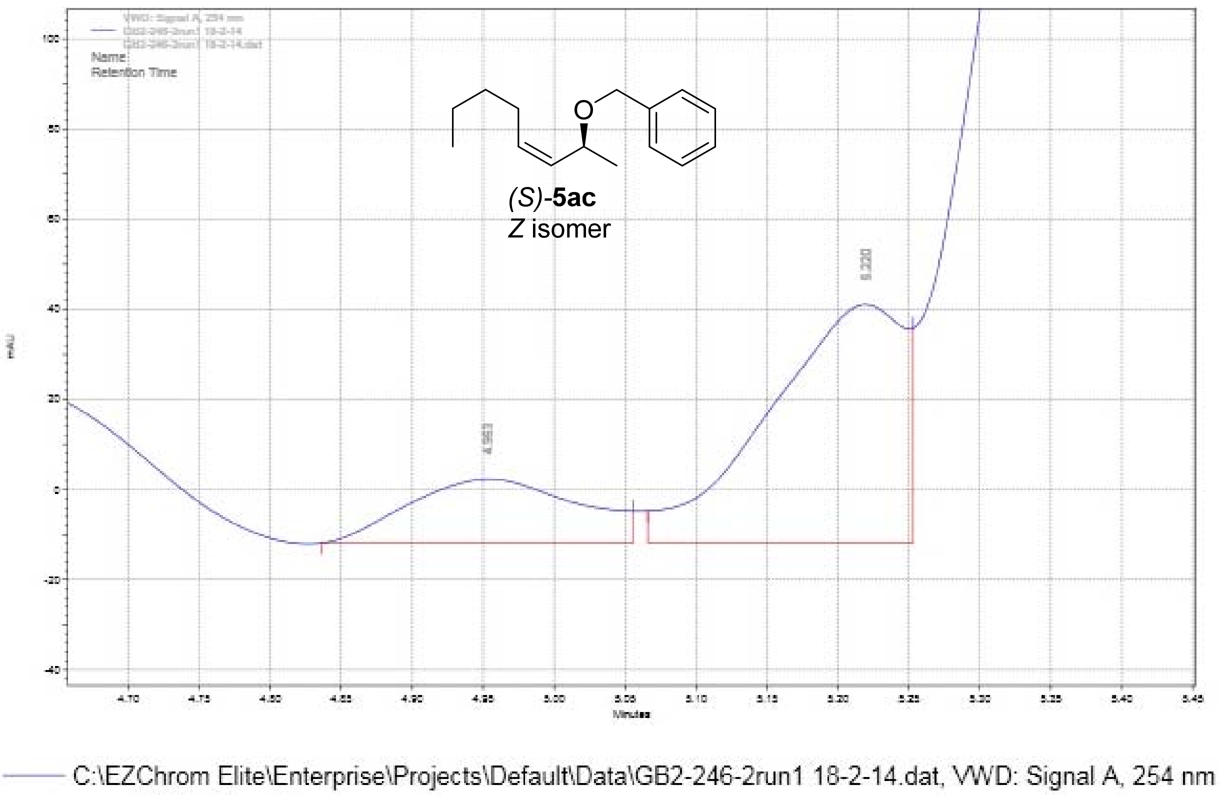

VWD: Signal A,
254 nm Results

Retention Time	Area	Area %	Height	Height %
4.953	1971993	25.15	238005	21.15
5.220	5868991	74.85	887400	78.85

(*S*,*E*)-1-Methoxy-4-((oct-3-en-2-yloxy)methyl)benzene (*S*)-5ae

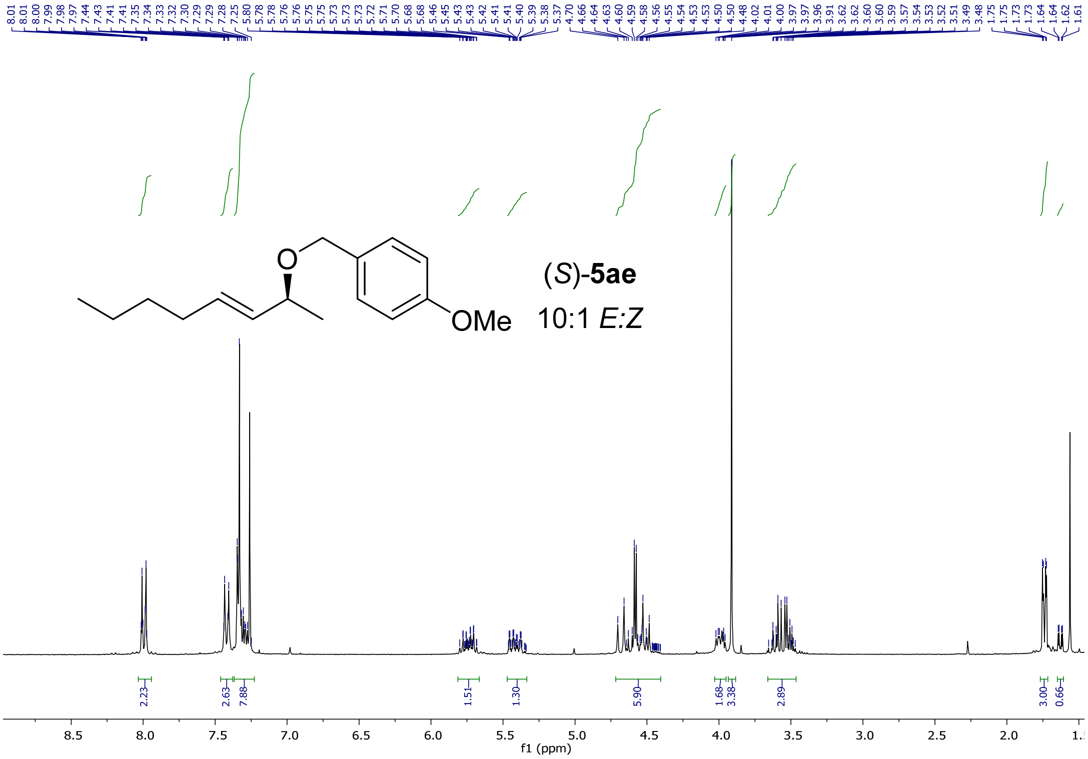

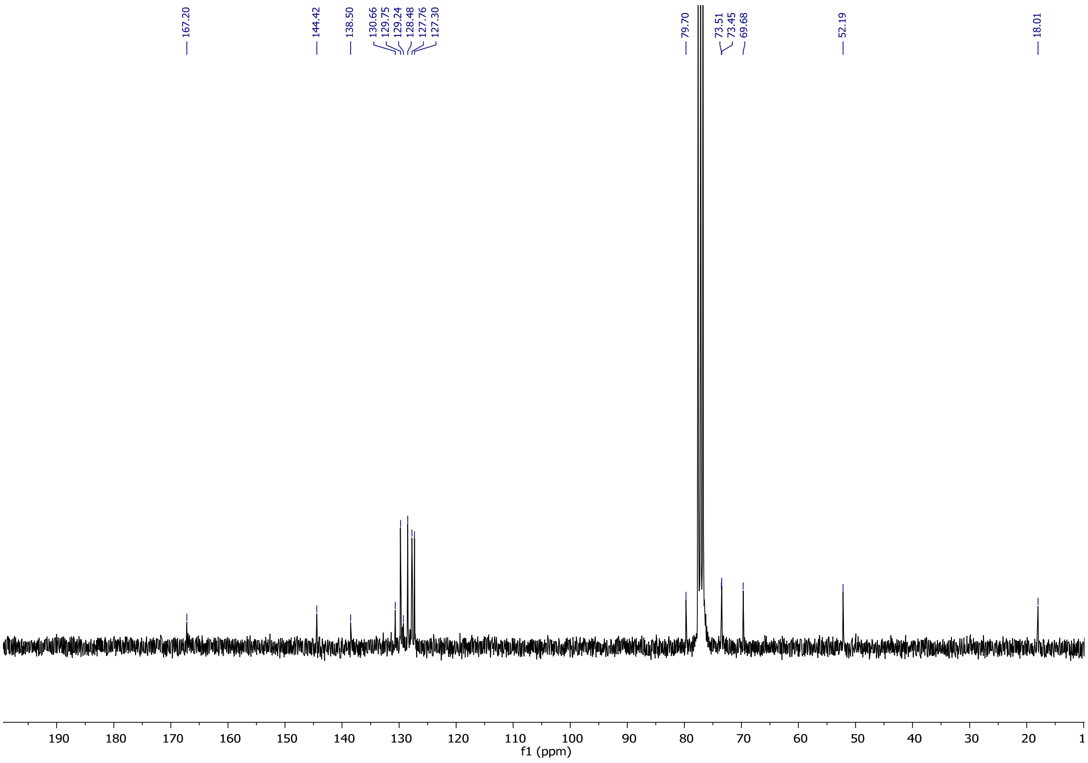

(*E*)-1-Methoxy-4-((oct-3-en-2-yloxy)methyl)benzene *Rac*-5ae + Chiral Shift Reagent

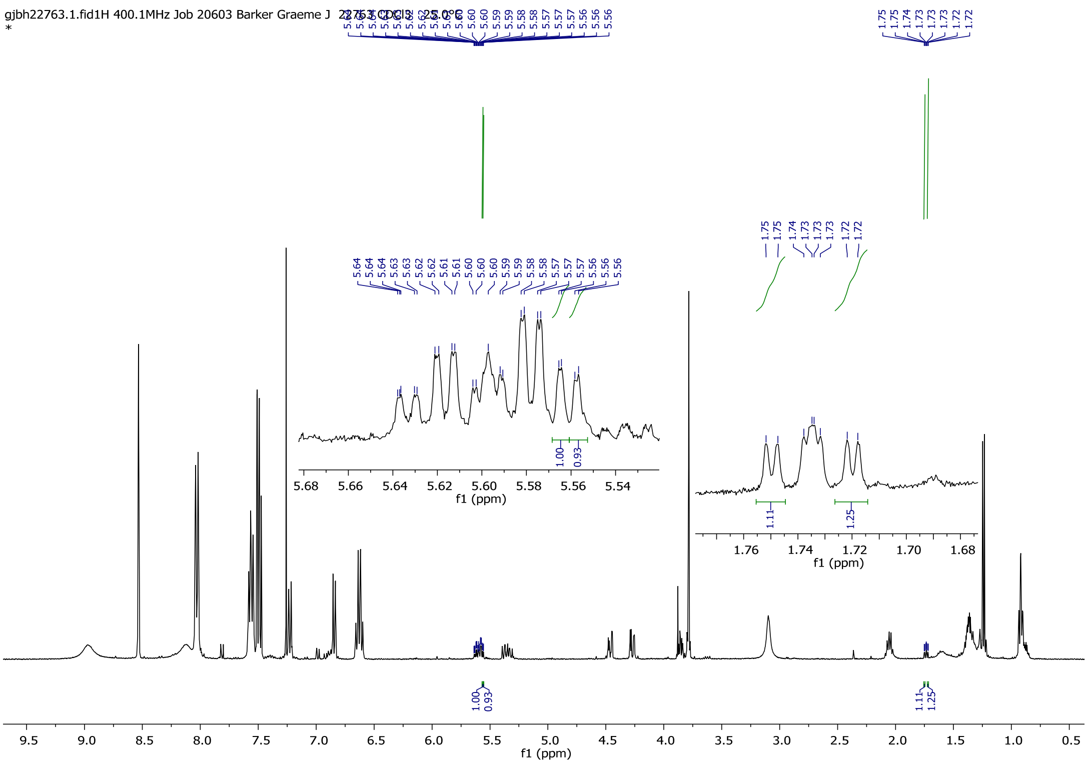

(*S*,*E*)-1-Methoxy-4-((oct-3-en-2-yloxy)methyl)benzene (*S*)-5ae + Chiral Shift Reagent

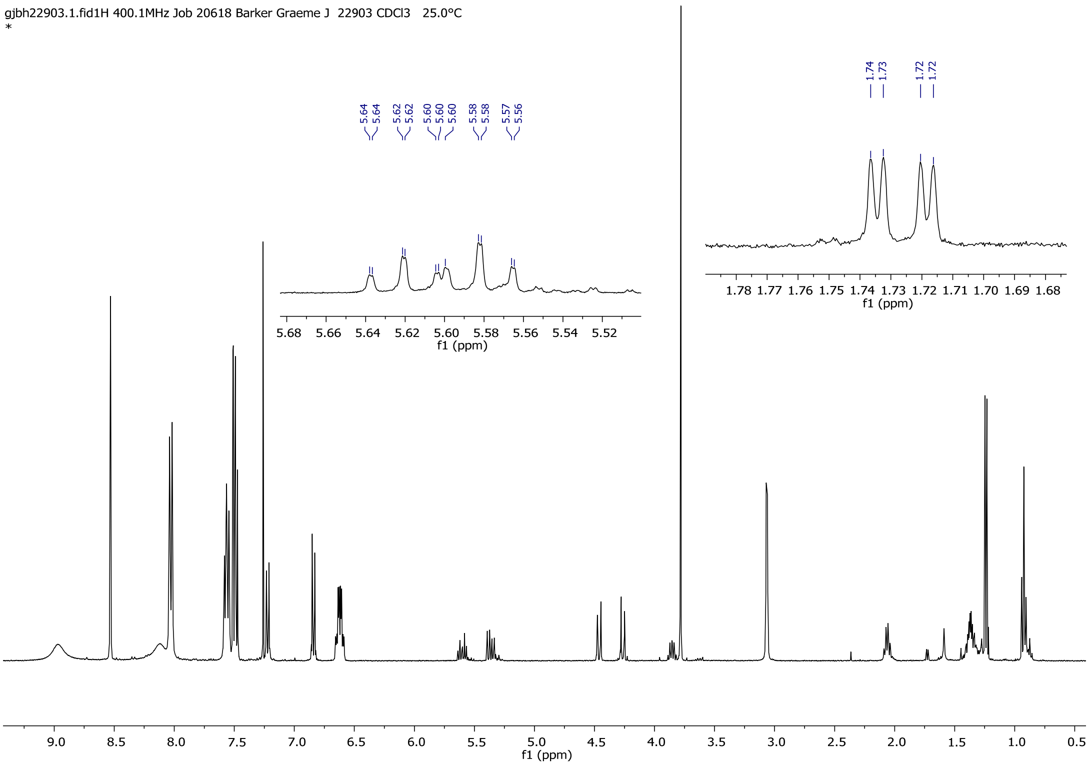

(*S*,*E*)-2-((Oct-3-en-2-yloxy)methyl)furan (*S*)-5af

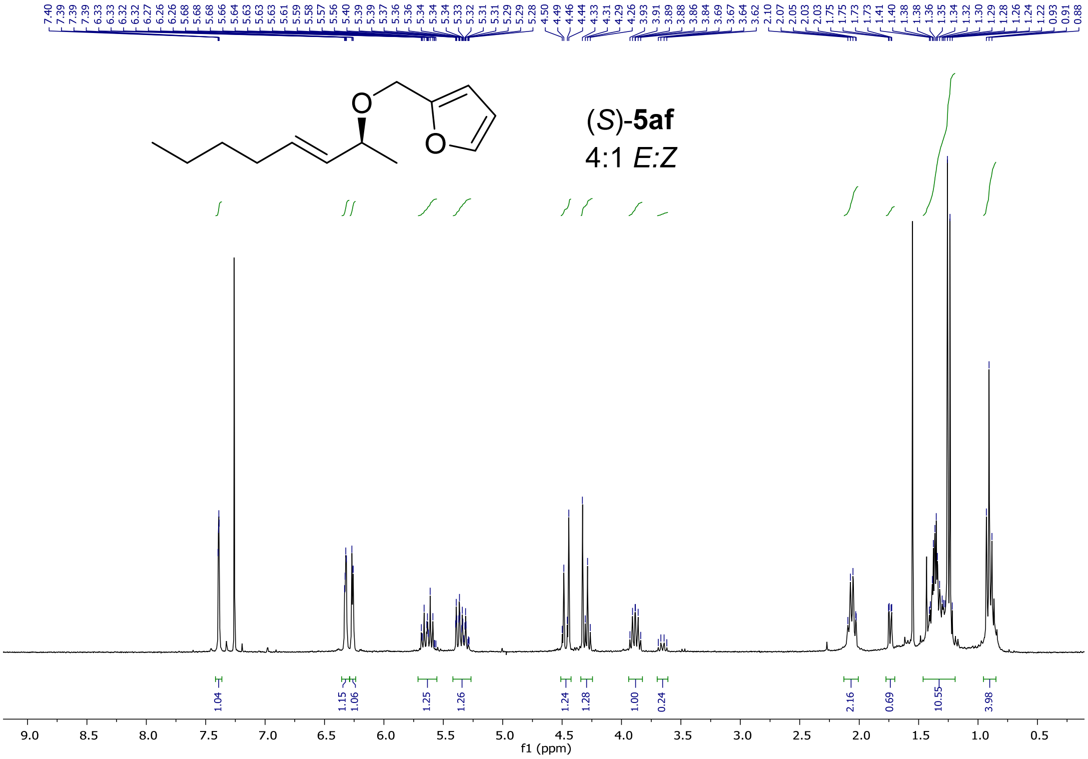

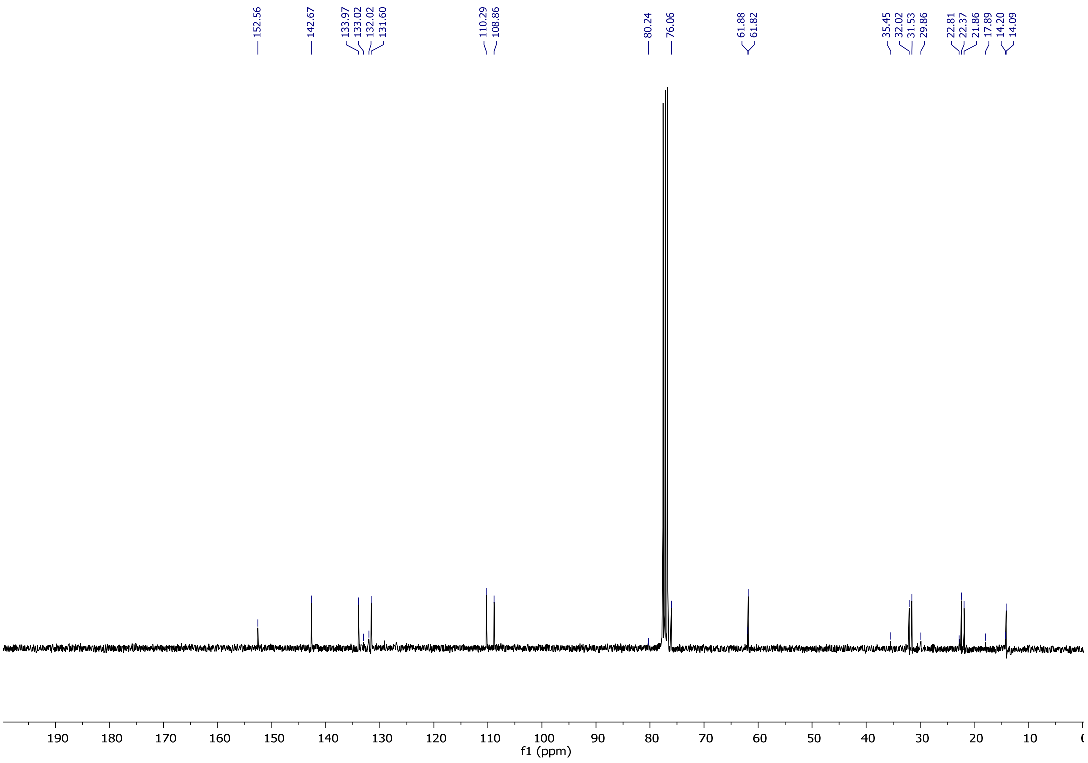

(*E*)-2-((Oct-3-en-2-yloxy)methyl)furan *Rac*-5af

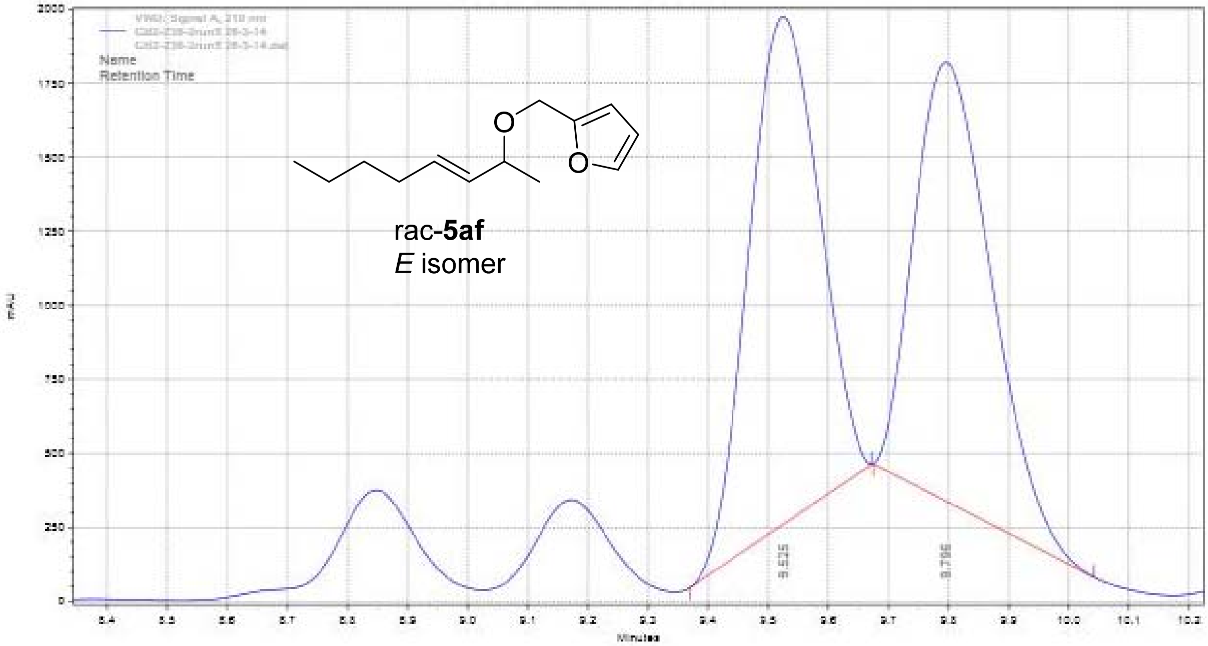

C:\EZChrom Elite\Enterprise\Projects\Default\Data\GB2-236-2run5 26-3-14.dat, VWD: Signal A, 210 nm

VWD: Signal A,
210 nm Results

Retention Time	Area	Area %	Height	Height %
9.525	234480192	51.26	28728948	53.62
9.795	222961669	48.74	24847776	46.38

(*Z*)-2-((Oct-3-en-2-yloxy)methyl)furan *Rac*-5af

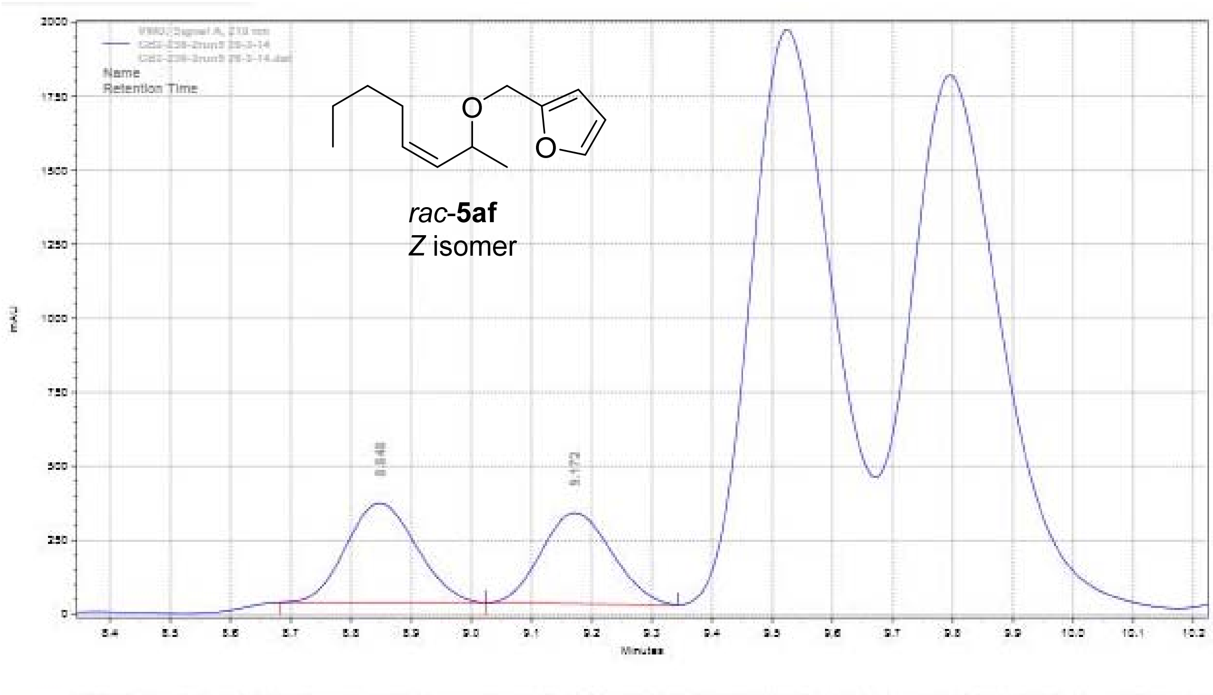

C:\EZChrom Elite\Enterprise\Projects\Default\Data\GB2-236-2run5 26-3-14.dat, VWD: Signal A, 210 nm

VWD: Signal A, 210 nm Results

Retention Time	Area	Area %	Height	Height %
8.848	45695554	52.20	5629971	52.21
9.172	41835756	47.80	5153525	47.79

(*S*,*E*)-2-((Oct-3-en-2-yloxy)methyl)furan (*S*)-5af

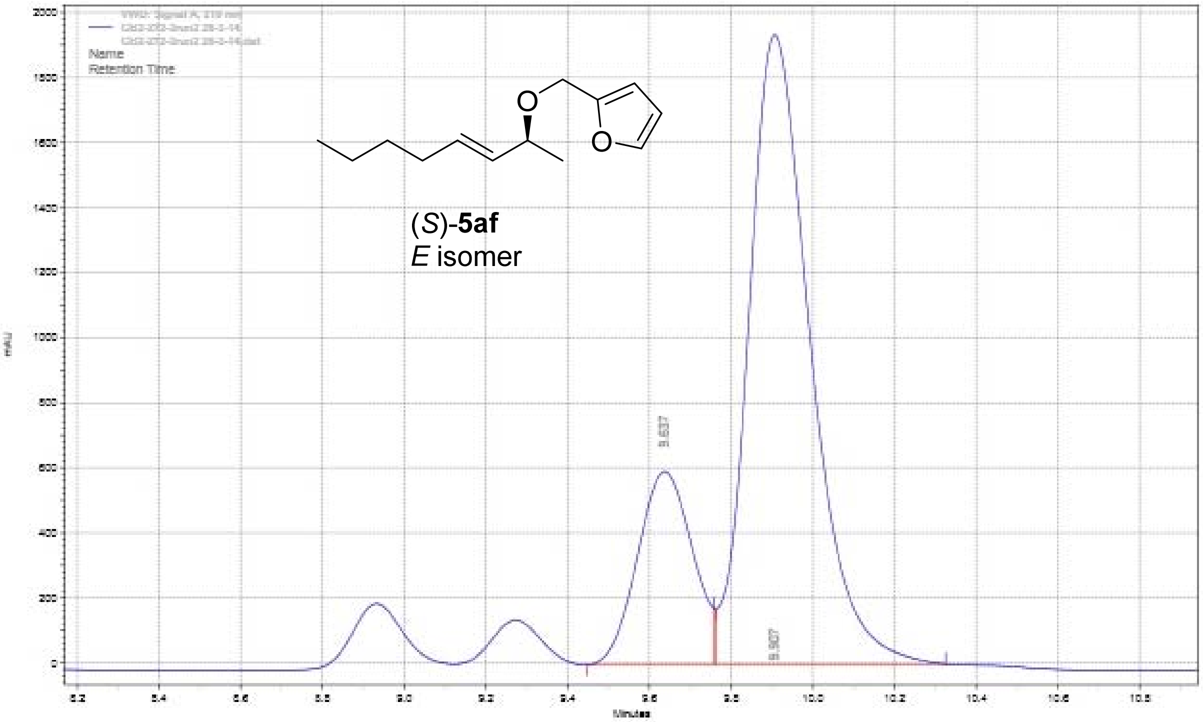

C:\EZChrom Elite\Enterprise\Projects\Default\Data\GB2-272-2run2 26-3-14.dat, VWD: Signal A, 210 nm

VWD: Signal A, 210 nm Results

Retention Time	Area	Area %	Height	Height %
9.637	93208721	21.47	9975619	23.49
9.907	340837878	78.53	32490010	76.51

(*S*,*Z*)-2-((Oct-3-en-2-yloxy)methyl)furan (*S*)-5af

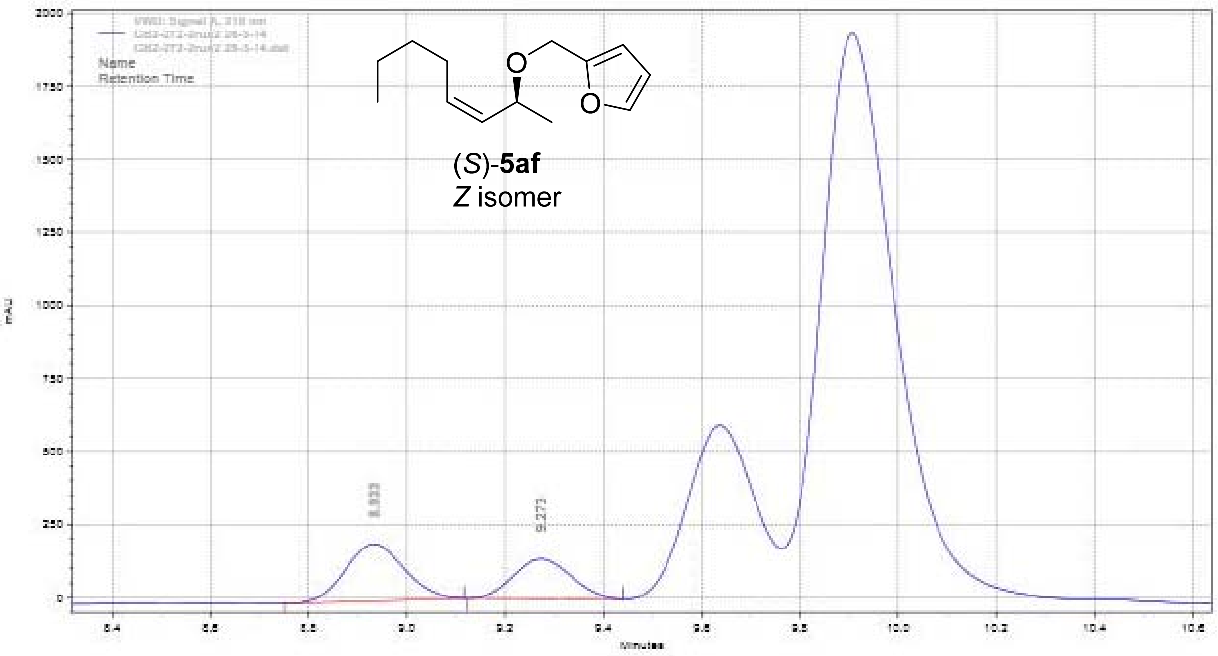

C:\EZChrom Elite\Enterprise\Projects\Default\Data\GB2-272-2run2 26-3-14.dat, VWD: Signal A, 210 nm

VWD: Signal A, 210 nm Results

Retention Time	Area	Area %	Height	Height %
8.933	27249855	59.29	3255733	58.72
9.273	18710525	40.71	2288829	41.28

(*S*, *E*)-(3-(Oct-3-en-2-yloxy)propyl)benzene (*S*)-5ag

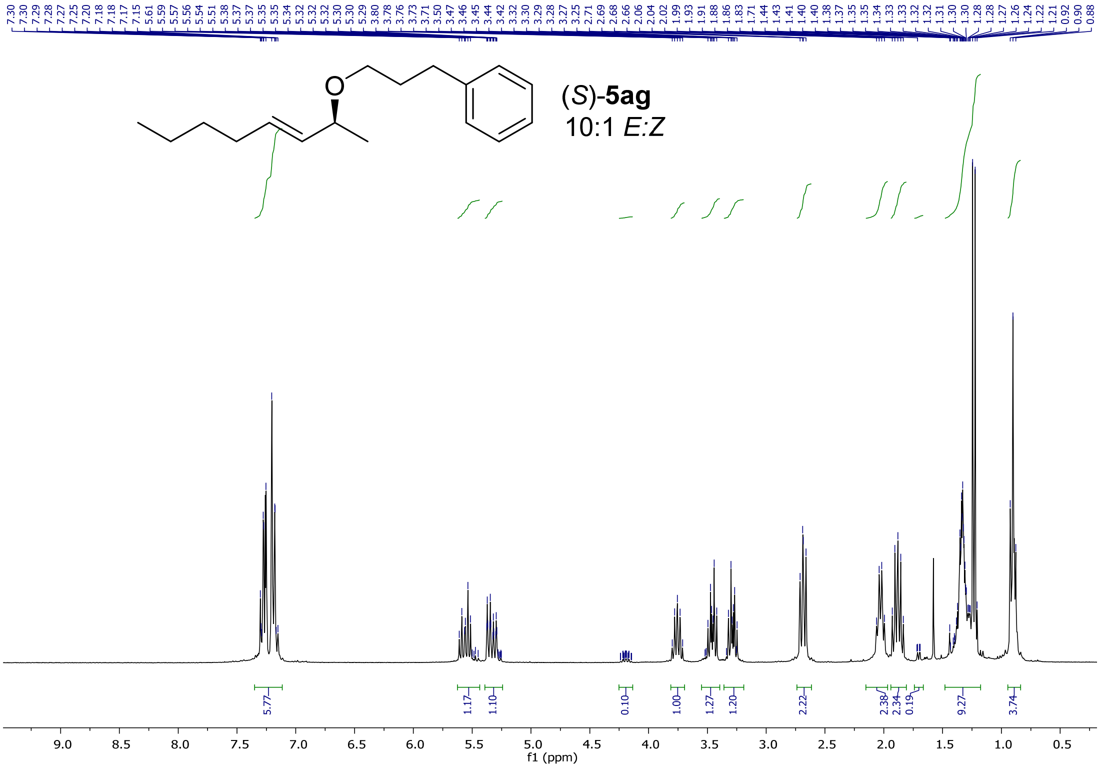

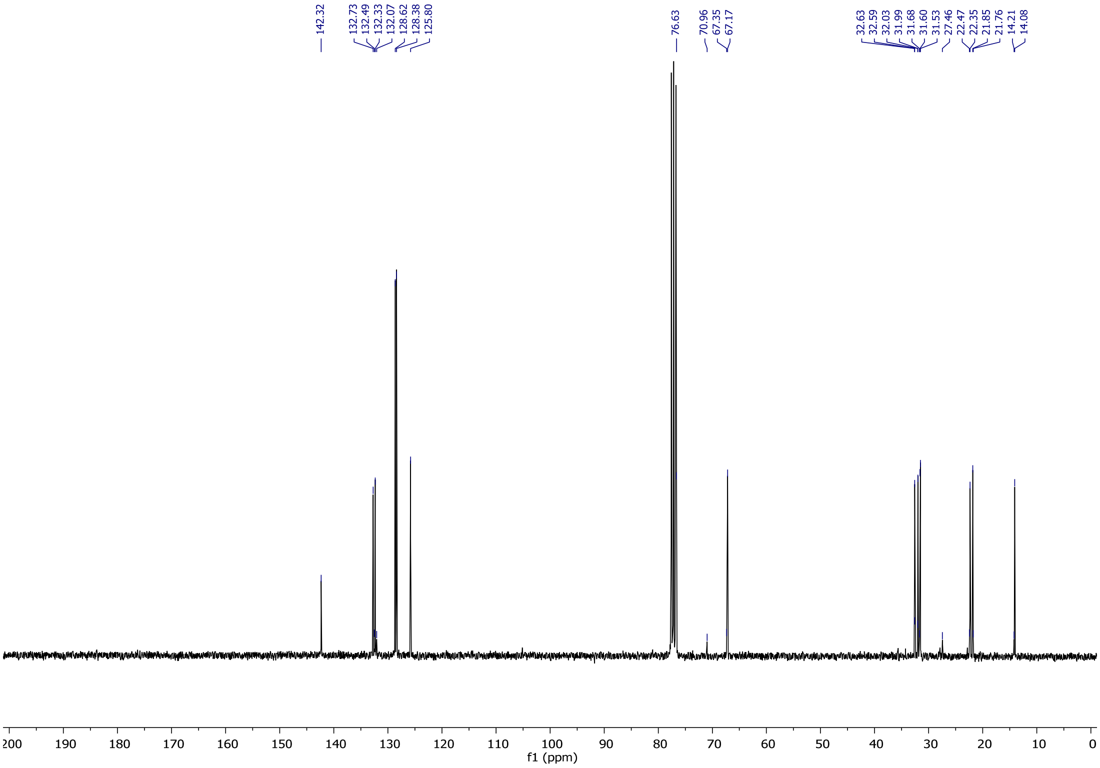

Rac-(_E_)-(3-(Oct-3-en-2-yloxy)propyl)benzene _rac_-5ag

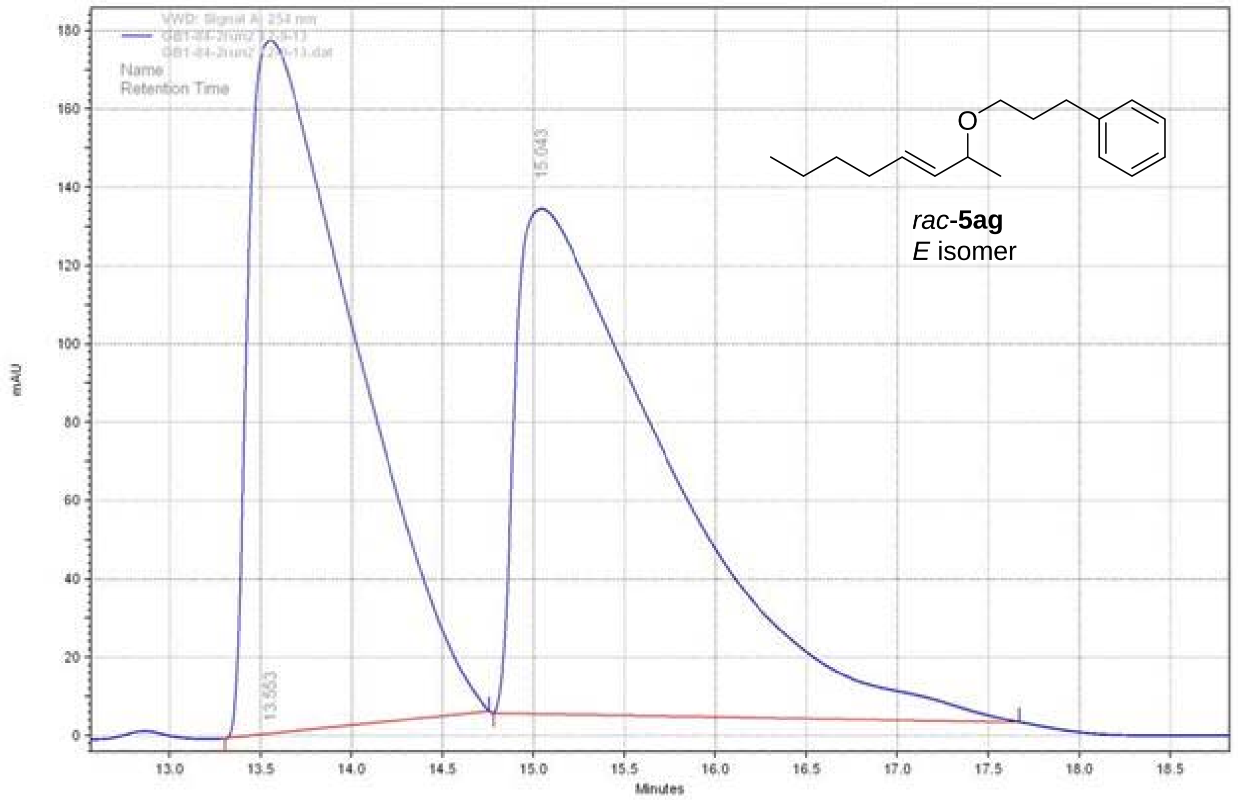

C:\EZChrom Elite\Enterprise\Projects\Default\Data\GB1-84-2run2 12-9-13.dat, VWD: Signal A, 254 nm

VWD: Signal A,
254 nm Results

Retention Time	Area	Area %	Height	Height %
13.553	121132791	48.98	2971006	57.88
15.043	126186425	51.02	2162081	42.12

***Rac*-(*Z*)-(3-(Oct-3-en-2-yloxy)propyl)benzene *rac*-5ag**

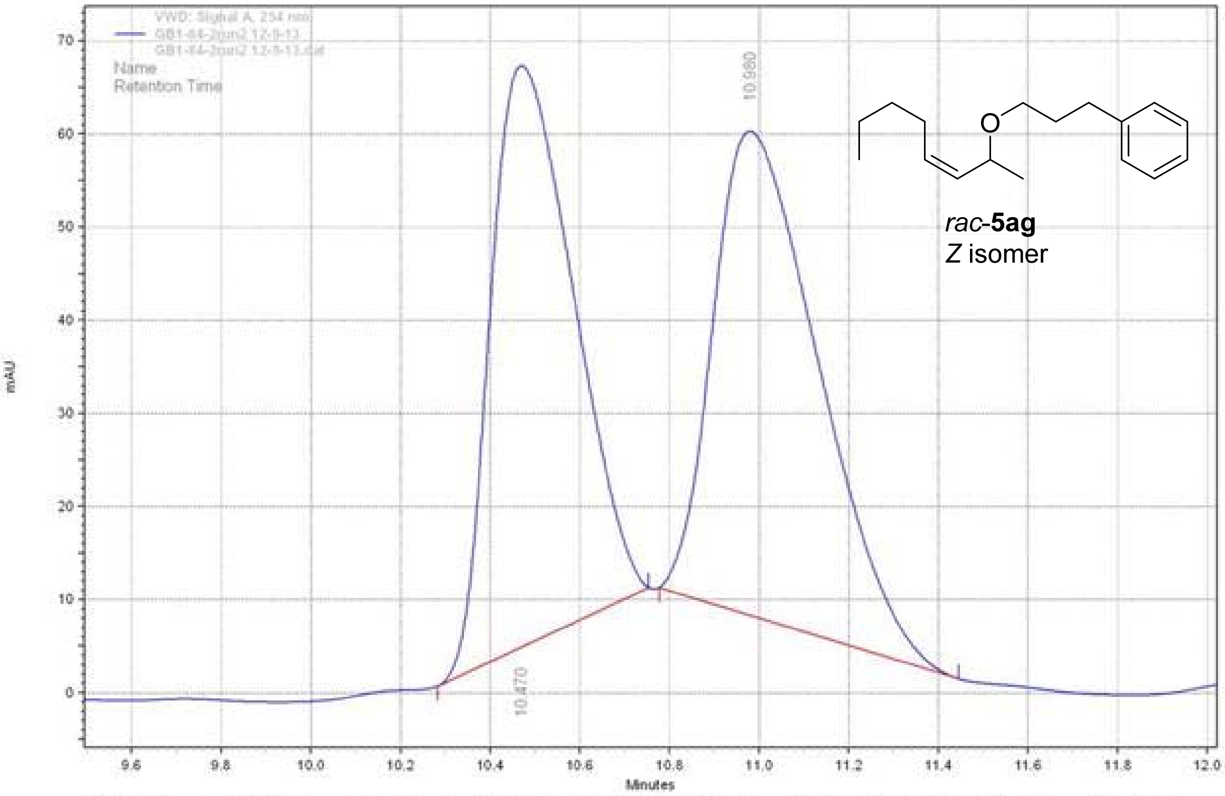

C:\EZChrom Elite\Enterprise\Projects\Default\Data\GB1-84-2run2 12-9-13.dat, VWD: Signal A, 254 nm

VWD: Signal A, 254 nm Results

Retention Time	Area	Area %	Height	Height %
10.470	13416465	48.31	1047041	54.56
10.980	14353045	51.69	871859	45.44

(*S*, *E*)-(3-(Oct-3-en-2-yloxy)propyl)benzene (*S*)-5ag

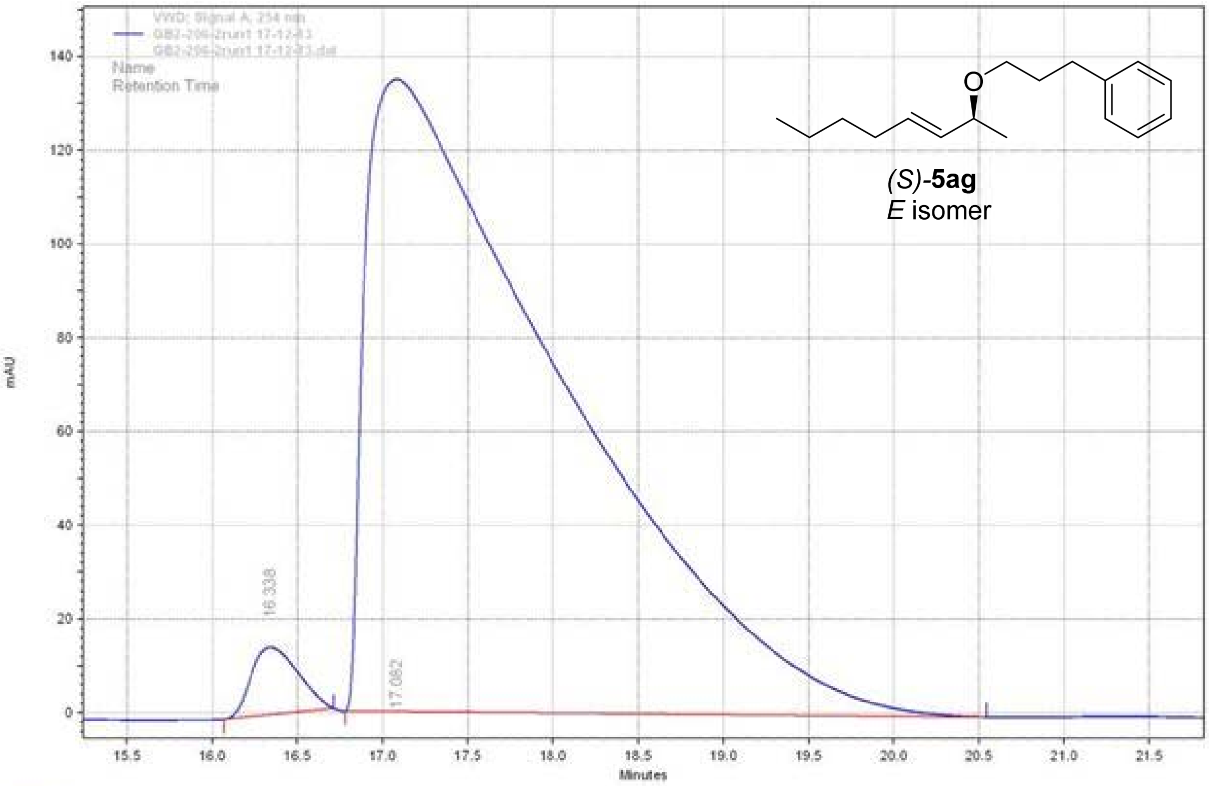

C:\EZChrom Elite\Enterprise\Projects\Default\Data\GB2-206-2run1 17-12-13.dat, VWD: Signal A, 254 nm

VWD: Signal A, 254 nm Results

Retention Time	Area	Area %	Height	Height %
16.338	4591030	2.45	242322	9.67
17.082	182953540	97.55	2263541	90.33

(*S*, *Z*)-(3-(Oct-3-en-2-yloxy)propyl)benzene (*S*)-5ag

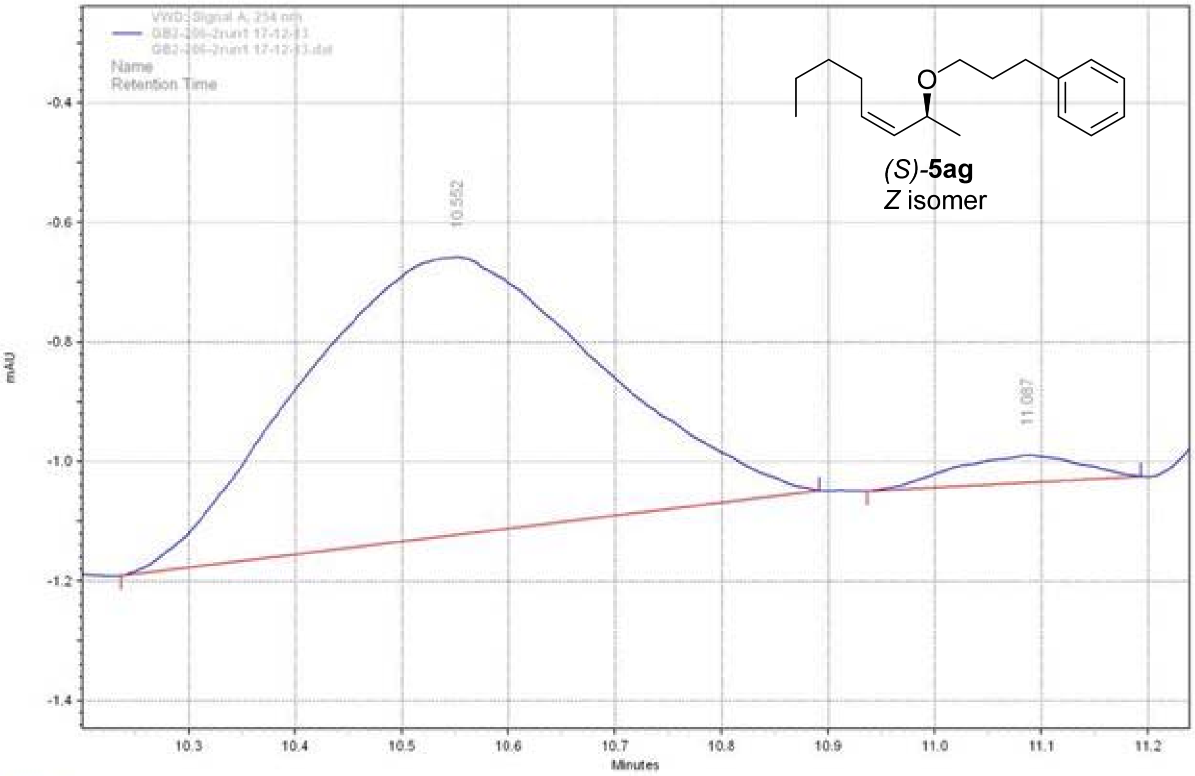

C:\EZChrom Elite\Enterprise\Projects\Default\Data\GB2-206-2run1 17-12-13.dat, VWD: Signal A, 254 nm

VWD: Signal A,
254 nm Results

Retention Time	Area	Area %	Height	Height %
10.552	151361	96.00	7779	91.07
11.087	6314	4.00	763	8.93

(*S*,*E*)-2-(3,3,3-Trifluoropropoxy)oct-3-ene (*S*)-5ah

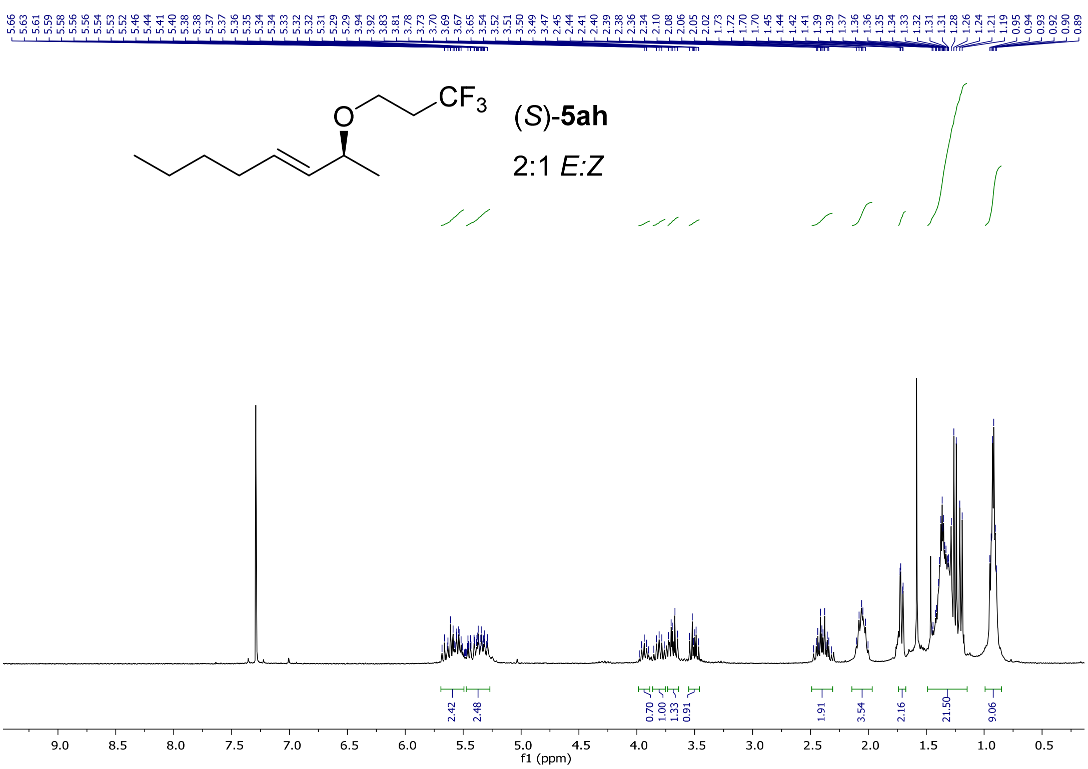

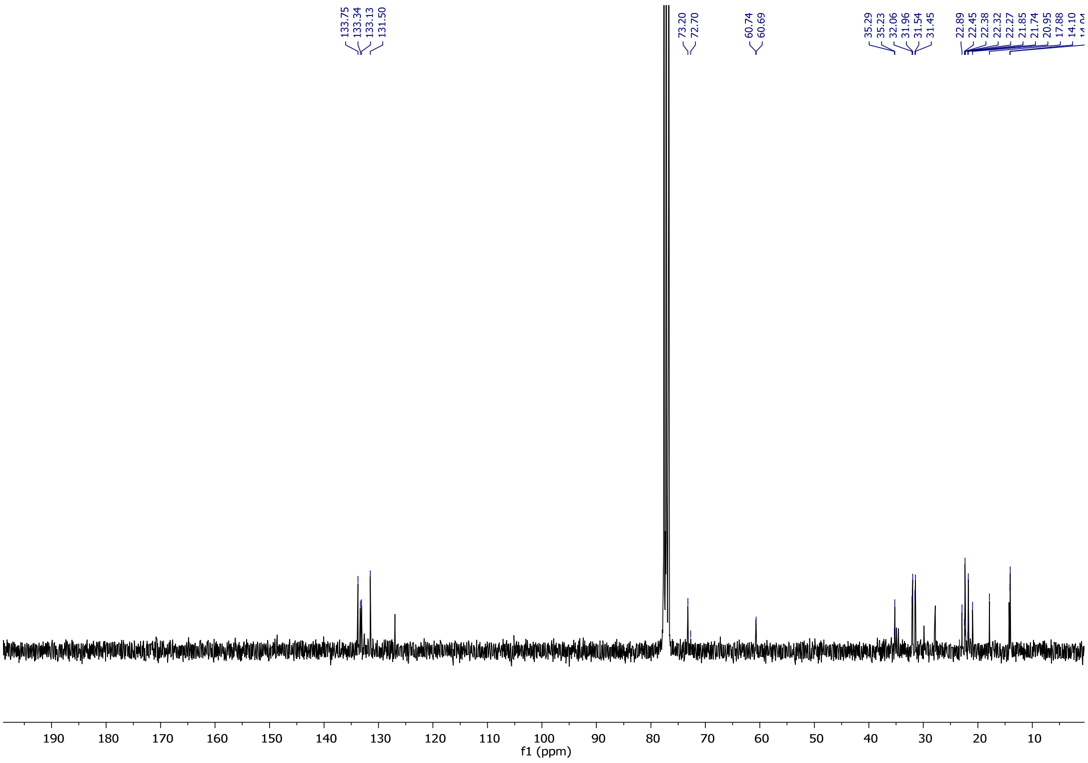

(*E*)-2-(3,3,3-Trifluoropropoxy)oct-3-ene *Rac*-5ah

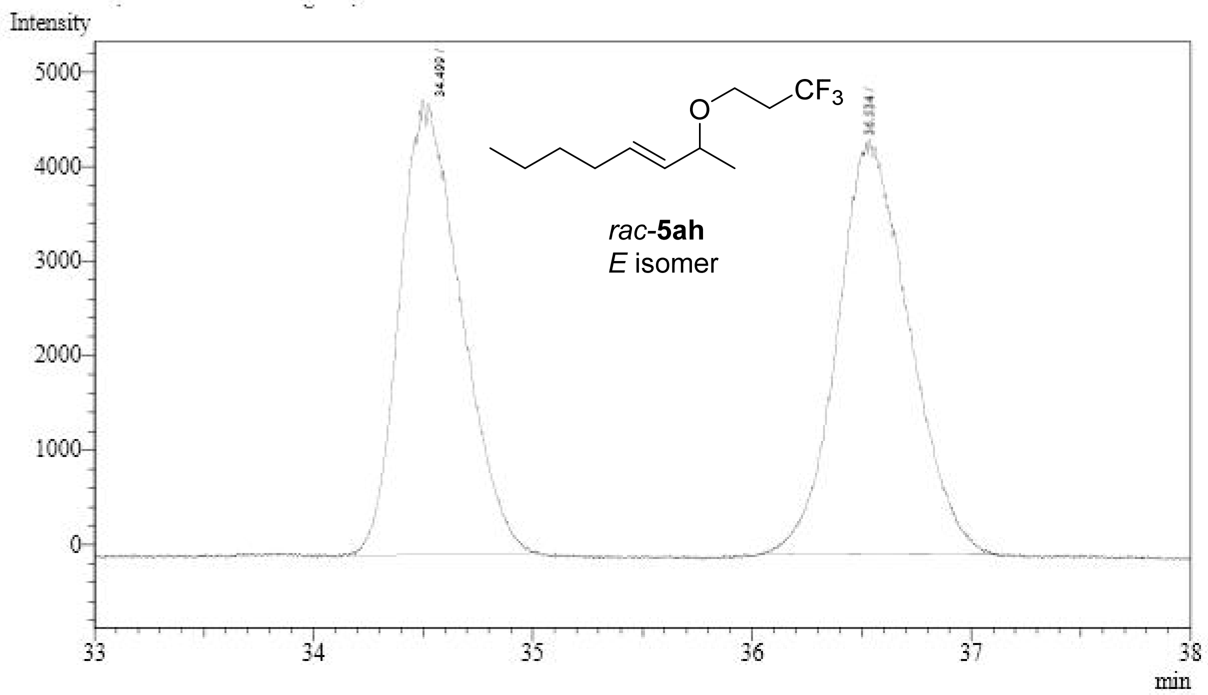

Peak#	Ret.Time	Area	Height	Conc.	Unit	Mark	ID#	Cmpd Name	Area%
1	34.499	93265	4793	48.237					48.2371
2	36.534	100082	4376	51.763					51.7629
**Total**		193347	9169						100.0000

(*Z*)-2-(3,3,3-Trifluoropropoxy)oct-3-ene *Rac*-XX

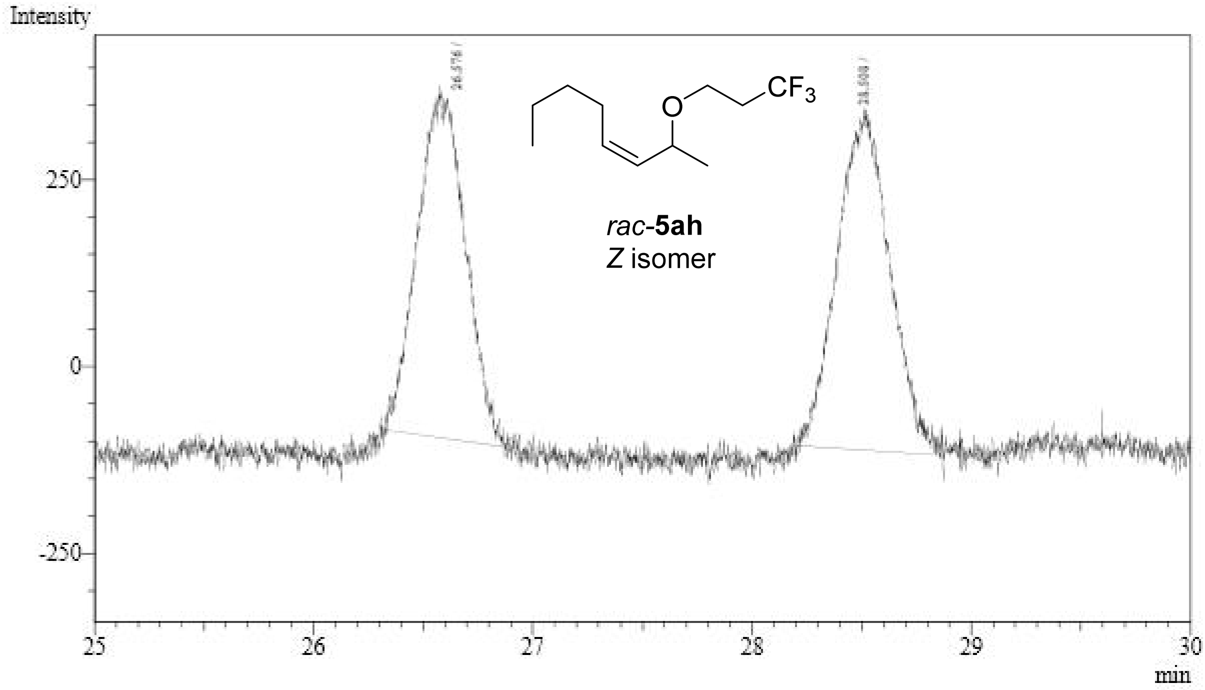

Peak#	Ret.Time	Area	Height	Conc.	Unit	Mark	ID#	Cmpd Name	Area%
1	26.576	7055	459	48.267					48.2669
2	28.508	7562	447	51.733					51.7331
**Total**		14617	906						100.0000

(*S*,*E*)-2-(3,3,3-Trifluoropropoxy)oct-3-ene (*S*)-5ah

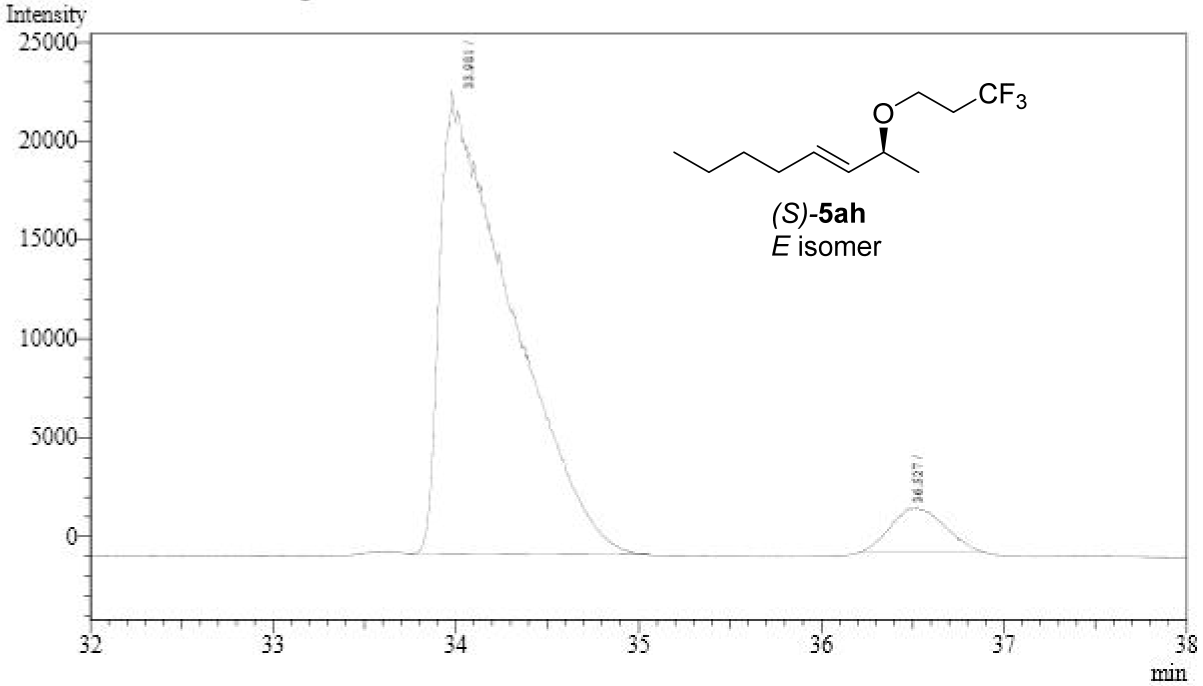

Peak#	Ret.Time	Area	Height	Conc.	Unit	Mark	ID#	Cmpd Name	Area%
1	33.981	637093	23287	93.261					93.2611
2	36.527	46035	2278	6.739					6.7389
**Total**		683128	25565						100.0000

(*S*,*Z*)-2-(3,3,3-Trifluoropropoxy)oct-3-ene (*S*)-5ah

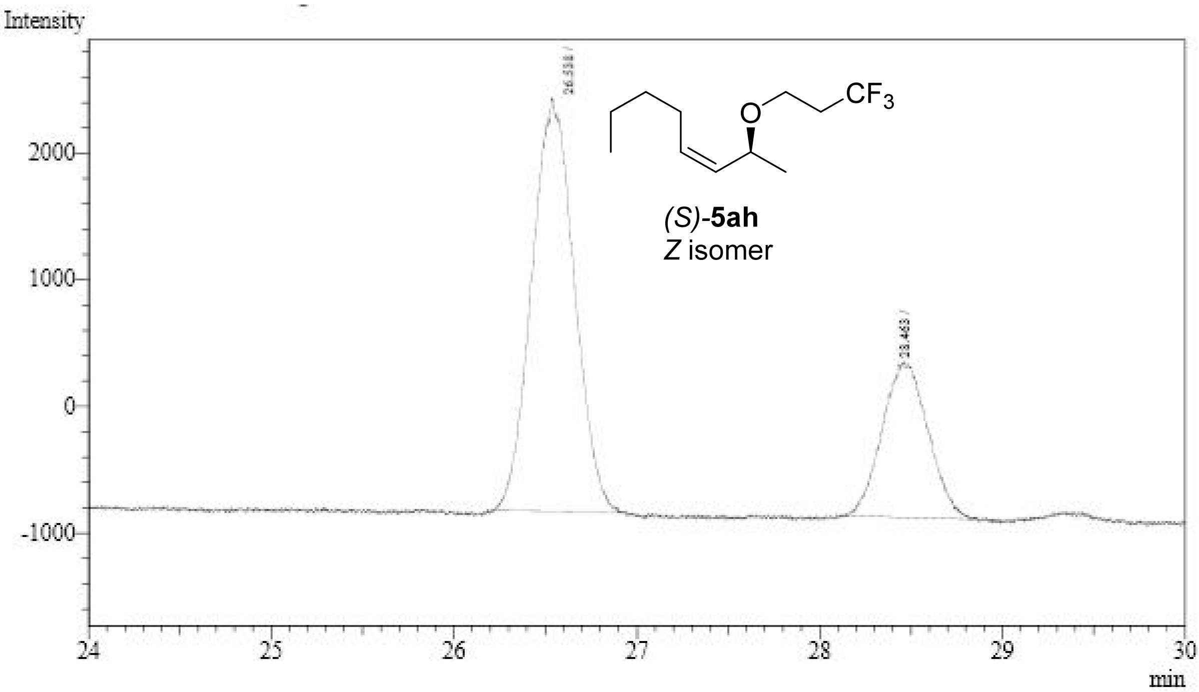

Peak#	Ret.Time	Area	Height	Conc.	Unit	Mark	ID#	Cmpd Name	Area%
1	26.538	52742	3257	71.170					71.1701
2	28.463	21365	1222	28.830					28.8299
Total		74107	4479						100.0000

(*S*,*E*)-2-(3-Chloropropoxy)oct-3-ene (*S*)-5ai

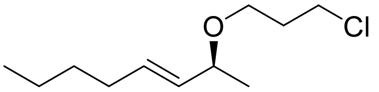

(*S*)-**5ai**

6:1 *E*:*Z*

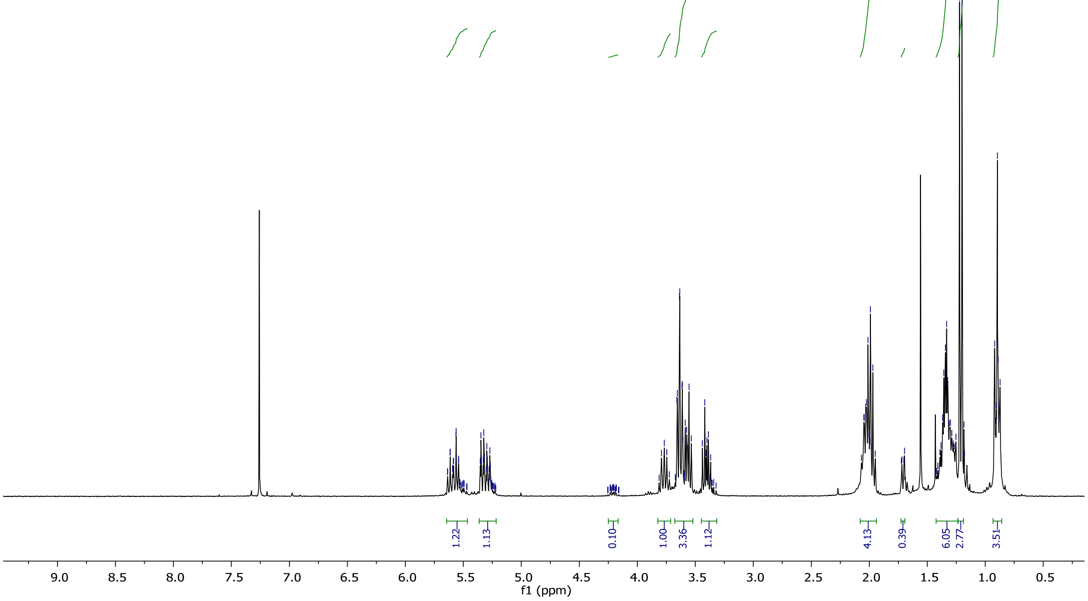

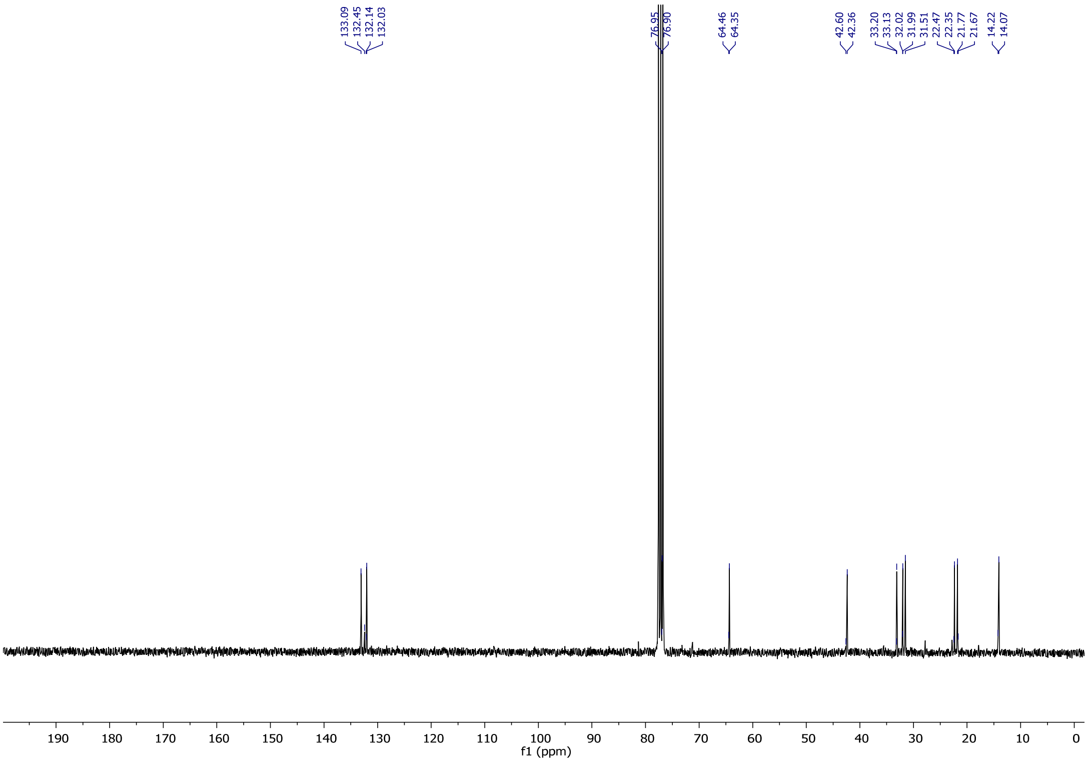

(*E*)-2-(3-Chloropropoxy)oct-3-ene *Rac*-5ai

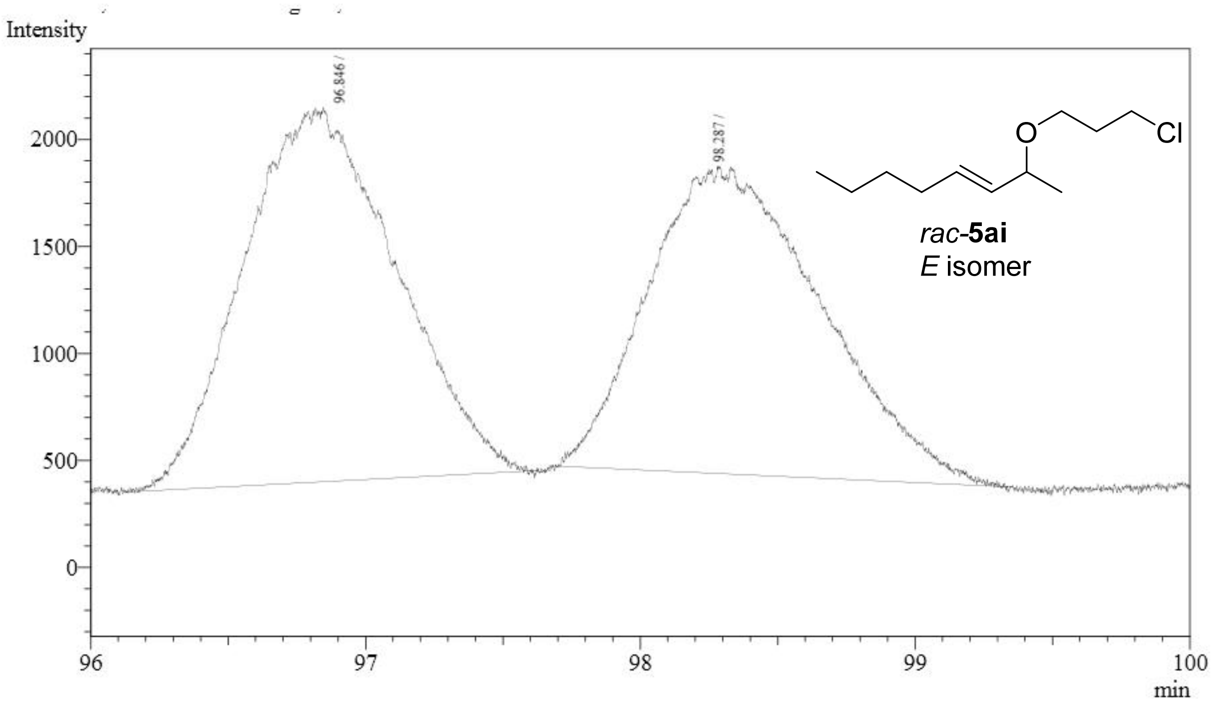

Peak#	Ret.Time	Area	Height	Conc.	Unit	Mark	ID#	Cmpd Name	Area%
1	96.846	67479	1742	51.745					51.7453
2	98.287	62927	1431	48.255					48.2547
Total		130406	3173						100.0000

(*Z*)-2-(3-Chloropropoxy)oct-3-ene *Rac*-5ai

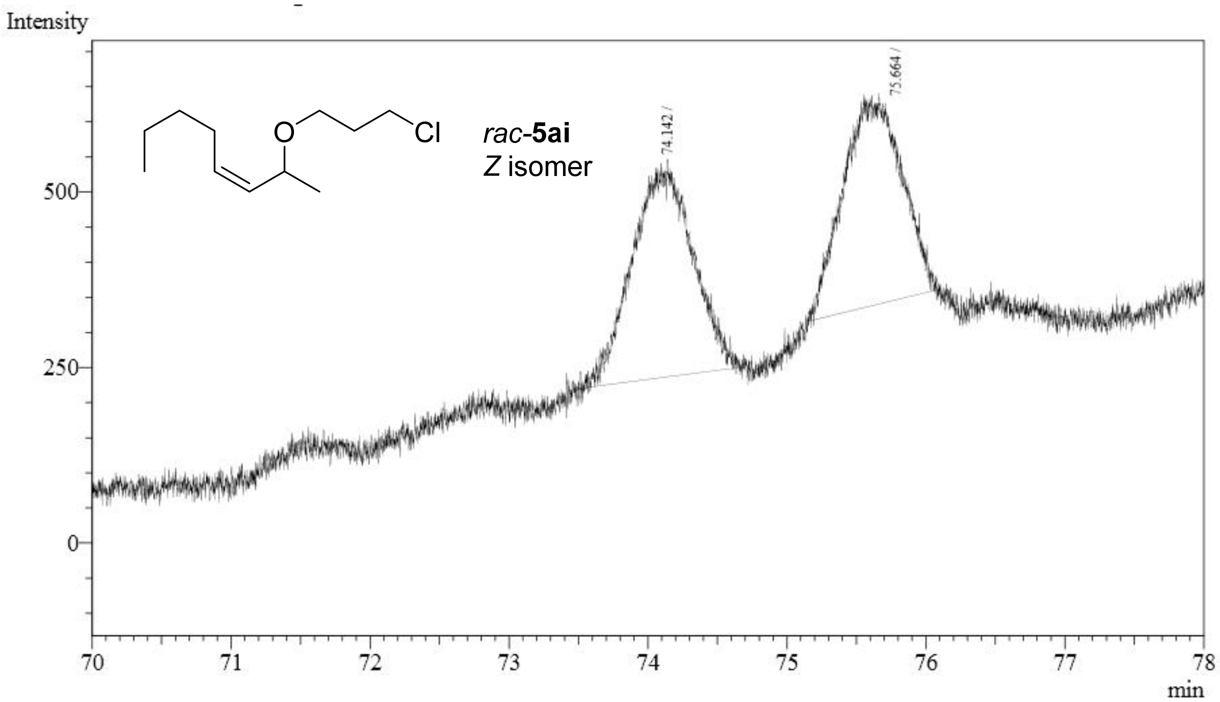

Peak#	Ret.Time	Area	Height	Conc.	Unit	Mark	ID#	Cmpd Name	Area%
1	74.142	9184	300	51.201					51.2011
2	75.664	8753	293	48.799					48.7989
Total		17937	593						100.0000

(*S*,*E*)-2-(3-Chloropropoxy)oct-3-ene (*S*)-5ai

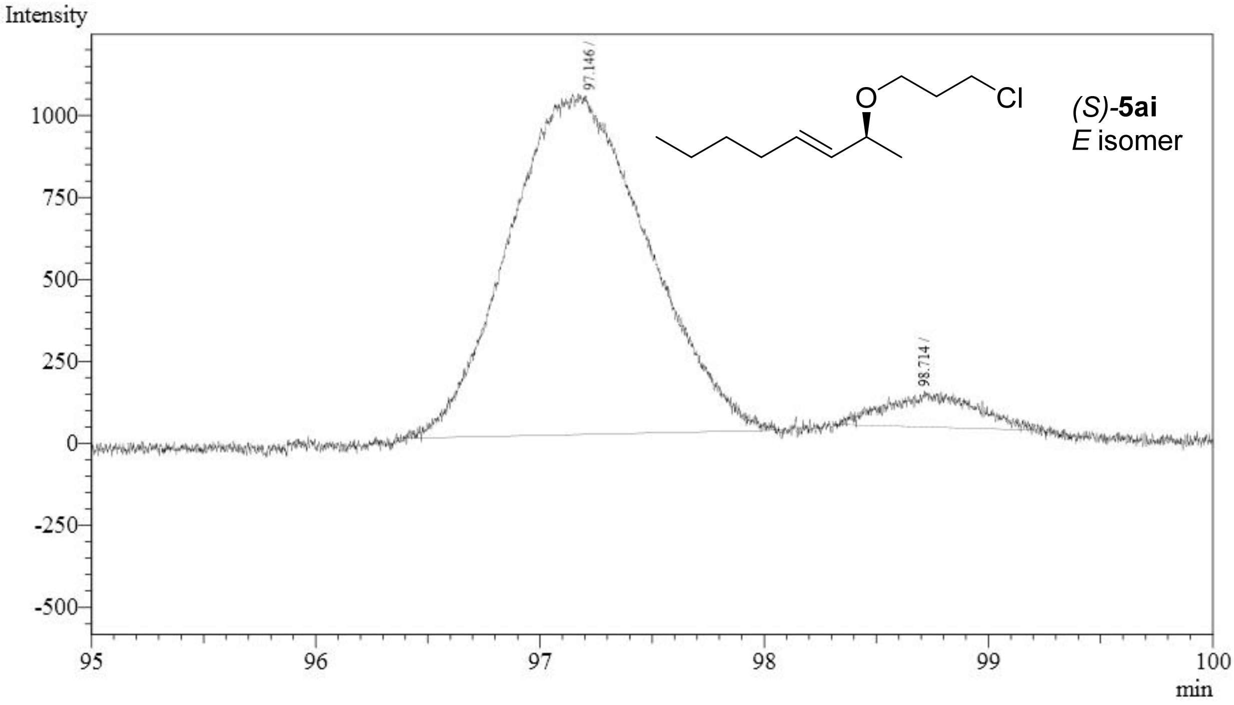

Peak#	Ret.Time	Area	Height	Conc.	Unit	Mark	ID#	Cmpd Name	Area%
1	97.146	44562	1035	94.063					94.0634
2	98.714	2812	100	5.937					5.9366
**Total**		47374	1135						100.0000

(*S*,*Z*)-2-(3-Chloropropoxy)oct-3-ene (*S*)-5ai

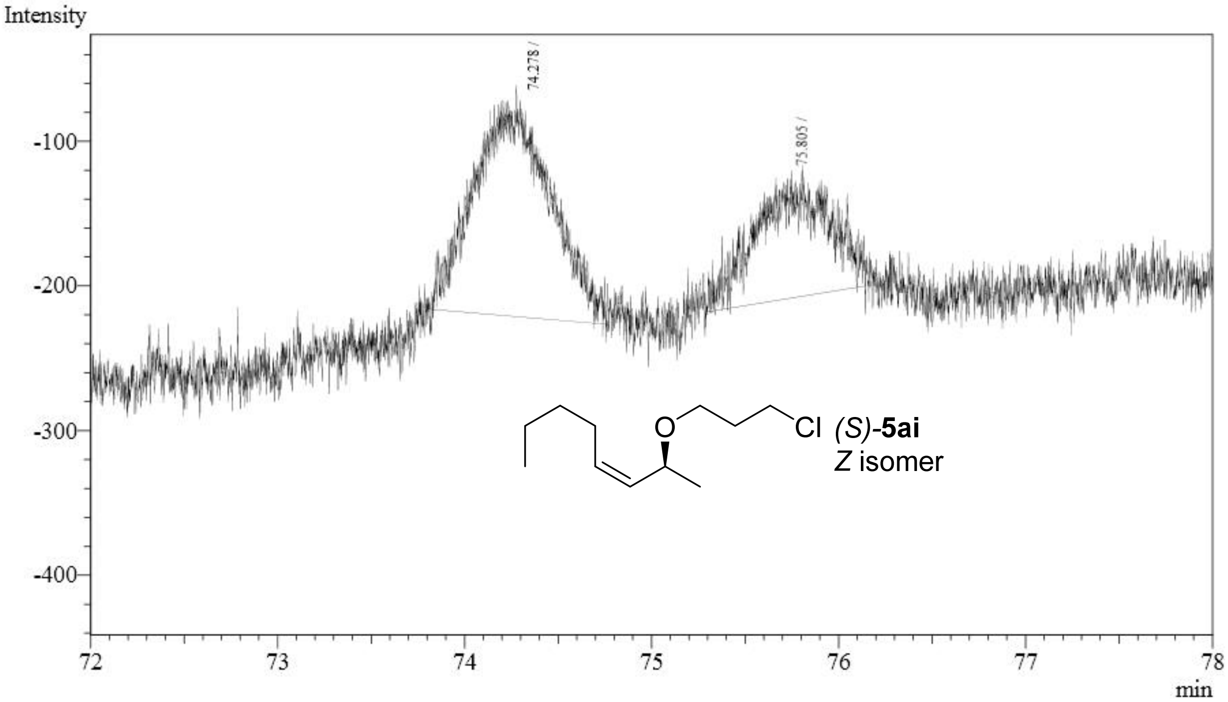

Peak#	Ret.Time	Area	Height	Conc.	Unit	Mark	ID#	Cmpd Name	Area%
1	74.278	4205	152	65.354					65.3539
2	75.805	2229	79	34.646					34.6461
**Total**		6434	231						100.0000

(*S*,*E*)-2-(3-Chloropropoxy)oct-3-ene (*S*)-5ai (Scheme 7, Eq. 3) using unactivated MS

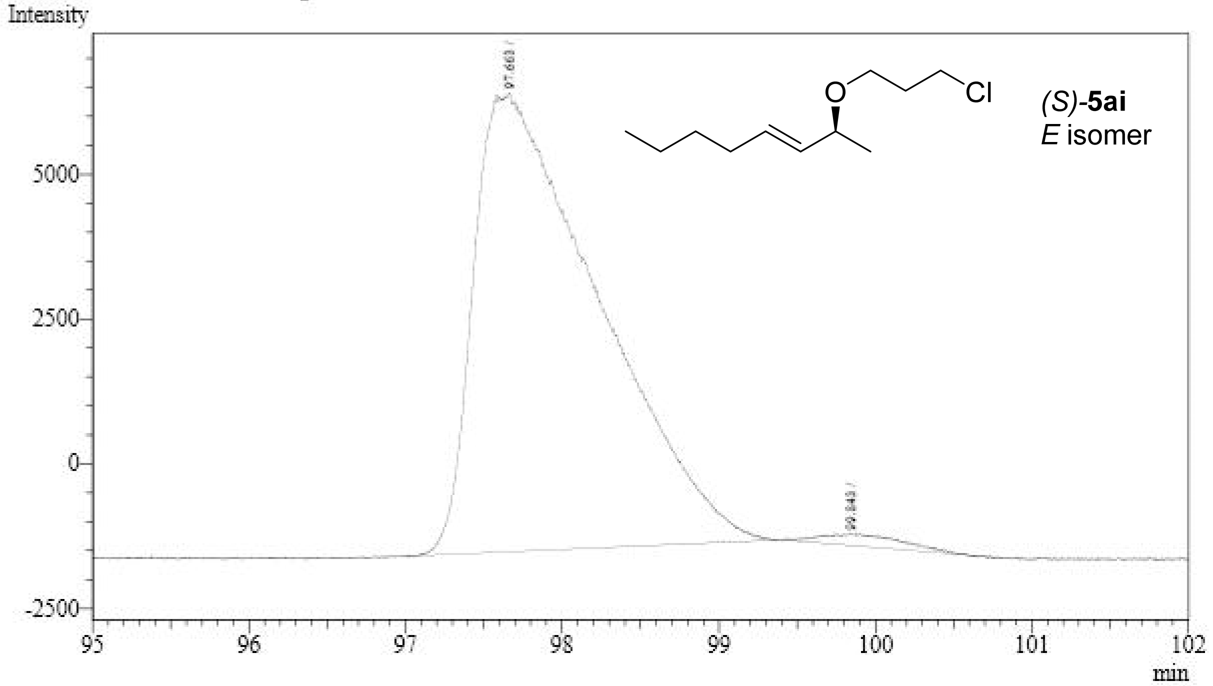

Peak#	Ret.Time	Area	Height	Conc.	Unit	Mark	ID#	Cmpd Name	Area%
1	97.663	437475	7917	98.317					98.3173
2	99.843	7488	196	1.683					1.6827
Total		444963	8113						100.0000

(*S*,*Z*)-2-(3-Chloropropoxy)oct-3-ene (*S*)-5ai (Scheme 7, Eq. 3)

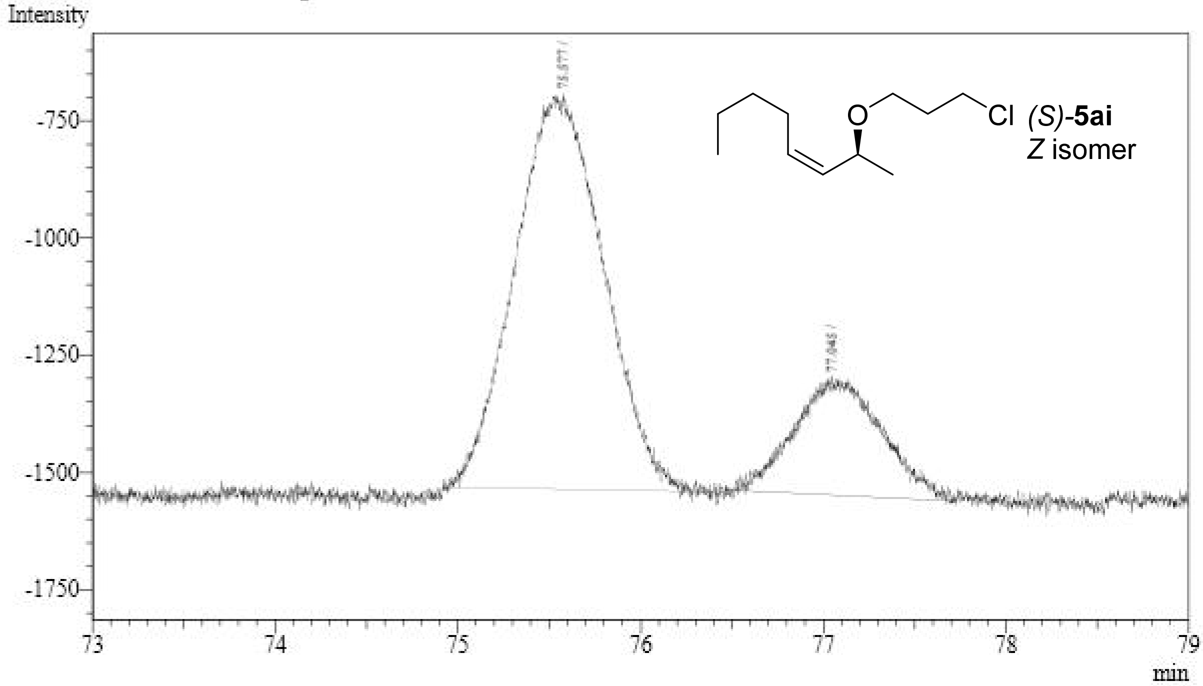

Peak#	Ret.Time	Area	Height	Conc.	Unit	Mark	ID#	Cmpd Name	Area%
1	75.577	28309	836	77.622					77.6225
2	77.045	8161	245	22.378					22.3775
Total		36470	1081						100.0000

(*E*)-2-(3-Chloropropoxy)oct-3-ene *Rac*-5ai (Scheme 7, Eq. 1). No MS used

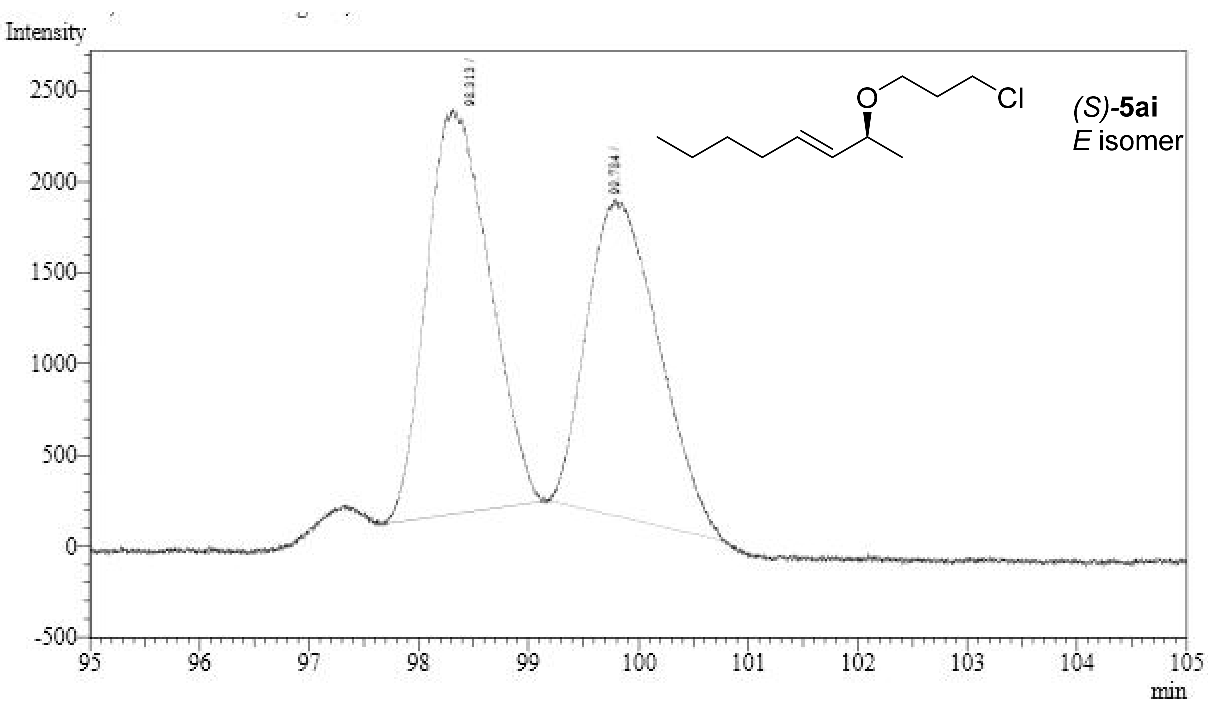

Peak#	Ret.Time	Area	Height	Conc.	Unit	Mark	ID#	Cmpd Name	Area%
1	98.313	89061	2216	53.096					53.0963
2	99.784	78673	1731	46.904					46.9037
**Total**		167734	3947						100.0000

(*Z*)-2-(3-Chloropropoxy)oct-3-ene *Rac*-5ai (Scheme 7, Eq. 1) No MS used

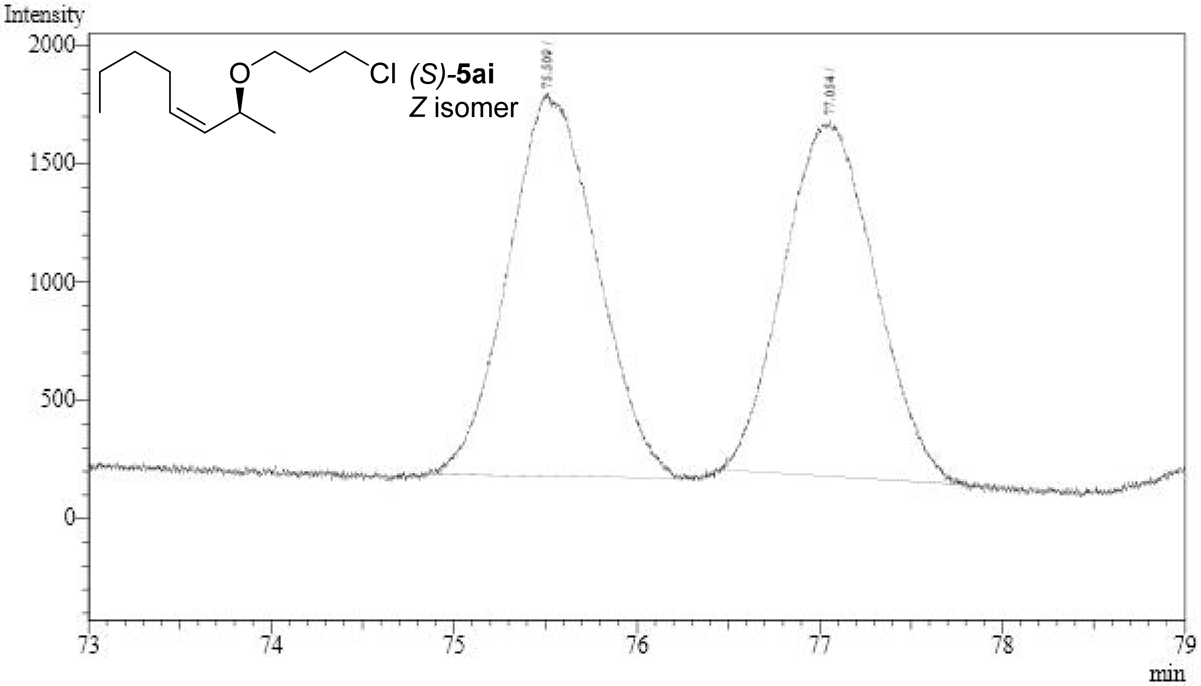

Peak#	Ret.Time	Area	Height	Conc.	Unit	Mark	ID#	Cmpd Name	Area%
1	75.509	54593	1612	51.114					51.1141
2	77.054	52213	1498	48.886					48.8859
Total		106806	3110						100.0000

(*S*,*E*)-(((2-(Hex-5-en-1-yloxy)pent-3-en-1-yl)oxy)methyl)benzene (*S*)-5jj

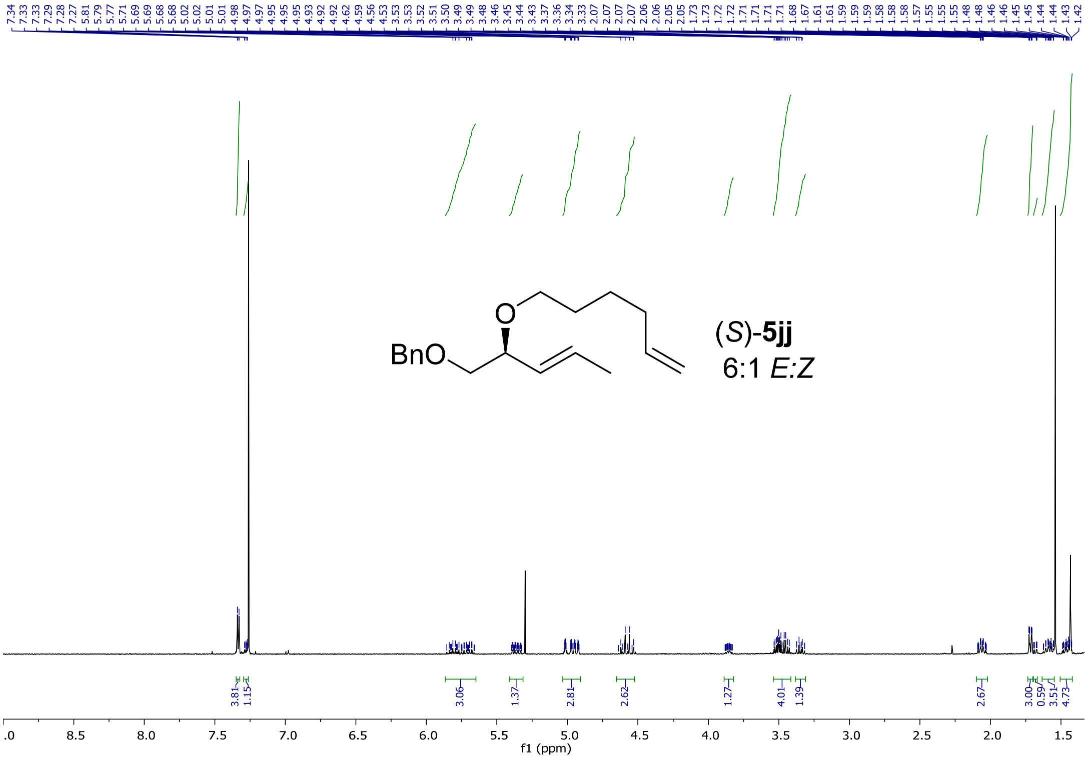

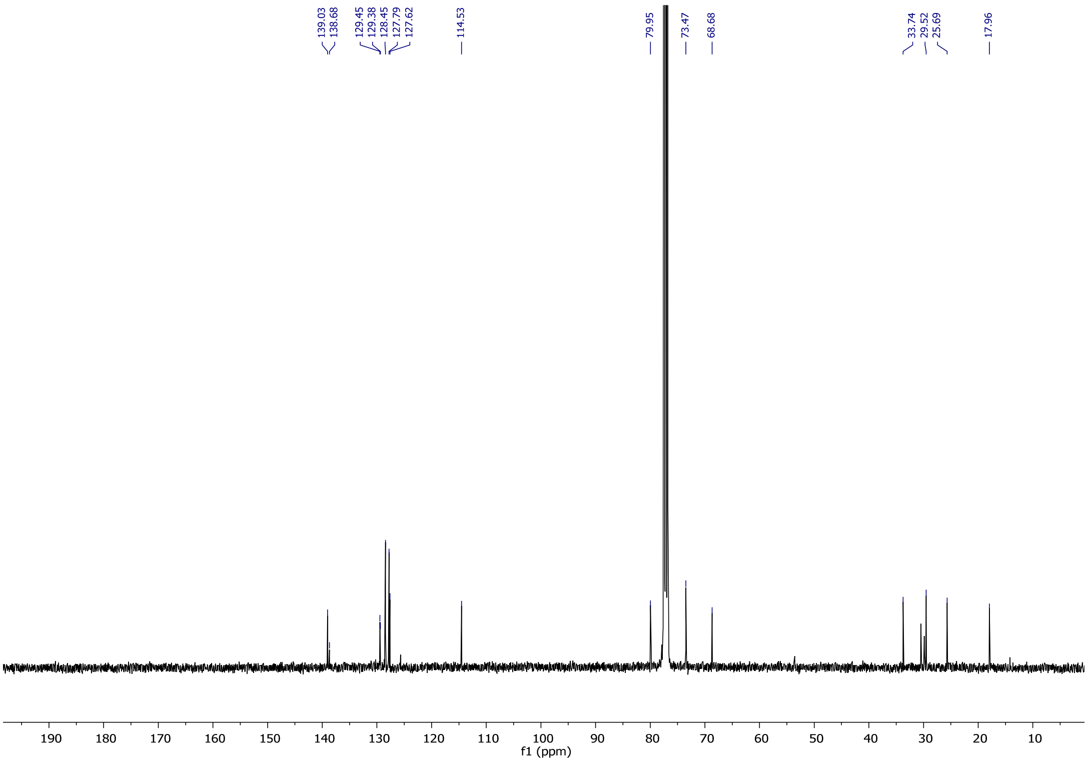

(*E*)-(((2-(Hex-5-en-1-yloxy)pent-3-en-1-yl)oxy)methyl)benzene *Rac*-5jj

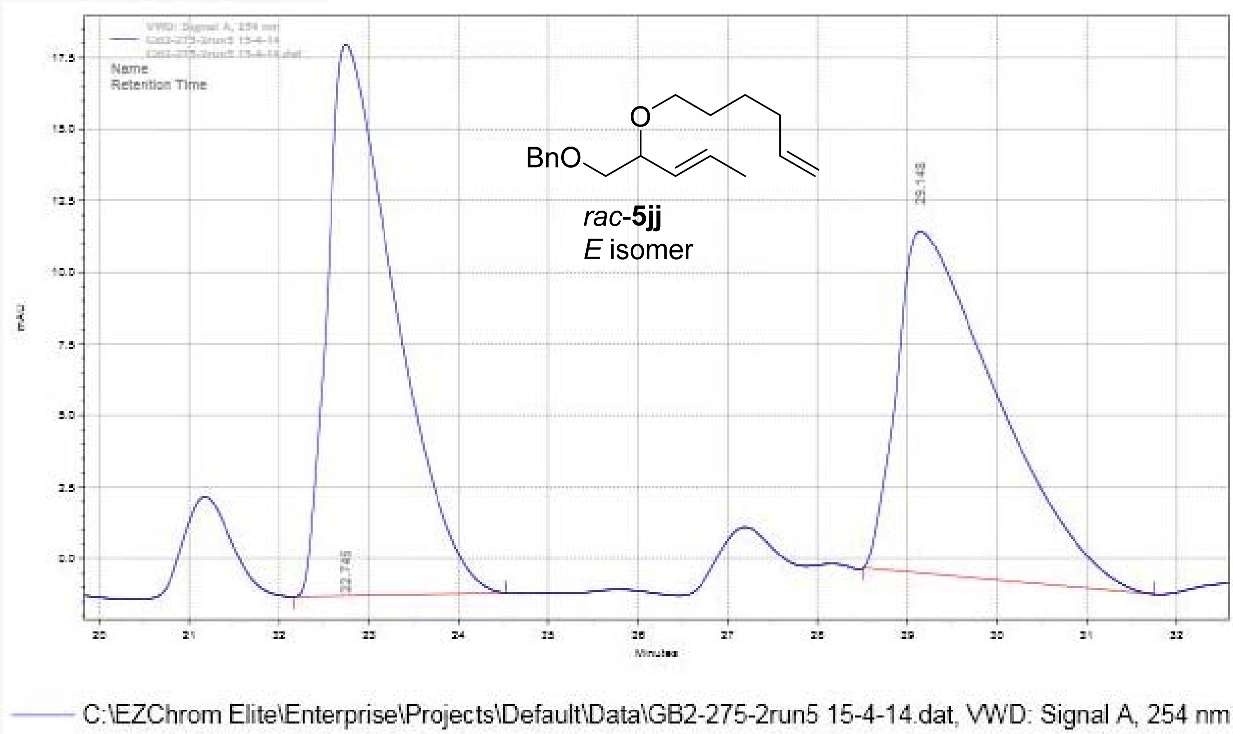

VWD: Signal A,
254 nm Results

Retention Time	Area	Area %	Height	Height %
22.745	16946640	52.05	322993	61.75
29.148	15609501	47.95	200103	38.25

(*Z*)-(((2-(Hex-5-en-1-yloxy)pent-3-en-1-yl)oxy)methyl)benzene *Rac*-5jj

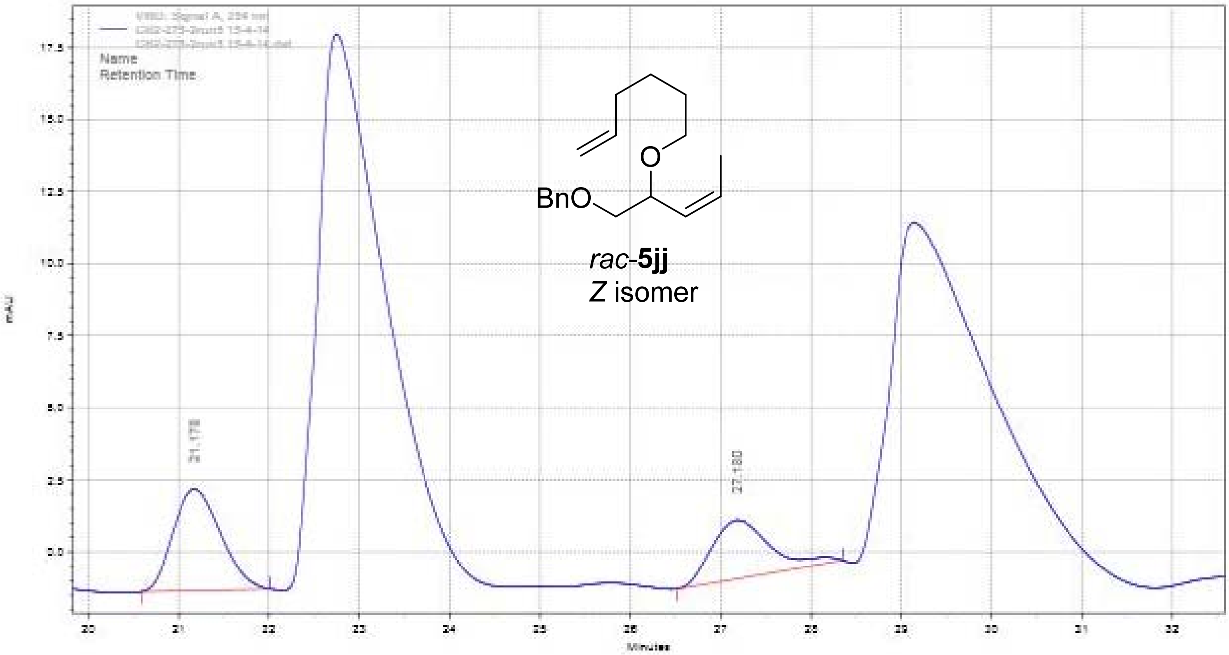

C:\EZChrom Elite\Enterprise\Projects\Default\Data\GB2-275-2run5 15-4-14.dat, VWD: Signal A, 254 nm

VWD: Signal A, 254 nm Results

Retention Time	Area	Area %	Height	Height %
21.178	2144678	58.65	58885	63.39
27.180	1512186	41.35	34007	36.61

(*S*,*E*)-(((2-(Hex-5-en-1-yloxy)pent-3-en-1-yl)oxy)methyl)benzene (*S*)-5jj

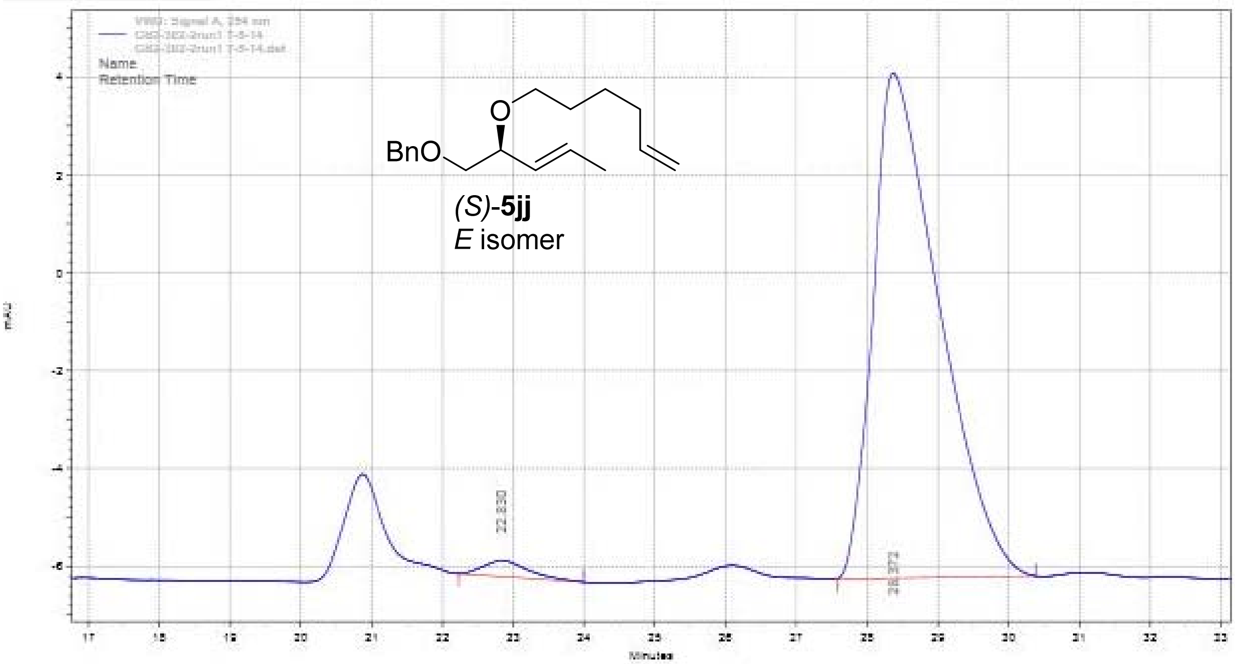

C:\EZChrom Elite\Enterprise\Projects\Default\Data\GB2-302-2run1 7-5-14.dat, VWD: Signal A, 254 nm

VWD: Signal A,
254 nm Results

Retention Time	Area	Area %	Height	Height %
22.830	240056	2.09	5486	3.07
28.372	11227000	97.91	173035	96.93

(*S, Z*)-(((2-(Hex-5-en-1-yloxy)pent-3-en-1-yl)oxy)methyl)benzene (*S*)-5jj

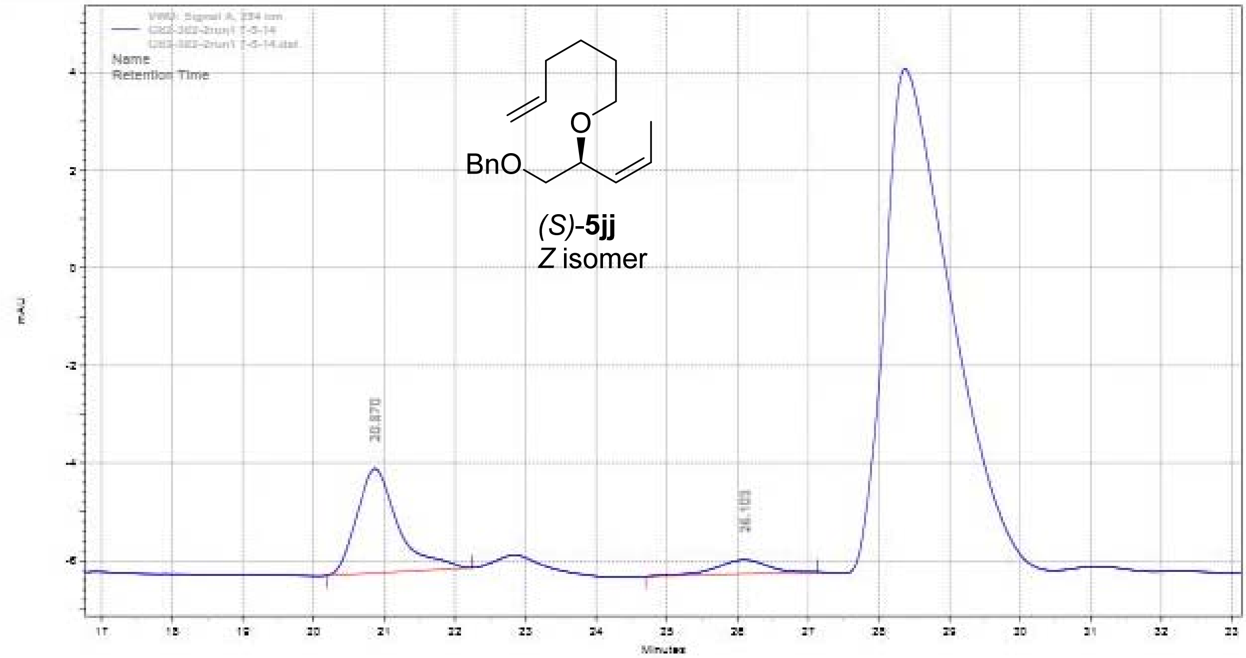

C:\EZChrom Elite\Enterprise\Projects\Default\Data\GB2-302-2run1 7-5-14.dat, VWD: Signal A, 254 nm

VWD: Signal A, 254 nm Results

Retention Time	Area	Area %	Height	Height %
20.870	1465085	85.70	35924	88.05
26.103	244498	14.30	4877	11.95

(*S*,*E*)-2-Methyl-4-(oct-3-en-2-yloxy)butan-2-ol (*S*)-5ak

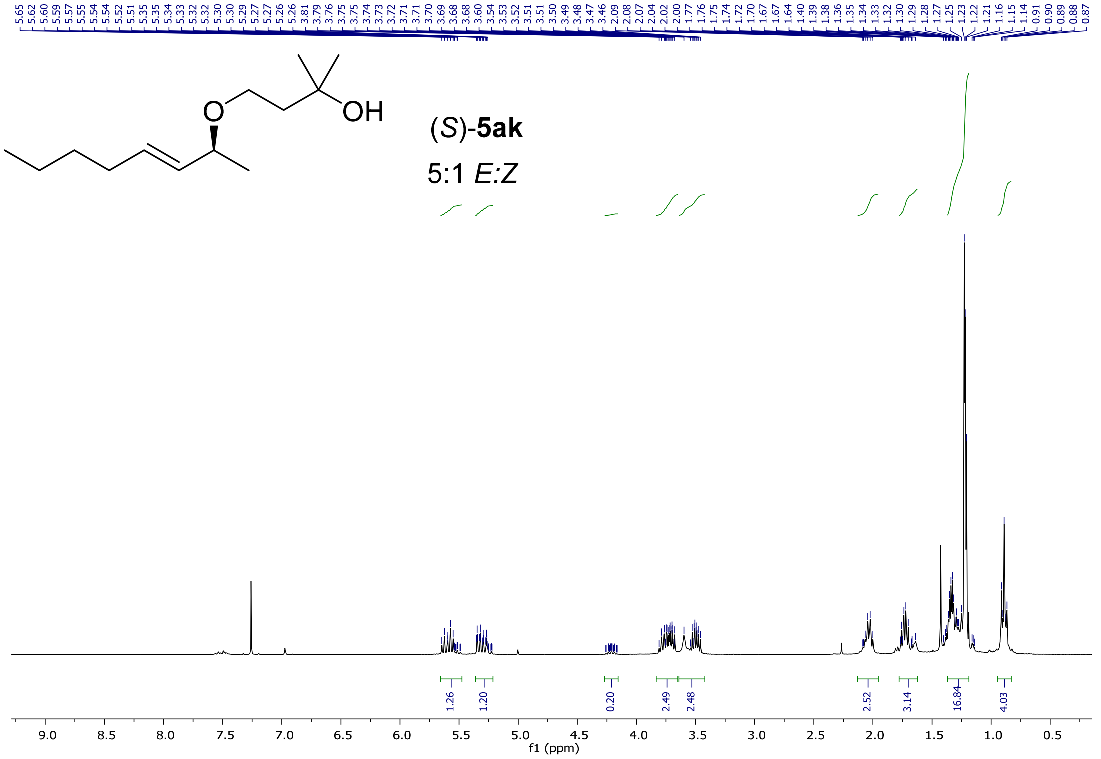

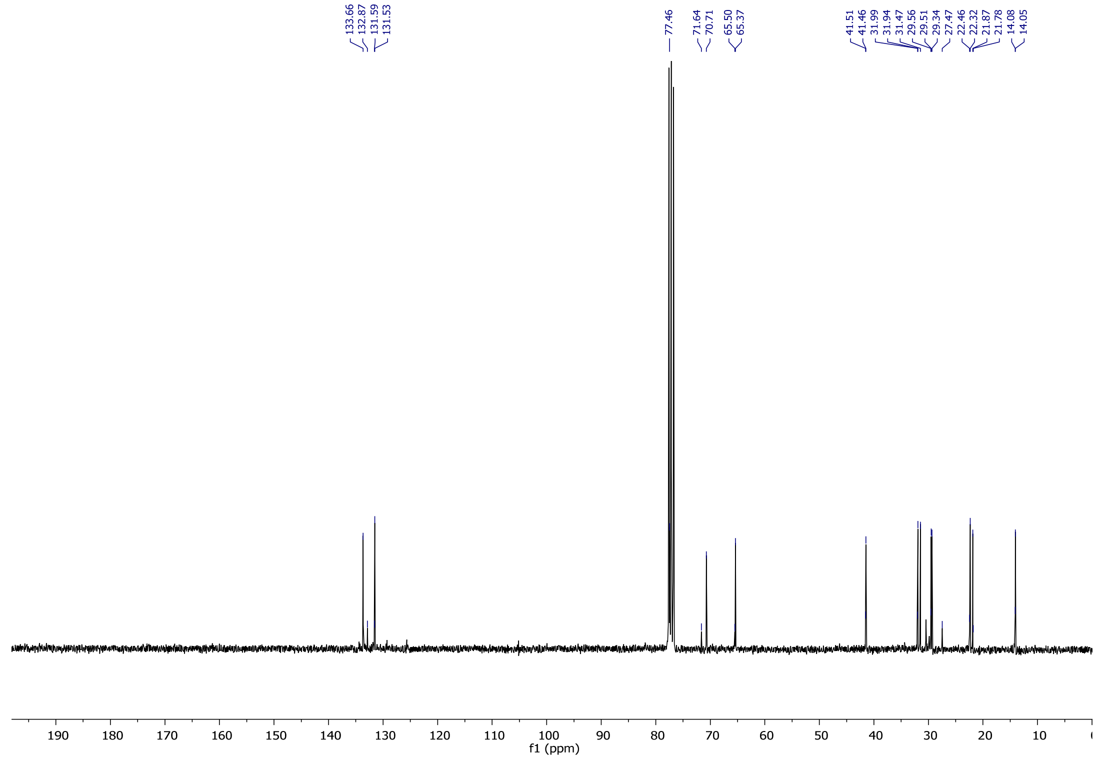

(*E*)-2-Methyl-4-(oct-3-en-2-yloxy)butan-2-ol *Rac*-5ak

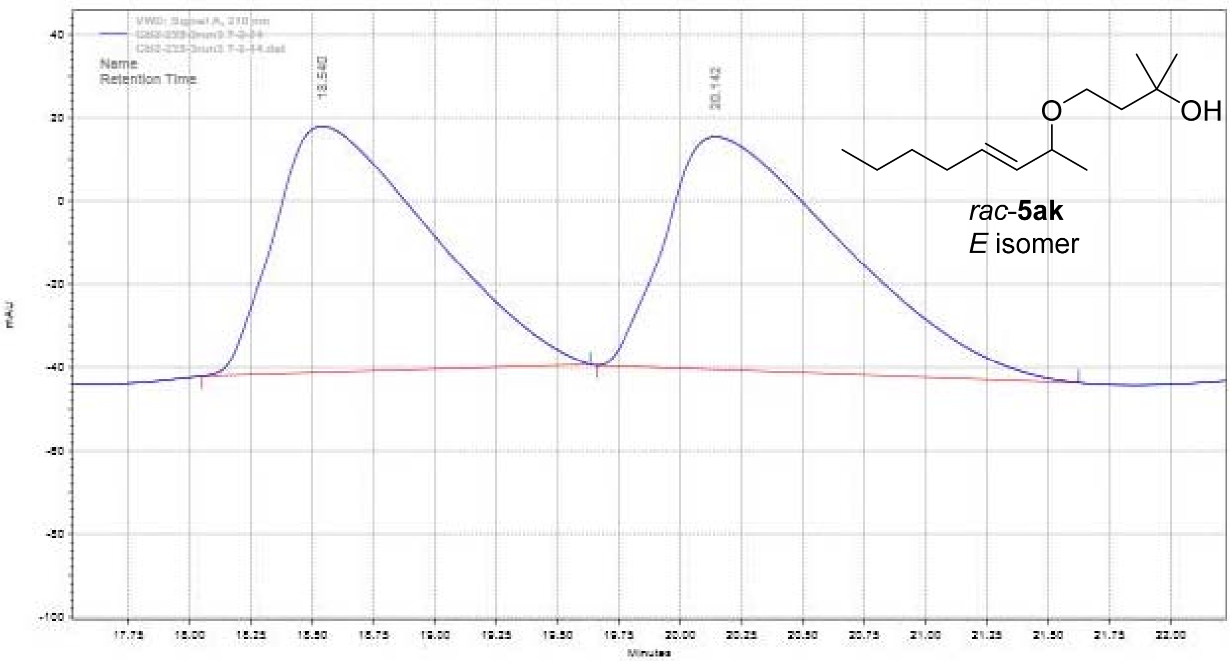

C:\EZChrom Elite\Enterprise\Projects\Default\Data\GB2-235-3run3 7-2-14.dat, VWD: Signal A, 210 nm

VWD: Signal A, 210 nm Results

Retention Time	Area	Area %	Height	Height %
18.540	43763123	47.90	993001	51.40
20.142	47593335	52.10	938958	48.60

(*Z*)-2-Methyl-4-(oct-3-en-2-yloxy)butan-2-ol *Rac*-5ak

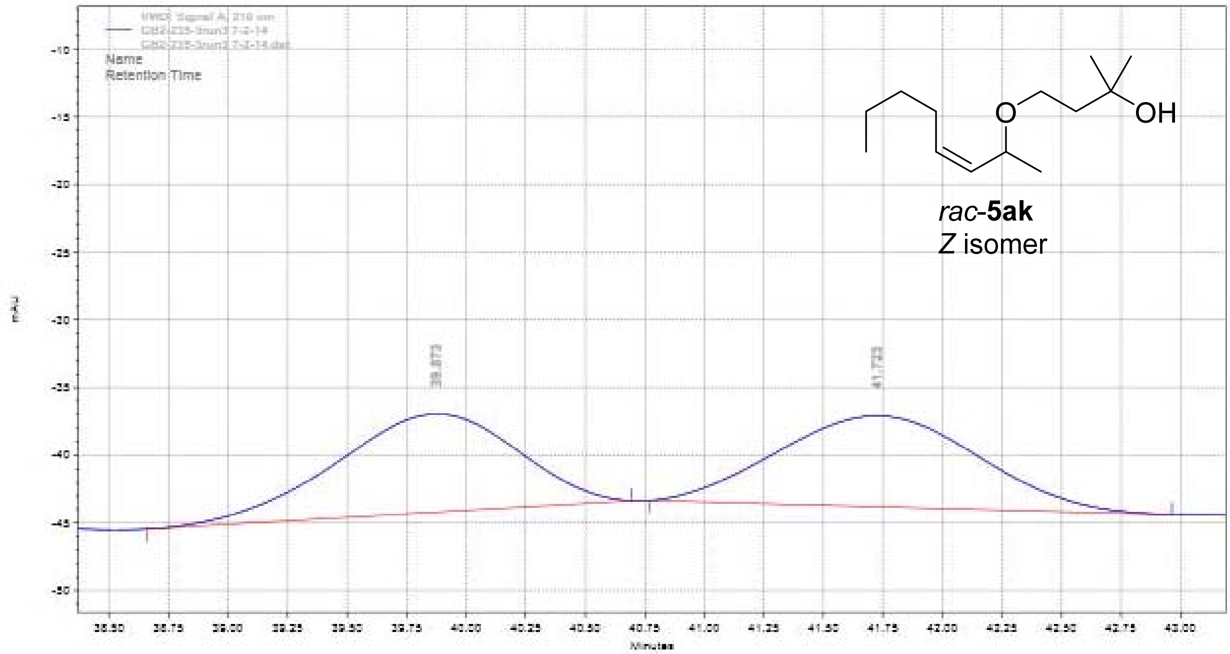

C:\EZChrom Elite\Enterprise\Projects\Default\Data\GB2-235-3run3 7-2-14.dat, VWD: Signal A, 210 nm

VWD: Signal A,
210 nm Results

Retention Time	Area	Area %	Height	Height %
39.873	6451071	49.48	121819	52.00
41.723	6586650	50.52	112443	48.00

(*S*,*E*)-2-Methyl-4-(oct-3-en-2-yloxy)butan-2-ol (*S*)-5ak

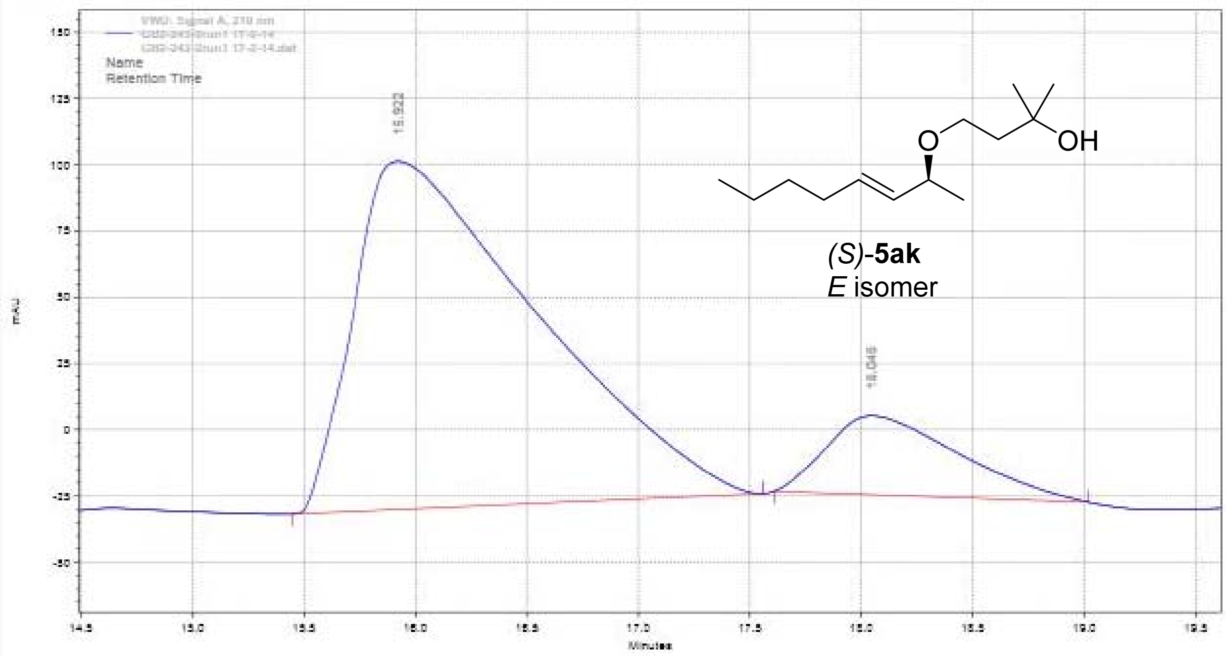

C:\EZChrom Elite\Enterprise\Projects\Default\Data\GB2-243-2run1 17-2-14.dat, VWD: Signal A, 210 nm

VWD: Signal A,
210 nm Results

Retention Time	Area	Area %	Height	Height %
15.922	125690998	85.92	2203848	81.54
18.045	20589813	14.08	498863	18.46

(*S*,*Z*)-2-Methyl-4-(oct-3-en-2-yloxy)butan-2-ol (*S*)-5ak

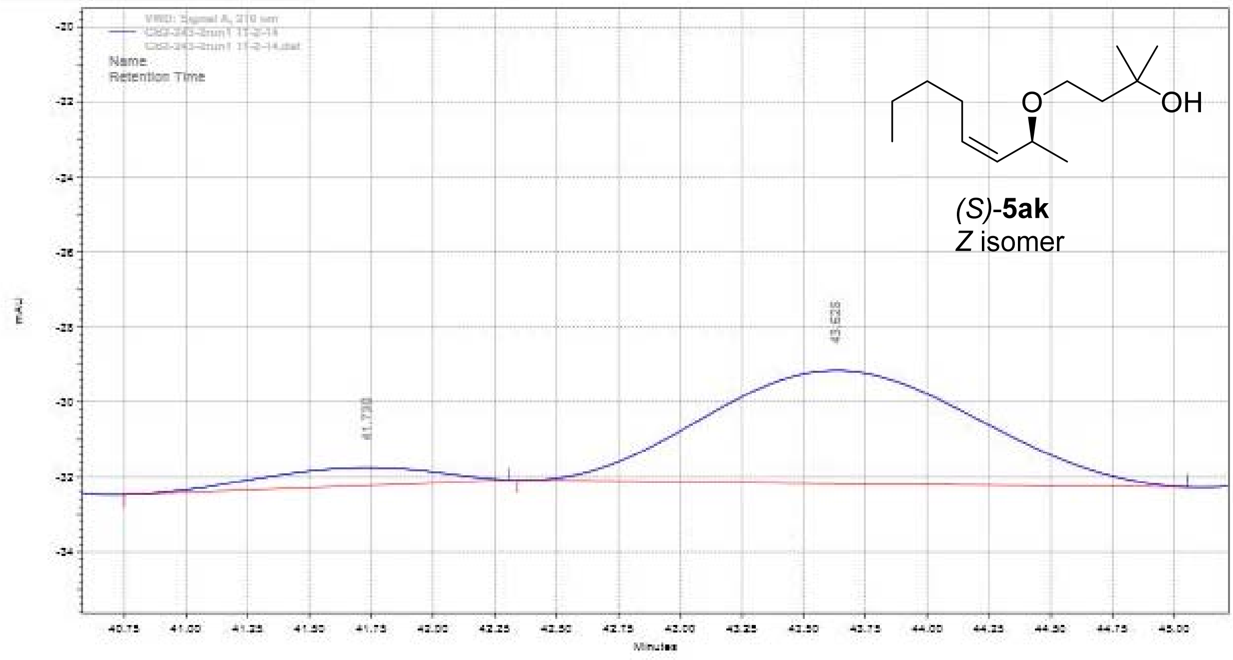

C:\EZChrom Elite\Enterprise\Projects\Default\Data\GB2-243-2run1 17-2-14.dat, VWD: Signal A, 210 nm

VWD: Signal A, 210 nm Results

Retention Time	Area	Area %	Height	Height %
41.730	392989	9.32	7963	13.63
43.628	3823799	90.68	50476	86.37

2,2-Dimethyl-4-(((*S*, *E*)-oct-3-en-2-yloxy)methyl)-1,3-dioxolane (*S*)-5al

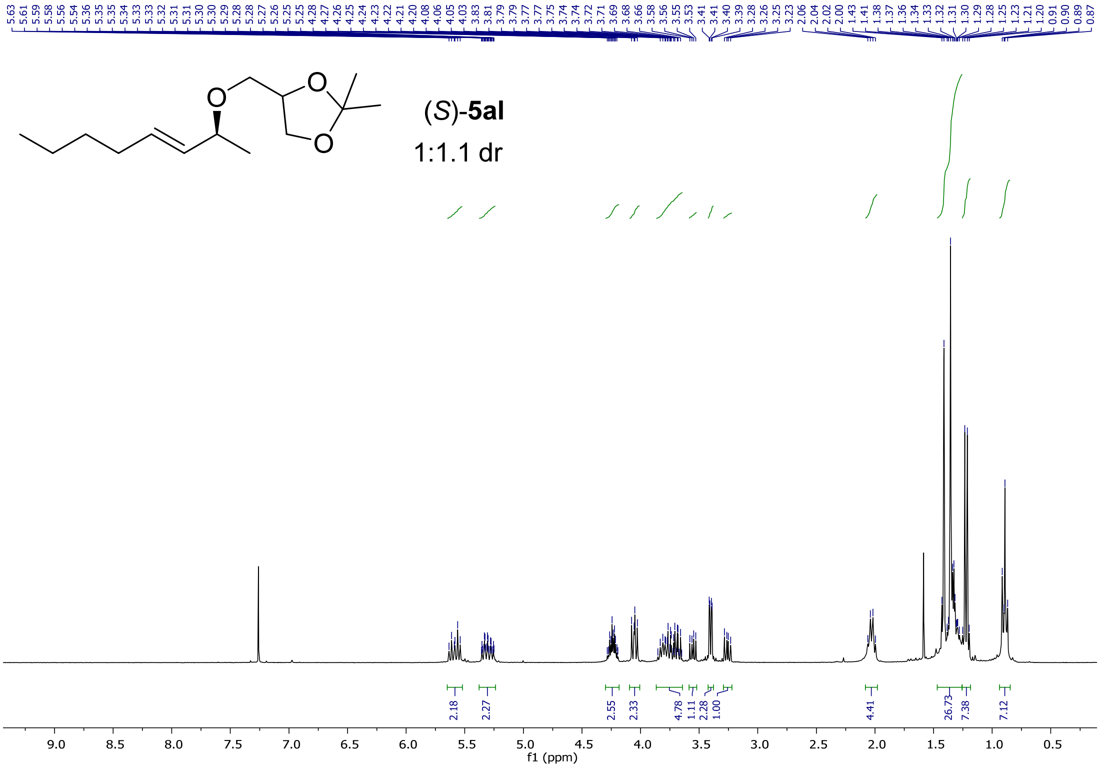

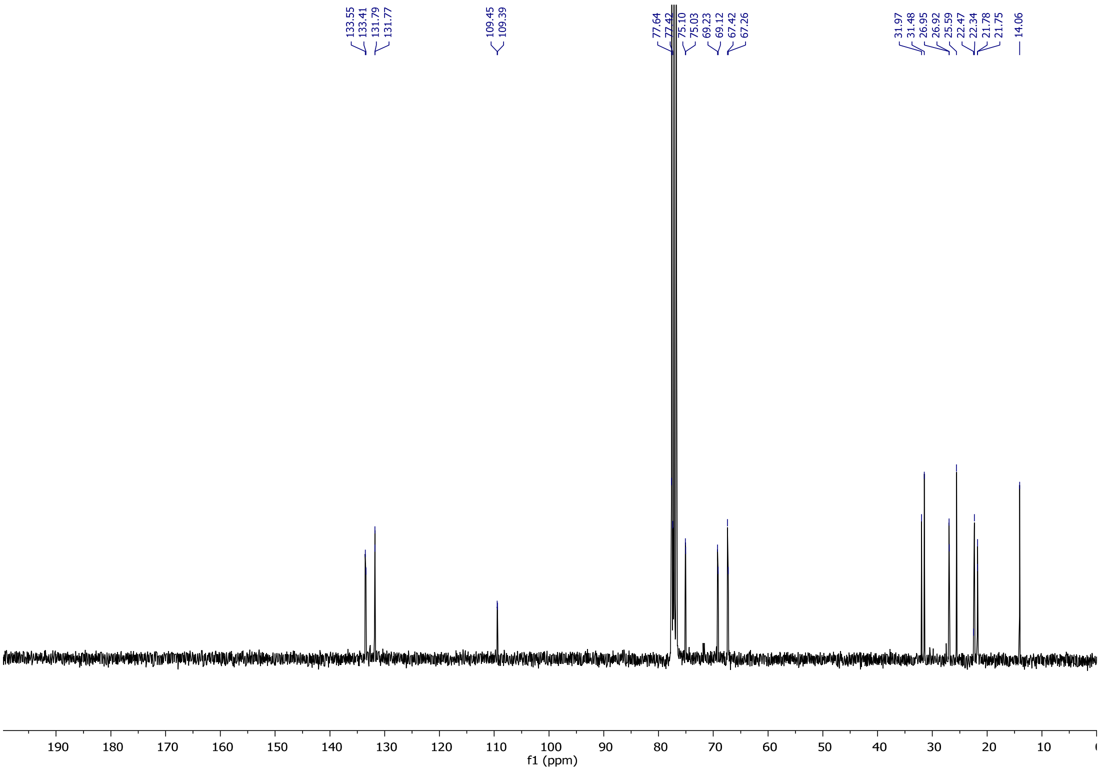

***Rac*-2,2-Dimethyl-4-(((*E*)-oct-3-en-2-yloxy)methyl)-1,3-dioxolane *rac*-5al: first diastereomer**

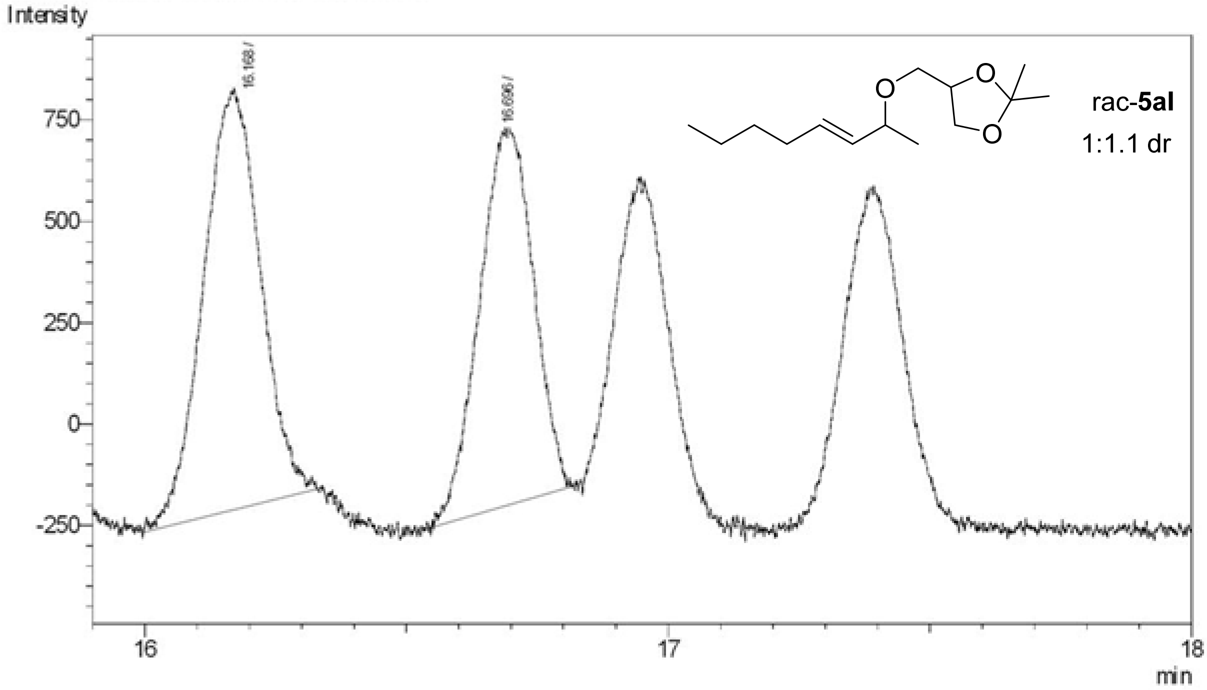

Peak#	Ret.Time	Area	Height	Conc.	Unit	Mark	ID#	Cmpd Name	Area%
1	16.168	7886	1032	54.018					54.0175
2	16.696	6713	924	45.982					45.9825
Total		14599	1956						100.0000

***Rac*-2,2-Dimethyl-4-(((*E*)-oct-3-en-2-yloxy)methyl)-1,3-dioxolane *rac*-5al: second diastereomer**

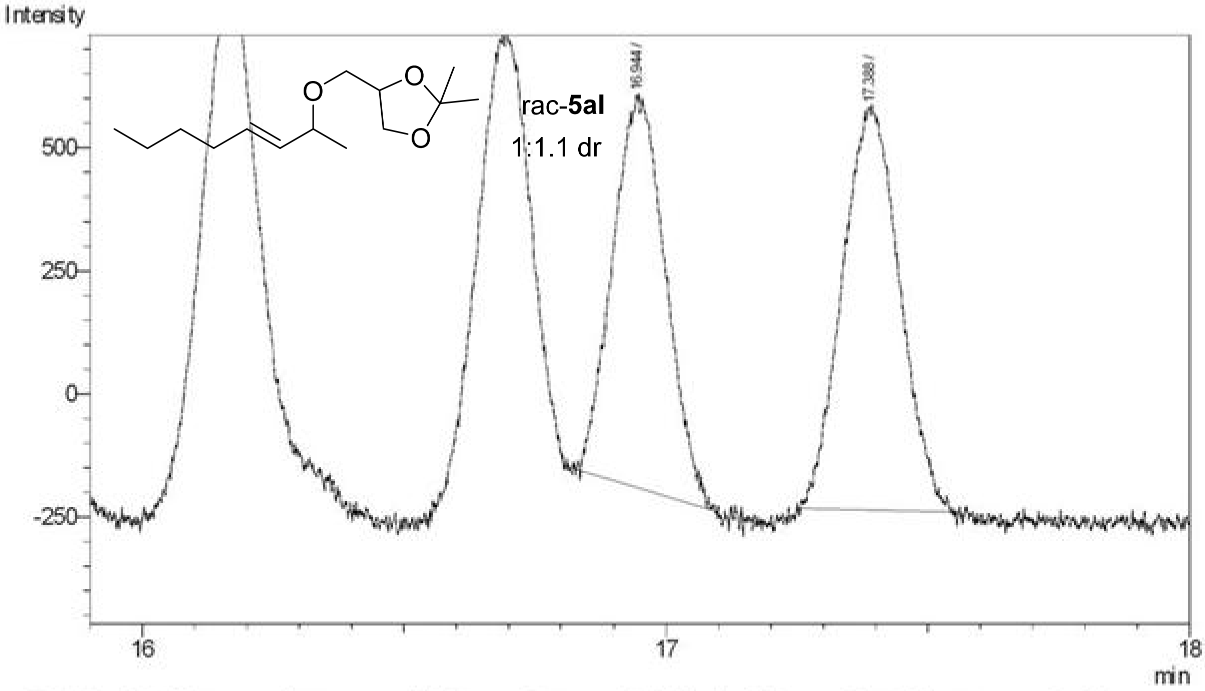

Peak#	Ret.Time	Area	Height	Conc.	Unit	Mark	ID#	Cmpd Name	Area%
1	16.944	5608	792	47.249					47.2492
2	17.388	6261	815	52.751					52.7508
Total		11869	1607						100.0000

2,2-Dimethyl-4-(((*S*, *E*)-oct-3-en-2-yloxy)methyl)-1,3-dioxolane (*S*)-5al: first diastereomer

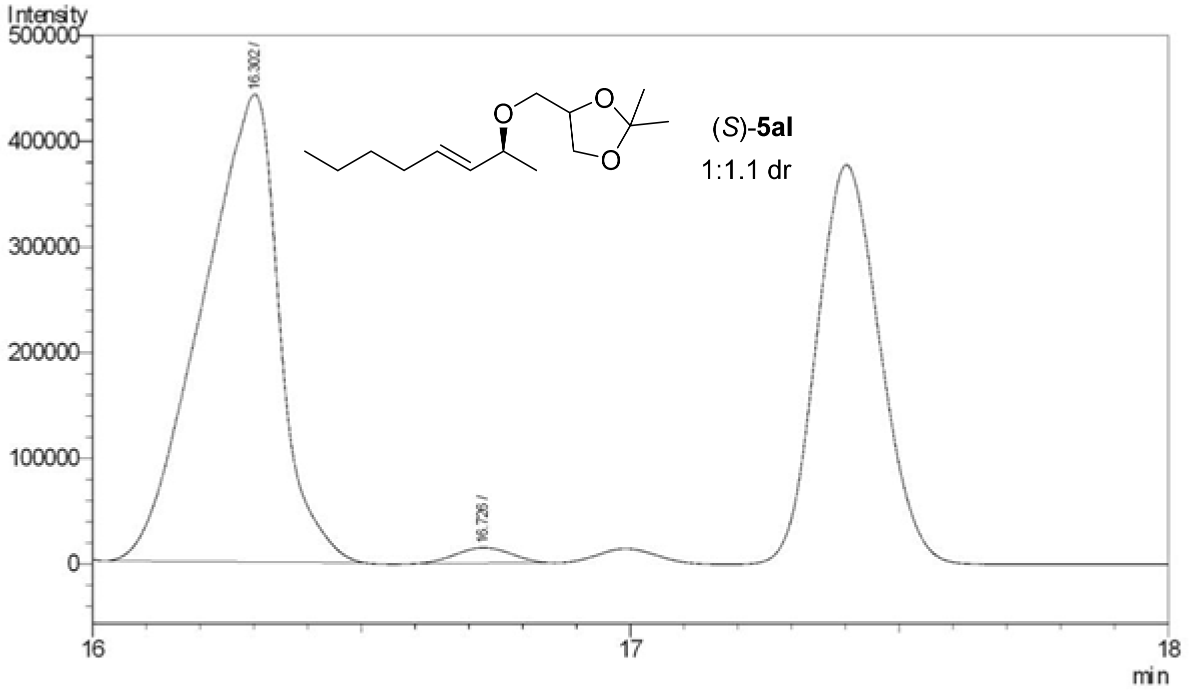

Peak#	Ret.Time	Area	Height	Conc.	Unit	Mark	ID#	Cmpd Name	Area%
1	16.302	4424448	441692	97.695					97.6955
2	16.726	104367	14646	2.305					2.3045
Total		4528815	456338						100.0000

2,2-Dimethyl-4-(((*S*,*E*)-oct-3-en-2-yloxy)methyl)-1,3-dioxolane (*S*)-5al: second diastereomer

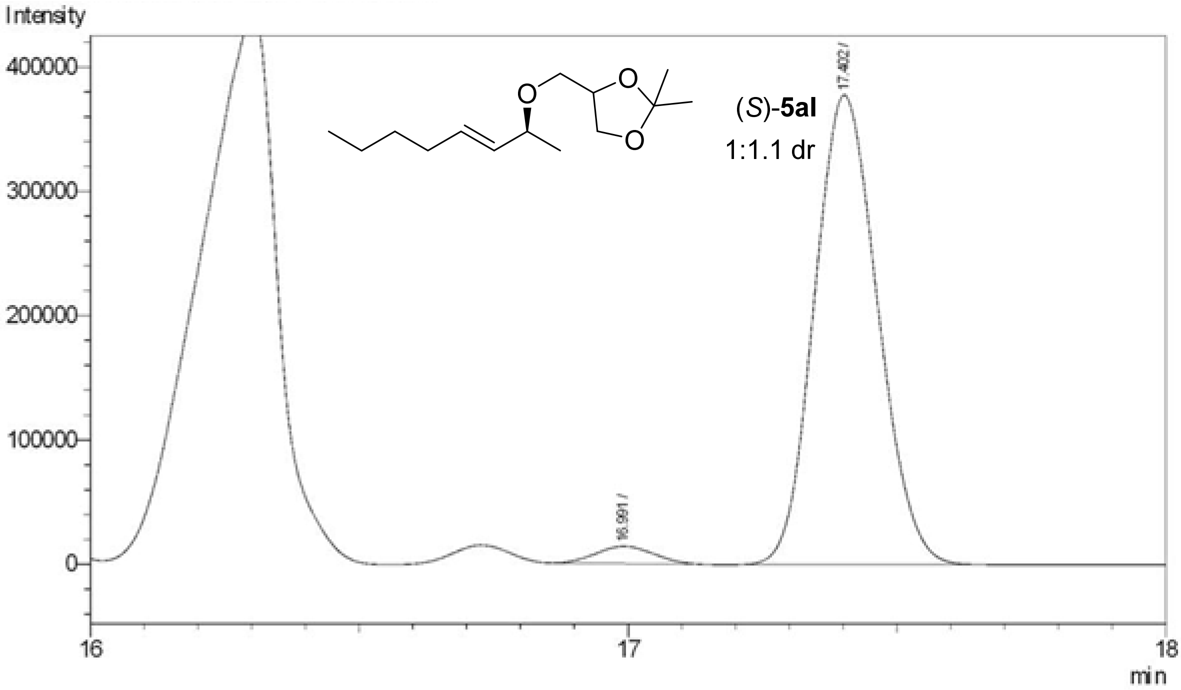

Peak#	Ret.Time	Area	Height	Conc.	Unit	Mark	ID#	Cmpd Name	Area%
1	16.991	100280	13717	3.147					3.1466
2	17.402	3086667	377320	96.853					96.8534
Total		3186947	391037						100.0000

2-(2-((*S*, *E*)-Oct-3-en-2-yloxy)ethoxy)tetrahydro-2H-pyran (*S*)-5am

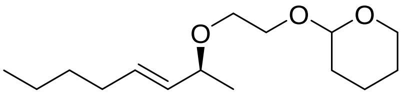

(*S*)-**5am**
10:1 *E*:*Z*

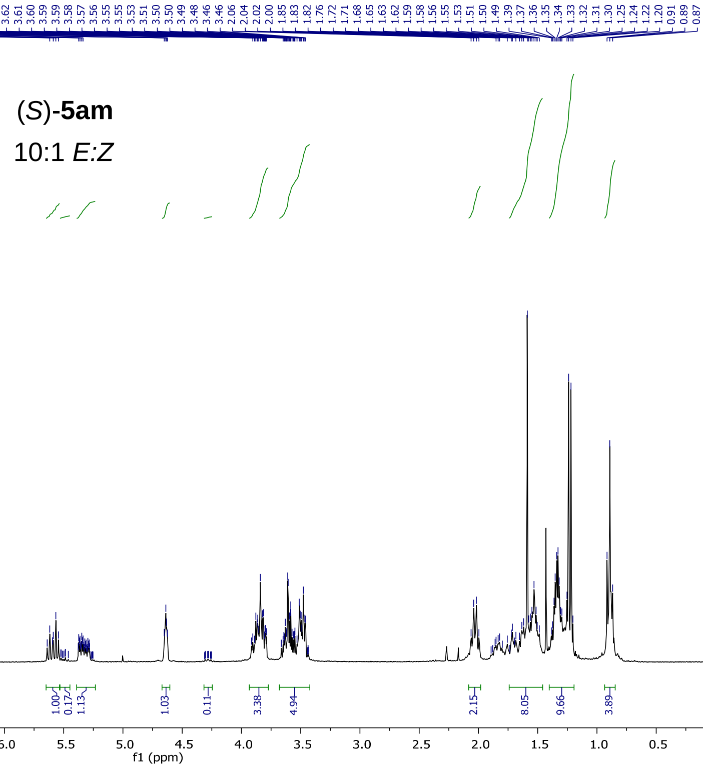

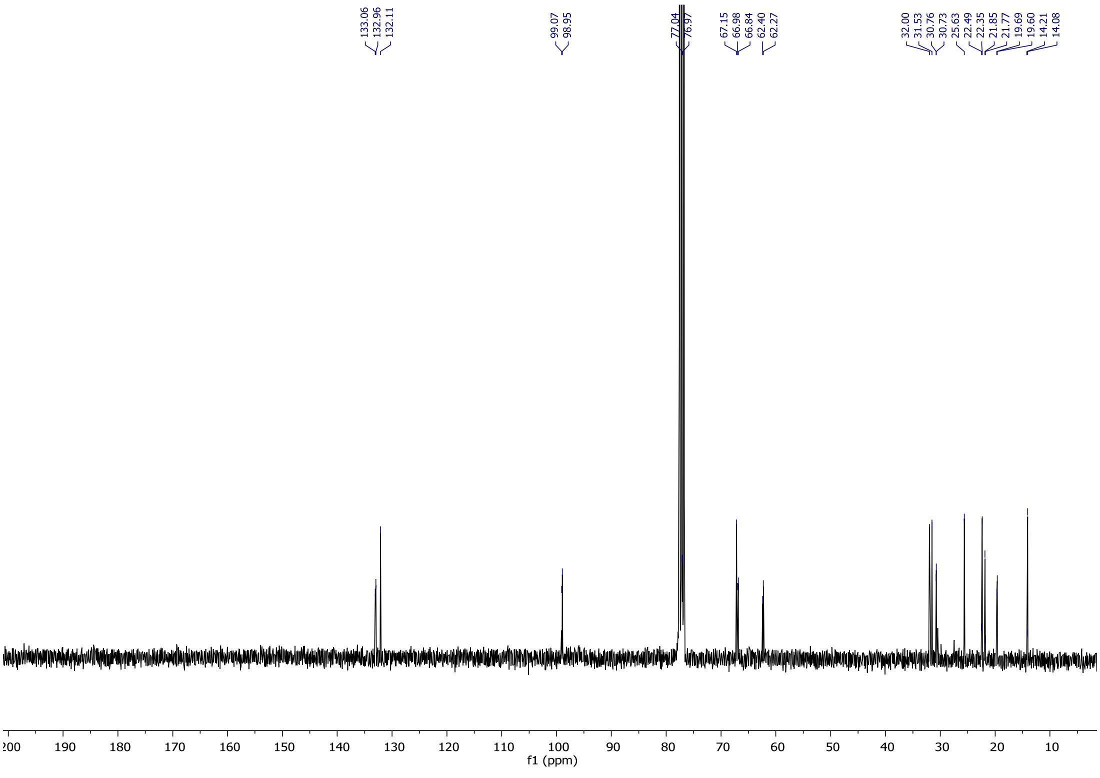

***Rac*-2-(2-((*E*)-Oct-3-en-2-yloxy)ethoxy)tetrahydro-2H-pyran *rac*-5am**

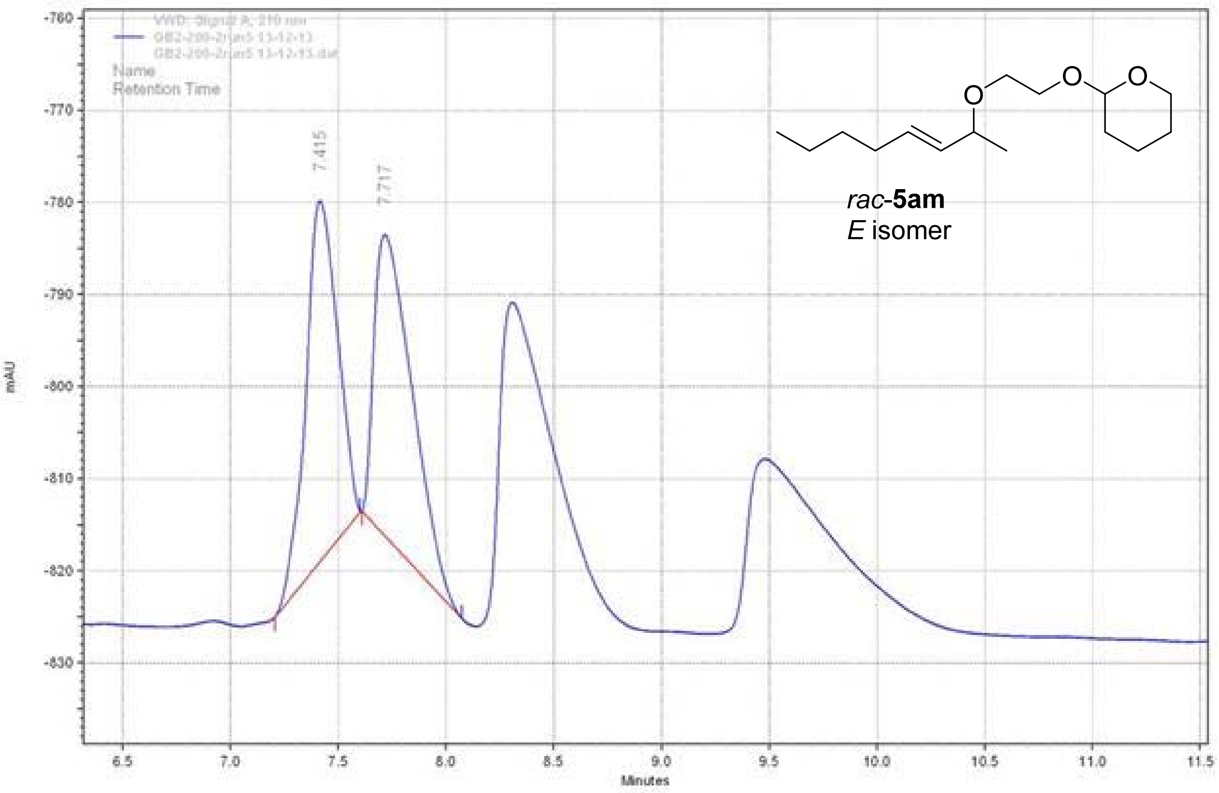

C:\EZChrom Elite\Enterprise\Projects\Default\Data\GB2-200-2run5 13-12-13.dat, VWD: Signal A, 210 nm

VWD: Signal A, 210 nm Results

Retention Time	Area	Area %	Height	Height %
7.415	6719971	49.55	657853	54.53
7.717	6841465	50.45	548535	45.47

***Rac*-2-(2-((*Z*)-Oct-3-en-2-yloxy)ethoxy)tetrahydro-2H-pyran *rac*-5am**

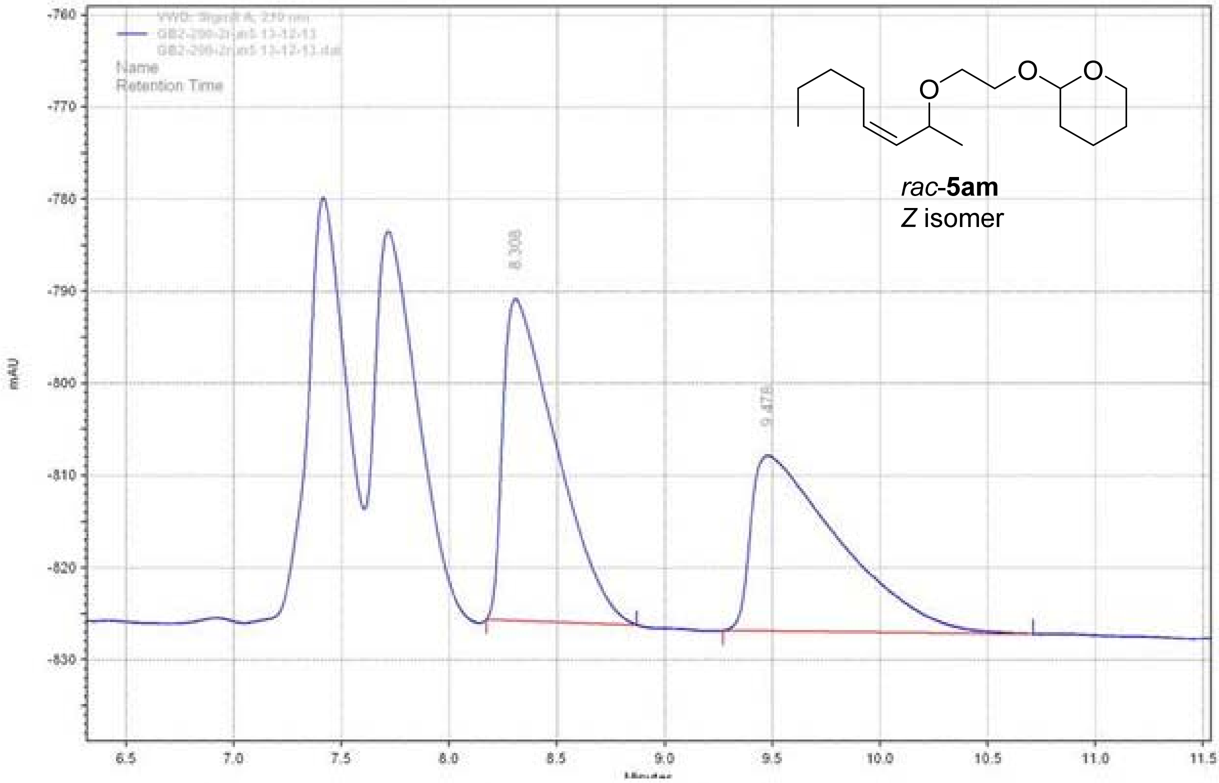

C:\EZChrom Elite\Enterprise\Projects\Default\Data\GB2-200-2run5 13-12-13.dat, VWD: Signal A, 210 nm

VWD: Signal A, 210 nm Results

Retention Time	Area	Area %	Height	Height %
8.308	10137320	52.79	585410	64.73
9.478	9067097	47.21	318921	35.27

2-(2-((*S*, *E*)-Oct-3-en-2-yloxy)ethoxy)tetrahydro-2H-pyran (*S*)-5am

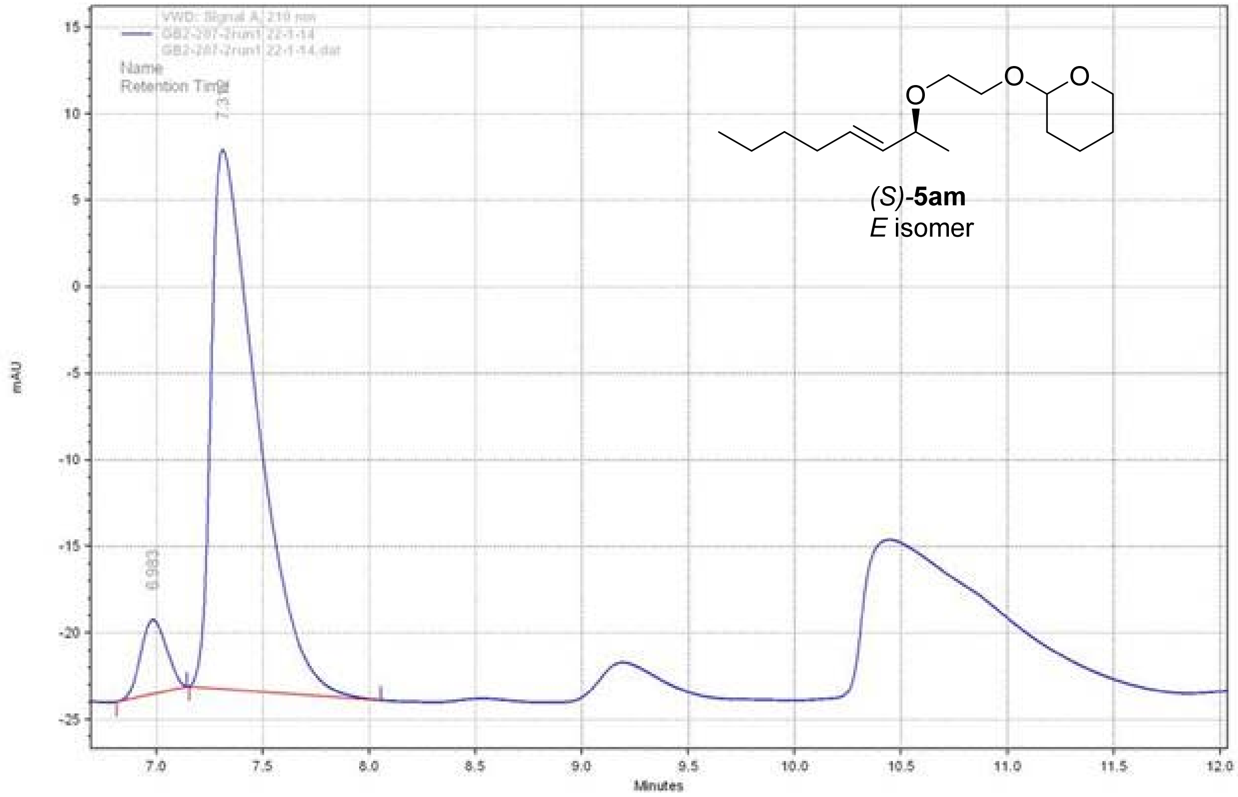

C:\EZChrom Elite\Enterprise\Projects\Default\Data\GB2-207-2run1 22-1-14.dat, VWD: Signal A, 210 nm

VWD: Signal A, 210 nm Results

Retention Time	Area	Area %	Height	Height %
6.983	604641	7.13	71756	12.07
7.312	7873828	92.87	522635	87.93

2-(2-((*S*, *Z*)-Oct-3-en-2-yloxy)ethoxy)tetrahydro-2H-pyran (*S*)-5am

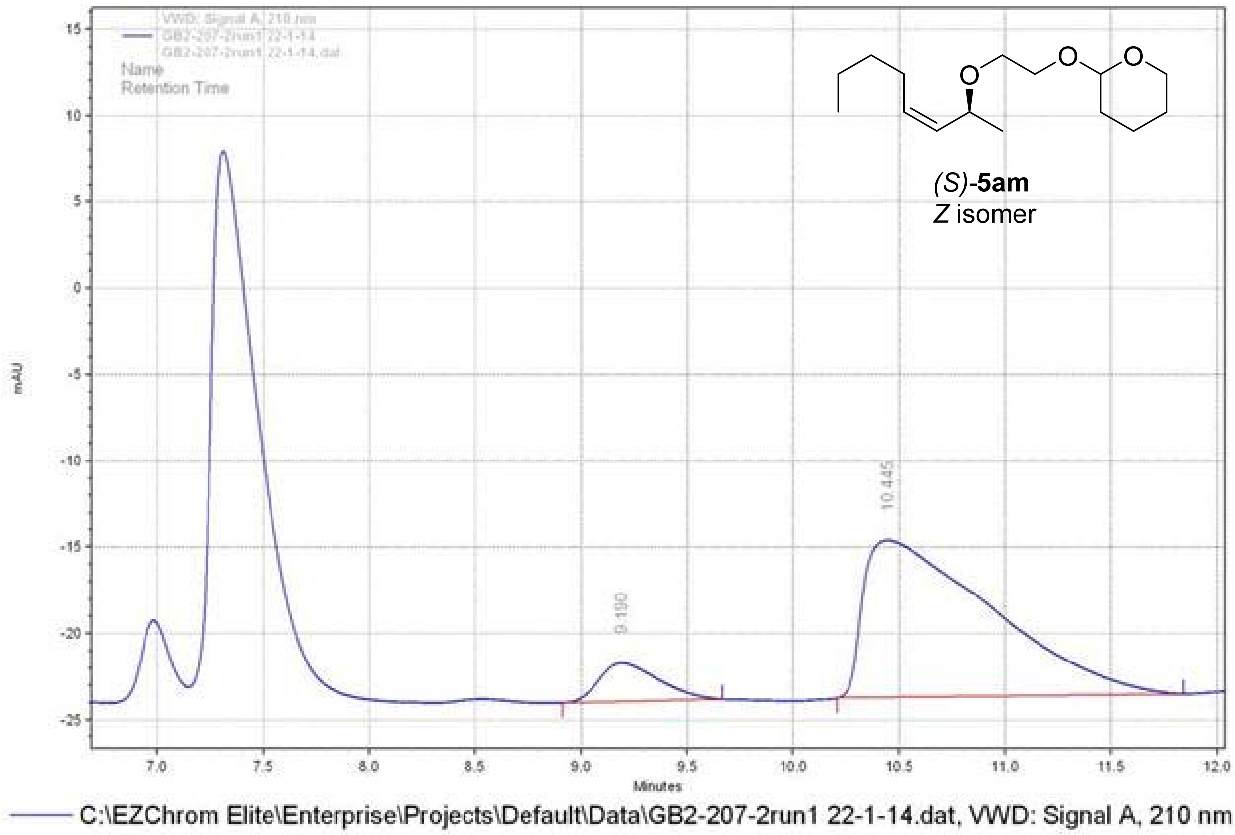

VWD: Signal A, 210 nm Results

Retention Time	Area	Area %	Height	Height %
9.190	723087	10.13	37213	19.65
10.445	6412384	89.87	152134	80.35

(*S*,*E*)-(Oct-3-en-2-yloxy)cyclohexane (*S*)-5gn

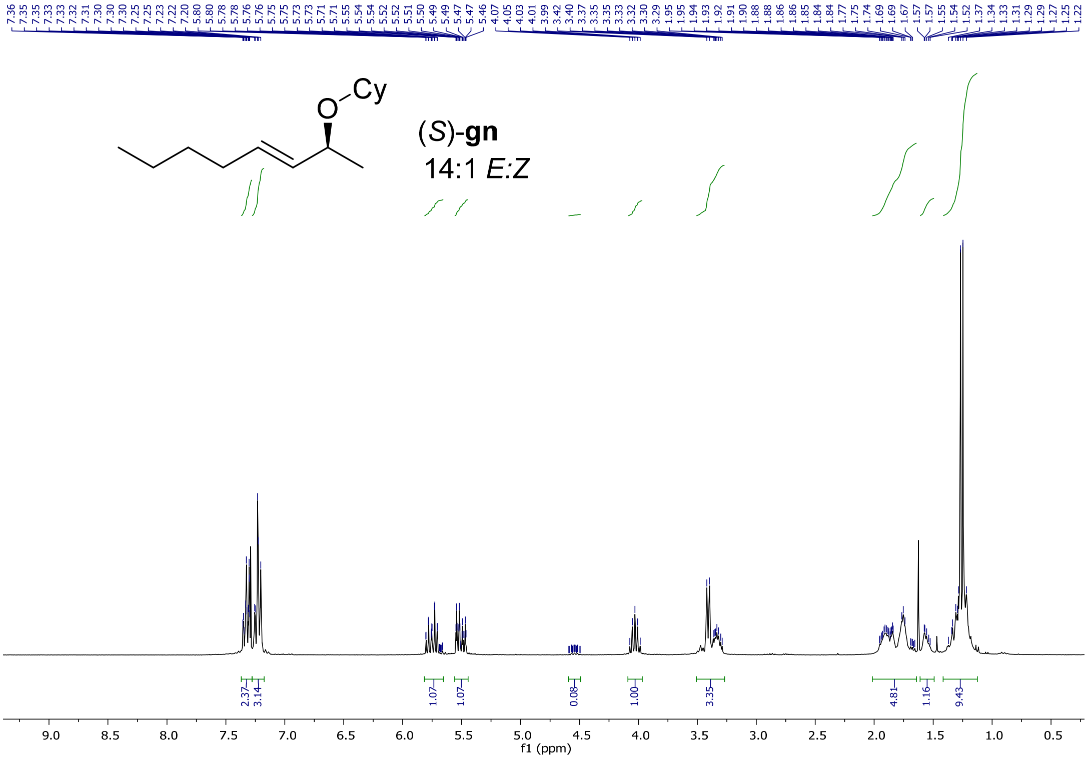

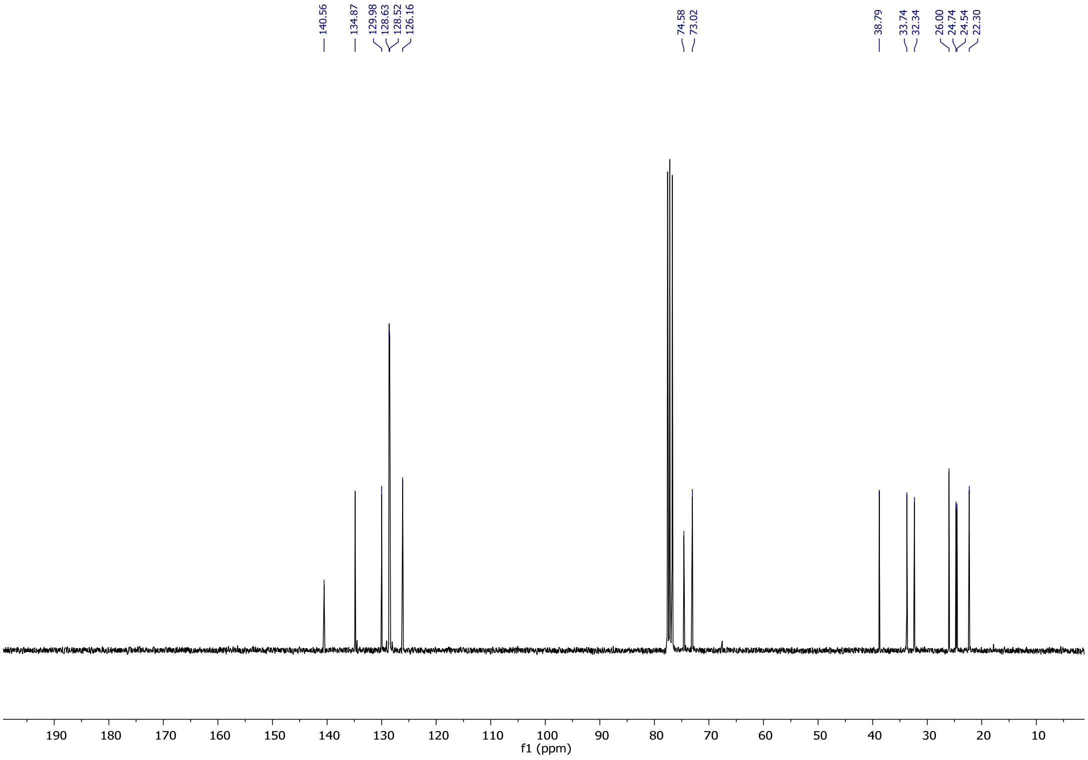

(*E*)-(Oct-3-en-2-yloxy)cyclohexane *Rac*-5gn

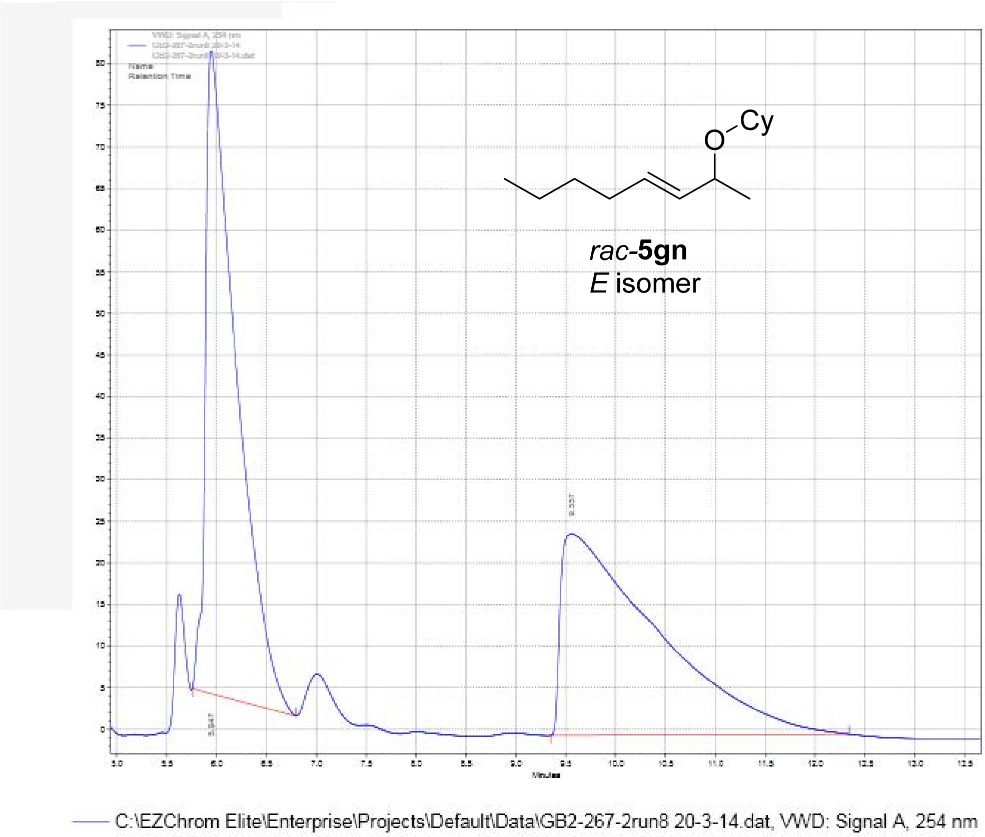

VWD: Signal A,
254 nm Results

Retention Time	Area	Area %	Height	Height %
5.947	27949975	50.68	1296038	76.15
9.557	27204146	49.32	405943	23.85

(*Z*)-(Oct-3-en-2-yloxy)cyclohexane *Rac*-5gn

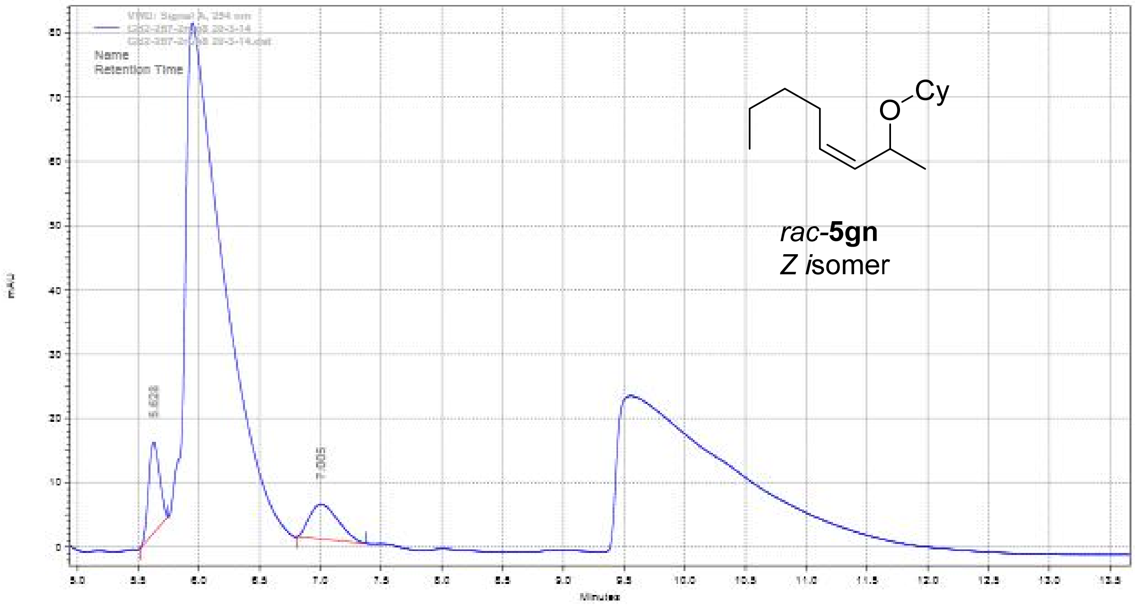

C:\EZChrom Elite\Enterprise\Projects\Default\Data\GB2-267-2run8 20-3-14.dat, VWD: Signal A, 254 nm

VWD: Signal A, 254 nm Results

Retention Time	Area	Area %	Height	Height %
5.628	1480792	49.82	235749	72.21
7.005	1491540	50.18	90717	27.79
Totals	2972332	100.00	326466	100.00

(*S*,*E*)-(Oct-3-en-2-yloxy)cyclohexane (*S*)-5gn

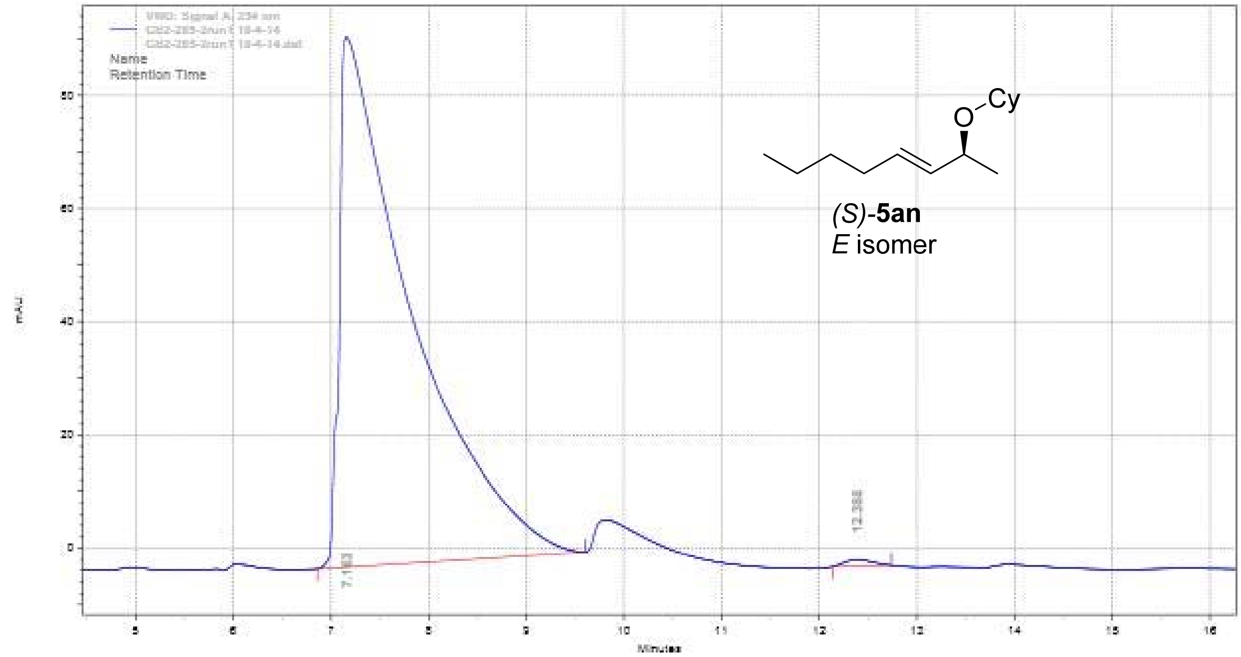

C:\EZChrom Elite\Enterprise\Projects\Default\Data\GB2-285-2run1 10-4-14.dat, VWD: Signal A, 254 nm

VWD: Signal A, 254 nm Results

Retention Time	Area	Area %	Height	Height %
7.163	80196135	99.53	1570170	98.82
12.388	376068	0.47	18694	1.18

(*S*,*Z*)-(Oct-3-en-2-yloxy)cyclohexane (*S*)-5gn

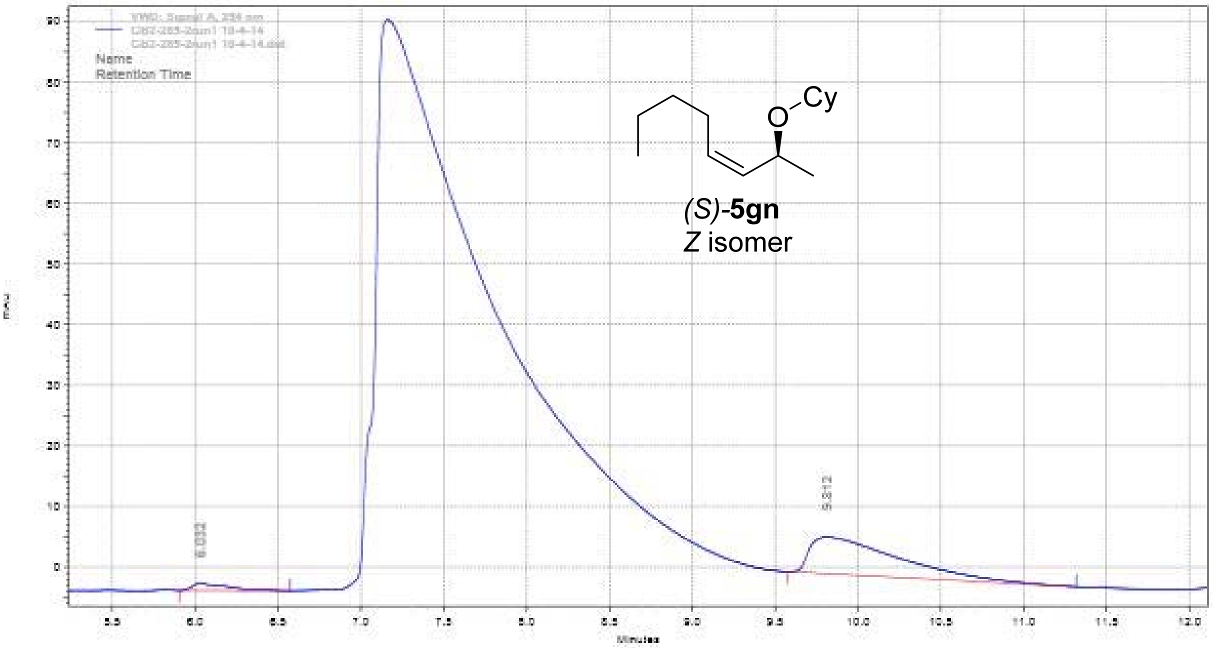

C:\EZChrom Elite\Enterprise\Projects\Default\Data\GB2-285-2run1 10-4-14.dat, VWD: Signal A, 254 nm

VWD: Signal A,
254 nm Results

Retention Time	Area	Area %	Height	Height %
6.032	282379	6.91	18765	15.72
9.812	3803668	93.09	100608	84.28

References

[1] Z. Li, B. T. Parr and H. M. L. Davies, *J. Am. Chem. Soc.*, 2012, **134**, 10942

[2] W. Oppolzer and R. N. Radinov, *Tetrahedron Letters*, 1991, **32**, 5777

[3] K. M. Buchner, T. B. Clark, J. M. N. Loy, T. X. Nguyen and K. A. Woerpel, *Org. Lett.*, 2009, **11**, 2173

[4] J. Ye, J. Zhao, J. Xu, Y. Mao and Y. J. Zhang, *Chem. Commun.*, 2013, **49**, 9761

[5] D. C. Dittmer, R. P. Discordia, Y. Zhang, C. K. Murphy, A. Kumar, A. S. Pepito and Y. Wang, *J. Org. Chem.*, 1993, **58**, 718

[6] P. Mukherjee and R. A. Widenhoefer, *Chem. Eur. J.*, 2013, **19**, 3437

Computational Supporting Information

Contents

Computational Details: 191

Organic Products 192

Ligand Exchange Energies 199

Pathways for Direct Etherification: 207

(i) Single EtOH nucleophile, Pathway (i), anti attack 207

(i) Single EtOH nucleophile, Pathway (ii), *syn* attack 233

(iii) Three EtOH nucleophiles 259

Computational Details:

Calculations were run with Gaussian 03 Revision D.01[1] with PCM solvent corrections run with Gaussian 09, Revision A.02.[2] Geometry optimisations were performed using the BP86 functional[3] with Au and P centres described with the Stuttgart RECPs and associated basis sets[4] (with added d-orbital polarisation on P (ζ = 0.387))[5] and 6-31G** basis sets for all other atoms.[6] All stationary points were fully characterized *via* analytical frequency calculations as either minima (all positive eigenvalues) or transition states (one negative eigenvalue). Frequency calculations also provided a free energy in the gas-phase, computed at 298.15 K and 1 atm. For transition states IRC calculations and subsequent geometry optimizations were used to confirm the minima linked by each transition state. Energies reported in the text are based on the gas-phase free energies and incorporate a correction for dispersion effects using Grimme's D3 parameter set[7] (i.e. BP86-D3) as well as solvation (PCM approach) in toluene and ethanol. All organic fragments were manually searched to identify the lowest energy conformers.

1. Gaussian 03, Revision C.02, M. J. Frisch, G. W. Trucks, H. B. Schlegel, G. E. Scuseria, M. A. Robb, J. R. Cheeseman, J. A. Montgomery, Jr., T. Vreven, K. N. Kudin, J. C. Burant, J. M. Millam, S. S. Iyengar, J. Tomasi, V. Barone, B. Mennucci, M. Cossi, G. Scalmani, N. Rega, G. A. Petersson, H. Nakatsuji, M. Hada, M. Ehara, K. Toyota, R. Fukuda, J. Hasegawa, M. Ishida, T. Nakajima, Y. Honda, O. Kitao, H. Nakai, M. Klene, X. Li, J. E. Knox, H. P. Hratchian, J. B. Cross, C. Adamo, J. Jaramillo, R. Gomperts, R. E. Stratmann, O. Yazyev, A. J. Austin, R. Cammi, C. Pomelli, J. W. Ochterski, P. Y. Ayala, K. Morokuma, G. A. Voth, P. Salvador, J. J. Dannenberg, V. G. Zakrzewski, S. Dapprich, A. D. Daniels, M. C. Strain, O. Farkas, D. K. Malick, A. D. Rabuck, K. Raghavachari, J. B. Foresman, J. V. Ortiz, Q. Cui, A. G. Baboul, S. Clifford, J. Cioslowski, B. B. Stefanov, G. Liu, A. Liashenko, P. Piskorz, I. Komaromi, R. L. Martin, D. J. Fox, T. Keith, M. A. Al-Laham, C. Y. Peng, A. Nanayakkara, M. Challacombe, P. M. W. Gill, B. Johnson, W. Chen, M. W. Wong, C. Gonzalez, and J. A. Pople, Gaussian, Inc., Wallingford CT, **2004**.
2. Gaussian 09, Revision A.02, M. J. Frisch, G. W. Trucks, H. B. Schlegel, G. E. Scuseria, M. A. Robb, J. R. Cheeseman, G. Scalmani, V. Barone, B. Mennucci, G. A. Petersson, H. Nakatsuji, M. Caricato, X. Li, H. P. Hratchian, A. F. Izmaylov, J. Bloino, G. Zheng, J. L. Sonnenberg, M. Hada, M. Ehara, K. Toyota, R. Fukuda, J. Hasegawa, M. Ishida, T. Nakajima, Y. Honda, O. Kitao, H. Nakai, T. Vreven, J. A. Montgomery, Jr., J. E. Peralta, F. Ogliaro, M. Bearpark, J. J. Heyd, E. Brothers, K. N. Kudin, V. N. Staroverov, R. Kobayashi, J. Normand, K. Raghavachari, A. Rendell, J. C. Burant, S. S. Iyengar, J. Tomasi, M. Cossi, N. Rega, J. M. Millam, M. Klene, J. E. Knox, J. B. Cross, V. Bakken, C. Adamo, J. Jaramillo, R. Gomperts, R. E. Stratmann, O. Yazyev, A. J. Austin, R. Cammi, C. Pomelli, J. W. Ochterski, R. L. Martin, K. Morokuma, V. G. Zakrzewski, G. A. Voth, P. Salvador, J. J. Dannenberg, S. Dapprich, A. D. Daniels, O. Farkas, J. B. Foresman, J. V. Ortiz, J. Cioslowski, and D. J. Fox, Gaussian, Inc., Wallingford CT, **2009**.
3. (a) A. D. Becke, *Phys. Rev. A*, **1988**, *38*, 3098. (b) J. P. Perdew, *Physical Review B*, **1986**, 33, 8822.
4. D. Andrae, U. Häußermann, M. Dolg, H. Stoll and H. Preuß, *Theor. Chim. Acta*, **1990**, *77*, 123.
5. A. Hollwarth, M. Bohme, S. Dapprich, A. W. Ehlers, A. Gobbi, V. Jonas, K. F. Kohler, R. Stegmann, A. Veldkamp and G. Frenking, *Chem. Phys. Lett.*, **1993**, *208*, 237.
6. (a) W. J. Hehre, R. Ditchfield and J. A. Pople, J. Chem. Phys. 1972, 56, 2257. (b) P. C. Hariharan and J. A. Pople, *Theor. Chim. Acta.*, **1973**, *28*, 213.
7. S. Grimme, J. Antony, S. Ehrlich and H. Krieg, *J. Chem. Phys.*, **2010**, *132*, 154104.

Organic Products

The lowest energy conformers of (*R*,*E*) **4k** and the *S*,*E* and *R*,*Z* forms of **5ko** were calculated:

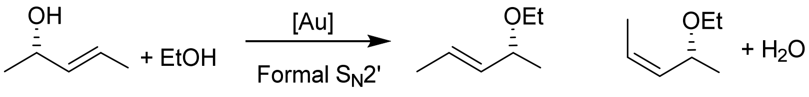

(*R*,*E*) 4k **2o** **(*S*,*E*) 5ko** **(*R*,*Z*) 5ko**

Table S1. Energies of the (*S*,*E*) and (*R*,*Z*) forms of **5ko**. Energies are reported as ΔE (gas phase SCF energies), ΔG (free energies at 298.15 K and 1atm), ΔG_{disp} (including a correction for dispersion effects using Grimme's D3 parameter set) and $\Delta G_{toluene+disp}$ (including an additional correction for toluene solvent *via* the PCM approach).

Isomer	ΔE	ΔG	ΔG_{disp}	$\Delta G_{toluene+disp}$
(*S*,*E*) 5ko	-0.5	-0.6	-2.2	-2.4
(*R*,*Z*) 5ko	+1.1	+1.1	-0.9	-1.1

(*R*,*E*) 4k

16

O	1.725138	1.224555	-0.151754
C	1.031679	0.046385	0.314685
C	-0.348855	-0.026797	-0.291610
C	-1.495575	-0.012240	0.414805
H	-0.380562	-0.073541	-1.390986
C	-2.881323	-0.070711	-0.167460
H	-3.431637	-0.958401	0.196952
H	-3.480798	0.809740	0.131041
H	-2.858616	-0.112561	-1.268974
H	-1.430781	0.039100	1.512966
H	1.116151	1.972595	-0.001461
H	0.932538	0.069629	1.423947
C	1.913848	-1.142459	-0.078838
H	1.490562	-2.085083	0.304024
H	1.995552	-1.210941	-1.176788
H	2.927844	-1.012042	0.333810

SCF(BP86) = -271.749680085
H 0K = -271.612444
H 298K = -271.603614
G 298K = -271.643975
Solvent correction(toluene) = -0.00199119
Solvent correction(ethanol) = -0.004146901
BP86-D3 correction = -0.01064140
Lowest frequencies = 84.2266 cm-1, 158.9769 cm-1

2o

9

O	-1.248963	-0.261164	-0.109076
C	-0.085259	0.557092	0.047343
H	-0.129953	1.286704	-0.780201
C	1.220996	-0.240065	-0.022727
H	-0.122857	1.142194	0.992827
H	1.292136	-0.961527	0.811970
H	1.279233	-0.805133	-0.967612
H	2.098837	0.426772	0.041444
H	-1.240116	-0.901857	0.626484

SCF(BP86) = -155.036798628
H 0K = -154.958855
H 298K = -154.953618
G 298K = -154.984240
Solvent correction(toluene) = -0.002071581
Solvent correction(ethanol) = -0.004304837
BP86-D3 correction = -0.00390074
Lowest frequencies = 265.7162 cm-1, 313.8926 cm-1

4k+2o adduct

25

C	1.538238	0.617917	0.252437
C	0.857668	1.587441	-0.386398
C	1.486769	-0.831639	-0.154578
O	0.579150	-1.502076	0.780824
H	0.627834	-2.457209	0.574499
C	0.873896	3.044059	-0.016919
H	0.217602	1.298243	-1.232373
H	2.155367	0.858941	1.130518
H	-0.143673	3.401525	0.227144
H	1.227361	3.666790	-0.859970
H	1.524300	3.240329	0.851558
O	-1.638916	-0.864325	-0.837465
C	-2.946606	-0.740056	-0.287310
H	-1.012484	-1.030532	-0.090654
C	-3.098371	0.466982	0.647232
H	-3.253755	-1.665456	0.251550
H	-3.633494	-0.636854	-1.147731
H	-2.841553	1.399513	0.117291
H	-2.427226	0.375954	1.519329
H	-4.133575	0.553322	1.023869
C	2.865033	-1.502945	-0.120566
H	1.058732	-0.902238	-1.175111
H	3.558672	-1.003883	-0.817879
H	3.293365	-1.451993	0.894931
H	2.800888	-2.565781	-0.417231

SCF(BP86) = -426.794822128
H 0K = -426.577946
H 298K = -426.563228
G 298K = -426.620744
Solvent correction(toluene) = -0.00322877
Solvent correction(ethanol) = -0.006791169
BP86-D3 correction = -0.02052680
Lowest frequencies = 18.1852 cm-1, 27.4101 cm-1

(*S,E*) 5ko

22

O	1.329882	0.422182	-0.171892
C	0.069651	0.944020	0.294394
C	-1.096731	0.197576	-0.313954
C	-2.040911	-0.458047	0.386798
C	1.629954	-0.881012	0.333266
C	2.986608	-1.300745	-0.222437
H	1.652213	-0.861988	1.447102
H	0.842757	-1.607571	0.040706
H	3.264236	-2.300172	0.152659
H	2.959699	-1.337070	-1.323894
H	3.769136	-0.584766	0.078124
H	0.019016	0.846333	1.403707
C	0.065490	2.428646	-0.088286
H	-0.850991	2.920489	0.275841
H	0.943133	2.935057	0.345541
H	0.110612	2.540166	-1.184842
H	-1.144100	0.215397	-1.413591
C	-3.218370	-1.185733	-0.202824
H	-4.174033	-0.756902	0.152999
H	-3.214102	-1.141072	-1.304476
H	-3.224779	-2.250024	0.098099
H	-1.965999	-0.463568	1.485413

SCF(BP86) = -350.369431843
H 0K = -350.177442
H 298K = -350.165771
G 298K = -350.213686
Solvent correction(toluene) = -0.00122894
Solvent correction(ethanol) = -0.002705845
BP86-D3 correction = -0.01702856
Lowest frequencies = 52.4704 cm-1, 68.5437 cm-1

(*R*,*Z*) 5ko

22

O	1.220535	0.548740	0.017961
C	-0.178709	0.796349	-0.226223
C	-1.032398	0.130900	0.831853
C	-2.063387	-0.721704	0.657401
C	1.607040	-0.807717	-0.215624
C	3.099317	-0.929283	0.073618
H	1.023633	-1.498945	0.429015
H	1.389311	-1.089799	-1.271339
H	3.444030	-1.961474	-0.106191
H	3.677939	-0.249754	-0.573507
H	3.312872	-0.668293	1.123134
C	-0.344172	2.321129	-0.210273
H	-0.443103	0.410800	-1.235455
H	-0.744890	0.401150	1.858231
C	-2.636923	-1.227901	-0.640451
H	-2.658462	-2.333297	-0.655017
H	-3.685122	-0.895996	-0.762915
H	-2.073542	-0.886835	-1.522982
H	-2.566578	-1.094421	1.561376
H	-1.384401	2.600724	-0.442946
H	-0.088596	2.722429	0.784907
H	0.328025	2.783158	-0.951805

SCF(BP86) = -350.36675549
H 0K = -350.174553
H 298K = -350.162942
G 298K = -350.210979
Solvent correction(toluene) = -0.00124978
Solvent correction(ethanol) = -0.002749641
BP86-D3 correction = -0.01766432
Lowest frequencies = 36.1229 cm-1, 64.3052 cm-1

H_2O

3

O	0.000000	0.121120	0.000000
H	0.762492	-0.484454	0.000000
H	-0.762492	-0.484510	0.000000

SCF(BP86) = -76.4179149291
H 0K = -76.397125
H 298K = -76.393346
G 298K = -76.415464
Solvent correction(toluene) = -0.003251649
Solvent correction(ethanol) = -0.006553739
BP86-D3 correction = -0.00002181
Lowest frequencies = 1629.7801 cm-1, 3687.9216 cm-1

Ligand Exchange Energies

The exchange of the NTf_2 ligand with the allyl and OH bound **4k** at the Au centre was computed and is reported here, along with solvation energies for toluene and ethanol.

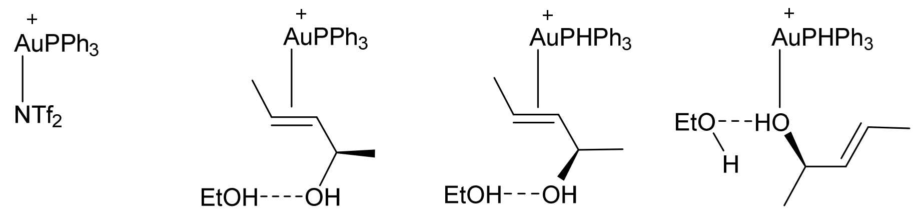

NTf_2 **Ia** **Ib** **Ie**

Table S2. Energies of ligand exchange at the Au centre. Energies are reported as ΔE (gas phase SCF energies), ΔG (free energies at 298.15 K and 1atm), ΔG_{disp} (including a correction for dispersion effects using Grimme's D3 parameter set), $\Delta G_{toluene+disp}$ (including an additional correction for toluene solvent *via* the PCM approach) and $\Delta G_{ethanol+disp}$ (including a correction for ethanol instead of toluene). All energies relative to **NTf_2**.

**Isomer**	**ΔE**	**ΔG**	**ΔG_{disp}**	**$\Delta G_{toluene+disp}$**	**$\Delta G_{ethanol+disp}$**
**NTf_2**	+0.0	+0.0	+0.0	+0.0	+0.0
**Ia**	+69.4	+71.7	+71.6	+33.4	+9.0
**Ib**	+74.4	+75.1	+78.4	+40.0	+15.2
**Ie**	+64.8	+64.6	+69.5	+32.1	+8.4

NTf_2 (fragment)

15

S	1.146708	-0.867736	0.110894
N	0.000015	0.000039	0.942115
S	-1.146727	0.867760	0.110912
O	-0.842898	1.277765	-1.297805
C	-2.577794	-0.387744	-0.061440
O	-1.701549	1.877553	1.068592
O	1.701491	-1.877568	1.068555
C	2.577819	0.387721	-0.061447
O	0.842848	-1.277708	-1.297826
F	3.610853	-0.199319	-0.720089
F	3.022157	0.779127	1.154880
F	2.201045	1.483242	-0.753608
F	-2.200988	-1.483256	-0.753598
F	-3.610840	0.199273	-0.720083
F	-3.022124	-0.779162	1.154887

SCF(BP86) = -1051.29286606
H 0K = -1051.243133
H 298K = -1051.226949
G 298K = -1051.288284
Solvent correction(toluene) = -0.04217749
Solvent correction(ethanol)= -0.07173155
BP86-D3 correction = -0.01990791
Lowest frequencies = 27.8924 cm-1, 34.9370 cm-1

Nucleophile-allylic alcohol adduct

25

C	1.538238	0.617917	0.252437
C	0.857668	1.587441	-0.386398
C	1.486769	-0.831639	-0.154578
O	0.579150	-1.502076	0.780824
H	0.627834	-2.457209	0.574499
C	0.873896	3.044059	-0.016919
H	0.217602	1.298243	-1.232373
H	2.155367	0.858941	1.130518
H	-0.143673	3.401525	0.227144
H	1.227361	3.666790	-0.859970
H	1.524300	3.240329	0.851558
O	-1.638916	-0.864325	-0.837465
C	-2.946606	-0.740056	-0.287310
H	-1.012484	-1.030532	-0.090654
C	-3.098371	0.466982	0.647232
H	-3.253755	-1.665456	0.251550
H	-3.633494	-0.636854	-1.147731
H	-2.841553	1.399513	0.117291
H	-2.427226	0.375954	1.519329
H	-4.133575	0.553322	1.023869
C	2.865033	-1.502945	-0.120566
H	1.058732	-0.902238	-1.175111
H	3.558672	-1.003883	-0.817879
H	3.293365	-1.451993	0.894931

H 2.800888 -2.565781 -0.417231

SCF(BP86) = -426.794822128
H 0K = -426.577946
H 298K = -426.563228
G 298K = -426.620744
Solvent correction(toluene) = -0.003228773
Solvent correction(ethanol)= -0.006791169
BP86-D3 correction = -0.0205268
Lowest frequencies = 18.1852 cm-1, 27.4101 cm-1

NTf_2 (Complex)

50

P	-2.126971	-0.020432	0.001435
C	-2.769602	1.559973	-0.711980
C	-2.851702	-1.390640	-1.009678
C	-2.882418	-0.183230	1.681656
Au	0.155507	-0.053178	0.026068
S	3.110670	-1.201797	0.880975
N	2.246208	-0.019025	-0.003234
S	3.010945	1.240608	-0.869425
O	1.998023	1.729030	-1.852456
C	3.122537	2.630441	0.446426
O	4.404362	0.912642	-1.264136
O	4.459494	-0.744659	1.302535
C	3.376190	-2.566702	-0.437116
O	2.126374	-1.782144	1.841136
F	4.051630	-3.576983	0.139925
F	4.071272	-2.090254	-1.478259
F	2.178180	-3.017905	-0.865224
F	1.878617	2.989435	0.830312
F	3.736582	3.687563	-0.114549
F	3.815474	2.215215	1.515229
C	-3.700986	3.942630	-1.875514
C	-2.442857	3.431716	-2.234740
C	-1.971545	2.244447	-1.654038
C	-4.028896	2.078801	-0.344654
C	-4.490888	3.268321	-0.929707
H	-4.062175	4.873393	-2.324833
H	-1.817619	3.962077	-2.959766
H	-0.979018	1.859792	-1.915727
H	-4.638568	1.565625	0.406256
H	-5.466447	3.671478	-0.639242
C	-3.900418	-3.545360	-2.479510
C	-4.501399	-2.279459	-2.563617
C	-3.981098	-1.199826	-1.830857
C	-2.243010	-2.662170	-0.933249
C	-2.771799	-3.735694	-1.664012
H	-4.305806	-4.382818	-3.056430
H	-5.374963	-2.126510	-3.205535
H	-4.444583	-0.211179	-1.908384
H	-1.352327	-2.804503	-0.310974
H	-2.294167	-4.718753	-1.604260
C	-3.993389	-0.342866	4.258554
C	-4.692430	-0.867774	3.159399
C	-4.140565	-0.791848	1.870587
C	-2.177112	0.334378	2.788665
C	-2.736300	0.256175	4.072634
H	-4.423786	-0.410391	5.262901
H	-5.666963	-1.345211	3.304043
H	-4.679244	-1.218040	1.017879
H	-1.187431	0.781598	2.642667
H	-2.182344	0.652701	4.929293

SCF(BP86) = -1888.66746417
H 0K = -1888.349127
H 298K = -1888.312359
G 298K = -1888.426758
Solvent correction(toluene) = -0.00931500

Solvent correction(ethanol) = -0.0187354
BP86-D3 correction = -0.08324212
Lowest frequencies = 6.1401 cm-1, 12.1915 cm-1

Ia

60

P	-1.269153	-0.019877	0.087412
C	-2.443999	-1.433555	-0.070749
C	-1.471086	0.676065	1.784221
Au	0.952280	-0.658071	-0.321711
C	2.966188	-1.761395	-0.043751
C	2.973159	-1.242070	-1.333718
C	3.721984	-1.169643	1.133937
O	4.104806	0.180849	0.803594
H	4.730783	0.482712	1.488931
C	2.639651	-2.029190	-2.576934
H	3.471184	-0.274252	-1.478449
H	2.642714	-2.801243	0.106152
C	-1.833104	1.270391	-1.106322
H	3.585276	-2.296479	-3.085931
H	2.052616	-1.433929	-3.295005
H	2.095738	-2.960113	-2.352811
C	-1.750109	1.791630	4.343766
C	-2.691410	0.858875	3.879963
C	-2.557573	0.297797	2.599610
C	-0.517506	1.610682	2.248778
C	-0.668495	2.166881	3.528051
H	-1.858100	2.225422	5.342864
H	-3.533519	0.564040	4.513693
H	-3.292149	-0.429698	2.241361
H	0.329541	1.900560	1.613395
H	0.064983	2.894362	3.890277
C	-2.720169	3.167330	-2.979313
C	-2.992657	3.347976	-1.614097
C	-2.551758	2.402751	-0.672984
C	-1.555018	1.091912	-2.479298
C	-2.002508	2.038340	-3.411694
H	-3.064622	3.907410	-3.708248
H	-3.550149	4.226925	-1.275948
H	-2.762700	2.548322	0.390733
H	-0.995144	0.212386	-2.817462
H	-1.787827	1.896847	-4.475511
C	-4.224638	-3.602027	-0.223581
C	-4.639058	-2.323464	-0.630749
C	-3.753988	-1.236054	-0.555556
C	-2.027804	-2.720230	0.333251
C	-2.919984	-3.799754	0.259886
H	-4.917113	-4.447019	-0.287931
H	-5.653397	-2.169351	-1.011495
H	-4.078992	-0.242398	-0.879332
H	-1.006370	-2.873177	0.700307
H	-2.594363	-4.796650	0.572778
O	2.030660	1.974104	0.118150
C	2.446350	2.962313	-0.849801
H	2.828562	1.476444	0.421486
C	3.295103	4.069795	-0.226255
H	1.513420	3.376653	-1.266306
H	2.993660	2.479529	-1.685870

H	4.243539	3.671787	0.175437
H	2.751291	4.563830	0.595366
H	3.549298	4.833848	-0.980915
C	4.960523	-2.032021	1.441975
H	3.056726	-1.168446	2.022821
H	4.672290	-3.066756	1.689533
H	5.644964	-2.050221	0.578585
H	5.498772	-1.625239	2.315714

SCF(BP86) = -1264.05887825
H 0K = -1263.572427
H 298K = -1263.537835
G 298K = -1263.644941
Solvent correction(toluene) = -0.03132121
Solvent correction(ethanol) = -0.05353354
BP86-D3 correction = -0.08400405
Lowest frequencies = 10.0318 cm-1, 18.4943 cm-1

Ib

60

P	-1.544864	0.137682	0.101894
C	-2.789293	-0.987441	-0.659270
C	-1.729805	0.026182	1.932722
Au	0.648846	-0.391269	-0.552938
C	2.659387	-1.406865	-0.926445
C	2.779250	-0.129861	-1.473099
C	3.257715	-1.770679	0.432544
O	4.688707	-1.823201	0.179792
H	5.087362	-2.381156	0.876771
C	2.640340	0.180996	-2.938886
H	3.231598	0.647061	-0.829506
H	2.436009	-2.249986	-1.599252
C	-1.990141	1.857833	-0.395770
H	3.657612	0.319609	-3.352930
H	2.103634	1.128520	-3.110464
H	2.149456	-0.627452	-3.503570
C	-1.976959	-0.070137	4.728304
C	-3.012403	-0.558194	3.915107
C	-2.895109	-0.511867	2.516477
C	-0.685393	0.511483	2.750905
C	-0.815127	0.466199	4.146410
H	-2.072750	-0.110894	5.817698
H	-3.915596	-0.978861	4.367602
H	-3.702564	-0.896289	1.885833
H	0.223543	0.924810	2.298397
H	-0.006252	0.844476	4.779022
C	-2.676979	4.450827	-1.226098
C	-2.934170	4.045272	0.092894
C	-2.593240	2.749307	0.514335
C	-1.727571	2.267270	-1.722071
C	-2.075965	3.560992	-2.133790
H	-2.942010	5.462502	-1.548263
H	-3.401196	4.737337	0.800283
H	-2.793126	2.437408	1.543818
H	-1.258804	1.575072	-2.430869
H	-1.874020	3.876490	-3.162013
C	-4.681127	-2.752458	-1.751036
C	-5.013367	-1.402154	-1.554056

C	-4.071722	-0.514765	-1.009064
C	-2.454800	-2.343766	-0.862644
C	-3.402917	-3.223122	-1.404968
H	-5.417321	-3.438741	-2.180673
H	-6.006907	-1.034488	-1.828208
H	-4.330694	0.538844	-0.865225
H	-1.455171	-2.708528	-0.599500
H	-3.141400	-4.273703	-1.564240
O	4.605736	0.961896	0.762783
C	5.641256	1.878676	1.160159
H	5.019599	0.092070	0.553943
C	6.664214	2.138960	0.053536
H	6.150272	1.513031	2.076060
H	5.120539	2.812756	1.432003
H	6.172473	2.535058	-0.850563
H	7.200632	1.213672	-0.222053
H	7.418338	2.873571	0.384376
C	2.728949	-3.100530	0.971371
H	3.060512	-0.952914	1.154523
H	1.641805	-3.045549	1.151406
H	2.925741	-3.920066	0.259262
H	3.207105	-3.351124	1.934008

SCF(BP86) = -1264.05080342
H 0K = -1263.564816
H 298K = -1263.530079
G 298K = -1263.639521
Solvent correction(toluene) = -0.03155091
Solvent correction(ethanol) = -0.05454000
BP86-D3 correction = -0.07866358
Lowest frequencies = 3.9853 cm-1, 12.1102 cm-1

Ie

60

P	1.404157	0.249582	-0.004919
C	1.806282	2.031129	-0.273996
C	2.265304	-0.259382	1.544409
Au	-0.848513	-0.107774	0.115524
C	2.170378	-0.694537	-1.392712
C	3.588745	-1.114392	3.869245
C	4.048972	0.016957	3.176215
C	3.390940	0.449593	2.013748
C	1.800262	-1.393502	2.244575
C	2.465546	-1.818995	3.403305
H	4.101946	-1.444421	4.777755
H	4.920037	0.569443	3.541705
H	3.746500	1.337408	1.481571
H	0.918387	-1.935302	1.884753
H	2.101932	-2.696199	3.947307
C	3.323003	-2.061927	-3.559539
C	4.015501	-1.965821	-2.341846
C	3.444603	-1.282995	-1.255598
C	1.471974	-0.797591	-2.615617
C	2.052295	-1.476865	-3.696364
H	3.771224	-2.597407	-4.402204
H	5.002835	-2.424993	-2.232651
H	3.985252	-1.214699	-0.306576
H	0.476956	-0.349626	-2.717503

H	1.509609	-1.555068	-4.643552
C	2.418742	4.754873	-0.587908
C	3.082257	3.792440	-1.364881
C	2.781110	2.428841	-1.211219
C	1.135202	3.000389	0.503883
C	1.446833	4.358163	0.347469
H	2.656523	5.815930	-0.711761
H	3.837621	4.099751	-2.094676
H	3.299773	1.681725	-1.819571
H	0.375069	2.691231	1.230399
H	0.928752	5.107666	0.953862
C	-2.744715	-2.772309	0.329925
C	-2.112149	-3.668619	-0.456216
C	-3.573342	-1.640426	-0.202999
O	-2.967174	-0.371935	0.289330
H	-3.503318	0.430092	-0.046722
C	-1.340780	-4.860955	0.029271
H	-2.175802	-3.541850	-1.547897
H	-2.723320	-2.880000	1.424109
H	-0.302848	-4.842211	-0.349932
H	-1.790260	-5.797197	-0.349490
H	-1.312770	-4.914892	1.129438
C	-5.027774	-1.671863	0.271889
H	-3.526849	-1.631023	-1.308765
H	-5.520030	-2.583664	-0.103345
H	-5.077649	-1.677552	1.373153
H	-5.586383	-0.800524	-0.108221
O	-4.364068	1.663843	-0.546169
H	-4.041705	2.138909	-1.335862
C	-4.843652	2.651933	0.417895
C	-5.976453	3.491069	-0.158802
H	-4.000318	3.284338	0.753979
H	-5.184189	2.054452	1.278131
H	-6.814844	2.853961	-0.482095
H	-5.636928	4.090660	-1.021816
H	-6.349575	4.194993	0.603797

SCF(BP86) = -1264.06611690
H 0K = -1263.580059
H 298K = -1263.545242
G 298K = -1263.656233
Solvent correction(toluene) = -0.02992969
Solvent correction(ethanol) = -0.0511787
BP86-D3 correction = -0.0760919
Lowest frequencies = 3.5174 cm-1, 15.5961 cm-1

Pathways for Direct Etherification:

(i) Single EtOH nucleophile, Pathway (i), anti attack

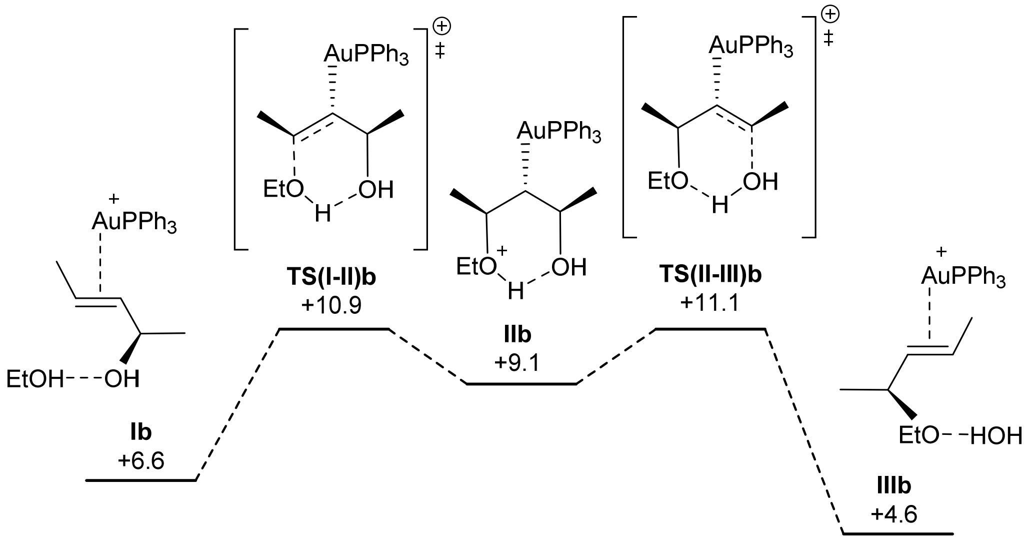

Figure S3. Computed free energy profile (BP86-D3(toluene)) for direct etherification with a single EtOH nucleophile at **Ib** *via* Pathway (i) (*anti* attack) leading to (*S*,*E*)-**5ko**. All energies are in kcal/mol and are quoted relative to **Ia** set to 0.0 kcal/mol.

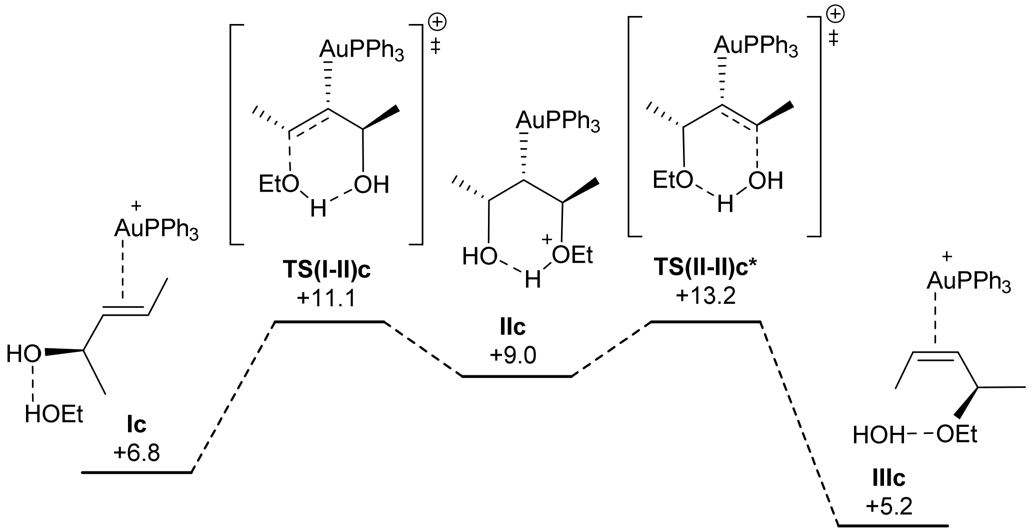

Figure S4. Computed free energy profile (BP86-D3(toluene)) for direct etherification with a single EtOH nucleophile at **Ic** *via* Pathway (i) (*anti* attack) leading to (*R*,*Z*)-**5ko**. All energies are in kcal/mol and are quoted relative to **Ia** set to 0.0 kcal/mol. * a residual second imaginary mode of -5 cm^{-1} was computed.

Table S3. Energies associated with direct etherification with a single EtOH nucleophile *via* Pathway (i) (*anti* attack). Energies are reported as ΔE (gas phase SCF energies), ΔG (free energies at 298.15 K and 1atm), ΔG_{disp} (including a correction for dispersion effects using Grimme's D3 parameter set) and $\Delta G_{toluene+disp}$ (including an additional correction for toluene solvent *via* the PCM approach). All energies relative to **Ia**.

Isomer	ΔE	ΔG	ΔG_{disp}	$\Delta G_{toluene+disp}$
Ib	+5.1	+3.4	+6.8	+6.6
Ic	+6.1	+4.7	+7.0	+6.8
TS(I-II)b	+8.8	+9.7	+10.5	+10.9
TS(I-II)c	+9.4	+10.6	+10.8	+11.1
IIb	+7.0	+8.7	+9.4	+9.1
IIc	+7.4	+9.2	+9.3	+9.0
TS(II-III)b	+10.0	+10.7	+11.2	+11.1
TS(II-III)c	+9.8	+13.0	+13.0	+13.2
IIIb	+5.7	+3.7	+4.7	+4.6
IIIc	+5.5	+4.4	+5.4	+5.2

Ib

60

P	-1.544864	0.137682	0.101894
C	-2.789293	-0.987441	-0.659270
C	-1.729805	0.026182	1.932722
Au	0.648846	-0.391269	-0.552938
C	2.659387	-1.406865	-0.926445
C	2.779250	-0.129861	-1.473099
C	3.257715	-1.770679	0.432544
O	4.688707	-1.823201	0.179792
H	5.087362	-2.381156	0.876771
C	2.640340	0.180996	-2.938886
H	3.231598	0.647061	-0.829506
H	2.436009	-2.249986	-1.599252
C	-1.990141	1.857833	-0.395770
H	3.657612	0.319609	-3.352930
H	2.103634	1.128520	-3.110464
H	2.149456	-0.627452	-3.503570
C	-1.976959	-0.070137	4.728304
C	-3.012403	-0.558194	3.915107
C	-2.895109	-0.511867	2.516477
C	-0.685393	0.511483	2.750905
C	-0.815127	0.466199	4.146410
H	-2.072750	-0.110894	5.817698
H	-3.915596	-0.978861	4.367602
H	-3.702564	-0.896289	1.885833
H	0.223543	0.924810	2.298397
H	-0.006252	0.844476	4.779022
C	-2.676979	4.450827	-1.226098
C	-2.934170	4.045272	0.092894
C	-2.593240	2.749307	0.514335
C	-1.727571	2.267270	-1.722071
C	-2.075965	3.560992	-2.133790
H	-2.942010	5.462502	-1.548263
H	-3.401196	4.737337	0.800283
H	-2.793126	2.437408	1.543818
H	-1.258804	1.575072	-2.430869
H	-1.874020	3.876490	-3.162013
C	-4.681127	-2.752458	-1.751036
C	-5.013367	-1.402154	-1.554056
C	-4.071722	-0.514765	-1.009064
C	-2.454800	-2.343766	-0.862644
C	-3.402917	-3.223122	-1.404968
H	-5.417321	-3.438741	-2.180673
H	-6.006907	-1.034488	-1.828208
H	-4.330694	0.538844	-0.865225
H	-1.455171	-2.708528	-0.599500
H	-3.141400	-4.273703	-1.564240
O	4.605736	0.961896	0.762783
C	5.641256	1.878676	1.160159
H	5.019599	0.092070	0.553943
C	6.664214	2.138960	0.053536
H	6.150272	1.513031	2.076060
H	5.120539	2.812756	1.432003
H	6.172473	2.535058	-0.850563
H	7.200632	1.213672	-0.222053
H	7.418338	2.873571	0.384376
C	2.728949	-3.100530	0.971371
H	3.060512	-0.952914	1.154523

H	1.641805	-3.045549	1.151406
H	2.925741	-3.920066	0.259262
H	3.207105	-3.351124	1.934008

SCF(BP86) = -1264.05080342
H 0K = -1263.564816
H 298K = -1263.530079
G 298K = -1263.639521
Solvent correction(toluene) = -0.03155091
Solvent correction(ethanol) = -0.05454
BP86-D3 correction = -0.07866358
Lowest frequencies = 3.9853 cm-1, 12.1102 cm-1

Ic

60

P	1.718604	-0.024899	-0.054736
C	2.658345	-0.332564	1.500524
C	2.213412	-1.321384	-1.269586
Au	-0.603896	-0.072190	0.310770
C	-2.744688	0.330546	0.973207
C	-2.825681	-0.819884	0.189202
C	-3.219477	1.722861	0.534770
O	-4.597653	1.845265	0.972154
H	-4.615637	1.921361	1.945753
C	-2.919770	-2.218640	0.728805
H	-3.071452	-0.699513	-0.873020
H	-2.693014	0.190074	2.065733
C	2.279016	1.600280	-0.721528
H	-2.333959	-2.938640	0.134682
H	-3.984256	-2.506014	0.633436
H	-2.628638	-2.289713	1.788642
C	2.943881	-3.252246	-3.173399
C	3.732793	-3.062970	-2.027453
C	3.372966	-2.098787	-1.071832
C	1.417008	-1.515567	-2.420097
C	1.787597	-2.477410	-3.370464
H	3.227212	-4.007360	-3.913034
H	4.631571	-3.667583	-1.872224
H	3.988055	-1.956965	-0.178015
H	0.512846	-0.915056	-2.572995
H	1.170262	-2.626101	-4.261704
C	3.132393	4.105235	-1.662011
C	3.624647	2.929761	-2.250894
C	3.202331	1.674007	-1.784378
C	1.781168	2.783288	-0.131892
C	2.212628	4.031813	-0.601238
H	3.462566	5.081032	-2.031243
H	4.339296	2.986147	-3.077665
H	3.585849	0.759869	-2.247605
H	1.062973	2.727459	0.694338
H	1.827289	4.947615	-0.142551
C	4.086301	-0.883533	3.854982
C	4.574008	0.068566	2.945270
C	3.864741	0.348167	1.766057
C	2.166070	-1.286451	2.418126
C	2.883888	-1.561833	3.590651
H	4.641159	-1.094809	4.774250
H	5.508409	0.599133	3.152270
H	4.244707	1.094569	1.061725
H	1.225967	-1.811875	2.214257
H	2.501202	-2.301097	4.300966
O	-5.555974	-0.446651	-0.479726
C	-6.874876	-0.571245	-1.042042
H	-5.535503	0.359212	0.085246
C	-7.948828	-0.818481	0.018647
H	-6.820856	-1.425497	-1.738939
H	-7.129765	0.324372	-1.645677
H	-8.032255	0.038883	0.709938
H	-7.713675	-1.718101	0.611274
H	-8.936868	-0.962685	-0.451413
C	-3.226508	1.981300	-0.969486
H	-2.605521	2.489286	1.047517

H	-2.217008	1.866252	-1.398740
H	-3.921595	1.285666	-1.467788
H	-3.571830	3.008681	-1.160277

SCF(BP86) = -1264.04912125
H 0K = -1263.562914
H 298K = -1263.528212
G 298K = -1263.637501
Solvent correction(toluene) = -0.03166331
BP86-D3 correction = -0.08023874
Lowest frequencies = 7.0816 cm-1, 12.3129 cm-1

TS(I-II)b

60

P	-1.642110	0.073930	0.054139
C	-2.596802	-0.487981	-1.421946
C	-2.239731	-0.918974	1.492008
Au	0.675931	-0.144393	-0.253733
C	2.799094	-0.501248	-0.620393
C	3.272245	0.778073	-0.173279
C	3.272692	-1.739970	0.140401
O	4.695899	-1.874118	-0.202144
H	5.067333	-2.634192	0.286029
C	3.111393	2.038289	-0.981584
H	3.370195	0.913585	0.913537
H	2.846272	-0.639282	-1.715232
C	-2.149425	1.823931	0.365972
H	3.942415	2.733099	-0.786726
H	2.180455	2.555282	-0.689033
H	3.069880	1.823560	-2.060393
C	-3.112457	-2.374445	3.732814
C	-3.909559	-2.317494	2.578218
C	-3.478956	-1.590829	1.456403
C	-1.436158	-0.983031	2.651610
C	-1.876858	-1.705664	3.769884
H	-3.451617	-2.944073	4.603579
H	-4.869898	-2.841336	2.546601
H	-4.101326	-1.552643	0.557061
H	-0.468903	-0.467798	2.676224
H	-1.252883	-1.751930	4.667888
C	-2.894906	4.505546	0.758294
C	-3.368437	3.510588	1.627607
C	-3.000132	2.168347	1.435430
C	-1.669487	2.826924	-0.505474
C	-2.047467	4.162592	-0.310010
H	-3.184177	5.549680	0.912563
H	-4.028298	3.775461	2.459567
H	-3.372350	1.395906	2.115095
H	-1.009282	2.561259	-1.339164
H	-1.678241	4.936794	-0.989868
C	-4.042350	-1.428654	-3.640777
C	-4.526481	-0.339358	-2.898205
C	-3.808239	0.134969	-1.788874
C	-2.109185	-1.579229	-2.173122
C	-2.834628	-2.048579	-3.277751
H	-4.603624	-1.791715	-4.507300
H	-5.464518	0.147036	-3.183164
H	-4.184454	0.989595	-1.217911
H	-1.162775	-2.056601	-1.894374
H	-2.452958	-2.893315	-3.859500
O	5.311161	0.647086	-0.343144
C	6.057664	1.170899	0.787167
H	5.323498	-0.361347	-0.295599
C	7.558720	1.006310	0.564402
H	5.734426	0.671892	1.722618
H	5.787563	2.238201	0.860049
H	7.871163	1.504415	-0.366620
H	7.842196	-0.057705	0.503390
H	8.114099	1.456628	1.404291
C	2.540191	-3.029662	-0.232993
H	3.188107	-1.556111	1.232104

H	1.479599	-2.974089	0.064741
H	2.594232	-3.204632	-1.320259
H	2.986037	-3.899398	0.280097

SCF(BP86) = -1264.04490859
H 0K = -1263.558477
H 298K = -1263.525185
G 298K = -1263.629540
Solvent correction(toluene) = -0.03065969
BP86-D3 correction = -0.08270367
Lowest frequencies = -107.2871 cm-1, 7.9837 cm-1

TS(I-II)c

60

P	1.667104	-0.041638	-0.045328
C	2.098592	-1.264512	-1.361248
C	2.359764	1.584013	-0.582595
Au	-0.647654	0.073703	0.336249
C	-2.761642	0.322238	0.810499
C	-3.273488	-0.803754	0.082558
C	-3.240790	1.733988	0.430853
O	-4.659948	1.878962	0.814779
H	-4.697781	2.022491	1.780912
C	-3.134005	-2.220187	0.571696
H	-3.392790	-0.687421	-1.002847
H	-2.769588	0.165174	1.904787
C	2.598310	-0.536309	1.469911
H	-2.222170	-2.675877	0.146307
H	-3.986020	-2.831643	0.236692
H	-3.069124	-2.266645	1.669708
C	3.404032	4.088668	-1.314710
C	3.828356	2.928742	-1.982035
C	3.310592	1.674262	-1.619527
C	1.930060	2.752506	0.084378
C	2.456453	3.999841	-0.280113
H	3.808934	5.063911	-1.602205
H	4.564552	2.996597	-2.788956
H	3.642114	0.772790	-2.143863
H	1.188856	2.684173	0.889068
H	2.123323	4.903344	0.240017
C	3.992873	-1.373220	3.761727
C	4.555006	-0.407128	2.911653
C	3.862760	0.014521	1.764953
C	2.032036	-1.503085	2.329104
C	2.732335	-1.921715	3.469638
H	4.534843	-1.695779	4.656119
H	5.534587	0.023779	3.140620
H	4.301325	0.772347	1.108342
H	1.045263	-1.924120	2.104914
H	2.290639	-2.670667	4.134254
C	2.723788	-3.071781	-3.421135
C	3.525689	-3.014042	-2.270132
C	3.218454	-2.112223	-1.238131
C	1.289030	-1.328526	-2.516803
C	1.606761	-2.227489	-3.544952
H	2.966305	-3.777691	-4.221508
H	4.393929	-3.672851	-2.171063
H	3.844247	-2.072250	-0.341328
H	0.414547	-0.674340	-2.610325
H	0.979315	-2.272986	-4.440578
O	-5.295416	-0.630036	0.340488
C	-6.120678	-0.892241	-0.827376
H	-5.270708	0.367139	0.514003
C	-7.594271	-0.656503	-0.508503
H	-5.938663	-1.947577	-1.092209
H	-5.791168	-0.263215	-1.677902
H	-7.785604	0.400361	-0.258673
H	-7.916096	-1.283271	0.337936
H	-8.211887	-0.912943	-1.385645
C	-3.173511	2.100147	-1.050767

H	-2.662678	2.474147	1.012848
H	-2.133871	2.049765	-1.412881
H	-3.789654	1.424101	-1.667364
H	-3.553012	3.122523	-1.198761

SCF(BP86) = -1264.04382996
H 0K = -1263.556941
H 298K = -1263.523789
G 298K = -1263.628034
Solvent correction(toluene) = -0.03091901
BP86-D3 correction = -0.08362608
Lowest frequencies = -118.4549 cm-1, 6.3197 cm-1

IIb

60

P	-1.630584	0.067442	0.035876
C	-2.584141	-0.505343	-1.440502
C	-2.294379	-0.875675	1.482097
Au	0.697629	-0.193164	-0.215271
C	2.802290	-0.453976	-0.486541
C	3.509665	0.789719	-0.006419
C	3.278435	-1.731219	0.196213
O	4.756592	-1.846108	-0.128751
H	5.181112	-2.483340	0.479819
C	3.194620	2.082678	-0.741089
H	3.449671	0.905084	1.091574
H	2.936582	-0.556865	-1.581909
C	-2.126956	1.831364	0.298825
H	3.880471	2.896364	-0.454517
H	2.171501	2.402297	-0.486879
H	3.254225	1.935839	-1.831184
C	-3.250794	-2.257051	3.738135
C	-4.029343	-2.192159	2.571347
C	-3.556755	-1.502546	1.442986
C	-1.510265	-0.948931	2.654282
C	-1.991996	-1.633668	3.779668
H	-3.622597	-2.797341	4.614348
H	-5.008020	-2.680829	2.535603
H	-4.165157	-1.458716	0.534296
H	-0.524531	-0.470063	2.681165
H	-1.382013	-1.686130	4.687059
C	-2.839836	4.534776	0.609593
C	-3.369454	3.564327	1.474261
C	-3.017931	2.212429	1.321891
C	-1.590802	2.810590	-0.566829
C	-1.952216	4.156499	-0.412939
H	-3.116845	5.586534	0.731801
H	-4.060716	3.856196	2.271094
H	-3.434502	1.459501	1.997876
H	-0.898754	2.516908	-1.364593
H	-1.538913	4.911091	-1.089674
C	-4.014378	-1.465831	-3.662988
C	-4.494165	-0.358885	-2.944021
C	-3.783547	0.124616	-1.833702
C	-2.100910	-1.613540	-2.169208
C	-2.818376	-2.092953	-3.274995
H	-4.569628	-1.836701	-4.530179
H	-5.422907	0.133794	-3.248369
H	-4.156288	0.993164	-1.281596
H	-1.162501	-2.095022	-1.871418
H	-2.439341	-2.951366	-3.838371
O	5.075648	0.557051	-0.240707
C	5.980370	1.214374	0.723376
H	5.145576	-0.529740	-0.167880
C	7.416452	1.024313	0.263043
H	5.795468	0.788569	1.727137
H	5.691668	2.276450	0.732209
H	7.568112	1.447869	-0.741588
H	7.705025	-0.039515	0.246580
H	8.091492	1.543207	0.963423
C	2.610681	-3.020126	-0.270007
H	3.210347	-1.629249	1.297450

H	1.550557	-3.025458	0.030784
H	2.666543	-3.109757	-1.367042
H	3.094877	-3.904698	0.178027

SCF(BP86) = -1264.04765396
H 0K = -1263.560613
H 298K = -1263.527644
G 298K = -1263.631064
Solvent correction(toluene) = -0.03181694
BP86-D3 correction = -0.08286885
Lowest frequencies = 4.9526 cm-1, 16.1418 cm-1

IIc

60

P	1.653636	-0.044128	-0.027610
C	2.094173	-1.254578	-1.355671
C	2.411717	1.567121	-0.529840
Au	-0.672753	0.120579	0.316226
C	-2.767698	0.296650	0.683592
C	-3.525538	-0.790511	-0.042473
C	-3.252091	1.713324	0.370547
O	-4.738483	1.822286	0.735308
H	-4.796025	1.988378	1.698484
C	-3.234666	-2.220058	0.384962
H	-3.488918	-0.673486	-1.139633
H	-2.857698	0.121853	1.775271
C	2.565802	-0.583829	1.487205
H	-2.222043	-2.498555	0.051775
H	-3.943799	-2.934218	-0.064092
H	-3.279108	-2.317969	1.481501
C	3.538950	4.054373	-1.203352
C	3.948560	2.892077	-1.875706
C	3.389537	1.647221	-1.541994
C	1.996863	2.738782	0.140920
C	2.564334	3.976693	-0.193444
H	3.976457	5.022171	-1.467579
H	4.705856	2.950688	-2.663751
H	3.710228	0.744088	-2.070349
H	1.233821	2.678706	0.925610
H	2.242344	4.881868	0.331046
C	3.914833	-1.490840	3.781780
C	4.523246	-0.549951	2.935177
C	3.853828	-0.094150	1.788004
C	1.953682	-1.524302	2.343917
C	2.630678	-1.978272	3.485298
H	4.439157	-1.840666	4.676531
H	5.521383	-0.165700	3.167521
H	4.328570	0.644564	1.134595
H	0.947966	-1.895988	2.116549
H	2.152432	-2.706987	4.147293
C	2.718703	-3.039704	-3.437937
C	3.517336	-2.998899	-2.283908
C	3.210342	-2.108319	-1.242068
C	1.288211	-1.302952	-2.514410
C	1.605131	-2.190006	-3.553416
H	2.961156	-3.736261	-4.246586
H	4.382902	-3.662209	-2.190556
H	3.833741	-2.082177	-0.342968
H	0.415780	-0.644885	-2.600270
H	0.979798	-2.221934	-4.451267
O	-5.064469	-0.554601	0.282174
C	-6.020511	-0.929284	-0.780659
H	-5.096620	0.528209	0.500128
C	-7.435433	-0.716282	-0.269080
H	-5.819845	-1.988034	-1.006127
H	-5.801392	-0.323601	-1.678895
H	-7.630565	0.344971	-0.043848
H	-7.625881	-1.317274	0.633571
H	-8.150161	-1.028821	-1.048088
C	-3.180550	2.182256	-1.079465
H	-2.739291	2.438532	1.023551

H	-2.129290	2.204336	-1.407265
H	-3.734387	1.513383	-1.759976
H	-3.606678	3.192341	-1.177624

SCF(BP86) = -1264.04705922
H 0K = -1263.559785
H 298K = -1263.526916
G 298K = -1263.630321
Solvent correction(toluene) = -0.03178276
BP86-D3 correction = -0.08378777
Lowest frequencies = 3.7765 cm-1, 16.9135 cm-1

TS(II-III)b

60

P	-1.630645	0.069045	0.035154
C	-2.582282	-0.541425	-1.424703
C	-2.305759	-0.805611	1.516391
Au	0.685848	-0.248380	-0.198716
C	2.828396	-0.450311	-0.495955
C	3.558238	0.794026	0.019006
C	3.073602	-1.694478	0.205069
O	4.966646	-1.970845	0.009967
H	5.332326	-2.282143	0.863220
C	3.134745	2.081650	-0.691341
H	3.414401	0.892426	1.116354
H	2.924431	-0.553997	-1.592254
C	-2.072578	1.853613	0.227975
H	3.723116	2.949319	-0.354051
H	2.074226	2.295561	-0.477885
H	3.258900	1.977802	-1.781961
C	-3.291635	-2.078547	3.821075
C	-4.054444	-2.070398	2.642269
C	-3.567213	-1.434939	1.488568
C	-1.536809	-0.821162	2.700871
C	-2.033783	-1.452146	3.850523
H	-3.675027	-2.576799	4.716912
H	-5.032382	-2.561017	2.616678
H	-4.163522	-1.434158	0.570944
H	-0.552577	-0.338895	2.720108
H	-1.436701	-1.460010	4.767816
C	-2.716275	4.582409	0.432988
C	-3.276312	3.659324	1.329866
C	-2.959186	2.294163	1.230962
C	-1.506073	2.784447	-0.671081
C	-1.832998	4.143941	-0.569110
H	-2.965937	5.644844	0.514478
H	-3.963928	3.998802	2.110712
H	-3.398328	1.578070	1.932154
H	-0.817717	2.444191	-1.453325
H	-1.395686	4.861898	-1.269976
C	-4.025137	-1.553456	-3.614317
C	-4.477200	-0.403509	-2.946760
C	-3.760124	0.106494	-1.852629
C	-2.126591	-1.693519	-2.101165
C	-2.850601	-2.198348	-3.191063
H	-4.585157	-1.944502	-4.469416
H	-5.388996	0.102467	-3.278952
H	-4.110934	1.007925	-1.340467
H	-1.205091	-2.189572	-1.775803
H	-2.493452	-3.090518	-3.714745
O	5.001174	0.548017	-0.208902
C	5.901445	1.401038	0.549660
H	5.186822	-0.959482	-0.065678
C	7.334382	0.997305	0.230649
H	5.677606	1.299438	1.632084
H	5.723700	2.453260	0.265472
H	7.537151	1.092512	-0.847624
H	7.544505	-0.041768	0.535557
H	8.034027	1.653871	0.772822
C	2.604022	-3.023218	-0.332622
H	3.110024	-1.620593	1.302808

H	1.555607	-3.192257	-0.031437
H	2.662460	-3.051551	-1.431500
H	3.202241	-3.850225	0.078931

SCF(BP86) = -1264.04289215
H 0K = -1263.556946
H 298K = -1263.523922
G 298K = -1263.627815
Solvent correction(toluene) = -0.03149553
BP86-D3 correction = -0.08329084
Lowest frequencies = -173.2852 cm-1, 6.9830 cm-1

TS(II-III)c

60

P	1.655787	-0.047940	-0.027020
C	2.448564	1.532223	-0.567137
C	2.532283	-0.569914	1.512127
Au	-0.660456	0.185832	0.299324
C	-2.791935	0.308500	0.664882
C	-3.580255	-0.795025	-0.045231
C	-3.059592	1.684317	0.281832
O	-4.912517	1.979784	0.735869
H	-4.945071	2.067963	1.710903
C	-3.210135	-2.199556	0.435967
H	-3.452649	-0.724206	-1.144148
H	-2.827630	0.172499	1.761945
C	2.054072	-1.307844	-1.318131
H	-2.161679	-2.422479	0.176019
H	-3.841552	-2.970357	-0.033253
H	-3.322232	-2.275839	1.530382
C	3.838056	-1.451509	3.839609
C	4.472731	-0.538882	2.981489
C	3.824995	-0.095245	1.817226
C	1.893513	-1.482631	2.379410
C	2.549324	-1.923787	3.537847
H	4.345637	-1.791461	4.747652
H	5.474562	-0.167113	3.217864
H	4.320108	0.621285	1.154409
H	0.885140	-1.844260	2.147509
H	2.051214	-2.630745	4.208570
C	2.631102	-3.169741	-3.343841
C	3.441486	-3.103487	-2.199084
C	3.158293	-2.174519	-1.184668
C	1.236321	-1.380657	-2.467180
C	1.529731	-2.307048	-3.478334
H	2.854615	-3.897063	-4.130481
H	4.297038	-3.777365	-2.091235
H	3.789813	-2.128917	-0.292059
H	0.373130	-0.712569	-2.567673
H	0.895011	-2.359816	-4.368431
C	3.634424	3.971267	-1.307010
C	3.978177	2.789701	-1.982455
C	3.389437	1.567895	-1.616033
C	2.099503	2.723007	0.107844
C	2.696598	3.936943	-0.260224
H	4.094900	4.920783	-1.597037
H	4.707147	2.814758	-2.798390
H	3.659285	0.649379	-2.146025
H	1.366381	2.697280	0.922318
H	2.426805	4.857297	0.267131
O	-5.006077	-0.519392	0.243758
C	-5.950313	-1.133636	-0.675904
H	-5.149575	0.990335	0.545918
C	-7.358475	-0.719226	-0.273041
H	-5.835955	-2.231546	-0.626459
H	-5.712210	-0.811318	-1.710261
H	-7.492265	0.373320	-0.341025
H	-7.584483	-1.042041	0.755766
H	-8.090789	-1.190815	-0.948234
C	-3.165482	2.152977	-1.149233
H	-2.668471	2.443575	0.972352

H	-2.147343	2.276377	-1.557819
H	-3.703186	1.436389	-1.788613
H	-3.672268	3.127163	-1.200254

SCF(BP86) = -1264.04332586
H 0K = -1263.556985
H 298K = -1263.524942
G 298K = -1263.624273
Solvent correction(toluene) = -0.03108530
BP86-D3 correction = -0.08390572
Lowest frequencies = -166.7523 cm-1, -5.0508 cm-1

IIIb

60

P	1.645164	-0.081919	0.053515
C	2.688569	1.353670	-0.448853
C	2.104753	-0.510756	1.786005
Au	-0.649411	0.383514	-0.166818
C	-2.866494	0.398794	-0.649978
C	-3.562435	-0.798121	-0.005436
C	-2.663119	1.603980	0.022765
O	-5.331828	2.192585	0.543421
H	-5.862488	2.215728	1.359002
C	-3.100339	-2.136298	-0.597286
H	-3.381830	-0.783533	1.092809
H	-2.897149	0.417902	-1.750578
C	2.118522	-1.507001	-1.017503
H	-3.609643	-2.989708	-0.123635
H	-2.015560	-2.269622	-0.441213
H	-3.303310	-2.167069	-1.680939
C	2.791091	-1.244617	4.408491
C	3.687373	-0.490688	3.633649
C	3.349947	-0.120768	2.321855
C	1.202121	-1.264111	2.567728
C	1.549753	-1.632219	3.875280
H	3.057380	-1.526613	5.431785
H	4.652189	-0.185201	4.049887
H	4.047821	0.472418	1.722892
H	0.231400	-1.559993	2.153665
H	0.848822	-2.215386	4.480388
C	2.840325	-3.628154	-2.710859
C	3.358739	-3.562778	-1.407620
C	3.002094	-2.504221	-0.556351
C	1.592925	-1.575918	-2.327078
C	1.959301	-2.634017	-3.170949
H	3.119750	-4.456595	-3.369011
H	4.042799	-4.337603	-1.048198
H	3.405922	-2.458008	0.459609
H	0.903125	-0.803108	-2.685820
H	1.552688	-2.685086	-4.185642
C	4.265271	3.572151	-1.140803
C	4.601824	2.295787	-1.618635
C	3.817605	1.182097	-1.275506
C	2.348164	2.638605	0.028727
C	3.139987	3.742925	-0.315464
H	4.877855	4.436854	-1.413836
H	5.476626	2.162257	-2.262342
H	4.081286	0.189259	-1.652291
H	1.470161	2.773581	0.670736
H	2.875657	4.737763	0.055958
O	-4.965449	-0.546293	-0.255285
C	-5.874108	-1.418697	0.460729
H	-5.390602	1.260987	0.227693
C	-7.295134	-0.960992	0.159826
H	-5.652001	-1.367843	1.548185
H	-5.729831	-2.466242	0.135949
H	-7.507423	-1.030344	-0.918675
H	-7.455586	0.082929	0.476226
H	-8.016168	-1.598254	0.697050
C	-2.481802	2.938840	-0.638148

H	-2.847190	1.619743	1.104935
H	-1.728468	3.560815	-0.128335
H	-2.233616	2.855838	-1.707568
H	-3.458011	3.450047	-0.535424

SCF(BP86) = -1264.04985855
H 0K = -1263.565302
H 298K = -1263.530164
G 298K = -1263.639059
Solvent correction(toluene) = -0.03136426
BP86-D3 correction = -0.08245070
Lowest frequencies = 6.0644 cm-1, 17.1101 cm-1

IIIc

60

P	1.676587	-0.055922	-0.029926
C	2.548186	1.479252	-0.565234
C	2.494085	-0.639553	1.514651
Au	-0.616239	0.351299	0.295791
C	-2.838004	0.252397	0.782070
C	-3.555140	-0.850553	0.007988
C	-2.638910	1.555708	0.320780
O	-5.631548	2.134318	0.566527
H	-6.177227	2.341116	1.345450
C	-3.102024	-2.251841	0.436187
H	-3.386649	-0.717200	-1.081504
H	-2.790483	0.093031	1.870134
C	1.957472	-1.333602	-1.328746
H	-2.030530	-2.398795	0.212452
H	-3.663547	-3.032629	-0.100243
H	-3.259794	-2.393631	1.518467
C	3.722320	-1.605750	3.847693
C	4.420128	-0.732365	2.997726
C	3.811426	-0.245698	1.829765
C	1.791688	-1.514137	2.372136
C	2.409334	-1.997378	3.534476
H	4.199892	-1.978172	4.759154
H	5.440843	-0.424011	3.243685
H	4.355176	0.440831	1.173484
H	0.765533	-1.814853	2.131157
H	1.863097	-2.673586	4.199169
C	2.375353	-3.224391	-3.362990
C	3.158907	-3.261653	-2.198508
C	2.955414	-2.318308	-1.178224
C	1.165617	-1.300009	-2.497993
C	1.380581	-2.242507	-3.513532
H	2.536581	-3.963469	-4.153765
H	3.931411	-4.027494	-2.079501
H	3.565856	-2.352453	-0.270678
H	0.386878	-0.537288	-2.613907
H	0.767914	-2.213960	-4.419853
C	3.873150	3.841454	-1.306060
C	4.135513	2.645093	-1.992143
C	3.476136	1.459996	-1.626242
C	2.281239	2.683991	0.122715
C	2.947940	3.860169	-0.247574
H	4.387452	4.762394	-1.597403
H	4.854853	2.629972	-2.816640
H	3.681032	0.529982	-2.165041
H	1.559635	2.700618	0.947606
H	2.741459	4.792503	0.286866
O	-4.955567	-0.634956	0.294266
C	-5.854683	-1.201414	-0.694233
H	-5.492558	1.160191	0.605995
C	-7.274364	-0.786826	-0.333875
H	-5.752626	-2.303855	-0.703709
H	-5.572805	-0.824356	-1.699609
H	-7.361688	0.312027	-0.323037
H	-7.559883	-1.179444	0.655370
H	-7.980790	-1.186565	-1.079409
C	-2.988071	2.103571	-1.030706
H	-2.374114	2.305619	1.080125

H	-2.274718	2.879987	-1.349454
H	-3.066224	1.333085	-1.812268
H	-3.982758	2.577866	-0.908811

SCF(BP86) = -1264.05012461
H 0K = -1263.564919
H 298K = -1263.530056
G 298K = -1263.637984
Solvent correction(toluene) = -0.03158397
BP86-D3 correction = -0.08239995
Lowest frequencies = 10.6717 cm-1, 14.6238 cm-1

IIIb (no water)

57

P	1.445084	-0.118429	0.027142
C	2.590841	1.290728	-0.293271
C	1.907642	-0.839848	1.658011
Au	-0.803919	0.565785	-0.028473
C	-3.056500	0.730921	-0.469623
C	-3.787953	-0.504047	0.052414
C	-2.722323	1.830158	0.317244
C	-3.311324	-1.818786	-0.578979
H	-3.675534	-0.545183	1.158526
H	-3.097399	0.864939	-1.561885
C	1.763624	-1.396119	-1.263348
H	-3.930685	-2.660128	-0.228929
H	-2.261336	-2.036207	-0.312395
H	-3.391944	-1.767535	-1.677482
C	2.594079	-2.015902	4.113829
C	3.532594	-1.229613	3.425980
C	3.195433	-0.638832	2.197689
C	0.963077	-1.626415	2.352733
C	1.310569	-2.215268	3.576863
H	2.860798	-2.470374	5.072916
H	4.530608	-1.071046	3.845791
H	3.926872	-0.021085	1.667574
H	-0.039687	-1.776613	1.936656
H	0.577232	-2.823767	4.114837
C	2.254379	-3.287026	-3.280377
C	2.811826	-3.462109	-2.003626
C	2.570610	-2.519722	-0.990742
C	1.198513	-1.222749	-2.546504
C	1.449782	-2.166772	-3.552219
H	2.443312	-4.026177	-4.064900
H	3.436150	-4.335270	-1.790946
H	3.004181	-2.660970	0.003956
H	0.567576	-0.351731	-2.758409
H	1.012474	-2.030841	-4.546083
C	4.327869	3.458135	-0.706126
C	4.529502	2.255203	-1.400990
C	3.664583	1.167061	-1.198201
C	2.386175	2.502355	0.403461
C	3.257835	3.580651	0.197712
H	5.003043	4.303586	-0.869977
H	5.361551	2.159328	-2.105213
H	3.823463	0.231682	-1.743136
H	1.552404	2.599543	1.108364
H	3.099409	4.518289	0.739258
O	-5.151311	-0.227639	-0.292689
C	-6.114837	-0.772444	0.632855
C	-7.505037	-0.412170	0.128756
H	-5.937129	-0.351968	1.646165
H	-5.997177	-1.873619	0.703092
H	-7.681806	-0.842075	-0.869884
H	-7.626814	0.680990	0.065081
H	-8.269130	-0.807980	0.817603
C	-2.483326	3.223991	-0.204979
H	-2.871819	1.737219	1.404240
H	-1.659125	3.730789	0.322474
H	-2.284916	3.237940	-1.287893
H	-3.396522	3.820516	-0.017062

SCF(BP86) = -1187.61810967
H 0K = -1187.157518
H 298K = -1187.125484
G 298K = -1187.228127
Solvent correction(toluene) = -0.03127179
BP86-D3 correction = -0.07479877
Lowest frequencies = 6.4199 cm-1, 16.4944 cm-1

IIIc (no water)

57

P	-1.467570	-0.076348	0.067401
C	-2.411438	1.457261	0.466933
C	-2.321018	-0.893811	-1.345721
Au	0.769441	0.430747	-0.446391
C	2.975685	0.333564	-1.038429
C	3.770595	-0.576586	-0.102486
C	2.718919	1.682941	-0.800657
C	3.521946	-2.059660	-0.415106
H	3.483660	-0.373398	0.955318
H	2.878574	-0.023137	-2.074924
C	-1.589326	-1.200761	1.522588
H	2.453082	-2.308342	-0.289594
H	4.094467	-2.712925	0.261102
H	3.818973	-2.288789	-1.452502
C	-3.599505	-2.213339	-3.468950
C	-4.307683	-1.279427	-2.695365
C	-3.674012	-0.616196	-1.632290
C	-1.608482	-1.829846	-2.126634
C	-2.251125	-2.489389	-3.183958
H	-4.097039	-2.723778	-4.299266
H	-5.356344	-1.061622	-2.919616
H	-4.226606	0.116269	-1.035835
H	-0.555449	-2.041409	-1.908031
H	-1.697306	-3.213156	-3.789640
C	-1.768734	-2.854028	3.785491
C	-2.611450	-3.077212	2.684838
C	-2.527182	-2.253399	1.550677
C	-0.737899	-0.980046	2.627778
C	-0.833904	-1.804456	3.757788
H	-1.836718	-3.500939	4.665500
H	-3.337154	-3.895968	2.704654
H	-3.183063	-2.432539	0.693280
H	-0.005496	-0.164844	2.604761
H	-0.174872	-1.631459	4.614117
C	-3.852332	3.807618	0.999556
C	-3.980836	2.697956	1.849759
C	-3.262895	1.519154	1.588661
C	-2.278900	2.575362	-0.386595
C	-3.003278	3.745346	-0.119440
H	-4.412043	4.724270	1.209276
H	-4.641011	2.745901	2.721149
H	-3.363945	0.657000	2.254708
H	-1.617584	2.528667	-1.259504
H	-2.901582	4.610114	-0.782275
O	5.125269	-0.167655	-0.313178
C	6.055102	-0.662228	0.672141
C	7.407643	-0.017080	0.402595
H	6.134144	-1.766350	0.614037
H	5.685445	-0.406882	1.689749
H	7.342841	1.080101	0.479264
H	7.767622	-0.275649	-0.605935
H	8.148412	-0.374601	1.136387
C	3.120743	2.463607	0.419686
H	2.380760	2.281733	-1.659603
H	2.359765	3.213768	0.689049
H	3.332565	1.826745	1.290672

H   4.047629   3.016566   0.175854

SCF(BP86) = -1187.61704285
H 0K = -1187.156353
H 298K = -1187.124336
G 298K = -1187.227291
Solvent correction(toluene) = -0.03121024
BP86-D3 correction = -0.07530524
Lowest frequencies = 5.2268 cm-1, 15.4945 cm-1

(i) Single EtOH nucleophile, Pathway (ii), *syn* attack

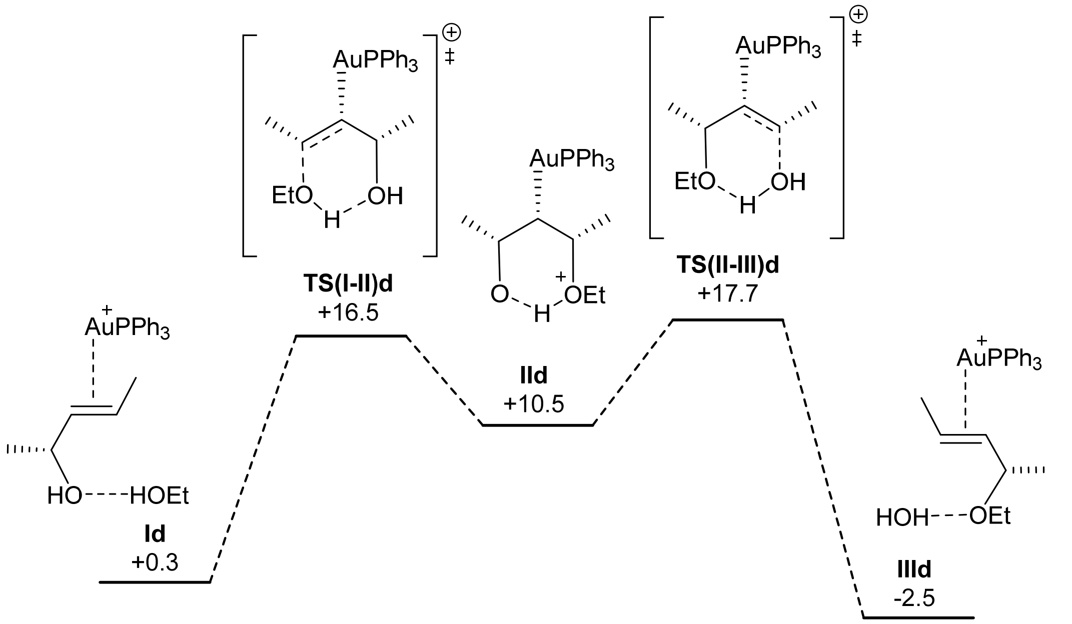

Figure S5. Computed free energy profile (BP86-D3(toluene)) for direct etherification with a single EtOH nucleophile at **Id** *via* Pathway (ii) (*syn* attack) leading to (*S*,*E*)-**5ko**. All energies are in kcal/mol and are quoted relative to **Ia** set to 0.0 kcal/mol.

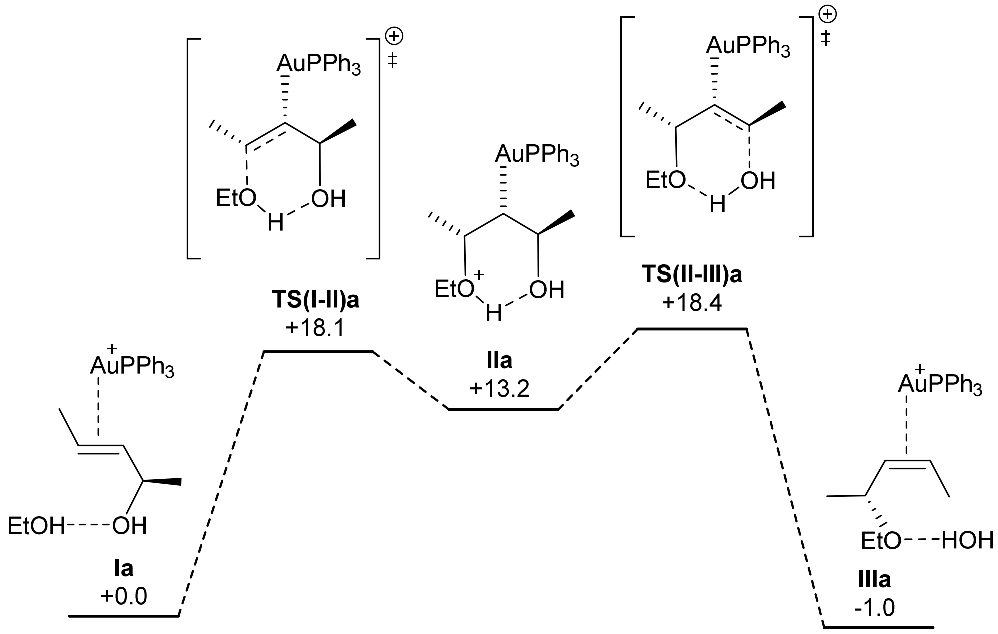

Figure S6. Computed free energy profile (BP86-D3(toluene)) for direct etherification with a single EtOH nucleophile at **Ia** *via* Pathway (ii) (*syn* attack) leading to (*R*,*Z*)-**5ko**. All energies are in kcal/mol and are quoted relative to **Ia** set to 0.0 kcal/mol.

Table S4. Energies associated with direct etherification with a single EtOH nucleophile *via* Pathway (ii) (*syn* attack). Energies are reported as ΔE (gas phase SCF energies), ΔG (free energies at 298.15 K and 1atm), ΔG_{disp} (including a correction for dispersion effects using Grimme's D3 parameter set) and $\Delta G_{toluene+disp}$ (including an additional correction for toluene solvent *via* the PCM approach). All energies relative to **Ia**.

Isomer	ΔE	ΔG	ΔG_{disp}	$\Delta G_{toluene+disp}$
Id	+1.0	+0.6	+0.4	+0.3
Ia	+0.0	+0.0	+0.0	+0.0
TS(I-II)d	+17.7	+17.9	+16.5	+16.5
TS(I-II)a	+17.8	+18.3	+18.0	+18.1
IId	+11.0	+13.0	+11.8	+10.5
IIa	+13.1	+15.4	+14.4	+13.2
TS(II-III)d	+18.9	+20.0	+18.3	+17.7
TS(II-III)a	+20.3	+20.7	+18.1	+18.4
IIId	-1.6	-0.8	-2.9	-2.5
IIIa	+1.5	+1.7	-1.2	-1.0

Id

60

P	1.303485	0.169640	0.047287
C	1.416913	1.956560	-0.386628
C	1.620428	0.009335	1.859961
Au	-0.795122	-0.715446	-0.513871
C	-2.674559	-2.076817	-0.610923
C	-2.857971	-1.040865	-1.522360
C	-3.291667	-2.156453	0.773410
O	-3.599978	-0.826893	1.229378
H	-3.978459	-0.905237	2.125277
C	-2.631859	-1.172208	-3.007729
H	-3.373100	-0.129197	-1.183969
H	-2.302286	-3.041303	-0.987394
C	2.679063	-0.705422	-0.813312
H	-2.143752	-0.277247	-3.427038
H	-3.619568	-1.250974	-3.500963
H	-2.045905	-2.066607	-3.272724
C	2.053091	-0.297793	4.619335
C	2.013980	0.985726	4.053039
C	1.798613	1.145436	2.673798
C	1.654721	-1.283242	2.430863
C	1.875953	-1.432106	3.807186
H	2.223771	-0.416820	5.693748
H	2.155227	1.869505	4.682797
H	1.772855	2.147338	2.235073
H	1.525670	-2.169721	1.799513
H	1.911845	-2.434195	4.245887
C	4.772051	-1.954013	-2.211183
C	4.975411	-1.507543	-0.895317
C	3.933313	-0.882541	-0.191650
C	2.475219	-1.158857	-2.134440
C	3.522780	-1.778846	-2.830500
H	5.586568	-2.443636	-2.753795
H	5.946947	-1.646712	-0.411216
H	4.093823	-0.543449	0.836439
H	1.498604	-1.026537	-2.614028
H	3.361822	-2.130648	-3.854160
C	1.582945	4.692445	-0.982584
C	2.741798	3.898504	-1.010533
C	2.664724	2.529648	-0.713801
C	0.247772	2.748965	-0.363884
C	0.342399	4.118342	-0.659915
H	1.646899	5.759116	-1.219416
H	3.708157	4.342611	-1.268355
H	3.567402	1.911550	-0.746733
H	-0.730511	2.309319	-0.126230
H	-0.560996	4.735835	-0.646015
O	-3.015848	1.746301	0.065678
C	-3.950368	2.796477	0.391424
H	-3.214717	0.973956	0.641202
C	-5.402744	2.395201	0.132753
H	-3.667957	3.651112	-0.246288
H	-3.818979	3.117406	1.445213
H	-5.691694	1.525696	0.749145
H	-5.555018	2.132004	-0.927204
H	-6.086797	3.224185	0.382496
C	-2.421927	-2.918657	1.781108
H	-4.242630	-2.720009	0.629521

H	-2.196268	-3.934692	1.415721
H	-1.473190	-2.384085	1.956410
H	-2.949250	-3.025870	2.744440

SCF(BP86) = -1264.05721366
H 0K = -1263.571127
H 298K = -1263.536385
G 298K = -1263.643916
Solvent correction(toluene) = -0.03147087
BP86-D3 correction = -0.08441153
Lowest frequencies = 8.0642 cm-1, 20.2802 cm-1

Ia

60

P	-1.269153	-0.019877	0.087412
C	-2.443999	-1.433555	-0.070749
C	-1.471086	0.676065	1.784221
Au	0.952280	-0.658071	-0.321711
C	2.966188	-1.761395	-0.043751
C	2.973159	-1.242070	-1.333718
C	3.721984	-1.169643	1.133937
O	4.104806	0.180849	0.803594
H	4.730783	0.482712	1.488931
C	2.639651	-2.029190	-2.576934
H	3.471184	-0.274252	-1.478449
H	2.642714	-2.801243	0.106152
C	-1.833104	1.270391	-1.106322
H	3.585276	-2.296479	-3.085931
H	2.052616	-1.433929	-3.295005
H	2.095738	-2.960113	-2.352811
C	-1.750109	1.791630	4.343766
C	-2.691410	0.858875	3.879963
C	-2.557573	0.297797	2.599610
C	-0.517506	1.610682	2.248778
C	-0.668495	2.166881	3.528051
H	-1.858100	2.225422	5.342864
H	-3.533519	0.564040	4.513693
H	-3.292149	-0.429698	2.241361
H	0.329541	1.900560	1.613395
H	0.064983	2.894362	3.890277
C	-2.720169	3.167330	-2.979313
C	-2.992657	3.347976	-1.614097
C	-2.551758	2.402751	-0.672984
C	-1.555018	1.091912	-2.479298
C	-2.002508	2.038340	-3.411694
H	-3.064622	3.907410	-3.708248
H	-3.550149	4.226925	-1.275948
H	-2.762700	2.548322	0.390733
H	-0.995144	0.212386	-2.817462
H	-1.787827	1.896847	-4.475511
C	-4.224638	-3.602027	-0.223581
C	-4.639058	-2.323464	-0.630749
C	-3.753988	-1.236054	-0.555556
C	-2.027804	-2.720230	0.333251
C	-2.919984	-3.799754	0.259886
H	-4.917113	-4.447019	-0.287931
H	-5.653397	-2.169351	-1.011495
H	-4.078992	-0.242398	-0.879332
H	-1.006370	-2.873177	0.700307
H	-2.594363	-4.796650	0.572778
O	2.030660	1.974104	0.118150
C	2.446350	2.962313	-0.849801
H	2.828562	1.476444	0.421486
C	3.295103	4.069795	-0.226255
H	1.513420	3.376653	-1.266306
H	2.993660	2.479529	-1.685870
H	4.243539	3.671787	0.175437
H	2.751291	4.563830	0.595366
H	3.549298	4.833848	-0.980915
C	4.960523	-2.032021	1.441975
H	3.056726	-1.168446	2.022821

H	4.672290	-3.066756	1.689533
H	5.644964	-2.050221	0.578585
H	5.498772	-1.625239	2.315714

SCF(BP86) = -1264.05887825
H 0K = -1263.572427
H 298K = -1263.537835
G 298K = -1263.644941
Solvent correction(toluene) = -0.03132121
BP86-D3 correction = -0.08400405
Lowest frequencies = 10.0318 cm-1, 18.4943 cm-1

TS(I-II)d

60

P	-1.385585	-0.101068	0.051670
C	-2.011313	-1.448316	-1.048414
C	-1.586768	-0.712612	1.783783
Au	0.823486	0.494635	-0.445356
C	2.858821	1.160238	-0.950210
C	3.684597	0.043909	-1.313980
C	3.432025	2.059968	0.145179
O	3.392903	1.233583	1.357623
H	3.891558	1.691095	2.062063
C	3.426305	-0.786781	-2.529918
H	4.725977	0.040681	-0.963024
H	2.484573	1.693863	-1.846524
C	-2.551376	1.319401	-0.139950
H	3.716330	-1.838465	-2.373815
H	4.065116	-0.407745	-3.351192
H	2.372123	-0.746306	-2.843399
C	-1.892752	-1.547057	4.450889
C	-2.623271	-2.176409	3.430139
C	-2.474460	-1.762980	2.096211
C	-0.846857	-0.086617	2.810468
C	-1.005776	-0.501941	4.140543
H	-2.011299	-1.873448	5.488830
H	-3.311528	-2.993018	3.669575
H	-3.042158	-2.259116	1.302835
H	-0.148405	0.721669	2.565674
H	-0.433106	-0.012847	4.934880
C	-4.316507	3.473324	-0.524555
C	-4.529971	2.569904	0.529064
C	-3.651707	1.491801	0.725209
C	-2.334423	2.233207	-1.194282
C	-3.219611	3.303836	-1.386619
H	-5.002167	4.313636	-0.671389
H	-5.381049	2.703458	1.204084
H	-3.818567	0.793301	1.551077
H	-1.472440	2.105349	-1.859110
H	-3.048662	4.009930	-2.205205
C	-2.902201	-3.555053	-2.682811
C	-3.748644	-2.467605	-2.415143
C	-3.308837	-1.412800	-1.598251
C	-1.158528	-2.539899	-1.324041
C	-1.607980	-3.591499	-2.135269
H	-3.248599	-4.373343	-3.321729
H	-4.755457	-2.435928	-2.843078
H	-3.970718	-0.565439	-1.394735
H	-0.147024	-2.563271	-0.902456
H	-0.945927	-4.437402	-2.345671
O	3.299435	-1.227930	0.246023
C	4.247444	-2.268528	0.633478
H	3.330820	-0.472059	0.906547
C	5.549410	-1.740966	1.229542
H	4.427795	-2.857265	-0.282062
H	3.720773	-2.926238	1.348436
H	5.362245	-1.136698	2.133159
H	6.121733	-1.130656	0.511693
H	6.188476	-2.589432	1.525625
C	2.669876	3.367404	0.346588
H	4.498846	2.286955	-0.072359

H	2.733637	3.994087	-0.559552
H	1.606773	3.166091	0.562054
H	3.095040	3.949622	1.181801

SCF(BP86) = -1264.030615
H 0K = -1263.545339
H 298K = -1263.511897
G 298K = -1263.616383
Solvent correction(toluene) = -0.03131495
BP86-D3 correction = -0.08628731
Lowest frequencies = -190.7641 cm-1, 6.2883 cm-1

TS(I-II)a

60

P	-1.414667	0.037417	0.029182
C	-2.544338	-0.721916	-1.220499
C	-2.044486	-0.478545	1.688089
Au	0.816285	-0.595573	-0.297480
C	2.853783	-1.355691	-0.570705
C	3.687104	-0.376651	-1.213948
C	3.386286	-1.922882	0.752689
O	3.528854	-0.752388	1.619502
H	3.995567	-1.013954	2.435317
C	3.463753	0.044906	-2.632579
H	4.719788	-0.255764	-0.856069
H	2.508094	-2.125419	-1.288683
C	-1.673115	1.864238	-0.081049
H	4.111889	-0.575146	-3.281902
H	3.763420	1.091190	-2.801979
H	2.416060	-0.086828	-2.942765
C	-2.951150	-1.198453	4.249826
C	-3.803525	-1.326677	3.141523
C	-3.355770	-0.967671	1.859645
C	-1.186230	-0.356916	2.802814
C	-1.643389	-0.712251	4.080019
H	-3.303739	-1.482134	5.246335
H	-4.820655	-1.709300	3.271284
H	-4.021520	-1.073821	0.997538
H	-0.163484	0.013201	2.667312
H	-0.976454	-0.616121	4.942588
C	-2.067278	4.634861	-0.350877
C	-2.740651	3.924522	0.655698
C	-2.548056	2.540110	0.794069
C	-0.990099	2.581908	-1.087051
C	-1.193237	3.962716	-1.222665
H	-2.220669	5.713555	-0.454847
H	-3.419228	4.446638	1.337456
H	-3.073375	1.990337	1.581122
H	-0.301956	2.058560	-1.760363
H	-0.665730	4.515434	-2.006503
C	-4.253286	-1.960055	-3.077446
C	-4.494772	-0.636825	-2.674529
C	-3.644443	-0.013630	-1.746633
C	-2.299446	-2.050276	-1.631941
C	-3.156577	-2.666738	-2.554834
H	-4.916993	-2.440195	-3.803234
H	-5.346114	-0.084001	-3.083718
H	-3.833231	1.019565	-1.439106
H	-1.438478	-2.596842	-1.230219
H	-2.964047	-3.696592	-2.871365
O	3.293326	1.282736	-0.118252
C	4.348204	2.290185	-0.047453
H	3.324825	0.735430	0.723187
C	4.040304	3.321194	1.033716
H	4.376162	2.756145	-1.045455
H	5.322137	1.795445	0.134902
H	4.016448	2.859787	2.035217
H	3.070522	3.808346	0.847118
H	4.824133	4.097098	1.039720
C	4.711548	-2.689029	0.652294
H	2.617816	-2.591752	1.185149

H	4.592620	-3.575287	0.007012
H	5.518624	-2.063254	0.235003
H	5.036848	-3.050693	1.644106

SCF(BP86) = -1264.03051292
H 0K = -1263.544744
H 298K = -1263.511302
G 298K = -1263.615831
Solvent correction(toluene) = -0.03110171
BP86-D3 correction = -0.08442209
Lowest frequencies = -178.4316 cm-1, 5.2084 cm-1

IId

60

P	1.462748	-0.089035	-0.064174
C	2.498569	-0.578886	1.388028
C	1.667009	-1.435910	-1.317737
Au	-0.797441	0.315148	0.493385
C	-2.811475	0.726723	1.056864
C	-3.728707	-0.495518	1.068466
C	-3.446195	1.869792	0.260009
O	-3.492628	1.364176	-1.157678
H	-4.124865	1.904917	-1.673462
C	-3.247404	-1.682555	1.882322
H	-4.771962	-0.228080	1.337771
H	-2.733891	1.061603	2.109446
C	2.293652	1.400740	-0.784473
H	-3.942459	-2.536822	1.837742
H	-3.176859	-1.374740	2.939248
H	-2.248994	-2.001268	1.544190
C	1.921257	-3.426137	-3.290710
C	2.840828	-3.364552	-2.231575
C	2.718932	-2.371671	-1.245575
C	0.738069	-1.506021	-2.379261
C	0.871108	-2.495043	-3.364783
H	2.020317	-4.201475	-4.056907
H	3.657436	-4.090716	-2.169597
H	3.437237	-2.329227	-0.420954
H	-0.085555	-0.784482	-2.430287
H	0.152076	-2.542421	-4.188962
C	3.536869	3.717134	-1.786564
C	3.747541	2.478976	-2.413591
C	3.130202	1.319213	-1.915822
C	2.078522	2.648902	-0.159122
C	2.703738	3.800861	-0.657523
H	4.019732	4.618023	-2.177951
H	4.395176	2.411415	-3.293418
H	3.297000	0.356312	-2.408374
H	1.426691	2.714810	0.719691
H	2.537507	4.765111	-0.166791
C	4.023576	-1.402462	3.603693
C	4.614869	-0.627231	2.592817
C	3.857676	-0.213852	1.484959
C	1.905665	-1.351656	2.409578
C	2.669244	-1.764862	3.511284
H	4.616683	-1.719150	4.467325
H	5.668241	-0.339107	2.666226
H	4.320059	0.397829	0.703785
H	0.845860	-1.622039	2.340927
H	2.204807	-2.362783	4.301791
O	-3.828745	-0.929313	-0.410945
C	-5.014682	-1.724563	-0.816709
H	-3.745597	0.031479	-0.945440
C	-6.268925	-0.886417	-1.022220
H	-5.141085	-2.488326	-0.035483
H	-4.692284	-2.226603	-1.741696
H	-6.137177	-0.140055	-1.822939
H	-6.589714	-0.374210	-0.100939
H	-7.091198	-1.553540	-1.330524
C	-2.704413	3.196736	0.297628
H	-4.507439	2.021376	0.558922

H	-2.685039	3.581080	1.331385
H	-1.667149	3.065836	-0.050077
H	-3.203107	3.954710	-0.330401

SCF(BP86) = -1264.04137304
H 0K = -1263.554304
H 298K = -1263.521646
G 298K = -1263.624168
Solvent correction(toluene) = -0.03332110
BP86-D3 correction = -0.08599679
Lowest frequencies = 4.4103 cm-1, 18.1740 cm-1

IIa

60

P	1.519915	-0.038929	0.058155
C	2.424714	-0.864819	-1.328694
C	2.432548	1.535619	0.395399
Au	-0.759690	0.331950	-0.440750
C	-2.781730	0.759217	-0.958971
C	-3.687905	-0.468387	-1.012452
C	-3.363594	1.884424	-0.082435
O	-3.448476	1.289895	1.293518
H	-4.091846	1.797063	1.828001
C	-3.214153	-1.593782	-1.914142
H	-4.746112	-0.219907	-1.225982
H	-2.714218	1.147298	-1.993546
C	1.797922	-1.106906	1.544703
H	-3.211681	-1.226341	-2.954300
H	-3.880058	-2.471554	-1.883172
H	-2.187834	-1.895358	-1.651776
C	3.755633	3.947629	0.983520
C	4.395318	2.965374	0.210822
C	3.739190	1.758946	-0.084322
C	1.788926	2.528337	1.166543
C	2.452845	3.727573	1.462674
H	4.269918	4.887089	1.209049
H	5.408442	3.136212	-0.166534
H	4.240099	0.997825	-0.690580
H	0.769858	2.358637	1.532943
H	1.950879	4.493711	2.062100
C	2.182742	-2.811796	3.749842
C	3.023516	-1.702935	3.563593
C	2.835944	-0.849953	2.463490
C	0.948791	-2.217991	1.739504
C	1.146327	-3.069454	2.836223
H	2.332374	-3.473796	4.608548
H	3.829328	-1.498271	4.275510
H	3.491832	0.015013	2.323833
H	0.136252	-2.412724	1.030183
H	0.488543	-3.932522	2.980897
C	3.779538	-2.037172	-3.497413
C	4.128078	-2.382239	-2.181564
C	3.455094	-1.798971	-1.095645
C	2.071182	-0.525689	-2.653129
C	2.751969	-1.107913	-3.732483
H	4.305634	-2.495710	-4.340593
H	4.925455	-3.108933	-1.996702
H	3.726827	-2.074691	-0.071824
H	1.262999	0.192326	-2.834389
H	2.475729	-0.840528	-4.757344
O	-3.715506	-0.982215	0.447356
C	-4.826233	-1.875305	0.860515
H	-3.665783	-0.047608	1.019910
C	-6.151844	-1.157878	1.073267
H	-4.453941	-2.342311	1.785241
H	-4.886191	-2.650340	0.082622
H	-6.538720	-0.699633	0.149062
H	-6.080531	-0.385428	1.856478
H	-6.898951	-1.896260	1.409610
C	-4.729374	2.458945	-0.474683
H	-2.630211	2.705628	-0.017194

H	-4.661164	2.924216	-1.471792
H	-5.523517	1.694469	-0.509950
H	-5.047426	3.251645	0.226641

SCF(BP86) = -1264.03797025
H 0K = -1263.550631
H 298K = -1263.518004
G 298K = -1263.620352
Solvent correction(toluene) = -0.03318025
BP86-D3 correction = -0.08566299
Lowest frequencies = 4.5670 cm-1, 17.7267 cm-1

TS(II-III)d

60

P	1.430355	0.086209	0.069874
C	2.606091	-0.958819	-0.900371
C	1.868529	1.844383	-0.294900
Au	-0.801934	-0.392457	-0.458542
C	-2.871022	-0.795889	-1.067444
C	-3.727239	0.474217	-1.050067
C	-3.422862	-1.903957	-0.332121
O	-3.003943	-1.322731	1.544145
H	-3.682346	-1.630317	2.183937
C	-3.217969	1.566157	-1.989358
H	-4.780779	0.227367	-1.304798
H	-2.549068	-1.117165	-2.078261
C	1.830698	-0.185848	1.852434
H	-3.822424	2.484342	-1.915904
H	-3.274517	1.218414	-3.035205
H	-2.168022	1.813532	-1.758215
C	2.481506	4.545326	-0.776597
C	3.388827	3.541727	-1.151127
C	3.087865	2.190617	-0.911882
C	0.954009	2.854860	0.075638
C	1.265148	4.201306	-0.161683
H	2.719497	5.596456	-0.967102
H	4.335019	3.807483	-1.632696
H	3.796320	1.411119	-1.208609
H	0.002949	2.585814	0.549450
H	0.555409	4.982511	0.128204
C	2.453842	-0.698310	4.545069
C	3.068026	0.371445	3.873744
C	2.760329	0.631632	2.528675
C	1.207704	-1.255047	2.531514
C	1.524613	-1.510934	3.873636
H	2.695516	-0.895998	5.594076
H	3.788023	1.008434	4.396890
H	3.236370	1.469935	2.010434
H	0.473520	-1.879073	2.009876
H	1.041166	-2.341515	4.397554
C	4.368389	-2.503255	-2.453759
C	4.658721	-2.251474	-1.103349
C	3.782266	-1.479703	-0.323044
C	2.312272	-1.219188	-2.257019
C	3.195956	-1.985351	-3.030600
H	5.053402	-3.107236	-3.056899
H	5.569358	-2.657182	-0.651720
H	4.010842	-1.289212	0.730078
H	1.393227	-0.822190	-2.703366
H	2.965813	-2.183602	-4.082098
O	-3.708629	0.923060	0.352630
C	-4.806305	1.794052	0.746349
H	-3.257875	-0.365358	1.301763
C	-6.075869	1.027555	1.115731
H	-5.000054	2.519014	-0.063945
H	-4.421422	2.360185	1.610317
H	-5.896790	0.336041	1.956909
H	-6.477202	0.451569	0.264995
H	-6.860723	1.735283	1.431471
C	-2.875033	-3.292023	-0.440764
H	-4.486093	-1.850221	-0.051248

H	-3.446967	-3.822991	-1.226669
H	-1.807826	-3.294646	-0.708699
H	-3.017228	-3.863315	0.489760

SCF(BP86) = -1264.02874927
H 0K = -1263.543526
H 298K = -1263.510484
G 298K = -1263.613115
Solvent correction(toluene) = -0.03217747
BP86-D3 correction = -0.08670203
Lowest frequencies = -239.1432 cm-1, 8.4612 cm-1

TS(II-III)a

60

P	-1.476773	0.051804	0.065806
C	-2.072343	1.689677	-0.542241
C	-2.661785	-1.212594	-0.566798
Au	0.734411	-0.370851	-0.594753
C	2.818989	-0.736770	-1.232179
C	3.682523	0.507788	-1.021019
C	3.272238	-1.970267	-0.662945
O	2.757124	-1.699795	1.422930
H	1.778633	-1.618928	1.363046
C	3.193082	1.707663	-1.835376
H	4.739016	0.294794	-1.289314
H	2.445031	-0.893019	-2.265673
C	-1.634661	0.074050	1.905841
H	3.244184	1.473217	-2.912682
H	3.812253	2.602857	-1.668896
H	2.147217	1.949866	-1.577751
C	-4.449026	-3.186299	-1.464565
C	-4.876467	-1.853641	-1.346431
C	-3.987844	-0.863223	-0.898763
C	-2.232286	-2.551293	-0.693004
C	-3.127669	-3.534979	-1.137313
H	-5.144494	-3.953631	-1.818366
H	-5.903735	-1.580471	-1.606630
H	-4.322286	0.175622	-0.815895
H	-1.197888	-2.818892	-0.448636
H	-2.791642	-4.571829	-1.235762
C	-1.813910	0.169045	4.709349
C	-2.781335	-0.526197	3.966744
C	-2.697484	-0.575935	2.565391
C	-0.657955	0.768754	2.654722
C	-0.753930	0.818625	4.052816
H	-1.883020	0.203220	5.800992
H	-3.605986	-1.033680	4.476678
H	-3.453186	-1.119804	1.990537
H	0.169859	1.273535	2.142852
H	0.001154	1.360710	4.630646
C	-2.990238	4.144255	-1.555546
C	-3.262781	3.788111	-0.225167
C	-2.806778	2.562116	0.286769
C	-1.794329	2.051235	-1.879128
C	-2.257709	3.274938	-2.382564
H	-3.345968	5.101555	-1.948872
H	-3.831797	4.464726	0.419890
H	-3.018739	2.289236	1.325079
H	-1.221721	1.374807	-2.524097
H	-2.042878	3.552119	-3.419258
O	3.615960	0.746582	0.422776
C	4.561585	1.711706	0.960594
H	3.068359	-0.755421	1.304916
C	5.979890	1.165206	1.120028
H	4.139844	1.986942	1.941153
H	4.554499	2.621591	0.333985
H	6.435929	0.891133	0.153765
H	5.997866	0.285877	1.785209
H	6.622687	1.939288	1.572607
C	4.678686	-2.303265	-0.273254
H	2.642281	-2.846522	-0.873187

H	5.196189	-2.595560	-1.210767
H	5.225048	-1.456021	0.162352
H	4.721014	-3.163657	0.409026

SCF(BP86) = -1264.02657328
H 0K = -1263.541396
H 298K = -1263.508239
G 298K = -1263.611956
Solvent correction(toluene) = -0.03089096
BP86-D3 correction = -0.08816101
Lowest frequencies = -228.3221 cm-1, 3.8292 cm-1

IIId

60

P	1.401104	0.095043	0.005132
C	2.576588	-1.129367	-0.716286
C	1.597826	1.669284	-0.937462
Au	-0.824611	-0.655697	-0.055165
C	-2.935358	-1.246386	-0.850072
C	-3.812075	-0.018214	-1.038123
C	-2.742732	-1.908669	0.357045
O	-1.755892	1.690949	1.325328
H	-1.741375	1.428691	2.263669
C	-3.242443	0.985820	-2.052077
H	-4.773663	-0.415469	-1.432774
H	-2.683378	-1.768617	-1.784719
C	1.962860	0.416067	1.734218
H	-3.937233	1.828110	-2.198176
H	-3.092444	0.502864	-3.032514
H	-2.273612	1.383726	-1.706714
C	1.856614	4.092856	-2.328247
C	2.761824	3.046927	-2.568574
C	2.638120	1.831939	-1.875183
C	0.679763	2.717573	-0.696439
C	0.820716	3.927672	-1.392366
H	1.957376	5.037658	-2.871567
H	3.568754	3.173124	-3.297026
H	3.345812	1.019325	-2.064600
H	-0.130060	2.585153	0.031534
H	0.115478	4.743431	-1.204032
C	2.836469	0.817706	4.374698
C	3.117584	1.776263	3.388420
C	2.683806	1.580622	2.066589
C	1.676736	-0.545708	2.728107
C	2.117444	-0.344298	4.043741
H	3.175413	0.975721	5.403211
H	3.676022	2.681838	3.644807
H	2.900524	2.332200	1.301553
H	1.116778	-1.452328	2.470732
H	1.897258	-1.092608	4.811501
C	4.357250	-2.954004	-1.897202
C	4.782632	-2.151361	-0.825963
C	3.897660	-1.236965	-0.232924
C	2.149267	-1.940739	-1.789045
C	3.041708	-2.847371	-2.379580
H	5.050069	-3.667706	-2.353622
H	5.805621	-2.237128	-0.446840
H	4.231341	-0.617213	0.605223
H	1.119057	-1.862135	-2.155251
H	2.707863	-3.475840	-3.211027
O	-4.060603	0.589151	0.243658
C	-5.353632	1.251799	0.361633
H	-2.639937	1.397236	0.986865
C	-6.473030	0.277868	0.718050
H	-5.574628	1.800453	-0.573284
H	-5.213457	2.000313	1.158398
H	-6.259828	-0.236359	1.668982
H	-6.627116	-0.483340	-0.065469
H	-7.422051	0.828567	0.831342
C	-2.301493	-3.345823	0.489109
H	-3.172997	-1.434593	1.249789

H	-3.190303	-3.963237	0.721359
H	-1.847251	-3.733178	-0.436111
H	-1.592989	-3.485970	1.321502

SCF(BP86) = -1264.06142538
H 0K = -1263.575733
H 298K = -1263.541258
G 298K = -1263.646290
Solvent correction(toluene) = -0.03066371
BP86-D3 correction = -0.08729298
Lowest frequencies = 10.2337 cm-1, 19.0744 cm-1

IIIa

60

P	-1.432314	0.056426	0.045268
C	-2.659899	-0.399143	-1.251761
C	-1.960788	-0.748625	1.619021
Au	0.765500	-0.562625	-0.509707
C	2.874173	-0.791903	-1.463993
C	3.848530	0.322809	-1.113925
C	2.592202	-1.984417	-0.796185
O	1.714043	0.877558	1.800513
H	1.380812	1.767735	1.583394
C	3.419441	1.659966	-1.743023
H	4.818268	0.013958	-1.563701
H	2.565470	-0.750932	-2.519036
C	-1.569648	1.884763	0.282647
H	3.364595	1.573549	-2.841222
H	4.143191	2.456165	-1.509097
H	2.427349	1.963608	-1.367967
C	-2.722862	-1.947545	4.038915
C	-3.629480	-1.892781	2.967984
C	-3.254439	-1.293677	1.754882
C	-1.042696	-0.806576	2.692718
C	-1.434563	-1.402796	3.900934
H	-3.019501	-2.418051	4.981560
H	-4.632202	-2.318498	3.072709
H	-3.962273	-1.256156	0.921357
H	-0.037157	-0.382515	2.582782
H	-0.727074	-1.446375	4.735001
C	-1.754593	4.675538	0.568663
C	-2.188383	3.832861	1.603997
C	-2.099457	2.437028	1.466077
C	-1.128871	2.734218	-0.757652
C	-1.227033	4.125473	-0.613321
H	-1.827669	5.761752	0.680358
H	-2.601317	4.258898	2.523595
H	-2.439797	1.783018	2.274515
H	-0.723712	2.307576	-1.682554
H	-0.892571	4.780729	-1.423734
C	-4.546114	-1.183823	-3.180236
C	-4.750388	-0.010424	-2.435849
C	-3.811797	0.386080	-1.469967
C	-2.453904	-1.575361	-2.004048
C	-3.399350	-1.966487	-2.963208
H	-5.279035	-1.486548	-3.934454
H	-5.641411	0.601438	-2.607051
H	-3.972212	1.304044	-0.895926
H	-1.553643	-2.178384	-1.838760
H	-3.236892	-2.878232	-3.546251
O	4.019111	0.435599	0.311565
C	5.297662	0.997091	0.731927
H	2.577116	0.803598	1.319827
C	6.421767	-0.035432	0.741774
H	5.108749	1.380842	1.747493
H	5.551881	1.862091	0.090700
H	6.623399	-0.444396	-0.262684
H	6.187516	-0.870981	1.420413
H	7.353182	0.439407	1.093635
C	3.130491	-2.490590	0.515256
H	2.062995	-2.737488	-1.399784

H	3.906052	-3.249397	0.294669
H	3.577286	-1.695257	1.122112
H	2.343596	-3.006920	1.089534

SCF(BP86) = -1264.05654077
H 0K = -1263.570845
H 298K = -1263.536286
G 298K = -1263.642153
Solvent correction(toluene) = -0.03107227
BP86-D3 correction = -0.08865973
Lowest frequencies = 10.9816 cm-1, 17.5420 cm-1

IIId (no water)

57

P	1.285784	0.129835	0.046247
C	2.488691	-0.775865	-1.016008
C	1.352742	1.910511	-0.427240
Au	-0.894873	-0.709827	-0.164785
C	-3.044419	-1.175227	-0.926213
C	-3.810872	0.137763	-0.940330
C	-2.824543	-1.932787	0.219837
C	-3.338972	1.089013	-2.052301
H	-4.868948	-0.148116	-1.144314
H	-2.889008	-1.643754	-1.909373
C	1.887094	-0.000125	1.785467
H	-3.944944	2.008665	-2.059700
H	-3.439275	0.615316	-3.043920
H	-2.283275	1.369437	-1.897705
C	1.429506	4.642926	-1.068856
C	2.467675	3.794804	-1.486657
C	2.435298	2.427458	-1.168249
C	0.304208	2.761895	-0.013031
C	0.349804	4.126855	-0.331398
H	1.458801	5.707307	-1.321598
H	3.306689	4.195181	-2.063926
H	3.244405	1.768663	-1.498091
H	-0.542022	2.358380	0.554849
H	-0.461417	4.786682	-0.008596
C	2.820798	-0.274885	4.419804
C	3.021949	0.928812	3.725978
C	2.557609	1.072313	2.408105
C	1.681298	-1.209551	2.485361
C	2.152365	-1.344422	3.798685
H	3.181850	-0.380123	5.447476
H	3.540729	1.762368	4.209251
H	2.713154	2.013129	1.871529
H	1.158858	-2.043409	2.002626
H	1.992883	-2.282348	4.339439
C	4.306360	-2.100378	-2.697775
C	4.714090	-1.670046	-1.424732
C	3.809919	-1.007103	-0.579409
C	2.078229	-1.213622	-2.293989
C	2.989579	-1.871117	-3.132580
H	5.013899	-2.620313	-3.350953
H	5.737928	-1.852376	-1.084148
H	4.128702	-0.679290	0.414910
H	1.049147	-1.040980	-2.629608
H	2.669788	-2.210733	-4.122495
O	-3.706547	0.724521	0.358544
C	-4.827762	1.561045	0.739674
C	-6.007320	0.754657	1.281630
H	-5.136336	2.193846	-0.115368
H	-4.425024	2.230992	1.516744
H	-5.706104	0.160945	2.159985
H	-6.426462	0.070586	0.523891
H	-6.817830	1.436189	1.590886
C	-2.466599	-3.398142	0.225214
H	-3.161739	-1.489322	1.167516
H	-3.378459	-3.977436	0.465314
H	-2.089271	-3.744928	-0.749676
H	-1.726511	-3.640567	1.005079

SCF(BP86) = -1187.62359147
H 0K = -1187.162408
H 298K = -1187.130732
G 298K = -1187.231102
Solvent correction(toluene) = -0.03045171
BP86-D3 correction = -0.07770280
Lowest frequencies = 7.9289 cm-1, 18.3823 cm-1

IIIa (no water)

57

P	-1.297131	0.091801	0.096491
C	-2.584019	-1.225137	-0.003682
C	-1.324807	0.758641	1.813170
Au	0.830761	-0.701294	-0.493477
C	2.943943	-0.909477	-1.403370
C	3.805282	0.275140	-0.987708
C	2.686993	-2.090407	-0.704303
C	3.482111	1.513254	-1.842807
H	4.858761	-0.030580	-1.183462
H	2.692065	-0.917196	-2.474074
C	-1.814185	1.448634	-1.042218
H	3.655914	1.303700	-2.912000
H	4.122091	2.365378	-1.567394
H	2.428747	1.811355	-1.706593
C	-1.346687	1.850944	4.398944
C	-2.507641	1.253835	3.880430
C	-2.502635	0.705599	2.588324
C	-0.155602	1.351866	2.336870
C	-0.172901	1.899897	3.628044
H	-1.354669	2.273134	5.408581
H	-3.419902	1.209665	4.483175
H	-3.405810	0.232338	2.190308
H	0.763344	1.376955	1.739549
H	0.733314	2.358428	4.035597
C	-2.605841	3.463477	-2.831765
C	-2.752448	3.655518	-1.448922
C	-2.358953	2.651356	-0.548896
C	-1.661034	1.257774	-2.433943
C	-2.062240	2.263849	-3.323945
H	-2.912527	4.249829	-3.528309
H	-3.174066	4.589436	-1.064810
H	-2.473807	2.805190	0.528274
H	-1.234867	0.324233	-2.819011
H	-1.946064	2.113184	-4.401643
C	-4.525504	-3.253203	-0.068056
C	-4.824111	-1.979732	-0.578126
C	-3.857413	-0.961192	-0.548670
C	-2.282991	-2.507415	0.505529
C	-3.255791	-3.516311	0.474884
H	-5.281181	-4.044191	-0.096867
H	-5.811460	-1.774973	-1.003253
H	-4.091785	0.028549	-0.952297
H	-1.292417	-2.713964	0.926815
H	-3.020938	-4.509630	0.869454
O	3.619786	0.518677	0.413763
C	4.673017	1.292586	1.043875
C	5.926899	0.475179	1.355934
H	4.215577	1.668023	1.973677
H	4.922246	2.173763	0.422124
H	6.407823	0.079294	0.445443
H	5.696617	-0.369783	2.025019
H	6.668936	1.116041	1.861730
C	3.213561	-2.510418	0.642118
H	2.219547	-2.892281	-1.296573
H	4.059169	-3.205309	0.473848
H	3.567108	-1.655430	1.230625
H	2.454823	-3.074941	1.209002

SCF(BP86) = -1187.61997735
H 0K = -1187.158718
H 298K = -1187.127012
G 298K = -1187.228605
Solvent correction(toluene) = -0.03045938
BP86-D3 correction = -0.07850106
Lowest frequencies = 3.5373 cm-1, 18.2232 cm-1

(iii) Three EtOH nucleophiles

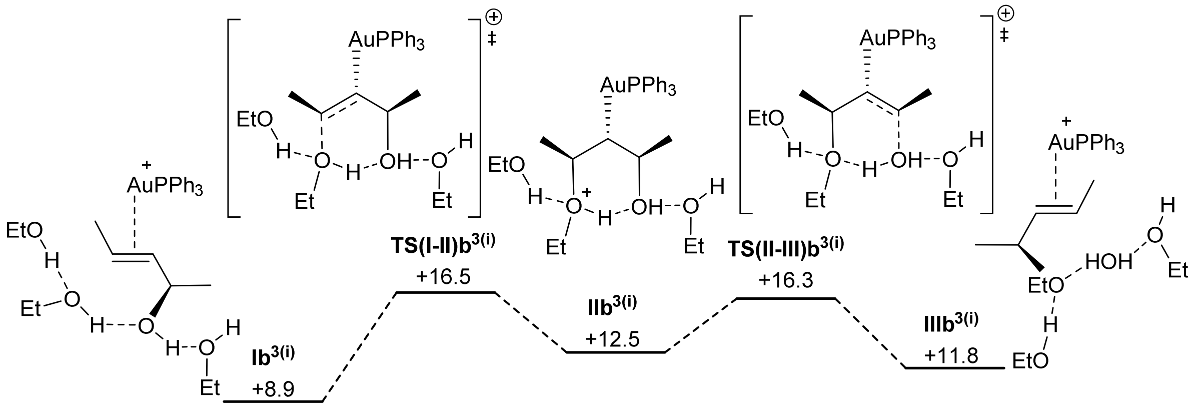

Figure S7. Computed free energy profile (BP86-D3(toluene)) for direct etherification with three EtOH nucleophiles at $\mathbf{Ib}^{3(i)}$ leading to (*S*,*E*)-**5ko**. All energies are in kcal/mol and are quoted relative to $\mathbf{Ia}^{3(ii)}$ set to 0.0 kcal/mol.

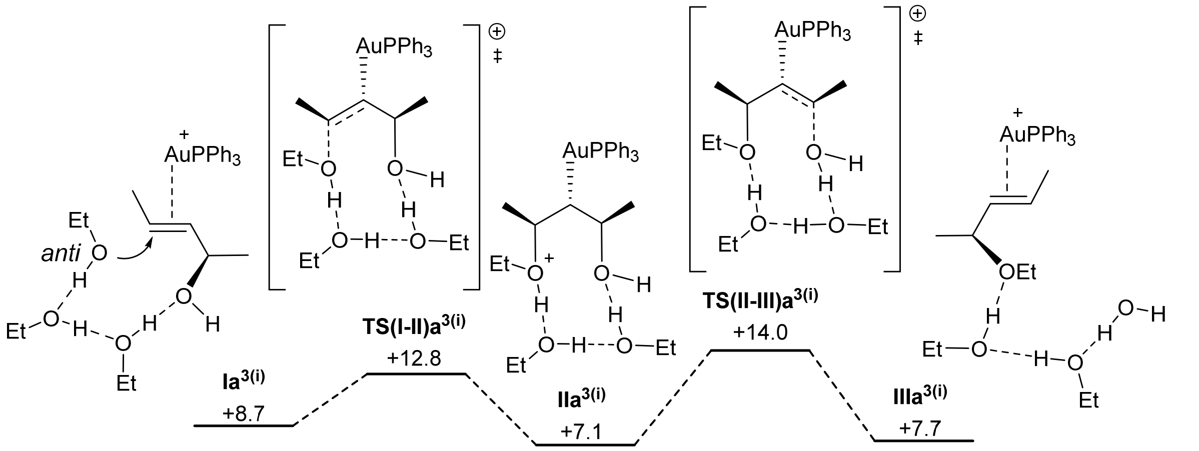

Figure S8. Computed free energy profile (BP86-D3(toluene)) for direct etherification with three EtOH nucleophiles at $\mathbf{Ia}^{3(i)}$ leading to (*S*,*E*)-**5ko**. All energies are in kcal/mol and are quoted relative to $\mathbf{Ia}^{3(ii)}$ set to 0.0 kcal/mol.

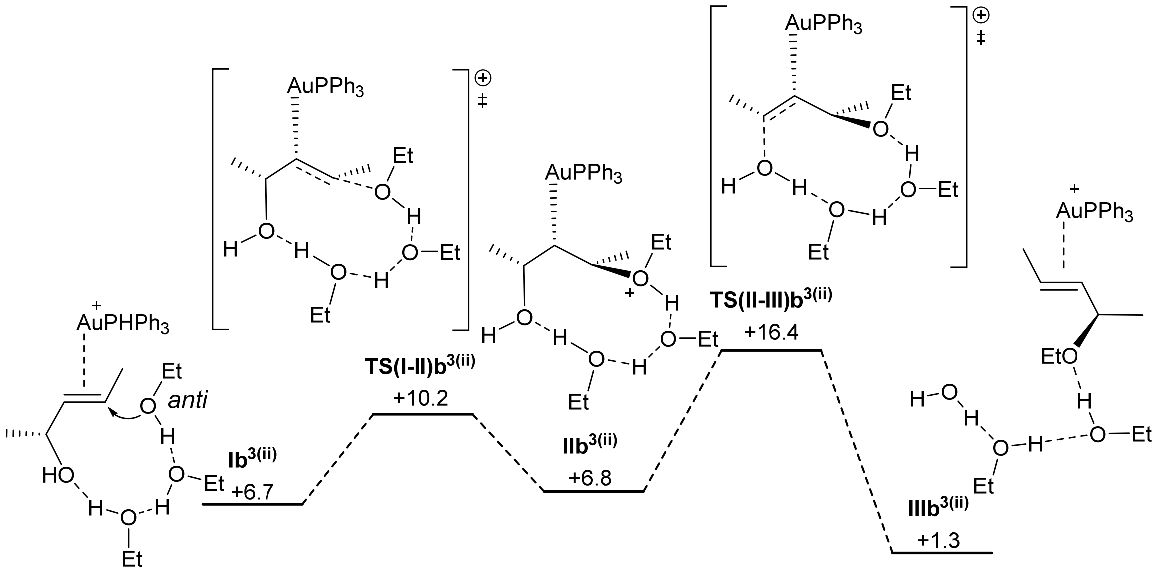

Figure S9. Computed free energy profile (BP86-D3(toluene)) for direct etherification with three EtOH nucleophiles at **Ib**$^{3(ii)}$ leading to (*R*,*E*)-**5ko**. All energies are in kcal/mol and are quoted relative to **Ia**$^{3(ii)}$ set to 0.0 kcal/mol.

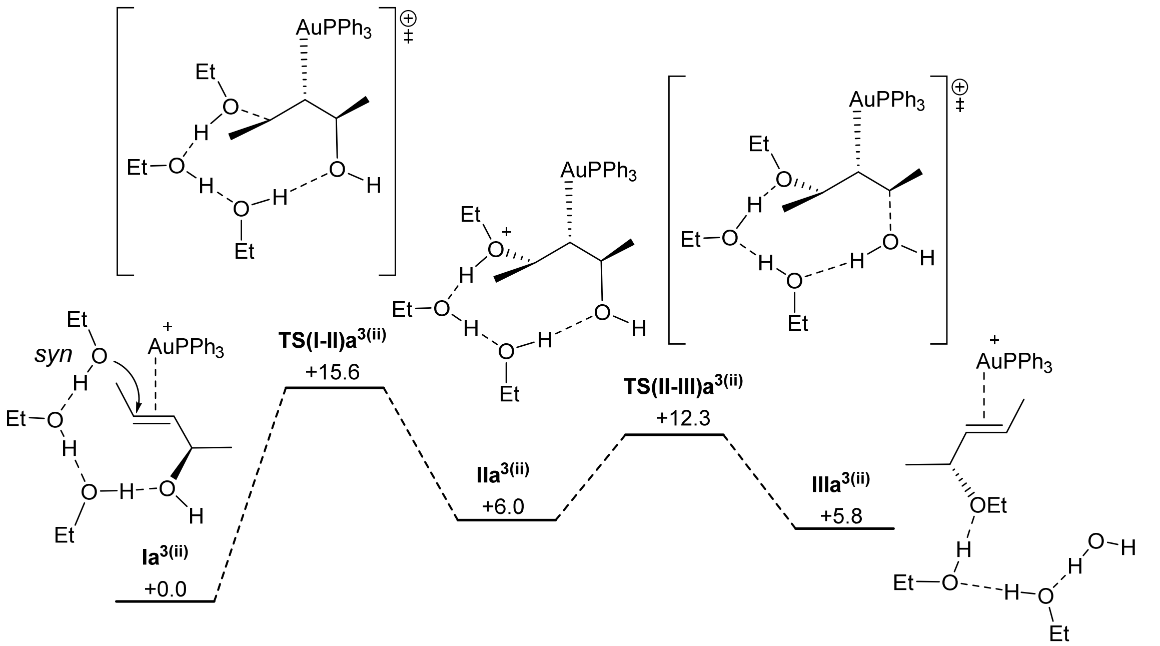

Figure S10. Computed free energy profile (BP86-D3(toluene)) for direct etherification with three EtOH nucleophiles at **Ia**$^{3(ii)}$ leading to (*R*,*E*)-**5ko**. All energies are in kcal/mol and are quoted relative to **Ia**$^{3(ii)}$ set to 0.0 kcal/mol.

Table S3. Energies associated with direct etherification with three EtOH nucleophiles. Energies are reported as ΔE (gas phase SCF energies), ΔG (free energies at 298.15 K and 1atm), ΔG_{disp} (including a correction for dispersion effects using Grimme's D3 parameter set) and $\Delta G_{toluene+disp}$ (including an additional correction for toluene solvent *via* the PCM approach). All energies relative to $\mathbf{Ia^{3(ii)}}$.

**Isomer**	**ΔE**	**ΔG**	$\mathbf{\Delta G_{disp}}$	$\mathbf{\Delta G_{toluene+disp}}$
$Ib^{3(i)}$	+4.4	+2.0	+9.0	+8.9
$TS(I\text{-}II)b^{3(i)}$	+12.4	+11.3	+16.5	+16.5
$IIb^{3(i)}$	+8.9	+8.7	+13.6	+12.5
$TS(II\text{-}III)b^{3(i)}$	+13.0	+12.2	+16.6	+16.3
$IIIb^{3(i)}$	+10.9	+9.0	+13.0	+11.8
$Ia^{3(i)}$	+6.2	+4.6	+10.5	+8.7
$TS(I\text{-}II)a^{3(i)}$	+8.8	+10.2	+12.5	+12.8
$IIa^{3(i)}$	+3.5	+6.6	+7.0	+7.1
$TS(II\text{-}III)a^{3(i)}$	+12.2	+12.2	+14.0	+14.0
$IIIa^{3(i)}$	+4.6	+4.5	+8.0	+7.7
$Ib^{3(ii)}$	+3.1	+2.2	+7.3	+6.7
$TS(I\text{-}II)b^{3(ii)}$	+8.3	+9.7	+10.2	+10.2
$IIb^{3(ii)}$	+4.5	+6.8	+7.3	+6.8
$TS(II\text{-}III)b^{3(ii)}$	+15.3	+17.0	+16.5	+16.4
$IIIb^{3(ii)}$	-0.6	+0.4	+1.0	+1.3
$Ia^{3(ii)}$	+0.0	+0.0	+0.0	+0.0
$TS(I\text{-}II)a^{3(ii)}$	+14.9	+15.9	+15.4	+15.6
$IIa^{3(ii)}$	+5.0	+7.9	+6.3	+6.0
$TS(II\text{-}III)a^{3(ii)}$	+9.1	+12.5	+12.2	+12.3
$IIIa^{3(ii)}$	+4.6	+4.3	+6.1	+5.8
Direct S_N2	+22.2	+19.3	+22.5	+22.6

Ib3(i)

78

P	-2.567998	0.143286	0.093875
C	-2.848750	-0.472527	1.808918
C	-3.162408	1.888175	0.030504
Au	-0.316129	-0.037343	-0.551026
C	1.960350	-0.036858	-0.611706
C	1.553580	-0.519628	-1.854714
C	2.523651	1.366531	-0.419645
O	3.937109	1.191214	-0.629929
H	4.414307	2.008979	-0.307303
C	1.502593	-1.977818	-2.226264
H	1.482604	0.203139	-2.683613
H	2.224738	-0.779962	0.164861
C	-3.658877	-0.847907	-1.017609
H	2.390963	-2.178694	-2.855388
H	0.615345	-2.226241	-2.831991
H	1.571636	-2.628040	-1.340176
C	-4.077653	4.537900	-0.150733
C	-4.513864	3.714971	0.900115
C	-4.060435	2.389378	0.995294
C	-2.718867	2.718716	-1.021996
C	-3.181750	4.039337	-1.112160
H	-4.432183	5.571041	-0.218773
H	-5.207940	4.103795	1.651556
H	-4.398615	1.752336	1.818365
H	-2.013713	2.331883	-1.766750
H	-2.837780	4.680873	-1.929348
C	-5.290181	-2.425251	-2.672389
C	-5.670171	-1.110467	-2.360173
C	-4.858772	-0.316825	-1.532430
C	-3.274062	-2.169368	-1.335824
C	-4.093561	-2.954880	-2.158316
H	-5.924585	-3.038286	-3.319966
H	-6.600536	-0.696604	-2.761110
H	-5.157078	0.708374	-1.293111
H	-2.339781	-2.582587	-0.938703
H	-3.794856	-3.978973	-2.402509
C	-3.257704	-1.333704	4.450605
C	-4.240721	-1.569645	3.475798
C	-4.041660	-1.141931	2.153385
C	-1.858878	-0.240912	2.788316
C	-2.067976	-0.669320	4.107021
H	-3.415693	-1.673685	5.478771
H	-5.164947	-2.091894	3.741599
H	-4.805484	-1.335754	1.393861
H	-0.926984	0.268727	2.518180
H	-1.298129	-0.491751	4.864215
O	4.744929	-1.464116	-0.944637
C	6.111304	-1.694656	-1.326063
H	4.614089	-0.497524	-0.764325
C	7.098825	-1.470324	-0.179038
H	6.373966	-1.055282	-2.193009
H	6.156213	-2.741778	-1.671524
H	6.862905	-2.123437	0.677538
H	7.070449	-0.423594	0.172028
H	8.131012	-1.688196	-0.503585
C	2.225005	1.950272	0.967010
H	2.108759	2.039200	-1.203450

H	1.141295	2.116022	1.099584
H	2.576286	1.264377	1.756590
O	5.130178	3.498677	0.176874
C	6.252107	3.557767	1.098194
H	6.284473	4.560376	1.565434
C	7.582512	3.220396	0.432224
H	6.004731	2.830448	1.888187
H	7.561743	2.213002	-0.015095
H	7.831353	3.947763	-0.361062
H	8.399453	3.251480	1.172728
H	5.332651	4.089713	-0.573506
O	2.905845	-2.682704	0.674851
C	3.370932	-3.376512	1.839937
H	4.118789	-2.766887	2.391037
C	3.954789	-4.753095	1.511385
H	2.494225	-3.485943	2.502994
H	3.212291	-5.371988	0.981094
H	4.847496	-4.664114	0.867982
H	4.255575	-5.283479	2.431752
H	3.696360	-2.409364	0.134117
H	2.738122	2.917909	1.089593

SCF(BP86) = -1574.16277147
H 0K = -1573.517225
H 298K = -1573.470917
G 298K = -1573.611668
Solvent correction(toluene) = -0.02938452
BP86-D3 correction = -0.10029286
Lowest frequencies = 4.4903 cm-1, 10.4339 cm-1

Ia$^{3(i)}$

78

P	-2.566491	0.004988	0.124564
C	-3.764236	-0.091594	-1.272941
C	-2.900930	-1.423437	1.242451
Au	-0.340938	0.009285	-0.629950
C	1.682876	-0.435770	-1.577308
C	1.823734	0.824253	-0.989289
C	2.276139	-1.707441	-0.969783
O	3.667225	-1.798159	-1.369222
H	3.714390	-2.043062	-2.313731
C	1.749016	2.128927	-1.716654
H	2.256781	0.869344	0.026248
H	1.461140	-0.483157	-2.657089
C	-2.954652	1.542637	1.067731
H	2.809693	2.483024	-1.772379
H	1.200381	2.894122	-1.142258
H	1.324645	2.041923	-2.729388
C	-3.374846	-3.558360	3.005373
C	-4.338417	-3.215183	2.043583
C	-4.107654	-2.147927	1.160297
C	-1.929316	-1.772853	2.206401
C	-2.171768	-2.835842	3.088079
H	-3.558962	-4.392102	3.689908
H	-5.274029	-3.778794	1.976740
H	-4.859920	-1.884889	0.410414
H	-0.989130	-1.212606	2.268216
H	-1.419283	-3.103442	3.836311
C	-3.554374	3.922885	2.431724
C	-3.951848	2.687819	2.967737
C	-3.655279	1.493685	2.290068
C	-2.551079	2.785190	0.530626
C	-2.856180	3.971372	1.212368
H	-3.785343	4.849916	2.965410
H	-4.494330	2.649042	3.917338
H	-3.964421	0.532795	2.712365
H	-2.004587	2.824628	-0.418581
H	-2.543752	4.933311	0.794545
C	-5.585351	-0.319894	-3.398825
C	-5.904217	0.472193	-2.283857
C	-4.997847	0.590348	-1.218054
C	-3.442657	-0.883455	-2.396682
C	-4.355675	-0.998130	-3.454723
H	-6.293407	-0.404588	-4.228915
H	-6.859493	1.004354	-2.242286
H	-5.245988	1.215047	-0.354379
H	-2.480253	-1.406154	-2.443156
H	-4.104028	-1.610614	-4.325963
O	4.785009	2.548942	-1.181579
C	5.725805	1.793658	-1.953377
H	4.638856	2.069654	-0.322665
C	7.074564	1.627095	-1.248504
H	5.854040	2.348109	-2.899973
H	5.318205	0.790983	-2.218837
H	6.967485	1.002254	-0.345200
H	7.479647	2.609986	-0.957060
H	7.806132	1.133146	-1.912029

C	1.518025	-2.982491	-1.343831
H	2.325912	-1.593991	0.126937
H	0.487107	-2.957670	-0.951030
H	1.454969	-3.097118	-2.441657
O	5.470734	-1.053015	0.626113
C	5.833234	-2.190725	1.437283
H	6.317605	-1.772241	2.335384
C	6.784097	-3.134766	0.704467
H	4.926653	-2.734879	1.774393
H	6.302733	-3.565604	-0.190904
H	7.693762	-2.600808	0.386306
H	7.081719	-3.970935	1.359843
H	4.986173	-1.379908	-0.170498
O	4.097383	1.188519	1.176674
C	4.487123	1.881819	2.381647
H	5.590477	1.965782	2.432295
C	3.942563	1.208297	3.641511
H	4.086949	2.905714	2.287336
H	2.841627	1.145262	3.607420
H	4.344290	0.186476	3.755091
H	4.226198	1.781133	4.541206
H	4.614199	0.330369	1.121640
H	2.028666	-3.866299	-0.930735

SCF(BP86) = -1574.16000197
H 0K = -1573.514332
H 298K = -1573.468246
G 298K = -1573.607575
Solvent correction(toluene) = -0.03194207
BP86-D3 correction = -0.10205099
Lowest frequencies = 4.1101 cm-1, 9.1401 cm-1

Ib$^{3(ii)}$

78

P	-2.572837	0.029773	0.086837
C	-3.455110	-0.727633	1.516910
C	-3.029529	1.816041	0.035286
Au	-0.243633	-0.241498	0.230785
C	1.912241	-1.065825	0.002623
C	1.955838	0.066734	0.815161
C	2.319395	-1.112730	-1.451462
O	2.313711	0.237686	-1.985635
H	2.423973	0.158055	-2.952418
C	2.094942	0.019747	2.314735
H	2.193847	1.028875	0.340203
H	1.898822	-2.051174	0.488144
C	-3.256416	-0.756957	-1.437602
H	1.546735	0.836328	2.811712
H	3.174881	0.163500	2.518649
H	1.786041	-0.947764	2.741076
C	-3.703139	4.538602	-0.115314
C	-4.537965	3.646239	0.576871
C	-4.206594	2.284249	0.655222
C	-2.186742	2.714995	-0.654703
C	-2.529194	4.072862	-0.731660
H	-3.964191	5.599998	-0.169730
H	-5.449384	4.009262	1.061700
H	-4.855762	1.593196	1.201812
H	-1.265817	2.352912	-1.126392
H	-1.874765	4.768461	-1.265987
C	-4.269450	-2.025631	-3.730477
C	-4.584096	-0.681883	-3.474659
C	-4.081104	-0.042411	-2.329232
C	-2.935345	-2.108252	-1.698024
C	-3.447070	-2.739054	-2.840624
H	-4.664124	-2.519122	-4.623982
H	-5.224943	-0.125679	-4.165737
H	-4.330055	1.004651	-2.132310
H	-2.296647	-2.667107	-1.004474
H	-3.202097	-3.787373	-3.037587
C	-4.789580	-1.800535	3.743402
C	-5.394649	-1.840497	2.476601
C	-4.732438	-1.305888	1.359908
C	-2.845850	-0.693132	2.789786
C	-3.516456	-1.226451	3.899868
H	-5.308143	-2.222716	4.609708
H	-6.383947	-2.291868	2.353552
H	-5.202947	-1.346793	0.372564
H	-1.849306	-0.252784	2.908996
H	-3.041543	-1.200629	4.885428
C	1.469568	-2.056004	-2.307163
H	3.366201	-1.496449	-1.397105
H	1.485703	-3.074574	-1.885217
H	0.422170	-1.711961	-2.362151
O	4.741846	-2.232596	0.027260
C	5.816477	-3.179635	0.036672
H	6.167713	-3.365221	1.073695
C	6.993933	-2.756253	-0.847056
H	5.392353	-4.131731	-0.329736
H	6.663868	-2.599054	-1.887821
H	7.444666	-1.815589	-0.485052

H	7.783899	-3.527346	-0.848599
H	5.055383	-1.400334	0.477567
O	5.273369	0.197415	1.226694
C	6.554327	0.600951	1.751104
H	6.876560	-0.213467	2.422169
C	6.479349	1.922151	2.516466
H	7.303799	0.669180	0.935794
H	6.159625	2.748788	1.858288
H	5.762717	1.851010	3.351234
H	7.467204	2.186477	2.931206
H	4.994391	0.856639	0.530399
O	4.210684	1.857434	-0.681841
C	4.890935	2.865839	-1.458952
H	5.564785	3.380201	-0.753186
C	3.919654	3.862402	-2.090117
H	5.527213	2.393154	-2.234526
H	3.244776	3.365640	-2.809310
H	3.303493	4.348348	-1.316067
H	4.470925	4.645740	-2.637635
H	3.644136	1.311865	-1.284626
H	1.874820	-2.123522	-3.332015

SCF(BP86) = -1574.16492468
H 0K = -1573.518931
H 298K = -1573.472954
G 298K = -1573.611295
Solvent correction(toluene) = -0.03002573
BP86-D3 correction = -0.10345170
Lowest frequencies = 4.0071 cm-1, 8.7305 cm-1

Ia$^{3(ii)}$

78

P	-2.129635	0.031315	0.059348
C	-3.400392	-1.305505	-0.003347
C	-2.266528	0.842257	1.711455
Au	0.046606	-0.759690	-0.343633
C	1.856803	-2.155682	-0.115851
C	2.078601	-1.390919	-1.259939
C	2.536667	-1.875364	1.224905
O	3.897946	-2.391286	1.161612
H	3.859513	-3.366345	1.100745
C	1.832820	-1.870739	-2.667622
H	2.682174	-0.479516	-1.155418
H	1.430862	-3.164871	-0.240666
C	-2.635428	1.280629	-1.205339
H	2.806695	-2.131730	-3.123819
H	1.392851	-1.080737	-3.298238
H	1.185440	-2.761319	-2.702102
C	-2.429900	2.130056	4.199196
C	-3.458107	1.266415	3.788740
C	-3.382457	0.620145	2.544419
C	-1.225464	1.705584	2.122926
C	-1.319222	2.349599	3.366184
H	-2.492750	2.630567	5.170624
H	-4.322668	1.091972	4.436724
H	-4.184546	-0.053725	2.228300
H	-0.356498	1.870586	1.470668
H	-0.517738	3.022408	3.687793
C	-3.420381	3.114845	-3.184673
C	-3.590318	3.430491	-1.827492
C	-3.200288	2.516975	-0.833612
C	-2.460593	0.965697	-2.571135
C	-2.857216	1.881137	-3.555984
H	-3.725306	3.829970	-3.954984
H	-4.028866	4.390095	-1.536566
H	-3.332599	2.766651	0.223395
H	-2.022325	0.004186	-2.862330
H	-2.723945	1.633073	-4.613633
C	-5.326776	-3.352092	-0.006293
C	-5.675138	-2.059623	-0.431588
C	-4.717063	-1.033382	-0.431464
C	-3.051371	-2.606091	0.418055
C	-4.015697	-3.624587	0.419302
H	-6.076211	-4.149622	-0.012086
H	-6.694639	-1.847554	-0.768239
H	-4.990747	-0.029410	-0.770851
H	-2.024464	-2.817113	0.737404
H	-3.741540	-4.632875	0.744928
O	1.203503	1.802820	-0.177570
C	1.251867	2.923393	-1.081287
H	2.105553	1.705043	0.248158
C	2.238173	2.721251	-2.232761
H	1.507830	3.846250	-0.521300
H	0.225519	3.050160	-1.465524
H	1.962351	1.841107	-2.838785
H	3.262228	2.572594	-1.849831
H	2.249910	3.602782	-2.896896

C	1.788955	-2.463906	2.420422
H	2.683071	-0.787751	1.350156
H	0.795359	-1.996404	2.526059
H	1.638759	-3.553113	2.302449
O	5.113742	-0.416064	-0.339820
C	6.545800	-0.363224	-0.540053
H	6.744203	0.620678	-0.997359
C	7.040387	-1.486608	-1.448659
H	7.071465	-0.389603	0.435196
H	6.858351	-2.477278	-0.996742
H	6.534079	-1.450754	-2.427028
H	8.126517	-1.393931	-1.618286
H	4.886306	-1.244728	0.156443
O	3.583090	1.336476	0.995735
C	4.237591	2.105637	2.025512
H	3.432692	2.494070	2.672611
C	5.075391	3.254215	1.463526
H	4.862803	1.435910	2.648268
H	5.891618	2.878187	0.823018
H	4.453841	3.938796	0.862946
H	5.533307	3.835395	2.282366
H	4.266194	0.804141	0.487446
H	2.357502	-2.289656	3.346862

SCF(BP86) = -1574.1698524
H 0K = -1573.523926
H 298K = -1573.477927
G 298K = -1573.614867
Solvent correction(toluene) = -0.02909093
BP86-D3 correction = -0.11151438
Lowest frequencies = 6.4984 cm-1, 11.4816 cm-1

TS(I-II)b$^{3(i)}$

78

P	-2.575016	0.107267	0.015841
C	-3.231135	-0.109457	1.729557
C	-3.207294	1.737110	-0.585878
Au	-0.231514	-0.035649	-0.077389
C	1.926157	-0.134607	-0.034650
C	2.209511	-0.916170	-1.223123
C	2.540778	1.268429	-0.004950
O	3.998905	1.074221	0.038538
H	4.461538	1.960284	-0.078041
C	1.873676	-2.382319	-1.325793
H	2.194979	-0.359969	-2.174443
H	2.142083	-0.700849	0.890933
C	-3.408328	-1.186535	-1.011595
H	2.434618	-2.858939	-2.145697
H	0.800970	-2.496320	-1.560092
H	2.096334	-2.896375	-0.377113
C	-4.139897	4.194221	-1.586665
C	-4.793965	3.582196	-0.505229
C	-4.332736	2.354816	-0.002565
C	-2.546273	2.358628	-1.667776
C	-3.016989	3.581425	-2.168311
H	-4.501822	5.151993	-1.973462
H	-5.665398	4.060703	-0.047486
H	-4.842189	1.884255	0.844137
H	-1.663693	1.884459	-2.112431
H	-2.502636	4.059362	-3.008023
C	-4.632305	-3.221601	-2.517592
C	-5.075311	-1.895250	-2.638013
C	-4.468147	-0.874942	-1.886620
C	-2.959876	-2.521061	-0.896055
C	-3.575827	-3.534161	-1.644229
H	-5.107914	-4.012899	-3.105402
H	-5.896962	-1.649320	-3.317995
H	-4.816900	0.157697	-1.983746
H	-2.134558	-2.765600	-0.217395
H	-3.228364	-4.567698	-1.548516
C	-4.199429	-0.353997	4.357548
C	-4.954153	-0.850449	3.282233
C	-4.474757	-0.731249	1.967905
C	-2.471340	0.382258	2.812708
C	-2.958770	0.262207	4.122350
H	-4.575112	-0.453286	5.380726
H	-5.917898	-1.336094	3.464623
H	-5.061079	-1.128300	1.133215
H	-1.498486	0.852082	2.628601
H	-2.365465	0.642275	4.959848
O	4.140621	-1.071483	-1.194245
C	4.786167	-1.042079	-2.496329
H	4.271300	-0.142942	-0.708648
C	6.288576	-1.265136	-2.351689
H	4.568510	-0.077441	-2.994796
H	4.325373	-1.846805	-3.093859
H	6.496274	-2.229163	-1.861025
H	6.759855	-0.462832	-1.760230
H	6.762810	-1.276406	-3.347490
C	2.118647	2.118226	1.194296
H	2.291573	1.800768	-0.947543

H	1.039533	2.342869	1.147765
H	2.326822	1.583658	2.136162
O	5.144628	3.480793	-0.065533
C	6.059817	3.759517	1.031058
H	6.093067	4.851679	1.201900
C	7.456276	3.194025	0.793344
H	5.586039	3.296554	1.911308
H	7.425815	2.100750	0.654909
H	7.929635	3.647796	-0.095641
H	8.107157	3.411096	1.656983
H	5.532201	3.851941	-0.880821
O	3.596729	-2.600918	1.366462
C	4.396301	-3.375829	2.275701
H	5.224414	-2.763379	2.690546
C	4.947413	-4.654799	1.643106
H	3.725273	-3.621758	3.116206
H	4.129009	-5.281294	1.251718
H	5.635135	-4.425234	0.809162
H	5.512666	-5.246491	2.383654
H	4.164356	-2.328833	0.615870
H	2.668233	3.073832	1.199551

SCF(BP86) = -1574.15015072
H 0K = -1573.504919
H 298K = -1573.460077
G 298K = -1573.596898
Solvent correction(toluene) = -0.02896923
BP86-D3 correction = -0.10326766
Lowest frequencies = -161.2001 cm-1, 3.3853 cm-1

TS(I-II)a$^{3(i)}$

78

P	-2.644980	-0.121611	0.103753
C	-3.645386	-0.734439	-1.321855
C	-2.834094	-1.360355	1.460195
Au	-0.394092	0.220471	-0.467006
C	1.709435	0.393095	-1.121099
C	1.879061	1.588842	-0.358847
C	2.494649	-0.878148	-0.764774
O	3.783345	-0.853732	-1.454688
H	3.622416	-1.076921	-2.392305
C	1.350040	2.932246	-0.798330
H	2.075178	1.470165	0.712986
H	1.602882	0.543842	-2.211057
C	-3.461445	1.432494	0.681869
H	2.060854	3.718346	-0.503407
H	0.391108	3.153294	-0.297096
H	1.188294	2.971751	-1.887441
C	-3.093250	-3.186204	3.581488
C	-4.041009	-3.174283	2.545356
C	-3.916810	-2.263674	1.483696
C	-1.878149	-1.378384	2.499472
C	-2.013095	-2.287374	3.559015
H	-3.193211	-3.899654	4.405365
H	-4.880076	-3.876767	2.559443
H	-4.654866	-2.261217	0.675590
H	-1.031211	-0.682839	2.476302
H	-1.271219	-2.298117	4.363599
C	-4.693612	3.829045	1.481047
C	-4.863646	2.663484	2.244302
C	-4.251323	1.462417	1.848736
C	-3.286877	2.607433	-0.082700
C	-3.907173	3.799734	0.315925
H	-5.171432	4.762500	1.794171
H	-5.474596	2.684522	3.152068
H	-4.386615	0.556219	2.446845
H	-2.671842	2.586705	-0.989759
H	-3.773065	4.707584	-0.280483
C	-5.138340	-1.753119	-3.474211
C	-5.727536	-0.846908	-2.577811
C	-4.986234	-0.334813	-1.500707
C	-3.053207	-1.640985	-2.227630
C	-3.801866	-2.150462	-3.298345
H	-5.719071	-2.146427	-4.314322
H	-6.766816	-0.533241	-2.716463
H	-5.446580	0.377097	-0.808530
H	-2.008611	-1.944365	-2.093858
H	-3.339812	-2.852277	-3.999600
O	3.920427	2.220426	-0.334161
C	4.643430	2.271098	-1.582712
H	4.315604	1.508673	0.284292
C	6.153726	2.319053	-1.349916
H	4.295834	3.182823	-2.099125
H	4.379889	1.395004	-2.204920
H	6.511025	1.381236	-0.891643
H	6.424568	3.164635	-0.696871
H	6.679155	2.446424	-2.311768
C	1.778244	-2.192540	-1.091394

H	2.771140	-0.842792	0.303298
H	0.875116	-2.319824	-0.470773
H	1.460643	-2.216871	-2.150739
O	6.142835	-1.121053	-0.061685
C	6.699847	-2.426170	0.213197
H	7.474419	-2.261256	0.980573
C	7.309257	-3.058968	-1.035874
H	5.931145	-3.091781	0.656030
H	6.540379	-3.245252	-1.806358
H	8.080916	-2.401842	-1.468020
H	7.775526	-4.028062	-0.789857
H	5.381837	-1.218975	-0.689286
O	4.687670	0.477204	1.457994
C	5.342094	1.099231	2.589713
H	6.345762	1.464407	2.296319
C	5.439663	0.149031	3.781246
H	4.732704	1.982909	2.845542
H	4.440163	-0.208291	4.079096
H	6.064141	-0.728922	3.543146
H	5.896624	0.660636	4.645511
H	5.318544	-0.192950	1.045155
H	2.448557	-3.048188	-0.908443

SCF(BP86) = -1574.15576299
H 0K = -1573.509442
H 298K = -1573.464882
G 298K = -1573.598672
Solvent correction(toluene) = -0.02873222
BP86-D3 correction = -0.10773556
Lowest frequencies = -100.9466 cm-1, 2.9776 cm-1

TS(I-II)b$^{3(ii)}$

78

P	-2.373753	0.104108	0.138926
C	-2.742800	0.680780	1.856737
C	-2.775509	1.519815	-0.977628
Au	-0.173549	-0.678597	-0.105397
C	1.782311	-1.611949	-0.425481
C	2.480415	-0.878768	0.582020
C	2.219973	-1.455851	-1.882909
O	2.168621	-0.035631	-2.228984
H	2.376674	0.023885	-3.182449
C	2.338314	-1.145476	2.057147
H	2.813978	0.130388	0.313282
H	1.596408	-2.668567	-0.166859
C	-3.597496	-1.227519	-0.240392
H	1.510588	-0.542481	2.470312
H	3.250105	-0.837678	2.591602
H	2.122233	-2.204806	2.268900
C	-3.406576	3.604810	-2.754304
C	-3.972981	3.581749	-1.469462
C	-3.661353	2.542256	-0.578269
C	-2.200246	1.549628	-2.266372
C	-2.521104	2.588656	-3.152170
H	-3.651479	4.417787	-3.444939
H	-4.658986	4.375045	-1.156523
H	-4.100513	2.530462	0.424232
H	-1.500568	0.762772	-2.570160
H	-2.075366	2.608204	-4.151684
C	-5.431302	-3.298514	-0.742149
C	-5.718309	-1.989341	-1.160900
C	-4.806130	-0.950947	-0.911960
C	-3.307728	-2.546172	0.174119
C	-4.226788	-3.576120	-0.073407
H	-6.144162	-4.104759	-0.941160
H	-6.653978	-1.772497	-1.685596
H	-5.031851	0.066972	-1.244567
H	-2.363635	-2.763154	0.687098
H	-3.998988	-4.596746	0.249534
C	-3.248519	1.627963	4.455350
C	-4.185211	0.802403	3.813922
C	-3.938049	0.327303	2.515116
C	-1.799013	1.506760	2.505993
C	-2.056259	1.981638	3.799819
H	-3.444661	1.993830	5.467990
H	-5.112881	0.523843	4.323478
H	-4.669990	-0.318107	2.019978
H	-0.867272	1.778660	1.996852
H	-1.323817	2.623767	4.299098
C	1.366864	-2.272719	-2.853863
H	3.277535	-1.784823	-1.944047
H	1.423169	-3.346797	-2.608573
H	0.309724	-1.960096	-2.806283
O	4.468519	-1.458484	0.282350
C	4.986668	-2.559450	1.058950
H	5.080085	-2.276333	2.125763
C	6.331404	-3.030536	0.504842
H	4.238086	-3.368630	0.990865
H	6.238301	-3.323276	-0.553168
H	7.095733	-2.239256	0.580747

H	6.691084	-3.902549	1.077086
H	4.977202	-0.586355	0.509778
O	5.470118	0.860873	0.761048
C	6.877361	1.123886	0.538245
H	7.421643	0.464416	1.235880
C	7.225440	2.585906	0.805292
H	7.165354	0.833869	-0.491844
H	6.705968	3.254233	0.097234
H	6.941534	2.873538	1.830436
H	8.309654	2.750626	0.685384
H	4.940151	1.355110	0.062032
O	3.917727	1.863735	-1.165489
C	3.262751	3.150690	-1.146873
H	4.059715	3.887691	-0.951126
C	2.165188	3.252459	-0.087287
H	2.856512	3.370283	-2.153096
H	1.362964	2.517324	-0.277097
H	2.577970	3.073833	0.920116
H	1.711557	4.258643	-0.097629
H	3.287302	1.182376	-1.522691
H	1.725850	-2.157081	-3.891852

SCF(BP86) = -1574.15669217
H 0K = -1573.510600
H 298K = -1573.466056
G 298K = -1573.599402
Solvent correction(toluene) = -0.02916633
BP86-D3 correction = -0.11064853
Lowest frequencies = -104.5242 cm-1, 6.4071 cm-1

TS(I-II)a$^{3(ii)}$

78

P	-2.267172	0.060583	0.082460
C	-3.551669	-1.023249	-0.686406
C	-2.653441	0.127345	1.889109
Au	-0.100016	-0.714864	-0.322440
C	1.869518	-1.646178	-0.619655
C	2.618742	-0.859884	-1.555185
C	2.514945	-1.969814	0.738119
O	3.891099	-2.451180	0.595609
H	3.873801	-3.262118	0.050762
C	2.377959	-0.990576	-3.028482
H	3.624991	-0.529695	-1.266009
H	1.418403	-2.531235	-1.117556
C	-2.577513	1.759999	-0.578568
H	3.006053	-1.824614	-3.398792
H	2.686332	-0.097588	-3.592009
H	1.327131	-1.226641	-3.258694
C	-3.184013	0.294479	4.645726
C	-4.193216	-0.115053	3.759533
C	-3.933617	-0.199222	2.381956
C	-1.638328	0.532369	2.782962
C	-1.907201	0.619546	4.156692
H	-3.390239	0.355409	5.718847
H	-5.186662	-0.373513	4.139092
H	-4.721579	-0.524611	1.695684
H	-0.640357	0.774825	2.400106
H	-1.118038	0.933967	4.846961
C	-3.051778	4.304588	-1.677754
C	-3.457320	4.020034	-0.364464
C	-3.224465	2.749653	0.188662
C	-2.162796	2.050828	-1.896910
C	-2.406518	3.318328	-2.444262
H	-3.236679	5.295125	-2.105025
H	-3.959135	4.786366	0.234574
H	-3.544535	2.531546	1.212070
H	-1.654201	1.284179	-2.492222
H	-2.090256	3.538070	-3.468916
C	-5.495261	-2.735180	-1.779720
C	-5.721262	-1.349690	-1.742035
C	-4.753843	-0.490132	-1.196805
C	-3.323751	-2.415619	-0.731934
C	-4.297118	-3.267504	-1.273902
H	-6.250808	-3.400665	-2.208819
H	-6.651820	-0.933239	-2.140170
H	-4.930959	0.589742	-1.175793
H	-2.383497	-2.826348	-0.346738
H	-4.116775	-4.346495	-1.308041
O	2.030378	1.113221	-1.218820
C	2.324235	2.188963	-2.153566
H	2.555460	1.301210	-0.354138
C	3.816725	2.444362	-2.356702
H	1.820992	3.095735	-1.768795
H	1.821794	1.916013	-3.097026
H	4.328035	1.570769	-2.794434
H	4.302343	2.685697	-1.397936
H	3.959348	3.296487	-3.042462
C	1.708162	-2.975748	1.563503

H	2.650299	-1.034490	1.308306
H	0.710507	-2.574319	1.809731
H	1.558600	-3.915738	1.000657
O	5.419955	-0.252260	0.272508
C	6.833725	-0.263542	0.577851
H	7.169270	0.781573	0.470001
C	7.614601	-1.179898	-0.361374
H	6.992915	-0.557927	1.634030
H	7.299249	-2.231734	-0.248993
H	7.467844	-0.880961	-1.412066
H	8.693318	-1.131364	-0.135748
H	5.031869	-1.157938	0.425900
O	3.566035	1.535200	0.822920
C	3.348484	1.933977	2.192236
H	2.382171	2.466821	2.203108
C	4.465565	2.839097	2.708128
H	3.240625	1.039520	2.839204
H	5.434924	2.312335	2.714526
H	4.562996	3.736918	2.076611
H	4.253343	3.162587	3.741488
H	4.357045	0.911515	0.747336
H	2.237670	-3.213183	2.499041

SCF(BP86) = -1574.14603896
H 0K = -1573.500585
H 298K = -1573.456088
G 298K = -1573.589492
Solvent correction(toluene) = -0.02876173
BP86-D3 correction = -0.11229229
Lowest frequencies = -159.4833 cm-1, 3.0284 cm-1

IIb$^{3(i)}$

78

P	-2.552446	0.114532	0.009902
C	-3.290084	-0.315595	1.651106
C	-3.195463	1.797988	-0.415239
Au	-0.197073	0.008455	-0.011713
C	1.928836	-0.122986	0.009689
C	2.441546	-0.811465	-1.253040
C	2.521488	1.253862	0.224098
O	4.072096	1.071148	0.318495
H	4.563283	1.932516	0.021953
C	1.890802	-2.215993	-1.487309
H	2.294819	-0.166550	-2.143448
H	2.164361	-0.764732	0.881996
C	-3.337244	-1.046120	-1.201425
H	2.354831	-2.698271	-2.363719
H	0.806431	-2.159155	-1.675245
H	2.068058	-2.838562	-0.594982
C	-4.124843	4.349513	-1.153242
C	-4.793827	3.617987	-0.158843
C	-4.334380	2.343526	0.211631
C	-2.519711	2.539937	-1.408790
C	-2.988532	3.809006	-1.778971
H	-4.485896	5.342826	-1.437931
H	-5.676361	4.038785	0.333249
H	-4.856781	1.778892	0.990193
H	-1.627228	2.120691	-1.887666
H	-2.462986	4.378900	-2.551901
C	-4.481450	-2.891934	-2.990017
C	-4.965653	-1.575658	-2.933025
C	-4.398512	-0.650814	-2.040186
C	-2.847750	-2.369563	-1.265084
C	-3.423580	-3.288755	-2.153706
H	-4.925943	-3.608970	-3.687374
H	-5.788441	-1.263717	-3.584084
H	-4.778883	0.374510	-1.999816
H	-2.020619	-2.678806	-0.615700
H	-3.043275	-4.314321	-2.196428
C	-4.370781	-0.892904	4.183103
C	-5.081428	-1.235840	3.021225
C	-4.545749	-0.950090	1.755209
C	-2.575034	0.020420	2.820670
C	-3.118070	-0.264920	4.082178
H	-4.790194	-1.121562	5.167905
H	-6.054503	-1.731140	3.098011
H	-5.098092	-1.228453	0.852022
H	-1.591867	0.497572	2.739023
H	-2.558515	-0.004946	4.986279
O	3.962052	-0.927914	-1.104673
C	4.689927	-1.052069	-2.359447
H	4.237748	0.231853	-0.327026
C	6.174984	-1.203633	-2.059295
H	4.488280	-0.159295	-2.985604
H	4.306977	-1.934879	-2.899611
H	6.365787	-2.099759	-1.447696
H	6.576231	-0.325701	-1.525099
H	6.735725	-1.309026	-3.002440
C	2.129867	1.978437	1.503059
H	2.384483	1.900572	-0.662458

H	1.066569	2.262328	1.452765
H	2.277608	1.325798	2.378462
O	5.201442	3.296045	-0.274532
C	6.060731	3.853453	0.771605
H	6.099381	4.948823	0.636338
C	7.453487	3.236218	0.779409
H	5.520980	3.647306	1.709129
H	7.409837	2.146752	0.941807
H	7.989276	3.432932	-0.166071
H	8.054517	3.676692	1.592444
H	5.642343	3.432527	-1.134963
O	3.516558	-2.707298	1.342024
C	4.319241	-3.619929	2.108371
H	5.202302	-3.102806	2.540544
C	4.762821	-4.844026	1.305368
H	3.681371	-3.927603	2.954235
H	3.890441	-5.379040	0.895505
H	5.417086	-4.555451	0.462425
H	5.332735	-5.544646	1.939709
H	4.053925	-2.381652	0.591180
H	2.722663	2.898703	1.635727

SCF(BP86) = -1574.15559097
H 0K = -1573.509458
H 298K = -1573.464695
G 298K = -1573.601004
Solvent correction(toluene) = -0.03081511
BP86-D3 correction = -0.10374236
Lowest frequencies = 4.0897 cm-1, 10.3140 cm-1

IIa3(i)

78

P	-2.649323	-0.086096	0.129188
C	-3.694871	-0.698807	-1.270018
C	-2.949265	-1.255014	1.533602
Au	-0.361395	0.115136	-0.425315
C	1.700799	0.306182	-0.962675
C	2.254657	1.546283	-0.254287
C	2.473272	-0.990396	-0.689536
O	3.798934	-0.988078	-1.375117
H	3.645813	-1.265779	-2.298990
C	1.514216	2.850043	-0.570185
H	2.253984	1.376388	0.837267
H	1.669123	0.474510	-2.059920
C	-3.411932	1.522257	0.642361
H	2.060647	3.713669	-0.156829
H	0.509018	2.826041	-0.120200
H	1.385105	2.994353	-1.656219
C	-3.343413	-2.972764	3.727656
C	-4.273699	-2.950459	2.676102
C	-4.081682	-2.093804	1.579884
C	-2.011210	-1.286150	2.588725
C	-2.213032	-2.139047	3.683897
H	-3.496472	-3.642998	4.579389
H	-5.152582	-3.602233	2.705753
H	-4.807429	-2.083241	0.760651
H	-1.124457	-0.643057	2.546762
H	-1.484170	-2.157492	4.500491
C	-4.540842	4.007760	1.325265
C	-4.823233	2.870962	2.098794
C	-4.263340	1.627484	1.760541
C	-3.124464	2.669186	-0.130341
C	-3.692792	3.905379	0.208927
H	-4.978863	4.974526	1.592712
H	-5.482074	2.948178	2.969510
H	-4.486064	0.744246	2.367117
H	-2.459391	2.590461	-0.998077
H	-3.469844	4.790309	-0.395634
C	-5.234127	-1.716731	-3.393784
C	-5.786320	-0.768833	-2.517020
C	-5.021819	-0.258113	-1.455554
C	-3.140535	-1.645847	-2.157973
C	-3.911508	-2.155245	-3.213235
H	-5.832513	-2.109649	-4.221784
H	-6.814683	-0.421903	-2.659252
H	-5.453446	0.486518	-0.779242
H	-2.104939	-1.977498	-2.021954
H	-3.476926	-2.888931	-3.899586
O	3.730921	1.802619	-0.471697
C	4.175351	2.075640	-1.827232
H	4.529387	1.162744	0.397920
C	5.697532	2.129662	-1.849862
H	3.742295	3.040993	-2.142666
H	3.802349	1.281179	-2.495263
H	6.137564	1.157088	-1.573297
H	6.081032	2.908008	-1.169398
H	6.041618	2.377290	-2.867296

C	1.754751	-2.282453	-1.092064
H	2.751370	-1.037712	0.380096
H	0.856399	-2.442953	-0.475123
H	1.423652	-2.231709	-2.146333
O	6.005693	-1.288480	-0.024484
C	6.298947	-2.558443	0.628454
H	7.100989	-2.340654	1.352504
C	6.749934	-3.601100	-0.387035
H	5.413460	-2.904371	1.195437
H	5.948796	-3.827801	-1.111090
H	7.634639	-3.250393	-0.941592
H	7.011745	-4.540925	0.126982
H	5.196836	-1.384509	-0.626271
O	5.114057	0.656547	1.193331
C	6.078022	1.562707	1.832136
H	6.936322	1.711244	1.152924
C	6.510564	0.998960	3.176453
H	5.552677	2.524259	1.945570
H	5.643781	0.850253	3.839386
H	7.037439	0.037186	3.061617
H	7.204544	1.702789	3.664895
H	5.584638	-0.226375	0.746189
H	2.422053	-3.153040	-0.973331

SCF(BP86) = -1574.16427007
H 0K = -1573.518301
H 298K = -1573.474745
G 298K = -1573.604426
Solvent correction(toluene) = -0.02882999
BP86-D3 correction = -0.11084027
Lowest frequencies = 4.9350 cm-1, 12.3973 cm-1

IIb$^{3(ii)}$

78

P	-2.404954	0.049841	0.085659
C	-2.789076	0.418595	1.860502
C	-2.890797	1.574775	-0.848777
Au	-0.160566	-0.611997	-0.249738
C	1.836660	-1.283063	-0.545750
C	2.672786	-1.101952	0.723072
C	2.472384	-0.673331	-1.798411
O	2.388987	0.806393	-1.714171
H	2.570863	1.146549	-2.612604
C	2.111473	-1.788625	1.969904
H	2.805925	-0.028485	0.939519
H	1.735661	-2.370117	-0.743482
C	-3.620936	-1.247316	-0.430828
H	1.172269	-1.293275	2.263164
H	2.817073	-1.718310	2.814720
H	1.875680	-2.850320	1.786279
C	-3.612733	3.833021	-2.366425
C	-4.069567	3.698011	-1.045794
C	-3.712951	2.571837	-0.285403
C	-2.425578	1.718571	-2.174174
C	-2.791609	2.841488	-2.930381
H	-3.894115	4.711365	-2.955938
H	-4.707368	4.469719	-0.603097
H	-4.070674	2.471674	0.744136
H	-1.778859	0.948585	-2.609867
H	-2.432918	2.944744	-3.959607
C	-5.423085	-3.285748	-1.147275
C	-5.778780	-1.938814	-1.325068
C	-4.882587	-0.918170	-0.968568
C	-3.263315	-2.602539	-0.261394
C	-4.165655	-3.616737	-0.614881
H	-6.123567	-4.078119	-1.429382
H	-6.755671	-1.679086	-1.745114
H	-5.160972	0.130159	-1.116140
H	-2.276251	-2.857394	0.141259
H	-3.883402	-4.665967	-0.481907
C	-3.289820	1.047117	4.558280
C	-4.253706	0.356776	3.806310
C	-4.008552	0.042363	2.459350
C	-1.818690	1.105949	2.622171
C	-2.072719	1.422999	3.964591
H	-3.484226	1.288157	5.608134
H	-5.200673	0.058834	4.267456
H	-4.761489	-0.499704	1.878724
H	-0.865623	1.387943	2.159797
H	-1.317581	1.957269	4.550157
C	1.809657	-1.131295	-3.096897
H	3.554617	-0.924009	-1.832225
H	1.921193	-2.221374	-3.219562
H	0.732626	-0.894087	-3.085479
O	4.108230	-1.527394	0.496806
C	4.395140	-2.949085	0.541264
H	4.269670	-3.322595	1.573819
C	5.815047	-3.192338	0.047823
H	3.664376	-3.468884	-0.104344
H	5.954150	-2.815458	-0.978827
H	6.559190	-2.718779	0.710859

H	6.023484	-4.274731	0.043790
H	5.038011	-0.485179	0.753012
O	5.571779	0.444631	0.854758
C	6.790933	0.467199	0.033394
H	7.284029	-0.505078	0.186937
C	7.682213	1.616237	0.475774
H	6.506704	0.549047	-1.031735
H	7.193116	2.592413	0.322140
H	7.947881	1.518909	1.539843
H	8.611800	1.610725	-0.116821
H	4.846870	1.221684	0.511406
O	3.841154	1.993659	0.099999
C	3.950498	3.437518	-0.039108
H	4.709076	3.751036	0.697133
C	2.614595	4.121141	0.227063
H	4.335289	3.683534	-1.047515
H	1.846759	3.795628	-0.494264
H	2.256884	3.894928	1.243979
H	2.727356	5.214310	0.133169
H	3.268531	1.573267	-0.638386
H	2.274762	-0.655230	-3.980020

SCF(BP86) = -1574.16271375
H 0K = -1573.516996
H 298K = -1573.473338
G 298K = -1573.604054
Solvent correction(toluene) = -0.02990826
BP86-D3 correction = -0.11069977
Lowest frequencies = 4.7426 cm-1, 10.2879 cm-1

IIa3(ii)

78

P	2.292258	0.145924	0.026878
C	3.509775	-1.223667	-0.236644
C	2.973894	1.602304	-0.892300
Au	0.105264	-0.459321	-0.641858
C	-1.826991	-1.053735	-1.300009
C	-2.520817	-2.013831	-0.323512
C	-2.650681	0.153628	-1.774940
O	-4.064010	-0.186852	-2.078731
H	-4.079715	-0.956939	-2.679333
C	-1.825161	-3.376539	-0.274933
H	-3.584160	-2.151089	-0.609446
H	-1.619272	-1.666721	-2.203438
C	2.426008	0.586519	1.822170
H	-1.786540	-3.799066	-1.292336
H	-2.356290	-4.106264	0.356662
H	-0.789619	-3.269100	0.088760
C	3.932302	3.868953	-2.258368
C	4.804500	2.812630	-1.950227
C	4.330769	1.680025	-1.267956
C	2.098500	2.663193	-1.210749
C	2.579081	3.794048	-1.887014
H	4.305057	4.748104	-2.793272
H	5.857734	2.866240	-2.243310
H	5.013009	0.856172	-1.036052
H	1.040759	2.596585	-0.930971
H	1.895860	4.613533	-2.132117
C	2.598646	1.148414	4.575262
C	3.326566	1.917676	3.653520
C	3.244668	1.639495	2.278905
C	1.687550	-0.179118	2.751270
C	1.780476	0.098788	4.123013
H	2.667261	1.366997	5.645627
H	3.963082	2.736943	4.002642
H	3.813816	2.242266	1.564257
H	1.044325	-0.992242	2.395706
H	1.212436	-0.502898	4.840007
C	5.335084	-3.303025	-0.746564
C	5.476580	-2.509963	0.403608
C	4.568250	-1.470295	0.661862
C	3.365527	-2.028133	-1.388330
C	4.280084	-3.060720	-1.642620
H	6.044137	-4.113461	-0.942689
H	6.295218	-2.699874	1.104989
H	4.679583	-0.858204	1.562447
H	2.535958	-1.844560	-2.080863
H	4.164588	-3.680707	-2.537285
O	-2.541705	-1.403263	1.037382
C	-3.136083	-2.209871	2.111168
H	-3.123675	-0.141609	1.203775
C	-4.590725	-2.610625	1.874294
H	-3.049658	-1.559590	2.996688
H	-2.491119	-3.087937	2.276795
H	-4.697927	-3.340719	1.055995
H	-5.220323	-1.734324	1.647264
H	-4.985522	-3.082493	2.789779

C	-2.052350	0.842510	-3.004805
H	-2.773115	0.894657	-0.966542
H	-1.048296	1.237765	-2.779903
H	-1.945869	0.124846	-3.838133
O	-5.552900	0.558384	-0.104255
C	-6.470647	1.643218	-0.441923
H	-6.803486	2.058797	0.523186
C	-7.649673	1.120432	-1.251495
H	-5.925091	2.433726	-0.990688
H	-7.317451	0.708358	-2.219054
H	-8.187976	0.334924	-0.698039
H	-8.353960	1.942483	-1.461611
H	-5.101273	0.202289	-0.946200
O	-3.708721	0.731955	1.477001
C	-2.933408	1.966774	1.637571
H	-1.928671	1.657442	1.966522
C	-3.608846	2.871809	2.656696
H	-2.833864	2.461011	0.653488
H	-4.614800	3.176623	2.323850
H	-3.700135	2.367904	3.631605
H	-3.008854	3.786816	2.793068
H	-4.587143	0.773288	0.768482
H	-2.697313	1.670356	-3.339598

SCF(BP86) = -1574.16194991
H 0K = -1573.516250
H 298K = -1573.472731
G 298K = -1573.602335
Solvent correction(toluene) = -0.02968320
BP86-D3 correction = -0.11395070
Lowest frequencies = 6.2400 cm-1, 8.7593 cm-1

TS(II-III)b3(i)

78

P	-2.501477	0.121257	-0.021290
C	-3.320405	-0.395682	1.550825
C	-3.102344	1.826933	-0.401453
Au	-0.159992	0.012723	0.102585
C	2.006264	-0.284891	0.187304
C	2.554817	-0.725370	-1.171430
C	2.270553	1.041699	0.641512
O	4.402987	1.138921	0.720913
H	4.629019	1.934251	0.152083
C	1.877886	-1.979755	-1.732829
H	2.489625	0.112305	-1.898751
H	2.111623	-1.077569	0.950446
C	-3.174331	-0.984013	-1.340802
H	2.315776	-2.283865	-2.697183
H	0.806473	-1.780947	-1.906216
H	1.976128	-2.809084	-1.012643
C	-3.984414	4.413530	-1.060500
C	-4.709934	3.636391	-0.143323
C	-4.274189	2.343304	0.188754
C	-2.369635	2.612993	-1.317647
C	-2.815355	3.900824	-1.648519
H	-4.326944	5.421568	-1.314193
H	-5.617992	4.035860	0.318887
H	-4.840092	1.742768	0.907622
H	-1.452759	2.215709	-1.768193
H	-2.246154	4.506739	-2.360453
C	-4.171140	-2.739963	-3.296019
C	-4.656716	-1.425116	-3.219899
C	-4.162887	-0.543695	-2.243695
C	-2.682834	-2.305942	-1.421496
C	-3.185879	-3.180445	-2.395113
H	-4.558115	-3.422529	-4.059012
H	-5.422921	-1.080144	-3.921155
H	-4.543327	0.480787	-2.188010
H	-1.913264	-2.650662	-0.721192
H	-2.805164	-4.204924	-2.453088
C	-4.549993	-1.104152	3.976838
C	-5.168024	-1.424018	2.756988
C	-4.557920	-1.072918	1.542121
C	-2.697940	-0.081187	2.777956
C	-3.315730	-0.432610	3.986931
H	-5.027182	-1.384327	4.921068
H	-6.126439	-1.952348	2.747913
H	-5.037806	-1.332490	0.593280
H	-1.730070	0.432657	2.783927
H	-2.829076	-0.189799	4.936652
O	3.991699	-0.981713	-0.932216
C	4.789312	-1.128027	-2.134565
H	4.493029	0.343128	0.128652
C	6.255527	-1.219678	-1.732120
H	4.606630	-0.257297	-2.798210
H	4.479494	-2.037258	-2.681431
H	6.433033	-2.090706	-1.080886
H	6.584360	-0.314250	-1.195301
H	6.883811	-1.333852	-2.630556
C	2.032509	1.486878	2.060817
H	2.319014	1.837255	-0.113704

H	1.093379	2.064470	2.129310
H	1.973289	0.628307	2.747324
O	4.938738	3.421133	-0.653910
C	5.514823	4.366484	0.293868
H	5.625978	5.349869	-0.199878
C	6.837422	3.877204	0.876086
H	4.750063	4.474646	1.079925
H	6.708471	2.897931	1.365787
H	7.611246	3.781030	0.093727
H	7.215716	4.592523	1.625679
H	5.574121	3.335931	-1.390714
O	3.230773	-3.060775	1.047370
C	3.999393	-3.948730	1.876030
H	4.705657	-3.379429	2.516257
C	4.750466	-5.009645	1.069599
H	3.267707	-4.427300	2.549119
H	4.052444	-5.591125	0.445147
H	5.504765	-4.549278	0.405964
H	5.282095	-5.707901	1.738808
H	3.847135	-2.564797	0.466232
H	2.849356	2.147241	2.387498

SCF(BP86) = -1574.14918697
H 0K = -1573.503587
H 298K = -1573.458495
G 298K = -1573.595359
Solvent correction(toluene) = -0.02955805
BP86-D3 correction = -0.10462763
Lowest frequencies = -128.3117 cm-1, 2.7547 cm-1

TS(II-III)a3(i)

78

P	-2.602690	-0.045559	0.196350
C	-3.813233	-0.442812	-1.139780
C	-2.837032	-1.306065	1.526427
Au	-0.387556	0.012129	-0.575700
C	1.662788	0.267393	-1.343813
C	2.304174	1.415735	-0.541168
C	2.045804	-1.081895	-1.088208
O	4.036497	-1.208154	-1.729517
H	4.171855	-2.024539	-2.248089
C	1.595924	2.761086	-0.758827
H	2.265538	1.158724	0.533339
H	1.483557	0.497094	-2.408169
C	-3.128323	1.578238	0.904578
H	2.155436	3.568338	-0.259462
H	0.579326	2.728502	-0.330695
H	1.498985	3.009719	-1.829146
C	-3.148476	-3.172651	3.604683
C	-4.160417	-3.015843	2.643972
C	-4.010497	-2.083933	1.604106
C	-1.816871	-1.470228	2.489225
C	-1.977922	-2.398422	3.528055
H	-3.269448	-3.901663	4.411952
H	-5.070792	-3.620630	2.700294
H	-4.800238	-1.967215	0.855571
H	-0.900849	-0.871738	2.424146
H	-1.186748	-2.521519	4.274242
C	-3.923575	4.084032	1.898967
C	-4.188264	2.919581	2.636629
C	-3.794263	1.664340	2.143696
C	-2.858675	2.751329	0.165175
C	-3.260936	3.999075	0.661919
H	-4.231231	5.059631	2.287899
H	-4.703375	2.983906	3.600098
H	-4.002324	0.759149	2.722144
H	-2.341237	2.686993	-0.798962
H	-3.053299	4.905809	0.085250
C	-5.639563	-1.133250	-3.161562
C	-6.012971	-0.223303	-2.159156
C	-5.104508	0.125355	-1.146914
C	-3.438158	-1.352331	-2.151898
C	-4.352793	-1.697921	-3.157252
H	-6.349762	-1.398401	-3.950887
H	-7.013094	0.221032	-2.164537
H	-5.396222	0.841251	-0.372126
H	-2.430997	-1.784272	-2.152455
H	-4.058317	-2.401908	-3.941760
O	3.734407	1.525287	-0.770564
C	4.180078	1.902724	-2.094240
H	4.682319	1.290677	0.654243
C	5.702861	1.886898	-2.090138
H	3.797018	2.909723	-2.348337
H	3.794572	1.175290	-2.831177
H	6.076292	0.880220	-1.843854
H	6.106665	2.608322	-1.360080
H	6.084391	2.165146	-3.086184

C	1.562777	-2.252088	-1.916069
H	2.375141	-1.317504	-0.067888
H	0.584269	-2.602376	-1.543447
H	1.442386	-1.974088	-2.975452
O	5.704142	-1.341683	0.212095
C	5.724326	-2.508066	1.059720
H	6.462712	-2.352377	1.869006
C	6.109133	-3.725915	0.227937
H	4.737015	-2.663407	1.543723
H	5.366623	-3.917952	-0.566677
H	7.092084	-3.577243	-0.246925
H	6.160276	-4.626007	0.862237
H	4.685571	-1.286246	-0.917877
O	5.089838	0.875641	1.464105
C	6.101678	1.763393	1.993802
H	6.885234	1.957094	1.233624
C	6.711599	1.128258	3.236126
H	5.634605	2.734496	2.248961
H	5.936215	0.932266	3.994430
H	7.208923	0.174942	2.989948
H	7.466407	1.800032	3.676659
H	5.552921	-0.512469	0.781812
H	2.251818	-3.106624	-1.835215

SCF(BP86) = -1574.15040234
H 0K = -1573.505252
H 298K = -1573.460785
G 298K = -1573.595450
Solvent correction(toluene) = -0.02910915
BP86-D3 correction = -0.10857184
Lowest frequencies = -130.2566 cm-1, 3.5247 cm-1

TS(II-III)b[3(ii)]

78

P	-2.293075	0.023781	0.045617
C	-2.659465	0.357840	1.826184
C	-2.739038	1.563091	-0.875153
Au	-0.080056	-0.646656	-0.277768
C	1.966204	-1.426544	-0.464310
C	2.673318	-1.375844	0.904630
C	2.608027	-0.787878	-1.572217
O	1.869567	1.144241	-1.695893
H	2.262987	1.585607	-2.476857
C	1.996980	-2.257529	1.960533
H	2.684171	-0.331009	1.261099
H	1.589070	-2.429922	-0.751844
C	-3.495484	-1.268783	-0.501821
H	0.993942	-1.861370	2.192415
H	2.591522	-2.266086	2.887913
H	1.867857	-3.297241	1.614007
C	-3.434948	3.843117	-2.365376
C	-3.902228	3.694676	-1.049763
C	-3.558258	2.556936	-0.301347
C	-2.261978	1.718732	-2.194826
C	-2.615867	2.854377	-2.937348
H	-3.705906	4.731215	-2.944895
H	-4.537313	4.465069	-0.601315
H	-3.923264	2.445527	0.724370
H	-1.613955	0.952902	-2.635210
H	-2.248196	2.970226	-3.961863
C	-5.302166	-3.286465	-1.255456
C	-5.627690	-1.937195	-1.468136
C	-4.728910	-0.925277	-1.093228
C	-3.167113	-2.626343	-0.295745
C	-4.072458	-3.630351	-0.668399
H	-6.004495	-4.071550	-1.552499
H	-6.582784	-1.668075	-1.929931
H	-4.983745	0.124852	-1.266833
H	-2.202464	-2.891887	0.151735
H	-3.814477	-4.681736	-0.507288
C	-3.161631	0.937334	4.531085
C	-4.140660	0.293924	3.757210
C	-3.894972	0.002596	2.405394
C	-1.673767	0.999078	2.607782
C	-1.929056	1.290770	3.955556
H	-3.356309	1.158841	5.585079
H	-5.099089	0.013447	4.205081
H	-4.658261	-0.504954	1.807396
H	-0.709376	1.264278	2.159363
H	-1.162691	1.787115	4.559225
C	2.345451	-1.235574	-2.981058
H	3.586691	-0.320808	-1.397940
H	3.000630	-2.104453	-3.186164
H	1.301503	-1.554418	-3.124971
O	4.108516	-1.666420	0.810693
C	4.479492	-3.000849	0.400430
H	4.135121	-3.730644	1.156976
C	5.996107	-3.049292	0.262855
H	3.990199	-3.254989	-0.561645
H	6.346587	-2.341312	-0.506984
H	6.483807	-2.801358	1.219584

H	6.317366	-4.061219	-0.032933
H	4.994523	-0.262554	0.373788
O	5.269839	0.621515	-0.007043
C	6.621190	0.932147	0.410726
H	6.653425	1.102919	1.505206
C	7.103599	2.165443	-0.340857
H	7.275986	0.068123	0.188601
H	7.080586	1.994312	-1.429388
H	6.476971	3.044123	-0.111726
H	8.139249	2.406356	-0.050407
H	4.007411	1.632913	0.290127
O	3.098261	2.078056	0.355533
C	3.264625	3.498327	0.578066
H	3.863900	3.647474	1.496186
C	1.896427	4.150308	0.726580
H	3.823339	3.956249	-0.263725
H	1.289192	4.015576	-0.184177
H	1.348302	3.715180	1.577923
H	2.008665	5.232414	0.904738
H	2.326625	1.594733	-0.872844
H	2.599430	-0.468557	-3.728232

SCF(BP86) = -1574.14553991
H 0K = -1573.500555
H 298K = -1573.456233
G 298K = -1573.587799
Solvent correction(toluene) = -0.02922881
BP86-D3 correction = -0.11229056
Lowest frequencies = -188.2635 cm-1, 6.0615 cm-1

TS(II-III)a3(ii)

78

P	2.323030	0.141417	-0.021216
C	3.572182	-1.220486	-0.016448
C	2.856043	1.336594	-1.328661
Au	0.153221	-0.677564	-0.386351
C	-1.794501	-1.593043	-0.718930
C	-2.462791	-1.949415	0.617424
C	-2.364350	-0.592684	-1.594445
O	-4.223631	-1.081559	-1.990539
H	-4.308871	-2.033548	-1.786019
C	-1.758520	-3.136095	1.287566
H	-3.520364	-2.231050	0.416599
H	-1.534286	-2.519420	-1.263142
C	2.516120	1.027628	1.589507
H	-1.729935	-3.993909	0.594994
H	-2.281071	-3.471150	2.197206
H	-0.720058	-2.870973	1.549410
C	3.598052	3.211299	-3.289016
C	4.505953	2.219468	-2.885998
C	4.140669	1.281320	-1.906094
C	1.941854	2.331372	-1.740241
C	2.316511	3.267992	-2.714029
H	3.886436	3.938542	-4.054369
H	5.503071	2.171549	-3.334678
H	4.850659	0.508052	-1.597054
H	0.940209	2.370866	-1.296667
H	1.606155	4.038902	-3.029052
C	2.819299	2.280362	4.088365
C	3.501059	2.777981	2.966155
C	3.353559	2.155292	1.715990
C	1.824534	0.534576	2.717109
C	1.982120	1.158453	3.963283
H	2.937101	2.769047	5.060613
H	4.150334	3.654000	3.060849
H	3.883198	2.548525	0.842614
H	1.164084	-0.333933	2.615316
H	1.447474	0.771559	4.836592
C	5.456402	-3.305426	-0.102544
C	5.641547	-2.227249	0.777998
C	4.703724	-1.183112	0.824497
C	3.384277	-2.309327	-0.895681
C	4.328473	-3.345492	-0.939733
H	6.188655	-4.118286	-0.132855
H	6.516882	-2.197417	1.434326
H	4.848647	-0.348060	1.517045
H	2.498161	-2.343967	-1.539970
H	4.179412	-4.187758	-1.622570
O	-2.480873	-0.771538	1.473314
C	-3.132036	-0.939436	2.767183
H	-3.050279	0.783564	0.934616
C	-4.592524	-1.387353	2.696661
H	-3.055416	0.061114	3.225220
H	-2.532274	-1.628158	3.389608
H	-4.693378	-2.443932	2.397848
H	-5.169183	-0.770312	1.987384
H	-5.049214	-1.288102	3.695812
C	-1.898018	-0.462521	-3.025871

H	-2.710940	0.341466	-1.134983
H	-1.033347	0.221777	-3.075603
H	-1.589245	-1.433858	-3.445084
O	-5.619097	0.234990	-0.317318
C	-6.747267	0.944280	-0.897415
H	-7.241934	1.528640	-0.099595
C	-7.711422	-0.069321	-1.497745
H	-6.392171	1.654126	-1.669881
H	-7.227732	-0.642788	-2.306369
H	-8.067869	-0.773823	-0.728929
H	-8.586334	0.446716	-1.925352
H	-4.831126	-0.571278	-1.299301
O	-3.505270	1.576654	0.530196
C	-3.332482	2.739691	1.375860
H	-3.831100	2.577921	2.352625
C	-3.922449	3.954343	0.672711
H	-2.253252	2.890692	1.567906
H	-3.426300	4.125043	-0.296544
H	-5.002933	3.824793	0.493703
H	-3.788611	4.854994	1.293945
H	-4.951091	0.895990	0.053255
H	-2.697232	-0.036343	-3.649467

SCF(BP86) = -1574.15536004
H 0K = -1573.508914
H 298K = -1573.465115
G 298K = -1573.594890
Solvent correction(toluene) = -0.02892781
BP86-D3 correction = -0.11201777
Lowest frequencies = -153.6520 cm-1, 5.3075 cm-1

IIIb3(i)

78

P	2.435219	-0.029347	-0.052109
C	3.324805	-0.323654	1.537389
C	3.014454	-1.313520	-1.242193
Au	0.100885	-0.052549	0.228945
C	-2.101427	0.437390	0.312486
C	-2.671378	0.643480	-1.089271
C	-2.024570	-0.824473	0.903624
O	-4.878539	-1.428987	0.895017
H	-4.551161	-2.127701	0.278991
C	-2.067442	1.844500	-1.824873
H	-2.546823	-0.286530	-1.684896
H	-2.158791	1.332891	0.954918
C	2.992500	1.607914	-0.694058
H	-2.512880	1.969053	-2.824870
H	-0.980882	1.702146	-1.960939
H	-2.235203	2.761881	-1.236558
C	3.885335	-3.212345	-3.119902
C	4.668422	-2.934903	-1.987780
C	4.238272	-1.986595	-1.045735
C	2.224969	-1.597860	-2.377565
C	2.664854	-2.543207	-3.315038
H	4.223521	-3.954868	-3.849249
H	5.616740	-3.458626	-1.832989
H	4.847675	-1.777242	-0.161150
H	1.269716	-1.081190	-2.525082
H	2.051615	-2.762410	-4.194552
C	3.834539	4.131398	-1.594587
C	4.432516	2.968300	-2.105903
C	4.015975	1.703769	-1.659226
C	2.387433	2.778032	-0.183959
C	2.813640	4.036150	-0.633145
H	4.160836	5.114186	-1.948576
H	5.225099	3.041729	-2.856903
H	4.481056	0.799241	-2.063075
H	1.586817	2.705383	0.561302
H	2.343402	4.941350	-0.236929
C	4.660975	-0.848277	3.951604
C	5.101787	0.190968	3.117105
C	4.438084	0.457719	1.908262
C	2.879234	-1.366222	2.379444
C	3.551206	-1.627912	3.581557
H	5.179398	-1.049514	4.894116
H	5.963939	0.800111	3.405512
H	4.781763	1.271296	1.262294
H	2.011616	-1.971921	2.093839
H	3.204724	-2.436256	4.232812
O	-4.096046	0.831093	-0.835776
C	-4.939345	0.720415	-2.012762
H	-4.725189	-0.593619	0.402829
C	-6.388544	0.658385	-1.548678
H	-4.658544	-0.199170	-2.567188
H	-4.768564	1.584295	-2.682473
H	-6.675637	1.582998	-1.022075
H	-6.541078	-0.194684	-0.867155
H	-7.055605	0.541796	-2.418479
C	-1.992591	-1.071750	2.384760
H	-2.191610	-1.707478	0.268268

H	-1.314439	-1.895497	2.660851
H	-1.740081	-0.169465	2.962683
O	-3.730529	-3.595276	-0.516452
C	-4.120074	-4.687371	0.363738
H	-3.639630	-5.624713	0.022566
C	-5.635123	-4.839685	0.464942
H	-3.686028	-4.425219	1.342085
H	-6.095920	-3.901963	0.816084
H	-6.077981	-5.108691	-0.511043
H	-5.894431	-5.643261	1.174896
H	-4.111757	-3.794466	-1.393281
O	-3.429256	3.152260	0.840963
C	-4.212876	4.044617	1.653739
H	-4.817217	3.476263	2.390897
C	-5.110956	4.962523	0.823439
H	-3.479971	4.639520	2.225202
H	-4.514316	5.542233	0.100067
H	-5.866206	4.383189	0.262615
H	-5.653600	5.670693	1.472977
H	-4.041164	2.558782	0.352550
H	-3.024510	-1.382379	2.639564

SCF(BP86) = -1574.15254856
H 0K = -1573.507275
H 298K = -1573.460937
G 298K = -1573.600598
Solvent correction(toluene) = -0.03092116
BP86-D3 correction = -0.10511258
Lowest frequencies = 1.5956 cm-1, 10.9616 cm-1

IIIa[3(i)]

78

P	-2.609065	-0.187045	0.003182
C	-3.245736	-1.473731	-1.154810
C	-2.925408	-0.789756	1.716527
Au	-0.336258	0.274643	-0.371052
C	1.709317	1.241909	-0.649861
C	2.112304	1.908611	0.674950
C	1.915230	-0.114323	-0.910718
O	4.902738	0.182293	-2.439777
H	5.557840	0.505971	-3.082291
C	1.476536	3.292837	0.849653
H	1.795441	1.258005	1.510190
H	1.557494	1.911062	-1.511600
C	-3.631438	1.328085	-0.246158
H	1.836167	3.759198	1.779762
H	0.377992	3.200004	0.906233
H	1.709065	3.965541	0.007571
C	-3.415969	-1.627727	4.352045
C	-4.125508	-2.207362	3.287957
C	-3.884977	-1.792219	1.968235
C	-2.208331	-0.211714	2.786676
C	-2.458947	-0.629213	4.101484
H	-3.604893	-1.957343	5.378332
H	-4.867914	-2.987538	3.481996
H	-4.435782	-2.249882	1.140749
H	-1.457610	0.562277	2.590259
H	-1.902505	-0.179889	4.929786
C	-5.184060	3.623136	-0.704852
C	-5.469801	2.789423	0.388139
C	-4.697290	1.640160	0.622416
C	-3.341053	2.169947	-1.342793
C	-4.121276	3.312320	-1.570909
H	-5.787374	4.518993	-0.881037
H	-6.295417	3.032111	1.064140
H	-4.920750	0.994544	1.477212
H	-2.510072	1.930575	-2.016445
H	-3.895757	3.962823	-2.421530
C	-4.186401	-3.491651	-2.866468
C	-4.973047	-2.354323	-2.623880
C	-4.508335	-1.341981	-1.768410
C	-2.452965	-2.615950	-1.402556
C	-2.927693	-3.623044	-2.254480
H	-4.551762	-4.275741	-3.536756
H	-5.951797	-2.250053	-3.102095
H	-5.122118	-0.454848	-1.584949
H	-1.469477	-2.717225	-0.929450
H	-2.312270	-4.507502	-2.445609
O	3.551455	1.941141	0.835335
C	4.327249	2.621876	-0.200773
H	4.050548	0.339426	1.280346
C	5.769658	2.684580	0.283893
H	3.916871	3.638089	-0.347807
H	4.268277	2.049702	-1.146867
H	6.206353	1.674682	0.357490
H	5.838614	3.185785	1.262981
H	6.373716	3.258284	-0.438648
C	1.984149	-0.721936	-2.278630
H	2.284022	-0.730504	-0.073548

H	1.627354	-1.763993	-2.290560
H	1.440457	-0.132003	-3.034082
O	6.239233	-0.800497	-0.308064
C	6.878758	-2.078565	-0.489858
H	7.492218	-2.322189	0.401293
C	7.758943	-2.012451	-1.732545
H	6.121587	-2.881843	-0.601516
H	7.147441	-1.797096	-2.625325
H	8.519366	-1.221047	-1.628861
H	8.277852	-2.971639	-1.893020
H	5.447733	-0.195109	-1.686844
O	4.164929	-0.641141	1.439636
C	4.455007	-0.847389	2.842491
H	5.418428	-0.368671	3.108013
C	4.509232	-2.343723	3.120428
H	3.666925	-0.371096	3.458858
H	3.549419	-2.824683	2.869080
H	5.303845	-2.827770	2.528628
H	4.720486	-2.526876	4.186820
H	5.520481	-0.883146	0.383052
H	3.064138	-0.709799	-2.558797

SCF(BP86) = -1574.16245993
H 0K = -1573.516807
H 298K = -1573.471076
G 298K = -1573.607774
Solvent correction(toluene) = -0.02957768
BP86-D3 correction = -0.10589418
Lowest frequencies = 2.2841 cm-1, 9.5209 cm-1

IIIb3(ii)

78

P	-2.217062	0.003589	0.011699
C	-2.427347	0.980119	1.563799
C	-2.922679	1.018775	-1.362983
Au	0.034183	-0.533301	-0.409331
C	1.967619	-1.748859	-0.124924
C	2.709216	-1.215306	1.105612
C	2.055783	-1.141511	-1.376115
O	0.764327	2.065580	-0.628969
H	0.803917	2.407860	-1.540218
C	2.105285	-1.695930	2.428074
H	2.708044	-0.110671	1.083861
H	1.601547	-2.785523	-0.075319
C	-3.296924	-1.486559	0.154642
H	1.105612	-1.248781	2.560225
H	2.738416	-1.378025	3.271340
H	1.988326	-2.792036	2.467218
C	-4.002118	2.485296	-3.504529
C	-4.220772	2.906480	-2.183488
C	-3.684145	2.176982	-1.109255
C	-2.699727	0.598431	-2.692779
C	-3.242942	1.329541	-3.758597
H	-4.421540	3.057602	-4.337795
H	-4.811180	3.806093	-1.983629
H	-3.853365	2.510821	-0.081131
H	-2.108930	-0.303311	-2.891777
H	-3.071566	0.998942	-4.787851
C	-4.927073	-3.755647	0.461092
C	-5.468143	-2.567324	-0.056860
C	-4.658672	-1.430851	-0.211068
C	-2.754319	-2.683137	0.668576
C	-3.571116	-3.812883	0.824555
H	-5.561401	-4.640091	0.575866
H	-6.522832	-2.523392	-0.345690
H	-5.082464	-0.509006	-0.621886
H	-1.692587	-2.726018	0.936855
H	-3.147011	-4.740179	1.222247
C	-2.702802	2.520672	3.893694
C	-3.552289	1.424794	3.673885
C	-3.420188	0.651268	2.509189
C	-1.564857	2.078316	1.784523
C	-1.714275	2.846621	2.948838
H	-2.809473	3.120525	4.803047
H	-4.321723	1.168012	4.408638
H	-4.084654	-0.201480	2.340558
H	-0.790697	2.325382	1.047090
H	-1.051696	3.700776	3.120662
C	1.743841	-1.825293	-2.683744
H	2.620707	-0.200952	-1.443119
H	2.696767	-2.096845	-3.176722
H	1.151786	-2.744081	-2.547085
O	4.130993	-1.522590	0.992772
C	4.512403	-2.920170	1.023145
H	4.141148	-3.384611	1.955453
C	6.032569	-2.987929	0.964846
H	4.062692	-3.461910	0.166994
H	6.416200	-2.557192	0.024930
H	6.480481	-2.442829	1.811028

H	6.363806	-4.037933	1.015786
H	4.802791	-0.318862	-0.156003
O	4.855119	0.453674	-0.782355
C	6.236532	0.701831	-1.133197
H	6.821633	0.965058	-0.229492
C	6.290735	1.830945	-2.153337
H	6.677802	-0.220195	-1.559872
H	5.721563	1.567747	-3.059826
H	5.872312	2.762798	-1.737041
H	7.334595	2.029533	-2.446193
H	3.899949	1.626608	-0.032009
O	3.185348	2.130574	0.463741
C	3.751473	3.317140	1.057707
H	4.600558	3.034470	1.710641
C	2.677337	4.031453	1.867931
H	4.146933	3.990742	0.269775
H	1.829357	4.318761	1.223918
H	2.300096	3.380275	2.673603
H	3.087927	4.946681	2.325045
H	1.699108	2.155829	-0.258123
H	1.211772	-1.155137	-3.379239

SCF(BP86) = -1574.17081231
H 0K = -1573.525411
H 298K = -1573.479592
G 298K = -1573.614241
Solvent correction(toluene) = -0.02860204
BP86-D3 correction = -0.11048905
Lowest frequencies = 7.8765 cm-1, 13.0124 cm-1

IIIa$^{3(ii)}$

78

P	2.393108	0.143851	0.001127
C	3.450526	-1.362206	0.142678
C	3.157243	1.223826	-1.285589
Au	0.184870	-0.440894	-0.555200
C	-1.786018	-1.597259	-0.835869
C	-2.396196	-1.983240	0.496131
C	-1.923665	-0.384034	-1.504426
O	-4.791789	-1.689501	-1.585139
H	-5.467921	-2.385749	-1.659764
C	-1.641327	-3.141090	1.162677
H	-3.421590	-2.301059	0.195206
H	-1.505403	-2.469446	-1.445282
C	2.512857	1.048280	1.603578
H	-1.535855	-3.977100	0.451297
H	-2.180635	-3.530669	2.039748
H	-0.632588	-2.825568	1.479988
C	4.278454	2.934851	-3.210485
C	5.037988	1.878437	-2.683113
C	4.482871	1.019817	-1.720009
C	2.392201	2.283771	-1.820112
C	2.956521	3.138161	-2.777727
H	4.714541	3.598728	-3.963282
H	6.066110	1.717466	-3.021700
H	5.075997	0.194201	-1.314936
H	1.359686	2.439952	-1.486819
H	2.362198	3.958886	-3.191063
C	2.705137	2.340292	4.090149
C	3.576367	2.693672	3.046840
C	3.485114	2.050903	1.801919
C	1.631701	0.699840	2.649983
C	1.734192	1.343845	3.891664
H	2.779070	2.845700	5.058056
H	4.328996	3.473422	3.198930
H	4.161156	2.332970	0.988679
H	0.866918	-0.068635	2.489741
H	1.051654	1.072105	4.702919
C	5.060207	-3.660952	0.272721
C	5.218469	-2.638475	1.221840
C	4.417061	-1.486653	1.161570
C	3.287434	-2.394256	-0.808016
C	4.096145	-3.537767	-0.742854
H	5.685580	-4.557456	0.325886
H	5.966629	-2.734885	2.014661
H	4.541186	-0.693441	1.905138
H	2.531630	-2.301073	-1.596429
H	3.969254	-4.335353	-1.481367
O	-2.493058	-0.816980	1.349588
C	-3.183030	-1.034431	2.618509
H	-3.191479	0.762751	0.850683
C	-4.623266	-1.526144	2.486035
H	-3.152803	-0.041838	3.098855
H	-2.580859	-1.717880	3.245075
H	-4.676827	-2.564267	2.118082
H	-5.215123	-0.886227	1.809783
H	-5.097538	-1.510710	3.482172
C	-1.810279	-0.255239	-3.001165
H	-2.390373	0.463196	-0.981580

H	-1.381307	0.710464	-3.311986
H	-1.235521	-1.076516	-3.458090
O	-5.952268	0.337642	-0.152435
C	-6.980362	1.081049	-0.837847
H	-7.496255	1.755718	-0.124752
C	-7.972237	0.096082	-1.445059
H	-6.536114	1.713472	-1.633050
H	-7.464897	-0.557746	-2.174623
H	-8.424322	-0.534657	-0.661888
H	-8.782047	0.632224	-1.966316
H	-5.255103	-0.959114	-1.086174
O	-3.598534	1.581343	0.459881
C	-3.408493	2.696516	1.361743
H	-3.939929	2.510705	2.317050
C	-3.941111	3.959129	0.697757
H	-2.330658	2.808190	1.591639
H	-3.412091	4.156208	-0.248964
H	-5.018173	3.867281	0.481065
H	-3.800341	4.827690	1.361815
H	-5.193675	0.947041	0.062570
H	-2.844420	-0.306519	-3.393398

SCF(BP86) = -1574.16250404
H 0K = -1573.516947
H 298K = -1573.470991
G 298K = -1573.608064
Solvent correction(toluene) = -0.02964151
BP86-D3 correction = -0.10856954
Lowest frequencies = 5.0885 cm-1, 9.9916 cm-1

Direct S_N2 TS

78

P	-2.502316	-0.003136	0.047908
C	-3.412882	1.574719	-0.256135
C	-3.556190	-1.356748	-0.635788
Au	-0.394945	0.023411	-0.839569
C	-2.463969	-0.233422	1.882948
C	-5.123011	-3.471433	-1.622474
C	-5.728908	-2.255358	-1.267021
C	-4.950781	-1.195684	-0.774212
C	-2.948403	-2.577235	-1.000556
C	-3.733413	-3.631815	-1.489132
H	-5.733109	-4.292792	-2.011015
H	-6.810030	-2.126442	-1.377814
H	-5.425421	-0.245666	-0.509139
H	-1.862534	-2.693292	-0.908799
H	-3.259024	-4.576026	-1.774355
C	-2.372637	-0.493735	4.681383
C	-3.314489	-1.222708	3.938539
C	-3.365009	-1.095108	2.540164
C	-1.511881	0.494718	2.630839
C	-1.472702	0.366220	4.027002
H	-2.338128	-0.595085	5.770651
H	-4.014779	-1.893802	4.445559
H	-4.100274	-1.666144	1.964923
H	-0.807476	1.161566	2.120321
H	-0.739858	0.938627	4.604623
C	-4.825815	3.939632	-0.810179
C	-4.973475	3.309646	0.435896
C	-4.269904	2.127320	0.717944
C	-3.260802	2.212696	-1.506253
C	-3.970311	3.390566	-1.780703
H	-5.373549	4.862684	-1.024101
H	-5.635330	3.739024	1.194434
H	-4.382037	1.643495	1.693270
H	-2.586421	1.788351	-2.258673
H	-3.849727	3.883615	-2.750307
C	2.614218	-2.114074	0.052882
C	2.555217	-1.654020	1.341268
C	3.250808	-1.370113	-0.986727
O	1.536938	0.107496	-1.601615
H	1.470907	0.156095	-2.576483
C	1.935015	-2.387371	2.477791
H	3.035990	-0.688556	1.563701
H	2.193366	-3.096659	-0.198224
H	1.141895	-1.768953	2.940394
H	2.682904	-2.554865	3.276113
H	1.506092	-3.356709	2.180689
C	3.468416	-1.961187	-2.339377
H	3.626032	-0.374782	-0.772649
H	4.276591	-2.712603	-2.268936
H	2.569543	-2.498896	-2.684629
O	5.515904	-1.550599	-0.115931
C	6.667333	-1.350210	-0.955211
H	6.620290	-2.127531	-1.739175
C	7.970763	-1.485020	-0.163579
H	6.621224	-0.363765	-1.460722
H	8.049495	-0.700876	0.608359
H	8.021349	-2.466820	0.333527

H	8.840451	-1.387945	-0.835847
H	5.375775	-0.707769	0.410392
O	4.787047	0.746726	1.133780
C	5.555812	1.595750	2.012246
H	6.300434	0.939371	2.494397
C	4.683433	2.281920	3.062522
H	6.115893	2.348269	1.421438
H	3.925509	2.927972	2.587657
H	4.161949	1.536700	3.685796
H	5.300682	2.913124	3.724443
H	4.149440	1.311151	0.596409
O	2.939443	2.029859	-0.343338
C	3.272225	3.160671	-1.175201
H	2.342915	3.604764	-1.581723
C	4.240321	2.806418	-2.305402
H	3.721322	3.912137	-0.503505
H	5.171906	2.373651	-1.903009
H	3.791510	2.075998	-3.001391
H	4.504721	3.704215	-2.889898
H	2.380209	1.385998	-0.877857
H	3.766157	-1.211359	-3.085569

SCF(BP86) = -1574.13445567
H 0K = -1573.491723
H 298K = -1573.445672
G 298K = -1573.584187
Solvent correction(toluene) = -0.02895928
BP86-D3 correction = -0.10631286
Lowest frequencies = -144.8291 cm-1, 3.9523 cm-1
